# Study on the Action Mechanism of the Yifei Jianpi Tongfu Formula in Treatment of Colorectal Cancer Lung Metastasis Based on Network Analysis, Molecular Docking, and Experimental Validation

**DOI:** 10.1155/2022/6229444

**Published:** 2022-07-30

**Authors:** Wanli Zhu, Rundong Zhang, Chenchao Ma, Yangyang Hu, Xuan Shi, Xiyu Wang, Xing Wu, Kaixing Ai

**Affiliations:** ^1^Department of General Surgery, Shanghai Pulmonary Hospital, Tongji University School of Medicine, Shanghai 200433, China; ^2^Department of Thoracic Surgery, Shanghai Pulmonary Hospital, Tongji University School of Medicine, Shanghai 200433, China; ^3^Department of Anesthesiology, Shanghai Pulmonary Hospital, Tongji University School of Medicine, Shanghai 200433, China; ^4^Department of Oncology, Hospital of Integrated Traditional and Western Medicine of Baoshan District, Shanghai 201999, China; ^5^Department of Cardiovasology, Jingan Hospital of Traditional Chinese Medicine, Shanghai 200072, China

## Abstract

**Objective:**

The lung is the second most common site of colorectal cancer (CRC) metastasis. This study aims to investigate the therapeutic effects and potential action mechanisms of *Yifei Jianpi Tongfu* formula (YJTF) in CRC lung metastasis in a comprehensive and systematic way by network analysis, molecular docking, and experimental verification.

**Methods:**

The main ingredients in YJTF were screened from the Traditional Chinese Medicine System Pharmacology Database and Analysis Platform (TCMSP) and Traditional Chinese Medicine Integrated Database (TCMID), and the disease-related targets from the Online Mendelian Inheritance in Man (OMIM) and GeneCards and the compound-related targets from SwissTargetPrediction were collected. Then, Metascape was used for pathway annotation and enrichment analysis, and meanwhile, a protein-protein interaction (PPI) network was constructed. Molecular docking was carried out to investigate interactions between the active compounds and the potential targets. The in vivo effect of YJTF on CRC lung metastasis was observed in a tail vein injection mouse model.

**Results:**

A total of 243 active compounds and 81 disease-related targets of YJTF were selected for analysis. The results of multiple network analysis showed that the core targets of YJTF were enriched onto various cancer-related pathways, especially focal adhesion and adherens junction. The results of molecular docking demonstrated that all core compounds (quercetin, kaempferol, luteolin, apigenin, and isorhamnetin) were capable of binding with AKT1, EGFR, SRC, ESR1, and PTGS2. Experimental validation in vivo demonstrated that YJTF combined with oxaliplatin could significantly reduce the number of lung metastases and improve the quality of life in mice. Further research suggested that YJTF inhibited CRC lung metastasis probably by modulating epithelial-to-mesenchymal transition (EMT).

**Conclusions:**

According to the analysis, YJTF can be considered as an effective adjuvant therapy for CRC lung metastasis.

## 1. Introduction

Colorectal cancer (CRC) is one of the most common diagnostic cancers and the fourth leading cause of cancer-related deaths worldwide [1]. Due to early detection and lifestyle change, the incidence and mortality of CRC have been steadily declining [2]. Nevertheless, postoperative recurrence and distant metastasis remain the main cause of CRC-related death and poor prognosis [3].

The lung is the second most common distant metastatic site for primary CRC, and about 10–25% CRC patients eventually develop pulmonary metastasis [4]. The survival time of CRC patients with lung metastasis is relatively short, with a median survival duration of less than 10 months in untreated patients [4]. Surgical resection combined with adjuvant therapy, such as chemotherapy, radiotherapy, and immunotherapy, remains the first curative option for CRC [5]. Multidisciplinary standardized comprehensive diagnosis and treatment models have become the general trend in the treatment of malignant tumors. Traditional Chinese medicine (TCM) is an important part of the comprehensive treatment of CRC. The efficacy of TCM in increasing chemotherapy efficiency, reducing toxicity, prolonging the survival duration, and improving the patient's quality of life and immune function has been fully affirmed [6, 7]. Recently, the potential role of TCM in preventing postoperative recurrence and metastasis has also attracted attention [8].

*Yifei Jianpi Tongfu* formula (YJTF), a TCM prescription composed of diverse medicinal herbs, has proven effective for the treatment of various respiratory diseases, such as chronic obstructive pulmonary disease (COPD) and other diseases related to airway inflammation [9–11]. What is noteworthy is that it is also widely used in the treatment of lung cancer. Related studies have demonstrated that it can inhibit the growth, invasion, and migration of lung cancer cells [12–14]. Knowing that “Interior and Exterior Relationship between the Lung and Large Intestine” is one of the most typical viscera correlation theories, intestinal diseases are presumably closely related to lung injury, which just matches with the concept of the gut-lung axis. Based on the theory of “Exterior of the Lung and Large Intestine,” the present study aimed to observe the therapeutic efficacy of YJTF for CRC lung metastasis and explored the related action mechanism of YJTF composed of Radix Astragali (RA, *Astragalus mongholicus* Bunge (Fabaceae)), Radix Codonopsis (RC, *Codonopsis pilosula* (Campanulaceae)), Rhizoma Atractylodis Macrocephalae (Asteraceae) (RAM), *Poria cocos* (Po, *Wolfiporia extensa* (Polyporaceae)), Semen Coicis (SC, *Coix lacryma-jobi* (Poaceae)), Radix Ophiopogonis (RO, *Ophiopogon japonicus* (Asparagaceae)), Akebia Trifoliata Koidz (AK, *Akebia quinata* (Lardizabalaceae)), *Ampelopsis sinica* (Am, *Ampelopsis glandulosa* (Vitaceae)), *Sargentodoxa cuneate* (Sa, *Sargentodoxa cuneata* (Oliv.) Rehd. et wils. (Lardizabalaceae)), Radix Asteris Tatarici (RAT, *Aster tataricus* (Asteraceae)), Radix Platycodonis (RP, *Platycodon grandiflorus* (Campanulaceae)), and Radix Glycyrrhiza (RG, *Glycyrrhiza uralensis* (Fabaceae)). Then, we tried to verify its curative effect and explore the potential mechanism by using the network analysis method in combination with in vivo experiments, attempting to propose new strategies for the adjuvant treatment of CRC lung metastasis.

## 2. Materials and Methods

### 2.1. Data Preparation

#### 2.1.1. Chemical Components of YJTF

YJTF compounds were obtained from the Traditional Chinese Medicine System Pharmacology Database and Analysis Platform 2.3 (TCMSP, http://lsp.nwu.edu.cn/tcmspsearch.php) [15] and the Traditional Chinese Medicine Integrated Database 2.0 (TCMID, http://www.megabionet.org/tcmid/) [16]. The TCMSP database was also used to collect the pharmacokinetic (ADME) information of YJTF compounds. The ADME parameters, oral bioavailability (OB) ≥ 30%, and drug-like (DL) ≥ 0.18 were used as the parameters for screening the chemical components in YJTF. For several compounds that are not included in the TCMSP database but are found in the TCMID, Lipinski's rule (LR) was used for active compound identification according to the following criteria: molecular weight (MW) ≤ 500, chemical composition with not more than 10 hydrogen bond acceptors (Hacc ≤ 10), lesser than five hydrogen bond donors (Hdon ≤ 5), and octanol-water partition coefficient lesser than 5 (LogP ≤ 5) [17]. Compounds that failed to satisfy at least two of the above requirements were excluded.

#### 2.1.2. Prediction of Putative Targets of YJTF

To identify the corresponding targets of the main compounds of YJTF, all chemical structures were prepared and converted into canonical SMILES using PubChem (https://pubchem.ncbi.nlm.nih.gov/) [18], then the compound SMILES was input into the SwissTargetPrediction (http://www.swisstargetprediction.ch/) [19], and the compound predicted targets were output. UniProt (https://www.uniprot.org/) (UniProt, 2021) was used to normalize the naming of the drug targets.

#### 2.1.3. CRC Lung Metastasis-Related Targets

Potential genes associated with CRC lung metastasis were collected from GeneCards (https://www.genecards.org/) and Online Mendelian Inheritance in Man (OMIM) (https://www.omim.org/). GeneCards is a comprehensive database that provides concise genome, proteome, transcriptome, disease, and functional data of all known and predicted human genes [20]. The OMIM database is also an extensive and canonical research resource for information on genes and phenotypes that are secured from the peer-reviewed biomedical literature [21]. UniProt (https://www.uniprot.org/) (UniProt, 2021) was used to normalize the naming of the disease-related targets.

### 2.2. Protein-Protein Interaction (PPI) Data

PPI data in human genomes were extracted from version11.0 of STRING (https://string-db.org/cgi/input.pl), a weighted interaction database with physical and functional interactions that are integrated from multiple data sources. To construct a PPI network with high confidence edges, we filtered the STRING with threshold 0.4 and only interactions with weight above the threshold were selected for the newly constructed PPI network.

The visual network graphs were created by Cytoscape (version 3.7.2) (http://www.cytoscape.org/), an open-source software platform for visualizing complex networks [22]. The target interaction network parameters were calculated by NetworkAnalyzer. Molecular Complex Detection (MCODE) of Cytoscape was used to search the highly connected subnetworks in the PPI network.

### 2.3. GO and KEGG Pathway Enrichment Analysis

To reveal the potential biological functions of YJTF in the treatment of CRC lung metastasis, the Gene Ontology (GO) and Kyoto Encyclopedia of Genes and Genomes (KEGG) pathway enrichment analyses were performed using the Metascape database (http://metascape.org/) [23], where the smaller the *p* value, the more the enrichment would be.

### 2.4. Network Construction

To demonstrate the multicompound therapeutic features of YJTF, network construction was performed as follows: (1) the herb-compound-target network (H-C-T network) was constructed to explore the active compounds and their potential targets, and the core compounds were obtained through the H-C-T network; (2) the PPI network was built to analyze target interactions, from which Hub targets involved in YJTF treatment of CRC lung metastasis were selected; and (3) the target-pathway (T-P) network was constructed to show the functional pathways of YJTF for the treatment of CRC lung metastasis.

All these networks were constructed and analyzed in Cytoscape 3.7.2 software. Then, four topological properties (“degree,” “betweenness,” “closeness,” and “average shortest path length”) were calculated to screen the putative targets for topological importance, knowing that larger the node degree, betweenness, or closeness centrality, the more important the node would be in the network [24].

### 2.5. Molecular Docking Simulation

Molecular docking was conducted to validate whether the compounds in YJTF could bind to the potential targets. The 2D structures of the top five core compounds were downloaded from the TCMSP database [15] and PubChem [18]. The polar hydrogens and partial charge were added to the structures using AutoDock Tools (version 1.5.6). The protein crystal structures corresponding to the core target genes were downloaded from the Protein Data Bank (PDB) database (http://www1.rcsb.org/) [25]. Water molecules and heteromolecules of the proteins were removed by Pymol. Hydrogen atoms and charge operations to the proteins were added in AutoDock Tools. The results were analyzed and interpreted by Pymol.

### 2.6. Cell Culture

HCT-116 cells (American Type Culture Collection, Manassas, VA, US) were cultured in RPMI1640 medium (Invitrogen, Carlsbad, CA, US) supplemented with 10% fetal bovine serum (FBS, Invitrogen) and kept in a 37°C incubator with 5% CO_2_.

The HCT 116 cell line was isolated from the colon of an adult male, colon cancer patient, which is commonly used to study cancer biology including the research of treatment schemes and drug screening. This is a growth factor-independent cell line that has been shown to be invasive and highly motile in in vitro studies. Subcutaneous xenograft experiments have demonstrated it to be highly tumorigenic. Therefore, it is currently widely used in the study of colorectal cancer metastasis [26, 27].

### 2.7. YJTF Preparation

The components of YJTF are as follows: RA 18 g, RC 20 g, RAM 15 g, Po 12 g, SC 15 g, RO 15 g, AK 9 g, Am 30 g, Sa 30 g, RAT 12 g, RP 9 g, and RG 5 g. The herbs were prepared into fluid extracts according to the standard operating procedures by the pharmacy of Yueyang Hospital of Integrated Traditional Chinese and Western Medicine, Shanghai University of Traditional Chinese Medicine (Shanghai, China). All the herbs were extracted with boiling water, and then, the extracts were concentrated to obtain the decoction (2.47 g/mL).

### 2.8. Animal Experiments

All the animal experiments were approved by the Institutional Review Board of Shanghai Pulmonary Hospital, Tongji University School of Medicine (Shanghai, China), and performed in accordance with the national guidelines for the care and use of laboratory animals. The lung metastasis mouse model was established by intravenous injection of HCT116 cells (5 × 10^6^ cells in 200 *μ*l) via the tail vein into male BALB/c nude mice aged 4–5 weeks (SLAC Laboratory, Shanghai, China). Twenty-four mice were equally randomized into four groups: a control (Con) group, an oxaliplatin (Oxa) group, a YJTF (YFT) group, and a YJTF + Oxaliplatin (YJT + Oxa) group. Mice in YJT and YJT + Oxa groups were intragastrically administered with 200 *μ*l YJTF decoction daily for 6 weeks, and mice in Con and Oxa groups were administered with 200 *μ*l PBS. Mice in Oxa and YJT + Oxa groups were intraperitoneally injected with 5 mg/kg oxaliplatin twice a week for 6 weeks, and mice in Con and YJT groups were injected with an equal amount of glucose solution.

The mice were sacrificed 6 weeks after treatment. The number of lung metastatic nodes was measured, and the metastatic tumor tissues were collected for immunohistochemical (IHC) staining with E-cadherin, N-cadherin, and vimentin antibodies. The primary organs including the heart, liver, spleen, kidney, skin, and skeletal muscle were collected for hematoxylin and eosin (H&E) staining. Changes in the body weight of the animals were also recorded during the experiment.

### 2.9. Statistical Analysis

The SPSS software version 18.0 (SPSS Inc., Chicago, IL, USA) was used for statistical analysis. Data are expressed as the means ± standard deviation (SD). For comparisons of the means of multiple groups, one-way ANOVAs followed by LSD tests were performed. *P* < 0.05 was considered statistically significant.

## 3. Results

### 3.1. Construction of the Herb-Compound-Target Network of YJTF

Altogether 935 components in YJTF were obtained from the TCMSP and TCMID databases, 87 of which belonged to RA, 134 to RC, 55 to RAM, 34 to Po, 38 to SC, 55 to RO, 12 to AK, 22 to Am, 25 to Sa, 91 to RAT, 102 to RP, and 280 to RG. According to the screening conditions of OB ≥ 30%, DL ≥ 0.18, and Lipinski's rule, 243 active components from 12 herbs of YJTF were collected, comprising 20 components extracted from RA, 21 from RC, 7 from RAM, 15 from Po, 9 from SC, 33 from RO, 6 from AK, 8 from Am, 5 from Sa, 19 from RAT, 7 from RP, and 93 from RG. After removing the redundancy, 214 components were selected as candidate bioactive components. Then, 872 protein targets were harvested based on the target prediction performed using the SwissTargetPrediction website tool. After eliminating the overlapping targets, 131 targets remained.

**Figure 1:**
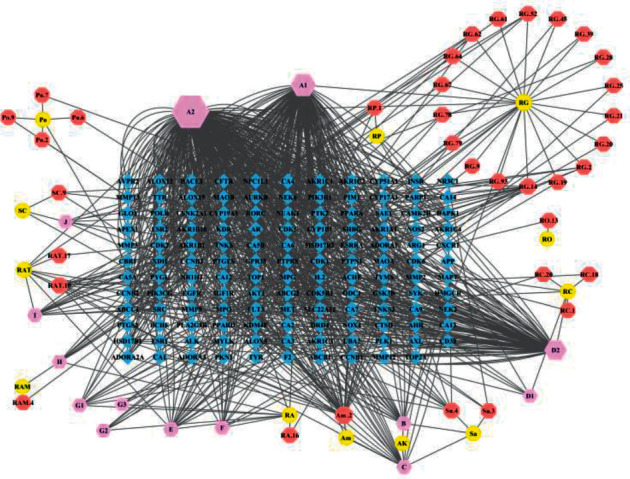
Herb-compound-target network (H-C-T network) of YJTF. Yellow ellipses represent the herbs present in YJTF; red octagons represent active compounds in each herb; pink hexagons represent active compounds shared by two or more herbs; and blue diamonds correspond to related targets (the IDs of the components are described in Supplementary Table 1).

The H-C-T network of YJTF was visualized in Cytoscape (Figure 1), which contained 191 nodes and 946 edges. It was found that quercetin had the highest degree of connectivity (364) in the network, followed by kaempferol (172), luteolin (123), apigenin (38), and isorhamnetin (21). The properties of the H-C-T network were suitable for displaying complex ingredients, multiple targets, and close interactions between ingredients and targets. Detailed information about the active compounds and targets identified in YJTF is shown in Supplementary Table 1.

### 3.2. Screening Results of Target Proteins for CRC Lung Metastasis

The targets for CRC lung metastasis were integrated from multisource databases including GeneCards and OMIM, and finally, a list of 2,047 disease-related targets was obtained after eliminating the duplicates (Supplementary Table 2). Eighty-one overlapping targets were identified as the key targets for studying the therapeutic effect of YJTF on CRC lung metastasis (Supplementary Table 3).

### 3.3. Overlapping Targets' PPI Network Analysis

**Figure 2:**
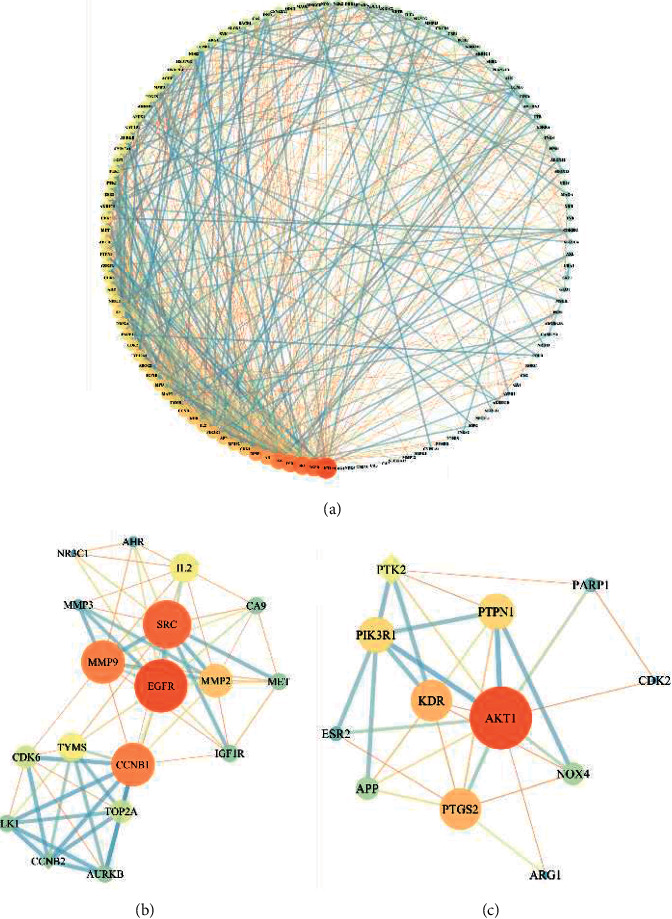
PPI networks of YJTF for CRC lung metastasis treatment. Each node represents a protein target and each line represents the interaction between two nodes. Nodes in red are important and nodes in green are less important in the network. (a) The PPI network diagram arranged according to the degree value. Two clusters are detected in the YJTF-CRC lung metastasis PPI network. (b, c) Clusters 1 and 2. The diamonds are seed nodes of each cluster.

The 81 overlapping targets were input into the STRING database to acquire PPI network. The visualized PPI network was constructed by Cytoscape 3.7.2. The 122 nodes represented proteins, and the 729 edges represented the interactions between the proteins (Figure 2(a), Supplementary Table 4). By degree order, the top five were AKT1 (60), EGFR (48), SRC (47), ESR1 (43), and PTGS2 (42).

Similar function clusters of the PPI network were selected by MCODE analysis based on the topology to find densely interconnected regions using Cytoscape. Two clusters of functional modules were detected (K-core = 5) (Figure 2). The clusters in a PPI network often represent protein complexes and parts of pathways, while the clusters in a protein similarity network usually represent protein families. It was found that Cluster 1 comprised 18 nodes and 68 edges with a score of 8.000 (Figure 2(b)). The seed node of this cluster was CCNB1, which was involved in cell proliferation, growth, apoptosis, and migration in many gastrointestinal cancers [28–30]. Cluster 2 comprised 12 nodes and 30 edges with a score of 5.455 (Figure 2(c)). The seed node of this cluster was PTK2, which is a nonreceptor protein-tyrosine kinase playing an essential role in regulating cell migration, adhesion, spreading, reorganization of the actin cytoskeleton, formation and disassembly of focal adhesions and cell protrusions, cell cycle progression, cell proliferation, and apoptosis [31, 32]. Further research demonstrated that PTK2 played a critical role in CRC growth and metastasis and may serve as a potential therapeutic target for CRC metastasis and may also be a promising biomarker for early diagnosis of metastasis [33, 34]. Thus, the PPI network and the further clustering indicate that YJTF may inhibit CRC lung metastasis by regulating various biological processes.

### 3.4. GO Enrichment Analysis

**Figure 3:**
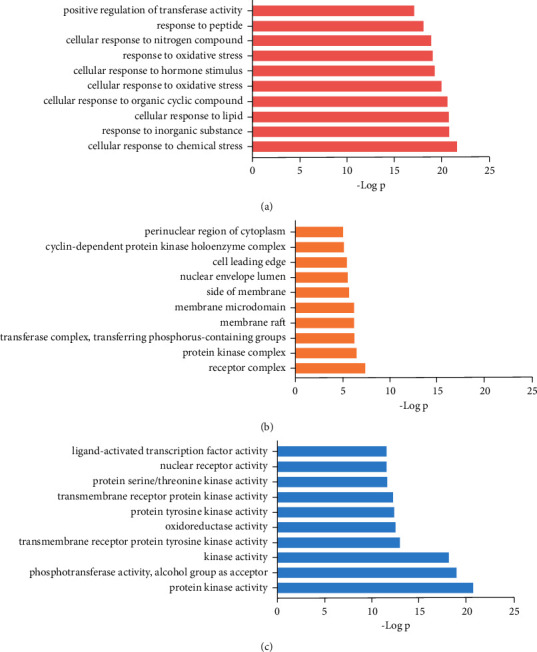
GO enrichment analysis of the 81 putative targets. The top 10 significantly enriched terms in BP (biological process) (a), CC (cellular component) (b), and MF (molecular function) (c) categories.

To further excavate the significance of the 81 hub targets, gene ontology enrichment analysis was used, which consisted of three parts: BP (biological process), CC (cellular component), and MF (molecular function). As shown in the results of the enrichment, a total of 1,344 GO enrichment results were obtained, including BP (1,155 terms), MF (126 terms), and CC (63 terms). We set the level of statistical significance at *PP* values<0.01. The top 10 significantly enriched terms were selected in the BP, MF, and CC categories, which are listed in Figure 3. Detailed information about the results of GO enrichment analysis is shown in Supplementary Table 5.

### 3.5. KEGG Pathway Enrichment Analysis

**Figure 4:**
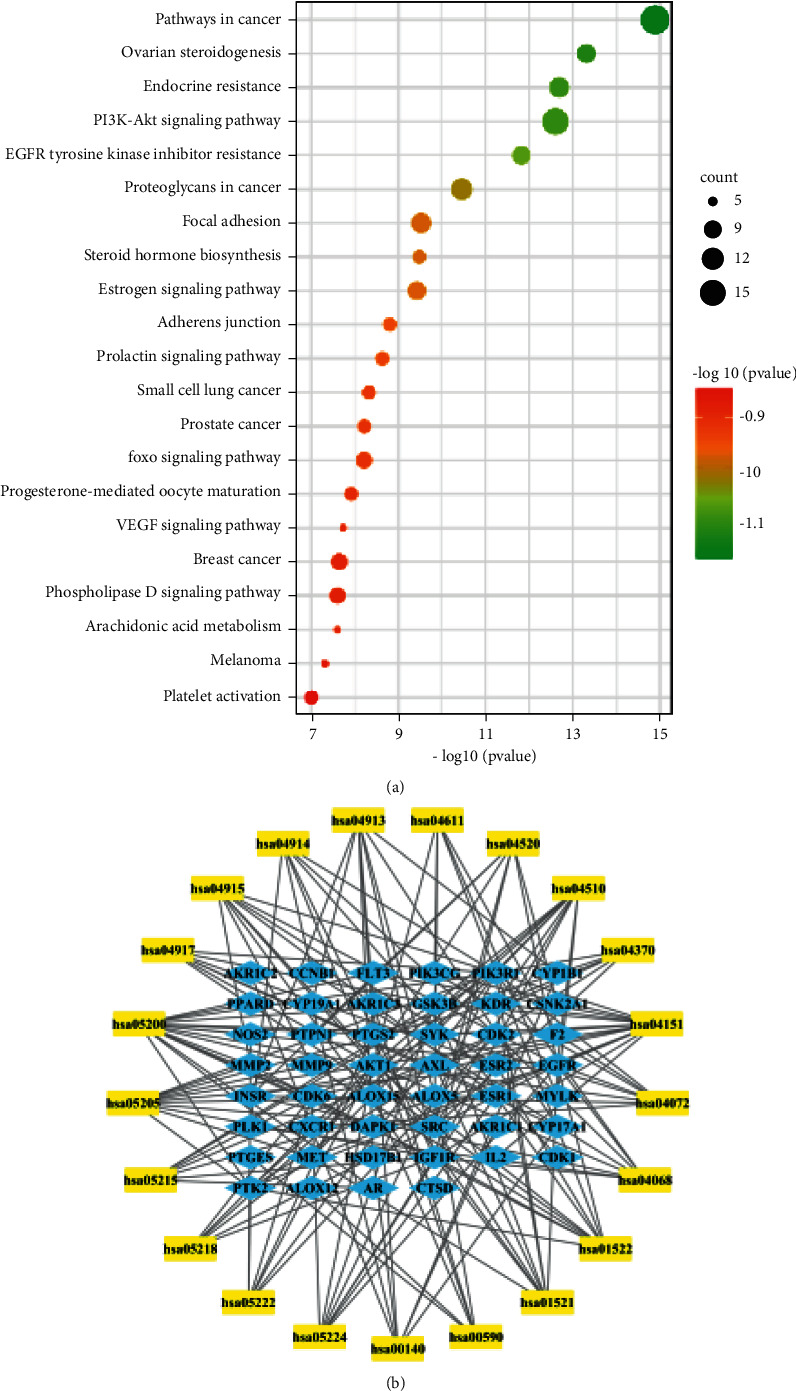
Results of the pathway analysis of the top 21 pathways: bubble diagram of the pathway (a) and T-P network diagram (b).

The 81 putative targets of active compounds were mapped onto 102 KEGG pathways (*P* < 0.01). The top 21 pathways at a significance threshold of LogP < -7 are displayed in Table 1 and Figure 4(a), and detailed information about the results of KEGG pathway enrichment analysis is shown in Supplementary Table 5. As shown in the table, many of the top 21 pathways were related to cancer, such as pathways in cancer (hsa05200), PI3K-Akt pathway (hsa04151), and proteoglycans in cancer (hsa05205). In particular, focal adhesion (hsa04510, top 7) and adherens junction (hsa04520, top 10) were highly related to cell adhesion probably through participation in invasion and migration of cancer, suggesting that the putative targets of YJTF were highly related to cancer metastasis.

Based on the target identification and pathway analysis, a T-P network was built with 67 nodes and 181 edges (Figure 4(b)). The results revealed that the active compounds of the YJTF had effects on CRC lung metastasis through regulating multiple pathways, especially by regulating pathways related to cell adhesion.

### 3.6. Molecular Docking Analysis

Then, we conducted molecular docking to the high-degree compounds and proteins to verify the binding energy. According to the H-C-T network, we found that quercetin, kaempferol, luteolin, apigenin, and isorhamnetin were the core compounds of YJTF. The hub protein targets were identified by the top five node degrees of the PPI network, including AKT1, EGFR, SRC, ESR1, and PTGS2.

**Figure 5:**
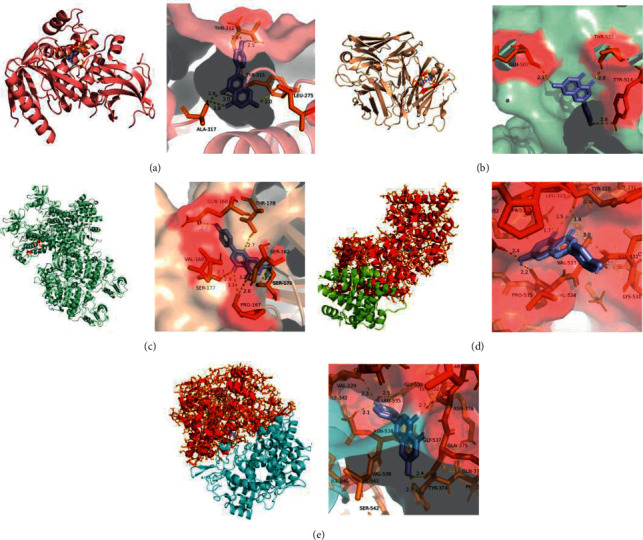
Molecular docking diagrams of AKT1 (a), EGFR (b), SRC (c), ESR1 (d), and PTGS2 (e) complexed with apigenin.

The results obtained by the AutoDock Tools are shown in Table 2. It is generally believed that ligand-receptor pairs with lower-energy-binding conformational stability have a higher possibility of interaction. As seen in Table 2, the binding energy for the core compounds and proteins was all less than -5 kcal/mol, indicating that the compounds had a certain affinity for the protein crystal structure [35]. Apigenin showed the lowest binding energy in the top five proteins, as −9.39 kcal/mol in AKT1, −7.96 kcal/mol in EGFR, −6.61 kcal/mol in SRC, −7.65 kcal/mol in ESR1, and −7.36 kcal/mol in PTGS2. The results of the molecular docking between apigenin and the five high-degree targets are presented in Figure 5.

### 3.7. Drug Safety Verification

**Figure 6:**
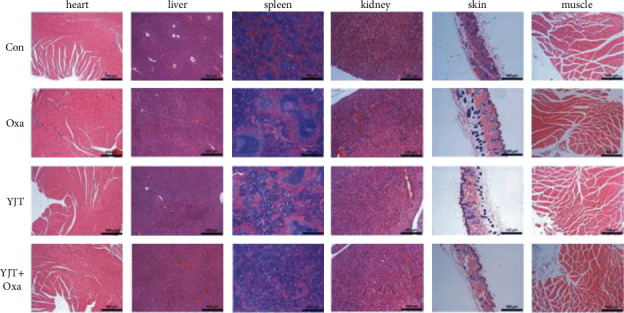
H&E analysis in each group indicated that there were no obvious abnormal changes (not shown) in the HE slices.

Paraffin-embedded tissues (including the heart, liver, spleen, kidney, skin, and skeletal muscle) were sectioned and stained with H&E for histological evaluation to validate the drug safety. After staining, the nucleus presented as blue and the cytoplasm as red by microscopy, and the tissue morphology can be clearly observed. H&E analysis of the major organs and tissues in each group indicated that there were no obvious abnormal changes (not shown) in the HE slices. It can be considered that the drug treatment (Oxa, YJT, and YJT + Oxa) is safe and have no obvious toxic side effects (Figure 6).

### 3.8. Quality of Life Assessment

**Figure 7:**
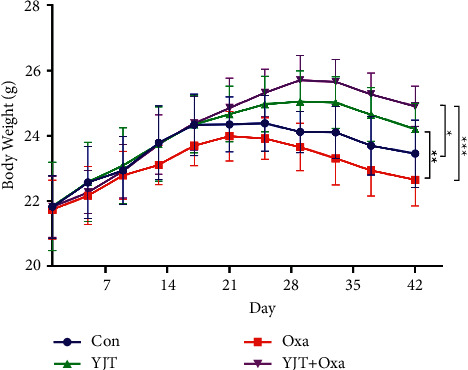
Changes in body weight of mice. The body weight of mice treated with YJTF was relatively stable. (^*∗*^*P* < 0.05, ^*∗∗*^*P* < 0.01, ^*∗∗∗*^*P* < 0.001).

During the experiment, the body weight of the mice was measured at 4-day intervals (Figure 7). It was found that the body weight in Con group increased first and then decreased, which may be due to tumor growth. Significant weight loss was observed in the mice treated with Oxa. Body weight of the mice treated with YJTF was relatively stable, especially in the YJT + Oxa group, and the weight was the highest at the end of the experiment, indicating that YJTF treatment could improve the quality of life of the mice significantly.

### 3.9. Numbers of Lung Metastasis

**Figure 8:**
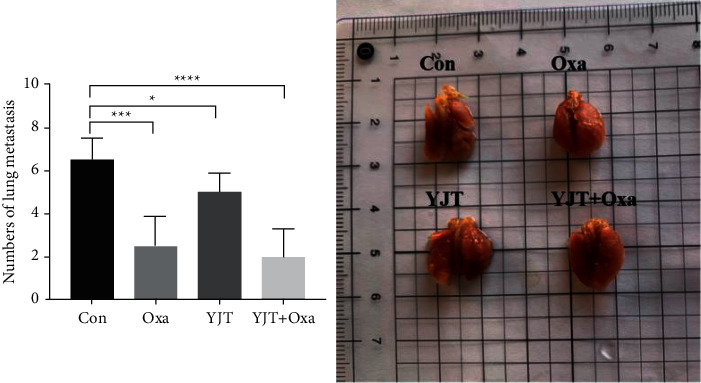
Number of lung metastatic nodes in BALB/c nude mice injected with HCT116 cells in each group. Data are presented as the mean ± SEM (^∗^*P* < 0.05, ^*∗∗∗*^*P* < 0.001, and ^*∗∗∗∗*^*P* < 0.0001).

After 6-week treatment, all mice were sacrificed and their lungs were collected and fixed in 4% PFA. Then, visible lung metastases were counted in each group of mice. The representative lung of each group and the quantitative mean number of visible lung metastases are shown in Figure 8. It was found that the number of metastases in Oxa and YJF + Oxa groups was significantly reduced as compared with that in the Con group (*P* < 0.01), and the number of lung metastases in the YJT group was slightly reduced (*P* < 0.05).

### 3.10. IHC Staining Assessment

**Figure 9:**
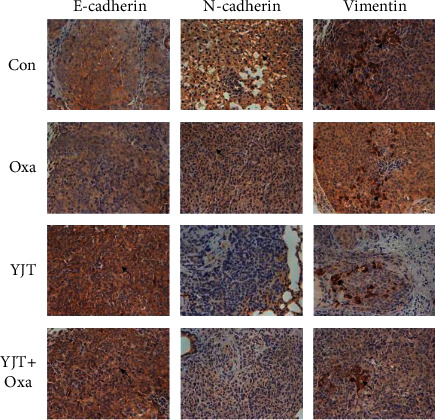
Immunofluorescence staining to evaluate the levels of E-cadherin, N-cadherin, and vimentin. All experiments were performed in triplicate.

We performed IHC staining to evaluate the expression of E-cadherin, N-cadherin, and vimentin in the metastatic tumor tissues. Brown staining indicates positive expression areas, the shade of the color represents the expression level of the target protein, and the cell nuclei were stained in blue color by hematoxylin. As the picture shows, we found that the expression of E-cadherin was significantly upregulated in YJT and YJT + Oxa groups, while it was significantly downregulated in YJT and YJT + Oxa groups (Figure 9), suggesting that YJTF may inhibit CRC metastasis by modulating epithelial-mesenchymal transition (EMT).

## 4. Discussion

Distant metastasis is the main cause of cancer-related death and remains one of the focuses of cancer research. Metastasis is known as a multistep complex process involving multiple factors. EMT is an embryonic program that loosens cell-cell adherence complex and endows cells with enhanced progression. Cancer cells undergoing EMT are more aggressive and often display increased invasiveness, stem-like features, and resistance to apoptosis [36, 37]. EMT-expressing cancer cells showed decreased expression of epithelial cell markers such as E-cadherin and EpCAM, while expression of mesenchymal cell markers such as vimentin and N-cadherin increased. Expression of the proteins' characteristic of mesenchymal cells (N-cadherin and vimentin) and loss of epithelial markers (E-cadherin) correlate with tumor progression and poor prognosis [38]. Increasing evidence suggests that inhibiting EMT can effectively inhibit tumor metastasis, recurrence, and drug resistance [39]. Due to the role of EMT in cancer progression, this process has attracted more attention from many researchers in the field of cancer treatment [40].

In this study, we applied YJTF in the treatment of CRC lung metastasis and verified its therapeutic efficacy through in vivo experiments. To provide further insights into the underlying mechanisms, we used the systematic pharmacology method and molecular docking to explore the potential molecular mechanisms of the 243 bioactive compounds and 81 putative targets of YJTF obtained from the TCMSP and TCMID by overlapping the targets between YJTF-associated targets and the targets of CRC lung metastasis. According to the H-C-T network topological analysis, we found that the core compounds included quercetin, kaempferol, luteolin, apigenin, and isorhamnetin. Previous studies have demonstrated that all these compounds have therapeutic effects on various cancers. As the compound with the highest degree, quercetin is ubiquitously present in fruits and vegetables, being one of the most common dietary flavanols. The anticancer effects of quercetin include its ability to promote the loss of cell viability, apoptosis, and autophagy through the modulation of PI3K-Akt-mTOR, Wnt/*β*-catenin, and MAPK/ERK1/2 pathways [41]. Quercetin also prevents metastasis by reducing VEGF secretion and MMP levels [42]. Both kaempferol and luteolin have antitumor effects and have been linked to a decrease in the risk of developing some types of cancers, including colon, liver, lung, and breast cancers. In particular, they all have the effect to reverse EMT, a process believed to be closely related to metastasis [43, 44]. In addition, apigenin was found to inhibit cell invasion, migration, and metastasis through NEDD9-Src-Akt pathway in CRC in the previous study [45]. Furthermore, all these compounds were also reported to have effects on cancer-related inflammation. These previous studies suggest that YJTF may inhibit CRC lung metastasis through the compounds of quercetin, kaempferol, luteolin, apigenin, and isorhamnetin.

Furthermore, according to PPI analysis, we determined the interactions between the targets and their importance. AKT1, EGFR, SRC, ESR1, and PTGS2 are the top five targets in the PPI network, and they were reported as critical regulatory factors in cancer metastasis and EMT [46–49]. Through cluster analysis of putative targets, we screened two clusters of functional modules, whose seed nodes were CCNB1 and PTK2, respectively, and both proved to play an important role in metastasis of CRC [28–32]. In addition, the results of our KEGG pathway enrichment analysis showed that multiple pathways were related to cancer, such as pathways in cancer, PI3K-Akt signaling pathway, proteoglycans in cancer, and many other cancer-related pathways. Significantly, our results revealed multiple pathways involved in EMT including the PI3K-Akt signaling pathway, focal adhesion, and adherens junction, all of which have proved to participate in tumor invasion and migration [50–52], suggesting that YJTF may inhibit CRC lung metastasis by modulating EMT. The results of the subsequent in vivo experiments confirmed our hypothesis. As inflammation plays an important role in the occurrence and progression of cancer, inflammation is also a potent inducer of EMT in cancer. These two phenomena may sustain each other in an alliance for metastasis [37]. Moreover, our data showed that GO was enriched mainly in response to oxidative stress and regulation of inflammatory response and multiple inflammation-related pathways, suggesting that YJTF may affect CRC lung metastasis through its regulatory effect on the inflammatory response.

Although the incidence and mortality rate of CRC have decreased due to the advent of effective cancer screening measures, there has been an increase in the number of young patients diagnosed with CRC. Therefore, how to improve the quality of life of cancer patients has also become one of the focuses of comprehensive tumor treatment. In recent decades, several forms of complementary and alternative medicines have been utilized to defeat cancer worldwide. Traditional Chinese herbal medicines have been widely accepted as a mainstream form of complementary and alternative therapy with beneficial effects for cancer patients in China [53, 54]. Previous studies have demonstrated that some Chinese herbal medicines can suppress CRC cell proliferation, invasion, and migration, inhibit EMT, induce cell apoptosis, and block angiogenesis in the treatment of CRC [55]. Furthermore, they have advantages in reducing unwanted effects from other therapies and improving the immune system, thus prolonging cancer patient survival and improving their quality of life [56]. In addition, Chinese herbal medicines can alleviate cancer pain-induced hypersensitivity and relieve depressive symptoms in cancer patients [57, 58]. There are also studies indicating that Chinese herbal medicines can significantly improve fatigue in cancer patients [59, 60]. It was found in our study that body weight loss caused by oxaliplatin was attenuated after YJTF treatment, suggesting that YJTF may also have an effect on improving the quality of life of patients with CRC lung metastasis.

## 5. Conclusion

In this study, we used the network analysis approach to investigate the active compounds and potential targets of YJTF, and explored their underlying molecular mechanisms in treating CRC lung metastasis. The results demonstrated that YJTF participated in various biological processes related to cancer, probably by regulating pathways related to cell adhesion, thus inhibiting CRC invasion and migration. Our subsequent molecular docking showed that each core compound of YJTF had a favorable binding ability with AKT1, EGFR, SRC, ESR1, and PTGS2. More importantly, our in vivo experiments validated the therapeutic effect of YJTF on CRC lung metastasis by regulating EMT. All these findings may provide a new thought and reference for the comprehensive treatment of CRC lung metastasis.

## Figures and Tables

**Table 1:** Information on enrichment analysis based on Metascape.

Term ID	Pathway	Counts	Log*P*	Genes
hsa05200	Pathways in cancer	17	−14.8972	AKT1, AR, CDK2, CDK6, DAPK1, EGFR, FLT3, GSK3B, IGF1R, MET, MMP2, MMP9, NOS2, PIK3R1, PPARD, PTGS2, PTK2
hsa04913	Ovarian steroidogenesis	9	−13.3126	ALOX5, CYP1B1, CYP17A1, CYP19A1, HSD17B1, IGF1R, INSR, PTGS2, AKR1C3
hsa01522	Endocrine resistance	10	−12.6956	AKT1, EGFR, ESR1, ESR2, IGF1R, MMP2, MMP9, PIK3R1, PTK2, SRC
hsa04151	PI3K-Akt signaling pathway	15	−12.6073	AKT1, CDK2, CDK6, EGFR, FLT3, GSK3B, IGF1R, IL2, INSR, KDR, MET, PIK3CG, PIK3R1, PTK2, SYK
hsa01521	EGFR tyrosine kinase inhibitor resistance	9	−11.8265	AKT1, AXL, EGFR, GSK3B, IGF1R, KDR, MET, PIK3R1, SRC
hsa05205	Proteoglycans in cancer	11	−10.4553	AKT1, EGFR, ESR1, IGF1R, KDR, MET, MMP2, MMP9, PIK3R1, PTK2, SRC
hsa04510	Focal adhesion	10	−9.52178	AKT1, EGFR, GSK3B, IGF1R, KDR, MET, MYLK, PIK3R1, PTK2, SRC
hsa00140	Steroid hormone biosynthesis	7	−9.47634	CYP1B1, CYP17A1, CYP19A1, AKR1C1, AKR1C2, HSD17B1, AKR1C3
hsa04915	Estrogen signaling pathway	9	−9.42356	AKT1, CTSD, EGFR, ESR1, ESR2, MMP2, MMP9, PIK3R1, SRC
hsa04520	Adherens junction	7	−8.8002	CSNK2A1, EGFR, IGF1R, INSR, MET, PTPN1, SRC
hsa04917	Prolactin signaling pathway	7	−8.63274	AKT1, CYP17A1, ESR1, ESR2, GSK3B, PIK3R1, SRC
hsa05222	Small cell lung cancer	7	−8.32439	AKT1, CDK2, CDK6, NOS2, PIK3R1, PTGS2, PTK2
hsa05215	Prostate cancer	7	−8.21676	AKT1, AR, CDK2, EGFR, GSK3B, IGF1R, PIK3R1
hsa04068	Foxo signaling pathway	8	−8.21522	AKT1, CCNB1, CDK2, EGFR, IGF1R, INSR, PIK3R1, PLK1
hsa04914	Progesterone-mediated oocyte maturation	7	−7.91617	AKT1, CCNB1, CDK1, CDK2, IGF1R, PIK3R1, PLK1
hsa04370	VEGF signaling pathway	6	−7.72779	AKT1, KDR, PIK3R1, PTGS2, PTK2, SRC
hsa05224	Breast cancer	8	−7.64375	AKT1, CDK6, EGFR, ESR1, ESR2, GSK3B, IGF1R, PIK3R1
hsa04072	Phospholipase D signaling pathway	8	−7.60218	AKT1, EGFR, F2, CXCR1, INSR, PIK3CG, PIK3R1, SYK
hsa00590	Arachidonic acid metabolism	6	−7.59584	ALOX12, ALOX5, ALOX15, PTGS2, AKR1C3, PTGES
hsa05218	Melanoma	6	−7.31274	AKT1, CDK6, EGFR, IGF1R, MET, PIK3R1
hsa04611	Platelet activation	7	−7.00258	AKT1, F2, MYLK, PIK3CG, PIK3R1, SRC, SYK

**Table 2:** The binding energy values of core compounds of YJTF and core targets.

Target	Compounds	Binding affinity/(kcal/mol)
AKT1 (6HHF)	Luteolin	−6.73
	Quercetin	−6.86
	Kaempferol	−6.84
	Isorhamnetin	−6.91
	Apigenin	−9.39

EGFR (3GKW)	Luteolin	−6.54
	Quercetin	−7.13
	Kaempferol	−6.39
	Isorhamnetin	−7.24
	Apigenin	−7.96

SRC (6E6E)	Luteolin	−5.59
	Quercetin	−5.05
	Kaempferol	−5.30
	Isorhamnetin	−5.18
	Apigenin	−6.61

ESR1 (1R5K)	Luteolin	−5.73
	Quercetin	−5.19
	Kaempferol	−5.51
	Isorhamnetin	−6.97
	Apigenin	−7.65

PTGS2 (5IKQ)	Luteolin	−6.90
	Quercetin	−6.79
	Kaempferol	−6.45
	Isorhamnetin	−7.05
	Apigenin	−7.36

## Data Availability

The data used to support the findings of this study are included within the supplementary information file (s).

## Supplementary Materials

Supplementary Materials Detailed information about the active compounds and targets identified in YJTF is shown in Supplementary Table 1. All of the disease-related targets for CRC lung metastasis are listed in Supplementary Table 2. Detailed information about the 81 overlapping targets identified as the key targets for studying the therapeutic effect of YJTF on CRC lung metastasis is shown in Supplementary Table 3. Detailed information about the PPI network is shown in Supplementary Table 4. Detailed information about the GO and KEGG enrichment analysis of the putative targets is shown in Supplementary Table 5.
