# Supplementary material for: Study on the Action Mechanism of the Yifei Jianpi Tongfu Formula in Treatment of Colorectal Cancer Lung Metastasis Based on Network Analysis, Molecular Docking, and Experimental Validation
Source: Evid Based Complement Alternat Med. 2022 Jul 30;2022:6229444. doi: 10.1155/2022/6229444 (PMC9356795; doi:10.1155/2022/6229444)
Supplement: Supplementary Materials — Detailed information about the active compounds and targets identified in YJTF is shown in Supplementary Table 1. All of the disease-related targets for CRC lung metastasis are listed in Supplementary Table 2. Detailed information about the 81 overlapping targets identified as the key targets for studying the therapeutic effect of YJTF on CRC lung metastasis is shown in Supplementary Table 3. Detailed information about the PPI network is shown in Supplementary Table 4. Detailed information about the GO and KEGG enrichment analysis of the putative targets is shown in Supplementary Table 5. [file 6229444.f1.zip › Supplementary Table.1-revised 2.0.docx]

| ID | Herb | Ingredient_name | Target_name |
| --- | --- | --- | --- |
| A1(RA.15、RAT.15、RG.13、Am.5) | RA、RAT、RG、Am | kaempferol | NOX4 |
| A1(RA.15、RAT.15、RG.13、Am.5) | RA、RAT、RG、Am | kaempferol | AKR1B1 |
| A1(RA.15、RAT.15、RG.13、Am.5) | RA、RAT、RG、Am | kaempferol | XDH |
| A1(RA.15、RAT.15、RG.13、Am.5) | RA、RAT、RG、Am | kaempferol | TYR |
| A1(RA.15、RAT.15、RG.13、Am.5) | RA、RAT、RG、Am | kaempferol | FLT3 |
| A1(RA.15、RAT.15、RG.13、Am.5) | RA、RAT、RG、Am | kaempferol | CA2 |
| A1(RA.15、RAT.15、RG.13、Am.5) | RA、RAT、RG、Am | kaempferol | ALOX5 |
| A1(RA.15、RAT.15、RG.13、Am.5) | RA、RAT、RG、Am | kaempferol | CA7 |
| A1(RA.15、RAT.15、RG.13、Am.5) | RA、RAT、RG、Am | kaempferol | HSD17B2 |
| A1(RA.15、RAT.15、RG.13、Am.5) | RA、RAT、RG、Am | kaempferol | ABCC1 |
| A1(RA.15、RAT.15、RG.13、Am.5) | RA、RAT、RG、Am | kaempferol | HSD17B1 |
| A1(RA.15、RAT.15、RG.13、Am.5) | RA、RAT、RG、Am | kaempferol | AHR |
| A1(RA.15、RAT.15、RG.13、Am.5) | RA、RAT、RG、Am | kaempferol | CA12 |
| A1(RA.15、RAT.15、RG.13、Am.5) | RA、RAT、RG、Am | kaempferol | ESRRA |
| A1(RA.15、RAT.15、RG.13、Am.5) | RA、RAT、RG、Am | kaempferol | ABCB1 |
| A1(RA.15、RAT.15、RG.13、Am.5) | RA、RAT、RG、Am | kaempferol | CYP1B1 |
| A1(RA.15、RAT.15、RG.13、Am.5) | RA、RAT、RG、Am | kaempferol | ABCG2 |
| A1(RA.15、RAT.15、RG.13、Am.5) | RA、RAT、RG、Am | kaempferol | ADORA1 |
| A1(RA.15、RAT.15、RG.13、Am.5) | RA、RAT、RG、Am | kaempferol | CA4 |
| A1(RA.15、RAT.15、RG.13、Am.5) | RA、RAT、RG、Am | kaempferol | ACHE |
| A1(RA.15、RAT.15、RG.13、Am.5) | RA、RAT、RG、Am | kaempferol | MAOA |
| A1(RA.15、RAT.15、RG.13、Am.5) | RA、RAT、RG、Am | kaempferol | GLO1 |
| A1(RA.15、RAT.15、RG.13、Am.5) | RA、RAT、RG、Am | kaempferol | SYK |
| A1(RA.15、RAT.15、RG.13、Am.5) | RA、RAT、RG、Am | kaempferol | GSK3B |
| A1(RA.15、RAT.15、RG.13、Am.5) | RA、RAT、RG、Am | kaempferol | MMP9 |
| A1(RA.15、RAT.15、RG.13、Am.5) | RA、RAT、RG、Am | kaempferol | MMP2 |
| A1(RA.15、RAT.15、RG.13、Am.5) | RA、RAT、RG、Am | kaempferol | ALOX15 |
| A1(RA.15、RAT.15、RG.13、Am.5) | RA、RAT、RG、Am | kaempferol | ALOX12 |
| A1(RA.15、RAT.15、RG.13、Am.5) | RA、RAT、RG、Am | kaempferol | PTPRS |
| A1(RA.15、RAT.15、RG.13、Am.5) | RA、RAT、RG、Am | kaempferol | ADORA2A |
| A1(RA.15、RAT.15、RG.13、Am.5) | RA、RAT、RG、Am | kaempferol | CDK5 |
| A1(RA.15、RAT.15、RG.13、Am.5) | RA、RAT、RG、Am | kaempferol | CDK5R1 |
| A1(RA.15、RAT.15、RG.13、Am.5) | RA、RAT、RG、Am | kaempferol | CCNB2 |
| A1(RA.15、RAT.15、RG.13、Am.5) | RA、RAT、RG、Am | kaempferol | CCNB1 |
| A1(RA.15、RAT.15、RG.13、Am.5) | RA、RAT、RG、Am | kaempferol | CDK1 |
| A1(RA.15、RAT.15、RG.13、Am.5) | RA、RAT、RG、Am | kaempferol | CCNB3 |
| A1(RA.15、RAT.15、RG.13、Am.5) | RA、RAT、RG、Am | kaempferol | ARG1 |
| A1(RA.15、RAT.15、RG.13、Am.5) | RA、RAT、RG、Am | kaempferol | GPR35 |
| A1(RA.15、RAT.15、RG.13、Am.5) | RA、RAT、RG、Am | kaempferol | ESR2 |
| A1(RA.15、RAT.15、RG.13、Am.5) | RA、RAT、RG、Am | kaempferol | DAPK1 |
| A1(RA.15、RAT.15、RG.13、Am.5) | RA、RAT、RG、Am | kaempferol | MPG |
| A1(RA.15、RAT.15、RG.13、Am.5) | RA、RAT、RG、Am | kaempferol | SLC22A12 |
| A2(RA.20、RAT.19、RG.92、Am.6) | RA、RAT、RG、Am | quercetin | NOX4 |
| A2(RA.20、RAT.19、RG.92、Am.6) | RA、RAT、RG、Am | quercetin | AVPR2 |
| A2(RA.20、RAT.19、RG.92、Am.6) | RA、RAT、RG、Am | quercetin | AKR1B1 |
| A2(RA.20、RAT.19、RG.92、Am.6) | RA、RAT、RG、Am | quercetin | XDH |
| A2(RA.20、RAT.19、RG.92、Am.6) | RA、RAT、RG、Am | quercetin | MAOA |
| A2(RA.20、RAT.19、RG.92、Am.6) | RA、RAT、RG、Am | quercetin | IGF1R |
| A2(RA.20、RAT.19、RG.92、Am.6) | RA、RAT、RG、Am | quercetin | FLT3 |
| A2(RA.20、RAT.19、RG.92、Am.6) | RA、RAT、RG、Am | quercetin | CYP19A1 |
| A2(RA.20、RAT.19、RG.92、Am.6) | RA、RAT、RG、Am | quercetin | EGFR |
| A2(RA.20、RAT.19、RG.92、Am.6) | RA、RAT、RG、Am | quercetin | F2 |
| A2(RA.20、RAT.19、RG.92、Am.6) | RA、RAT、RG、Am | quercetin | CA2 |
| A2(RA.20、RAT.19、RG.92、Am.6) | RA、RAT、RG、Am | quercetin | PIM1 |
| A2(RA.20、RAT.19、RG.92、Am.6) | RA、RAT、RG、Am | quercetin | ALOX5 |
| A2(RA.20、RAT.19、RG.92、Am.6) | RA、RAT、RG、Am | quercetin | AURKB |
| A2(RA.20、RAT.19、RG.92、Am.6) | RA、RAT、RG、Am | quercetin | DRD4 |
| A2(RA.20、RAT.19、RG.92、Am.6) | RA、RAT、RG、Am | quercetin | ADORA1 |
| A2(RA.20、RAT.19、RG.92、Am.6) | RA、RAT、RG、Am | quercetin | CA7 |
| A2(RA.20、RAT.19、RG.92、Am.6) | RA、RAT、RG、Am | quercetin | GLO1 |
| A2(RA.20、RAT.19、RG.92、Am.6) | RA、RAT、RG、Am | quercetin | MPO |
| A2(RA.20、RAT.19、RG.92、Am.6) | RA、RAT、RG、Am | quercetin | PIK3R1 |
| A2(RA.20、RAT.19、RG.92、Am.6) | RA、RAT、RG、Am | quercetin | ADORA2A |
| A2(RA.20、RAT.19、RG.92、Am.6) | RA、RAT、RG、Am | quercetin | DAPK1 |
| A2(RA.20、RAT.19、RG.92、Am.6) | RA、RAT、RG、Am | quercetin | PYGL |
| A2(RA.20、RAT.19、RG.92、Am.6) | RA、RAT、RG、Am | quercetin | CA1 |
| A2(RA.20、RAT.19、RG.92、Am.6) | RA、RAT、RG、Am | quercetin | GSK3B |
| A2(RA.20、RAT.19、RG.92、Am.6) | RA、RAT、RG、Am | quercetin | SRC |
| A2(RA.20、RAT.19、RG.92、Am.6) | RA、RAT、RG、Am | quercetin | PTK2 |
| A2(RA.20、RAT.19、RG.92、Am.6) | RA、RAT、RG、Am | quercetin | HSD17B2 |
| A2(RA.20、RAT.19、RG.92、Am.6) | RA、RAT、RG、Am | quercetin | KDR |
| A2(RA.20、RAT.19、RG.92、Am.6) | RA、RAT、RG、Am | quercetin | MMP13 |
| A2(RA.20、RAT.19、RG.92、Am.6) | RA、RAT、RG、Am | quercetin | MMP3 |
| A2(RA.20、RAT.19、RG.92、Am.6) | RA、RAT、RG、Am | quercetin | CA3 |
| A2(RA.20、RAT.19、RG.92、Am.6) | RA、RAT、RG、Am | quercetin | ALOX15 |
| A2(RA.20、RAT.19、RG.92、Am.6) | RA、RAT、RG、Am | quercetin | ABCC1 |
| A2(RA.20、RAT.19、RG.92、Am.6) | RA、RAT、RG、Am | quercetin | PLK1 |
| A2(RA.20、RAT.19、RG.92、Am.6) | RA、RAT、RG、Am | quercetin | CA6 |
| A2(RA.20、RAT.19、RG.92、Am.6) | RA、RAT、RG、Am | quercetin | CDK1 |
| A2(RA.20、RAT.19、RG.92、Am.6) | RA、RAT、RG、Am | quercetin | MMP9 |
| A2(RA.20、RAT.19、RG.92、Am.6) | RA、RAT、RG、Am | quercetin | CA12 |
| A2(RA.20、RAT.19、RG.92、Am.6) | RA、RAT、RG、Am | quercetin | MMP2 |
| A2(RA.20、RAT.19、RG.92、Am.6) | RA、RAT、RG、Am | quercetin | PKN1 |
| A2(RA.20、RAT.19、RG.92、Am.6) | RA、RAT、RG、Am | quercetin | CA14 |
| A2(RA.20、RAT.19、RG.92、Am.6) | RA、RAT、RG、Am | quercetin | CA9 |
| A2(RA.20、RAT.19、RG.92、Am.6) | RA、RAT、RG、Am | quercetin | CSNK2A1 |
| A2(RA.20、RAT.19、RG.92、Am.6) | RA、RAT、RG、Am | quercetin | ALOX12 |
| A2(RA.20、RAT.19、RG.92、Am.6) | RA、RAT、RG、Am | quercetin | MET |
| A2(RA.20、RAT.19、RG.92、Am.6) | RA、RAT、RG、Am | quercetin | CA4 |
| A2(RA.20、RAT.19、RG.92、Am.6) | RA、RAT、RG、Am | quercetin | NEK2 |
| A2(RA.20、RAT.19、RG.92、Am.6) | RA、RAT、RG、Am | quercetin | CXCR1 |
| A2(RA.20、RAT.19、RG.92、Am.6) | RA、RAT、RG、Am | quercetin | CAMK2B |
| A2(RA.20、RAT.19、RG.92、Am.6) | RA、RAT、RG、Am | quercetin | ALK |
| A2(RA.20、RAT.19、RG.92、Am.6) | RA、RAT、RG、Am | quercetin | AKT1 |
| A2(RA.20、RAT.19、RG.92、Am.6) | RA、RAT、RG、Am | quercetin | ABCB1 |
| A2(RA.20、RAT.19、RG.92、Am.6) | RA、RAT、RG、Am | quercetin | NEK6 |
| A2(RA.20、RAT.19、RG.92、Am.6) | RA、RAT、RG、Am | quercetin | PLA2G1B |
| A2(RA.20、RAT.19、RG.92、Am.6) | RA、RAT、RG、Am | quercetin | CA5A |
| A2(RA.20、RAT.19、RG.92、Am.6) | RA、RAT、RG、Am | quercetin | BACE1 |
| A2(RA.20、RAT.19、RG.92、Am.6) | RA、RAT、RG、Am | quercetin | CYP1B1 |
| A2(RA.20、RAT.19、RG.92、Am.6) | RA、RAT、RG、Am | quercetin | AXL |
| A2(RA.20、RAT.19、RG.92、Am.6) | RA、RAT、RG、Am | quercetin | ABCG2 |
| A2(RA.20、RAT.19、RG.92、Am.6) | RA、RAT、RG、Am | quercetin | NUAK1 |
| A2(RA.20、RAT.19、RG.92、Am.6) | RA、RAT、RG、Am | quercetin | AKR1C2 |
| A2(RA.20、RAT.19、RG.92、Am.6) | RA、RAT、RG、Am | quercetin | AKR1C1 |
| A2(RA.20、RAT.19、RG.92、Am.6) | RA、RAT、RG、Am | quercetin | AKR1C3 |
| A2(RA.20、RAT.19、RG.92、Am.6) | RA、RAT、RG、Am | quercetin | AKR1C4 |
| A2(RA.20、RAT.19、RG.92、Am.6) | RA、RAT、RG、Am | quercetin | CA13 |
| A2(RA.20、RAT.19、RG.92、Am.6) | RA、RAT、RG、Am | quercetin | AKR1A1 |
| A2(RA.20、RAT.19、RG.92、Am.6) | RA、RAT、RG、Am | quercetin | GPR35 |
| A2(RA.20、RAT.19、RG.92、Am.6) | RA、RAT、RG、Am | quercetin | SYK |
| A2(RA.20、RAT.19、RG.92、Am.6) | RA、RAT、RG、Am | quercetin | MAPT |
| A2(RA.20、RAT.19、RG.92、Am.6) | RA、RAT、RG、Am | quercetin | KDM4E |
| A2(RA.20、RAT.19、RG.92、Am.6) | RA、RAT、RG、Am | quercetin | TOP2A |
| A2(RA.20、RAT.19、RG.92、Am.6) | RA、RAT、RG、Am | quercetin | INSR |
| A2(RA.20、RAT.19、RG.92、Am.6) | RA、RAT、RG、Am | quercetin | ACHE |
| A2(RA.20、RAT.19、RG.92、Am.6) | RA、RAT、RG、Am | quercetin | MYLK |
| A2(RA.20、RAT.19、RG.92、Am.6) | RA、RAT、RG、Am | quercetin | PIK3CG |
| A2(RA.20、RAT.19、RG.92、Am.6) | RA、RAT、RG、Am | quercetin | APEX1 |
| A2(RA.20、RAT.19、RG.92、Am.6) | RA、RAT、RG、Am | quercetin | CDK5 |
| A2(RA.20、RAT.19、RG.92、Am.6) | RA、RAT、RG、Am | quercetin | CDK5R1 |
| A2(RA.20、RAT.19、RG.92、Am.6) | RA、RAT、RG、Am | quercetin | CCNB2 |
| A2(RA.20、RAT.19、RG.92、Am.6) | RA、RAT、RG、Am | quercetin | CCNB1 |
| A2(RA.20、RAT.19、RG.92、Am.6) | RA、RAT、RG、Am | quercetin | CDK1 |
| A2(RA.20、RAT.19、RG.92、Am.6) | RA、RAT、RG、Am | quercetin | CCNB3 |
| A2(RA.20、RAT.19、RG.92、Am.6) | RA、RAT、RG、Am | quercetin | ARG1 |
| A2(RA.20、RAT.19、RG.92、Am.6) | RA、RAT、RG、Am | quercetin | PTPRS |
| A2(RA.20、RAT.19、RG.92、Am.6) | RA、RAT、RG、Am | quercetin | ESR2 |
| A2(RA.20、RAT.19、RG.92、Am.6) | RA、RAT、RG、Am | quercetin | MPG |
| A2(RA.20、RAT.19、RG.92、Am.6) | RA、RAT、RG、Am | quercetin | SLC22A12 |
| A2(RA.20、RAT.19、RG.92、Am.6) | RA、RAT、RG、Am | quercetin | CDK6 |
| A2(RA.20、RAT.19、RG.92、Am.6) | RA、RAT、RG、Am | quercetin | CDK2 |
| B(SC.5、Sa.2、AK.4、RG.8) | SC、Sa、AK、RG | sitosterol | NPC1L1 |
| B(SC.5、Sa.2、AK.4、RG.8) | SC、Sa、AK、RG | sitosterol | NR1H3 |
| B(SC.5、Sa.2、AK.4、RG.8) | SC、Sa、AK、RG | sitosterol | RORC |
| B(SC.5、Sa.2、AK.4、RG.8) | SC、Sa、AK、RG | sitosterol | CYP17A1 |
| C(Sa.1、AK.3、RAT.13、Am.8) | Sa、AK、RAT、Am | beta-sitosterol | NPC1L1 |
| C(Sa.1、AK.3、RAT.13、Am.8) | Sa、AK、RAT、Am | beta-sitosterol | NR1H3 |
| C(Sa.1、AK.3、RAT.13、Am.8) | Sa、AK、RAT、Am | beta-sitosterol | RORC |
| C(Sa.1、AK.3、RAT.13、Am.8) | Sa、AK、RAT、Am | beta-sitosterol | HMGCR |
| D1(RC.7、RAT.16、RP.2) | RC、RAT、RP | Spinasterol | AR |
| D2(RC.10、RAT.18、RP.5) | RC、RAT、RP | luteolin | NOX4 |
| D2(RC.10、RAT.18、RP.5) | RC、RAT、RP | luteolin | AKR1B1 |
| D2(RC.10、RAT.18、RP.5) | RC、RAT、RP | luteolin | CDK5 |
| D2(RC.10、RAT.18、RP.5) | RC、RAT、RP | luteolin | CDK5R1 |
| D2(RC.10、RAT.18、RP.5) | RC、RAT、RP | luteolin | XDH |
| D2(RC.10、RAT.18、RP.5) | RC、RAT、RP | luteolin | MAOA |
| D2(RC.10、RAT.18、RP.5) | RC、RAT、RP | luteolin | FLT3 |
| D2(RC.10、RAT.18、RP.5) | RC、RAT、RP | luteolin | CA2 |
| D2(RC.10、RAT.18、RP.5) | RC、RAT、RP | luteolin | CCNB2 |
| D2(RC.10、RAT.18、RP.5) | RC、RAT、RP | luteolin | CCNB1 |
| D2(RC.10、RAT.18、RP.5) | RC、RAT、RP | luteolin | CDK1 |
| D2(RC.10、RAT.18、RP.5) | RC、RAT、RP | luteolin | CCNB3 |
| D2(RC.10、RAT.18、RP.5) | RC、RAT、RP | luteolin | ALOX5 |
| D2(RC.10、RAT.18、RP.5) | RC、RAT、RP | luteolin | ADORA1 |
| D2(RC.10、RAT.18、RP.5) | RC、RAT、RP | luteolin | CA7 |
| D2(RC.10、RAT.18、RP.5) | RC、RAT、RP | luteolin | GLO1 |
| D2(RC.10、RAT.18、RP.5) | RC、RAT、RP | luteolin | APP |
| D2(RC.10、RAT.18、RP.5) | RC、RAT、RP | luteolin | SYK |
| D2(RC.10、RAT.18、RP.5) | RC、RAT、RP | luteolin | GSK3B |
| D2(RC.10、RAT.18、RP.5) | RC、RAT、RP | luteolin | PARP1 |
| D2(RC.10、RAT.18、RP.5) | RC、RAT、RP | luteolin | TTR |
| D2(RC.10、RAT.18、RP.5) | RC、RAT、RP | luteolin | MMP9 |
| D2(RC.10、RAT.18、RP.5) | RC、RAT、RP | luteolin | CA12 |
| D2(RC.10、RAT.18、RP.5) | RC、RAT、RP | luteolin | MMP2 |
| D2(RC.10、RAT.18、RP.5) | RC、RAT、RP | luteolin | CA4 |
| D2(RC.10、RAT.18、RP.5) | RC、RAT、RP | luteolin | MMP12 |
| D2(RC.10、RAT.18、RP.5) | RC、RAT、RP | luteolin | CD38 |
| D2(RC.10、RAT.18、RP.5) | RC、RAT、RP | luteolin | CYP1B1 |
| D2(RC.10、RAT.18、RP.5) | RC、RAT、RP | luteolin | ABCG2 |
| D2(RC.10、RAT.18、RP.5) | RC、RAT、RP | luteolin | AKR1B10 |
| D2(RC.10、RAT.18、RP.5) | RC、RAT、RP | luteolin | TNKS2 |
| D2(RC.10、RAT.18、RP.5) | RC、RAT、RP | luteolin | TNKS |
| D2(RC.10、RAT.18、RP.5) | RC、RAT、RP | luteolin | TOP1 |
| D2(RC.10、RAT.18、RP.5) | RC、RAT、RP | luteolin | ARG1 |
| D2(RC.10、RAT.18、RP.5) | RC、RAT、RP | luteolin | PTPRS |
| D2(RC.10、RAT.18、RP.5) | RC、RAT、RP | luteolin | ABCC1 |
| D2(RC.10、RAT.18、RP.5) | RC、RAT、RP | luteolin | HSD17B1 |
| D2(RC.10、RAT.18、RP.5) | RC、RAT、RP | luteolin | ACHE |
| D2(RC.10、RAT.18、RP.5) | RC、RAT、RP | luteolin | CDK6 |
| D2(RC.10、RAT.18、RP.5) | RC、RAT、RP | luteolin | ABCB1 |
| E(RA.5、RAT.12、RG.7) | RA、RAT、RG | isorhamnetin | XDH |
| E(RA.5、RAT.12、RG.7) | RA、RAT、RG | isorhamnetin | CA2 |
| E(RA.5、RAT.12、RG.7) | RA、RAT、RG | isorhamnetin | CA7 |
| E(RA.5、RAT.12、RG.7) | RA、RAT、RG | isorhamnetin | CA12 |
| E(RA.5、RAT.12、RG.7) | RA、RAT、RG | isorhamnetin | CA4 |
| E(RA.5、RAT.12、RG.7) | RA、RAT、RG | isorhamnetin | CYP1B1 |
| F(RA.3、Po.14) | RA、Po | hederagenin | AR |
| F(RA.3、Po.14) | RA、Po | hederagenin | HMGCR |
| F(RA.3、Po.14) | RA、Po | hederagenin | CYP51A1 |
| F(RA.3、Po.14) | RA、Po | hederagenin | NPC1L1 |
| F(RA.3、Po.14) | RA、Po | hederagenin | NR1H3 |
| F(RA.3、Po.14) | RA、Po | hederagenin | CYP19A1 |
| G1(RA.1、RG.3) | RA、RG | Mairin | SAE1 |
| G1(RA.1、RG.3) | RA、RG | Mairin | UBA2 |
| G1(RA.1、RG.3) | RA、RG | Mairin | POLB |
| G1(RA.1、RG.3) | RA、RG | Mairin | AKR1B10 |
| G1(RA.1、RG.3) | RA、RG | Mairin | PTPN1 |
| G2(RA.2、RG.5) | RA、RG | Jaranol | ADORA1 |
| G2(RA.2、RG.5) | RA、RG | Jaranol | ADORA3 |
| G3(RA.12、RG.11) | RA、RG | formononetin | IL2 |
| H(RA.4、RAM.5) | RA、RAM | (3S,8S,9S,10R,13R,14S,17R)-10,13-dimethyl-17-[(2R,5S)-5-propan-2-yloctan-2-yl]-2,3,4,7,8,9,11,12,14,15,16,17-dodecahydro-1H-cyclopenta[a]phenanthren-3-ol | NPC1L1 |
| H(RA.4、RAM.5) | RA、RAM | (3S,8S,9S,10R,13R,14S,17R)-10,13-dimethyl-17-[(2R,5S)-5-propan-2-yloctan-2-yl]-2,3,4,7,8,9,11,12,14,15,16,17-dodecahydro-1H-cyclopenta[a]phenanthren-3-ol | RORC |
| H(RA.4、RAM.5) | RA、RAM | (3S,8S,9S,10R,13R,14S,17R)-10,13-dimethyl-17-[(2R,5S)-5-propan-2-yloctan-2-yl]-2,3,4,7,8,9,11,12,14,15,16,17-dodecahydro-1H-cyclopenta[a]phenanthren-3-ol | NR1H3 |
| H(RA.4、RAM.5) | RA、RAM | (3S,8S,9S,10R,13R,14S,17R)-10,13-dimethyl-17-[(2R,5S)-5-propan-2-yloctan-2-yl]-2,3,4,7,8,9,11,12,14,15,16,17-dodecahydro-1H-cyclopenta[a]phenanthren-3-ol | AR |
| I(RC.4、RAT.11) | RC、RAT | ZINC03978781 | AR |
| I(RC.4、RAT.11) | RC、RAT | ZINC03978781 | NPC1L1 |
| I(RC.4、RAT.11) | RC、RAT | ZINC03978781 | NR1H3 |
| I(RC.4、RAT.11) | RC、RAT | ZINC03978781 | RORC |
| I(RC.4、RAT.11) | RC、RAT | ZINC03978781 | HMGCR |
| I(RC.4、RAT.11) | RC、RAT | ZINC03978781 | CYP51A1 |
| J(RC.5、SC.6) | RC、SC | Stigmasterol | NPC1L1 |
| J(RC.5、SC.6) | RC、SC | Stigmasterol | NR1H3 |
| J(RC.5、SC.6) | RC、SC | Stigmasterol | RORC |
| Am.2 | Am | apigenin | NOX4 |
| Am.2 | Am | apigenin | AKR1B1 |
| Am.2 | Am | apigenin | CDK5 |
| Am.2 | Am | apigenin | CDK5R1 |
| Am.2 | Am | apigenin | XDH |
| Am.2 | Am | apigenin | MAOA |
| Am.2 | Am | apigenin | FLT3 |
| Am.2 | Am | apigenin | CYP19A1 |
| Am.2 | Am | apigenin | CCNB2 |
| Am.2 | Am | apigenin | CCNB1 |
| Am.2 | Am | apigenin | CDK1 |
| Am.2 | Am | apigenin | ESR1 |
| Am.2 | Am | apigenin | CCNB3 |
| Am.2 | Am | apigenin | ACHE |
| Am.2 | Am | apigenin | ADORA1 |
| Am.2 | Am | apigenin | PTGS2 |
| Am.2 | Am | apigenin | ESR2 |
| Am.2 | Am | apigenin | CDK6 |
| Am.2 | Am | apigenin | ADORA2A |
| Am.2 | Am | apigenin | SYK |
| Am.2 | Am | apigenin | GSK3B |
| Am.2 | Am | apigenin | ABCC1 |
| Am.2 | Am | apigenin | HSD17B1 |
| Am.2 | Am | apigenin | TTR |
| Am.2 | Am | apigenin | CSNK2A1 |
| Am.2 | Am | apigenin | CFTR |
| Am.2 | Am | apigenin | CYP1B1 |
| Am.2 | Am | apigenin | ABCG2 |
| Am.2 | Am | apigenin | AKR1B10 |
| Am.2 | Am | apigenin | TNKS2 |
| Am.2 | Am | apigenin | TNKS |
| Am.2 | Am | apigenin | ALOX5 |
| Am.2 | Am | apigenin | PARP1 |
| Am.2 | Am | apigenin | CA2 |
| Am.2 | Am | apigenin | CA7 |
| Am.2 | Am | apigenin | CA12 |
| Am.2 | Am | apigenin | ABCB1 |
| Po.2 | Po | trametenolic acid | PTGES |
| Po.6 | Po | ergosta-7,22E-dien-3beta-ol | AR |
| Po.7 | Po | Ergosterol peroxide | NOS2 |
| Po.9 | Po | 3beta-Hydroxy-24-methylene-8-lanostene-21-oic acid | PTGES |
| RA.16 | RA | FA | TYMS |
| RAM.4 | RAM | α-Amyrin | AR |
| RAT.10 | RAT | galangin | BCHE |
| RAT.10 | RAT | galangin | XDH |
| RAT.10 | RAT | galangin | ACHE |
| RAT.10 | RAT | galangin | ADORA1 |
| RAT.10 | RAT | galangin | CA7 |
| RAT.10 | RAT | galangin | ADORA2A |
| RAT.10 | RAT | galangin | ADORA3 |
| RAT.10 | RAT | galangin | CA12 |
| RAT.10 | RAT | galangin | CA4 |
| RAT.10 | RAT | galangin | ABCB1 |
| RAT.10 | RAT | galangin | CYP1B1 |
| RAT.10 | RAT | galangin | AKR1B1 |
| RAT.17 | RAT | 5,7-dihydroxy-2-(3-hydroxy-4-methoxyphenyl)chroman-4-one | CYP1B1 |
| RC.1 | RC | poriferasta-7,22E-dien-3beta-ol | AR |
| RC.18 | RC | glycitein | EGFR |
| RC.20 | RC | (8S,9S,10R,13R,14S,17R)-17-[(E,2R,5S)-5-ethyl-6-methylhept-3-en-2-yl]-10,13-dimethyl-1,2,4,7,8,9,11,12,14,15,16,17-dodecahydrocyclopenta[a]phenanthren-3-one | CTSD |
| RG.14 | RG | naringenin | CYP19A1 |
| RG.14 | RG | naringenin | CA7 |
| RG.14 | RG | naringenin | ABCC1 |
| RG.14 | RG | naringenin | HSD17B1 |
| RG.14 | RG | naringenin | CA12 |
| RG.14 | RG | naringenin | SHBG |
| RG.14 | RG | naringenin | CA4 |
| RG.14 | RG | naringenin | CYP1B1 |
| RG.14 | RG | naringenin | CBR1 |
| RG.14 | RG | naringenin | ESR1 |
| RG.14 | RG | naringenin | ESR2 |
| RG.19 | RG | Glyasperin C | TYR |
| RG.2 | RG | DFV | CYP19A1 |
| RG.2 | RG | DFV | ESR1 |
| RG.2 | RG | DFV | ESR2 |
| RG.2 | RG | DFV | HSD17B1 |
| RG.20 | RG | Isotrifoliol | ALOX5 |
| RG.21 | RG | (E)-1-(2,4-dihydroxyphenyl)-3-(2,2-dimethylchromen-6-yl)prop-2-en-1-one | ODC1 |
| RG.25 | RG | Glepidotin A | ABCB1 |
| RG.28 | RG | Glypallichalcone | MAOB |
| RG.39 | RG | 2-(3,4-dihydroxyphenyl)-5,7-dihydroxy-6-(3-methylbut-2-enyl)chromone | AKT1 |
| RG.45 | RG | shinpterocarpin | PTPN1 |
| RG.52 | RG | Glabranin | ESR1 |
| RG.52 | RG | Glabranin | ESR2 |
| RG.52 | RG | Glabranin | CYP19A1 |
| RG.61 | RG | (2R)-7-hydroxy-2-(4-hydroxyphenyl)chroman-4-one | CYP19A1 |
| RG.62 | RG | (2S)-7-hydroxy-2-(4-hydroxyphenyl)-8-(3-methylbut-2-enyl)chroman-4-one | ESR1 |
| RG.62 | RG | (2S)-7-hydroxy-2-(4-hydroxyphenyl)-8-(3-methylbut-2-enyl)chroman-4-one | ESR2 |
| RG.62 | RG | (2S)-7-hydroxy-2-(4-hydroxyphenyl)-8-(3-methylbut-2-enyl)chroman-4-one | ABCG2 |
| RG.62 | RG | (2S)-7-hydroxy-2-(4-hydroxyphenyl)-8-(3-methylbut-2-enyl)chroman-4-one | CYP19A1 |
| RG.64 | RG | Isolicoflavonol | CYP19A1 |
| RG.67 | RG | Quercetin der. | AKR1B1 |
| RG.78 | RG | 8-prenylated eriodictyol | ABCG2 |
| RG.78 | RG | 8-prenylated eriodictyol | ESR1 |
| RG.79 | RG | gadelaidic acid | PPARA |
| RG.79 | RG | gadelaidic acid | PPARD |
| RG.9 | RG | Lupiwighteone | PTPN1 |
| RG.93 | RG | Pinocembrin | CYP19A1 |
| RG.93 | RG | Pinocembrin | CYP1B1 |
| RO.13 | RO | stigmasterol-beta-d-glucoside | IL2 |
| RP.1 | RP | acacetin | CYP1B1 |
| RP.1 | RP | acacetin | CYP19A1 |
| Sa.3 | Sa | meso-1,4-Bis-(4-hydroxy-3-methoxyphenyl)-2,3-dimethylbutane | TTR |
| Sa.3 | Sa | meso-1,4-Bis-(4-hydroxy-3-methoxyphenyl)-2,3-dimethylbutane | ALOX15 |
| Sa.3 | Sa | meso-1,4-Bis-(4-hydroxy-3-methoxyphenyl)-2,3-dimethylbutane | NR3C1 |
| Sa.4 | Sa | 2-(4-hydroxyphenyl)ethyl(E)-3-(4-hydroxyphenyl)prop-2-enoate | CA7 |
| Sa.4 | Sa | 2-(4-hydroxyphenyl)ethyl(E)-3-(4-hydroxyphenyl)prop-2-enoate | CA1 |
| Sa.4 | Sa | 2-(4-hydroxyphenyl)ethyl(E)-3-(4-hydroxyphenyl)prop-2-enoate | CA6 |
| Sa.4 | Sa | 2-(4-hydroxyphenyl)ethyl(E)-3-(4-hydroxyphenyl)prop-2-enoate | CA14 |
| Sa.4 | Sa | 2-(4-hydroxyphenyl)ethyl(E)-3-(4-hydroxyphenyl)prop-2-enoate | CA9 |
| Sa.4 | Sa | 2-(4-hydroxyphenyl)ethyl(E)-3-(4-hydroxyphenyl)prop-2-enoate | CA4 |
| Sa.4 | Sa | 2-(4-hydroxyphenyl)ethyl(E)-3-(4-hydroxyphenyl)prop-2-enoate | CA5B |
| Sa.4 | Sa | 2-(4-hydroxyphenyl)ethyl(E)-3-(4-hydroxyphenyl)prop-2-enoate | CA5A |
| Sa.4 | Sa | 2-(4-hydroxyphenyl)ethyl(E)-3-(4-hydroxyphenyl)prop-2-enoate | AKR1B10 |
| Sa.4 | Sa | 2-(4-hydroxyphenyl)ethyl(E)-3-(4-hydroxyphenyl)prop-2-enoate | AKR1B1 |
| SC.9 | SC | CLR | NPC1L1 |
| SC.9 | SC | CLR | NR1H3 |
| SC.9 | SC | CLR | RORC |

Abbreviation notes: RA-Radix Astragali, Astragalus mongholicus Bunge [Fabaceae], RC-Radix Codonopsis, Codonopsis pilosula [Campanulaceae], RAM-Rhizoma Atractylodis Macrocephalae [Asteraceae], Po-Poria cocos, Wolfiporia extensa [Polyporaceae], SC-Semen Coicis, Coix lacryma-jobi [Poaceae], RO-Radix Ophiopogonis, Ophiopogon japonicus [Asparagaceae], AK-Akebia trifoliata Koidz, Akebia quinata [Lardizabalaceae], Am-Ampelopsis sinica, Ampelopsis glandulosa [Vitaceae], Sa-Sargentodoxa cuneate, Sargentodoxa cuneata (Oliv.) Rehd. et wils. [Lardizabalaceae], RAT-Radix Asteris Tatarici, Aster tataricus [Asteraceae], RP-Radix Platycodonis, Platycodon grandiflorus [Campanulaceae], and RG-Radix Glycyrrhiza, Glycyrrhiza uralensis [Fabaceae].
