# Supplementary material for: Study on the Action Mechanism of the Yifei Jianpi Tongfu Formula in Treatment of Colorectal Cancer Lung Metastasis Based on Network Analysis, Molecular Docking, and Experimental Validation
Source: Evid Based Complement Alternat Med. 2022 Jul 30;2022:6229444. doi: 10.1155/2022/6229444 (PMC9356795; doi:10.1155/2022/6229444)
Supplement: Supplementary Materials — Detailed information about the active compounds and targets identified in YJTF is shown in Supplementary Table 1. All of the disease-related targets for CRC lung metastasis are listed in Supplementary Table 2. Detailed information about the 81 overlapping targets identified as the key targets for studying the therapeutic effect of YJTF on CRC lung metastasis is shown in Supplementary Table 3. Detailed information about the PPI network is shown in Supplementary Table 4. Detailed information about the GO and KEGG enrichment analysis of the putative targets is shown in Supplementary Table 5. [file 6229444.f1.zip › Supplementary Table.2-revised.docx]

| Disease Targets | |
| --- | --- |
| Gene | Description |
| TP53 | Cellular tumor antigen p53 |
| BRCA2 | Breast cancer type 2 susceptibility protein |
| EGFR | Epidermal growth factor receptor |
| APC | Adenomatous polyposis coli protein |
| MLH1 | DNA mismatch repair protein Mlh1 |
| KRAS | GTPase KRas |
| BRCA1 | Breast cancer type 1 susceptibility protein |
| BRAF | Serine/threonine-protein kinase B-raf |
| PTEN | Phosphatidylinositol 3,4,5-trisphosphate 3-phosphatase and dual-specificity protein phosphatase PTEN |
| MSH2 | DNA mismatch repair protein Msh2 |
| CDH1 | Cadherin-1 |
| MSH6 | DNA mismatch repair protein Msh6 |
| CTNNB1 | Catenin beta-1 |
| ERBB2 | Receptor tyrosine-protein kinase erbB-2 |
| ATM | Serine-protein kinase ATM |
| CDKN2A | Tumor suppressor ARF |
| VEGFA | Vascular endothelial growth factor A |
| PIK3CA | Phosphatidylinositol 4,5-bisphosphate 3-kinase catalytic subunit alpha isoform |
| AKT1 | RAC-alpha serine/threonine-protein kinase |
| IL6 | Interleukin-6 |
| CCND1 | G1/S-specific cyclin-D1 |
| CHEK2 | Serine/threonine-protein kinase Chk2 |
| TNF | Tumor necrosis factor |
| SMAD4 | Mothers against decapentaplegic homolog 4 |
| MYC | Myc proto-oncogene protein |
| TGFB1 | Transforming growth factor beta-1 proprotein |
| AXIN2 | Axin-2 |
| STK11 | Serine/threonine-protein kinase STK11 |
| STAT3 | Signal transducer and activator of transcription 3 |
| TGFBR2 | TGF-beta receptor type-2 |
| RB1 | Retinoblastoma-associated protein |
| TERT | Telomerase reverse transcriptase |
| HRAS | GTPase HRas |
| MET | Hepatocyte growth factor receptor |
| CDKN1A | Cyclin-dependent kinase inhibitor 1 |
| EGF | Pro-epidermal growth factor |
| ESR1 | Estrogen receptor |
| CXCR4 | C-X-C chemokine receptor type 4 |
| PMS2 | Mismatch repair endonuclease PMS2 |
| BAX | Apoptosis regulator BAX |
| MIR21 | MicroRNA 21 |
| MDM2 | E3 ubiquitin-protein ligase Mdm2 |
| MTOR | Serine/threonine-protein kinase mTOR |
| EPCAM | Epithelial cell adhesion molecule |
| IL10 | Interleukin-10 |
| CDK4 | Cyclin-dependent kinase 4 |
| NRAS | GTPase NRas |
| CDKN1B | Cyclin-dependent kinase inhibitor 1B |
| SRC | Proto-oncogene tyrosine-protein kinase Src |
| RET | Proto-oncogene tyrosine-protein kinase receptor Ret |
| POLE | DNA polymerase epsilon catalytic subunit A |
| FAS | Tumor necrosis factor receptor superfamily member 6 |
| CASP8 | Caspase-8 |
| NOTCH1 | Neurogenic locus notch homolog protein 1 |
| SMAD3 | Mothers against decapentaplegic homolog 3 |
| MMP9 | Matrix metalloproteinase-9 |
| NFKB1 | Nuclear factor NF-kappa-B p105 subunit |
| ALK | ALK tyrosine kinase receptor |
| MMP1 | Interstitial collagenase |
| FGFR3 | Fibroblast growth factor receptor 3 |
| FGFR2 | Fibroblast growth factor receptor 2 |
| TYMS | Thymidylate synthase |
| CAV1 | Caveolin-1 |
| PLAU | Urokinase-type plasminogen activator |
| IFNG | Interferon gamma |
| SPP1 | Osteopontin |
| PTGS2 | Prostaglandin G/H synthase 2 |
| EP300 | Histone acetyltransferase p300 |
| CXCL12 | Stromal cell-derived factor 1 |
| MMP2 | 72 kDa type IV collagenase |
| FHIT | Bis(5'-adenosyl)-triphosphatase |
| EZH2 | Histone-lysine N-methyltransferase EZH2 |
| IGF2 | Insulin-like growth factor II |
| NF1 | Neurofibromin |
| MAP2K1 | Dual specificity mitogen-activated protein kinase kinase 1 |
| KIT | Mast/stem cell growth factor receptor Kit |
| FASLG | Tumor necrosis factor ligand superfamily member 6 |
| H19 | H19 Imprinted Maternally Expressed Transcript |
| PDGFRB | Platelet-derived growth factor receptor beta |
| PPARG | Peroxisome proliferator-activated receptor gamma |
| ERCC1 | DNA excision repair protein ERCC-1 |
| NKX2-1 | Homeobox protein Nkx-2.1 |
| STAT1 | Signal transducer and activator of transcription 1-alpha/beta |
| MIR145 | MicroRNA 145 |
| NFE2L2 | Nuclear factor erythroid 2-related factor 2 |
| TYMP | Thymidine phosphorylase |
| AURKA | Aurora kinase A |
| ERBB3 | Receptor tyrosine-protein kinase erbB-3 |
| RAF1 | RAF proto-oncogene serine/threonine-protein kinase |
| ESR2 | Estrogen receptor beta |
| PIK3R1 | Phosphatidylinositol 3-kinase regulatory subunit alpha |
| MIR17 | MicroRNA 17 |
| DNMT1 | DNA (cytosine-5)-methyltransferase 1 |
| FLT1 | Vascular endothelial growth factor receptor 1 |
| XIAP | E3 ubiquitin-protein ligase XIAP |
| TWIST1 | Twist-related protein 1 |
| MIR34A | MicroRNA 34a |
| ERCC2 | General transcription and DNA repair factor IIH helicase subunit XPD |
| AKT2 | RAC-beta serine/threonine-protein kinase |
| BCL2 | Apoptosis regulator Bcl-2 |
| RARB | Retinoic acid receptor beta |
| SNAI2 | Zinc finger protein SNAI2 |
| CCR6 | C-C chemokine receptor type 6 |
| PROM1 | Prominin-1 |
| SOX2 | Transcription factor SOX-2 |
| ZEB1 | Zinc finger E-box-binding homeobox 1 |
| TNFRSF10B | Tumor necrosis factor receptor superfamily member 10B |
| RELA | Transcription factor p65 |
| RAD51 | DNA repair protein RAD51 homolog 1 |
| TOP2A | DNA topoisomerase 2-alpha |
| FGFR1 | Fibroblast growth factor receptor 1 |
| NOS2 | Nitric oxide synthase, inducible |
| CXCL8 | Interleukin-8 |
| SLC2A1 | Solute carrier family 2, facilitated glucose transporter member 1 |
| GLI1 | Zinc finger protein GLI1 |
| CDKN2B | Cyclin-dependent kinase 4 inhibitor B |
| MIR143 | MicroRNA 143 |
| MAPK1 | Mitogen-activated protein kinase 1 |
| OGG1 | N-glycosylase/DNA lyase |
| DICER1 | Endoribonuclease Dicer |
| MIR125A | MicroRNA 125a |
| DPYD | Dihydropyrimidine dehydrogenase |
| CREB1 | Cyclic AMP-responsive element-binding protein 1 |
| MIR126 | MicroRNA 126 |
| MUC1 | Mucin-1 |
| TP73 | Tumor protein p73 |
| MIR146A | MicroRNA 146a |
| MIR155 | MicroRNA 155 |
| MIR31 | MicroRNA 31 |
| CASP3 | Caspase-3 |
| HIF1A | Hypoxia-inducible factor 1-alpha |
| TGFBR1 | TGF-beta receptor type-1 |
| MIR200C | MicroRNA 200c |
| MIR221 | MicroRNA 221 |
| SMAD7 | Mothers against decapentaplegic homolog 7 |
| MGMT | Methylated-DNA--protein-cysteine methyltransferase |
| ERBB4 | Receptor tyrosine-protein kinase erbB-4 |
| MEG3 | Maternally Expressed 3 |
| MIR27A | MicroRNA 27a |
| MUTYH | Adenine DNA glycosylase |
| ICAM1 | Intercellular adhesion molecule 1 |
| MIR214 | MicroRNA 214 |
| CYP1B1 | Cytochrome P450 1B1 |
| BIRC5 | Baculoviral IAP repeat-containing protein 5 |
| MIR182 | MicroRNA 182 |
| INS | Insulin |
| MIR141 | MicroRNA 141 |
| MIR200A | MicroRNA 200a |
| IL1B | Interleukin-1 beta |
| GSTM1 | Glutathione S-transferase Mu 1 |
| MIR200B | MicroRNA 200b |
| CD44 | CD44 antigen |
| TLR4 | Toll-like receptor 4 |
| PDGFRA | Platelet-derived growth factor receptor alpha |
| MIR142 | MicroRNA 142 |
| MIR223 | MicroRNA 223 |
| MIR203A | MicroRNA 203a |
| MAP2K2 | Dual specificity mitogen-activated protein kinase kinase 2 |
| BIRC3 | Baculoviral IAP repeat-containing protein 3 |
| SETD2 | Histone-lysine N-methyltransferase SETD2 |
| MIR195 | MicroRNA 195 |
| GSTP1 | Glutathione S-transferase P |
| MTHFR | Methylenetetrahydrofolate reductase |
| CEACAM5 | Carcinoembryonic antigen-related cell adhesion molecule 5 |
| MIR20A | MicroRNA 20a |
| MIR29A | MicroRNA 29a |
| CD274 | Programmed cell death 1 ligand 1 |
| ODC1 | Ornithine decarboxylase |
| MIRLET7A1 | MicroRNA Let-7a-1 |
| JUN | Transcription factor AP-1 |
| NFKBIA | NF-kappa-B inhibitor alpha |
| MIR183 | MicroRNA 183 |
| ABCB1 | ATP-dependent translocase ABCB1 |
| BUB1 | Mitotic checkpoint serine/threonine-protein kinase BUB1 |
| VDR | Vitamin D3 receptor |
| MIR150 | MicroRNA 150 |
| IRS1 | Insulin receptor substrate 1 |
| PALB2 | Partner and localizer of BRCA2 |
| IL13 | Interleukin-13 |
| IGF1 | Insulin-like growth factor I |
| MIR181A1 | MicroRNA 181a-1 |
| DNMT3A | DNA (cytosine-5)-methyltransferase 3A |
| IGF1R | Insulin-like growth factor 1 receptor |
| FLCN | Folliculin |
| ENG | Endoglin |
| CHEK1 | Serine/threonine-protein kinase Chk1 |
| NBN | Nibrin |
| PIK3R2 | Phosphatidylinositol 3-kinase regulatory subunit beta |
| VEGFC | Vascular endothelial growth factor C |
| KLF6 | Krueppel-like factor 6 |
| ZEB2 | Zinc finger E-box-binding homeobox 2 |
| MIR25 | MicroRNA 25 |
| HSPB1 | Heat shock protein beta-1 |
| MMP7 | Matrilysin |
| MIR140 | MicroRNA 140 |
| AR | Androgen receptor |
| MIR193A | MicroRNA 193a |
| MIR100 | MicroRNA 100 |
| MIR34C | MicroRNA 34c |
| MIR144 | MicroRNA 144 |
| KDR | Vascular endothelial growth factor receptor 2 |
| CYP1A1 | Cytochrome P450 1A1 |
| MIR106B | MicroRNA 106b |
| MSH3 | DNA mismatch repair protein Msh3 |
| BCL2L1 | Bcl-2-like protein 1 |
| XRCC1 | DNA repair protein XRCC1 |
| MIR106A | MicroRNA 106a |
| MIR96 | MicroRNA 96 |
| TERC | Telomerase RNA component |
| MIR26A1 | MicroRNA 26a-1 |
| MIR23A | MicroRNA 23a |
| MIRLET7B | MicroRNA Let-7b |
| TGFB2 | Transforming growth factor beta-2 proprotein |
| TCF7L2 | Transcription factor 7-like 2 |
| MIR18A | MicroRNA 18a |
| MIR224 | MicroRNA 224 |
| WNT5A | Protein Wnt-5a |
| AKT3 | RAC-gamma serine/threonine-protein kinase |
| MIR205 | MicroRNA 205 |
| IL2 | Interleukin-2 |
| BMP2 | Bone morphogenetic protein 2 |
| MAPK3 | Mitogen-activated protein kinase 3 |
| PTK2 | Focal adhesion kinase 1 |
| TP63 | Tumor protein 63 |
| MYCN | N-myc proto-oncogene protein |
| NTRK1 | High affinity nerve growth factor receptor |
| KCNQ1OT1 | KCNQ1 Opposite Strand/Antisense Transcript 1 |
| CASP9 | Caspase-9 |
| MIR93 | MicroRNA 93 |
| MIR191 | MicroRNA 191 |
| HGF | Hepatocyte growth factor |
| MIR19A | MicroRNA 19a |
| MIR483 | MicroRNA 483 |
| TSC2 | Tuberin |
| MALAT1 | Metastasis-associated lung adenocarcinoma transcript 1 |
| MIR148A | MicroRNA 148a |
| FGF2 | Fibroblast growth factor 2 |
| SMAD2 | Mothers against decapentaplegic homolog 2 |
| IDH1 | Isocitrate dehydrogenase [NADP] cytoplasmic |
| WT1 | Wilms tumor protein |
| MIR10A | MicroRNA 10a |
| RASSF1 | Ras association domain-containing protein 1 |
| MIR424 | MicroRNA 424 |
| NME1 | Nucleoside diphosphate kinase A |
| MKI67 | Proliferation marker protein Ki-67 |
| ARAF | Serine/threonine-protein kinase A-Raf |
| MIR222 | MicroRNA 222 |
| FOXP3 | Forkhead box protein P3 |
| HMOX1 | Heme oxygenase 1 |
| KRT20 | Keratin, type I cytoskeletal 20 |
| CTNNA1 | Catenin alpha-1 |
| MIR15B | MicroRNA 15b |
| MMP14 | Matrix metalloproteinase-14 |
| XRCC3 | DNA repair protein XRCC3 |
| CCL2 | C-C motif chemokine 2 |
| PARP1 | Poly [ADP-ribose] polymerase 1 |
| KRT7 | Keratin, type II cytoskeletal 7 |
| MIR22 | MicroRNA 221 |
| MIR29B2 | MicroRNA 29b-2 |
| MIR32 | MicroRNA 32 |
| FN1 | Fibronectin |
| MIR34B | MicroRNA 34b |
| KRT19 | Keratin, type I cytoskeletal 19 |
| MAPK8 | Mitogen-activated protein kinase 8 |
| ETS1 | Protein C-ets-1 |
| CRP | C-reactive protein |
| MIR107 | MicroRNA 107 |
| ALB | Serum albumin |
| MIR15A | MicroRNA 15a |
| MIR296 | MicroRNA 296 |
| MPO | Myeloperoxidase |
| TNFSF10 | Tumor necrosis factor ligand superfamily member 10 |
| MIRLET7G | MicroRNA Let-7g |
| IGFBP3 | Insulin-like growth factor-binding protein 3 |
| SNAI1 | Zinc finger protein SNAI1 |
| MIR10B | MicroRNA 10b |
| RAD51C | DNA repair protein RAD51 homolog 3 |
| MIR99A | MicroRNA 99a |
| AHR | Aryl hydrocarbon receptor |
| MIR29C | MicroRNA 29c |
| MIR375 | MicroRNA 375 |
| MIR192 | MicroRNA 192 |
| SMARCA4 | Transcription activator BRG1 |
| TGFA | Protransforming growth factor alpha |
| MIR335 | MicroRNA 335 |
| PGR | Progesterone receptor |
| TOP1 | DNA topoisomerase 1 |
| PLAUR | Urokinase plasminogen activator surface receptor |
| WNT1 | Proto-oncogene Wnt-1 |
| ABCC1 | Multidrug resistance-associated protein 1 |
| MIR128-2 | MicroRNA 128-2 |
| GRP | Gastrin-releasing peptide |
| SP1 | Transcription factor Sp1 |
| PCNA | Proliferating cell nuclear antigen |
| NQO1 | NAD |
| ABCG2 | Broad substrate specificity ATP-binding cassette transporter ABCG2 |
| RAD51D | DNA repair protein RAD51 homolog 4 |
| PLG | Plasminogen |
| ITGB1 | Integrin beta-1 |
| CYCS | Cytochrome c |
| RAC1 | Ras-related C3 botulinum toxin substrate 1 |
| MIR24-2 | MicroRNA 24-2 |
| GNAS | Protein ALEX |
| TNFRSF1A | Tumor necrosis factor receptor superfamily member 1A |
| IL4 | Interleukin-4 |
| BMP4 | Bone morphogenetic protein 4 |
| FLT4 | Vascular endothelial growth factor receptor 3 |
| HMGA2 | High mobility group protein HMGI-C |
| TLR2 | Toll-like receptor 2 |
| TIMP2 | Metalloproteinase inhibitor 2 |
| RHOA | Transforming protein RhoA |
| E2F1 | Transcription factor E2F1 |
| HOTAIR | HOX Transcript Antisense RNA |
| VIM | Vimentin |
| CALR | Calreticulin |
| CDK2 | Cyclin-dependent kinase 2 |
| JAK2 | Tyrosine-protein kinase JAK2 |
| ROS1 | Proto-oncogene tyrosine-protein kinase ROS |
| BMP6 | Bone morphogenetic protein 6 |
| TSC1 | Hamartin |
| S100A4 | Protein S100-A4 |
| MIR30E | MicroRNA 30e |
| CDKN3 | Cyclin-dependent kinase inhibitor 3 |
| MAPK14 | Mitogen-activated protein kinase 14 |
| EPHB2 | Ephrin type-B receptor 2 |
| MUC5AC | Mucin-5AC |
| KRT18 | Keratin, type I cytoskeletal 18 |
| TIMP1 | Metalloproteinase inhibitor 1 |
| CTSB | Cathepsin B |
| YAP1 | Transcriptional coactivator YAP1 |
| ENO2 | Gamma-enolase |
| HSP90AA1 | Heat shock protein HSP 90-alpha |
| MCL1 | Induced myeloid leukemia cell differentiation protein Mcl-1 |
| BARD1 | BRCA1-associated RING domain protein 1 |
| BRIP1 | Fanconi anemia group J protein |
| CCNB1 | G2/mitotic-specific cyclin-B1 |
| KEAP1 | Kelch-like ECH-associated protein 1 |
| HDAC1 | Histone deacetylase 1 |
| MIR486-1 | MicroRNA 486-1 |
| VHL | von Hippel-Lindau disease tumor suppressor |
| CDK1 | Cyclin-dependent kinase 1 |
| CCNA2 | Cyclin-A2 |
| VEGFD | Vascular endothelial growth factor D |
| PDCD1 | Programmed cell death protein 1 |
| ABCC2 | Canalicular multispecific organic anion transporter 1 |
| MMP3 | Stromelysin-1 |
| PPP2R1B | Serine/threonine-protein phosphatase 2A 65 kDa regulatory subunit A beta isoform |
| THBS1 | Thrombospondin-1 |
| FOS | Proto-oncogene c-Fos |
| LOX | Protein-lysine 6-oxidase |
| PDGFB | Platelet-derived growth factor subunit B |
| PTPN11 | Tyrosine-protein phosphatase non-receptor type 11 |
| CYP17A1 | Steroid 17-alpha-hydroxylase/17,20 lyase |
| FOXM1 | Forkhead box protein M1 |
| ANXA5 | Annexin A5 |
| LGALS3 | Galectin-3 |
| LEP | Leptin |
| CTLA4 | Cytotoxic T-lymphocyte protein 4 |
| PTCH1 | Protein patched homolog 1 |
| UGT1A1 | UDP-glucuronosyltransferase 1-1 |
| CD82 | CD82 antigen |
| RPS6KB1 | Ribosomal protein S6 kinase beta-1 |
| MIR185 | MicroRNA 185 |
| BMI1 | Polycomb complex protein BMI-1 |
| AREG | Amphiregulin |
| PROS1 | Vitamin K-dependent protein S |
| PRSS1 | Trypsin-1 |
| BAK1 | Bcl-2 homologous antagonist/killer |
| MIR127 | MicroRNA 127 |
| CDH2 | Cadherin-2 |
| PLA2G2A | Phospholipase A2, membrane associated |
| MIR30A | MicroRNA 30a |
| SERPINB5 | Serpin B5 |
| HSPA5 | Endoplasmic reticulum chaperone BiP |
| TIMP3 | Metalloproteinase inhibitor 3 |
| MRE11 | Double-strand break repair protein MRE11 |
| MEN1 | Menin |
| MIR320A | MicroRNA 320a |
| GJA1 | Gap junction alpha-1 protein |
| DNMT3B | DNA (cytosine-5)-methyltransferase 3B |
| BMPR1A | Bone morphogenetic protein receptor type-1A |
| CSF3 | Granulocyte colony-stimulating factor |
| CDK6 | Cyclin-dependent kinase 6 |
| NF2 | Merlin |
| CHGA | Chromogranin-A |
| PLK1 | Serine/threonine-protein kinase PLK1 |
| GSK3B | Glycogen synthase kinase-3 beta |
| BAP1 | Ubiquitin carboxyl-terminal hydrolase BAP1 |
| GRB2 | Growth factor receptor-bound protein 2 |
| KRT8 | Keratin, type II cytoskeletal 8 |
| HPGD | 15-hydroxyprostaglandin dehydrogenase [NAD(+)] |
| MIR199A1 | MicroRNA 199a-1 |
| GREM1 | Gremlin-1 |
| IFI27 | Interferon alpha-inducible protein 27, mitochondrial |
| IL1RN | Interleukin-1 receptor antagonist protein |
| MIR451A | MicroRNA 451a |
| MMP13 | Collagenase 3 |
| FOXO3 | Forkhead box protein O3 |
| MIR23B | MicroRNA 23b |
| CFLAR | CASP8 and FADD-like apoptosis regulator |
| MUC4 | Mucin-4 |
| CDC42 | Cell division control protein 42 homolog |
| KLK3 | Prostate-specific antigen |
| BCL2L11 | Bcl-2-like protein 11 |
| BUB1B | Mitotic checkpoint serine/threonine-protein kinase BUB1 beta |
| SOS1 | Son of sevenless homolog 1 |
| U2AF1 | Splicing factor U2AF 35 kDa subunit |
| HDAC9 | Histone deacetylase 9 |
| TNFRSF10A | Tumor necrosis factor receptor superfamily member 10A |
| FGFR4 | Fibroblast growth factor receptor 4 |
| MIR210 | MicroRNA 210 |
| PVT1 | Pvt1 Oncogene |
| MIR29B1 | MicroRNA 29b-1 |
| ADIPOQ | Adiponectin |
| NRP1 | Neuropilin-1 |
| PRKCA | Protein kinase C alpha type |
| EPHA2 | Ephrin type-A receptor 2 |
| SERPINE1 | Plasminogen activator inhibitor 1 |
| GDF15 | Growth/differentiation factor 15 |
| UCA1 | Urothelial Cancer Associated 1 |
| LGALS1 | Galectin-1 |
| CCN2 | CCN family member 2 |
| TCF3 | Transcription factor E2-alpha |
| GADD45A | Growth arrest and DNA damage-inducible protein GADD45 alpha |
| BAD | Bcl2-associated agonist of cell death |
| CFTR | Cystic fibrosis transmembrane conductance regulator |
| PIK3CG | Phosphatidylinositol 4,5-bisphosphate 3-kinase catalytic subunit gamma isoform |
| CA9 | Carbonic anhydrase 9 |
| CTSD | Cathepsin D |
| SYP | Synaptophysin |
| GAS5 | Growth Arrest Specific 5 |
| MIR204 | MicroRNA 204 |
| FSCN1 | Fascin |
| HPRT1 | Hypoxanthine-guanine phosphoribosyltransferase |
| PIK3R3 | Phosphatidylinositol 3-kinase regulatory subunit gamma |
| MUC16 | Mucin-16 |
| FASN | Fatty acid synthase |
| CAT | Catalase |
| CDX2 | Homeobox protein CDX-2 |
| IFNA1 | Interferon Alpha 1 |
| EZR | Ezrin |
| CD24 | Signal transducer CD24 |
| CYP3A4 | Cytochrome P450 3A4 |
| RECK | Reversion-inducing cysteine-rich protein with Kazal motifs |
| MIR137 | MicroRNA 137 |
| HLA-A | HLA class I histocompatibility antigen, A alpha chain |
| FOXO1 | Forkhead box protein O1 |
| TLR3 | Toll-like receptor 3 |
| CSF2 | Granulocyte-macrophage colony-stimulating factor |
| CXCL1 | Growth-regulated alpha protein |
| MIR133B | MicroRNA 133b |
| CASR | Extracellular calcium-sensing receptor |
| PXN | Paxillin |
| CTNND1 | Catenin delta-1 |
| TUG1 | Taurine Up-Regulated 1 |
| B2M | Beta-2-microglobulin |
| CYP19A1 | Aromatase |
| BECN1 | Beclin-1 |
| CXCR2 | C-X-C chemokine receptor type 2 |
| ILK | Integrin-linked protein kinase |
| NEAT1 | Nuclear Paraspeckle Assembly Transcript 1 |
| PIK3CB | Phosphatidylinositol 4,5-bisphosphate 3-kinase catalytic subunit beta isoform |
| CDKN2B-AS1 | CDKN2B Antisense RNA 1 |
| HMGB1 | High mobility group protein B1 |
| PRKN | E3 ubiquitin-protein ligase parkin |
| MTA1 | Metastasis-associated protein MTA1 |
| SOD2 | Superoxide dismutase [Mn], mitochondrial |
| CD40 | Tumor necrosis factor receptor superfamily member 5 |
| WRN | Werner syndrome ATP-dependent helicase |
| SDHB | Succinate dehydrogenase [ubiquinone] iron-sulfur subunit, mitochondrial |
| SOX9 | Transcription factor SOX-9 |
| NTRK3 | NT-3 growth factor receptor |
| XRCC2 | DNA repair protein XRCC2 |
| GAPDH | Glyceraldehyde-3-phosphate dehydrogenase |
| CASP7 | Caspase-7 |
| EGR1 | Early growth response protein 1 |
| PECAM1 | Platelet endothelial cell adhesion molecule |
| MIR9-1 | MicroRNA 9-1 |
| CCAT2 | Colon Cancer Associated Transcript 2 |
| CCND2 | G1/S-specific cyclin-D2 |
| MIR149 | MicroRNA 149 |
| MIR342 | MicroRNA 342 |
| MUC5B | Mucin-5B |
| SHH | Sonic hedgehog protein |
| MIR181A2 | MicroRNA 181a-2 |
| STAT5B | Signal transducer and activator of transcription 5B |
| SIRT1 | NAD-dependent protein deacetylase sirtuin-1 |
| AXIN1 | Axin-1 |
| CCND3 | G1/S-specific cyclin-D3 |
| MIR16-1 | MicroRNA 16-1 |
| NAT2 | Arylamine N-acetyltransferase 2 |
| TFAP2A | Transcription factor AP-2-alpha |
| CCNE1 | G1/S-specific cyclin-E1 |
| RUNX3 | Runt-related transcription factor 3 |
| MIRLET7D | MicroRNA Let-7d |
| MIR139 | MicroRNA 139 |
| HLA-G | HLA class I histocompatibility antigen, alpha chain G |
| IL17A | Interleukin-17A |
| ANXA2 | Annexin A2 |
| RAD50 | DNA repair protein RAD50 |
| SERPINA3 | Alpha-1-antichymotrypsin |
| CEACAM3 | Carcinoembryonic antigen-related cell adhesion molecule 3 |
| CCKBR | Gastrin/cholecystokinin type B receptor |
| ARID1A | AT-rich interactive domain-containing protein 1A |
| IFNA2 | Interferon alpha-2 |
| MIR124-1 | MicroRNA 124-1 |
| IGFBP2 | Insulin-like growth factor-binding protein 2 |
| CCAT1 | Colon cancer-associated transcript 1, noncoding |
| DCC | Netrin receptor DCC |
| MIR196A1 | MicroRNA 196a-1 |
| DIABLO | Diablo homolog, mitochondrial |
| XIST | X Inactive Specific Transcript |
| FH | Fumarate hydratase, mitochondrial |
| NOD2 | Nucleotide-binding oligomerization domain-containing protein 2 |
| EPHA3 | Ephrin type-A receptor 3 |
| RNASEL | 2-5A-dependent ribonuclease |
| TLR9 | Toll-like receptor 9 |
| GAST | Gastrin |
| F2 | Prothrombin |
| FBXW7 | F-box/WD repeat-containing protein 7 |
| GRPR | Gastrin-releasing peptide receptor |
| H2AC18 | Histone H2A type 2-A |
| HLA-DRB1 | HLA class II histocompatibility antigen, DRB1 beta chain |
| APEX1 | DNA-(apurinic or apyrimidinic site) endonuclease |
| PTGS1 | Prostaglandin G/H synthase 1 |
| XRCC6 | X-ray repair cross-complementing protein 6 |
| NTRK2 | BDNF/NT-3 growth factors receptor |
| ALOX5 | Arachidonate 5-lipoxygenase |
| MAD1L1 | Mitotic spindle assembly checkpoint protein MAD1 |
| KLF4 | Krueppel-like factor 4 |
| DAPK1 | Death-associated protein kinase 1 |
| MIR146B | MicroRNA 146b |
| JAK1 | Tyrosine-protein kinase JAK1 |
| CASC2 | Protein CASC2, isoform 3 |
| NOS3 | Nitric oxide synthase, endothelial |
| MIR373 | MicroRNA 373 |
| ITGA2 | Integrin alpha-2 |
| TLR5 | Toll-like receptor 5 |
| SOD1 | Superoxide dismutase [Cu-Zn] |
| MAP2K4 | Dual specificity mitogen-activated protein kinase kinase 4 |
| LINC-ROR | Long Intergenic Non-Protein Coding RNA, Regulator Of Reprogramming |
| HOTTIP | HOXA Distal Transcript Antisense RNA |
| COMT | Catechol O-methyltransferase |
| SPRY4-IT1 | SPRY4 Intronic Transcript 1 |
| EIF4E | Eukaryotic translation initiation factor 4E |
| HLA-B | HLA class I histocompatibility antigen, B alpha chain |
| IL6R | Interleukin-6 receptor subunit alpha |
| PMS1 | PMS1 protein homolog 1 |
| MIR199B | MicroRNA 199b |
| HDAC2 | Histone deacetylase 2 |
| CEACAM6 | Carcinoembryonic antigen-related cell adhesion molecule 6 |
| CDH13 | Cadherin-13 |
| MIR372 | MicroRNA 372 |
| PKM | Pyruvate kinase PKM |
| CYP2E1 | Cytochrome P450 2E1 |
| SPINK1 | Serine protease inhibitor Kazal-type 1 |
| VWF | von Willebrand factor |
| PCAT1 | Prostate cancer-associated transcript 1, noncoding |
| MIR497 | MicroRNA 497 |
| NCAM1 | Neural cell adhesion molecule 1 |
| IL2RA | Interleukin-2 receptor subunit alpha |
| DES | Desmin |
| CDC25C | M-phase inducer phosphatase 3 |
| IGF2R | Cation-independent mannose-6-phosphate receptor |
| WNT3 | Proto-oncogene Wnt-3 |
| GSTT1 | Glutathione S-transferase theta-1 |
| TG | Thyroglobulin |
| MMP11 | Stromelysin-3 |
| POU5F1 | POU domain, class 5, transcription factor 1 |
| E2F3 | Transcription factor E2F3 |
| JUP | Junction plakoglobin |
| AFAP1-AS1 | AFAP1 Antisense RNA 1 |
| MCC | Colorectal mutant cancer protein |
| TGFB3 | Transforming growth factor beta-3 proprotein |
| COL18A1 | Collagen alpha-1 |
| MIR30B | MicroRNA 30b |
| ITGA6 | Integrin alpha-6 |
| PDPN | Podoplanin |
| CD36 | Platelet glycoprotein 4 |
| KITLG | Kit ligand |
| MT-CO1 | Cytochrome c oxidase subunit 1 |
| BSG | Basigin |
| LEF1 | Lymphoid enhancer-binding factor 1 |
| SST | Somatostatin |
| MIRLET7C | MicroRNA Let-7c |
| SNHG1 | Small Nucleolar RNA Host Gene 1 |
| GLI3 | Transcriptional activator GLI3 |
| TRIM28 | Transcription intermediary factor 1-beta |
| ZFAS1 | ZNFX1 Antisense RNA 1 |
| IL4R | Interleukin-4 receptor subunit alpha |
| MIR24-1 | MicroRNA 24-1 |
| GFAP | Glial fibrillary acidic protein |
| SMARCB1 | SWI/SNF-related matrix-associated actin-dependent regulator of chromatin subfamily B member 1 |
| DLEC1 | Deleted in lung and esophageal cancer protein 1 |
| CCL5 | C-C motif chemokine 5 |
| EDNRA | Endothelin-1 receptor |
| DANCR | Putative uncharacterized protein DANCR |
| EDN1 | Endothelin-1 |
| SAT1 | Diamine acetyltransferase 1 |
| SKP2 | S-phase kinase-associated protein 2 |
| TXN | Thioredoxin |
| DKK1 | Dickkopf-related protein 1 |
| PTHLH | Parathyroid hormone-related protein |
| ICOSLG | ICOS ligand |
| E2F2 | Transcription factor E2F2 |
| RRM2 | Ribonucleoside-diphosphate reductase subunit M2 |
| SFTPC | Pulmonary surfactant-associated protein C |
| CCN1 | CCN family member 1 |
| TNFRSF1B | Tumor necrosis factor receptor superfamily member 1B |
| SELP | P-selectin |
| MMP12 | Macrophage metalloelastase |
| TEK | Angiopoietin-1 receptor |
| MIR27B | MicroRNA 27b |
| MIR193B | MicroRNA 193b |
| ACTC1 | Actin, alpha cardiac muscle 1 |
| MIRLET7I | MicroRNA Let-7i |
| ITGA3 | Integrin alpha-3 |
| SPARC | SPARC |
| ANXA1 | Annexin A1 |
| CSF1 | Macrophage colony-stimulating factor 1 |
| TIAM1 | T-lymphoma invasion and metastasis-inducing protein 1 |
| ELANE | Neutrophil elastase |
| MUC6 | Mucin-6 |
| GPC3 | Glypican-3 |
| BMPR2 | Bone morphogenetic protein receptor type-2 |
| MIR331 | MicroRNA 331 |
| PRKD1 | Serine/threonine-protein kinase D1 |
| TKT | Transketolase |
| POLK | DNA polymerase kappa |
| TCF7 | Transcription factor 7 |
| CREBBP | CREB-binding protein |
| PDCD4 | Programmed cell death protein 4 |
| ALDOA | Fructose-bisphosphate aldolase A |
| CADM1 | Cell adhesion molecule 1 |
| LAMC2 | Laminin subunit gamma-2 |
| SLC29A1 | Equilibrative nucleoside transporter 1 |
| HSPA4 | Heat shock 70 kDa protein 4 |
| DDR2 | Discoidin domain-containing receptor 2 |
| FGF7 | Fibroblast growth factor 7 |
| SERPINA1 | Alpha-1-antitrypsin |
| HNF4A | Hepatocyte nuclear factor 4-alpha |
| ELAVL1 | ELAV-like protein 1 |
| MIR148B | MicroRNA 148b |
| ACE | Angiotensin-converting enzyme |
| NAT1 | Arylamine N-acetyltransferase 1 |
| GUCY2C | Heat-stable enterotoxin receptor |
| SNHG16 | Small Nucleolar RNA Host Gene 16 |
| KDM4C | Lysine-specific demethylase 4C |
| MIR378A | MicroRNA 378a |
| SOCS1 | Suppressor of cytokine signaling 1 |
| MUC2 | Mucin-2 |
| LRP5 | Low-density lipoprotein receptor-related protein 5 |
| IL7 | Interleukin-7 |
| BDNF | Brain-derived neurotrophic factor |
| CD80 | T-lymphocyte activation antigen CD80 |
| TGM2 | Protein-glutamine gamma-glutamyltransferase 2 |
| RUNX2 | Runt-related transcription factor 2 |
| MIR324 | MicroRNA 324 |
| DLC1 | Rho GTPase-activating protein 7 |
| ANGPT2 | Angiopoietin-2 |
| RARA | Retinoic acid receptor alpha |
| CD46 | Membrane cofactor protein |
| MME | Neprilysin |
| MIR132 | MicroRNA 132 |
| MIR198 | MicroRNA 198 |
| FOLH1 | Glutamate carboxypeptidase 2 |
| MAX | Protein max |
| DHFR | Dihydrofolate reductase |
| DVL1 | Segment polarity protein dishevelled homolog DVL-1 |
| AMACR | Alpha-methylacyl-CoA racemase |
| MT-CYB | Cytochrome b |
| NRG1 | Pro-neuregulin-1, membrane-bound isoform |
| ETV4 | ETS translocation variant 4 |
| NTS | Neurotensin/neuromedin N |
| PAX3 | Paired box protein Pax-3 |
| DDIT3 | DNA damage-inducible transcript 3 protein |
| WNT3A | Protein Wnt-3a |
| IRF1 | Interferon regulatory factor 1 |
| LIG4 | DNA ligase 4 |
| AFP | Alpha-fetoprotein |
| SELE | E-selectin |
| STMN1 | Stathmin |
| YBX1 | Y-box-binding protein 1 |
| ITGB4 | Integrin beta-4 |
| POSTN | Periostin |
| MIF | Macrophage migration inhibitory factor |
| PANDAR | Promoter Of CDKN1A Antisense DNA Damage Activated RNA |
| CEACAM7 | Carcinoembryonic antigen-related cell adhesion molecule 7 |
| MACC1 | Metastasis-associated in colon cancer protein 1 |
| CD40LG | CD40 ligand |
| NPM1 | Nucleophosmin |
| CD4 | T-cell surface glycoprotein CD4 |
| COX5A | Cytochrome c oxidase subunit 5A, mitochondrial |
| SFTPB | Pulmonary surfactant-associated protein B |
| CASP2 | Caspase-2 |
| CYTOR | Cytoskeleton Regulator RNA |
| SFRP1 | Secreted frizzled-related protein 1 |
| ALOX12 | Polyunsaturated fatty acid lipoxygenase ALOX12 |
| P2RX7 | P2X purinoceptor 7 |
| PSG2 | Pregnancy-specific beta-1-glycoprotein 2 |
| HBEGF | Proheparin-binding EGF-like growth factor |
| CYP2A6 | Cytochrome P450 2A6 |
| AXL | Tyrosine-protein kinase receptor UFO |
| TP53COR1 | Tumor Protein P53 Pathway Corepressor 1 |
| PTGER4 | Prostaglandin E2 receptor EP4 subtype |
| EREG | Proepiregulin |
| MIR30D | MicroRNA 30d |
| PDPK1 | 3-phosphoinositide-dependent protein kinase 1 |
| MIR532 | MicroRNA 532 |
| NCOA3 | Nuclear receptor coactivator 3 |
| BCL10 | B-cell lymphoma/leukemia 10 |
| CDKN1C | Cyclin-dependent kinase inhibitor 1C |
| MAPK9 | Mitogen-activated protein kinase 9 |
| CYP2D6 | Cytochrome P450 2D6 |
| MTDH | Protein LYRIC |
| ABCA1 | Phospholipid-transporting ATPase ABCA1 |
| VCAM1 | Vascular cell adhesion protein 1 |
| SUFU | Suppressor of fused homolog |
| TCF4 | Transcription factor 4 |
| HNF1A-AS1 | HNF1A Antisense RNA 1 |
| MIR103A1 | MicroRNA 103a-1 |
| CLDN7 | Claudin-7 |
| TYR | Tyrosinase |
| HOXB13 | Homeobox protein Hox-B13 |
| ELN | Elastin |
| PRTN3 | Myeloblastin |
| CEACAM1 | Carcinoembryonic antigen-related cell adhesion molecule 1 |
| EIF4EBP1 | Eukaryotic translation initiation factor 4E-binding protein 1 |
| RNF43 | E3 ubiquitin-protein ligase RNF43 |
| MSLN | Mesothelin |
| MIR345 | MicroRNA 345 |
| CALCA | Calcitonin gene-related peptide 1 |
| FADD | FAS-associated death domain protein |
| IKBKB | Inhibitor of nuclear factor kappa-B kinase subunit beta |
| S100B | Protein S100-B |
| CTAG1B | Cancer/Testis Antigen 1B |
| XPA | DNA repair protein complementing XP-A cells |
| EWSR1 | RNA-binding protein EWS |
| GHET1 | Gastric Carcinoma Proliferation Enhancing Transcript 1 |
| CRNDE | Colorectal Neoplasia Differentially Expressed |
| XRCC5 | X-ray repair cross-complementing protein 5 |
| CLU | Clusterin |
| ATF3 | Cyclic AMP-dependent transcription factor ATF-3 |
| EDNRB | Endothelin receptor type B |
| MIR423 | MicroRNA 423 |
| PHB | Prohibitin |
| ANPEP | Aminopeptidase N |
| GUSB | Beta-glucuronidase |
| IGFBP7 | Insulin-like growth factor-binding protein 7 |
| XPC | DNA repair protein complementing XP-C cells |
| EPHB4 | Ephrin type-B receptor 4 |
| ENO1 | Alpha-enolase |
| SYNE1 | Nesprin-1 |
| MIR101-1 | MicroRNA 101-1 |
| MIR338 | MicroRNA 338 |
| BIRC2 | Baculoviral IAP repeat-containing protein 2 |
| FZD4 | Frizzled-4 |
| WIF1 | Wnt inhibitory factor 1 |
| CDCP1 | CUB domain-containing protein 1 |
| HULC | Highly upregulated in liver cancer |
| ERCC5 | DNA repair protein complementing XP-G cells |
| HNF1B | Hepatocyte nuclear factor 1-beta |
| MSR1 | Macrophage scavenger receptor types I and II |
| APOB | Apolipoprotein B-100 |
| CYP1A2 | Cytochrome P450 1A2 |
| GALNT12 | Polypeptide N-acetylgalactosaminyltransferase 12 |
| KISS1 | Metastasis-suppressor KiSS-1 |
| CYP24A1 | 1,25-dihydroxyvitamin D |
| VIP | VIP peptides |
| PRKAR1A | cAMP-dependent protein kinase type I-alpha regulatory subunit |
| CALB2 | Calretinin |
| NANOG | Homeobox protein NANOG |
| SLC22A18 | Solute carrier family 22 member 18 |
| MIR135A1 | MicroRNA 135a-1 |
| MST1R | Macrophage-stimulating protein receptor |
| BNIP3 | BCL2/adenovirus E1B 19 kDa protein-interacting protein 3 |
| ITGA5 | Integrin alpha-5 |
| APAF1 | Apoptotic protease-activating factor 1 |
| GNRH1 | Progonadoliberin-1 |
| ALDH1A1 | Retinal dehydrogenase 1 |
| CHUK | Inhibitor of nuclear factor kappa-B kinase subunit alpha |
| SOCS3 | Suppressor of cytokine signaling 3 |
| ERG | Transcriptional regulator ERG |
| CYP3A5 | Cytochrome P450 3A5 |
| CD8A | T-cell surface glycoprotein CD8 alpha chain |
| WWOX | WW domain-containing oxidoreductase |
| ERCC4 | DNA repair endonuclease XPF |
| ITGAV | Integrin alpha-V |
| NGF | Beta-nerve growth factor |
| FLNA | Filamin-A |
| MIRLET7F1 | MicroRNA Let-7f-1 |
| CCR7 | C-C chemokine receptor type 7 |
| GSN | Gelsolin |
| LNCRNA-ATB | Long Noncoding RNA Activated By TGF-Beta |
| MTAP | S-methyl-5'-thioadenosine phosphorylase |
| CXCR3 | C-X-C chemokine receptor type 3 |
| RRM1 | Ribonucleoside-diphosphate reductase large subunit |
| MIR328 | MicroRNA 328 |
| MIR152 | MicroRNA 152 |
| DMBT1 | Deleted in malignant brain tumors 1 protein |
| ITGB3 | Integrin beta-3 |
| FGF8 | Fibroblast growth factor 8 |
| SDHC | Succinate dehydrogenase cytochrome b560 subunit, mitochondrial |
| MT-CO2 | Cytochrome c oxidase subunit 2 |
| CD34 | Hematopoietic progenitor cell antigen CD34 |
| MIR122 | MicroRNA 122 |
| EML4 | Echinoderm microtubule-associated protein-like 4 |
| MIR181B1 | MicroRNA 181b-1 |
| TFE3 | Transcription factor E3 |
| EPAS1 | Endothelial PAS domain-containing protein 1 |
| MIR92A1 | MicroRNA 92a-1 |
| MAP2K7 | Dual specificity mitogen-activated protein kinase kinase 7 |
| SHC1 | SHC-transforming protein 1 |
| IL1A | Interleukin-1 alpha |
| HMGA1 | High mobility group protein HMG-I/HMG-Y |
| TUBB | Tubulin beta chain |
| ABL1 | Tyrosine-protein kinase ABL1 |
| HPSE | Heparanase |
| PRMT1 | Protein arginine N-methyltransferase 1 |
| BIRC7 | Baculoviral IAP repeat-containing protein 7 |
| SDHD | Succinate dehydrogenase [ubiquinone] cytochrome b small subunit, mitochondrial |
| MIR181C | MicroRNA 181c |
| WNT2B | Protein Wnt-2b |
| LINC00261 | Long Intergenic Non-Protein Coding RNA 261 |
| FAT4 | Protocadherin Fat 4 |
| NOTCH3 | Neurogenic locus notch homolog protein 3 |
| SSTR2 | Somatostatin receptor type 2 |
| LCN2 | Neutrophil gelatinase-associated lipocalin |
| ETV6 | Transcription factor ETV6 |
| IDH2 | Isocitrate dehydrogenase [NADP], mitochondrial |
| SFTPD | Pulmonary surfactant-associated protein D |
| INSR | Insulin receptor |
| PTK2B | Protein-tyrosine kinase 2-beta |
| APOE | Apolipoprotein E |
| DLK1 | Protein delta homolog 1 |
| MYCL | Protein L-Myc |
| TBX1 | T-box transcription factor TBX1 |
| SERPINB2 | Plasminogen activator inhibitor 2 |
| MVP | Major vault protein |
| CCL3 | C-C motif chemokine 3 |
| ATR | Serine/threonine-protein kinase ATR |
| F3 | Tissue factor |
| RNY1 | RNA, Ro60-Associated Y1 |
| RNY3 | RNA, Ro60-Associated Y3 |
| KAT5 | Histone acetyltransferase KAT5 |
| RECQL4 | ATP-dependent DNA helicase Q4 |
| IGFBP1 | Insulin-like growth factor-binding protein 1 |
| LGALS3BP | Galectin-3-binding protein |
| DDB2 | DNA damage-binding protein 2 |
| MIR125B1 | MicroRNA 125b-1 |
| KRT5 | Keratin, type II cytoskeletal 5 |
| SLC6A4 | Sodium-dependent serotonin transporter |
| TTR | Transthyretin |
| FZD6 | Frizzled-6 |
| LMNA | Prelamin-A/C |
| MYLK | Myosin light chain kinase, smooth muscle |
| BID | BH3-interacting domain death agonist |
| MIR330 | MicroRNA 330 |
| TFF1 | Trefoil factor 1 |
| RALA | Ras-related protein Ral-A |
| FGF1 | Fibroblast growth factor 1 |
| ABCC3 | Canalicular multispecific organic anion transporter 2 |
| CDH3 | Cadherin-3 |
| LRP6 | Low-density lipoprotein receptor-related protein 6 |
| GZMB | Granzyme B |
| RARS1 | Arginine--tRNA ligase, cytoplasmic |
| IKBKG | NF-kappa-B essential modulator |
| TH | Tyrosine 3-monooxygenase |
| IL18 | Interleukin-18 |
| HOXA11-AS | HOXA11 Antisense RNA |
| TMEFF2 | Tomoregulin-2 |
| LIMK1 | LIM domain kinase 1 |
| CSF1R | Macrophage colony-stimulating factor 1 receptor |
| NDRG1 | Protein NDRG1 |
| CLDN4 | Claudin-4 |
| SHBG | Sex hormone-binding globulin |
| PRLR | Prolactin receptor |
| TAP1 | Antigen peptide transporter 1 |
| FOSL1 | Fos-related antigen 1 |
| TNS4 | Tensin-4 |
| ACTA2 | Actin, aortic smooth muscle |
| ADRB2 | Beta-2 adrenergic receptor |
| BANCR | BRAF-Activated Non-Protein Coding RNA |
| ATF1 | Cyclic AMP-dependent transcription factor ATF-1 |
| PEBP1 | Phosphatidylethanolamine-binding protein 1 |
| TNFRSF11B | Tumor necrosis factor receptor superfamily member 11B |
| HAVCR2 | Hepatitis A virus cellular receptor 2 |
| S100A6 | Protein S100-A6 |
| PTENP1 | Phosphatase And Tensin Homolog Pseudogene 1 |
| GGT1 | Glutathione hydrolase 1 proenzyme |
| FOXA1 | Hepatocyte nuclear factor 3-alpha |
| JAG1 | Protein jagged-1 |
| IL24 | Interleukin-24 |
| WNT2 | Protein Wnt-2 |
| PDGFD | Platelet-derived growth factor D |
| WRAP53 | Telomerase Cajal body protein 1 |
| GATA2 | Endothelial transcription factor GATA-2 |
| DUSP1 | Dual specificity protein phosphatase 1 |
| H2AX | Histone H2AX |
| S100A2 | Protein S100-A2 |
| TRPS1 | Zinc finger transcription factor Trps1 |
| CDC6 | Cell division control protein 6 homolog |
| STAT6 | Signal transducer and activator of transcription 6 |
| ACVRL1 | Serine/threonine-protein kinase receptor R3 |
| PDGFRL | Platelet-derived growth factor receptor-like protein |
| SOX2-OT | SOX2 Overlapping Transcript |
| MTR | Methionine synthase |
| CCNE2 | G1/S-specific cyclin-E2 |
| CLDN1 | Claudin-1 |
| MIR151A | MicroRNA 151a |
| NR3C1 | Glucocorticoid receptor |
| COL1A1 | Collagen alpha-1 |
| MIR28 | MicroRNA 28 |
| CCNG1 | Cyclin-G1 |
| HFE | Hereditary hemochromatosis protein |
| GATA3 | Trans-acting T-cell-specific transcription factor GATA-3 |
| REST | RE1-silencing transcription factor |
| PTPRC | Receptor-type tyrosine-protein phosphatase C |
| FLT3 | Receptor-type tyrosine-protein kinase FLT3 |
| GDNF | Glial cell line-derived neurotrophic factor |
| FZD7 | Frizzled-7 |
| KDM1A | Lysine-specific histone demethylase 1A |
| UCHL1 | Ubiquitin carboxyl-terminal hydrolase isozyme L1 |
| HSPA8 | Heat shock cognate 71 kDa protein |
| SULT1A1 | Sulfotransferase 1A1 |
| SOX4 | Transcription factor SOX-4 |
| BRMS1 | Breast cancer metastasis-suppressor 1 |
| PGF | Placenta growth factor |
| F2R | Proteinase-activated receptor 1 |
| ZFHX3 | Zinc finger homeobox protein 3 |
| LAMB3 | Laminin subunit beta-3 |
| MIR542 | MicroRNA 542 |
| MIR429 | MicroRNA 429 |
| PTGER2 | Prostaglandin E2 receptor EP2 subtype |
| FUS | RNA-binding protein FUS |
| PAX8 | Paired box protein Pax-8 |
| MIR130B | MicroRNA 130b |
| NOTCH2 | Neurogenic locus notch homolog protein 2 |
| CHI3L1 | Chitinase-3-like protein 1 |
| TUSC3 | Tumor suppressor candidate 3 |
| MITF | Microphthalmia-associated transcription factor |
| MIR625 | MicroRNA 625 |
| SCGB1A1 | Uteroglobin |
| GHR | Growth hormone receptor |
| CD9 | CD9 antigen |
| CCN4 | CCN family member 4 |
| JAK3 | Tyrosine-protein kinase JAK3 |
| SNHG5 | Small Nucleolar RNA Host Gene 5 |
| HLA-DQB1 | HLA class II histocompatibility antigen, DQ beta 1 chain |
| ROCK1 | Rho-associated protein kinase 1 |
| NAMPT | Nicotinamide phosphoribosyltransferase |
| FANCC | Fanconi anemia group C protein |
| SOS2 | Son of sevenless homolog 2 |
| BCAR4 | Breast Cancer Anti-Estrogen Resistance 4 |
| MBL2 | Mannose-binding protein C |
| BLACAT1 | Bladder cancer-associated transcript 1, noncoding |
| ENPP2 | Ectonucleotide pyrophosphatase/phosphodiesterase family member 2 |
| AGR2 | Anterior gradient protein 2 homolog |
| UHRF1 | E3 ubiquitin-protein ligase UHRF1 |
| ADA | Adenosine deaminase |
| MIR499A | MicroRNA 499a |
| MIRLET7E | MicroRNA Let-7e |
| PTPRG | Receptor-type tyrosine-protein phosphatase gamma |
| PTPA | Serine/threonine-protein phosphatase 2A activator |
| TLR7 | Toll-like receptor 7 |
| HLA-C | HLA class I histocompatibility antigen, C alpha chain |
| CISH | Cytokine-inducible SH2-containing protein |
| MIR186 | MicroRNA 186 |
| PTH | Parathyroid hormone |
| NR1H2 | Oxysterols receptor LXR-beta |
| RHO | Rhodopsin |
| SNHG12 | Putative uncharacterized protein SNHG12 |
| TRIP13 | Pachytene checkpoint protein 2 homolog |
| LASP1 | LIM and SH3 domain protein 1 |
| MXRA5 | Matrix-remodeling-associated protein 5 |
| FANCG | Fanconi anemia group G protein |
| NAPSA | Napsin-A |
| MAP3K8 | Mitogen-activated protein kinase kinase kinase 8 |
| NPTN-IT1 | NPTN Intronic Transcript 1 |
| ST14 | Suppressor of tumorigenicity 14 protein |
| TET2 | Methylcytosine dioxygenase TET2 |
| IL3 | Interleukin-3 |
| STK4 | Serine/threonine-protein kinase 4 |
| TINCR | TINCR Ubiquitin Domain Containing |
| TCF7L1 | Transcription factor 7-like 1 |
| MIR98 | MicroRNA 98 |
| ANGPT1 | Angiopoietin-1 |
| STAT5A | Signal transducer and activator of transcription 5A |
| SERPINC1 | Antithrombin-III |
| IL15 | Interleukin-15 |
| ETV1 | ETS translocation variant 1 |
| MAGEA1 | Melanoma-associated antigen 1 |
| CXCL10 | C-X-C motif chemokine 10 |
| AGER | Advanced glycosylation end product-specific receptor |
| DCK | Deoxycytidine kinase |
| CCL11 | Eotaxin |
| KRT14 | Keratin, type I cytoskeletal 14 |
| MIR339 | MicroRNA 339 |
| MIR206 | MicroRNA 206 |
| NES | Nestin |
| PTPN13 | Tyrosine-protein phosphatase non-receptor type 13 |
| INHA | Inhibin alpha chain |
| TUSC7 | Tumor Suppressor Candidate 7 |
| RPSA | 40S ribosomal protein SA |
| PSAT1 | Phosphoserine aminotransferase |
| TFPI2 | Tissue factor pathway inhibitor 2 |
| TFF3 | Trefoil factor 3 |
| MIR135B | MicroRNA 135b |
| TLR1 | Toll-like receptor 1 |
| ID1 | DNA-binding protein inhibitor ID-1 |
| PLAT | Tissue-type plasminogen activator |
| WNT11 | Protein Wnt-11 |
| CYP2C9 | Cytochrome P450 2C9 |
| LYVE1 | Lymphatic vessel endothelial hyaluronic acid receptor 1 |
| MIR130A | MicroRNA 130a |
| EXO1 | Exonuclease 1 |
| CSK | Tyrosine-protein kinase CSK |
| GATA4 | Transcription factor GATA-4 |
| IL5 | Interleukin-5 |
| LEPR | Leptin receptor |
| MIR574 | MicroRNA 574 |
| STS | Steryl-sulfatase |
| MAGEA3 | Melanoma-associated antigen 3 |
| PRNCR1 | Prostate cancer-associated noncoding RNA 1 |
| MSMB | Beta-microseminoprotein |
| CASC8 | Cancer susceptibility candidate 8, noncoding |
| CBR3-AS1 | CBR3 Antisense RNA 1 |
| CXCR1 | C-X-C chemokine receptor type 1 |
| LIFR | Leukemia inhibitory factor receptor |
| SLPI | Antileukoproteinase |
| AKR1C3 | Aldo-keto reductase family 1 member C3 |
| TTN | Titin |
| MIR33A | MicroRNA 33a |
| ELAC2 | Zinc phosphodiesterase ELAC protein 2 |
| TF | Serotransferrin |
| CHKA | Choline kinase alpha |
| MIR503 | MicroRNA 503 |
| BLM | Bloom syndrome protein |
| YWHAE | 14-3-3 protein epsilon |
| MIR197 | MicroRNA 197 |
| TJP1 | Tight junction protein ZO-1 |
| CD28 | T-cell-specific surface glycoprotein CD28 |
| ALOX15 | Arachidonate 15-lipoxygenase |
| FKBP5 | Peptidyl-prolyl cis-trans isomerase FKBP5 |
| EPB41L3 | Band 4.1-like protein 3 |
| MLANA | Melanoma antigen recognized by T-cells 1 |
| BCYRN1 | Brain Cytoplasmic RNA 1 |
| DUXAP9 | Double Homeobox A Pseudogene 9 |
| LINC01133 | Long Intergenic Non-Protein Coding RNA 1133 |
| CTSL | Cathepsin L1 |
| SRD5A2 | 3-oxo-5-alpha-steroid 4-dehydrogenase 2 |
| PRKCI | Protein kinase C iota type |
| ECM1 | Extracellular matrix protein 1 |
| HOPX | Homeodomain-only protein |
| PPARD | Peroxisome proliferator-activated receptor delta |
| CASP10 | Caspase-10 |
| PRKCD | Protein kinase C delta type |
| PSCA | Prostate stem cell antigen |
| PRDM10 | PR domain zinc finger protein 10 |
| NTN1 | Netrin-1 |
| LTA | Lymphotoxin-alpha |
| SEMA3B | Semaphorin-3B |
| TIMELESS | Protein timeless homolog |
| F5 | Coagulation factor V |
| ASS1 | Argininosuccinate synthase |
| MYD88 | Myeloid differentiation primary response protein MyD88 |
| BAG1 | BAG family molecular chaperone regulator 1 |
| PTP4A3 | Protein tyrosine phosphatase type IVA 3 |
| SNHG20 | Small Nucleolar RNA Host Gene 20 |
| BMP7 | Bone morphogenetic protein 7 |
| LRP1 | Prolow-density lipoprotein receptor-related protein 1 |
| ALCAM | CD166 antigen |
| HOXB9 | Homeobox protein Hox-B9 |
| TDGF1 | Teratocarcinoma-derived growth factor 1 |
| CD79A | B-cell antigen receptor complex-associated protein alpha chain |
| PTTG1 | Securin |
| UMPS | Uridine 5'-monophosphate synthase |
| CTTN | Src substrate cortactin |
| WNT4 | Protein Wnt-4 |
| GATA6 | Transcription factor GATA-6 |
| EPHB6 | Ephrin type-B receptor 6 |
| FEZF1-AS1 | FEZF1 Antisense RNA 1 |
| POMC | Pro-opiomelanocortin |
| CBL | E3 ubiquitin-protein ligase CBL |
| PLCG1 | 1-phosphatidylinositol 4,5-bisphosphate phosphodiesterase gamma-1 |
| AURKB | Aurora kinase B |
| NORAD | Non-Coding RNA Activated By DNA Damage |
| RXRA | Retinoic acid receptor RXR-alpha |
| CLCA2 | Calcium-activated chloride channel regulator 2 |
| PPM1D | Protein phosphatase 1D |
| MIRLET7A3 | MicroRNA Let-7a-3 |
| MUC3A | Mucin-3A |
| MAP2K5 | Dual specificity mitogen-activated protein kinase kinase 5 |
| ALDH2 | Aldehyde dehydrogenase, mitochondrial |
| TPX2 | Targeting protein for Xklp2 |
| CD164 | Sialomucin core protein 24 |
| HP | Haptoglobin |
| TMPRSS2 | Transmembrane protease serine 2 |
| FUT4 | Alpha-(1,3)-fucosyltransferase 4 |
| AIMP2 | Aminoacyl tRNA synthase complex-interacting multifunctional protein 2 |
| FLI1 | Friend leukemia integration 1 transcription factor |
| LINC00460 | Long Intergenic Non-Protein Coding RNA 460 |
| MXI1 | Max-interacting protein 1 |
| KLF5 | Krueppel-like factor 5 |
| MIR376A1 | MicroRNA 376a-1 |
| MIR212 | MicroRNA 212 |
| TLR6 | Toll-like receptor 6 |
| ATRX | Transcriptional regulator ATRX |
| PTPRT | Receptor-type tyrosine-protein phosphatase T |
| PRKDC | DNA-dependent protein kinase catalytic subunit |
| HSP90B1 | Endoplasmin |
| CDH5 | Cadherin-5 |
| SPHK1 | Sphingosine kinase 1 |
| TNC | Tenascin |
| IL1R1 | Interleukin-1 receptor type 1 |
| AGTR1 | Type-1 angiotensin II receptor |
| GPER1 | G-protein coupled estrogen receptor 1 |
| LINC00673 | Long Intergenic Non-Protein Coding RNA 673 |
| MIR129-1 | MicroRNA 129-1 |
| BRD4 | Bromodomain-containing protein 4 |
| VEGFB | Vascular endothelial growth factor B |
| ROBO1 | Roundabout homolog 1 |
| CDC73 | Parafibromin |
| CXCL9 | C-X-C motif chemokine 9 |
| DNAJC6 | Putative tyrosine-protein phosphatase auxilin |
| SQSTM1 | Sequestosome-1 |
| IGF2BP3 | Insulin-like growth factor 2 mRNA-binding protein 3 |
| EBAG9 | Receptor-binding cancer antigen expressed on SiSo cells |
| IL2RB | Interleukin-2 receptor subunit beta |
| CXCL5 | C-X-C motif chemokine 5 |
| ITGAM | Integrin alpha-M |
| SNHG15 | Small Nucleolar RNA Host Gene 15 |
| PAK1 | Serine/threonine-protein kinase PAK 1 |
| MIR455 | MicroRNA 455 |
| TGM1 | Protein-glutamine gamma-glutamyltransferase K |
| MAPT | Microtubule-associated protein tau |
| MIR449A | MicroRNA 449a |
| MORC2 | ATPase MORC2 |
| TLR10 | Toll-like receptor 10 |
| F2RL1 | Proteinase-activated receptor 2 |
| MIR30C1 | MicroRNA 30c-1 |
| FAM83H-AS1 | FAM83H Antisense RNA 1 (Head To Head) |
| MCAM | Cell surface glycoprotein MUC18 |
| TPBG | Trophoblast glycoprotein |
| MUC7 | Mucin-7 |
| DKC1 | H/ACA ribonucleoprotein complex subunit DKC1 |
| HAGLR | HOXD Antisense Growth-Associated Long Non-Coding RNA |
| TRAF6 | TNF receptor-associated factor 6 |
| S100A9 | Protein S100-A9 |
| MIR202 | MicroRNA 202 |
| EPOR | Erythropoietin receptor |
| IDO1 | Indoleamine 2,3-dioxygenase 1 |
| PRKCZ | Protein kinase C zeta type |
| MYOD1 | Myoblast determination protein 1 |
| CCR5 | C-C chemokine receptor type 5 |
| KLLN | Killin |
| MIR377 | MicroRNA 377 |
| FANCD2 | Fanconi anemia group D2 protein |
| PRKCE | Protein kinase C epsilon type |
| CD86 | T-lymphocyte activation antigen CD86 |
| NGFR | Tumor necrosis factor receptor superfamily member 16 |
| SNCA | Alpha-synuclein |
| VTN | Vitronectin |
| MAGEA4 | Melanoma-associated antigen 4 |
| EPO | Erythropoietin |
| FGF10 | Fibroblast growth factor 10 |
| CLPTM1L | Cleft lip and palate transmembrane protein 1-like protein |
| E2F4 | Transcription factor E2F4 |
| HSPA1A | Heat shock 70 kDa protein 1A |
| FCGR3A | Low affinity immunoglobulin gamma Fc region receptor III-A |
| SMO | Smoothened homolog |
| RMRP | RNA Component Of Mitochondrial RNA Processing Endoribonuclease |
| HSPD1 | 60 kDa heat shock protein, mitochondrial |
| SSX2 | Protein SSX2 |
| TLR8 | Toll-like receptor 8 |
| LGR5 | Leucine-rich repeat-containing G-protein coupled receptor 5 |
| MCM2 | DNA replication licensing factor MCM2 |
| NCOR1 | Nuclear receptor corepressor 1 |
| PRKACA | cAMP-dependent protein kinase catalytic subunit alpha |
| ETV5 | ETS translocation variant 5 |
| FBN1 | Fibrillin-1 |
| FENDRR | FOXF1 Adjacent Non-Coding Developmental Regulatory RNA |
| IL11 | Interleukin-11 |
| MIR196B | MicroRNA 196b |
| SPDEF | SAM pointed domain-containing Ets transcription factor |
| ECT2 | Protein ECT2 |
| ATP7A | Copper-transporting ATPase 1 |
| AKR1C1 | Aldo-keto reductase family 1 member C1 |
| MIR95 | MicroRNA 95 |
| TGIF1 | Homeobox protein TGIF1 |
| CTSC | Dipeptidyl peptidase 1 |
| MIR138-1 | MicroRNA 138-1 |
| PSMA7 | Proteasome subunit alpha type-7 |
| GJB2 | Gap junction beta-2 protein |
| IL12B | Interleukin-12 subunit beta |
| AGT | Angiotensinogen |
| BBC3 | Bcl-2-binding component 3, isoforms 1/2 |
| MIR26B | MicroRNA 26b |
| PRDX1 | Peroxiredoxin-1 |
| MIR196A2 | MicroRNA 196a-2 |
| LIF | Leukemia inhibitory factor |
| TNFRSF11A | Tumor necrosis factor receptor superfamily member 11A |
| HLA-DQA1 | HLA class II histocompatibility antigen, DQ alpha 1 chain |
| TSG101 | Tumor susceptibility gene 101 protein |
| THPO | Thrombopoietin |
| CDC25A | M-phase inducer phosphatase 1 |
| ATG5 | Autophagy protein 5 |
| PML | Protein PML |
| CCK | Cholecystokinin |
| MMP10 | Stromelysin-2 |
| LDHA | L-lactate dehydrogenase A chain |
| PRL | Prolactin |
| KLK10 | Kallikrein-10 |
| PDGFA | Platelet-derived growth factor subunit A |
| HLTF | Helicase-like transcription factor |
| ACTN4 | Alpha-actinin-4 |
| DKK3 | Dickkopf-related protein 3 |
| CYP27B1 | 25-hydroxyvitamin D-1 alpha hydroxylase, mitochondrial |
| CD55 | Complement decay-accelerating factor |
| PMAIP1 | Phorbol-12-myristate-13-acetate-induced protein 1 |
| ACP3 | Prostatic acid phosphatase |
| NTHL1 | Endonuclease III-like protein 1 |
| GRN | Progranulin |
| CLCA1 | Calcium-activated chloride channel regulator 1 |
| PON1 | Serum paraoxonase/arylesterase 1 |
| LOXL2 | Lysyl oxidase homolog 2 |
| WNT7B | Protein Wnt-7b |
| SERPINH1 | Serpin H1 |
| KLK4 | Kallikrein-4 |
| MAPK10 | Mitogen-activated protein kinase 10 |
| LCK | Tyrosine-protein kinase Lck |
| DPP4 | Dipeptidyl peptidase 4 |
| HNRNPA2B1 | Heterogeneous nuclear ribonucleoproteins A2/B1 |
| CLDN3 | Claudin-3 |
| ALPP | Alkaline phosphatase, placental type |
| CTBP1 | C-terminal-binding protein 1 |
| PALLD | Palladin |
| SERPINB3 | Serpin B3 |
| FOXF1 | Forkhead box protein F1 |
| S100A8 | Protein S100-A8 |
| LAMA5 | Laminin subunit alpha-5 |
| CD59 | CD59 glycoprotein |
| TFRC | Transferrin receptor protein 1 |
| G6PD | Glucose-6-phosphate 1-dehydrogenase |
| PIM1 | Serine/threonine-protein kinase pim-1 |
| MELK | Maternal embryonic leucine zipper kinase |
| SOX10 | Transcription factor SOX-10 |
| SELENBP1 | Methanethiol oxidase |
| THBD | Thrombomodulin |
| IRS2 | Insulin receptor substrate 2 |
| CSNK2A1 | Casein kinase II subunit alpha |
| MB | Myoglobin |
| ALPL | Alkaline phosphatase, tissue-nonspecific isozyme |
| GPX2 | Glutathione peroxidase 2 |
| RHOB | Rho-related GTP-binding protein RhoB |
| CCR3 | C-C chemokine receptor type 3 |
| MIR370 | MicroRNA 370 |
| MYH9 | Myosin-9 |
| MMP8 | Neutrophil collagenase |
| APRT | Adenine phosphoribosyltransferase |
| LGALS4 | Galectin-4 |
| ADAMTS13 | A disintegrin and metalloproteinase with thrombospondin motifs 13 |
| ING1 | Inhibitor of growth protein 1 |
| TNFRSF6B | Tumor necrosis factor receptor superfamily member 6B |
| RACK1 | Receptor of activated protein C kinase 1 |
| NOG | Noggin |
| NCOA1 | Nuclear receptor coactivator 1 |
| HSD17B1 | Estradiol 17-beta-dehydrogenase 1 |
| EIF4G1 | Eukaryotic translation initiation factor 4 gamma 1 |
| AKR1B10 | Aldo-keto reductase family 1 member B10 |
| CIP2A | Protein CIP2A |
| GHRL | Appetite-regulating hormone |
| NCOR2 | Nuclear receptor corepressor 2 |
| PTPRJ | Receptor-type tyrosine-protein phosphatase eta |
| PTPRU | Receptor-type tyrosine-protein phosphatase U |
| TRPV6 | Transient receptor potential cation channel subfamily V member 6 |
| KLK6 | Kallikrein-6 |
| NPRL2 | GATOR complex protein NPRL2 |
| PRKCB | Protein kinase C beta type |
| WWTR1 | WW domain-containing transcription regulator protein 1 |
| HMMR | Hyaluronan mediated motility receptor |
| MIR124-3 | MicroRNA 124-3 |
| APP | Amyloid-beta precursor protein |
| RHOC | Rho-related GTP-binding protein RhoC |
| USP7 | Ubiquitin carboxyl-terminal hydrolase 7 |
| LAMC1 | Laminin subunit gamma-1 |
| HDGF | Hepatoma-derived growth factor |
| ADORA3 | Adenosine receptor A3 |
| IFNGR1 | Interferon gamma receptor 1 |
| DAB2IP | Disabled homolog 2-interacting protein |
| GPRC5A | Retinoic acid-induced protein 3 |
| COL3A1 | Collagen alpha-1 |
| ZEB1-AS1 | ZEB1 Antisense RNA 1 |
| KRT1 | Keratin, type II cytoskeletal 1 |
| AGO2 | Protein argonaute-2 |
| DUSP6 | Dual specificity protein phosphatase 6 |
| DSP | Desmoplakin |
| DDR1 | Epithelial discoidin domain-containing receptor 1 |
| TK1 | Thymidine kinase, cytosolic |
| L1CAM | Neural cell adhesion molecule L1 |
| CEBPB | CCAAT/enhancer-binding protein beta |
| TRAF3 | TNF receptor-associated factor 3 |
| SLC19A1 | Reduced folate transporter |
| FOXC1 | Forkhead box protein C1 |
| HDAC4 | Histone deacetylase 4 |
| IGFBP5 | Insulin-like growth factor-binding protein 5 |
| FCGR2A | Low affinity immunoglobulin gamma Fc region receptor II-a |
| IL32 | Interleukin-32 |
| MIR133A1 | MicroRNA 133a-1 |
| PTGES | Prostaglandin E synthase |
| GPT | Alanine aminotransferase 1 |
| EHBP1 | EH domain-binding protein 1 |
| HSP90AB1 | Heat shock protein HSP 90-beta |
| IL13RA2 | Interleukin-13 receptor subunit alpha-2 |
| MIR675 | MicroRNA 675 |
| HDC | Histidine decarboxylase |
| TNFRSF9 | Tumor necrosis factor receptor superfamily member 9 |
| BCAR1 | Breast cancer anti-estrogen resistance protein 1 |
| STUB1 | E3 ubiquitin-protein ligase CHIP |
| FOXE1 | Forkhead box protein E1 |
| S100P | Protein S100-P |
| SFRP2 | Secreted frizzled-related protein 2 |
| AKR1C2 | Aldo-keto reductase family 1 member C2 |
| ADAM12 | Disintegrin and metalloproteinase domain-containing protein 12 |
| UROD | Uroporphyrinogen decarboxylase |
| ARG1 | Arginase-1 |
| COLCA2 | Colorectal cancer-associated protein 2 |
| MDK | Midkine |
| FURIN | Furin |
| AIFM1 | Apoptosis-inducing factor 1, mitochondrial |
| SATB1 | DNA-binding protein SATB1 |
| MIR218-1 | MicroRNA 218-1 |
| PRKAA1 | 5'-AMP-activated protein kinase catalytic subunit alpha-1 |
| AMFR | E3 ubiquitin-protein ligase AMFR |
| ADAM9 | Disintegrin and metalloproteinase domain-containing protein 9 |
| HNF1A | Hepatocyte nuclear factor 1-alpha |
| TCIM | Transcriptional and immune response regulator |
| RAD54B | DNA repair and recombination protein RAD54B |
| PUF60 | Poly |
| CXCL2 | C-X-C motif chemokine 2 |
| CCL18 | C-C motif chemokine 18 |
| HTRA1 | Serine protease HTRA1 |
| TRAF2 | TNF receptor-associated factor 2 |
| COL11A1 | Collagen alpha-1 |
| HSPG2 | Basement membrane-specific heparan sulfate proteoglycan core protein |
| SETBP1 | SET-binding protein |
| CEBPA | CCAAT/enhancer-binding protein alpha |
| IL33 | Interleukin-33 |
| COL1A2 | Collagen alpha-2 |
| MIR454 | MicroRNA 454 |
| CHRM3 | Muscarinic acetylcholine receptor M3 |
| SEMA3F | Semaphorin-3F |
| CGB3 | Choriogonadotropin subunit beta 3 |
| S100A1 | Protein S100-A1 |
| IFNB1 | Interferon beta |
| CTCF | Transcriptional repressor CTCF |
| GLI2 | Zinc finger protein GLI2 |
| GH1 | Somatotropin |
| CUL1 | Cullin-1 |
| ANXA3 | Annexin A3 |
| KRT17 | Keratin, type I cytoskeletal 17 |
| ANTXR1 | Anthrax toxin receptor 1 |
| MIR425 | MicroRNA 425 |
| CD276 | CD276 antigen |
| REN | Renin |
| ACTG2 | Actin, gamma-enteric smooth muscle |
| SYK | Tyrosine-protein kinase SYK |
| PPP2R1A | Serine/threonine-protein phosphatase 2A 65 kDa regulatory subunit A alpha isoform |
| ADORA1 | Adenosine receptor A1 |
| VCP | Transitional endoplasmic reticulum ATPase |
| TPD52 | Tumor protein D52 |
| PIN1 | Peptidyl-prolyl cis-trans isomerase NIMA-interacting 1 |
| GPX1 | Glutathione peroxidase 1 |
| ACTB | Actin, cytoplasmic 1 |
| REG4 | Regenerating islet-derived protein 4 |
| MAP3K1 | Mitogen-activated protein kinase kinase kinase 1 |
| MSN | Moesin |
| RETN | Resistin |
| MECP2 | Methyl-CpG-binding protein 2 |
| SEPTIN9 | Septin-9 |
| NNT-AS1 | NNT Antisense RNA 1 |
| ESRRA | Steroid hormone receptor ERR1 |
| MIR340 | MicroRNA 340 |
| CD99 | CD99 antigen |
| TPO | Thyroid peroxidase |
| IRF5 | Interferon regulatory factor 5 |
| IL7R | Interleukin-7 receptor subunit alpha |
| EEF1A1 | Elongation factor 1-alpha 1 |
| NPY | Pro-neuropeptide Y |
| PTPN1 | Tyrosine-protein phosphatase non-receptor type 1 |
| DNAH8 | Dynein heavy chain 8, axonemal |
| CCL20 | C-C motif chemokine 20 |
| FOXD2-AS1 | FOXD2 Adjacent Opposite Strand RNA 1 |
| IL23R | Interleukin-23 receptor |
| SNHG6 | Small Nucleolar RNA Host Gene 6 |
| RIOX2 | Ribosomal oxygenase 2 |
| CTAG2 | Cancer/testis antigen 2 |
| SPINT1 | Kunitz-type protease inhibitor 1 |
| GNRHR | Gonadotropin-releasing hormone receptor |
| PRODH | Proline dehydrogenase 1, mitochondrial |
| ACHE | Acetylcholinesterase |
| APOA1 | Apolipoprotein A-I |
| PAX2 | Paired box protein Pax-2 |
| ACTG1 | Actin, cytoplasmic 2 |
| PRNP | Major prion protein |
| CA2 | Carbonic anhydrase 2 |
| PRKG1 | cGMP-dependent protein kinase 1 |
| FGF3 | Fibroblast growth factor 3 |
| DCN | Decorin |
| NLRP3 | NACHT, LRR and PYD domains-containing protein 3 |
| PAX6 | Paired box protein Pax-6 |
| DRD2 | D(2) dopamine receptor |
| COL4A3 | Collagen alpha-3 |
| IFIH1 | Interferon-induced helicase C domain-containing protein 1 |
| AGK | Acylglycerol kinase, mitochondrial |
| DBH | Dopamine beta-hydroxylase |
| CYP11A1 | Cholesterol side-chain cleavage enzyme, mitochondrial |
| STIM1 | Stromal interaction molecule 1 |
| DMD | Dystrophin |
| XDH | Xanthine dehydrogenase/oxidase |
| ITGB2 | Integrin beta-2 |
| CHAT | Choline O-acetyltransferase |
| RELB | Transcription factor RelB |
| CHD7 | Chromodomain-helicase-DNA-binding protein 7 |
| DACT1 | Dapper homolog 1 |
| COL4A2 | Collagen alpha-2 |
| CRH | Corticoliberin |
| CR1 | Complement receptor type 1 |
| SALL4 | Sal-like protein 4 |
| BCR | Breakpoint cluster region protein |
| KNG1 | Kininogen-1 |
| MTRR | Methionine synthase reductase |
| MYO18B | Unconventional myosin-XVIIIb |
| F10 | Coagulation factor X |
| LDLR | Low-density lipoprotein receptor |
| NR3C2 | Mineralocorticoid receptor |
| MYH11 | Myosin-11 |
| BMPR1B | Bone morphogenetic protein receptor type-1B |
| F8 | Coagulation factor VIII |
| FOXC2 | Forkhead box protein C2 |
| INHBA | Inhibin beta A chain |
| PITX2 | Pituitary homeobox 2 |
| FGA | Fibrinogen alpha chain |
| PSEN1 | Presenilin-1 |
| HSD3B2 | 3 beta-hydroxysteroid dehydrogenase/Delta 5-->4-isomerase type 2 |
| SMPD1 | Sphingomyelin phosphodiesterase |
| BTK | Tyrosine-protein kinase BTK |
| KRT10 | Keratin, type I cytoskeletal 10 |
| HELLS | Lymphoid-specific helicase |
| HNRNPK | Heterogeneous nuclear ribonucleoprotein K |
| ERCC3 | General transcription and DNA repair factor IIH helicase subunit XPB |
| SLCO1B3 | Solute carrier organic anion transporter family member 1B3 |
| GNAQ | Guanine nucleotide-binding protein G |
| PLK4 | Serine/threonine-protein kinase PLK4 |
| SERPINF1 | Pigment epithelium-derived factor |
| RASA1 | Ras GTPase-activating protein 1 |
| WNT7A | Protein Wnt-7a |
| ACP5 | Tartrate-resistant acid phosphatase type 5 |
| SDHA | Succinate dehydrogenase [ubiquinone] flavoprotein subunit, mitochondrial |
| NEU1 | Sialidase-1 |
| PAX5 | Paired box protein Pax-5 |
| PKD1 | Polycystin-1 |
| FOLR1 | Folate receptor alpha |
| FLNB | Filamin-B |
| LAMA2 | Laminin subunit alpha-2 |
| EYA1 | Eyes absent homolog 1 |
| PLA2G4A | Cytosolic phospholipase A2 |
| SEMA3A | Semaphorin-3A |
| HMGCR | 3-hydroxy-3-methylglutaryl-coenzyme A reductase |
| AHCY | Adenosylhomocysteinase |
| CYP2C19 | Cytochrome P450 2C19 |
| IL21 | Interleukin-21 |
| TPI1 | Triosephosphate isomerase |
| CDH11 | Cadherin-11 |
| KCNQ1 | Potassium voltage-gated channel subfamily KQT member 1 |
| RUNX1 | Runt-related transcription factor 1 |
| AVP | Vasopressin-neurophysin 2-copeptin |
| CR2 | Complement receptor type 2 |
| ACP1 | Low molecular weight phosphotyrosine protein phosphatase |
| PSEN2 | Presenilin-2 |
| PRF1 | Perforin-1 |
| LBR | Delta(14)-sterol reductase LBR |
| KMT2A | Histone-lysine N-methyltransferase 2A |
| PIK3CD | Phosphatidylinositol 4,5-bisphosphate 3-kinase catalytic subunit delta isoform |
| CBS | Cystathionine beta-synthase |
| RBP4 | Retinol-binding protein 4 |
| RAP1A | Ras-related protein Rap-1A |
| HADHA | Trifunctional enzyme subunit alpha, mitochondrial |
| TAC1 | Protachykinin-1 |
| PHOX2B | Paired mesoderm homeobox protein 2B |
| KCNH1 | Potassium voltage-gated channel subfamily H member 1 |
| CDKN2C | Cyclin-dependent kinase 4 inhibitor C |
| HSD11B2 | Corticosteroid 11-beta-dehydrogenase isozyme 2 |
| FBLN5 | Fibulin-5 |
| CTSG | Cathepsin G |
| INPPL1 | Phosphatidylinositol 3,4,5-trisphosphate 5-phosphatase 2 |
| CCR1 | C-C chemokine receptor type 1 |
| GAL | Galanin peptides |
| SELL | L-selectin |
| PARK7 | Parkinson disease protein 7 |
| GATA1 | Erythroid transcription factor |
| CX3CR1 | CX3C chemokine receptor 1 |
| WNT10A | Protein Wnt-10a |
| SLC6A2 | Sodium-dependent noradrenaline transporter |
| ADM | ADM |
| CGA | Glycoprotein hormones alpha chain |
| SLC11A1 | Natural resistance-associated macrophage protein 1 |
| IL10RA | Interleukin-10 receptor subunit alpha |
| MECOM | Histone-lysine N-methyltransferase MECOM |
| TGFBI | Transforming growth factor-beta-induced protein ig-h3 |
| MC1R | Melanocyte-stimulating hormone receptor |
| TNFRSF8 | Tumor necrosis factor receptor superfamily member 8 |
| EIF2AK2 | Interferon-induced, double-stranded RNA-activated protein kinase |
| LYN | Tyrosine-protein kinase Lyn |
| ZAP70 | Tyrosine-protein kinase ZAP-70 |
| TAF15 | TATA-binding protein-associated factor 2N |
| KRT13 | Keratin, type I cytoskeletal 13 |
| RAD54L | DNA repair and recombination protein RAD54-like |
| BCAR3 | Breast cancer anti-estrogen resistance protein 3 |
| FALEC | Focally amplified lncRNA on chromosome 1 |
| COPA | Coatomer subunit alpha |
| FMO2 | Dimethylaniline monooxygenase [N-oxide-forming] 2 |
| SDCCAG8 | Serologically defined colon cancer antigen 8 |
| GACAT3 | Gastric cancer-associated transcript 3, noncoding |
| KCNK3 | Potassium channel subfamily K member 3 |
| ECRG4 | Augurin |
| SCHLAP1 | SWI/SNF complex antagonist associated with prostate cancer 1, noncoding |
| DIRC1 | Disrupted in renal carcinoma protein 1 |
| CPS1 | Carbamoyl-phosphate synthase [ammonia], mitochondrial |
| FARSB | Phenylalanine-tRNA synthetase-like, beta subunit |
| RABL3 | Rab-like protein 3 |
| SLC34A2 | Sodium-dependent phosphate transport protein 2B |
| PCAT4 | Prostate cancer-associated transcript 4 |
| ADH1B | All-trans-retinol dehydrogenase |
| CCDC110 | Cancer/testis antigen KM-HN-1 |
| DAB2 | Disabled homolog 2 |
| LUCAT1 | Lung cancer-associated transcript 1, noncoding |
| NQO2 | Ribosyldihydronicotinamide dehydrogenase [quinone] |
| CAGE1 | Cancer-associated gene 1 protein |
| CASC15 | Cancer susceptibility candidate 15, noncoding |
| PBOV1 | Prostate and breast cancer overexpressed gene 1 protein |
| SASH1 | SAM and SH3 domain-containing protein 1 |
| TIAM2 | T-lymphoma invasion and metastasis-inducing protein 2 |
| CAHM | Colorectal adenocarcinoma hypermethylated gene, noncoding |
| VOPP1 | Vesicular, overexpressed in cancer, prosurvival protein 1 |
| PTPN12 | Tyrosine-protein phosphatase non-receptor type 12 |
| RB1CC1 | RB1-inducible coiled-coil protein 1 |
| MTSS1 | Protein MTSS 1 |
| PCAT2 | Prostate cancer-associated transcript 2, noncoding |
| CASC19 | Cancer susceptibility candidate 19, noncoding |
| CASC21 | Cancer susceptibility candidate 21, noncoding |
| CASC11 | Cancer susceptibility candidate 11, noncoding |
| PCA3 | Prostate cancer antigen 3 |
| DELEC1 | Deleted in esophageal cancer 1 |
| ERCC6 | Chimeric ERCC6-PGBD3 protein |
| SFTPA2 | Pulmonary surfactant-associated protein A2 |
| SFTPA1 | Pulmonary surfactant-associated protein A1 |
| SNCG | Gamma-synuclein |
| HABP2 | Hyaluronan-binding protein 2 |
| FAM111B | Protein FAM111B |
| MTA2 | Metastasis-associated protein MTA2 |
| LTO1 | Oral cancer overexpressed gene 1 |
| COLCA1 | Colorectal cancer-associated protein 1 |
| HEPN1 | Putative cancer susceptibility gene HEPN1 protein |
| OPCML | Opioid-binding protein/cell adhesion molecule |
| CFAP94 | Cancer susceptibility candidate 1 |
| ACVR1B | Activin receptor type-1B |
| MARS1 | Methionine--tRNA ligase, cytoplasmic |
| SRGAP1 | SLIT-ROBO Rho GTPase-activating protein 1 |
| ZCCHC8 | Zinc finger CCHC domain-containing protein 8 |
| CDK2AP1 | Cyclin-dependent kinase 2-associated protein 1 |
| SMAD9 | Mothers against decapentaplegic homolog 9 |
| MLH3 | DNA mismatch repair protein Mlh3 |
| NSMCE3 | Non-structural maintenance of chromosomes element 3 homolog |
| EIF2AK4 | eIF-2-alpha kinase GCN2 |
| PCAT29 | Prostate cancer-associated transcript 29, noncoding |
| CHRNA5 | Neuronal acetylcholine receptor subunit alpha-5 |
| CHRNA3 | Neuronal acetylcholine receptor subunit alpha-3 |
| IQGAP1 | Ras GTPase-activating-like protein IQGAP1 |
| ABCA3 | ATP-binding cassette sub-family A member 3 |
| PARN | Poly(A)-specific ribonuclease PARN |
| MTSS2 | Metastasis suppressor 1-like protein |
| OVCA2 | Esterase OVCA2 |
| HIC1 | Hypermethylated in cancer 1 protein |
| PCAP | Predisposing for prostate cancer |
| COPD | Pulmonary disease, chronic obstructive, severe early-onset |
| LCO | Liver cancer oncogene |
| HPC5 | Prostate cancer, hereditary, 5 |
| OVCAS1 | Ovarian cancer, susceptibility to, 1 |
| SCLC1 | Small-cell cancer of lung |
| LNCR5 | Lung cancer susceptibility 5 |
| TAPVR1 | Total anomalous pulmonary venous return 1 |
| LNCR3 | Lung cancer susceptibility 3 |
| CTEPH1 | Pulmonary hypertension, chronic thromboembolic, without deep vein thrombosis, susceptibility to |
| LNCR4 | Lung cancer susceptibility 4 |
| PLF | Pulmonary function |
| LNCR1 | Lung cancer 1 |
| TTIM1 | T-cell tumor invasion and metastasis-1 (invasion-metastasis of neoplasms, chromosome 7 determined) |
| HPC4 | Prostate cancer, hereditary, 4 |
| GAEC1 | Gene amplified in esophageal cancer 1 |
| CRCS6 | Colorectal cancer, susceptibility to, 6 |
| CRCS2 | Colorectal cancer, susceptibility to, 2 |
| HPC10 | Prostate cancer, hereditary, 10 |
| CRCS5 | Colorectal cancer, susceptibility to, 5 |
| HPC14 | Prostate cancer, hereditary, 14 |
| GCRG224 | Putative gastric cancer-related gene 224 protein |
| CRCS7 | Colorectal cancer, susceptibility to, 7 |
| TSG11 | Tumor suppressor gene on chromosome 11 |
| CRCS8 | Colorectal cancer, susceptibility to, 8 |
| HPC7 | Prostate cancer, hereditary, 7 |
| CRCS4 | Polyposis syndrome, mixed, hereditary 1 |
| DUP15q | Polyposis syndrome, mixed, hereditary 1 |
| C15DUPq | Polyposis syndrome, mixed, hereditary 1 |
| CRAC1 | Polyposis syndrome, mixed, hereditary 1 |
| HMPS1 | Polyposis syndrome, mixed, hereditary 1 |
| CRCS9 | Colorectal cancer, susceptibility to, 9 |
| BCPR | Breast cancer-related regulator of TP53 |
| HPC11 | Prostate cancer, hereditary, 11 |
| HPC9 | Prostate cancer, hereditary, 9 |
| WHS | Wolf-Hirschhorn syndrome |
| SF | Stoltzfus blood group |
| CIMT | Carotid intimal medial thickness |
| ARVD3 | Arrhythmogenic right ventricular dysplasia 3 |
| CLCNKA | Chloride channel protein ClC-Ka |
| RLF | Zinc finger protein Rlf |
| HOXD12 | Homeobox protein Hox-D12 |
| MIR1258 | Micro RNA 1258 |
| EOMES | Eomesodermin homolog |
| CP | Ceruloplasmin |
| PAICS | Multifunctional protein ADE2 |
| PF4 | Platelet factor 4 |
| PPBP | Platelet basic protein |
| LST1 | Leukocyte-specific transcript 1 protein |
| C4B | Complement C4-B |
| GNB2 | Guanine nucleotide-binding protein G |
| ADRA1A | Adrenergic, alpha-1C-, receptor |
| TAL2 | T-cell acute lymphocytic leukemia protein 2 |
| MLLT10 | Protein AF-10 |
| FOLR2 | Folate receptor beta |
| SNORD15A | RNA, U15a small nucleolar |
| EMSY | BRCA2-interacting transcriptional repressor EMSY |
| NR2F2 | COUP transcription factor 2 |
| ARRB1 | Beta-arrestin-1 |
| TSPO | Putative peripheral benzodiazepine receptor-related protein |
| VCAN | Versican core protein |
| FPR1 | fMet-Leu-Phe receptor |
| TACSTD2 | Tumor-associated calcium signal transducer 2 |
| GAS1 | Growth arrest-specific protein 1 |
| GHRH | Somatoliberin |
| HPN | Serine protease hepsin |
| LAG3 | Lymphocyte activation gene 3 protein |
| PI5 | Protease Inhibitor 5 |
| MC4R | Melanocortin receptor 4 |
| NME2 | Nucleoside diphosphate kinase B |
| CCL7 | C-C motif chemokine 7 |
| FGF4 | Fibroblast growth factor 4 |
| POLD1 | DNA polymerase delta catalytic subunit |
| PTPN3 | Tyrosine-protein phosphatase non-receptor type 3 |
| SERPINE2 | Glia-derived nexin |
| PTPRF | Receptor-type tyrosine-protein phosphatase F |
| RCVRN | Recoverin |
| RPA1 | Replication protein A 70 kDa DNA-binding subunit |
| ARID4A | AT-rich interactive domain-containing protein 4A |
| RPS6 | 40S ribosomal protein S6 |
| POLR2A | DNA-directed RNA polymerase II subunit RPB1 |
| SEMG1 | Semenogelin-1 |
| ANXA7 | Annexin A7 |
| MYB | Transcriptional activator Myb |
| TM4SF1 | Transmembrane 4 L6 family member 1 |
| XBP1 | X-box-binding protein 1 |
| TMSB4X | Thymosin beta-4 |
| PAGE1 | P antigen family member 1 |
| SPANXC | Sperm protein associated with the nucleus on the X chromosome C |
| ARMCX1 | Armadillo repeat-containing X-linked protein 1 |
| ARMCX2 | Armadillo repeat-containing X-linked protein 2 |
| ARMCX3 | Armadillo repeat-containing X-linked protein 3 |
| LDOC1 | Protein LDOC1 |
| MAGEC2 | Melanoma-associated antigen C2 |
| CT47A11 | Cancer/Testis Antigen Family 47, menber A11 |
| GAGE1 | G antigen 1 |
| GAGE2C | G Antigen 2C |
| GAGE7 | G antigen 7 |
| CT45A1 | Cancer/testis antigen family 45 member A1 |
| CTAG1A | Cancer/testis antigen 1 |
| STARD8 | StAR-related lipid transfer protein 8 |
| TFDP3 | Transcription factor Dp family member 3 |
| CT47A1 | Cancer/testis antigen 47A |
| CT47A2 | Cancer/Testis Antigen Family 47, menber A2 |
| CT47A3 | Cancer/Testis Antigen Family 47, menber A3 |
| CT47A4 | Cancer/Testis Antigen Family 47, menber A4 |
| CT47A5 | Cancer/Testis Antigen Family 47, menber A5 |
| CT47A6 | Cancer/Testis Antigen Family 47, menber A6 |
| CT47A7 | Cancer/Testis Antigen Family 47, menber A7 |
| CT47A8 | Cancer/Testis Antigen Family 47, menber A8 |
| CT47A9 | Cancer/Testis Antigen Family 47, menber A9 |
| CT47A10 | Cancer/Testis Antigen Family 47, menber A10 |
| CT47B1 | Cancer/testis antigen 47B |
| CT45A2 | Cancer/testis antigen family 45 member A2 |
| CT45A3 | Cancer/testis antigen family 45 member A3 |
| CT45A4 | Cancer/Testis Antigen Family 45, menber A4 |
| CT45A5 | Cancer/testis antigen family 45 member A5 |
| CT45A6 | Cancer/testis antigen family 45 member A6 |
| PSMD10 | 26S proteasome non-ATPase regulatory subunit 10 |
| PASD1 | Circadian clock protein PASD1 |
| ZMAT1 | Zinc finger matrin-type protein 1 |
| PAGE5 | P antigen family member 5 |
| TMSB15B | Thymosin beta-15B |
| FUNDC2 | FUN14 domain-containing protein 2 |
| FMR1 | Synaptic functional regulator FMR1 |
| MTND1 | Complex I, subunit ND1 |
| MTND4L | Complex I, subunit ND4L |
| MTND6 | Complex I, subunit ND6 |
| MTCYB | Cytochrome b of Complex III |
| MTCO1 | Complex IV, Cytochrome c Oxidase subunitT I |
| MTCO2 | Complex IV, Cytochrome c Oxidase subunitT II |
| NNMT | Nicotinamide N-methyltransferase |
| MAP3K11 | Mitogen-activated protein kinase kinase kinase 11 |
| ID3 | DNA-binding protein inhibitor ID-3 |
| PCM1 | Pericentriolar material 1 protein |
| TYRO3 | Tyrosine-protein kinase receptor TYRO3 |
| RPS8 | 40S ribosomal protein S8 |
| ID2 | DNA-binding protein inhibitor ID-2 |
| GPC1 | Glypican-1 |
| CSNK1A1 | Casein kinase I isoform alpha |
| SERPINB4 | Serpin B4 |
| PLCL1 | Inactive phospholipase C-like protein 1 |
| EPHB1 | Ephrin type-B receptor 1 |
| IHH | Indian hedgehog protein |
| TGFBR3 | Transforming growth factor beta receptor type 3 |
| PDCD2 | Programmed cell death protein 2 |
| FAT1 | Protocadherin Fat 1 |
| PLXNB1 | Plexin-B1 |
| PRDM2 | PR domain zinc finger protein 2 |
| SIX1 | Homeobox protein SIX1 |
| MSRA | Mitochondrial peptide methionine sulfoxide reductase |
| CCR2 | C-C chemokine receptor type 2 |
| C1QBP | Complement component 1 Q subcomponent-binding protein, mitochondrial |
| SFN | 14-3-3 protein sigma |
| MIA | Melanoma-derived growth regulatory protein |
| GPR68 | Ovarian cancer G-protein coupled receptor 1 |
| GRB7 | Growth factor receptor-bound protein 7 |
| HIP1 | Huntingtin-interacting protein 1 |
| MGAT5 | Alpha-1,6-mannosylglycoprotein 6-beta-N-acetylglucosaminyltransferase A |
| NME3 | Nucleoside diphosphate kinase 3 |
| NME4 | Nucleoside diphosphate kinase, mitochondrial |
| NEO1 | Neogenin |
| TIMP4 | Metalloproteinase inhibitor 4 |
| KLF2 | Krueppel-like factor 2 |
| PLCD1 | 1-phosphatidylinositol 4,5-bisphosphate phosphodiesterase delta-1 |
| SIPA1 | Signal-induced proliferation-associated protein 1 |
| NEDD9 | Enhancer of filamentation 1 |
| ADAM8 | Disintegrin and metalloproteinase domain-containing protein 8 |
| GNRH2 | Progonadoliberin-2 |
| NR1H3 | Oxysterols receptor LXR-alpha |
| PTPRH | Receptor-type tyrosine-protein phosphatase H |
| PTPRK | Receptor-type tyrosine-protein phosphatase kappa |
| MAP3K7 | Mitogen-activated protein kinase kinase kinase 7 |
| TNFSF11 | Tumor necrosis factor ligand superfamily member 11 |
| FOXO3A | Forkhead Box O3A |
| NFATC4 | Nuclear factor of activated T-cells, cytoplasmic 4 |
| BRINP1 | BMP/retinoic acid-inducible neural-specific protein 1 |
| VWA5A | von Willebrand factor A domain-containing protein 5A |
| BCAS1 | Breast carcinoma-amplified sequence 1 |
| PCDH7 | Protocadherin-7 |
| RNF139 | E3 ubiquitin-protein ligase RNF139 |
| S100A11 | Protein S100-A11 |
| PTPN14 | Tyrosine-protein phosphatase non-receptor type 14 |
| CDK8 | Cyclin-dependent kinase 8 |
| CST7 | Cystatin-F |
| CHD4 | Chromodomain-helicase-DNA-binding protein 4 |
| CTSV | Cathepsin L2 |
| TNFRSF25 | Tumor necrosis factor receptor superfamily member 25 |
| TRIM24 | Transcription intermediary factor 1-alpha |
| BTRC | F-box/WD repeat-containing protein 1A |
| NFS1 | Cysteine desulfurase, mitochondrial |
| DPH1 | 2-(3-amino-3-carboxypropyl)histidine synthase subunit 1 |
| MBD4 | Methyl-CpG-binding domain protein 4 |
| TRPM1 | Transient receptor potential cation channel subfamily M member 1 |
| UNC5C | Netrin receptor UNC5C |
| RPS5 | 40S ribosomal protein S5 |
| RPS9 | 40S ribosomal protein S9 |
| RPS10 | 40S ribosomal protein S10 |
| RPS29 | 40S ribosomal protein S29 |
| RPL5 | 60S ribosomal protein L5 |
| RPL21 | 60S ribosomal protein L21 |
| RPL27A | 60S ribosomal protein L27a |
| RPL28 | 60S ribosomal protein L28 |
| RPS12 | 40S ribosomal protein S12 |
| RPS20 | 40S ribosomal protein S20 |
| BUB3 | Mitotic checkpoint protein BUB3 |
| SLC43A1 | Large neutral amino acids transporter small subunit 3 |
| DACH1 | Dachshund homolog 1 |
| ITGA9 | Integrin alpha-9 |
| ERN1 | Serine/threonine-protein kinase/endoribonuclease IRE1 |
| ADGRG1 | Adhesion G-protein coupled receptor G1 |
| RBBP8 | DNA endonuclease RBBP8 |
| SYT7 | Synaptotagmin-7 |
| KISS1R | KiSS-1 receptor |
| SLC25A11 | Mitochondrial 2-oxoglutarate/malate carrier protein |
| INTS6 | Integrator complex subunit 6 |
| GPNMB | Transmembrane glycoprotein NMB |
| STEAP1 | Metalloreductase STEAP1 |
| BAMBI | BMP and activin membrane-bound inhibitor homolog |
| CBLB | E3 ubiquitin-protein ligase CBL-B |
| PPARGC1A | Peroxisome proliferator-activated receptor gamma coactivator 1-alpha |
| MUC12 | Mucin-12 |
| RECQL3 | RECQ Protein-like 3 |
| AKAP13 | A-kinase anchor protein 13 |
| MERTK | Tyrosine-protein kinase Mer |
| LTBP4 | Latent-transforming growth factor beta-binding protein 4 |
| ARHGEF12 | Rho guanine nucleotide exchange factor 12 |
| CTNNAL1 | Alpha-catulin |
| TARBP2 | RISC-loading complex subunit TARBP2 |
| STEAP2 | Metalloreductase STEAP2 |
| ANO7 | Anoctamin-7 |
| SLC45A3 | Solute carrier family 45 member 3 |
| ENC1 | Ectoderm-neural cortex protein 1 |
| CHFR | E3 ubiquitin-protein ligase CHFR |
| ARHGEF4 | Rho guanine nucleotide exchange factor 4 |
| POLI | DNA polymerase iota |
| NCOA6 | Nuclear receptor coactivator 6 |
| PFKFB4 | 6-phosphofructo-2-kinase/fructose-2,6-bisphosphatase 4 |
| HOXC10 | Homeobox protein Hox-C10 |
| SCGB2A2 | Mammaglobin-A |
| IL23A | Interleukin-23 subunit alpha |
| PI14 | Protease Inhibitor 14 |
| HTATIP2 | Oxidoreductase HTATIP2 |
| BACE2 | Beta-secretase 2 |
| NOP53 | Ribosome biogenesis protein NOP53 |
| TNFRSF21 | Tumor necrosis factor receptor superfamily member 21 |
| HID1 | Protein HID1 |
| FGL1 | Fibrinogen-like protein 1 |
| EIF5A2 | Eukaryotic translation initiation factor 5A-2 |
| ANGPTL4 | Angiopoietin-related protein 4 |
| TNFRSF12A | Tumor necrosis factor receptor superfamily member 12A |
| MBOAT7 | Lysophospholipid acyltransferase 7 |
| CD248 | Endosialin |
| PBRM1 | Protein polybromo-1 |
| PIGN | GPI ethanolamine phosphate transferase 1 |
| RNF26 | E3 ubiquitin-protein ligase RNF26 |
| BHLHE41 | Class E basic helix-loop-helix protein 41 |
| SIRT6 | NAD-dependent protein deacetylase sirtuin-6 |
| EGLN1 | Egl nine homolog 1 |
| SCGB3A1 | Secretoglobin family 3A member 1 |
| CASC3 | Protein CASC3 |
| CYP27A1 | Sterol 26-hydroxylase, mitochondrial |
| HUNK | Hormonally up-regulated neu tumor-associated kinase |
| TMPRSS4 | Transmembrane protease serine 4 |
| GNMT | Glycine N-methyltransferase |
| DCD | Dermcidin |
| FXYD5 | FXYD domain-containing ion transport regulator 5 |
| PKHD1 | Fibrocystin |
| PARD3 | Partitioning defective 3 homolog |
| ST13 | Hsc70-interacting protein |
| ADGRA2 | Adhesion G protein-coupled receptor A2 |
| TNS3 | Tensin-3 |
| PLXDC2 | Plexin domain-containing protein 2 |
| RBM5 | RNA-binding protein 5 |
| ERBIN | Erbin |
| TNK2 | Activated CDC42 kinase 1 |
| SEZ6L | Seizure 6-like protein |
| STARD3 | StAR-related lipid transfer protein 3 |
| FANCA | Fanconi anemia group A protein |
| ORAOV1 | Oral cancer overexpressed gene 1 |
| SETD8 | SET Domain-Containing Protein 8 |
| NDC80 | Kinetochore protein NDC80 homolog |
| LSM1 | U6 snRNA-associated Sm-like protein LSm1 |
| TGIF2 | Homeobox protein TGIF2 |
| TLN2 | Talin-2 |
| RHOBTB2 | Rho-related BTB domain-containing protein 2 |
| CCAR2 | Cell cycle and apoptosis regulator protein 2 |
| BPIFA1 | BPI fold-containing family A member 1 |
| DOCK4 | Dedicator of cytokinesis protein 4 |
| HIC2 | Hypermethylated in cancer 2 protein |
| SARM1 | NAD(+) hydrolase SARM1 |
| UNC5A | Netrin receptor UNC5A |
| UNC5B | Netrin receptor UNC5B |
| LIMS2 | LIM and senescent cell antigen-like-containing domain protein 2 |
| KCNRG | Potassium channel regulatory protein |
| SEMA3E | Semaphorin-3E |
| IGF2BP1 | Insulin-like growth factor 2 mRNA-binding protein 1 |
| IGF2BP2 | Insulin-like growth factor 2 mRNA-binding protein 2 |
| CTAG3 | Cancer/testis antigen 3 |
| NEMF | Nuclear export mediator factor NEMF |
| GSDMC | Gasdermin-C |
| GLDN | Gliomedin |
| SMYD3 | Histone-lysine N-methyltransferase SMYD3 |
| BLID | BH3-like motif-containing cell death inducer |
| MTA3 | Metastasis-associated protein MTA3 |
| AKIP1 | A-kinase-interacting protein 1 |
| ARL11 | ADP-ribosylation factor-like protein 11 |
| AGR3 | Anterior gradient protein 3 |
| FAM84B | Family with Sequence Similarity 84, menber B |
| LYPD3 | Ly6/PLAUR domain-containing protein 3 |
| RSPO1 | R-spondin-1 |
| KLF17 | Krueppel-like factor 17 |
| NECTIN4 | Nectin-4 |
| EIF3M | Eukaryotic translation initiation factor 3 subunit M |
| ARID4B | AT-rich interactive domain-containing protein 4B |
| PSMG2 | Proteasome assembly chaperone 2 |
| XRRA1 | X-ray radiation resistance-associated protein 1 |
| STARD13 | StAR-related lipid transfer protein 13 |
| COL23A1 | Collagen alpha-1 |
| H2AFY | H2A Histone Family, menber Y |
| ST6GALNAC5 | Alpha-N-acetylgalactosaminide alpha-2,6-sialyltransferase 5 |
| ST6GALNAC1 | Alpha-N-acetylgalactosaminide alpha-2,6-sialyltransferase 1 |
| TBC1D3B | TBC1 domain family member 3B |
| EPB41L4B | Band 4.1-like protein 4B |
| LEMD1 | LEM domain-containing protein 1 |
| RSPO2 | R-spondin-2 |
| BOP1 | Ribosome biogenesis protein BOP1 |
| ALKBH3 | Alpha-ketoglutarate-dependent dioxygenase alkB homolog 3 |
| PLPP5 | Phospholipid phosphatase 5 |
| ADRM1 | Proteasomal ubiquitin receptor ADRM1 |
| PDZD2 | PDZ domain-containing protein 2 |
| HSPH1 | Heat shock protein 105 kDa |
| ANKRD30A | Ankyrin repeat domain-containing protein 30A |
| AGPAT9 | 1-Acylglycerol-3-Phosphate O-Acyltransferase 9 |
| MEX3C | RNA-binding E3 ubiquitin-protein ligase MEX3C |
| ARMC3 | Armadillo repeat-containing protein 3 |
| FAM84A | Family with Sequence Similarity 84, menber A |
| CENPW | Centromere protein W |
| SCARA5 | Scavenger receptor class A member 5 |
| TOX3 | TOX high mobility group box family member 3 |
| STYK1 | Tyrosine-protein kinase STYK1 |
| PLEKHM1 | Pleckstrin homology domain-containing family M member 1 |
| PLA2G3 | Group 3 secretory phospholipase A2 |
| TMPRSS11A | Transmembrane protease serine 11A |
| C2ORF40 | Esophageal cancer-related gene 4 |
| USHBP1 | Usher syndrome type-1C protein-binding protein 1 |
| ATAD2 | ATPase family AAA domain-containing protein 2 |
| TNFAIP8 | Tumor necrosis factor alpha-induced protein 8 |
| PREX2 | Phosphatidylinositol 3,4,5-trisphosphate-dependent Rac exchanger 2 protein |
| RPRM | Protein reprimo |
| MSMP | Prostate-associated microseminoprotein |
| ABHD1 | Protein ABHD1 |
| ABHD2 | Monoacylglycerol lipase ABHD2 |
| ABHD3 | Phospholipase ABHD3 |
| ATG9B | Autophagy-related protein 9B |
| B4GALNT3 | Beta-1,4-N-acetylgalactosaminyltransferase 3 |
| MIRN610 | micro RNA 610 |
| STING1 | Stimulator of interferon genes protein |
| MED19 | Mediator of RNA polymerase II transcription subunit 19 |
| MIRN101-1 | micro RNA 101-1 |
| RFX6 | DNA-binding protein RFX6 |
| PYHIN1 | Pyrin and HIN domain-containing protein 1 |
| CCNY | Cyclin-Y |
| ZKSCAN3 | Zinc finger protein with KRAB and SCAN domains 3 |
| UTP20 | Small subunit processome component 20 homolog |
| RNASET2 | Ribonuclease T2 |
| CHD1L | Chromodomain-helicase-DNA-binding protein 1-like |
| BLCAP | Bladder cancer-associated protein |
| LAPTM4B | Lysosomal-associated transmembrane protein 4B |
| SPATA13 | Spermatogenesis-associated protein 13 |
| DSCR8 | Down syndrome critical region protein 8 |
| LRRC26 | Leucine-rich repeat-containing protein 26 |
| ACKR1 | Atypical chemokine receptor 1 |
| MIR661 | micro RNA 661 |
| PRDM5 | PR domain zinc finger protein 5 |
| VTI1A | Vesicle transport through interaction with t-SNAREs homolog 1A |
| SCUBE3 | Signal peptide, CUB and EGF-like domain-containing protein 3 |
| CARMIL3 | Capping protein, Arp2/3 and myosin-I linker protein 3 |
| KCMF1 | E3 ubiquitin-protein ligase KCMF1 |
| STRN3 | Striatin-3 |
| NUPR1 | Nuclear protein 1 |
| TRIAP1 | TP53-regulated inhibitor of apoptosis 1 |
| ZNF516 | Zinc finger protein 516 |
| NPTNIT1 | NPTN Intronic Transcript 1, noncoding |
| RHBDD2 | Rhomboid domain-containing protein 2 |
| MIR297 | micro RNA 297 |
| TIMMDC1 | Complex I assembly factor TIMMDC1, mitochondrial |
| NLRC3 | NLR family CARD domain-containing protein 3 |
| RGS22 | Regulator of G-protein signaling 22 |
| BUD23 | Probable 18S rRNA |
| SUSD2 | Sushi domain-containing protein 2 |
| MIR190A | micro RNA 190A |
| GACAT2 | Gastric Cancer-associated Transcript 2, noncoding |
| SLC38A9 | Sodium-coupled neutral amino acid transporter 9 |
| NBAT1 | Neuroblastoma-associated Transcript 1, noncoding |
| C4ORF46 | Renal cancer differentiation gene 1 protein |
| CREB3L1 | Cyclic AMP-responsive element-binding protein 3-like protein 1 |
| ATG2B | Autophagy-related protein 2 homolog B |
| MIR520G | micro RNA 520G |
| FRMD4A | FERM domain-containing protein 4A |
| FRMD5 | FERM domain-containing protein 5 |
| FAM168A | Protein FAM168A |
| LAMB4 | Laminin subunit beta-4 |
| TNFAIP8L3 | Tumor necrosis factor alpha-induced protein 8-like protein 3 |
| MFAP3L | Microfibrillar-associated protein 3-like |
| ANKRD30B | Ankyrin repeat domain-containing protein 30B |
| ASAP3 | Arf-GAP with SH3 domain, ANK repeat and PH domain-containing protein 3 |
| TBC1D16 | TBC1 domain family member 16 |
| TAOK3 | Serine/threonine-protein kinase TAO3 |
| MYCT1 | Myc target protein 1 |
| TMEM9 | Transmembrane protein 9 |
| TMEM8B | Transmembrane protein 8B |
| CASC1 | Protein CASC1 |
| MTSS1L | Metastasis suppressor 1-like protein |
| ERGIC3 | Endoplasmic reticulum-Golgi intermediate compartment protein 3 |
| ZNF703 | Zinc finger protein 703 |
| CWC27 | Spliceosome-associated protein CWC27 homolog |
| NEURL3 | E3 ubiquitin-protein ligase NEURL3 |
| SPINK7 | Serine protease inhibitor Kazal-type 7 |
| SHC4 | SHC-transforming protein 4 |
| PCAT18 | Prostate Cancer-associated Transcript 18, noncoding |
| LINC01488 | Long Intergenic noncoding RNA 1488 |
| DDX46 | Probable ATP-dependent RNA helicase DDX46 |
| STRIP1 | Striatin-interacting protein 1 |
| STRIP2 | Striatin-interacting protein 2 |
| ZNF768 | Zinc finger protein 768 |
| DDIAS | DNA damage-induced apoptosis suppressor protein |
| COX6B2 | Cytochrome c oxidase subunit 6B2 |
| CEACAM21 | Carcinoembryonic antigen-related cell adhesion molecule 21 |
| PCAT19 | Prostate Cancer-associated Transcript 19, noncoding |
| CREB5 | Cyclic AMP-responsive element-binding protein 5 |
| VWA2 | von Willebrand factor A domain-containing protein 2 |
| ENTR1 | Endosome-associated-trafficking regulator 1 |
| KIAA1211 | KIAA1211 Gene |
| LINC00958 | Long Intergenic noncoding RNA 958 |
| PRR15 | Proline-rich protein 15 |
| GPR107 | Protein GPR107 |
| GPR108 | Protein GPR108 |
| BRMS1L | Breast cancer metastasis-suppressor 1-like protein |
| C2ORF68 | UPF0561 protein C2orf68 |
| CCDC33 | Coiled-coil domain-containing protein 33 |
| CKAP4 | Cytoskeleton-associated protein 4 |
| ZDHHC2 | Palmitoyltransferase ZDHHC2 |
| CACUL1 | CDK2-associated and cullin domain-containing protein 1 |
| RALGAPA2 | Ral GTPase-activating protein subunit alpha-2 |
| AFF1 | AF4/FMR2 family member 1 |
| MLLT3 | Protein AF-9 |
| AFDN | Afadin |
| FOXO4 | Forkhead box protein O4 |
| MAGEB1 | Melanoma-associated antigen B1 |
| MAGEB2 | Melanoma-associated antigen B2 |
| EPS15 | Epidermal growth factor receptor substrate 15 |
| MLLT6 | Protein AF-17 |
| RAB27A | Ras-related protein Rab-27A |
| AFF4 | AF4/FMR2 family member 4 |
| MLLT11 | Protein AF1q |
| NCKIPSD | NCK-interacting protein with SH3 domain |
| KNL1 | Kinetochore scaffold 1 |
| PCBD2 | Pterin-4-alpha-carbinolamine dehydratase 2 |
| AHCTF1 | Protein ELYS |
| BATF3 | Basic leucine zipper transcriptional factor ATF-like 3 |
| LAMTOR1 | Ragulator complex protein LAMTOR1 |
| TFAP2AAS2 | TFAP2A Antisense RNA 2 |
