# Supplementary material for: Study on the Action Mechanism of the Yifei Jianpi Tongfu Formula in Treatment of Colorectal Cancer Lung Metastasis Based on Network Analysis, Molecular Docking, and Experimental Validation
Source: Evid Based Complement Alternat Med. 2022 Jul 30;2022:6229444. doi: 10.1155/2022/6229444 (PMC9356795; doi:10.1155/2022/6229444)
Supplement: Supplementary Materials — Detailed information about the active compounds and targets identified in YJTF is shown in Supplementary Table 1. All of the disease-related targets for CRC lung metastasis are listed in Supplementary Table 2. Detailed information about the 81 overlapping targets identified as the key targets for studying the therapeutic effect of YJTF on CRC lung metastasis is shown in Supplementary Table 3. Detailed information about the PPI network is shown in Supplementary Table 4. Detailed information about the GO and KEGG enrichment analysis of the putative targets is shown in Supplementary Table 5. [file 6229444.f1.zip › Supplementary Table.3-revised.docx]

| Intersecting targets | |
| --- | --- |
| Gene Symbol | Protein names |
| AKR1B10 | Aldo-keto reductase family 1 member B10 |
| PTPN1 | Tyrosine-protein phosphatase non-receptor type 1 |
| ADORA1 | Adenosine receptor A1 |
| ADORA3 | Adenosine receptor A3 |
| AR | Androgen receptor |
| HMGCR | 3-hydroxy-3-methylglutaryl-coenzyme A reductase |
| NR1H3 | Oxysterols receptor LXR-alpha |
| CYP19A1 | Aromatase |
| XDH | Xanthine dehydrogenase/oxidase |
| CA2 | Carbonic anhydrase 2 |
| CYP1B1 | Cytochrome P450 1B1 |
| IL2 | Interleukin-2 |
| TYR | Tyrosinase |
| FLT3 | Receptor-type tyrosine-protein kinase FLT3 |
| ALOX5 | Arachidonate 5-lipoxygenase |
| ABCC1 | Multidrug resistance-associated protein 1 |
| HSD17B1 | Estradiol 17-beta-dehydrogenase 1 |
| AHR | Aryl hydrocarbon receptor |
| ESRRA | Steroid hormone receptor ERR1 |
| ABCB1 | ATP-dependent translocase ABCB1 |
| ABCG2 | Broad substrate specificity ATP-binding cassette transporter ABCG2 |
| ACHE | Acetylcholinesterase |
| SYK | Tyrosine-protein kinase SYK |
| GSK3B | Glycogen synthase kinase-3 beta |
| MMP9 | Matrix metalloproteinase-9 |
| MMP2 | 72 kDa type IV collagenase |
| ALOX15 | Arachidonate 15-lipoxygenase |
| ALOX12 | Polyunsaturated fatty acid lipoxygenase ALOX12 |
| ARG1 | Arginase-1 |
| ESR2 | Estrogen receptor beta |
| DAPK1 | Death-associated protein kinase 1 |
| TYMS | Thymidylate synthase |
| IGF1R | Insulin-like growth factor 1 receptor |
| EGFR | Epidermal growth factor receptor |
| F2 | Prothrombin |
| PIM1 | Serine/threonine-protein kinase pim-1 |
| AURKB | Aurora kinase B |
| MPO | Myeloperoxidase |
| PIK3R1 | Phosphatidylinositol 3-kinase regulatory subunit alpha |
| SRC | Proto-oncogene tyrosine-protein kinase Src |
| PTK2 | Focal adhesion kinase 1 |
| KDR | Vascular endothelial growth factor receptor 2 |
| MMP13 | Collagenase 3 |
| MMP3 | Stromelysin-1 |
| PLK1 | Serine/threonine-protein kinase PLK1 |
| CDK1 | Cyclin-dependent kinase 1 |
| CA9 | Carbonic anhydrase 9 |
| CSNK2A1 | Casein kinase II subunit alpha |
| MET | Hepatocyte growth factor receptor |
| CXCR1 | C-X-C chemokine receptor type 1 |
| ALK | ALK tyrosine kinase receptor |
| AKT1 | RAC-alpha serine/threonine-protein kinase |
| AXL | Tyrosine-protein kinase receptor UFO |
| AKR1C2 | Aldo-keto reductase family 1 member C2 |
| AKR1C1 | Aldo-keto reductase family 1 member C1 |
| AKR1C3 | Aldo-keto reductase family 1 member C3 |
| MAPT | Microtubule-associated protein tau |
| TOP2A | DNA topoisomerase 2-alpha |
| INSR | Insulin receptor |
| MYLK | Myosin light chain kinase, smooth muscle |
| PIK3CG | Phosphatidylinositol 4,5-bisphosphate 3-kinase catalytic subunit gamma isoform |
| APEX1 | DNA-(apurinic or apyrimidinic site) endonuclease |
| CDK6 | Cyclin-dependent kinase 6 |
| CDK2 | Cyclin-dependent kinase 2 |
| APP | Amyloid-beta precursor protein |
| PARP1 | Poly [ADP-ribose] polymerase 1 |
| TTR | Transthyretin |
| MMP12 | Macrophage metalloelastase |
| TOP1 | DNA topoisomerase 1 |
| CTSD | Cathepsin D |
| PTGES | Prostaglandin E synthase |
| NOS2 | Nitric oxide synthase, inducible |
| CYP17A1 | Steroid 17-alpha-hydroxylase/17,20 lyase |
| ESR1 | Estrogen receptor |
| PTGS2 | Prostaglandin G/H synthase 2 |
| CFTR | Cystic fibrosis transmembrane conductance regulator |
| NR3C1 | Glucocorticoid receptor |
| SHBG | Sex hormone-binding globulin |
| ODC1 | Ornithine decarboxylase |
| PPARD | Peroxisome proliferator-activated receptor delta |
| CCNB1 | G2/mitotic-specific cyclin-B1 |
