# Supplementary material for: Study on the Action Mechanism of the Yifei Jianpi Tongfu Formula in Treatment of Colorectal Cancer Lung Metastasis Based on Network Analysis, Molecular Docking, and Experimental Validation
Source: Evid Based Complement Alternat Med. 2022 Jul 30;2022:6229444. doi: 10.1155/2022/6229444 (PMC9356795; doi:10.1155/2022/6229444)
Supplement: Supplementary Materials — Detailed information about the active compounds and targets identified in YJTF is shown in Supplementary Table 1. All of the disease-related targets for CRC lung metastasis are listed in Supplementary Table 2. Detailed information about the 81 overlapping targets identified as the key targets for studying the therapeutic effect of YJTF on CRC lung metastasis is shown in Supplementary Table 3. Detailed information about the PPI network is shown in Supplementary Table 4. Detailed information about the GO and KEGG enrichment analysis of the putative targets is shown in Supplementary Table 5. [file 6229444.f1.zip › Supplementary Table.4.pdf]

node1	node2	node1_string_id	node2_string_id	neighborhood_on_chromosome	gene_fusion	phylogenetic_cooccurrence	homology	coexpression	experimentally_determined_interaction	database_annotated	automated_textmining	combined_score
ABCB1	APEX1	9606.ENSP00000478255	9606.ENSP00000216714	0.042	0	0	0	0.063	0.328	0	0.189	0.445
ABCB1	IL2	9606.ENSP00000478255	9606.ENSP00000226730	0	0	0	0	0	0	0	0.579	0.58
ABCB1	ABCG2	9606.ENSP00000478255	9606.ENSP00000237612	0	0	0	0.556	0.091	0.091	0	0.944	0.498
ABCB1	KDR	9606.ENSP00000478255	9606.ENSP00000263923	0	0	0	0	0.062	0	0	0.421	0.433
ABCB1	EGFR	9606.ENSP00000478255	9606.ENSP00000275493	0	0	0	0	0.062	0	0	0.748	0.754
ABCB1	AKR1B1	9606.ENSP00000478255	9606.ENSP00000285930	0	0	0	0	0.145	0.05	0	0.48	0.541
ABCB1	HMGCR	9606.ENSP00000478255	9606.ENSP00000287936	0	0	0	0	0	0.177	0	0.384	0.472
ABCB1	TYMS	9606.ENSP00000478255	9606.ENSP00000315644	0	0	0	0	0.063	0	0	0.679	0.687
ABCB1	TOP1	9606.ENSP00000478255	9606.ENSP00000354522	0	0	0	0	0.061	0.055	0	0.501	0.518
ABCB1	PTGS2	9606.ENSP00000478255	9606.ENSP00000356438	0	0	0	0	0	0	0	0.514	0.514
ABCB1	PIM1	9606.ENSP00000478255	9606.ENSP00000362608	0	0	0	0	0	0.404	0	0.408	0.632

ABCB1	SRC	9606.ENSP00000478255	9606.ENSP00000362680	0	0	0	0	0	0.057	0	0.639	0.646
ABCB1	ABCC1	9606.ENSP00000478255	9606.ENSP00000382342	0	0	0	0.591	0.052	0.05	0	0.931	0.417
ABCB1	ESR1	9606.ENSP00000478255	9606.ENSP00000405330	0	0	0	0	0	0	0	0.46	0.46
ABCB1	TOP2A	9606.ENSP00000478255	9606.ENSP00000411532	0	0	0	0	0.062	0.176	0	0.332	0.439
ABCB1	AKT1	9606.ENSP00000478255	9606.ENSP00000451828	0	0	0	0	0.061	0.057	0	0.579	0.594
ABCB1	CYP1B1	9606.ENSP00000478255	9606.ENSP00000478561	0	0	0	0	0.053	0	0	0.454	0.461
ABCC1	CSNK2A1	9606.ENSP00000382342	9606.ENSP00000217244	0	0	0	0	0.066	0.454	0	0.143	0.525
ABCC1	ABCG2	9606.ENSP00000382342	9606.ENSP00000237612	0	0	0	0	0.062	0.091	0	0.939	0.943
ABCC1	EGFR	9606.ENSP00000382342	9606.ENSP00000275493	0	0	0	0	0	0	0	0.454	0.454
ABCC1	AKR1B1	9606.ENSP00000382342	9606.ENSP00000285930	0	0	0	0	0.062	0.05	0	0.394	0.413
ABCC1	TYMS	9606.ENSP00000382342	9606.ENSP00000315644	0	0	0	0	0.061	0	0	0.526	0.535
ABCC1	TOP1	9606.ENSP00000382342	9606.ENSP00000354522	0	0	0	0	0	0.064	0	0.42	0.434
ABCC1	AKT1	9606.ENSP00000382342	9606.ENSP00000451828	0	0	0	0	0.062	0.057	0	0.658	0.672

ABCG2	CFTR	9606.ENSP00000237612	9606.ENSP00000003084	0	0	0	0	0.062	0.091	0	0.539	0.573
ABCG2	APEX1	9606.ENSP00000237612	9606.ENSP00000216714	0	0	0	0	0.061	0	0	0.464	0.476
ABCG2	SRC	9606.ENSP00000237612	9606.ENSP00000362680	0	0	0	0	0.062	0.056	0	0.38	0.403
ABCG2	CA4	9606.ENSP00000237612	9606.ENSP00000300900	0	0	0	0	0.084	0	0	0.386	0.414
ABCG2	AR	9606.ENSP00000237612	9606.ENSP00000363822	0	0	0	0	0	0.076	0	0.399	0.42
ABCG2	CYP1B1	9606.ENSP00000237612	9606.ENSP00000478561	0	0	0	0	0	0.05	0	0.418	0.423
ABCG2	MET	9606.ENSP00000237612	9606.ENSP00000317272	0	0	0	0	0.062	0.056	0	0.433	0.454
ABCG2	PIM1	9606.ENSP00000237612	9606.ENSP00000362608	0	0	0	0	0.063	0.379	0	0.166	0.472
ABCG2	TYMS	9606.ENSP00000237612	9606.ENSP00000315644	0	0	0	0	0	0	0	0.497	0.497
ABCG2	KDR	9606.ENSP00000237612	9606.ENSP00000263923	0	0	0	0	0.065	0.076	0	0.477	0.508
ABCG2	ESR2	9606.ENSP00000237612	9606.ENSP00000343925	0	0	0	0	0	0.076	0	0.507	0.525
ABCG2	TOP1	9606.ENSP00000237612	9606.ENSP00000354522	0	0	0	0	0	0	0	0.588	0.588
ABCG2	EGFR	9606.ENSP00000237612	9606.ENSP00000275493	0	0	0	0	0.08	0	0	0.647	0.661

ABCG2	ESR1	9606.ENSP00000237612	9606.ENSP00000405330	0	0	0	0	0	0.076	0	0.673	0.685
ABCG2	AHR	9606.ENSP00000237612	9606.ENSP00000242057	0	0	0	0	0	0	0	0.697	0.697
ABCG2	AKT1	9606.ENSP00000237612	9606.ENSP00000451828	0	0	0	0	0.062	0.056	0	0.753	0.763
ABCG2	SLC22A12	9606.ENSP00000237612	9606.ENSP00000366797	0	0	0	0	0.055	0.158	0	0.73	0.766
ACHE	DRD4	9606.ENSP00000303211	9606.ENSP00000176183	0	0	0	0	0	0	0	0.447	0.447
ACHE	MPO	9606.ENSP00000303211	9606.ENSP00000225275	0	0	0	0	0	0.064	0	0.41	0.424
ACHE	TYR	9606.ENSP00000303211	9606.ENSP00000263321	0	0	0	0	0	0	0	0.531	0.531
ACHE	KDR	9606.ENSP00000303211	9606.ENSP00000263923	0	0	0	0	0	0	0	0.427	0.426
ACHE	APP	9606.ENSP00000303211	9606.ENSP00000284981	0	0	0	0	0.091	0.379	0	0.73	0.834
ACHE	CA4	9606.ENSP00000303211	9606.ENSP00000300900	0	0	0	0	0.088	0	0	0.405	0.435
ACHE	F2	9606.ENSP00000303211	9606.ENSP00000308541	0	0	0	0	0.062	0.052	0	0.41	0.429
ACHE	PTGS2	9606.ENSP00000303211	9606.ENSP00000356438	0	0	0	0	0	0.064	0	0.437	0.451
ACHE	AKT1	9606.ENSP00000303211	9606.ENSP00000451828	0	0	0	0	0	0.062	0	0.448	0.46

ACHE	MAPT	9606.ENSP00000303211	9606.ENSP00000340820	0	0	0	0	0.095	0	0	0.555	0.58
ACHE	MAOA	9606.ENSP00000303211	9606.ENSP00000340684	0.045	0	0	0	0.095	0	0	0.593	0.617
ACHE	BACE1	9606.ENSP00000303211	9606.ENSP00000318585	0	0	0	0	0.063	0	0	0.646	0.654
ACHE	MAOB	9606.ENSP00000303211	9606.ENSP00000367309	0.045	0	0	0	0.107	0	0	0.673	0.697
ADORA1	DRD4	9606.ENSP00000356205	9606.ENSP00000176183	0	0	0	0.635	0	0	0.9	0.642	0.922
ADORA1	APP	9606.ENSP00000356205	9606.ENSP00000284981	0	0	0	0	0.139	0	0.9	0.243	0.929
ADORA1	CXCR1	9606.ENSP00000356205	9606.ENSP00000295683	0	0	0	0.58	0	0	0.9	0.136	0.904
ADORA1	ADORA2A	9606.ENSP00000356205	9606.ENSP00000336630	0	0	0	0.93	0.062	0.454	0	0.714	0.492
ADORA1	AKT1	9606.ENSP00000356205	9606.ENSP00000451828	0	0	0	0	0.062	0.072	0	0.447	0.476
ADORA1	CYP1B1	9606.ENSP00000356205	9606.ENSP00000478561	0	0	0	0	0	0	0	0.507	0.507
ADORA1	ADORA3	9606.ENSP00000356205	9606.ENSP00000358730	0	0	0	0.727	0	0	0.9	0.759	0.92
ADORA2A	MPO	9606.ENSP00000336630	9606.ENSP00000225275	0	0	0	0	0	0.056	0	0.398	0.407
ADORA2A	MAOB	9606.ENSP00000336630	9606.ENSP00000367309	0	0	0	0	0	0	0	0.475	0.475

ADORA2A	AKT1	9606.ENSP00000336630	9606.ENSP00000451828	0	0	0	0	0	0.072	0	0.517	0.533
ADORA3	DRD4	9606.ENSP00000358730	9606.ENSP00000176183	0	0	0	0.569	0	0	0.9	0.59	0.924
ADORA3	MMP2	9606.ENSP00000358730	9606.ENSP00000219070	0	0	0	0	0	0.064	0	0.533	0.544
ADORA3	MPO	9606.ENSP00000358730	9606.ENSP00000225275	0	0	0	0	0.098	0.056	0	0.384	0.429
ADORA3	APP	9606.ENSP00000358730	9606.ENSP00000284981	0	0	0	0	0	0	0.9	0.262	0.923
ADORA3	CXCR1	9606.ENSP00000358730	9606.ENSP00000295683	0	0	0	0	0.125	0	0.9	0.139	0.918
ADORA3	AKT1	9606.ENSP00000358730	9606.ENSP00000451828	0	0	0	0	0	0.072	0	0.472	0.489
AHR	IL2	9606.ENSP00000242057	9606.ENSP00000226730	0	0	0	0	0	0	0	0.48	0.48
AHR	NR3C1	9606.ENSP00000242057	9606.ENSP00000231509	0	0	0	0	0.059	0.05	0	0.582	0.594
AHR	AKT1	9606.ENSP00000242057	9606.ENSP00000451828	0	0	0	0	0	0	0	0.447	0.447
AHR	GPR35	9606.ENSP00000242057	9606.ENSP00000411788	0	0	0	0	0	0	0	0.465	0.465
AHR	PPARA	9606.ENSP00000242057	9606.ENSP00000385523	0	0	0	0	0.059	0.05	0	0.484	0.499
AHR	NOS2	9606.ENSP00000242057	9606.ENSP00000327251	0	0	0	0	0	0	0	0.505	0.505

AHR	ESR2	9606.ENSP00000242057	9606.ENSP00000343925	0	0	0	0	0	0.05	0	0.514	0.519
AHR	RORC	9606.ENSP00000242057	9606.ENSP00000327025	0	0	0	0	0	0	0	0.534	0.534
AHR	CYP19A1	9606.ENSP00000242057	9606.ENSP00000379683	0	0	0	0	0	0	0	0.534	0.534
AHR	MMP9	9606.ENSP00000242057	9606.ENSP00000361405	0	0	0	0	0	0	0	0.56	0.56
AHR	SRC	9606.ENSP00000242057	9606.ENSP00000362680	0	0	0	0	0	0	0	0.628	0.628
AHR	EGFR	9606.ENSP00000242057	9606.ENSP00000275493	0	0	0	0	0.061	0	0	0.635	0.643
AHR	PTGS2	9606.ENSP00000242057	9606.ENSP00000356438	0	0	0	0	0.098	0	0	0.671	0.69
AHR	AR	9606.ENSP00000242057	9606.ENSP00000363822	0	0	0	0	0	0.384	0	0.589	0.736
AHR	ESR1	9606.ENSP00000242057	9606.ENSP00000405330	0	0	0	0	0	0.384	0	0.826	0.888
AHR	CYP1B1	9606.ENSP00000242057	9606.ENSP00000478561	0	0	0	0	0.095	0	0	0.939	0.943
AKR1A1	CBR1	9606.ENSP00000361140	9606.ENSP00000290349	0.041	0	0	0	0.063	0	0	0.717	0.724
AKR1A1	GLO1	9606.ENSP00000361140	9606.ENSP00000362463	0	0	0	0	0.177	0.128	0	0.441	0.564
AKR1A1	MAOB	9606.ENSP00000361140	9606.ENSP00000367309	0	0	0	0	0.062	0	0.9	0.223	0.92

AKR1B1	EGFR	9606.ENSP00000285930	9606.ENSP00000275493	0	0	0	0	0.231	0	0	0.285	0.426
AKR1B1	PTGES	9606.ENSP00000285930	9606.ENSP00000342385	0	0	0	0	0	0	0	0.416	0.416
AKR1B1	PTPN1	9606.ENSP00000285930	9606.ENSP00000360683	0	0	0	0	0.061	0	0	0.428	0.439
AKR1B1	PIM1	9606.ENSP00000285930	9606.ENSP00000362608	0	0	0	0	0	0	0	0.473	0.473
AKR1B1	XDH	9606.ENSP00000285930	9606.ENSP00000368727	0	0	0	0	0	0	0	0.498	0.497
AKR1B1	AKT1	9606.ENSP00000285930	9606.ENSP00000451828	0	0	0	0	0	0	0	0.535	0.535
AKR1B1	PTGS2	9606.ENSP00000285930	9606.ENSP00000356438	0	0	0	0	0.062	0	0	0.653	0.661
AKR1B1	CBR1	9606.ENSP00000285930	9606.ENSP00000290349	0.041	0	0	0	0.063	0	0	0.762	0.768
AKR1B1	GLO1	9606.ENSP00000285930	9606.ENSP00000362463	0	0	0	0	0.172	0.42	0	0.634	0.809
AKR1B1	AKR1C3	9606.ENSP00000285930	9606.ENSP00000369927	0	0	0.449	0.939	0	0	0.9	0.63	0.906
AKR1B1	AKR1B10	9606.ENSP00000285930	9606.ENSP00000352584	0	0	0.449	0.975	0.099	0	0.9	0.743	0.908
AKR1B10	CBR1	9606.ENSP00000352584	9606.ENSP00000290349	0.041	0	0	0	0.063	0	0	0.458	0.471
AKR1B10	AKR1C3	9606.ENSP00000352584	9606.ENSP00000369927	0	0	0.449	0.933	0.102	0	0.9	0.669	0.913

AKR1C1	HSD17B2	9606.ENSP00000370254	9606.ENSP00000199936	0	0	0	0	0.062	0	0	0.445	0.457
AKR1C1	CBR1	9606.ENSP00000370254	9606.ENSP00000290349	0.041	0	0	0	0.063	0	0	0.59	0.6
AKR1C1	CYP17A1	9606.ENSP00000370254	9606.ENSP00000358903	0	0	0	0	0.061	0	0.9	0.4	0.938
AKR1C1	AKR1C3	9606.ENSP00000370254	9606.ENSP00000369927	0	0	0.449	0.983	0.214	0.943	0.9	0.836	0.995
AKR1C1	AKR1C2	9606.ENSP00000370254	9606.ENSP00000370129	0	0	0.449	0.986	0.405	0.86	0.9	0.892	0.991
AKR1C1	CYP1B1	9606.ENSP00000370254	9606.ENSP00000478561	0	0	0	0	0.061	0	0	0.44	0.451
AKR1C1	HSD17B1	9606.ENSP00000370254	9606.ENSP00000466799	0.041	0	0	0	0.062	0	0	0.458	0.47
AKR1C2	HSD17B2	9606.ENSP00000370129	9606.ENSP00000199936	0	0	0	0	0.062	0	0	0.428	0.44
AKR1C2	CBR1	9606.ENSP00000370129	9606.ENSP00000290349	0.041	0	0	0	0.063	0	0	0.569	0.579
AKR1C2	CYP17A1	9606.ENSP00000370129	9606.ENSP00000358903	0	0	0	0	0.061	0	0	0.473	0.483
AKR1C2	AR	9606.ENSP00000370129	9606.ENSP00000363822	0	0	0	0	0	0.05	0	0.455	0.46
AKR1C2	AKR1C4	9606.ENSP00000370129	9606.ENSP00000369814	0	0	0.449	0.98	0.066	0.86	0	0.905	0.867
AKR1C2	AKR1C3	9606.ENSP00000370129	9606.ENSP00000369927	0	0	0.449	0.983	0.214	0.912	0.8	0.836	0.985

AKR1C2	HSD17B1	9606.ENSP00000370129	9606.ENSP00000466799	0.041	0	0	0	0.062	0	0	0.485	0.496
AKR1C3	HSD17B2	9606.ENSP00000369927	9606.ENSP00000199936	0	0	0	0	0.062	0	0.8	0.63	0.924
AKR1C3	CBR1	9606.ENSP00000369927	9606.ENSP00000290349	0.041	0	0	0	0.063	0	0.9	0.668	0.966
AKR1C3	PTGES	9606.ENSP00000369927	9606.ENSP00000342385	0	0	0	0	0	0	0	0.709	0.709
AKR1C3	PTGS2	9606.ENSP00000369927	9606.ENSP00000356438	0	0	0	0	0	0	0	0.535	0.535
AKR1C3	CYP17A1	9606.ENSP00000369927	9606.ENSP00000358903	0	0	0	0	0.061	0	0.9	0.669	0.966
AKR1C3	AR	9606.ENSP00000369927	9606.ENSP00000363822	0	0	0	0	0	0.05	0	0.769	0.772
AKR1C3	AKR1C4	9606.ENSP00000369927	9606.ENSP00000369814	0	0	0.449	0.981	0.076	0.933	0	0.81	0.936
AKR1C3	ESR1	9606.ENSP00000369927	9606.ENSP00000405330	0	0	0	0	0	0.05	0	0.42	0.425
AKR1C3	HSD17B1	9606.ENSP00000369927	9606.ENSP00000466799	0.041	0	0	0	0.062	0	0.8	0.663	0.931
AKR1C3	CYP1B1	9606.ENSP00000369927	9606.ENSP00000478561	0	0	0	0	0.061	0	0.9	0.344	0.933
AKR1C3	CYP19A1	9606.ENSP00000369927	9606.ENSP00000379683	0	0	0	0	0.061	0	0.9	0.531	0.952
AKR1C4	CBR1	9606.ENSP00000369814	9606.ENSP00000290349	0.041	0	0	0	0.063	0	0	0.594	0.603

AKR1C4	CYP17A1	9606.ENSP00000369814	9606.ENSP00000358903	0	0	0	0	0.061	0	0	0.398	0.41
AKR1C4	HSD17B1	9606.ENSP00000369814	9606.ENSP00000466799	0.041	0	0	0	0.062	0	0	0.439	0.451
AKT1	DRD4	9606.ENSP00000451828	9606.ENSP00000176183	0	0	0	0	0	0.072	0	0.4	0.419
AKT1	CSNK2A1	9606.ENSP00000451828	9606.ENSP00000217244	0	0	0.223	0.581	0.057	0.406	0	0.377	0.541
AKT1	MMP2	9606.ENSP00000451828	9606.ENSP00000219070	0	0	0	0	0.061	0	0	0.833	0.836
AKT1	MPO	9606.ENSP00000451828	9606.ENSP00000225275	0	0	0	0	0	0.058	0	0.559	0.567
AKT1	CD38	9606.ENSP00000451828	9606.ENSP00000226279	0	0	0	0	0	0.062	0	0.421	0.433
AKT1	IL2	9606.ENSP00000451828	9606.ENSP00000226730	0	0	0	0	0	0	0.9	0.793	0.978
AKT1	NR3C1	9606.ENSP00000451828	9606.ENSP00000231509	0	0	0	0	0	0.407	0	0.561	0.728
AKT1	ODC1	9606.ENSP00000451828	9606.ENSP00000234111	0	0	0	0	0.059	0.077	0	0.435	0.466
AKT1	CTSD	9606.ENSP00000451828	9606.ENSP00000236671	0	0	0	0	0.095	0.085	0	0.423	0.481
AKT1	FLT3	9606.ENSP00000451828	9606.ENSP00000241453	0	0	0	0	0	0.096	0	0.669	0.687
AKT1	CCNB1	9606.ENSP00000451828	9606.ENSP00000256442	0	0	0	0	0	0.138	0	0.804	0.824

AKT1	MMP13	9606.ENSP00000451828	9606.ENSP00000260302	0	0	0	0	0	0	0	0.506	0.506
AKT1	NOX4	9606.ENSP00000451828	9606.ENSP00000263317	0	0	0	0	0	0.079	0	0.802	0.809
AKT1	TYR	9606.ENSP00000451828	9606.ENSP00000263321	0	0	0	0	0	0	0	0.485	0.485
AKT1	KDR	9606.ENSP00000451828	9606.ENSP00000263923	0	0	0	0.555	0	0.096	0	0.905	0.458
AKT1	CDK6	9606.ENSP00000451828	9606.ENSP00000265734	0	0	0.389	0.604	0.062	0.185	0	0.694	0.502
AKT1	CDK2	9606.ENSP00000451828	9606.ENSP00000266970	0	0	0.394	0.655	0.062	0.093	0	0.873	0.457
AKT1	IGF1R	9606.ENSP00000451828	9606.ENSP00000268035	0	0	0	0.554	0.052	0.179	0	0.955	0.533
AKT1	EGFR	9606.ENSP00000451828	9606.ENSP00000275493	0	0	0	0.554	0	0.431	0	0.973	0.678
AKT1	APP	9606.ENSP00000451828	9606.ENSP00000284981	0	0	0	0	0.063	0.07	0	0.62	0.64
AKT1	HMGCR	9606.ENSP00000451828	9606.ENSP00000287936	0	0	0	0	0	0.052	0	0.521	0.526
AKT1	CXCR1	9606.ENSP00000451828	9606.ENSP00000295683	0	0	0	0	0	0.072	0	0.491	0.508
AKT1	MMP3	9606.ENSP00000451828	9606.ENSP00000299855	0	0	0	0	0	0	0	0.585	0.585
AKT1	INSR	9606.ENSP00000451828	9606.ENSP00000303830	0	0	0	0.559	0.052	0.288	0.9	0.677	0.948

AKT1	F2	9606.ENSP00000451828	9606.ENSP00000308541	0	0	0	0	0	0.05	0	0.662	0.665
AKT1	PLA2G1B	9606.ENSP00000451828	9606.ENSP00000312286	0	0	0	0	0	0	0	0.44	0.44
AKT1	TYMS	9606.ENSP00000451828	9606.ENSP00000315644	0	0	0	0	0.064	0	0	0.419	0.432
AKT1	BACE1	9606.ENSP00000451828	9606.ENSP00000318585	0	0	0	0	0.061	0.085	0	0.477	0.512
AKT1	GSK3B	9606.ENSP00000451828	9606.ENSP00000324806	0	0	0.218	0.576	0.049	0.995	0.9	0.918	0.999
AKT1	NOS2	9606.ENSP00000451828	9606.ENSP00000327251	0	0	0	0	0.063	0.065	0.8	0.543	0.909
AKT1	MAPT	9606.ENSP00000451828	9606.ENSP00000340820	0	0	0	0	0	0.336	0	0.717	0.804
AKT1	PTK2	9606.ENSP00000451828	9606.ENSP00000341189	0	0	0	0.568	0.082	0.399	0	0.816	0.626
AKT1	PKN1	9606.ENSP00000451828	9606.ENSP00000343325	0	0	0	0.871	0.118	0.454	0	0.382	0.521
AKT1	ESR2	9606.ENSP00000451828	9606.ENSP00000343925	0	0	0	0	0	0.407	0	0.722	0.828
AKT1	ARG1	9606.ENSP00000451828	9606.ENSP00000349446	0	0	0	0	0.062	0	0	0.412	0.425
AKT1	PIK3CG	9606.ENSP00000451828	9606.ENSP00000352121	0	0	0	0	0.063	0.174	0.9	0.771	0.979
AKT1	TOP1	9606.ENSP00000451828	9606.ENSP00000354522	0	0	0	0	0	0	0	0.452	0.452

AKT1	PARP1	9606.ENSP00000451828	9606.ENSP00000355759	0	0	0	0	0.061	0.057	0	0.783	0.791
AKT1	PTGS2	9606.ENSP00000451828	9606.ENSP00000356438	0	0	0	0	0.062	0.058	0	0.848	0.854
AKT1	PTPN1	9606.ENSP00000451828	9606.ENSP00000360683	0	0	0	0	0.09	0.397	0.9	0.796	0.987
AKT1	MMP9	9606.ENSP00000451828	9606.ENSP00000361405	0	0	0	0	0.061	0	0	0.885	0.887
AKT1	SRC	9606.ENSP00000451828	9606.ENSP00000362680	0	0	0.391	0.562	0.097	0.546	0.9	0.96	0.978
AKT1	ALOX5	9606.ENSP00000451828	9606.ENSP00000363512	0	0	0	0	0	0	0	0.429	0.429
AKT1	AR	9606.ENSP00000451828	9606.ENSP00000363822	0	0	0	0	0	0.407	0	0.89	0.932
AKT1	CA9	9606.ENSP00000451828	9606.ENSP00000367608	0	0	0	0	0	0	0	0.475	0.475
AKT1	CDK1	9606.ENSP00000451828	9606.ENSP00000378699	0	0	0.355	0.639	0.062	0.093	0	0.735	0.422
AKT1	CYP19A1	9606.ENSP00000451828	9606.ENSP00000379683	0	0	0	0	0	0.055	0	0.63	0.635
AKT1	PPARA	9606.ENSP00000451828	9606.ENSP00000385523	0	0	0	0	0	0.085	0	0.471	0.495
AKT1	ESR1	9606.ENSP00000451828	9606.ENSP00000405330	0	0	0	0	0.055	0.407	0.9	0.867	0.991
AKT1	CDK5	9606.ENSP00000451828	9606.ENSP00000419782	0	0	0.352	0.633	0	0.068	0	0.761	0.405

AKT1	PIK3R1	9606.ENSP00000451828	9606.ENSP00000428056	0	0	0	0	0.062	0.447	0.9	0.835	0.99
ALK	IGF1R	9606.ENSP00000373700	9606.ENSP00000268035	0	0	0	0.607	0	0.389	0	0.61	0.531
ALK	EGFR	9606.ENSP00000373700	9606.ENSP00000275493	0	0	0	0.562	0.049	0.172	0	0.906	0.503
ALK	INSR	9606.ENSP00000373700	9606.ENSP00000303830	0	0	0	0.597	0	0.389	0	0.384	0.477
ALK	TYMS	9606.ENSP00000373700	9606.ENSP00000315644	0	0	0	0	0.055	0	0	0.421	0.429
ALK	SRC	9606.ENSP00000373700	9606.ENSP00000362680	0	0	0	0.696	0.063	0.439	0	0.605	0.55
ALK	ESR1	9606.ENSP00000373700	9606.ENSP00000405330	0	0	0	0	0	0.085	0	0.441	0.466
ALK	PIK3R1	9606.ENSP00000373700	9606.ENSP00000428056	0	0	0	0	0	0.372	0.6	0.304	0.81
ALOX12	PTGES	9606.ENSP00000251535	9606.ENSP00000342385	0	0	0	0	0	0	0	0.43	0.43
ALOX12	MMP9	9606.ENSP00000251535	9606.ENSP00000361405	0	0	0	0	0	0	0	0.506	0.506
ALOX12	PLA2G1B	9606.ENSP00000251535	9606.ENSP00000312286	0	0	0	0	0	0.294	0.65	0.511	0.868
ALOX12	ALOX15	9606.ENSP00000251535	9606.ENSP00000458832	0	0	0.407	0.97	0.061	0	0.9	0.709	0.905
ALOX12	ALOX5	9606.ENSP00000251535	9606.ENSP00000363512	0	0	0.386	0.911	0.063	0	0.9	0.863	0.912

ALOX12	PTGS2	9606.ENSP00000251535	9606.ENSP00000356438	0	0	0	0	0.061	0	0.9	0.526	0.951
ALOX15	PLA2G1B	9606.ENSP00000458832	9606.ENSP00000312286	0	0	0	0	0	0	0.65	0.52	0.824
ALOX15	PTGES	9606.ENSP00000458832	9606.ENSP00000342385	0	0	0	0	0	0	0	0.504	0.504
ALOX15	PTGS2	9606.ENSP00000458832	9606.ENSP00000356438	0	0	0	0	0.061	0	0.9	0.601	0.959
ALOX15	MMP9	9606.ENSP00000458832	9606.ENSP00000361405	0	0	0	0	0.061	0	0	0.561	0.57
ALOX15	ALOX5	9606.ENSP00000458832	9606.ENSP00000363512	0	0	0.298	0.905	0.062	0	0.9	0.891	0.912
ALOX5	MMP2	9606.ENSP00000363512	9606.ENSP00000219070	0	0	0	0	0	0	0	0.501	0.501
ALOX5	MPO	9606.ENSP00000363512	9606.ENSP00000225275	0	0	0	0	0.12	0	0	0.543	0.581
ALOX5	PLA2G1B	9606.ENSP00000363512	9606.ENSP00000312286	0	0	0	0	0	0	0.65	0.646	0.87
ALOX5	PTGES	9606.ENSP00000363512	9606.ENSP00000342385	0	0	0	0	0	0	0	0.67	0.67
ALOX5	PTGS2	9606.ENSP00000363512	9606.ENSP00000356438	0	0	0	0	0.08	0	0.9	0.758	0.975
ALOX5	MMP9	9606.ENSP00000363512	9606.ENSP00000361405	0	0	0	0	0.159	0	0	0.423	0.494
ALOX5	SYK	9606.ENSP00000363512	9606.ENSP00000364907	0	0	0	0	0.253	0	0	0.245	0.411

ALOX5	HSD17B1	9606.ENSP00000363512	9606.ENSP00000466799	0	0	0	0	0	0.426	0	0	0.426
APEX1	SAE1	9606.ENSP00000216714	9606.ENSP00000270225	0.043	0	0	0	0.298	0	0	0.186	0.406
APEX1	TYMS	9606.ENSP00000216714	9606.ENSP00000315644	0.075	0	0	0	0.191	0	0	0.363	0.482
APEX1	PTGS2	9606.ENSP00000216714	9606.ENSP00000356438	0	0	0	0	0	0	0	0.492	0.492
APEX1	GLO1	9606.ENSP00000216714	9606.ENSP00000362463	0.043	0	0	0	0.152	0	0	0.47	0.532
APEX1	TOP2A	9606.ENSP00000216714	9606.ENSP00000411532	0.129	0	0	0	0.098	0	0	0.489	0.564
APEX1	TOP1	9606.ENSP00000216714	9606.ENSP00000354522	0	0	0	0	0.08	0	0	0.549	0.568
APEX1	CDK5	9606.ENSP00000216714	9606.ENSP00000419782	0	0	0	0	0	0	0	0.581	0.581
APEX1	CSNK2A1	9606.ENSP00000216714	9606.ENSP00000217244	0	0	0	0	0.107	0.379	0	0.572	0.742
APEX1	MPG	9606.ENSP00000216714	9606.ENSP00000219431	0.048	0	0	0	0	0	0.9	0.604	0.959
APEX1	PARP1	9606.ENSP00000216714	9606.ENSP00000355759	0	0	0	0	0.118	0	0.9	0.8	0.98
APEX1	POLB	9606.ENSP00000216714	9606.ENSP00000265421	0	0	0	0	0.062	0.379	0.9	0.799	0.986
APP	DRD4	9606.ENSP00000284981	9606.ENSP00000176183	0	0	0	0	0	0	0.9	0.251	0.921

APP	ODC1	9606.ENSP00000284981	9606.ENSP00000234111	0	0	0	0	0	0	0	0.434	0.434
APP	CTSD	9606.ENSP00000284981	9606.ENSP00000236671	0	0	0	0	0.086	0.406	0	0.562	0.742
APP	TTR	9606.ENSP00000284981	9606.ENSP00000237014	0	0	0	0	0.063	0.379	0.9	0.546	0.97
APP	KDR	9606.ENSP00000284981	9606.ENSP00000263923	0	0	0	0	0.076	0.472	0	0.198	0.574
APP	BCHE	9606.ENSP00000284981	9606.ENSP00000264381	0	0	0	0	0.086	0	0	0.694	0.709
APP	EGFR	9606.ENSP00000284981	9606.ENSP00000275493	0	0	0	0	0.146	0.393	0	0.503	0.72
APP	ESR1	9606.ENSP00000284981	9606.ENSP00000405330	0	0	0	0	0	0	0	0.44	0.44
APP	MAOB	9606.ENSP00000284981	9606.ENSP00000367309	0	0	0	0	0.083	0	0	0.435	0.46
APP	MMP9	9606.ENSP00000284981	9606.ENSP00000361405	0	0	0	0	0.06	0	0	0.474	0.485
APP	HMGCR	9606.ENSP00000284981	9606.ENSP00000287936	0	0	0	0	0	0	0	0.576	0.577
APP	PTGS2	9606.ENSP00000284981	9606.ENSP00000356438	0	0	0	0	0.061	0.379	0	0.454	0.654
APP	SRC	9606.ENSP00000284981	9606.ENSP00000362680	0	0	0	0	0.088	0.397	0	0.464	0.68
APP	GSK3B	9606.ENSP00000284981	9606.ENSP00000324806	0	0	0	0	0.057	0.397	0	0.748	0.845

APP	CDK5	9606.ENSP00000284981	9606.ENSP00000419782	0	0	0	0	0.061	0.379	0	0.842	0.9
APP	CXCR1	9606.ENSP00000284981	9606.ENSP00000295683	0	0	0	0	0	0	0.9	0.064	0.902
APP	PIK3R1	9606.ENSP00000284981	9606.ENSP00000428056	0	0	0	0	0	0	0.9	0.19	0.915
APP	F2	9606.ENSP00000284981	9606.ENSP00000308541	0	0	0	0	0	0.132	0.9	0.413	0.944
APP	MAPT	9606.ENSP00000284981	9606.ENSP00000340820	0	0	0	0	0.064	0.379	0	0.919	0.949
APP	BACE1	9606.ENSP00000284981	9606.ENSP00000318585	0	0	0	0	0.089	0.406	0.9	0.958	0.997
AR	HSD17B2	9606.ENSP00000363822	9606.ENSP00000199936	0	0	0	0	0	0.05	0	0.423	0.428
AR	MMP2	9606.ENSP00000363822	9606.ENSP00000219070	0	0	0	0	0.061	0	0	0.4	0.412
AR	NR3C1	9606.ENSP00000363822	9606.ENSP00000231509	0	0	0	0.816	0	0.379	0.9	0.806	0.944
AR	ODC1	9606.ENSP00000363822	9606.ENSP00000234111	0	0	0	0	0	0	0	0.423	0.422
AR	CCNB1	9606.ENSP00000363822	9606.ENSP00000256442	0	0	0	0	0	0.05	0	0.408	0.413
AR	KDR	9606.ENSP00000363822	9606.ENSP00000263923	0	0	0	0	0	0.051	0	0.414	0.42
AR	CDK6	9606.ENSP00000363822	9606.ENSP00000265734	0	0	0	0	0	0.524	0.9	0.399	0.968

AR	CDK2	9606.ENSP00000363822	9606.ENSP00000266970	0	0	0	0	0	0	0	0.535	0.535
AR	IGF1R	9606.ENSP00000363822	9606.ENSP00000268035	0	0	0	0	0.051	0.085	0	0.77	0.783
AR	EGFR	9606.ENSP00000363822	9606.ENSP00000275493	0	0	0	0	0.062	0.402	0	0.678	0.803
AR	CA2	9606.ENSP00000363822	9606.ENSP00000285379	0	0	0	0	0	0.048	0	0.497	0.5
AR	INSR	9606.ENSP00000363822	9606.ENSP00000303830	0	0	0	0	0.061	0.085	0	0.522	0.553
AR	MET	9606.ENSP00000363822	9606.ENSP00000317272	0	0	0	0	0	0.085	0	0.738	0.75
AR	GSK3B	9606.ENSP00000363822	9606.ENSP00000324806	0	0	0	0	0	0.407	0.9	0.49	0.967
AR	PKN1	9606.ENSP00000363822	9606.ENSP00000343325	0	0	0	0	0	0	0.9	0.664	0.965
AR	PARP1	9606.ENSP00000363822	9606.ENSP00000355759	0	0	0	0	0	0	0	0.445	0.445
AR	PTGS2	9606.ENSP00000363822	9606.ENSP00000356438	0	0	0	0	0	0.05	0	0.437	0.442
AR	CYP17A1	9606.ENSP00000363822	9606.ENSP00000358903	0	0	0	0	0	0.085	0	0.797	0.807
AR	MMP9	9606.ENSP00000363822	9606.ENSP00000361405	0	0	0	0	0	0	0	0.716	0.717
AR	PIM1	9606.ENSP00000363822	9606.ENSP00000362608	0	0	0	0	0	0	0	0.595	0.595

AR	SRC	9606.ENSP00000363822	9606.ENSP00000362680	0	0	0	0	0	0.407	0.9	0.857	0.99
AR	HSD17B1	9606.ENSP00000363822	9606.ENSP00000466799	0	0	0	0	0	0	0	0.499	0.499
AR	CDK5	9606.ENSP00000363822	9606.ENSP00000419782	0	0	0	0	0	0	0	0.561	0.562
AR	ESR1	9606.ENSP00000363822	9606.ENSP00000405330	0	0	0	0.656	0.062	0.379	0	0.936	0.587
AR	SHBG	9606.ENSP00000363822	9606.ENSP00000369816	0	0	0	0	0	0	0	0.742	0.742
AR	CDK1	9606.ENSP00000363822	9606.ENSP00000378699	0	0	0	0	0	0.379	0	0.734	0.827
AR	CYP19A1	9606.ENSP00000363822	9606.ENSP00000379683	0	0	0	0	0	0.085	0	0.873	0.879
AR	PIK3R1	9606.ENSP00000363822	9606.ENSP00000428056	0	0	0	0	0	0.153	0.9	0.517	0.955
ARG1	MPO	9606.ENSP00000349446	9606.ENSP00000225275	0	0	0	0	0.102	0	0.9	0.39	0.94
ARG1	IL2	9606.ENSP00000349446	9606.ENSP00000226730	0	0	0	0	0	0	0	0.48	0.48
ARG1	ODC1	9606.ENSP00000349446	9606.ENSP00000234111	0.11	0	0	0	0.064	0	0.9	0.737	0.975
ARG1	CTSD	9606.ENSP00000349446	9606.ENSP00000236671	0	0	0	0	0.062	0	0.9	0.096	0.907
ARG1	TTR	9606.ENSP00000349446	9606.ENSP00000237014	0	0	0	0	0.064	0	0.9	0.083	0.906

ARG1	MMP3	9606.ENSP00000349446	9606.ENSP00000299855	0	0	0	0	0	0	0	0.428	0.428
ARG1	NOS2	9606.ENSP00000349446	9606.ENSP00000327251	0	0	0	0	0.06	0.086	0.9	0.782	0.978
ARG1	MMP12	9606.ENSP00000349446	9606.ENSP00000458585	0	0	0	0	0	0	0	0.483	0.483
ARG1	MMP9	9606.ENSP00000349446	9606.ENSP00000361405	0	0	0	0	0	0	0	0.541	0.541
ARG1	PTGS2	9606.ENSP00000349446	9606.ENSP00000356438	0	0	0	0	0	0	0	0.705	0.705
AURKB	FLT3	9606.ENSP00000313950	9606.ENSP00000241453	0	0	0	0	0	0	0	0.419	0.418
AURKB	CCNB1	9606.ENSP00000313950	9606.ENSP00000256442	0	0	0	0	0.933	0.291	0.9	0.797	0.998
AURKB	CDK6	9606.ENSP00000313950	9606.ENSP00000265734	0	0	0.422	0.669	0.204	0.157	0	0.491	0.487
AURKB	CDK2	9606.ENSP00000313950	9606.ENSP00000266970	0	0	0.438	0.724	0.433	0.184	0	0.635	0.646
AURKB	CCNB2	9606.ENSP00000313950	9606.ENSP00000288207	0	0	0	0	0.962	0.291	0.9	0.677	0.999
AURKB	PLK1	9606.ENSP00000313950	9606.ENSP00000300093	0	0	0	0.751	0.947	0.365	0.9	0.907	0.997
AURKB	NEK6	9606.ENSP00000313950	9606.ENSP00000362702	0	0	0.42	0.665	0.062	0.185	0	0.439	0.404
AURKB	PARP1	9606.ENSP00000313950	9606.ENSP00000355759	0	0	0	0	0.134	0.299	0	0.52	0.683

AURKB	CCNB3	9606.ENSP00000313950	9606.ENSP00000365210	0	0	0	0	0.729	0.177	0	0.321	0.835
AURKB	MYLK	9606.ENSP00000313950	9606.ENSP00000353452	0	0	0	0.739	0	0	0.9	0.161	0.903
AURKB	NEK2	9606.ENSP00000313950	9606.ENSP00000355966	0	0	0.388	0.695	0.895	0	0	0.676	0.926
AURKB	TYMS	9606.ENSP00000313950	9606.ENSP00000315644	0	0	0	0	0.914	0	0	0.23	0.931
AURKB	TOP2A	9606.ENSP00000313950	9606.ENSP00000411532	0	0	0	0	0.941	0.261	0	0.565	0.979
AURKB	CDK1	9606.ENSP00000313950	9606.ENSP00000378699	0	0	0.422	0.726	0.963	0.399	0.9	0.852	0.998
AVPR2	CFTR	9606.ENSP00000351805	9606.ENSP00000003084	0	0	0	0	0	0	0.9	0.421	0.939
AVPR2	DRD4	9606.ENSP00000351805	9606.ENSP00000176183	0	0	0	0	0	0	0	0.421	0.421
AVPR2	EGFR	9606.ENSP00000351805	9606.ENSP00000275493	0	0	0	0	0	0.084	0.9	0.287	0.928
AXL	MMP2	9606.ENSP00000301178	9606.ENSP00000219070	0	0	0	0	0.154	0	0	0.53	0.586
AXL	KDR	9606.ENSP00000301178	9606.ENSP00000263923	0	0	0	0.602	0.061	0.096	0.9	0.587	0.928
AXL	EGFR	9606.ENSP00000301178	9606.ENSP00000275493	0	0	0	0.604	0.162	0.418	0	0.819	0.655
AXL	SRC	9606.ENSP00000301178	9606.ENSP00000362680	0	0	0	0.698	0.061	0.379	0.8	0.528	0.892

AXL	PIK3R1	9606.ENSP00000301178	9606.ENSP00000428056	0	0	0	0	0	0.405	0.9	0.157	0.945
BACE1	MMP2	9606.ENSP00000318585	9606.ENSP00000219070	0	0	0	0	0.051	0.518	0	0.189	0.596
BACE1	BCHE	9606.ENSP00000318585	9606.ENSP00000264381	0	0	0	0	0.063	0	0	0.592	0.601
BACE1	EGFR	9606.ENSP00000318585	9606.ENSP00000275493	0	0	0	0	0.069	0	0	0.43	0.447
BACE1	SYK	9606.ENSP00000318585	9606.ENSP00000364907	0	0	0	0	0	0.085	0	0.421	0.448
BACE1	GSK3B	9606.ENSP00000318585	9606.ENSP00000324806	0	0	0	0	0.059	0.085	0	0.44	0.476
BACE1	MAPT	9606.ENSP00000318585	9606.ENSP00000340820	0	0	0	0	0.07	0	0	0.684	0.693
BACE1	CDK5	9606.ENSP00000318585	9606.ENSP00000419782	0	0	0	0	0	0	0	0.788	0.788
BCHE	TTR	9606.ENSP00000264381	9606.ENSP00000237014	0	0	0	0	0.085	0	0	0.528	0.549
BCHE	TYR	9606.ENSP00000264381	9606.ENSP00000263321	0	0	0	0	0.165	0	0	0.491	0.556
BCHE	MAOA	9606.ENSP00000264381	9606.ENSP00000340684	0.045	0	0	0	0.061	0	0	0.603	0.613
BCHE	F2	9606.ENSP00000264381	9606.ENSP00000308541	0	0	0	0	0.126	0.052	0	0.669	0.701
BCHE	MAOB	9606.ENSP00000264381	9606.ENSP00000367309	0.045	0	0	0	0.086	0	0	0.694	0.71

BCHE	MAPT	9606.ENSP00000264381	9606.ENSP00000340820	0	0	0	0	0.061	0.379	0	0.555	0.717
CA2	CFTR	9606.ENSP00000285379	9606.ENSP00000003084	0	0	0	0	0	0	0	0.451	0.451
CA2	MMP9	9606.ENSP00000285379	9606.ENSP00000361405	0	0	0	0	0	0	0	0.414	0.414
CA3	SRC	9606.ENSP00000285381	9606.ENSP00000362680	0	0	0	0	0	0.379	0	0.132	0.438
CA4	MAPT	9606.ENSP00000300900	9606.ENSP00000340820	0	0	0	0	0.082	0	0	0.416	0.441
CA7	PTGS2	9606.ENSP00000345659	9606.ENSP00000356438	0	0	0	0	0	0	0	0.718	0.718
CA9	MMP2	9606.ENSP00000367608	9606.ENSP00000219070	0	0	0	0	0.07	0	0	0.399	0.417
CA9	IL2	9606.ENSP00000367608	9606.ENSP00000226730	0	0	0	0	0	0	0	0.445	0.445
CA9	KDR	9606.ENSP00000367608	9606.ENSP00000263923	0	0	0	0	0	0	0	0.525	0.525
CA9	EGFR	9606.ENSP00000367608	9606.ENSP00000275493	0	0	0	0	0.109	0	0	0.732	0.751
CA9	MET	9606.ENSP00000367608	9606.ENSP00000317272	0	0	0	0	0.076	0	0	0.421	0.442
CA9	PTGS2	9606.ENSP00000367608	9606.ENSP00000356438	0	0	0	0	0	0	0	0.598	0.598
CA9	MMP9	9606.ENSP00000367608	9606.ENSP00000361405	0	0	0	0	0	0	0	0.68	0.68

CA9	SRC	9606.ENSP00000367608	9606.ENSP00000362680	0	0	0	0	0	0	0	0.541	0.541
CA9	ESR1	9606.ENSP00000367608	9606.ENSP00000405330	0	0	0	0	0	0.048	0	0.401	0.405
CAMK2B	TYR	9606.ENSP00000379098	9606.ENSP00000263321	0	0	0	0	0	0.269	0	0.214	0.4
CAMK2B	MAPT	9606.ENSP00000379098	9606.ENSP00000340820	0	0	0	0	0.252	0.064	0.9	0.212	0.937
CAMK2B	MYLK	9606.ENSP00000379098	9606.ENSP00000353452	0	0	0	0.649	0.061	0	0.9	0.368	0.913
CAMK2B	SRC	9606.ENSP00000379098	9606.ENSP00000362680	0	0	0	0.557	0.061	0.123	0.9	0.191	0.916
CBR1	PTGES	9606.ENSP00000290349	9606.ENSP00000342385	0	0	0	0	0	0	0.9	0.456	0.943
CCNB1	MMP2	9606.ENSP00000256442	9606.ENSP00000219070	0	0	0	0	0	0	0	0.65	0.65
CCNB1	SRC	9606.ENSP00000256442	9606.ENSP00000362680	0	0	0	0	0.053	0.09	0	0.361	0.401
CCNB1	PTGS2	9606.ENSP00000256442	9606.ENSP00000356438	0	0	0	0	0.061	0	0	0.398	0.41
CCNB1	IGF1R	9606.ENSP00000256442	9606.ENSP00000268035	0	0	0	0	0.062	0.09	0	0.369	0.414
CCNB1	MMP9	9606.ENSP00000256442	9606.ENSP00000361405	0	0	0	0	0	0	0	0.485	0.485
CCNB1	TNKS	9606.ENSP00000256442	9606.ENSP00000311579	0	0	0	0	0	0.26	0	0.394	0.533

CCNB1	ESR1	9606.ENSP00000256442	9606.ENSP00000405330	0	0	0	0	0	0.05	0	0.53	0.534
CCNB1	PARP1	9606.ENSP00000256442	9606.ENSP00000355759	0	0	0	0	0.201	0	0	0.458	0.549
CCNB1	EGFR	9606.ENSP00000256442	9606.ENSP00000275493	0	0	0	0	0	0.085	0	0.722	0.735
CCNB1	TYMS	9606.ENSP00000256442	9606.ENSP00000315644	0	0	0	0	0.725	0	0	0.405	0.829
CCNB1	NEK2	9606.ENSP00000256442	9606.ENSP00000355966	0	0	0	0	0.899	0	0	0.571	0.955
CCNB1	CDK5	9606.ENSP00000256442	9606.ENSP00000419782	0	0	0	0	0	0.267	0.9	0.529	0.962
CCNB1	CDK6	9606.ENSP00000256442	9606.ENSP00000265734	0	0	0	0	0.152	0.265	0.9	0.793	0.985
CCNB1	TOP2A	9606.ENSP00000256442	9606.ENSP00000411532	0	0	0	0	0.964	0.057	0	0.622	0.986
CCNB1	CCNB3	9606.ENSP00000256442	9606.ENSP00000365210	0	0	0	0.752	0.81	0.736	0.8	0.771	0.991
CCNB1	CDK1	9606.ENSP00000256442	9606.ENSP00000378699	0	0	0	0	0.969	0.999	0.9	0.983	0.999
CCNB1	CCNB2	9606.ENSP00000256442	9606.ENSP00000288207	0	0	0	0.943	0.955	0.945	0.9	0.839	0.999
CCNB1	CDK2	9606.ENSP00000256442	9606.ENSP00000266970	0	0	0	0	0.433	0.974	0.9	0.856	0.999
CCNB1	PLK1	9606.ENSP00000256442	9606.ENSP00000300093	0	0	0	0	0.936	0.772	0.9	0.942	0.999

CCNB2	CDK6	9606.ENSP00000288207	9606.ENSP00000265734	0	0	0	0	0.154	0.265	0.9	0.65	0.975
CCNB2	CDK2	9606.ENSP00000288207	9606.ENSP00000266970	0	0	0	0	0.459	0.995	0.9	0.689	0.999
CCNB2	TYMS	9606.ENSP00000288207	9606.ENSP00000315644	0	0	0	0	0.834	0	0	0.273	0.874
CCNB2	CDK5	9606.ENSP00000288207	9606.ENSP00000419782	0	0	0	0	0	0.064	0.9	0.327	0.931
CCNB2	NEK2	9606.ENSP00000288207	9606.ENSP00000355966	0	0	0	0	0.955	0	0	0.564	0.979
CCNB2	TOP2A	9606.ENSP00000288207	9606.ENSP00000411532	0	0	0	0	0.965	0.057	0	0.623	0.986
CCNB2	CCNB3	9606.ENSP00000288207	9606.ENSP00000365210	0	0	0	0.75	0.81	0.736	0.8	0.784	0.991
CCNB2	PLK1	9606.ENSP00000288207	9606.ENSP00000300093	0	0	0	0	0.937	0.303	0.9	0.779	0.998
CCNB2	CDK1	9606.ENSP00000288207	9606.ENSP00000378699	0	0	0	0	0.993	0.966	0.9	0.853	0.999
CCNB3	CDK6	9606.ENSP00000365210	9606.ENSP00000265734	0	0	0	0	0.111	0.265	0	0.448	0.608
CCNB3	CDK2	9606.ENSP00000365210	9606.ENSP00000266970	0	0	0	0	0.273	0.595	0	0.889	0.964
CCNB3	PLK1	9606.ENSP00000365210	9606.ENSP00000300093	0	0	0	0	0.917	0.175	0.8	0.427	0.991
CCNB3	CDK1	9606.ENSP00000365210	9606.ENSP00000378699	0	0	0	0	0.917	0.795	0.8	0.738	0.998

CD38	MPO	9606.ENSP00000226279	9606.ENSP00000225275	0	0	0	0	0.069	0	0	0.511	0.525
CD38	SYK	9606.ENSP00000226279	9606.ENSP00000364907	0	0	0	0	0.095	0.062	0	0.402	0.448
CD38	EGFR	9606.ENSP00000226279	9606.ENSP00000275493	0	0	0	0	0	0	0	0.495	0.495
CD38	FLT3	9606.ENSP00000226279	9606.ENSP00000241453	0	0	0	0	0.061	0	0	0.637	0.645
CD38	IL2	9606.ENSP00000226279	9606.ENSP00000226730	0	0	0	0	0	0	0	0.669	0.669
CDK1	CSNK2A1	9606.ENSP00000378699	9606.ENSP00000217244	0	0	0.338	0.774	0.098	0.397	0	0.431	0.521
CDK1	UBA2	9606.ENSP00000378699	9606.ENSP00000246548	0	0	0	0	0.158	0.082	0	0.332	0.439
CDK1	CDK6	9606.ENSP00000378699	9606.ENSP00000265734	0	0	0.425	0.923	0.154	0.185	0.9	0.864	0.932
CDK1	CDK2	9606.ENSP00000378699	9606.ENSP00000266970	0	0	0.441	0.968	0.684	0.819	0.9	0.935	0.994
CDK1	SAE1	9606.ENSP00000378699	9606.ENSP00000270225	0	0	0	0	0.539	0.082	0	0.236	0.648
CDK1	EGFR	9606.ENSP00000378699	9606.ENSP00000275493	0	0	0	0.62	0	0.544	0	0.557	0.637
CDK1	PLK1	9606.ENSP00000378699	9606.ENSP00000300093	0	0	0	0.627	0.963	0.267	0.9	0.961	0.998
CDK1	TNKS	9606.ENSP00000378699	9606.ENSP00000311579	0	0	0	0	0.062	0.186	0	0.361	0.469

CDK1	TYMS	9606.ENSP00000378699	9606.ENSP00000315644	0	0	0	0	0.981	0	0	0.318	0.986
CDK1	MAPT	9606.ENSP00000378699	9606.ENSP00000340820	0	0	0	0	0	0.379	0	0.271	0.528
CDK1	PKN1	9606.ENSP00000378699	9606.ENSP00000343325	0	0	0	0.626	0.048	0.079	0.9	0.174	0.909
CDK1	TOP1	9606.ENSP00000378699	9606.ENSP00000354522	0	0	0	0	0.09	0	0	0.48	0.507
CDK1	PARP1	9606.ENSP00000378699	9606.ENSP00000355759	0	0	0	0	0.187	0	0	0.515	0.589
CDK1	NEK2	9606.ENSP00000378699	9606.ENSP00000355966	0	0	0.345	0.74	0.922	0	0.9	0.597	0.993
CDK1	PTPN1	9606.ENSP00000378699	9606.ENSP00000360683	0	0	0	0	0.062	0.467	0	0.553	0.757
CDK1	SRC	9606.ENSP00000378699	9606.ENSP00000362680	0	0	0.259	0.648	0.062	0.093	0.9	0.75	0.937
CDK1	ESR1	9606.ENSP00000378699	9606.ENSP00000405330	0	0	0	0	0	0	0	0.459	0.459
CDK1	CDK5	9606.ENSP00000378699	9606.ENSP00000419782	0	0	0.424	0.954	0	0.064	0.9	0.726	0.907
CDK1	TOP2A	9606.ENSP00000378699	9606.ENSP00000411532	0	0	0	0	0.986	0.386	0	0.597	0.996
CDK2	MMP2	9606.ENSP00000266970	9606.ENSP00000219070	0	0	0	0	0	0	0	0.442	0.442
CDK2	CDK6	9606.ENSP00000266970	9606.ENSP00000265734	0	0	0.439	0.927	0.108	0.711	0.9	0.908	0.974

CDK2	MAPT	9606.ENSP00000266970	9606.ENSP00000340820	0	0	0	0	0	0.313	0	0.265	0.473
CDK2	MMP9	9606.ENSP00000266970	9606.ENSP00000361405	0	0	0	0	0	0	0	0.49	0.49
CDK2	PARP1	9606.ENSP00000266970	9606.ENSP00000355759	0	0	0	0	0.093	0.066	0	0.446	0.49
CDK2	TOP1	9606.ENSP00000266970	9606.ENSP00000354522	0	0	0	0	0.091	0.066	0	0.476	0.517
CDK2	CDK5R1	9606.ENSP00000266970	9606.ENSP00000318486	0	0	0	0	0.061	0.32	0	0.421	0.598
CDK2	PLK1	9606.ENSP00000266970	9606.ENSP00000300093	0	0	0	0.631	0.296	0.267	0	0.724	0.603
CDK2	TOP2A	9606.ENSP00000266970	9606.ENSP00000411532	0	0	0	0	0.377	0.052	0	0.423	0.63
CDK2	ESR1	9606.ENSP00000266970	9606.ENSP00000405330	0	0	0	0	0	0.354	0	0.582	0.718
CDK2	TYMS	9606.ENSP00000266970	9606.ENSP00000315644	0	0	0	0	0.686	0	0	0.467	0.825
CDK2	CDK5	9606.ENSP00000266970	9606.ENSP00000419782	0	0	0.435	0.959	0	0.064	0.9	0.744	0.906
CDK5	NR3C1	9606.ENSP00000419782	9606.ENSP00000231509	0	0	0	0	0	0	0.9	0.317	0.928
CDK5	CDK6	9606.ENSP00000419782	9606.ENSP00000265734	0	0	0.411	0.912	0.058	0.094	0.9	0.6	0.914
CDK5	TNKS	9606.ENSP00000419782	9606.ENSP00000311579	0	0	0	0	0	0.079	0	0.439	0.461

CDK5	CDK5R1	9606.ENSP00000419782	9606.ENSP00000318486	0	0	0	0	0.061	0.975	0.9	0.923	0.999
CDK5	GSK3B	9606.ENSP00000419782	9606.ENSP00000324806	0	0	0.317	0.824	0.062	0.396	0.9	0.768	0.949
CDK5	MAPT	9606.ENSP00000419782	9606.ENSP00000340820	0	0	0	0	0.061	0.379	0.9	0.883	0.992
CDK5	SRC	9606.ENSP00000419782	9606.ENSP00000362680	0	0	0	0.663	0.049	0.437	0	0.553	0.542
CDK5R1	NR3C1	9606.ENSP00000318486	9606.ENSP00000231509	0	0	0	0	0.061	0	0.9	0.074	0.905
CDK5R1	CDK6	9606.ENSP00000318486	9606.ENSP00000265734	0	0	0	0	0.049	0.32	0	0.256	0.477
CDK5R1	GSK3B	9606.ENSP00000318486	9606.ENSP00000324806	0	0	0	0	0.061	0.08	0.9	0.186	0.92
CDK5R1	MAPT	9606.ENSP00000318486	9606.ENSP00000340820	0	0	0	0	0.139	0.294	0.8	0.638	0.95
CDK6	TYMS	9606.ENSP00000265734	9606.ENSP00000315644	0	0	0	0	0.213	0	0	0.284	0.412
CDK6	MMP9	9606.ENSP00000265734	9606.ENSP00000361405	0	0	0	0	0.062	0	0	0.411	0.423
CDK6	PIK3R1	9606.ENSP00000265734	9606.ENSP00000428056	0	0	0	0	0	0.157	0	0.354	0.432
CDK6	TOP2A	9606.ENSP00000265734	9606.ENSP00000411532	0	0	0	0	0.182	0.05	0	0.35	0.451
CDK6	PLK1	9606.ENSP00000265734	9606.ENSP00000300093	0	0	0	0.611	0.129	0.261	0	0.505	0.455

CDK6	ESR1	9606.ENSP00000265734	9606.ENSP00000405330	0	0	0	0	0	0	0	0.498	0.497
CDK6	GSK3B	9606.ENSP00000265734	9606.ENSP00000324806	0	0	0.304	0.73	0.063	0.093	0.9	0.386	0.922
CFTR	MYLK	9606.ENSP00000003084	9606.ENSP00000353452	0	0	0	0	0	0	0	0.406	0.406
CFTR	SRC	9606.ENSP00000003084	9606.ENSP00000362680	0	0	0	0	0	0.057	0	0.403	0.412
CFTR	SYK	9606.ENSP00000003084	9606.ENSP00000364907	0	0	0	0	0.049	0.057	0	0.511	0.523
CFTR	F2	9606.ENSP00000003084	9606.ENSP00000308541	0	0	0	0	0	0	0.9	0.246	0.921
CFTR	EGFR	9606.ENSP00000003084	9606.ENSP00000275493	0	0	0	0	0	0.051	0.9	0.383	0.936
CSNK2A1	SRC	9606.ENSP00000217244	9606.ENSP00000362680	0	0	0	0.568	0.058	0.357	0	0.3	0.442
CSNK2A1	MAPT	9606.ENSP00000217244	9606.ENSP00000340820	0	0	0	0	0	0.379	0	0.19	0.475
CSNK2A1	TOP1	9606.ENSP00000217244	9606.ENSP00000354522	0	0	0	0	0.064	0.379	0	0.243	0.521
CSNK2A1	ODC1	9606.ENSP00000217244	9606.ENSP00000234111	0	0	0	0	0.085	0	0	0.535	0.556
CSNK2A1	PTPN1	9606.ENSP00000217244	9606.ENSP00000360683	0	0	0	0	0.082	0.379	0	0.417	0.639
CSNK2A1	TOP2A	9606.ENSP00000217244	9606.ENSP00000411532	0	0	0	0	0.084	0.892	0	0.463	0.942

CTSD	MMP2	9606.ENSP00000236671	9606.ENSP00000219070	0	0	0	0	0.051	0	0	0.457	0.462
CTSD	EGFR	9606.ENSP00000236671	9606.ENSP00000275493	0	0	0	0	0	0.099	0	0.495	0.526
CTSD	MMP9	9606.ENSP00000236671	9606.ENSP00000361405	0	0	0	0	0.08	0	0.9	0.445	0.944
CTSD	ESR1	9606.ENSP00000236671	9606.ENSP00000405330	0	0	0	0	0.087	0	0.9	0.587	0.959
CXCR1	DRD4	9606.ENSP00000295683	9606.ENSP00000176183	0	0	0	0.58	0	0	0.9	0.053	0.9
CXCR1	MPO	9606.ENSP00000295683	9606.ENSP00000225275	0	0	0	0	0.098	0.056	0	0.465	0.505
CXCR1	EGFR	9606.ENSP00000295683	9606.ENSP00000275493	0	0	0	0	0	0.084	0	0.404	0.431
CXCR1	MMP9	9606.ENSP00000295683	9606.ENSP00000361405	0	0	0	0	0.14	0.064	0	0.478	0.543
CYP17A1	HSD17B2	9606.ENSP00000358903	9606.ENSP00000199936	0	0	0	0	0.064	0.064	0.9	0.607	0.96
CYP17A1	NR3C1	9606.ENSP00000358903	9606.ENSP00000231509	0	0	0	0	0	0.085	0	0.446	0.471
CYP17A1	ESR2	9606.ENSP00000358903	9606.ENSP00000343925	0	0	0	0	0	0.085	0	0.578	0.597
CYP17A1	XDH	9606.ENSP00000358903	9606.ENSP00000368727	0	0	0	0	0.055	0	0	0.41	0.42
CYP17A1	SRC	9606.ENSP00000358903	9606.ENSP00000362680	0	0	0	0	0	0.055	0	0.514	0.521

CYP17A1	SHBG	9606.ENSP00000358903	9606.ENSP00000369816	0	0	0	0	0	0	0	0.587	0.587
CYP17A1	ESR1	9606.ENSP00000358903	9606.ENSP00000405330	0	0	0	0	0.061	0.085	0	0.601	0.627
CYP17A1	HSD17B1	9606.ENSP00000358903	9606.ENSP00000466799	0	0	0	0	0	0.092	0	0.792	0.803
CYP17A1	CYP19A1	9606.ENSP00000358903	9606.ENSP00000379683	0	0	0.421	0.575	0	0	0.9	0.921	0.949
CYP19A1	CYP51A1	9606.ENSP00000379683	9606.ENSP00000003100	0	0	0.285	0.577	0.108	0.06	0	0.581	0.405
CYP19A1	HSD17B2	9606.ENSP00000379683	9606.ENSP00000199936	0	0	0	0	0	0.082	0.9	0.629	0.962
CYP19A1	NR3C1	9606.ENSP00000379683	9606.ENSP00000231509	0	0	0	0	0	0.085	0	0.455	0.48
CYP19A1	IGF1R	9606.ENSP00000379683	9606.ENSP00000268035	0	0	0	0	0	0.055	0	0.556	0.562
CYP19A1	EGFR	9606.ENSP00000379683	9606.ENSP00000275493	0	0	0	0	0	0	0	0.648	0.648
CYP19A1	HMGCR	9606.ENSP00000379683	9606.ENSP00000287936	0	0	0	0	0.063	0	0	0.42	0.433
CYP19A1	ESR2	9606.ENSP00000379683	9606.ENSP00000343925	0	0	0	0	0.062	0.085	0	0.845	0.855
CYP19A1	PTGS2	9606.ENSP00000379683	9606.ENSP00000356438	0	0	0	0	0.05	0	0	0.635	0.639
CYP19A1	SRC	9606.ENSP00000379683	9606.ENSP00000362680	0	0	0	0	0	0.055	0	0.443	0.451

CYP19A1	SHBG	9606.ENSP00000379683	9606.ENSP00000369816	0	0	0	0	0	0	0	0.796	0.796
CYP19A1	CYP1B1	9606.ENSP00000379683	9606.ENSP00000478561	0	0	0.365	0.555	0	0	0	0.679	0.426
CYP19A1	ESRRA	9606.ENSP00000379683	9606.ENSP00000384851	0	0	0	0	0	0.085	0	0.414	0.441
CYP19A1	ESR1	9606.ENSP00000379683	9606.ENSP00000405330	0	0	0	0	0	0.085	0	0.96	0.962
CYP19A1	HSD17B1	9606.ENSP00000379683	9606.ENSP00000466799	0	0	0	0	0.063	0.059	0.9	0.845	0.984
CYP1B1	HSD17B2	9606.ENSP00000478561	9606.ENSP00000199936	0	0	0	0	0.063	0.064	0.9	0.35	0.935
CYP1B1	MMP2	9606.ENSP00000478561	9606.ENSP00000219070	0	0	0	0	0.063	0	0	0.479	0.491
CYP1B1	ESR2	9606.ENSP00000478561	9606.ENSP00000343925	0	0	0	0	0.063	0.085	0	0.689	0.711
CYP1B1	PTGS2	9606.ENSP00000478561	9606.ENSP00000356438	0	0	0	0	0.101	0	0	0.656	0.678
CYP1B1	SHBG	9606.ENSP00000478561	9606.ENSP00000369816	0	0	0	0	0.062	0	0	0.399	0.412
CYP1B1	ESR1	9606.ENSP00000478561	9606.ENSP00000405330	0	0	0	0	0.073	0.085	0	0.645	0.673
CYP1B1	HSD17B1	9606.ENSP00000478561	9606.ENSP00000466799	0	0	0	0	0	0.092	0.9	0.534	0.954
CYP51A1	HMGCR	9606.ENSP00000003100	9606.ENSP00000287936	0	0	0	0	0.863	0	0	0.839	0.977

DAPK1	MAPT	9606.ENSP00000386135	9606.ENSP00000340820	0	0	0	0	0	0	0	0.646	0.646
DAPK1	ESR1	9606.ENSP00000386135	9606.ENSP00000405330	0	0	0	0	0	0	0	0.444	0.444
DRD4	MAOB	9606.ENSP00000176183	9606.ENSP00000367309	0	0	0	0	0	0	0	0.67	0.67
DRD4	MAOA	9606.ENSP00000176183	9606.ENSP00000340684	0	0	0	0	0	0	0	0.787	0.787
EGFR	MMP2	9606.ENSP00000275493	9606.ENSP00000219070	0	0	0	0	0.11	0	0	0.673	0.697
EGFR	IL2	9606.ENSP00000275493	9606.ENSP00000226730	0	0	0	0	0	0	0.6	0.548	0.811
EGFR	NR3C1	9606.ENSP00000275493	9606.ENSP00000231509	0	0	0	0	0	0.415	0	0.414	0.643
EGFR	MMP13	9606.ENSP00000275493	9606.ENSP00000260302	0	0	0	0	0	0	0	0.6	0.601
EGFR	NOX4	9606.ENSP00000275493	9606.ENSP00000263317	0	0	0	0	0.063	0	0	0.619	0.628
EGFR	KDR	9606.ENSP00000275493	9606.ENSP00000263923	0	0	0	0.559	0.074	0.177	0	0.914	0.525
EGFR	IGF1R	9606.ENSP00000275493	9606.ENSP00000268035	0	0	0	0.567	0	0.418	0	0.918	0.648
EGFR	F2	9606.ENSP00000275493	9606.ENSP00000308541	0	0	0	0	0.062	0	0	0.4	0.413
EGFR	PIM1	9606.ENSP00000275493	9606.ENSP00000362608	0	0	0	0.582	0	0.393	0	0.246	0.447

EGFR	TOP2A	9606.ENSP00000275493	9606.ENSP00000411532	0	0	0	0	0	0	0	0.53	0.53
EGFR	PIK3CG	9606.ENSP00000275493	9606.ENSP00000352121	0	0	0	0	0	0.08	0	0.511	0.53
EGFR	TOP1	9606.ENSP00000275493	9606.ENSP00000354522	0	0	0	0	0	0	0	0.531	0.531
EGFR	MAPT	9606.ENSP00000275493	9606.ENSP00000340820	0	0	0	0	0.062	0.393	0	0.296	0.564
EGFR	PTGES	9606.ENSP00000275493	9606.ENSP00000342385	0	0	0	0	0.067	0	0	0.554	0.566
EGFR	PARP1	9606.ENSP00000275493	9606.ENSP00000355759	0	0	0	0	0	0.099	0	0.546	0.573
EGFR	NPC1L1	9606.ENSP00000275493	9606.ENSP00000289547	0	0	0	0	0.062	0	0	0.604	0.613
EGFR	PTPRS	9606.ENSP00000275493	9606.ENSP00000349932	0	0	0	0	0.07	0.407	0	0.369	0.622
EGFR	TYMS	9606.ENSP00000275493	9606.ENSP00000315644	0	0	0	0	0	0	0	0.632	0.632
EGFR	MET	9606.ENSP00000275493	9606.ENSP00000317272	0	0	0	0.58	0.229	0.418	0	0.933	0.715
EGFR	ESR2	9606.ENSP00000275493	9606.ENSP00000343925	0	0	0	0	0	0.077	0	0.739	0.749
EGFR	MMP9	9606.ENSP00000275493	9606.ENSP00000361405	0	0	0	0	0	0	0	0.828	0.828
EGFR	PTGS2	9606.ENSP00000275493	9606.ENSP00000356438	0	0	0	0	0.061	0	0	0.869	0.872

EGFR	PTK2	9606.ENSP00000275493	9606.ENSP00000341189	0	0	0	0.597	0.109	0.479	0.8	0.69	0.926
EGFR	ESR1	9606.ENSP00000275493	9606.ENSP00000405330	0	0	0	0	0	0.402	0	0.908	0.943
EGFR	MMP3	9606.ENSP00000275493	9606.ENSP00000299855	0	0	0	0	0.076	0	0.9	0.543	0.954
EGFR	SRC	9606.ENSP00000275493	9606.ENSP00000362680	0	0	0	0.671	0	0.484	0.9	0.954	0.963
EGFR	PTPN1	9606.ENSP00000275493	9606.ENSP00000360683	0	0	0	0	0	0.47	0.9	0.798	0.988
EGFR	PIK3R1	9606.ENSP00000275493	9606.ENSP00000428056	0	0	0	0	0	0.882	0.9	0.581	0.994
ESR1	HSD17B2	9606.ENSP00000405330	9606.ENSP00000199936	0	0	0	0	0.061	0.05	0	0.427	0.444
ESR1	MMP2	9606.ENSP00000405330	9606.ENSP00000219070	0	0	0	0	0	0	0	0.532	0.532
ESR1	MPG	9606.ENSP00000405330	9606.ENSP00000219431	0	0	0	0	0	0.328	0.9	0.08	0.932
ESR1	NR3C1	9606.ENSP00000405330	9606.ENSP00000231509	0	0	0	0.66	0	0	0.9	0.751	0.925
ESR1	ODC1	9606.ENSP00000405330	9606.ENSP00000234111	0	0	0	0	0	0	0	0.432	0.432
ESR1	KDR	9606.ENSP00000405330	9606.ENSP00000263923	0	0	0	0	0	0.051	0	0.511	0.516
ESR1	IGF1R	9606.ENSP00000405330	9606.ENSP00000268035	0	0	0	0	0	0.407	0.9	0.835	0.989

ESR1	MMP3	9606.ENSP00000405330	9606.ENSP00000299855	0	0	0	0	0	0	0	0.426	0.426
ESR1	PLK1	9606.ENSP00000405330	9606.ENSP00000300093	0	0	0	0	0	0.05	0	0.399	0.404
ESR1	MET	9606.ENSP00000405330	9606.ENSP00000317272	0	0	0	0	0	0.085	0	0.448	0.473
ESR1	GSK3B	9606.ENSP00000405330	9606.ENSP00000324806	0	0	0	0	0	0.085	0	0.38	0.409
ESR1	MAPT	9606.ENSP00000405330	9606.ENSP00000340820	0	0	0	0	0	0	0	0.634	0.634
ESR1	ESR2	9606.ENSP00000405330	9606.ENSP00000343925	0	0	0	0.925	0.061	0.379	0.9	0.967	0.941
ESR1	TOP1	9606.ENSP00000405330	9606.ENSP00000354522	0	0	0	0	0.061	0.043	0	0.398	0.411
ESR1	PARP1	9606.ENSP00000405330	9606.ENSP00000355759	0	0	0	0	0	0.342	0	0.652	0.761
ESR1	PTGS2	9606.ENSP00000405330	9606.ENSP00000356438	0	0	0	0	0	0.05	0	0.771	0.773
ESR1	PTPN1	9606.ENSP00000405330	9606.ENSP00000360683	0	0	0	0	0	0.31	0	0.283	0.484
ESR1	MMP9	9606.ENSP00000405330	9606.ENSP00000361405	0	0	0	0	0	0	0	0.581	0.581
ESR1	SRC	9606.ENSP00000405330	9606.ENSP00000362680	0	0	0	0	0.061	0.52	0.9	0.803	0.99
ESR1	SHBG	9606.ENSP00000405330	9606.ENSP00000369816	0	0	0	0	0	0	0	0.683	0.683

ESR1	ESRRA	9606.ENSP00000405330	9606.ENSP00000384851	0	0	0	0.849	0	0.379	0.9	0.668	0.941
ESR1	TOP2A	9606.ENSP00000405330	9606.ENSP00000411532	0	0	0	0	0.061	0.043	0	0.477	0.488
ESR1	HSD17B1	9606.ENSP00000405330	9606.ENSP00000466799	0	0	0	0	0	0	0	0.59	0.59
ESR1	PIK3R1	9606.ENSP00000405330	9606.ENSP00000428056	0	0	0	0	0.063	0.404	0.9	0.418	0.963
ESR2	HSD17B2	9606.ENSP00000343925	9606.ENSP00000199936	0	0	0	0	0	0.05	0	0.427	0.432
ESR2	IGF1R	9606.ENSP00000343925	9606.ENSP00000268035	0	0	0	0	0	0.085	0	0.644	0.66
ESR2	PTGS2	9606.ENSP00000343925	9606.ENSP00000356438	0	0	0	0	0	0.05	0	0.439	0.444
ESR2	SHBG	9606.ENSP00000343925	9606.ENSP00000369816	0	0	0	0	0	0	0	0.479	0.479
ESR2	HSD17B1	9606.ENSP00000343925	9606.ENSP00000466799	0	0	0	0	0.062	0	0	0.632	0.64
ESR2	PIK3R1	9606.ENSP00000343925	9606.ENSP00000428056	0	0	0	0	0	0.081	0.9	0.222	0.922
ESR2	SRC	9606.ENSP00000343925	9606.ENSP00000362680	0	0	0	0	0	0.407	0.9	0.459	0.965
ESRRA	NR3C1	9606.ENSP00000384851	9606.ENSP00000231509	0	0	0	0.746	0	0	0.9	0.348	0.908
ESRRA	UBA2	9606.ENSP00000384851	9606.ENSP00000246548	0	0	0	0	0	0.404	0	0	0.404

ESRRA	SAE1	9606.ENSP00000384851	9606.ENSP00000270225	0	0	0	0	0	0.421	0	0	0.421
ESRRA	MAOB	9606.ENSP00000384851	9606.ENSP00000367309	0	0	0	0	0	0.064	0	0.476	0.489
ESRRA	PPARA	9606.ENSP00000384851	9606.ENSP00000385523	0	0	0	0.616	0.05	0	0.9	0.719	0.927
F2	MPO	9606.ENSP00000308541	9606.ENSP00000225275	0	0	0	0	0	0.057	0	0.479	0.488
F2	IL2	9606.ENSP00000308541	9606.ENSP00000226730	0	0	0	0	0	0	0	0.406	0.406
F2	TTR	9606.ENSP00000308541	9606.ENSP00000237014	0	0	0	0	0.278	0	0	0.558	0.667
F2	MMP13	9606.ENSP00000308541	9606.ENSP00000260302	0	0	0	0	0	0	0	0.465	0.465
F2	TYR	9606.ENSP00000308541	9606.ENSP00000263321	0	0	0	0	0.527	0	0	0.322	0.665
F2	PTGES	9606.ENSP00000308541	9606.ENSP00000342385	0	0	0	0	0	0	0	0.448	0.448
F2	PARP1	9606.ENSP00000308541	9606.ENSP00000355759	0	0	0	0	0	0	0	0.477	0.477
F2	MMP9	9606.ENSP00000308541	9606.ENSP00000361405	0	0	0	0	0	0	0	0.481	0.481
F2	PTGS2	9606.ENSP00000308541	9606.ENSP00000356438	0	0	0	0	0	0.057	0	0.502	0.511
F2	PIK3R1	9606.ENSP00000308541	9606.ENSP00000428056	0	0	0	0	0	0	0.9	0.085	0.904

F2	PPARA	9606.ENSP00000308541	9606.ENSP00000385523	0	0	0	0	0.063	0	0.9	0.106	0.908
F2	SRC	9606.ENSP00000308541	9606.ENSP00000362680	0	0	0	0	0	0.05	0.9	0.608	0.959
FLT3	MPO	9606.ENSP00000241453	9606.ENSP00000225275	0	0	0	0	0.317	0	0	0.447	0.606
FLT3	IL2	9606.ENSP00000241453	9606.ENSP00000226730	0	0	0	0	0	0	0	0.556	0.556
FLT3	SYK	9606.ENSP00000241453	9606.ENSP00000364907	0	0	0	0.607	0.098	0.414	0	0.662	0.589
FLT3	PIM1	9606.ENSP00000241453	9606.ENSP00000362608	0	0	0	0	0	0	0	0.721	0.721
FLT3	PIK3R1	9606.ENSP00000241453	9606.ENSP00000428056	0	0	0	0	0.065	0.38	0.9	0.237	0.949
GLO1	MAOA	9606.ENSP00000362463	9606.ENSP00000340684	0	0	0	0	0	0	0.8	0.189	0.831
GLO1	MAOB	9606.ENSP00000362463	9606.ENSP00000367309	0	0	0	0	0	0	0.8	0.133	0.819
GSK3B	NR3C1	9606.ENSP00000324806	9606.ENSP00000231509	0	0	0	0	0	0.085	0.9	0.432	0.943
GSK3B	IGF1R	9606.ENSP00000324806	9606.ENSP00000268035	0	0	0	0	0.062	0.056	0	0.468	0.488
GSK3B	TOP2A	9606.ENSP00000324806	9606.ENSP00000411532	0	0	0	0	0.062	0.294	0	0.208	0.429
GSK3B	PTK2	9606.ENSP00000324806	9606.ENSP00000341189	0	0	0	0.569	0	0.433	0	0.27	0.491

GSK3B	SRC	9606.ENSP00000324806	9606.ENSP00000362680	0	0	0	0	0	0.123	0	0.486	0.53
GSK3B	PTGS2	9606.ENSP00000324806	9606.ENSP00000356438	0	0	0	0	0	0.058	0	0.63	0.637
GSK3B	PTPN1	9606.ENSP00000324806	9606.ENSP00000360683	0	0	0	0	0.062	0.456	0	0.454	0.697
GSK3B	PIK3R1	9606.ENSP00000324806	9606.ENSP00000428056	0	0	0	0	0.062	0.085	0.8	0.303	0.864
GSK3B	MAPT	9606.ENSP00000324806	9606.ENSP00000340820	0	0	0	0	0.061	0.379	0.9	0.804	0.987
HMGCR	NOS2	9606.ENSP00000287936	9606.ENSP00000327251	0	0	0	0	0.049	0	0	0.5	0.505
HMGCR	PPARA	9606.ENSP00000287936	9606.ENSP00000385523	0	0	0	0	0.061	0	0	0.562	0.571
HMGCR	NR1H3	9606.ENSP00000287936	9606.ENSP00000477707	0	0	0	0	0.061	0	0	0.613	0.621
HMGCR	NPC1L1	9606.ENSP00000287936	9606.ENSP00000289547	0	0	0	0	0.061	0	0	0.667	0.673
HSD17B1	HSD17B2	9606.ENSP00000466799	9606.ENSP00000199936	0	0	0	0	0	0	0.8	0.838	0.966
HSD17B1	SHBG	9606.ENSP00000466799	9606.ENSP00000369816	0	0	0	0	0	0	0	0.42	0.42
HSD17B2	NR3C1	9606.ENSP00000199936	9606.ENSP00000231509	0	0	0	0	0	0.05	0	0.473	0.477
HSD17B2	UBA2	9606.ENSP00000199936	9606.ENSP00000246548	0	0	0	0	0	0	0	0.636	0.636

IGF1R	MMP2	9606.ENSP00000268035	9606.ENSP00000219070	0	0	0	0	0	0	0	0.473	0.473
IGF1R	TTR	9606.ENSP00000268035	9606.ENSP00000237014	0	0	0	0	0	0	0	0.592	0.592
IGF1R	PTGS2	9606.ENSP00000268035	9606.ENSP00000356438	0	0	0	0	0	0.094	0	0.389	0.422
IGF1R	MMP9	9606.ENSP00000268035	9606.ENSP00000361405	0	0	0	0	0	0	0	0.473	0.473
IGF1R	MET	9606.ENSP00000268035	9606.ENSP00000317272	0	0	0	0.579	0	0.389	0	0.707	0.567
IGF1R	PIK3CG	9606.ENSP00000268035	9606.ENSP00000352121	0	0	0	0	0.062	0.124	0	0.522	0.573
IGF1R	PTK2	9606.ENSP00000268035	9606.ENSP00000341189	0	0	0	0.61	0	0.365	0.8	0.51	0.892
IGF1R	SRC	9606.ENSP00000268035	9606.ENSP00000362680	0	0	0	0.711	0	0.408	0.9	0.835	0.953
IGF1R	PIK3R1	9606.ENSP00000268035	9606.ENSP00000428056	0	0	0	0	0.049	0.586	0.9	0.546	0.979
IGF1R	INSR	9606.ENSP00000268035	9606.ENSP00000303830	0	0	0	0.957	0.062	0.974	0.8	0.869	0.994
IGF1R	PTPN1	9606.ENSP00000268035	9606.ENSP00000360683	0	0	0	0	0.062	0.973	0.9	0.656	0.999
IL2	MMP2	9606.ENSP00000226730	9606.ENSP00000219070	0	0	0	0	0	0	0	0.44	0.44
IL2	MPO	9606.ENSP00000226730	9606.ENSP00000225275	0	0	0	0	0	0	0	0.548	0.548

IL2	MMP3	9606.ENSP00000226730	9606.ENSP00000299855	0	0	0	0	0	0	0	0.43	0.43
IL2	NR3C1	9606.ENSP00000226730	9606.ENSP00000231509	0	0	0	0	0	0	0	0.473	0.473
IL2	KDR	9606.ENSP00000226730	9606.ENSP00000263923	0	0	0	0	0	0	0	0.473	0.473
IL2	NOS2	9606.ENSP00000226730	9606.ENSP00000327251	0	0	0	0	0	0	0	0.486	0.486
IL2	TYR	9606.ENSP00000226730	9606.ENSP00000263321	0	0	0	0	0	0	0	0.53	0.53
IL2	ODC1	9606.ENSP00000226730	9606.ENSP00000234111	0	0	0	0	0	0	0	0.555	0.555
IL2	MMP9	9606.ENSP00000226730	9606.ENSP00000361405	0	0	0	0	0	0	0	0.556	0.556
IL2	SRC	9606.ENSP00000226730	9606.ENSP00000362680	0	0	0	0	0	0	0	0.571	0.571
IL2	PTGS2	9606.ENSP00000226730	9606.ENSP00000356438	0	0	0	0	0	0	0	0.577	0.577
IL2	RORC	9606.ENSP00000226730	9606.ENSP00000327025	0	0	0	0	0	0	0	0.586	0.586
IL2	PIK3R1	9606.ENSP00000226730	9606.ENSP00000428056	0	0	0	0	0	0	0.9	0.232	0.919
IL2	SYK	9606.ENSP00000226730	9606.ENSP00000364907	0	0	0	0	0	0	0.9	0.559	0.954
INSR	PTK2	9606.ENSP00000303830	9606.ENSP00000341189	0	0	0	0.599	0	0.433	0	0.198	0.47

INSR	MET	9606.ENSP00000303830	9606.ENSP00000317272	0	0	0	0.576	0.061	0.389	0	0.312	0.473
INSR	PIK3CG	9606.ENSP00000303830	9606.ENSP00000352121	0	0	0	0	0.062	0.124	0	0.429	0.49
INSR	SRC	9606.ENSP00000303830	9606.ENSP00000362680	0	0	0	0.708	0	0.468	0	0.446	0.534
INSR	PIK3R1	9606.ENSP00000303830	9606.ENSP00000428056	0	0	0	0	0.061	0.472	0.9	0.492	0.971
INSR	PTPN1	9606.ENSP00000303830	9606.ENSP00000360683	0	0	0	0	0.062	0.972	0.9	0.418	0.998
KDR	MMP2	9606.ENSP00000263923	9606.ENSP00000219070	0	0	0	0	0.098	0	0	0.648	0.668
KDR	NOX4	9606.ENSP00000263923	9606.ENSP00000263317	0	0	0	0	0.061	0	0	0.456	0.468
KDR	MMP3	9606.ENSP00000263923	9606.ENSP00000299855	0	0	0	0	0	0	0	0.426	0.426
KDR	PIK3CG	9606.ENSP00000263923	9606.ENSP00000352121	0	0	0	0	0	0.263	0	0.275	0.443
KDR	PPARA	9606.ENSP00000263923	9606.ENSP00000385523	0	0	0	0	0	0.051	0	0.454	0.459
KDR	PTGS2	9606.ENSP00000263923	9606.ENSP00000356438	0	0	0	0	0	0	0	0.511	0.511
KDR	MET	9606.ENSP00000263923	9606.ENSP00000317272	0	0	0	0.564	0	0.472	0	0.727	0.636
KDR	PTPN1	9606.ENSP00000263923	9606.ENSP00000360683	0	0	0	0	0	0.094	0	0.63	0.651

KDR	MMP9	9606.ENSP00000263923	9606.ENSP00000361405	0	0	0	0	0	0	0	0.677	0.677
KDR	PTK2	9606.ENSP00000263923	9606.ENSP00000341189	0	0	0	0.577	0.065	0.093	0.9	0.591	0.93
KDR	PIK3R1	9606.ENSP00000263923	9606.ENSP00000428056	0	0	0	0	0.055	0.157	0.9	0.243	0.931
KDR	SRC	9606.ENSP00000263923	9606.ENSP00000362680	0	0	0	0.651	0.058	0.46	0.9	0.843	0.96
MAOA	NR3C1	9606.ENSP00000340684	9606.ENSP00000231509	0	0	0	0	0	0.064	0	0.527	0.538
MAOA	MAOB	9606.ENSP00000340684	9606.ENSP00000367309	0	0	0.448	0.976	0.18	0.685	0.8	0.931	0.945
MAOB	MAPT	9606.ENSP00000367309	9606.ENSP00000340820	0	0	0	0	0.088	0	0	0.4	0.429
MAPT	PKN1	9606.ENSP00000340820	9606.ENSP00000343325	0	0	0	0	0.048	0.379	0	0.067	0.4
MAPT	PIK3R1	9606.ENSP00000340820	9606.ENSP00000428056	0	0	0	0	0	0.379	0	0.116	0.427
MAPT	SRC	9606.ENSP00000340820	9606.ENSP00000362680	0	0	0	0	0	0.379	0	0.355	0.582
MAPT	SYK	9606.ENSP00000340820	9606.ENSP00000364907	0	0	0	0	0	0.379	0	0.524	0.692
MET	MMP2	9606.ENSP00000317272	9606.ENSP00000219070	0	0	0	0	0.065	0	0	0.511	0.523
MET	PTGS2	9606.ENSP00000317272	9606.ENSP00000356438	0	0	0	0	0.061	0.058	0	0.4	0.422

MET	MMP 9	9606.ENSP00 000317272	9606.ENSP00 000361405	0	0	0	0	0	0	0	0.546	0.546
MET	PARP 1	9606.ENSP00 000317272	9606.ENSP00 000355759	0	0	0	0	0.086	0.057	0	0.555	0.583
MET	PTK2	9606.ENSP00 000317272	9606.ENSP00 000341189	0	0	0	0.592	0.075	0.125	0.9	0.542	0.93
MET	PIK3R 1	9606.ENSP00 000317272	9606.ENSP00 000428056	0	0	0	0	0	0.407	0.9	0.262	0.952
MET	SRC	9606.ENSP00 000317272	9606.ENSP00 000362680	0	0	0	0.684	0.063	0.407	0.9	0.795	0.954
MET	PTPN 1	9606.ENSP00 000317272	9606.ENSP00 000360683	0	0	0	0	0.062	0.538	0.9	0.323	0.966
MMP 12	MMP 9	9606.ENSP00 000458585	9606.ENSP00 000361405	0	0	0	0.732	0.506	0	0	0.816	0.613
MMP 13	MMP 2	9606.ENSP00 000260302	9606.ENSP00 000219070	0	0	0	0.89	0.061	0	0.9	0.835	0.911
MMP 13	PTGE S	9606.ENSP00 000260302	9606.ENSP00 000342385	0	0	0	0	0.057	0	0	0.44	0.449
MMP 13	PTGS 2	9606.ENSP00 000260302	9606.ENSP00 000356438	0	0	0	0	0.063	0	0	0.606	0.615
MMP 13	MMP 3	9606.ENSP00 000260302	9606.ENSP00 000299855	0	0	0.249	0.944	0.085	0	0.9	0.893	0.91
MMP 13	MMP 9	9606.ENSP00 000260302	9606.ENSP00 000361405	0	0	0	0.797	0.064	0	0.9	0.878	0.919
MMP 2	MPO	9606.ENSP00 000219070	9606.ENSP00 000225275	0	0	0	0	0	0	0	0.511	0.511

MMP2	PTK2	9606.ENSP00000219070	9606.ENSP00000341189	0	0	0	0	0.051	0	0	0.638	0.642
MMP2	PTGS2	9606.ENSP00000219070	9606.ENSP00000356438	0	0	0	0	0.066	0	0	0.696	0.704
MMP2	MMP9	9606.ENSP00000219070	9606.ENSP00000361405	0	0	0	0.929	0.061	0	0.9	0.958	0.908
MMP2	MMP3	9606.ENSP00000219070	9606.ENSP00000299855	0	0	0	0.88	0.181	0	0.9	0.892	0.923
MMP2	SRC	9606.ENSP00000219070	9606.ENSP00000362680	0	0	0	0	0.055	0	0.9	0.748	0.974
MMP3	MPO	9606.ENSP00000299855	9606.ENSP00000225275	0	0	0	0	0	0	0	0.456	0.456
MMP3	PTGES	9606.ENSP00000299855	9606.ENSP00000342385	0	0	0	0	0.061	0	0	0.39	0.402
MMP3	SRC	9606.ENSP00000299855	9606.ENSP00000362680	0	0	0	0	0	0	0	0.46	0.46
MMP3	PTGS2	9606.ENSP00000299855	9606.ENSP00000356438	0	0	0	0	0.095	0	0	0.774	0.787
MMP3	MMP9	9606.ENSP00000299855	9606.ENSP00000361405	0	0	0	0.791	0.518	0	0.9	0.911	0.959
MMP9	MPO	9606.ENSP00000361405	9606.ENSP00000225275	0	0	0	0	0.076	0	0	0.704	0.714
MMP9	NOX4	9606.ENSP00000361405	9606.ENSP00000263317	0	0	0	0	0	0	0	0.454	0.454
MMP9	NOS2	9606.ENSP00000361405	9606.ENSP00000327251	0	0	0	0	0.064	0	0	0.519	0.53

MMP9	PTK2	9606.ENSP00000361405	9606.ENSP00000341189	0	0	0	0	0.061	0	0	0.625	0.633
MMP9	PTGES	9606.ENSP00000361405	9606.ENSP00000342385	0	0	0	0	0	0	0	0.421	0.42
MMP9	PTGS2	9606.ENSP00000361405	9606.ENSP00000356438	0	0	0	0	0.097	0	0	0.725	0.741
MMP9	SRC	9606.ENSP00000361405	9606.ENSP00000362680	0	0	0	0	0.088	0	0.9	0.796	0.979
MPG	POLB	9606.ENSP00000219431	9606.ENSP00000265421	0.167	0	0	0	0	0	0	0.507	0.572
MPO	NOX4	9606.ENSP00000225275	9606.ENSP00000263317	0	0	0	0	0.049	0.085	0	0.494	0.521
MPO	PLA2G1B	9606.ENSP00000225275	9606.ENSP00000312286	0	0	0	0	0	0	0	0.532	0.532
MPO	XDH	9606.ENSP00000225275	9606.ENSP00000368727	0	0	0	0	0	0	0	0.563	0.563
MPO	NOS2	9606.ENSP00000225275	9606.ENSP00000327251	0	0	0	0	0	0.157	0	0.595	0.644
MPO	PTGS2	9606.ENSP00000225275	9606.ENSP00000356438	0	0	0	0	0.065	0	0	0.691	0.699
MPO	TTR	9606.ENSP00000225275	9606.ENSP00000237014	0	0	0	0	0	0	0.9	0.242	0.921
MYLK	PTK2	9606.ENSP00000353452	9606.ENSP00000341189	0	0	0	0.546	0.074	0	0.9	0.438	0.921
MYLK	SRC	9606.ENSP00000353452	9606.ENSP00000362680	0	0	0	0.567	0	0.405	0	0.725	0.589

NEK2	PLK1	9606.ENSP00000355966	9606.ENSP00000300093	0	0	0	0.649	0.617	0	0.9	0.83	0.971
NEK2	TNKS	9606.ENSP00000355966	9606.ENSP00000311579	0	0	0	0	0.088	0.061	0	0.378	0.42
NEK2	TYMS	9606.ENSP00000355966	9606.ENSP00000315644	0	0	0	0	0.339	0	0	0.144	0.41
NEK2	TNKS2	9606.ENSP00000355966	9606.ENSP00000360689	0	0	0	0	0.088	0.061	0	0.37	0.413
NEK2	TOP2A	9606.ENSP00000355966	9606.ENSP00000411532	0	0	0	0	0.923	0	0	0.45	0.955
NOS2	ODC1	9606.ENSP00000327251	9606.ENSP00000234111	0.043	0	0	0	0.061	0	0	0.653	0.662
NOS2	PARP1	9606.ENSP00000327251	9606.ENSP00000355759	0	0	0	0	0.061	0	0	0.512	0.523
NOS2	PTGS2	9606.ENSP00000327251	9606.ENSP00000356438	0	0	0	0	0.063	0.299	0	0.868	0.906
NOS2	PPARA	9606.ENSP00000327251	9606.ENSP00000385523	0	0	0	0	0	0.091	0.9	0.211	0.922
NOS2	SRC	9606.ENSP00000327251	9606.ENSP00000362680	0	0	0	0	0.064	0.394	0.9	0.6	0.974
NOX4	PTGS2	9606.ENSP00000263317	9606.ENSP00000356438	0	0	0	0	0.049	0.085	0	0.464	0.493
NOX4	SRC	9606.ENSP00000263317	9606.ENSP00000362680	0	0	0	0	0	0.079	0	0.485	0.505
NOX4	XDH	9606.ENSP00000263317	9606.ENSP00000368727	0	0	0	0	0.061	0	0	0.574	0.583

NOX4	PTPN1	9606.ENSP00000263317	9606.ENSP00000360683	0	0	0	0	0.05	0	0.9	0.515	0.949
NPC1L1	NR1H3	9606.ENSP00000289547	9606.ENSP00000477707	0	0	0	0	0	0	0	0.45	0.45
NR1H3	NR3C1	9606.ENSP00000477707	9606.ENSP00000231509	0	0	0	0.62	0	0.379	0	0.412	0.47
NR1H3	PPARA	9606.ENSP00000477707	9606.ENSP00000385523	0	0	0	0.714	0.063	0.379	0.9	0.733	0.949
NR3C1	PTGS2	9606.ENSP00000231509	9606.ENSP00000356438	0	0	0	0	0.069	0.05	0	0.475	0.495
NR3C1	SRC	9606.ENSP00000231509	9606.ENSP00000362680	0	0	0	0	0	0.326	0	0.473	0.629
NR3C1	PIK3R1	9606.ENSP00000231509	9606.ENSP00000428056	0	0	0	0	0.062	0.081	0.9	0.211	0.922
NUAK1	PLK1	9606.ENSP00000261402	9606.ENSP00000300093	0	0	0	0.645	0	0.354	0	0.654	0.5
ODC1	PTGS2	9606.ENSP00000234111	9606.ENSP00000356438	0	0	0	0	0	0	0	0.515	0.515
ODC1	TYMS	9606.ENSP00000234111	9606.ENSP00000315644	0.119	0	0	0	0.135	0	0	0.567	0.641
PARP1	POLB	9606.ENSP00000355759	9606.ENSP00000265421	0	0	0	0	0	0.747	0.9	0.491	0.986
PARP1	TNKS	9606.ENSP00000355759	9606.ENSP00000311579	0	0	0	0	0.078	0	0	0.719	0.73
PARP1	PTK2	9606.ENSP00000355759	9606.ENSP00000341189	0	0	0	0	0	0.294	0	0.185	0.4

PARP1	TOP1	9606.ENSP00000355759	9606.ENSP00000354522	0	0	0	0	0.062	0.379	0	0.81	0.88
PARP1	TOP2A	9606.ENSP00000355759	9606.ENSP00000411532	0	0	0	0	0.175	0	0	0.326	0.42
PARP1	SRC	9606.ENSP00000355759	9606.ENSP00000362680	0	0	0	0	0.061	0.189	0	0.362	0.472
PARP1	TNKS2	9606.ENSP00000355759	9606.ENSP00000360689	0	0	0	0	0.078	0	0	0.683	0.695
PIK3CG	SRC	9606.ENSP00000352121	9606.ENSP00000362680	0	0	0	0	0.058	0.124	0	0.419	0.479
PIK3CG	SYK	9606.ENSP00000352121	9606.ENSP00000364907	0	0	0	0	0.147	0.378	0.9	0.23	0.953
PIK3CG	PIK3R1	9606.ENSP00000352121	9606.ENSP00000428056	0	0	0	0	0.061	0.446	0.9	0.691	0.981
PIK3R1	PTK2	9606.ENSP00000428056	9606.ENSP00000341189	0	0	0	0	0	0.379	0.9	0.209	0.946
PIK3R1	PTPN1	9606.ENSP00000428056	9606.ENSP00000360683	0	0	0	0	0	0.123	0.9	0.234	0.927
PIK3R1	SRC	9606.ENSP00000428056	9606.ENSP00000362680	0	0	0	0	0	0.701	0.9	0.544	0.985
PIK3R1	SYK	9606.ENSP00000428056	9606.ENSP00000364907	0	0	0	0	0	0.407	0.9	0.344	0.957
PLA2G1B	PTGES	9606.ENSP00000312286	9606.ENSP00000342385	0	0	0	0	0	0	0	0.553	0.553
PLA2G1B	PTGS2	9606.ENSP00000312286	9606.ENSP00000356438	0	0	0	0	0	0	0.65	0.669	0.879

PLK1	TOP1	9606.ENSP00000300093	9606.ENSP00000354522	0	0	0	0	0.091	0	0	0.382	0.414
PLK1	PTPN1	9606.ENSP00000300093	9606.ENSP00000360683	0	0	0	0	0	0	0	0.506	0.506
PLK1	TNKS	9606.ENSP00000300093	9606.ENSP00000311579	0	0	0	0	0.06	0.164	0	0.587	0.647
PLK1	TYMS	9606.ENSP00000300093	9606.ENSP00000315644	0	0	0	0	0.626	0	0	0.294	0.724
PLK1	TOP2A	9606.ENSP00000300093	9606.ENSP00000411532	0	0	0	0	0.854	0.294	0	0.551	0.949
POLB	TYMS	9606.ENSP00000265421	9606.ENSP00000315644	0	0	0	0	0	0	0	0.422	0.422
PPARA	PTGS2	9606.ENSP00000385523	9606.ENSP00000356438	0	0	0	0	0	0.05	0	0.686	0.689
PPARD	SRC	9606.ENSP00000310928	9606.ENSP00000362680	0	0	0	0	0.065	0.351	0	0.091	0.4
PPARD	PTGS2	9606.ENSP00000310928	9606.ENSP00000356438	0	0	0	0	0	0.05	0	0.41	0.416
PTGES	PTGS2	9606.ENSP00000342385	9606.ENSP00000356438	0	0	0	0	0.063	0	0.9	0.848	0.984
PTGS2	PTPN1	9606.ENSP00000356438	9606.ENSP00000360683	0	0	0	0	0	0.066	0	0.562	0.573
PTGS2	SRC	9606.ENSP00000356438	9606.ENSP00000362680	0	0	0	0	0.061	0.058	0	0.565	0.581
PTK2	PTPN1	9606.ENSP00000341189	9606.ENSP00000360683	0	0	0	0	0.077	0.064	0	0.411	0.446

PTK2	SYK	9606.ENSP00000341189	9606.ENSP00000364907	0	0	0	0.703	0.073	0.433	0.9	0.473	0.95
PTK2	SRC	9606.ENSP00000341189	9606.ENSP00000362680	0	0	0	0.735	0.062	0.626	0.9	0.952	0.971
PTPN1	SRC	9606.ENSP00000360683	9606.ENSP00000362680	0	0	0	0	0.085	0.456	0.9	0.868	0.992
PTPRS	SRC	9606.ENSP00000349932	9606.ENSP00000362680	0	0	0	0	0.062	0.16	0	0.359	0.451
RORC	SRC	9606.ENSP00000327025	9606.ENSP00000362680	0	0	0	0	0	0.379	0	0.149	0.449
SAE1	UBA2	9606.ENSP00000270225	9606.ENSP00000246548	0	0	0	0.58	0.733	0.996	0.9	0.983	0.999
SAE1	TYMS	9606.ENSP00000270225	9606.ENSP00000315644	0	0	0	0	0.175	0.39	0	0.046	0.478
SLC22A12	XDH	9606.ENSP00000366797	9606.ENSP00000368727	0	0	0	0	0.059	0	0	0.484	0.494
SRC	TYMS	9606.ENSP00000362680	9606.ENSP00000315644	0	0	0	0	0.055	0	0	0.567	0.573
SRC	SYK	9606.ENSP00000362680	9606.ENSP00000364907	0	0	0	0.729	0.054	0.393	0.9	0.913	0.953
TNKS	TNKS2	9606.ENSP00000311579	9606.ENSP00000360689	0	0	0	0.982	0.077	0.305	0.9	0.87	0.931
TOP1	TYMS	9606.ENSP00000354522	9606.ENSP00000315644	0	0	0	0	0.042	0	0	0.76	0.761
TOP1	TOP2A	9606.ENSP00000354522	9606.ENSP00000411532	0	0	0	0	0.112	0.925	0.54	0.918	0.997

TOP2A	UBA2	9606.ENSP00000411532	9606.ENSP00000246548	0.043	0	0	0	0.685	0	0	0.292	0.768
TOP2A	TYMS	9606.ENSP00000411532	9606.ENSP00000315644	0.112	0	0	0	0.83	0	0	0.653	0.943
TTR	XDH	9606.ENSP00000237014	9606.ENSP00000368727	0.219	0	0	0	0.114	0	0	0.204	0.401
