# Supplementary material for: Study on the Action Mechanism of the Yifei Jianpi Tongfu Formula in Treatment of Colorectal Cancer Lung Metastasis Based on Network Analysis, Molecular Docking, and Experimental Validation
Source: Evid Based Complement Alternat Med. 2022 Jul 30;2022:6229444. doi: 10.1155/2022/6229444 (PMC9356795; doi:10.1155/2022/6229444)
Supplement: Supplementary Materials — Detailed information about the active compounds and targets identified in YJTF is shown in Supplementary Table 1. All of the disease-related targets for CRC lung metastasis are listed in Supplementary Table 2. Detailed information about the 81 overlapping targets identified as the key targets for studying the therapeutic effect of YJTF on CRC lung metastasis is shown in Supplementary Table 3. Detailed information about the PPI network is shown in Supplementary Table 4. Detailed information about the GO and KEGG enrichment analysis of the putative targets is shown in Supplementary Table 5. [file 6229444.f1.zip › Supplementary Table.5.pdf]

GO-BP

_LogP_MyList	GO	Description	PARENT_GO	LogP	Enrichment	Z-score	#GeneInGO	#GeneInGOAndHitList	%InGO	STDV%InGO	GeneID	Hits	Log(q-value)	GROUP_ID	FirstInGroupByEnrichment	FirstInGroupByLogP	BestLogPInGroup	BestEnrichmentInGroup
-21.7275	GO:0062197	cellular response to chemical stress	19_GO:0050896 response to stimulus	-21.7275	21.53611	20.431111	339	21	25.92593	4.869201	142|207|240|328|383|558|983|1545|1612|1956|4137|4233|4313|4314|4318|4353|4363|4638|5743|6714|8644	PARP1|AKT1|ALOX5|APEX1|ARG1|AXL|CDK1|CYP1B1|DAPK1|EGFR|MAPT|MET|MMP2|MMP3|MMP9|MPO|ABCC1|MYLK|PTGS2|SRC|AKR1C3	-17.5304	1	1	1	-21.7275	31.40104
-20.891	GO:0010035	response to inorganic substance	19_GO:0050896 response to stimulus	-20.891	14.53607	17.59805	574	24	29.62963	5.073592	142|207|246|328|351|383|558|760|891|983|1017|1545|1956|3292|3791|4137|4233|4314|4318|4353|5743|6714|8644|9536	PARP1|AKT1|ALOX15|APEX1|APP|ARG1|AXL|CA2|CCNB1|CDK1|CDK2|CYP1B1|EGFR|HSD17B1|KDR|MAPT|MET|MMP3|MMP9|MPO|PTGS2|SRC|AKR1C3|PTGES	-17.1534	1	0	0	-21.7275	31.40104
-20.8734	GO:0071396	cellular response to lipid	19_GO:0050896 response to stimulus	-20.8734	14.51079	17.58079	575	24	29.62963	5.073592	142|196|207|239|367|383|558|891|1080|1645|1646|1956|2099|2100|2101|2322|2908|4843|5292|5467|6714|6850|8644|10062	PARP1|AHR|AKT1|ALOX12|AR|ARG1|AXL|CCNB1|CFTR|AKR1C1|AKR1C2|EGFR|ESR1|ESR2|ESRRA|FLT3|NR3C1|NOS2|PIM1|PPARD|SRC|SYK|AKR1C3|NR1H3	-17.1534	2	1	1	-20.8734	36.9845
-20.6986	GO:0071407	cellular response to organic cyclic compound	19_GO:0050896 response to stimulus	-20.6986	14.26274	17.4106	585	24	29.62963	5.073592	142|196|207|238|328|351|367|383|891|1080|1545|1956|2099|2100|2101|2322|2908|2932|5292|5294|5467|5743|6714|8644	PARP1|AHR|AKT1|ALK|APEX1|APP|AR|ARG1|CCNB1|CFTR|CYP1B1|EGFR|ESR1|ESR2|ESRRA|FLT3|NR3C1|GSK3B|PIM1|PIK3CG|PPARD|PTGS2|SRC|AKR1C3	-17.1036	2	0	0	-20.8734	36.9845
-20.0839	GO:0034599	cellular response to oxidative stress	19_GO:0050896 response to stimulus	-20.0839	22.77735	20.02	290	19	23.45679	4.708094	142|207|240|328|383|558|983|1545|1612|1956|4137|4233|4313|4314|	PARP1|AKT1|ALOX5|APEX1|ARG1|AXL|CDK1|CYP1B1|DAPK1|EGFR|MAPT|MET|MMP2|MMP3|MMP9|MPO|	-16.5858	1	0	0	-21.7275	31.40104

						147					4318|4353|4363|6714|8644	ABCC1|SRC|AKR1C3						
–19.3412	GO:0032870	cellular response to hormone stimulus	19_GO:0050896 response to stimulus	–19.3412	13.59872	16.58217	588	23	28.39506	5.010146	142|207|328|367|383|760|1645|1646|1956|2099|2100|2101|2322|2908|2932|3480|3643|5295|5467|5747|5770|6714|8644	PARP1|AKT1|APEX1|AR|ARG1|CA2|AKR1C1|AKR1C2|EGFR|ESR1|ESR2|ESRRA|FLT3|NR3C1|GSK3B|IGF1R|INSR|PIK3R1|PPARD|PTK2|PTPN1|SRC|AKR1C3	–15.9222	3	1	1	–19.3412	20.62356
–19.1747	GO:0006979	response to oxidative stress	19_GO:0050896 response to stimulus	–19.1747	16.22387	17.48531	450	21	25.92593	4.869201	142|207|240|328|351|383|558|983|1545|1612|1956|4137|4233|4313|4314|4318|4353|4363|5743|6714|8644	PARP1|AKT1|ALOX5|APEX1|APP|ARG1|AXL|CDK1|CYP1B1|DAPK1|EGFR|MAPT|MET|MMP2|MMP3|MMP9|MPO|ABCC1|PTGS2|SRC|AKR1C3	–15.8228	1	0	0	–21.7275	31.40104
–19.0075	GO:1901699	cellular response to nitrogen compound	19_GO:0050896 response to stimulus	–19.0075	12.05738	15.81801	692	24	29.62963	5.073592	43|142|196|207|238|328|351|383|760|1017|1080|2101|2932|3480|3643|4313|4314|4363|5294|5295|5743|5747|5770|6714	ACHE|PARP1|AHR|AKT1|ALK|APEX1|APP|ARG1|CA2|CDK2|CFTR|ESRRA|GSK3B|IGF1R|INSR|MMP2|MMP3|ABCC1|PIK3CG|PIK3R1|PTGS2|PTK2|PTPN1|SRC	–15.7135	3	0	0	–19.3412	20.62356
–18.0973	GO:1901652	response to peptide	19_GO:0050896 response to stimulus	–18.0973	14.37154	16.33466	508	21	25.92593	4.869201	142|207|328|351|383|760|2101|2932|3480|3643|4313|4314|4318|4321|4322|4363|5295|5743|5747|5770|6714	PARP1|AKT1|APEX1|APP|ARG1|CA2|ESRRA|GSK3B|IGF1R|INSR|MMP2|MMP3|MMP9|MMP12|MMP13|ABCC1|PIK3R1|PTGS2|PTK2|PTPN1|SRC	–14.8544	3	0	0	–19.3412	20.62356
–17.1911	GO:0051347	positive regulation of transferase activity	19_GO:0065007 biological regulation	–17.1911	12.96757	15.40598	563	21	25.92593	4.869201	134|207|238|558|891|1956|2147|2322|3480|3558|3643|3791|4137|4233|5294|5347|5747|5770|6714|6850|9212	ADORA1|AKT1|ALK|AXL|CCNB1|EGFR|F2|FLT3|IGF1R|IL2|INSR|KDR|MAPT|MET|PIK3CG|PLK1|PTK2|PTPN1|SRC|SYK|AURKB	–13.9941	4	1	1	–17.1911	66.21987
–16.83	GO:0043549	regulation of kinase activity	19_GO:0050789 regulation of	–16.83	10.46603	14.	764	23	28.39506	5.010146	134|207|238|351|558|891|1956|	ADORA1|AKT1|ALK|APP|AXL|CCNB1|EGFR|F2|FLT3|	–13.680	4	0	0	–17.	66.21987

59			biological process	59		24726					2147|2322|3156|3480|3558|3643|3791|4137|4233|5294|5295|5347|5747|5770|6714|6850	HMGCR|IGF1R|IL2|INSR|KDR|MAPT|MET|PIK3CG|PIK3R1|PLK1|PTK2|PTPN1|SRC|SYK	2				1911	
-16.7671	GO:0034614	cellular response to reactive oxygen species	19_GO:0050896 response to stimulus	-16.7671	31.40104	20.38434	155	14	17.28395	4.201205	207|328|383|558|983|1545|1956|4137|4233|4313|4318|4353|6714|8644	AKT1|APEX1|ARG1|AXL|CDK1|CYP1B1|EGFR|MAPT|MET|MMP2|MMP9|MPO|SRC|AKR1C3	-13.6492	1	0	0	-21.7275	31.40104
-16.1122	GO:0071417	cellular response to organonitrogen compound	19_GO:0050896 response to stimulus	-16.1122	11.46113	14.34369	637	21	25.92593	4.869201	142|196|207|238|328|351|383|760|1080|2101|2932|3480|3643|4313|4363|5294|5295|5743|5747|5770|6714	PARP1|AHR|AKT1|ALK|APEX1|APP|ARG1|CA2|CFTR|ESRRA|GSK3B|IGF1R|INSR|MMP2|ABCC1|PIK3CG|PIK3R1|PTGS2|PTK2|PTPN1|SRC	-13.0291	3	0	0	-19.3412	20.62356
-15.9956	GO:0033674	positive regulation of kinase activity	19_GO:0048518 positive regulation of biological process	-15.9956	13.76132	15.14584	480	19	23.45679	4.708094	134|207|238|558|891|1956|2147|2322|3480|3558|3643|3791|4137|4233|5294|5747|5770|6714|6850	ADORA1|AKT1|ALK|AXL|CCNB1|EGFR|F2|FLT3|IGF1R|IL2|INSR|KDR|MAPT|MET|PIK3CG|PTK2|PTPN1|SRC|SYK	-12.9446	4	0	0	-17.1911	66.21987
-15.4495	GO:1901654	response to ketone	19_GO:0050896 response to stimulus	-15.4495	25.34979	18.18342	192	14	17.28395	4.201205	142|196|207|367|383|768|1080|1646|1956|2908|6714|7298|8644|10062	PARP1|AHR|AKT1|AR|ARG1|CA9|CFTR|AKR1C2|EGFR|NR3C1|SRC|TYMS|AKR1C3|NR1H3	-12.4285	2	0	0	-20.8734	36.9845
-15.0818	GO:0031347	regulation of defense response	19_GO:0050896 response to stimulus	-15.0818	11.12494	13.74834	625	20	24.69136	4.791289	134|240|246|351|383|1956|2099|3558|4314|4318|4321|4363|5294|5467|5743|5770|6714|6850|9536|10062	ADORA1|ALOX5|ALOX15|APP|ARG1|EGFR|ESR1|IL2|MMP3|MMP9|MMP12|ABCC1|PIK3CG|PPARD|PTGS2|PTPN1|SRC|SYK|PTGES|NR1H3	-12.0888	5	1	1	-15.0818	15.19312
-	GO:000	transmembrane	19_GO:0023052	-	11.05	1	62	20	24.6	4.791	134|207|238|367|	ADORA1|AKT1|ALK|AR|AXL|	-	4	0	0	-	66.21

15.0292	7169	receptor protein tyrosine kinase signaling pathway	signaling	15.0292	419	3.69688	9		9136	289	558|1956|2322|2932|3480|3643|3791|4233|4313|4318|5295|5747|5770|6714|6850|7498	EGFR|FLT3|GSK3B|IGF1R|INSR|KDR|MET|MMP2|MMP9|PIK3R1|PTK2|PTPN1|SRC|SYK|XDH	12.0651				17.1911	987
-15.0069	GO:0050727	regulation of inflammatory response	19_GO:0050896 response to stimulus	-15.0069	15.19312	15.1397	389	17	20.98765	4.52467	134|240|246|351|1956|2099|3558|4314|4318|4363|5294|5467|5743|6714|6850|9536|10062	ADORA1|ALOX5|ALOX15|APP|EGFR|ESR1|IL2|MMP3|MMP9|ABCC1|PIK3CG|PPARD|PTGS2|SRC|SYK|PTGES|NR1H3	-12.0651	5	0	0	-15.0818	15.19312
-14.9128	GO:1904645	response to amyloid-beta	19_GO:0050896 response to stimulus	-14.9128	58.92446	23.92145	59	10	12.34568	3.65512	142|351|2932|3480|4313|4314|4318|4321|4322|4363	PARP1|APP|GSK3B|IGF1R|MMP2|MMP3|MMP9|MMP12|MMP13|ABCC1	-11.9945	6	1	1	-14.9128	124.1623
-14.6455	GO:0048545	response to steroid hormone	19_GO:0050896 response to stimulus	-14.6455	16.40846	15.32968	339	16	19.75309	4.423735	142|367|383|760|1956|2099|2100|2101|2322|2908|5467|5743|6714|7298|8644|10062	PARP1|AR|ARG1|CA2|EGFR|ESR1|ESR2|ESRRA|FLT3|NR3C1|PPARD|PTGS2|SRC|TYMS|AKR1C3|NR1H3	-11.7494	2	0	0	-20.8734	36.9845
-14.5396	GO:0000302	response to reactive oxygen species	19_GO:0050896 response to stimulus	-14.5396	21.82583	16.76697	223	14	17.28395	4.201205	207|328|383|558|983|1545|1956|4137|4233|4313|4318|4353|6714|8644	AKT1|APEX1|ARG1|AXL|CDK1|CYP1B1|EGFR|MAPT|MET|MMP2|MMP9|MPO|SRC|AKR1C3	-11.6648	1	0	0	-21.7275	31.40104
-14.4322	GO:0046777	protein autophosphorylation	19_GO:0008152 metabolic process	-14.4322	21.44124	16.60816	227	14	17.28395	4.201205	207|238|1612|1956|2322|2932|3480|3643|3791|5292|5747|6714|6850|9212	AKT1|ALK|DAPK1|EGFR|FLT3|GSK3B|IGF1R|INSR|KDR|PIM1|PTK2|SRC|SYK|AURKB	-11.5775	4	0	0	-17.1911	66.21987
-14.1215	GO:0072593	reactive oxygen species metabolic process	19_GO:0008152 metabolic process	-14.1215	20.36469	16.1	239	14	17.28395	4.201205	207|239|240|1545|1645|1956|2147|4137|4314|4353|	AKT1|ALOX12|ALOX5|CYP1B1|AKR1C1|EGFR|F2|MAPT|MMP3|MPO|NOS2|	-11.2862	7	1	1	-14.121	57.94239

						4746					4843|6850|7498|8644	SYK|XDH|AKR1C3					5	
-14.0531	GO:0014065	phosphatidylinositol 3-kinase signaling	19_GO:0023052 signaling	-14.0531	29.37924	18.209022	142	12	14.81481	3.947186	207|1956|2147|2322|3480|3643|3791|5294|5295|5467|5747|6714	AKT1|EGFR|F2|FLT3|IGF1R|INSR|KDR|PIK3CG|PIK3R1|PPARD|PTK2|SRC	-11.2363	4	0	0	-17.1911	66.21987
-14.0025	GO:0010942	positive regulation of cell death	19_GO:0048518 positive regulation of biological process	-14.0025	10.6884	13.080004	618	19	23.45679	4.708094	134|142|207|239|383|1509|1545|1612|2932|3156|4137|4314|4318|4843|5743|6714|6850|7153|8644	ADORA1|PARP1|AKT1|ALOX12|ARG1|CTSD|CYP1B1|DAPK1|GSK3B|HMGCR|MAPT|MMP3|MMP9|NOS2|PTGS2|SRC|SYK|TOP2A|AKR1C3	-11.2034	8	1	1	-14.0025	18.03867
-13.6942	GO:0009611	response to wounding	19_GO:0050896 response to stimulus	-13.6942	11.33655	13.17331	552	18	22.22222	4.61933	140|239|240|246|383|558|983|1956|2147|3156|3791|4321|4638|5294|5467|5747|6714|6850	ADORA3|ALOX12|ALOX5|ALOX15|ARG1|AXL|CDK1|EGFR|F2|HMGCR|KDR|MMP12|MYLK|PIK3CG|PPARD|PTK2|SRC|SYK	-10.9121	9	1	1	-13.6942	26.07407
-13.4112	GO:0043491	protein kinase B signaling	19_GO:0023052 signaling	-13.4112	21.41946	15.990052	211	13	16.04938	4.078483	207|558|1646|1956|3480|3643|3791|4233|5294|5747|6714|7498|8644	AKT1|AXL|AKR1C2|EGFR|IGF1R|INSR|KDR|MET|PIK3CG|PTK2|SRC|XDH|AKR1C3	-10.6455	4	0	0	-17.1911	66.21987
-13.3451	GO:0018209	peptidyl-serine modification	19_GO:0008152 metabolic process	-13.3451	15.42845	14.333323	338	15	18.51852	4.316086	142|207|351|891|983|1017|1457|1956|2932|5347|5743|6714|6850|7150|9212	PARP1|AKT1|APP|CCNB1|CDK1|CDK2|CSNK2A1|EGFR|GSK3B|PLK1|PTGS2|SRC|SYK|TOP1|AURKB	-10.5952	10	1	1	-13.3451	41.71852
-13.1836	GO:0006690	icosanoid metabolic process	19_GO:0008152 metabolic process	-13.1836	31.09104	17.96	123	11	13.58025	3.80643	239|240|246|1545|1645|1646|4363|5743|6850|8644|9536	ALOX12|ALOX5|ALOX15|CYP1B1|AKR1C1|AKR1C2|ABCC1|PTGS2|SYK|AKR1C3|PTGES	-10.4489	11	1	1	-13.1836	260.7407

						322												
-13.14	GO:0010817	regulation of hormone levels	19_GO:0065007 biological regulation	-13.14	11.68009	13.02061	506	17	20.98765	4.52467	134|240|1080|1545|1586|1588|1645|1646|1956|2099|3156|3292|4843|5467|7276|8644|57016	ADORA1|ALOX5|CFTR|CYP1B1|CYP17A1|CYP19A1|AKR1C1|AKR1C2|EGFR|ESR1|HMGCR|HSD17B1|NOS2|PPARD|TTR|AKR1C3|AKR1B10	-10.4201	12	1	1	-13.14	260.7407
-12.927	GO:0048015	phosphatidylinositol-mediated signaling	19_GO:0023052 signaling	-12.927	23.7037	16.22776	176	12	14.81481	3.947186	207|1956|2147|2322|3480|3643|3791|5294|5295|5467|5747|6714	AKT1|EGFR|F2|FLT3|IGF1R|INSR|KDR|PIK3CG|PIK3R1|PPARD|PTK2|SRC	-10.2213	4	0	0	-17.1911	66.21987
-12.8948	GO:1901653	cellular response to peptide	19_GO:0050896 response to stimulus	-12.8948	14.36588	13.76614	363	15	18.51852	4.316086	142|207|328|351|383|760|2101|2932|3480|3643|4363|5295|5747|5770|6714	PARP1|AKT1|APEX1|APP|ARG1|CA2|ESRRA|GSK3B|IGF1R|INSR|ABCC1|PIK3R1|PTK2|PTPN1|SRC	-10.2029	3	0	0	-19.3412	20.62356
-12.81	GO:0048017	inositol lipid-mediated signaling	19_GO:0023052 signaling	-12.81	23.17695	16.03152	180	12	14.81481	3.947186	207|1956|2147|2322|3480|3643|3791|5294|5295|5467|5747|6714	AKT1|EGFR|F2|FLT3|IGF1R|INSR|KDR|PIK3CG|PIK3R1|PPARD|PTK2|SRC	-10.1315	4	0	0	-17.1911	66.21987
-12.7896	GO:1901655	cellular response to ketone	19_GO:0050896 response to stimulus	-12.7896	36.9845	18.76936	94	10	12.34568	3.65512	196|207|367|383|1080|1646|1956|2908|6714|8644	AHR|AKT1|AR|ARG1|CFTR|AKR1C2|EGFR|NR3C1|SRC|AKR1C3	-10.1241	2	0	0	-20.8734	36.9845
-12.7441	GO:0048511	rhythmic process	19_GO:0048511 rhythmic process	-12.7441	16.16997	14.21	301	14	17.28395	4.201205	134|196|558|983|1457|1956|2099|2932|4843|6714|7150|7153|7298|	ADORA1|AHR|AXL|CDK1|CSNK2A1|EGFR|ESR1|GSK3B|NOS2|SRC|TOP1|TOP2A|TYMS|NR1H3	-10.0911	13	1	1	-12.7441	16.39879

						168				10062								
-12.724	GO:0010038	response to metal ion	19_GO:0050896 response to stimulus	-12.724	13.98074	13.5548	373	15	18.51852	4.316086	142|207|246|351|383|760|891|983|1956|3292|4137|4318|5743|8644|9536	PARP1|AKT1|ALOX15|APP|ARG1|CA2|CCNB1|CDK1|EGFR|HSD17B1|MAPT|MMP9|PTGS2|AKR1C3|PTGES	-10.0832	14	1	1	-12.724	34.76543
-12.6737	GO:0018108	peptidyl-tyrosine phosphorylation	19_GO:0008152 metabolic process	-12.6737	13.86919	13.49298	376	15	18.51852	4.316086	134|238|351|558|1956|2322|3480|3558|3643|3791|4233|5747|5770|6714|6850	ADORA1|ALK|APP|AXL|EGFR|FLT3|IGF1R|IL2|INSR|KDR|MET|PTK2|PTPN1|SRC|SYK	-10.0448	4	0	0	-17.1911	66.21987
-12.6238	GO:0018212	peptidyl-tyrosine modification	19_GO:0008152 metabolic process	-12.6238	13.75941	13.43187	379	15	18.51852	4.316086	134|238|351|558|1956|2322|3480|3558|3643|3791|4233|5747|5770|6714|6850	ADORA1|ALK|APP|AXL|EGFR|FLT3|IGF1R|IL2|INSR|KDR|MET|PTK2|PTPN1|SRC|SYK	-10.0065	4	0	0	-17.1911	66.21987
-12.4753	GO:0018105	peptidyl-serine phosphorylation	19_GO:0008152 metabolic process	-12.4753	15.4513	13.85316	315	14	17.28395	4.201205	207|351|891|983|1017|1457|1956|2932|5347|5743|6714|6850|7150|9212	AKT1|APP|CCNB1|CDK1|CDK2|CSNK2A1|EGFR|GSK3B|PLK1|PTGS2|SRC|SYK|TOP1|AURKB	-9.86932	10	0	0	-13.3451	41.71852
-12.2065	GO:0009636	response to toxic substance	19_GO:0050896 response to stimulus	-12.2065	17.25002	14.19314	262	13	16.04938	4.078483	196|383|891|983|1545|4353|5243|5292|5743|7298|9429|9536|57016	AHR|ARG1|CCNB1|CDK1|CYP1B1|MPO|ABCB1|PIM1|PTGS2|TYMS|ABCG2|PTGES|AKR1B10	-9.61154	15	1	1	-12.2065	35.47493
-12.1619	GO:0071383	cellular response to steroid hormone stimulus	19_GO:0050896 response to stimulus	-12.1619	20.45025	14.974	204	12	14.81481	3.947186	142|367|383|1956|2099|2100|2101|2322|2908|5467|6714|8644	PARP1|AR|ARG1|EGFR|ESR1|ESR2|ESRRA|FLT3|NR3C1|PPARD|SRC|AKR1C3	-9.57763	2	0	0	-20.8734	36.9845

						87										
-12.1229	GO:0030522	intracellular receptor signaling pathway	19_GO:0023052 signaling	-12.1229	16.99062	14.07379	266	13	16.04938	4.078483	142|196|246|367|2099|2100|2101|2908|5292|5467|6714|8644|10062	PARP1|AHR|ALOX15|AR|ESR1|ESR2|ESRRA|NR3C1|PIM1|PPARD|SRC|AKR1C3|NR1H3	-9.5491	2	0	0	-20.8734	36.9845
-12.0071	GO:2000377	regulation of reactive oxygen species metabolic process	19_GO:0050789 regulation of biological process	-12.0071	24.35795	15.76313	157	11	13.58025	3.80643	207|240|1545|1645|1956|2147|4137|4314|6850|7498|8644	AKT1|ALOX5|CYP1B1|AKR1C1|EGFR|F2|MAPT|MMP3|SYK|XDH|AKR1C3	-9.4435	7	0	0	-14.1215	57.94239
-11.9232	GO:0042060	wound healing	19_GO:0050896 response to stimulus	-11.9232	12.29909	12.59113	424	15	18.51852	4.316086	239|240|246|558|1956|2147|3156|3791|4321|4638|5294|5467|5747|6714|6850	ALOX12|ALOX5|ALOX15|AXL|EGFR|F2|HMGCR|KDR|MMP12|MYLK|PIK3CG|PPARD|PTK2|SRC|SYK	-9.36963	9	0	0	-13.6942	26.07407
-11.7071	GO:0051897	positive regulation of protein kinase B signaling	19_GO:0048518 positive regulation of biological process	-11.7071	28.97119	16.49198	120	10	12.34568	3.65512	558|1646|1956|3480|3643|4233|5294|5747|6714|8644	AXL|AKR1C2|EGFR|IGF1R|INSR|MET|PIK3CG|PTK2|SRC|AKR1C3	-9.16326	4	0	0	-17.1911	66.21987
-11.6706	GO:0120254	olefinic compound metabolic process	19_GO:0008152 metabolic process	-11.6706	28.73176	16.41909	121	10	12.34568	3.65512	239|240|246|1545|1586|1645|1646|3292|5743|8644	ALOX12|ALOX5|ALOX15|CYP1B1|CYP17A1|AKR1C1|AKR1C2|HSD17B1|PTGS2|AKR1C3	-9.13635	11	0	0	-13.1836	260.7407
-11.6357	GO:0071241	cellular response to inorganic substance	19_GO:0050896 response to stimulus	-11.6357	18.45952	14.154	226	12	14.81481	3.947186	142|207|246|351|891|1017|1956|3292|4314|4318|5743|8644	PARP1|AKT1|ALOX15|APP|CCNB1|CDK2|EGFR|HSD17B1|MMP3|MMP9|PTGS2|AKR1C3	-9.11075	14	0	0	-12.724	34.76543

						04												
-11.6266	GO:0001934	positive regulation of protein phosphorylation	19_GO:0048518 positive regulation of biological process	-11.6266	8.548877	11.11479	732	18	22.22222	4.61933	134|207|351|891|1956|2147|2322|3558|3643|3791|4318|5294|5347|5743|5747|5770|6714|6850	ADORA1|AKT1|APP|CCNB1|EGFR|F2|FLT3|IL2|INSR|KDR|MMP9|PIK3CG|PLK1|PTGS2|PTK2|PTPN1|SRC|SYK	-9.11075	4	0	0	-17.1911	66.21987
-11.6102	GO:0071375	cellular response to peptide hormone stimulus	19_GO:0050896 response to stimulus	-11.6102	15.47776	13.356673	292	13	16.04938	4.078483	142|207|328|383|760|2101|2932|3480|3643|5295|5747|5770|6714	PARP1|AKT1|APEX1|ARG1|CA2|ESRRA|GSK3B|IGF1R|INSR|PIK3R1|PTK2|PTPN1|SRC	-9.10334	3	0	0	-19.3412	20.62356
-11.5558	GO:0070201	regulation of establishment of protein localization	19_GO:0051179 localization	-11.5558	10.31998	11.73341	539	16	19.75309	4.423735	43|240|983|1080|1509|1956|2932|3156|4137|4843|5295|5467|5743|5770|6714|10062	ACHE|ALOX5|CDK1|CFTR|CTSD|EGFR|GSK3B|HMGCR|MAPT|NOS2|PIK3R1|PPARD|PTGS2|PTPN1|SRC|NR1H3	-9.05772	16	1	1	-11.5558	31.96821
-11.4322	GO:0030162	regulation of proteolysis	19_GO:0050789 regulation of biological process	-11.4322	8.321513	10.930288	752	18	22.22222	4.61933	207|239|351|1017|1457|1509|1612|2147|2932|4137|4318|5347|5743|5747|6714|6850|7498|10062	AKT1|ALOX12|APP|CDK2|CSNK2A1|CTSD|DAPK1|F2|GSK3B|MAPT|MMP9|PLK1|PTGS2|PTK2|SRC|SYK|XDH|NR1H3	-8.94272	8	0	0	-14.0025	18.03867
-11.2655	GO:0080135	regulation of cellular response to stress	19_GO:0050896 response to stimulus	-11.2655	8.914213	11.075988	663	17	20.98765	4.52467	142|207|240|351|1021|1956|2932|3156|3480|4137|4233|4314|5295|5743|5770|7498|10062	PARP1|AKT1|ALOX5|APP|CDK6|EGFR|GSK3B|HMGCR|IGF1R|MAPT|MET|MMP3|PIK3R1|PTGS2|PTPN1|XDH|NR1H3	-8.78443	17	1	1	-11.2655	11.58848
-11.2269	GO:0051896	regulation of protein kinase B signaling	19_GO:0023052 signaling	-11.2269	20.67134	14.41776	185	11	13.58025	3.80643	558|1646|1956|3480|3643|4233|5294|5747|6714|7498|8644	AXL|AKR1C2|EGFR|IGF1R|INSR|MET|PIK3CG|PTK2|SRC|XDH|AKR1C3	-8.75412	4	0	0	-17.1911	66.21987

						7												
-11.1436	GO:0043410	positive regulation of MAPK cascade	19_GO:0048518 positive regulation of biological process	-11.1436	10.84161	11.69294	481	15	18.51852	4.316086	238|246|351|367|1956|2322|3156|3480|3643|3791|5294|5770|6714|6850|7498	ALK|ALOX15|APP|AR|EGFR|FLT3|HMGCR|IGF1R|INSR|KDR|PIK3CG|PTPN1|SRC|SYK|XDH	-8.67892	4	0	0	-17.1911	66.21987
-11.1274	GO:0034754	cellular hormone metabolic process	19_GO:0008152 metabolic process	-11.1274	25.37623	15.36136	137	10	12.34568	3.65512	1545|1586|1588|1645|1646|2099|3292|7276|8644|57016	CYP1B1|CYP17A1|CYP19A1|AKR1C1|AKR1C2|ESR1|HSD17B1|TTR|AKR1C3|AKR1B10	-8.67073	12	0	0	-13.14	260.7407
-10.9633	GO:0008610	lipid biosynthetic process	19_GO:0008152 metabolic process	-10.9633	8.528317	10.77753	693	17	20.98765	4.52467	43|207|239|240|246|1080|1586|1588|3156|3292|5294|5295|5467|5743|8644|9536|10062	ACHE|AKT1|ALOX12|ALOX5|ALOX15|CFTR|CYP17A1|CYP19A1|HMGCR|HSD17B1|PIK3CG|PIK3R1|PPARD|PTGS2|AKR1C3|PTGES|NR1H3	-8.51446	18	1	1	-10.9633	18.38557
-10.8808	GO:0003013	circulatory system process	19_GO:0032501 multicellular organismal process	-10.8808	9.301788	11.02116	598	16	19.75309	4.423735	134|140|207|367|1956|3156|3558|3643|4363|4843|5243|5294|5467|5743|6714|9429	ADORA1|ADORA3|AKT1|AR|EGFR|HMGCR|IL2|INSR|ABCC1|NOS2|ABCB1|PIK3CG|PPARD|PTGS2|SRC|ABCG2	-8.43966	19	1	1	-10.8808	39.73192
-10.8515	GO:0043434	response to peptide hormone	19_GO:0050896 response to stimulus	-10.8515	11.69991	11.80871	416	14	17.28395	4.201205	142|207|328|383|760|2101|2932|3480|3643|5295|5743|5747|5770|6714	PARP1|AKT1|APEX1|ARG1|CA2|ESRRA|GSK3B|IGF1R|INSR|PIK3R1|PTGS2|PTK2|PTPN1|SRC	-8.41782	3	0	0	-19.3412	20.62356
-10.7972	GO:0007568	aging	19_GO:0032502 developmental process	-10.7972	13.33188	12.2688	339	13	16.04938	4.078483	207|239|328|351|383|983|1017|1021|3156|4353|5743|7298|9212	AKT1|ALOX12|APEX1|APP|ARG1|CDK1|CDK2|CDK6|HMGCR|MPO|PTGS2|TYMS|AURKB	-8.37099	10	0	0	-13.3451	41.71852

						2												
-10.7338	GO:0009411	response to UV	19_GO:0050896 response to stimulus	-10.7338	23.17695	14.62687	150	10	12.34568	3.65512	142|207|1956|4313|4314|4318|5295|5743|7299|9212	PARP1|AKT1|EGFR|MMP2|MMP3|MMP9|PIK3R1|PTGS2|TYR|AURKB	-8.31485	6	0	0	-14.9128	124.1623
-10.701	GO:0032103	positive regulation of response to external stimulus	19_GO:0048518 positive regulation of biological process	-10.701	11.3985	11.62911	427	14	17.28395	4.201205	351|383|1956|2147|3558|3791|4233|4321|4363|5294|5743|5747|6714|6850	APP|ARG1|EGFR|F2|IL2|KDR|MET|MMP12|ABCC1|PIK3CG|PTGS2|PTK2|SRC|SYK	-8.28926	5	0	0	-15.0818	15.19312
-10.6751	GO:0042180	cellular ketone metabolic process	19_GO:0008152 metabolic process	-10.6751	18.38557	13.51681	208	11	13.58025	3.80643	207|1586|1645|1646|3156|3292|5467|5743|8644|10062|57016	AKT1|CYP17A1|AKR1C1|AKR1C2|HMGCR|HSD17B1|PPARD|PTGS2|AKR1C3|NR1H3|AKR1B10	-8.27048	18	0	0	-10.9633	18.38557
-10.5859	GO:0043281	regulation of cysteine-type endopeptidase activity involved in apoptotic process	19_GO:0050789 regulation of biological process	-10.5859	18.03867	13.37484	212	11	13.58025	3.80643	207|239|1457|1509|1612|4137|4318|5743|6714|6850|7498	AKT1|ALOX12|CSNK2A1|CTSD|DAPK1|MAPT|MMP9|PTGS2|SRC|SYK|XDH	-8.18817	8	0	0	-14.0025	18.03867
-10.5403	GO:0014066	regulation of phosphatidylinositol 3-kinase signaling	19_GO:0023052 signaling	-10.5403	28.7054	15.56553	109	9	11.11111	3.491885	1956|2147|2322|3480|3643|3791|5467|5747|6714	EGFR|F2|FLT3|IGF1R|INSR|KDR|PPARD|PTK2|SRC	-8.14941	4	0	0	-17.1911	66.21987
-10.5157	GO:1901615	organic hydroxy compound metabolic process	19_GO:0008152 metabolic process	-10.5157	9.783893	10.99662	533	15	18.51852	4.316086	239|246|351|1080|1545|1588|1645|1646|3156|3292|5467|7276|7299|8644|57016	ALOX12|ALOX15|APP|CFTR|CYP1B1|CYP19A1|AKR1C1|AKR1C2|HMGCR|HSD17B1|PPARD|TTR|TYR|AKR1C3|AKR1B10	-8.13158	12	0	0	-13.14	260.7407

-10.2949	GO:0033559	unsaturated fatty acid metabolic process	19_GO:0008152 metabolic process	-10.2949	26.97318	415.05547	116	9	11.11111	3.491885	239|240|246|1545|1645|1646|5743|8644|9536	ALOX12|ALOX5|ALOX15|CYP1B1|AKR1C1|AKR1C2|PTGS2|AKR1C3|PTGES	-7.91738	11	0	0	-13.1836	260.7407
-10.2592	GO:0030335	positive regulation of cell migration	19_GO:0040011 locomotion	-10.2592	9.379163	10.71797	556	15	18.51852	4.316086	207|239|351|1956|3480|3643|3791|4233|4318|4638|5294|5295|5743|5747|6714	AKT1|ALOX12|APP|EGFR|IGF1R|INSR|KDR|MET|MMP9|MYLK|PIK3CG|PIK3R1|PTGS2|PTK2|SRC	-7.88819	20	1	1	-10.2592	15.71319
-10.2066	GO:0000165	MAPK cascade	19_GO:0023052 signaling	-10.2066	7.625966	10.04625	775	17	20.98765	4.52467	207|238|246|351|367|983|1956|2322|3156|3480|3643|3791|5294|5770|6714|6850|7498	AKT1|ALK|ALOX15|APP|AR|CDK1|EGFR|FLT3|HMGCR|IGF1R|INSR|KDR|PIK3CG|PTPN1|SRC|SYK|XDH	-7.84201	4	0	0	-17.1911	66.21987
-10.1384	GO:0014068	positive regulation of phosphatidylinositol 3-kinase signaling	19_GO:0048518 positive regulation of biological process	-10.1384	35.2055	16.35172	79	8	9.876543	3.314965	2147|2322|3480|3643|3791|5467|5747|6714	F2|FLT3|IGF1R|INSR|KDR|PPARD|PTK2|SRC	-7.78023	4	0	0	-17.1911	66.21987
-10.1125	GO:0043408	regulation of MAPK cascade	19_GO:0023052 signaling	-10.1125	8.252922	10.23641	674	16	19.75309	4.423735	207|238|246|351|367|1956|2322|3156|3480|3643|3791|5294|5770|6714|6850|7498	AKT1|ALK|ALOX15|APP|AR|EGFR|FLT3|HMGCR|IGF1R|INSR|KDR|PIK3CG|PTPN1|SRC|SYK|XDH	-7.76052	4	0	0	-17.1911	66.21987
-10.0464	GO:2000116	regulation of cysteine-type endopeptidase activity	19_GO:0050789 regulation of biological process	-10.0464	16.06806	12.5381	238	11	13.58025	3.80643	207|239|1457|1509|1612|4137|4318|5743|6714|6850|7498	AKT1|ALOX12|CSNK2A1|CTSD|DAPK1|MAPT|MMP9|PTGS2|SRC|SYK|XDH	-7.70058	8	0	0	-14.0025	18.03867

						3												
-9.98291	GO:2000147	positive regulation of cell motility	19_GO:0040011 locomotion	-9.98291	8.960163	10.42227	582	15	18.51852	4.316086	207|239|351|1956|3480|3643|3791|4233|4318|4638|5294|5295|5743|5747|6714	AKT1|ALOX12|APP|EGFR|IGF1R|INSR|KDR|MET|MMP9|MYLK|PIK3CG|PIK3R1|PTGS2|PTK2|SRC	-7.64917	20	0	0	-10.2592	15.71319
-9.98291	GO:0097190	apoptotic signaling pathway	19_GO:0023052 signaling	-9.98291	8.960163	10.42227	582	15	18.51852	4.316086	134|142|207|367|1457|1545|1612|2932|3558|4318|5295|5467|5743|5770|6714	ADORA1|PARP1|AKT1|AR|CSNK2A1|CYP1B1|DAPK1|GSK3B|IL2|MMP9|PIK3R1|PPARD|PTGS2|PTPN1|SRC	-7.64917	21	1	1	-9.98291	27.26701
-9.92073	GO:0042493	response to drug	19_GO:0050896 response to stimulus	-9.92073	11.32708	11.15853	399	13	16.04938	4.078483	328|383|768|891|983|1956|4363|4843|5243|5743|6714|7150|7298	APEX1|ARG1|CA9|CCNB1|CDK1|EGFR|ABCC1|NOS2|ABCB1|PTGS2|SRC|TOP1|TYMS	-7.5929	22	1	1	-9.92073	11.32708
-9.89338	GO:0008202	steroid metabolic process	19_GO:0008152 metabolic process	-9.89338	13.07791	11.65212	319	12	14.81481	3.947186	351|1080|1545|1586|1588|1645|1646|2099|3156|3292|5467|8644	APP|CFTR|CYP1B1|CYP17A1|CYP19A1|AKR1C1|AKR1C2|ESR1|HMGCR|HSD17B1|PPARD|AKR1C3	-7.57139	12	0	0	-13.14	260.7407
-9.86222	GO:0009416	response to light stimulus	19_GO:0050896 response to stimulus	-9.86222	12.99642	11.61015	321	12	14.81481	3.947186	142|207|351|1956|3156|4313|4314|4318|5295|5743|7299|9212	PARP1|AKT1|APP|EGFR|HMGCR|MMP2|MMP3|MMP9|PIK3R1|PTGS2|TYR|AURKB	-7.55058	6	0	0	-14.9128	124.1623
-9.85998	GO:0051272	positive regulation of cellular component movement	19_GO:0048518 positive regulation of biological process	-9.85998	8.77915	10.2919	594	15	18.51852	4.316086	207|239|351|1956|3480|3643|3791|4233|4318|4638|5294|5295|5743|5747|6714	AKT1|ALOX12|APP|EGFR|IGF1R|INSR|KDR|MET|MMP9|MYLK|PIK3CG|PIK3R1|PTGS2|PTK2|SRC	-7.55058	20	0	0	-10.2592	15.71319

						8												
-9.85554	GO:0033002	muscle cell proliferation	19_GO:0009987 cellular process	-9.85554	15.42015	12.25069	248	11	13.58025	3.80643	207|239|891|983|1956|3156|4313|4318|5292|5467|5743	AKT1|ALOX12|CCNB1|CDK1|EGFR|HMGCR|MMP2|MMP9|PIM1|PPARD|PTGS2	-7.55058	19	0	0	-10.8808	39.73192
-9.83976	GO:0040017	positive regulation of locomotion	19_GO:0040011 locomotion	-9.83976	8.749689	10.27062	596	15	18.51852	4.316086	207|239|351|1956|3480|3643|3791|4233|4318|4638|5294|5295|5743|5747|6714	AKT1|ALOX12|APP|EGFR|IGF1R|INSR|KDR|MET|MMP9|MYLK|PIK3CG|PIK3R1|PTGS2|PTK2|SRC	-7.54032	20	0	0	-10.2592	15.71319
-9.79625	GO:1900182	positive regulation of protein localization to nucleus	19_GO:0048518 positive regulation of biological process	-9.79625	31.96821	15.53788	87	8	9.876543	3.314965	142|207|983|2147|5295|5347|5743|6714	PARP1|AKT1|CDK1|F2|PIK3R1|PLK1|PTGS2|SRC	-7.50228	16	0	0	-11.5558	31.96821
-9.78876	GO:0046683	response to organophosphorus	19_GO:0050896 response to stimulus	-9.78876	23.7037	14.04261	132	9	11.11111	3.491885	196|328|351|1080|1645|5294|5743|7298|7299	AHR|APEX1|APP|CFTR|AKR1C1|PIK3CG|PTGS2|TYMS|TYR	-7.50018	23	1	1	-9.78876	32.19021
-9.73962	GO:0019216	regulation of lipid metabolic process	19_GO:0050789 regulation of biological process	-9.73962	12.6804	11.44594	329	12	14.81481	3.947186	134|207|238|2147|2322|5294|5467|5743|5747|6714|8644|10062	ADORA1|AKT1|ALK|F2|FLT3|PIK3CG|PPARD|PTGS2|PTK2|SRC|AKR1C3|NR1H3	-7.45637	19	0	0	-10.8808	39.73192
-9.67556	GO:0048871	multicellular organismal homeostasis	19_GO:0032501 multicellular organismal process	-9.67556	9.524776	10.4451	511	14	17.28395	4.201205	43|134|238|239|760|1080|1956|3480|4233|5743|6714|6850|9536|10062	ACHE|ADORA1|ALK|ALOX12|CA2|CFTR|EGFR|IGF1R|MET|PTGS2|SRC|SYK|PTGES|NR1H3	-7.39966	9	0	0	-13.6942	26.07407

-9.67244	GO:1900180	regulation of protein localization to nucleus	19_GO:0051179 localization	-9.67244	23.00654	313.81708	136	9	11.11111	3.491885	142|207|983|2147|2932|5295|5347|5743|6714	PARP1|AKT1|CDK1|F2|GSK3B|PIK3R1|PLK1|PTGS2|SRC	-7.39966	16	0	0	-11.5558	31.96821
-9.65495	GO:0045927	positive regulation of growth	19_GO:0040007 growth	-9.65495	14.76524	11.9532	259	11	13.58025	3.80643	207|891|983|1457|1956|2147|3558|3643|4137|5292|5467	AKT1|CCNB1|CDK1|CSNK2A1|EGFR|F2|IL2|INSR|MAPT|PIM1|PPARD	-7.38731	24	1	1	-9.65495	14.76524
-9.64244	GO:0051223	regulation of protein transport	19_GO:0051179 localization	-9.64244	9.469184	10.408	514	14	17.28395	4.201205	43|240|983|1080|1956|2932|3156|4843|5295|5467|5743|5770|6714|10062	ACHE|ALOX5|CDK1|CFTR|EGFR|GSK3B|HMGCR|NOS2|PIK3R1|PPARD|PTGS2|PTPN1|SRC|NR1H3	-7.37988	16	0	0	-11.5558	31.96821
-9.58428	GO:0003018	vascular process in circulatory system	19_GO:0032501 multicellular organismal process	-9.58428	14.54068	11.84949	263	11	13.58025	3.80643	134|207|1956|3156|3643|4363|5243|5467|5743|6714|9429	ADORA1|AKT1|EGFR|HMGCR|INSR|ABCC1|ABCB1|PPARD|PTGS2|SRC|ABCG2	-7.32674	19	0	0	-10.8808	39.73192
-9.56467	GO:0071248	cellular response to metal ion	19_GO:0050896 response to stimulus	-9.56467	17.64743	12.59356	197	10	12.34568	3.65512	142|207|246|351|891|1956|3292|4318|5743|8644	PARP1|AKT1|ALOX15|APP|CCNB1|EGFR|HSD17B1|MMP9|PTGS2|AKR1C3	-7.3121	14	0	0	-12.724	34.76543
-9.48567	GO:0002683	negative regulation of immune system process	19_GO:0002376 immune system process	-9.48567	10.43766	10.6296	433	13	16.04938	4.078483	134|196|207|246|383|558|1021|3558|4321|5295|6714|6850|10062	ADORA1|AHR|AKT1|ALOX15|ARG1|AXL|CDK6|IL2|MMP12|PIK3R1|SRC|SYK|NR1H3	-7.238	25	1	1	-9.48567	22.67311
-9.46756	GO:0070141	response to UV-A	19_GO:0050896 response to stimulus	-9.46756	124.1623	24.7	14	5	6.17284	2.674018	207|1956|4313|4314|4318	AKT1|EGFR|MMP2|MMP3|MMP9	-7.22474	6	0	0	-14.912	124.1623

						5675											8	
-9.42683	GO:0030155	regulation of cell adhesion	19_GO:0022610 biological adhesion	-9.42683	7.40675	9.558097	751	16	19.75309	4.423735	207|239|240|246|383|1021|1545|2932|3558|3791|4321|5294|5295|5747|6714|6850	AKT1|ALOX12|ALOX5|ALOX15|ARG1|CDK6|CYP1B1|GSK3B|IL2|KDR|MMP12|PIK3CG|PIK3R1|PTK2|SRC|SYK	-7.18881	25	0	0	-9.48567	22.67311
-9.38519	GO:0030855	epithelial cell differentiation	19_GO:0032502 developmental process	-9.38519	8.110132	9.795868	643	15	18.51852	4.316086	207|367|983|1021|1645|1646|2099|2932|3791|4233|4318|6714|7298|7498|8644	AKT1|AR|CDK1|CDK6|AKR1C1|AKR1C2|ESR1|GSK3B|KDR|MET|MMP9|SRC|TYMS|XDH|AKR1C3	-7.15192	26	1	1	-9.38519	8.110132
-9.36233	GO:1903829	positive regulation of cellular protein localization	19_GO:0048518 positive regulation of biological process	-9.36233	13.85579	11.52752	276	11	13.58025	3.80643	142|207|983|1956|2147|2932|4137|5295|5347|5743|6714	PARP1|AKT1|CDK1|EGFR|F2|GSK3B|MAPT|PIK3R1|PLK1|PTGS2|SRC	-7.13376	16	0	0	-11.5558	31.96821
-9.31819	GO:0045834	positive regulation of lipid metabolic process	19_GO:0048518 positive regulation of biological process	-9.31819	20.99925	13.14628	149	9	11.11111	3.491885	134|207|2147|2322|5467|5743|5747|6714|10062	ADORA1|AKT1|F2|FLT3|PPARD|PTGS2|PTK2|SRC|NR1H3	-7.09426	19	0	0	-10.8808	39.73192
-9.25372	GO:0007623	circadian rhythm	19_GO:0048511 rhythmic process	-9.25372	16.39879	12.08758	212	10	12.34568	3.65512	134|196|983|1956|2932|4843|7150|7153|7298|10062	ADORA1|AHR|CDK1|EGFR|GSK3B|NOS2|TOP1|TOP2A|TYMS|NR1H3	-7.03439	13	0	0	-12.7441	16.39879
-9.22084	GO:0040008	regulation of growth	19_GO:0040007 growth	-9.22084	7.889281	9.62	661	15	18.51852	4.316086	207|351|367|891|983|1457|1956|2100|2147|2932|	AKT1|APP|AR|CCNB1|CDK1|CSNK2A1|EGFR|ESR2|F2|GSK3B|IL2|INSR|MAPT|	-7.00605	24	0	0	-9.6549	14.76524

						6679					3558|3643|4137|5292|5467	PIM1|PPARD					5	
-9.21012	GO:0043068	positive regulation of programmed cell death	19_GO:0048518 positive regulation of biological process	-9.21012	8.769659	9.9929214	555	14	17.28395	4.201205	207|239|383|1509|1545|1612|3156|4318|4843|5743|6714|6850|7153|8644	AKT1|ALOX12|ARG1|CTSD|CYP1B1|DAPK1|HMGCR|MMP9|NOS2|PTGS2|SRC|SYK|TOP2A|AKR1C3	-6.99983	8	0	0	-14.0025	18.03867
-9.16905	GO:0032963	collagen metabolic process	19_GO:0008152 metabolic process	-9.16905	26.74264	14.12593	104	8	9.876543	3.314965	383|2147|4313|4314|4318|4321|4322|5467	ARG1|F2|MMP2|MMP3|MMP9|MMP12|MMP13|PPARD	-6.96322	6	0	0	-14.9128	124.1623
-9.15141	GO:0034504	protein localization to nucleus	19_GO:0051179 localization	-9.15141	13.23252	11.22661	289	11	13.58025	3.80643	142|207|983|2147|2932|4321|5295|5347|5743|6714|6850	PARP1|AKT1|CDK1|F2|GSK3B|MMP12|PIK3R1|PLK1|PTGS2|SRC|SYK	-6.94998	16	0	0	-11.5558	31.96821
-9.1359	GO:0042445	hormone metabolic process	19_GO:0008152 metabolic process	-9.1359	15.94745	11.89945	218	10	12.34568	3.65512	1545|1586|1588|1645|1646|2099|3292|7276|8644|57016	CYP1B1|CYP17A1|CYP19A1|AKR1C1|AKR1C2|ESR1|HSD17B1|TTR|AKR1C3|AKR1B10	-6.93884	12	0	0	-13.14	260.7407
-9.07912	GO:0045862	positive regulation of proteolysis	19_GO:0048518 positive regulation of biological process	-9.07912	11.09535	10.58469	376	12	14.81481	3.947186	207|239|351|1509|1612|2932|4137|5347|5747|6714|6850|7498	AKT1|ALOX12|APP|CTSD|DAPK1|GSK3B|MAPT|PLK1|PTK2|SRC|SYK|XDH	-6.88638	8	0	0	-14.0025	18.03867
-9.00133	GO:0050878	regulation of body fluid levels	19_GO:0065007 biological regulation	-9.00133	10.92108	10.4	382	12	14.81481	3.947186	134|239|558|1080|1956|2147|4233|5294|6714|6850|	ADORA1|ALOX12|AXL|CFTR|EGFR|F2|MET|PIK3CG|SRC|SYK|XDH|NR1H3	-6.81287	9	0	0	-13.694	26.07407

						8577					7498|10062						2	
-8.92369	GO:0043269	regulation of ion transport	19_GO:0051179 localization	-8.92369	7.503331	9.323924	695	15	18.51852	4.316086	134|207|351|383|760|1080|1612|2147|4318|4638|5243|5294|5743|6850|9536	ADORA1|AKT1|APP|ARG1|CA2|CFTR|DAPK1|F2|MMP9|MYLK|ABCB1|PIK3CG|PTGS2|SYK|PTGES	-6.7404	27	1	1	-8.92369	24.25495
-8.92043	GO:0042886	amide transport	19_GO:0051179 localization	-8.92043	12.5796	10.902663	304	11	13.58025	3.80643	134|240|760|1080|1956|3156|4363|4843|5243|5467|9429	ADORA1|ALOX5|CA2|CFTR|EGFR|HMGCR|ABCC1|NOS2|ABCB1|PPARD|ABCG2	-6.7404	28	1	1	-8.92043	22.1907
-8.89967	GO:0006631	fatty acid metabolic process	19_GO:0008152 metabolic process	-8.89967	10.69706	10.357725	390	12	14.81481	3.947186	207|239|240|246|1545|1645|1646|5467|5743|8644|9536|10062	AKT1|ALOX12|ALOX5|ALOX15|CYP1B1|AKR1C1|AKR1C2|PPARD|PTGS2|AKR1C3|PTGES|NR1H3	-6.7238	11	0	0	-13.1836	260.7407
-8.87872	GO:0031960	response to corticosteroid	19_GO:0050896 response to stimulus	-8.87872	18.73586	12.346656	167	9	11.11111	3.491885	142|383|1956|2322|2908|5743|6714|7298|8644	PARP1|ARG1|EGFR|FLT3|NR3C1|PTGS2|SRC|TYMS|AKR1C3	-6.70697	29	1	1	-8.87872	35.96424
-8.82125	GO:0032787	monocarboxylic acid metabolic process	19_GO:0008152 metabolic process	-8.82125	8.180102	9.507589	595	14	17.28395	4.201205	207|239|240|246|351|1545|1645|1646|3643|5467|5743|8644|9536|10062	AKT1|ALOX12|ALOX5|ALOX15|APP|CYP1B1|AKR1C1|AKR1C2|INSR|PPARD|PTGS2|AKR1C3|PTGES|NR1H3	-6.65357	11	0	0	-13.1836	260.7407
-8.78901	GO:0006720	isoprenoid metabolic process	19_GO:0008152 metabolic process	-8.78901	23.97616	13.3	116	8	9.876543	3.314965	1545|1645|1956|3156|5467|7276|8644|57016	CYP1B1|AKR1C1|EGFR|HMGCR|PPARD|TTR|AKR1C3|AKR1B10	-6.62537	12	0	0	-13.14	260.7407

						1824												
-8.76561	GO:0007584	response to nutrient	19_GO:0050896 response to stimulus	-8.76561	18.19121	12.146333	172	9	11.11111	3.491885	383|1956|3156|5292|5467|5743|7298|7299|8644	ARG1|EGFR|HMGCR|PIM1|PPARD|PTGS2|TYMS|TYR|AKR1C3	-6.60598	30	1	1	-8.76561	29.79894
-8.71526	GO:0006801	superoxide metabolic process	19_GO:0008152 metabolic process	-8.71526	32.88622	14.75143	74	7	8.641975	3.122032	207|239|1956|4137|4353|4843|6850	AKT1|ALOX12|EGFR|MAPT|MPO|NOS2|SYK	-6.55959	7	0	0	-14.1215	57.94239
-8.69747	GO:0009991	response to extracellular stimulus	19_GO:0050896 response to stimulus	-8.69747	8.985102	9.705596	503	13	16.04938	4.078483	207|383|558|1956|3156|4353|5292|5467|5743|6714|7298|7299|8644	AKT1|ARG1|AXL|EGFR|HMGCR|MPO|PIM1|PPARD|PTGS2|SRC|TYMS|TYR|AKR1C3	-6.54573	30	0	0	-8.76561	29.79894
-8.64333	GO:0001503	ossification	19_GO:0032501 multicellular organismal process	-8.64333	10.15049	10.036695	411	12	14.81481	3.947186	43|207|240|246|1021|1956|2101|4313|4318|4322|5743|5747	ACHE|AKT1|ALOX5|ALOX15|CDK6|EGFR|ESRRA|MMP2|MMP9|MMP13|PTGS2|PTK2	-6.49548	31	1	1	-8.64333	10.15049
-8.63274	GO:2000379	positive regulation of reactive oxygen species metabolic process	19_GO:0048518 positive regulation of biological process	-8.63274	32.02079	14.544422	76	7	8.641975	3.122032	1645|1956|2147|4137|6850|7498|8644	AKR1C1|EGFR|F2|MAPT|SYK|XDH|AKR1C3	-6.48876	7	0	0	-14.1215	57.94239
-8.54943	GO:0032102	negative regulation of response to external stimulus	19_GO:0048519 negative regulation of	-8.54943	9.956687	9.92	419	12	14.81481	3.947186	134|239|240|383|2147|3156|3558|4321|5467|6714|	ADORA1|ALOX12|ALOX5|ARG1|F2|HMGCR|IL2|MMP12|PPARD|SRC|SYK|	-6.40928	25	0	0	-9.4856	22.67311

			biological process			0959					6850|10062	NR1H3					7	
-8.52802	GO:0097305	response to alcohol	19_GO:0050896 response to stimulus	-8.52802	13.79581	10.95882	252	10	12.34568	3.65512	142|196|207|983|1080|1646|3156|3558|7298|8644	PARP1|AHR|AKT1|CDK1|CFTR|AKR1C2|HMGCR|IL2|TYMS|AKR1C3	-6.39166	32	1	1	-8.52802	13.79581
-8.47999	GO:0062012	regulation of small molecule metabolic process	19_GO:0050789 regulation of biological process	-8.47999	11.41552	10.300221	335	11	13.58025	3.80643	142|207|351|2932|3643|4843|5467|5743|6714|8644|10062	PARP1|AKT1|APP|GSK3B|INSR|NOS2|PPARD|PTGS2|SRC|AKR1C3|NR1H3	-6.34739	19	0	0	-10.8808	39.73192
-8.45485	GO:1903827	regulation of cellular protein localization	19_GO:0051179 localization	-8.45485	8.575913	9.429455	527	13	16.04938	4.078483	142|207|367|983|1956|2147|2932|4137|5295|5347|5743|5770|6714	PARP1|AKT1|AR|CDK1|EGFR|F2|GSK3B|MAPT|PIK3R1|PLK1|PTGS2|PTPN1|SRC	-6.32597	16	0	0	-11.5558	31.96821
-8.37439	GO:0045859	regulation of protein kinase activity	19_GO:0050789 regulation of biological process	-8.37439	7.545985	9.032967	645	14	17.28395	4.201205	134|207|351|891|1956|2322|3156|3643|5294|5347|5747|5770|6714|6850	ADORA1|AKT1|APP|CCNB1|EGFR|FLT3|HMGCR|INSR|PIK3CG|PLK1|PTK2|PTPN1|SRC|SYK	-6.24921	4	0	0	-17.1911	66.21987
-8.36743	GO:0052548	regulation of endopeptidase activity	19_GO:0050789 regulation of biological process	-8.36743	9.590464	9.69808	435	12	14.81481	3.947186	207|239|351|1457|1509|1612|4137|4318|5743|6714|6850|7498	AKT1|ALOX12|APP|CSNK2A1|CTSD|DAPK1|MAPT|MMP9|PTGS2|SRC|SYK|XDH	-6.24592	8	0	0	-14.0025	18.03867
-8.24035	GO:0043401	steroid hormone mediated signaling pathway	19_GO:0023052 signaling	-8.24035	20.45025	12.21	136	8	9.876543	3.314965	142|367|2099|2100|2101|2908|5467|6714	PARP1|AR|ESR1|ESR2|ESRRA|NR3C1|PPARD|SRC	-6.12247	2	0	0	-20.8734	36.9845

						209												
-8.22491	GO:0022411	cellular component disassembly	19_GO:0009987 cellular process	-8.22491	9.312169	9.525324	448	12	14.81481	3.947186	983|2932|3480|3643|4313|4314|4318|4321|4322|5295|5347|7153	CDK1|GSK3B|IGF1R|INSR|MMP2|MMP3|MMP9|MMP12|MMP13|PIK3R1|PLK1|TOP2A	-6.11064	6	0	0	-14.9128	124.1623
-8.21676	GO:0016101	diterpenoid metabolic process	19_GO:0008152 metabolic process	-8.21676	27.97219	13.53293	87	7	8.641975	3.122032	1545|1645|1956|5467|7276|8644|57016	CYP1B1|AKR1C1|EGFR|PPARD|TTR|AKR1C3|AKR1B10	-6.10961	12	0	0	-13.14	260.7407
-8.21676	GO:0044070	regulation of anion transport	19_GO:0051179 localization	-8.21676	27.97219	13.53293	87	7	8.641975	3.122032	134|383|760|1080|5243|6850|9536	ADORA1|ARG1|CA2|CFTR|ABCB1|SYK|PTGES	-6.10961	33	1	1	-8.21676	32.59259
-8.16849	GO:0010631	epithelial cell migration	19_GO:0040011 locomotion	-8.16849	10.65236	9.885778	359	11	13.58025	3.80643	207|239|1545|3791|4233|4318|5294|5467|5743|5747|6714	AKT1|ALOX12|CYP1B1|KDR|MET|MMP9|PIK3CG|PPARD|PTGS2|PTK2|SRC	-6.06485	20	0	0	-10.2592	15.71319
-8.13686	GO:0032940	secretion by cell	19_GO:0051179 localization	-8.13686	6.559515	8.540496	795	15	18.51852	4.316086	43|134|240|558|1080|1588|1956|2932|3156|4843|5294|5467|6850|9536|10062	ACHE|ADORA1|ALOX5|AXL|CFTR|CYP19A1|EGFR|GSK3B|HMGCR|NOS2|PIK3CG|PPARD|SYK|PTGES|NR1H3	-6.03905	28	0	0	-8.92043	22.1907
-8.13506	GO:0031349	positive regulation of defense response	19_GO:0048518 positive regulation of biological process	-8.13506	12.5507	10.37	277	10	12.34568	3.65512	351|383|1956|3558|4321|4363|5294|5743|6714|6850	APP|ARG1|EGFR|IL2|MMP12|ABCC1|PIK3CG|PTGS2|SRC|SYK	-6.03905	5	0	0	-15.0818	15.19312

						621												
-8.13116	GO:0090132	epithelium migration	19_GO:0032501 multicellular organismal process	-8.13116	10.56408	9.836738	362	11	13.58025	3.80643	207|239|1545|3791|4233|4318|5294|5467|5743|5747|6714	AKT1|ALOX12|CYP1B1|KDR|MET|MMP9|PIK3CG|PPARD|PTGS2|PTK2|SRC	-6.03905	20	0	0	-10.2592	15.71319
-8.1289	GO:0009314	response to radiation	19_GO:0050896 response to stimulus	-8.1289	9.128779	9.409798	457	12	14.81481	3.947186	142|207|351|1956|3156|4313|4314|4318|5295|5743|7299|9212	PARP1|AKT1|APP|EGFR|HMGCR|MMP2|MMP3|MMP9|PIK3R1|PTGS2|TYR|AURKB	-6.03905	6	0	0	-14.9128	124.1623
-8.10153	GO:0044597	daunorubicin metabolic process	19_GO:0008152 metabolic process	-8.10153	154.513	24.73887	9	4	4.938272	2.407398	1645|1646|8644|57016	AKR1C1|AKR1C2|AKR1C3|AKR1B10	-6.01835	12	0	0	-13.14	260.7407
-8.09757	GO:0032869	cellular response to insulin stimulus	19_GO:0050896 response to stimulus	-8.09757	15.26287	11.00815	205	9	11.11111	3.491885	142|207|2101|2932|3480|3643|5295|5770|6714	PARP1|AKT1|ESRRA|GSK3B|IGF1R|INSR|PIK3R1|PTPN1|SRC	-6.01835	3	0	0	-19.3412	20.62356
-8.09757	GO:0071902	positive regulation of protein serine/threonine kinase activity	19_GO:0048518 positive regulation of biological process	-8.09757	15.26287	11.00815	205	9	11.11111	3.491885	207|891|1956|2322|3643|5294|5770|6714|6850	AKT1|CCNB1|EGFR|FLT3|INSR|PIK3CG|PTPN1|SRC|SYK	-6.01835	4	0	0	-17.1911	66.21987
-8.09484	GO:1903530	regulation of secretion by cell	19_GO:0051179 localization	-8.09484	7.999126	9.026	565	13	16.04938	4.078483	43|134|240|1080|1588|1956|2932|3156|4843|5467|6850|9536|10062	ACHE|ADORA1|ALOX5|CFTR|CYP19A1|EGFR|GSK3B|HMGCR|NOS2|PPARD|SYK|PTGES|NR1H3	-6.01835	28	0	0	-8.92043	22.1907

						37											
-8.08192	GO:0071900	regulation of protein serine/threonine kinase activity	19_GO:0050789 regulation of biological process	-8.08192	10.44863	9.772238	366	11	13.58025	3.80643	207|891|1956|2322|3156|3643|5294|5347|5770|6714|6850	AKT1|CCNB1|EGFR|FLT3|HMGCR|INSR|PIK3CG|PLK1|PTPN1|SRC|SYK	-6.01253	4	0	0	-17.1911	66.21987
-8.07926	GO:0034644	cellular response to UV	19_GO:0050896 response to stimulus	-8.07926	26.74264	13.21054	91	7	8.641975	3.122032	142|4313|4314|4318|5295|5743|9212	PARP1|MMP2|MMP3|MMP9|PIK3R1|PTGS2|AURKB	-6.01253	6	0	0	-14.9128	124.1623
-8.07926	GO:0033273	response to vitamin	19_GO:0050896 response to stimulus	-8.07926	26.74264	13.21054	91	7	8.641975	3.122032	383|1956|5292|5467|5743|7298|7299	ARG1|EGFR|PIM1|PPARD|PTGS2|TYMS|TYR	-6.01253	30	0	0	-8.76561	29.79894
-8.0697	GO:0090130	tissue migration	19_GO:0032501 multicellular organismal process	-8.0697	10.42016	9.756269	367	11	13.58025	3.80643	207|239|1545|3791|4233|4318|5294|5467|5743|5747|6714	AKT1|ALOX12|CYP1B1|KDR|MET|MMP9|PIK3CG|PPARD|PTGS2|PTK2|SRC	-6.00811	20	0	0	-10.2592	15.71319
-8.06845	GO:0062013	positive regulation of small molecule metabolic process	19_GO:0048518 positive regulation of biological process	-8.06845	19.44919	11.87941	143	8	9.876543	3.314965	207|351|3643|4843|5467|5743|6714|10062	AKT1|APP|INSR|NOS2|PPARD|PTGS2|SRC|NR1H3	-6.00811	19	0	0	-10.8808	39.73192
-8.05566	GO:0052547	regulation of peptidase activity	19_GO:0050789 regulation of biological process	-8.05566	8.99106	9.3221	464	12	14.81481	3.947186	207|239|351|1457|1509|1612|4137|4318|5743|6714|6850|7498	AKT1|ALOX12|APP|CSNK2A1|CTSD|DAPK1|MAPT|MMP9|PTGS2|SRC|SYK|XDH	-5.99848	8	0	0	-14.0025	18.03867

-7.97344	GO:0031667	response to nutrient levels	19_GO:0050896 response to stimulus	-7.97344	8.838669	39.224182	472	12	14.81481	3.947186	207|383|1956|3156|4353|5292|5467|5743|6714|7298|7299|8644	AKT1|ARG1|EGFR|HMGCR|MPO|PIM1|PPARD|PTGS2|SRC|TYMS|TYR|AKR1C3	-5.9194	30	0	0	-8.76561	29.79894
-7.88464	GO:0006721	terpenoid metabolic process	19_GO:0008152 metabolic process	-7.88464	25.08846	12.76404	97	7	8.641975	3.122032	1545|1645|1956|5467|7276|8644|57016	CYP1B1|AKR1C1|EGFR|PPARD|TTR|AKR1C3|AKR1B10	-5.8389	12	0	0	-13.14	260.7407
-7.88063	GO:0030638	polyketide metabolic process	19_GO:0008152 metabolic process	-7.88063	139.0617	23.45278	10	4	4.938272	2.407398	1645|1646|8644|57016	AKR1C1|AKR1C2|AKR1C3|AKR1B10	-5.8389	12	0	0	-13.14	260.7407
-7.88063	GO:0030647	aminoglycoside antibiotic metabolic process	19_GO:0008152 metabolic process	-7.88063	139.0617	23.45278	10	4	4.938272	2.407398	1645|1646|8644|57016	AKR1C1|AKR1C2|AKR1C3|AKR1B10	-5.8389	12	0	0	-13.14	260.7407
-7.88063	GO:0044598	doxorubicin metabolic process	19_GO:0008152 metabolic process	-7.88063	139.0617	23.45278	10	4	4.938272	2.407398	1645|1646|8644|57016	AKR1C1|AKR1C2|AKR1C3|AKR1B10	-5.8389	12	0	0	-13.14	260.7407
-7.85346	GO:0070301	cellular response to hydrogen peroxide	19_GO:0050896 response to stimulus	-7.85346	24.83245	12.6935	98	7	8.641975	3.122032	328|383|558|983|1545|4233|6714	APEX1|ARG1|AXL|CDK1|CYP1B1|MET|SRC	-5.81476	1	0	0	-21.7275	31.40104

-7.73825	GO:0015849	organic acid transport	19_GO:0051179 localization	-7.73825	11.3985	49.806848	305	10	12.34568	3.65512	134|207|383|1645|4363|5243|5467|6850|9429|9536	ADORA1|AKT1|ARG1|AKR1C1|ABCC1|ABCB1|PPARD|SYK|ABCG2|PTGES	-5.70255	33	0	0	-8.21676	32.59259
-7.72779	GO:0019369	arachidonic acid metabolic process	19_GO:0008152 metabolic process	-7.72779	35.35468	14.187668	59	6	7.407407	2.909904	239|240|246|1545|5743|8644	ALOX12|ALOX5|ALOX15|CYP1B1|PTGS2|AKR1C3	-5.69508	11	0	0	-13.1836	260.7407
-7.70805	GO:0006869	lipid transport	19_GO:0051179 localization	-7.70805	9.608537	9.289788	398	11	13.58025	3.80643	207|1080|1588|1645|4363|4843|5243|5467|6850|9536|10062	AKT1|CFTR|CYP19A1|AKR1C1|ABCC1|NOS2|ABCB1|PPARD|SYK|PTGES|NR1H3	-5.67831	33	0	0	-8.21676	32.59259
-7.6969	GO:0045860	positive regulation of protein kinase activity	19_GO:0048518 positive regulation of biological process	-7.6969	9.584455	9.275598	399	11	13.58025	3.80643	134|207|891|1956|2322|3643|5294|5747|5770|6714|6850	ADORA1|AKT1|CCNB1|EGFR|FLT3|INSR|PIK3CG|PTK2|PTPN1|SRC|SYK	-5.67011	4	0	0	-17.1911	66.21987
-7.67319	GO:0048661	positive regulation of smooth muscle cell proliferation	19_GO:0048518 positive regulation of biological process	-7.67319	23.39981	12.29916	104	7	8.641975	3.122032	207|239|1956|3156|4313|4318|5743	AKT1|ALOX12|EGFR|HMGCR|MMP2|MMP9|PTGS2	-5.64932	19	0	0	-10.8808	39.73192
-7.63905	GO:0019748	secondary metabolic process	19_GO:0008152 metabolic process	-7.63905	34.19551	13.939888	61	6	7.407407	2.909904	1545|1645|1646|7299|8644|57016	CYP1B1|AKR1C1|AKR1C2|TYR|AKR1C3|AKR1B10	-5.61808	12	0	0	-13.14	260.7407

-7.59584	GO:0060135	maternal process involved in female pregnancy	19_GO:0051704 multi-organism process	-7.59584	33.64397	13.82042	62	6	7.407407	2.909904	207|367|383|2099|5467|5743	AKT1|AR|ARG1|ESR1|PPARD|PTGS2	-5.5795	19	0	0	-10.8808	39.73192
-7.59471	GO:0051046	regulation of secretion	19_GO:0051179 localization	-7.59471	7.254424	8.478779	623	13	16.04938	4.078483	43|134|240|1080|1588|1956|2932|3156|4843|5467|6850|9536|10062	ACHE|ADORA1|ALOX5|CFTR|CYP19A1|EGFR|GSK3B|HMGCR|NOS2|PPARD|SYK|PTGES|NR1H3	-5.5795	28	0	0	-8.92043	22.1907
-7.5751	GO:0008015	blood circulation	19_GO:0032501 multicellular organismal process	-7.5751	8.132265	8.756336	513	12	14.81481	3.947186	134|140|207|367|1956|3156|3558|4843|5294|5467|5743|6714	ADORA1|ADORA3|AKT1|AR|EGFR|HMGCR|IL2|NOS2|PIK3CG|PPARD|PTGS2|SRC	-5.56273	19	0	0	-10.8808	39.73192
-7.56608	GO:0051129	negative regulation of cellular component organization	19_GO:0048519 negative regulation of biological process	-7.56608	6.506899	8.198754	748	14	17.28395	4.201205	142|207|351|367|891|2932|4137|4233|5295|5347|6714|7153|9212|10062	PARP1|AKT1|APP|AR|CCNB1|GSK3B|MAPT|MET|PIK3R1|PLK1|SRC|TOP2A|AURKB|NR1H3	-5.55654	34	1	1	-7.56608	35.65685
-7.56218	GO:0048589	developmental growth	19_GO:0040007 growth	-7.56218	7.208144	8.443628	627	13	16.04938	4.078483	207|351|367|891|983|2099|2932|3643|4137|4322|5292|5467|7298	AKT1|APP|AR|CCNB1|CDK1|ESR1|GSK3B|INSR|MAPT|MMP13|PIM1|PPARD|TYMS	-5.55545	35	1	1	-7.56218	38.62826
-7.5116	GO:0070542	response to fatty acid	19_GO:0050896 response to stimulus	-7.5116	32.59259	13.58981	64	6	7.407407	2.909904	891|1645|1646|5743|6714|8644	CCNB1|AKR1C1|AKR1C2|PTGS2|SRC|AKR1C3	-5.50767	12	0	0	-13.14	260.7407

-7.42818	GO:0071216	cellular response to biotic stimulus	19_GO:0050896 response to stimulus	-7.42818	12.77098	9.938869	245	9	11.11111	3.491885	207|383|558|2932|4843|5467|6714|6850|10062	AKT1|ARG1|AXL|GSK3B|NOS2|PPARD|SRC|SYK|NR1H3	-5.42952	19	0	0	-10.8808	39.73192
-7.42792	GO:0044772	mitotic cell cycle phase transition	19_GO:0009987 cellular process	-7.42792	9.019334	8.936338	424	11	13.58025	3.80643	207|328|351|891|983|1017|1021|1956|5243|5347|9212	AKT1|APEX1|APP|CCNB1|CDK1|CDK2|CDK6|EGFR|ABCB1|PLK1|AURKB	-5.42952	10	0	0	-13.3451	41.71852
-7.41617	GO:0048638	regulation of developmental growth	19_GO:0040007 growth	-7.41617	10.53498	9.357944	330	10	12.34568	3.65512	207|351|367|891|983|2932|3643|4137|5292|5467	AKT1|APP|AR|CCNB1|CDK1|GSK3B|INSR|MAPT|PIM1|PPARD	-5.4205	35	0	0	-7.56218	38.62826
-7.39607	GO:0043406	positive regulation of MAP kinase activity	19_GO:0048518 positive regulation of biological process	-7.39607	21.3472	11.69181	114	7	8.641975	3.122032	1956|2322|3643|5294|5770|6714|6850	EGFR|FLT3|INSR|PIK3CG|PTPN1|SRC|SYK	-5.40313	4	0	0	-17.1911	66.21987
-7.37932	GO:0071214	cellular response to abiotic stimulus	19_GO:0050896 response to stimulus	-7.37932	10.44007	9.307315	333	10	12.34568	3.65512	142|207|1956|4313|4314|4318|4638|5295|5743|9212	PARP1|AKT1|EGFR|MMP2|MMP3|MMP9|MYLK|PIK3R1|PTGS2|AURKB	-5.39178	6	0	0	-14.9128	124.1623
-7.37932	GO:0104004	cellular response to environmental stimulus	19_GO:0050896 response to stimulus	-7.37932	10.44007	9.307315	333	10	12.34568	3.65512	142|207|1956|4313|4314|4318|4638|5295|5743|9212	PARP1|AKT1|EGFR|MMP2|MMP3|MMP9|MYLK|PIK3R1|PTGS2|AURKB	-5.39178	6	0	0	-14.9128	124.1623

-7.35125	GO:0046686	response to cadmium ion	19_GO:0050896 response to stimulus	-7.35125	30.67538	13.15889	68	6	7.407407	2.909904	207|383|983|1956|4318|8644	AKT1|ARG1|CDK1|EGFR|MMP9|AKR1C3	-5.36638	14	0	0	-12.724	34.76543
-7.34456	GO:0010634	positive regulation of epithelial cell migration	19_GO:0040011 locomotion	-7.34456	15.71319	10.546446	177	8	9.876543	3.314965	207|239|3791|4233|4318|5294|5743|6714	AKT1|ALOX12|KDR|MET|MMP9|PIK3CG|PTGS2|SRC	-5.36499	20	0	0	-10.2592	15.71319
-7.34456	GO:0048771	tissue remodeling	19_GO:0032501 multicellular organismal process	-7.34456	15.71319	10.546446	177	8	9.876543	3.314965	383|558|760|1956|3558|4313|6714|6850	ARG1|AXL|CA2|EGFR|IL2|MMP2|SRC|SYK	-5.36499	36	1	1	-7.34456	54.89279
-7.31428	GO:0002521	leukocyte differentiation	19_GO:0002376 immune system process	-7.31428	7.697144	8.455776	542	12	14.81481	3.947186	142|351|558|760|1021|2101|2322|3558|4318|5295|6714|6850	PARP1|APP|AXL|CA2|CDK6|ESRRA|FLT3|IL2|MMP9|PIK3R1|SRC|SYK	-5.33733	37	1	1	-7.31428	16.2963
-7.29241	GO:0008286	insulin receptor signaling pathway	19_GO:0023052 signaling	-7.29241	20.62356	11.47294	118	7	8.641975	3.122032	207|2932|3480|3643|5295|5770|6714	AKT1|GSK3B|IGF1R|INSR|PIK3R1|PTPN1|SRC	-5.31807	3	0	0	-19.3412	20.62356
-7.288	GO:0048660	regulation of smooth muscle cell proliferation	19_GO:0050789 regulation of biological process	-7.288	15.4513	10.44671	180	8	9.876543	3.314965	207|239|1956|3156|4313|4318|5467|5743	AKT1|ALOX12|EGFR|HMGCR|MMP2|MMP9|PPARD|PTGS2	-5.31625	19	0	0	-10.8808	39.73192
-	GO:003	response to	19_GO:0044419	-	10.16	9.	34	10	12.3	3.655	207|383|558|4353|	AKT1|ARG1|AXL|MPO|NOS2|	-	19	0	0	-	39.73

<table>
<tr><th></th><th></th><th></th><th></th><th></th><th></th><th></th><th></th><th></th><th></th><th></th><th></th><th></th><th></th><th></th><th></th><th></th><th></th><th></th></tr>
<tr><td>7.27094</td><td>2496</td><td>lipopolysaccharide</td><td>biological process involved in interspecies interaction between organisms</td><td>7.27094</td><td>533</td><td>159221</td><td>2</td><td></td><td>4568</td><td>12</td><td>4843|5467|5743|6714|9536|10062</td><td>PPARD|PTGES2|SRC|PTGES|NR1H3</td><td>5.30276</td><td></td><td></td><td></td><td>10.8808</td><td>192</td></tr>
<tr><td>-7.26937</td><td>GO:0043405</td><td>regulation of MAP kinase activity</td><td>19_GO:0050789 regulation of biological process</td><td>-7.26937</td><td>15.36594</td><td>10.414</td><td>181</td><td>8</td><td>9.876543</td><td>3.314965</td><td>1956|2322|3156|3643|5294|5770|6714|6850</td><td>EGFR|FLT3|HMGCR|INSR|PIK3CG|PTPN1|SRC|SYK</td><td>-5.30276</td><td>4</td><td>0</td><td>0</td><td>-17.1911</td><td>66.21987</td></tr>
<tr><td>-7.2588</td><td>GO:0050801</td><td>ion homeostasis</td><td>19_GO:0065007 biological regulation</td><td>-7.2588</td><td>6.145405</td><td>7.888981</td><td>792</td><td>14</td><td>17.28395</td><td>4.201205</td><td>134|142|239|351|760|1080|1956|2099|2147|3558|3577|3791|5294|6714</td><td>ADORA1|PARP1|ALOX12|APP|CA2|CFTR|EGFR|ESR1|F2|IL2|CXCR1|KDR|PIK3CG|SRC</td><td>-5.29473</td><td>38</td><td>1</td><td>1</td><td>-7.2588</td><td>41.71852</td></tr>
<tr><td>-7.23584</td><td>GO:0031348</td><td>negative regulation of defense response</td><td>19_GO:0048519 negative regulation of biological process</td><td>-7.23584</td><td>12.12748</td><td>9.643822</td><td>258</td><td>9</td><td>11.11111</td><td>3.491885</td><td>134|240|383|3558|4321|5467|6714|6850|10062</td><td>ADORA1|ALOX5|ARG1|IL2|MMP12|PPARD|SRC|SYK|NR1H3</td><td>-5.27431</td><td>25</td><td>0</td><td>0</td><td>-9.48567</td><td>22.67311</td></tr>
<tr><td>-7.21414</td><td>GO:0048659</td><td>smooth muscle cell proliferation</td><td>19_GO:0009987 cellular process</td><td>-7.21414</td><td>15.11541</td><td>10.31739</td><td>184</td><td>8</td><td>9.876543</td><td>3.314965</td><td>207|239|1956|3156|4313|4318|5467|5743</td><td>AKT1|ALOX12|EGFR|HMGCR|MMP2|MMP9|PPARD|PTGS2</td><td>-5.25689</td><td>19</td><td>0</td><td>0</td><td>-10.8808</td><td>39.73192</td></tr>
<tr><td>-7.21173</td><td>GO:0001817</td><td>regulation of cytokine production</td><td>19_GO:0032501 multicellular organismal process</td><td>-7.21173</td><td>6.091565</td><td>7.841859</td><td>799</td><td>14</td><td>17.28395</td><td>4.201205</td><td>240|351|383|558|1545|2147|3558|4321|4843|5294|5295|5743|6714|6850</td><td>ALOX5|APP|ARG1|AXL|CYP1B1|F2|IL2|MMP12|NOS2|PIK3CG|PIK3R1|PTGS2|SRC|SYK</td><td>-5.25689</td><td>5</td><td>0</td><td>0</td><td>-15.0818</td><td>15.19312</td></tr>
<tr><td>-7.21092</td><td>GO:0044283</td><td>small molecule biosynthetic process</td><td>19_GO:0008152 metabolic process</td><td>-7.21092</td><td>7.530419</td><td>8.33</td><td>554</td><td>12</td><td>14.81481</td><td>3.947186</td><td>239|240|246|1080|3156|3292|4353|5743|6850|8644|</td><td>ALOX12|ALOX5|ALOX15|CFTR|HMGCR|HSD17B1|MPO|PTGS2|SYK|AKR1C3|</td><td>-5.25689</td><td>11</td><td>0</td><td>0</td><td>-13.183</td><td>260.7407</td></tr>
</table>

						7857					9536|10062	PTGES|NR1H3					6	
-7.18878	GO:0006066	alcohol metabolic process	19_GO:0008152 metabolic process	-7.18878	9.961442	9.047792	349	10	12.34568	3.65512	351|1080|1545|1645|1646|3156|5467|7276|8644|57016	APP|CFTR|CYP1B1|AKR1C1|AKR1C2|HMGCR|PPARD|TTR|AKR1C3|AKR1B10	-5.23724	12	0	0	-13.14	260.7407
-7.18577	GO:0010876	lipid localization	19_GO:0051179 localization	-7.18577	8.536155	8.636051	448	11	13.58025	3.80643	207|1080|1588|1645|4363|4843|5243|5467|6850|9536|10062	AKT1|CFTR|CYP19A1|AKR1C1|ABCC1|NOS2|ABCB1|PPARD|SYK|PTGES|NR1H3	-5.23668	33	0	0	-8.21676	32.59259
-7.16624	GO:0050673	epithelial cell proliferation	19_GO:0009987 cellular process	-7.16624	8.498217	8.612045	450	11	13.58025	3.80643	207|240|367|383|1021|1956|2099|3791|4321|5467|7498	AKT1|ALOX5|AR|ARG1|CDK6|EGFR|ESR1|KDR|MMP12|PPARD|XDH	-5.2196	39	1	1	-7.16624	9.980507
-7.15413	GO:0010639	negative regulation of organelle organization	19_GO:0048519 negative regulation of biological process	-7.15413	9.876543	9.000997	352	10	12.34568	3.65512	142|207|891|4137|4233|5295|5347|6714|7153|9212	PARP1|AKT1|CCNB1|MAPT|MET|PIK3R1|PLK1|SRC|TOP2A|AURKB	-5.21121	34	0	0	-7.56608	35.65685
-7.15212	GO:1901661	quinone metabolic process	19_GO:0008152 metabolic process	-7.15212	46.98031	15.0315	37	5	6.17284	2.674018	1645|1646|3156|8644|57016	AKR1C1|AKR1C2|HMGCR|AKR1C3|AKR1B10	-5.21121	12	0	0	-13.14	260.7407
-7.1506	GO:0015833	peptide transport	19_GO:0051179 localization	-7.1506	11.85185	9.514	264	9	11.1111	3.491885	134|240|760|1080|1956|3156|4363|4843|5467	ADORA1|ALOX5|CA2|CFTR|EGFR|HMGCR|ABCC1|NOS2|PPARD	-5.21121	28	0	0	-8.92043	22.1907

						701												
-7.14375	GO:0071482	cellular response to light stimulus	19_GO:0050896 response to stimulus	-7.14375	19.62565	11.16412	124	7	8.641975	3.122032	142|4313|4314|4318|5295|5743|9212	PARP1|MMP2|MMP3|MMP9|PIK3R1|PTGS2|AURKB	-5.20676	6	0	0	-14.9128	124.1623
-7.12867	GO:0051881	regulation of mitochondrial membrane potential	19_GO:0065007 biological regulation	-7.12867	28.18819	12.578	74	6	7.407407	2.909904	142|207|239|3791|4137|6714	PARP1|AKT1|ALOX12|KDR|MAPT|SRC	-5.19407	40	1	1	-7.12867	115.8848
-7.12265	GO:0032868	response to insulin	19_GO:0050896 response to stimulus	-7.12265	11.76274	9.472587	266	9	11.11111	3.491885	142|207|2101|2932|3480|3643|5295|5770|6714	PARP1|AKT1|ESRRA|GSK3B|IGF1R|INSR|PIK3R1|PTPN1|SRC	-5.1904	3	0	0	-19.3412	20.62356
-7.10652	GO:0009755	hormone-mediated signaling pathway	19_GO:0023052 signaling	-7.10652	14.63808	10.13081	190	8	9.876543	3.314965	142|367|2099|2100|2101|2908|5467|6714	PARP1|AR|ESR1|ESR2|ESRRA|NR3C1|PPARD|SRC	-5.17663	2	0	0	-20.8734	36.9845
-7.09183	GO:0071398	cellular response to fatty acid	19_GO:0050896 response to stimulus	-7.09183	45.74399	14.82394	38	5	6.17284	2.674018	891|1645|1646|6714|8644	CCNB1|AKR1C1|AKR1C2|SRC|AKR1C3	-5.16428	12	0	0	-13.14	260.7407
-7.08615	GO:0071363	cellular response to growth factor stimulus	19_GO:0050896 response to stimulus	-7.08615	6.559515	7.93541	689	13	16.04938	4.078483	142|207|351|383|1956|2322|2908|3791|4137|5747|5770|6714|7498	PARP1|AKT1|APP|ARG1|EGFR|FLT3|NR3C1|KDR|MAPT|PTK2|PTPN1|SRC|XDH	-5.16094	29	0	0	-8.87872	35.96424

						5												
-7.05408	GO:0007565	female pregnancy	19_GO:0051704 multi-organism process	-7.05408	14.41054	10.04066	193	8	9.876543	3.314965	207|367|383|2099|4313|4318|5467|5743	AKT1|AR|ARG1|ESR1|MMP2|MMP9|PPARD|PTGS2	-5.13118	19	0	0	-10.8808	39.73192
-7.04885	GO:0001952	regulation of cell-matrix adhesion	19_GO:0022610 biological adhesion	-7.04885	19.01235	10.97005	128	7	8.641975	3.122032	1021|2932|3791|4321|5295|5747|6714	CDK6|GSK3B|KDR|MMP12|PIK3R1|PTK2|SRC	-5.12825	41	1	1	-7.04885	38.62826
-7.04092	GO:0002237	response to molecule of bacterial origin	19_GO:0044419 biological process involved in interspecies interaction between organisms	-7.04092	9.603711	8.848988	362	10	12.34568	3.65512	207|383|558|4353|4843|5467|5743|6714|9536|10062	AKT1|ARG1|AXL|MPO|NOS2|PPARD|PTGS2|SRC|PTGES|NR1H3	-5.12753	19	0	0	-10.8808	39.73192
-7.03849	GO:2001301	lipoxin biosynthetic process	19_GO:0008152 metabolic process	-7.03849	260.7407	27.90261	4	3	3.703704	2.098362	239|240|246	ALOX12|ALOX5|ALOX15	-5.12753	11	0	0	-13.1836	260.7407
-7.03849	GO:0009753	response to jasmonic acid	19_GO:0050896 response to stimulus	-7.03849	260.7407	27.90261	4	3	3.703704	2.098362	1645|1646|8644	AKR1C1|AKR1C2|AKR1C3	-5.12753	12	0	0	-13.14	260.7407
-7.03849	GO:0071395	cellular response to jasmonic acid stimulus	19_GO:0050896 response to stimulus	-7.03849	260.7407	27.9026	4	3	3.703704	2.098362	1645|1646|8644	AKR1C1|AKR1C2|AKR1C3	-5.12753	12	0	0	-13.14	260.7407

-7.03679	GO:0050777	negative regulation of immune response	19_GO:0002376 immune system process	-7.03679	14.33626	110.01106	194	8	9.876543	3.314965	196|246|383|3558|4321|6714|6850|10062	AHR|ALOX15|ARG1|IL2|MMP12|SRC|SYK|NR1H3	-5.12753	25	0	0	-9.48567	22.67311
-7.02283	GO:0023061	signal release	19_GO:0051179 localization	-7.02283	8.224081	8.436624	465	11	13.58025	3.80643	134|240|1080|1588|1956|2932|3156|4843|5467|6850|9536	ADORA1|ALOX5|CFTR|CYP19A1|EGFR|GSK3B|HMGCR|NOS2|PPARD|SYK|PTGES	-5.1158	28	0	0	-8.92043	22.1907
-7.01869	GO:0031589	cell-substrate adhesion	19_GO:0022610 biological adhesion	-7.01869	9.550943	8.819295	364	10	12.34568	3.65512	246|558|1021|2932|3791|4321|5295|5467|5747|6714	ALOX15|AXL|CDK6|GSK3B|KDR|MMP12|PIK3R1|PPARD|PTK2|SRC	-5.11389	41	0	0	-7.04885	38.62826
-7.00258	GO:0043467	regulation of generation of precursor metabolites and energy	19_GO:0050789 regulation of biological process	-7.00258	18.71985	10.87628	130	7	8.641975	3.122032	207|351|891|983|2932|3643|4843	AKT1|APP|CCNB1|CDK1|GSK3B|INSR|NOS2	-5.09999	35	0	0	-7.56218	38.62826
-6.98731	GO:0044057	regulation of system process	19_GO:0032501 multicellular organismal process	-6.98731	7.180468	8.085008	581	12	14.81481	3.947186	134|140|142|207|351|1956|3156|3558|5294|5743|6714|10062	ADORA1|ADORA3|PARP1|AKT1|APP|EGFR|HMGCR|IL2|PIK3CG|PTGS2|SRC|NR1H3	-5.08692	19	0	0	-10.8808	39.73192
-6.93111	GO:0050900	leukocyte migration	19_GO:0040011 locomotion	-6.93111	9.345546	8.70277	372	10	12.34568	3.65512	134|207|240|351|3577|4318|5294|5747|6714|6850	ADORA1|AKT1|ALOX5|APP|CXCR1|MMP9|PIK3CG|PTK2|SRC|SYK	-5.0329	42	1	1	-6.93111	31.60494

						8										
-6.91539	GO:0034762	regulation of transmembrane transport	19_GO:0051179 localization	-6.91539	7.070935	8.004303	590	12	14.81481	3.947186	207|351|760|1080|1612|2147|3643|4318|5243|5292|5294|5295	AKT1|APP|CA2|CFTR|DAPK1|F2|INSR|MMP9|ABCB1|PIM1|PIK3CG|PIK3R1	-5.01936	27	0	0	-8.92369	24.25495
-6.90266	GO:0001667	ameboidal-type cell migration	19_GO:0040011 locomotion	-6.90266	8.000413	8.290853	478	11	13.58025	3.80643	207|239|1545|3791|4233|4318|5294|5467|5743|5747|6714	AKT1|ALOX12|CYP1B1|KDR|MET|MMP9|PIK3CG|PPARD|PTGS2|PTK2|SRC	-5.01016	20	0	0	-10.2592	15.71319
-6.90187	GO:0090087	regulation of peptide transport	19_GO:0051179 localization	-6.90187	13.76849	9.781882	202	8	9.876543	3.314965	134|240|760|1080|1956|3156|4843|5467	ADORA1|ALOX5|CA2|CFTR|EGFR|HMGCR|NOS2|PPARD	-5.01016	28	0	0	-8.92043	22.1907
-6.89211	GO:0001523	retinoid metabolic process	19_GO:0008152 metabolic process	-6.89211	25.75217	11.98189	81	6	7.407407	2.909904	1545|1645|5467|7276|8644|57016	CYP1B1|AKR1C1|PPARD|TTR|AKR1C3|AKR1B10	-5.00426	12	0	0	-13.14	260.7407
-6.89169	GO:0051345	positive regulation of hydrolase activity	19_GO:0065007 biological regulation	-6.89169	7.035163	7.977778	593	12	14.81481	3.947186	207|239|351|1509|1612|1956|2099|2932|4137|6850|7498|10062	AKT1|ALOX12|APP|CTSD|DAPK1|EGFR|ESR1|GSK3B|MAPT|SYK|XDH|NR1H3	-5.00426	8	0	0	-14.0025	18.03867
-6.86663	GO:0030574	collagen catabolic process	19_GO:0008152 metabolic process	-6.86663	41.38742	14.0682	42	5	6.17284	2.674018	4313|4314|4318|4321|4322	MMP2|MMP3|MMP9|MMP12|MMP13	-4.98301	6	0	0	-14.9128	124.1623

-6.8662	GO:0070848	response to growth factor	19_GO:0050896 response to stimulus	-6.8662	6.277092	17.704229	720	13	16.04938	4.078483	142|207|351|383|1956|2322|2908|3791|4137|5747|5770|6714|7498	PARP1|AKT1|APP|ARG1|EGFR|FLT3|NR3C1|KDR|MAPT|PTK2|PTPN1|SRC|XDH	-4.98301	29	0	0	-8.87872	35.96424
-6.84643	GO:0000086	G2/M transition of mitotic cell cycle	19_GO:0009987 cellular process	-6.84643	17.76336	10.5639	137	7	8.641975	3.122032	351|891|983|1017|5243|5347|9212	APP|CCNB1|CDK1|CDK2|ABCB1|PLK1|AURKB	-4.96534	34	0	0	-7.56608	35.65685
-6.83293	GO:0042448	progesterone metabolic process	19_GO:0008152 metabolic process	-6.83293	81.80102	17.898849	17	4	4.938272	2.407398	1586|1645|1646|8644	CYP17A1|AKR1C1|AKR1C2|AKR1C3	-4.95393	12	0	0	-13.14	260.7407
-6.78846	GO:0002573	myeloid leukocyte differentiation	19_GO:0002376 immune system process	-6.78846	13.30734	9.591777	209	8	9.876543	3.314965	142|351|760|1021|2101|4318|5295|6714	PARP1|APP|CA2|CDK6|ESRRA|MMP9|PIK3R1|SRC	-4.91155	37	0	0	-7.31428	16.2963
-6.76848	GO:0009896	positive regulation of catabolic process	19_GO:0048518 positive regulation of biological process	-6.76848	7.756993	8.129357	493	11	13.58025	3.80643	134|207|351|1457|1612|2932|3643|3791|5347|5747|5770	ADORA1|AKT1|APP|CSNK2A1|DAPK1|GSK3B|INSR|KDR|PLK1|PTK2|PTPN1	-4.89383	43	1	1	-6.76848	45.34622
-6.76632	GO:0010632	regulation of epithelial cell migration	19_GO:0040011 locomotion	-6.76632	10.6788	8.944799	293	9	11.11111	3.491885	207|239|3791|4233|4318|5294|5743|5747|6714	AKT1|ALOX12|KDR|MET|MMP9|PIK3CG|PTGS2|PTK2|SRC	-4.89383	20	0	0	-10.2592	15.71319

-6.76251	GO:0042554	superoxide anion generation	19_GO:0008152 metabolic process	-6.76251	39.50617	13.72904	44	5	6.17284	2.674018	207|239|1956|4137|6850	AKT1|ALOX12|EGFR|MAPT|SYK	-4.89383	7	0	0	-14.1215	57.94239
-6.76251	GO:0002066	columnar/cuboidal epithelial cell development	19_GO:0032502 developmental process	-6.76251	39.50617	13.72904	44	5	6.17284	2.674018	207|1021|2932|6714|7298	AKT1|CDK6|GSK3B|SRC|TYMS	-4.89383	43	0	0	-6.76848	45.34622
-6.7312	GO:0042176	regulation of protein catabolic process	19_GO:0050789 regulation of biological process	-6.7312	8.891415	8.439631	391	10	12.34568	3.65512	207|1017|1457|1956|2932|3156|4843|4953|5347|5747	AKT1|CDK2|CSNK2A1|EGFR|GSK3B|HMGCR|NOS2|ODC1|PLK1|PTK2	-4.86456	43	0	0	-6.76848	45.34622
-6.72098	GO:0050678	regulation of epithelial cell proliferation	19_GO:0050789 regulation of biological process	-6.72098	8.868733	8.426279	392	10	12.34568	3.65512	207|240|367|383|1021|1956|3791|4321|5467|7498	AKT1|ALOX5|AR|ARG1|CDK6|EGFR|KDR|MMP12|PPARD|XDH	-4.85636	39	0	0	-7.16624	9.980507
-6.70994	GO:0030100	regulation of endocytosis	19_GO:0051179 localization	-6.70994	12.99642	9.461481	214	8	9.876543	3.314965	246|351|558|3643|5770|6714|6850|10062	ALOX15|APP|AXL|INSR|PTPN1|SRC|SYK|NR1H3	-4.84733	44	1	1	-6.70994	13.24397
-6.69202	GO:1901990	regulation of mitotic cell cycle phase transition	19_GO:0050789 regulation of biological process	-6.69202	10.46451	8.836815	299	9	11.11111	3.491885	207|328|351|891|983|1017|1956|5347|9212	AKT1|APEX1|APP|CCNB1|CDK1|CDK2|EGFR|PLK1|AURKB	-4.83142	10	0	0	-13.3451	41.71852

-6.65776	GO:0042542	response to hydrogen peroxide	19_GO:0050896 response to stimulus	-6.65776	16.66836	10.19466	146	7	8.641975	3.122032	328|383|558|983|1545|4233|6714	APEX1|ARG1|AXL|CDK1|CYP1B1|MET|SRC	-4.79916	1	0	0	-21.7275	31.40104
-6.65547	GO:0051222	positive regulation of protein transport	19_GO:0048518 positive regulation of biological process	-6.65547	10.36056	8.783965	302	9	11.11111	3.491885	43|983|1080|1956|2932|5295|5467|5743|6714	ACHE|CDK1|CFTR|EGFR|GSK3B|PIK3R1|PPARD|PTGS2|SRC	-4.79886	16	0	0	-11.5558	31.96821
-6.64338	GO:0007162	negative regulation of cell adhesion	19_GO:0022610 biological adhesion	-6.64338	10.32637	8.766513	303	9	11.11111	3.491885	207|239|383|1545|3558|4321|5295|5747|6714	AKT1|ALOX12|ARG1|CYP1B1|IL2|MMP12|PIK3R1|PTK2|SRC	-4.78879	25	0	0	-9.48567	22.67311
-6.64146	GO:2001300	lipoxin metabolic process	19_GO:0008152 metabolic process	-6.64146	208.5926	24.93328	5	3	3.703704	2.098362	239|240|246	ALOX12|ALOX5|ALOX15	-4.78879	11	0	0	-13.1836	260.7407
-6.63034	GO:0006820	anion transport	19_GO:0051179 localization	-6.63034	7.513158	7.964443	509	11	13.58025	3.80643	134|383|760|1080|1645|4363|4843|5243|6850|9429|9536	ADORA1|ARG1|CA2|CFTR|AKR1C1|ABCC1|NOS2|ABCB1|SYK|ABCG2|PTGES	-4.77963	33	0	0	-8.21676	32.59259
-6.6183	GO:0044706	multi-multicellular organism process	19_GO:0051704 multi-organism process	-6.6183	12.64198	9.31076	220	8	9.876543	3.314965	207|367|383|2099|4313|4318|5467|5743	AKT1|AR|ARG1|ESR1|MMP2|MMP9|PPARD|PTGS2	-4.77072	19	0	0	-10.8808	39.73192
-	GO:004	cell cycle G2/M phase	19_GO:0009987	-	16.44	1	14	7	8.64	3.122	351|891|983|1017|	APP|CCNB1|CDK1|CDK2|	-	34	0	0	-	35.65

6.61753	4839	transition	cellular process	6.61753	311	0.11705	8		1975	032	5243|5347|9212	ABCB1|PLK1|AURKB	4.77072				7.56608	685
-6.60329	GO:0010810	regulation of cell-substrate adhesion	19_GO:0022610 biological adhesion	-6.60329	12.58477	9.28621	221	8	9.876543	3.314965	246|1021|2932|3791|4321|5295|5747|6714	ALOX15|CDK6|GSK3B|KDR|MMP12|PIK3R1|PTK2|SRC	-4.75841	41	0	0	-7.04885	38.62826
-6.59763	GO:0014074	response to purine-containing compound	19_GO:0050896 response to stimulus	-6.59763	16.33275	10.07881	149	7	8.641975	3.122032	196|328|351|1080|5294|5743|7299	AHR|APEX1|APP|CFTR|PIK3CG|PTGS2|TYR	-4.75468	23	0	0	-9.78876	32.19021
-6.59545	GO:0001666	response to hypoxia	19_GO:0050896 response to stimulus	-6.59545	10.19182	8.697512	307	9	11.11111	3.491885	134|207|768|891|4313|4843|5467|5743|6714	ADORA1|AKT1|CA9|CCNB1|MMP2|NOS2|PPARD|PTGS2|SRC	-4.75441	19	0	0	-10.8808	39.73192
-6.58945	GO:2001251	negative regulation of chromosome organization	19_GO:0048519 negative regulation of biological process	-6.58945	22.92226	11.25001	91	6	7.407407	2.909904	142|891|5347|6714|7153|9212	PARP1|CCNB1|PLK1|SRC|TOP2A|AURKB	-4.75032	34	0	0	-7.56608	35.65685
-6.57174	GO:0009914	hormone transport	19_GO:0051179 localization	-6.57174	10.12585	8.663486	309	9	11.11111	3.491885	134|240|1080|1588|1956|3156|4843|5467|7276	ADORA1|ALOX5|CFTR|CYP19A1|EGFR|HMGCR|NOS2|PPARD|TTR	-4.73451	28	0	0	-8.92043	22.1907
-6.568	GO:1904646	cellular response to amyloid-beta	19_GO:0050896 response to	-6.568	36.21399	13.	48	5	6.17284	2.674018	142|351|2932|3480|4363	PARP1|APP|GSK3B|IGF1R|ABCC1	-4.7333	45	1	1	-6.5	52.14815

<table>
<tr><td>67</td><td></td><td></td><td>stimulus</td><td>67</td><td></td><td>11446</td><td></td><td></td><td></td><td></td><td></td><td></td><td>3</td><td></td><td></td><td></td><td>6867</td><td></td></tr>
<tr><td>-6.55869</td><td>GO:2001234</td><td>negative regulation of apoptotic signaling pathway</td><td>19_GO:0048519 negative regulation of biological process</td><td>-6.55869</td><td>12.41623</td><td>9.213502</td><td>224</td><td>8</td><td>9.876543</td><td>3.314965</td><td>207|367|1457|2932|4318|5743|5770|6714</td><td>AKT1|AR|CSNK2A1|GSK3B|MMP9|PTGS2|PTPN1|SRC</td><td>-4.72525</td><td>21</td><td>0</td><td>0</td><td>-9.98291</td><td>27.26701</td></tr>
<tr><td>-6.55477</td><td>GO:0044770</td><td>cell cycle phase transition</td><td>19_GO:0009987 cellular process</td><td>-6.55477</td><td>7.382621</td><td>7.874799</td><td>518</td><td>11</td><td>13.58025</td><td>3.80643</td><td>207|328|351|891|983|1017|1021|1956|5243|5347|9212</td><td>AKT1|APEX1|APP|CCNB1|CDK1|CDK2|CDK6|EGFR|ABCB1|PLK1|AURKB</td><td>-4.7232</td><td>10</td><td>0</td><td>0</td><td>-13.3451</td><td>41.71852</td></tr>
<tr><td>-6.54397</td><td>GO:0002064</td><td>epithelial cell development</td><td>19_GO:0032502 developmental process</td><td>-6.54397</td><td>12.36104</td><td>9.189575</td><td>225</td><td>8</td><td>9.876543</td><td>3.314965</td><td>207|367|1021|2099|2932|4233|6714|7298</td><td>AKT1|AR|CDK6|ESR1|GSK3B|MET|SRC|TYMS</td><td>-4.71427</td><td>43</td><td>0</td><td>0</td><td>-6.76848</td><td>45.34622</td></tr>
<tr><td>-6.53878</td><td>GO:0098754</td><td>detoxification</td><td>19_GO:0098754 detoxification</td><td>-6.53878</td><td>16.0104</td><td>9.966283</td><td>152</td><td>7</td><td>8.641975</td><td>3.122032</td><td>4353|5243|5292|5743|9429|9536|57016</td><td>MPO|ABCB1|PIM1|PTGS2|ABCG2|PTGES|AKR1B10</td><td>-4.71094</td><td>15</td><td>0</td><td>0</td><td>-12.2065</td><td>35.47493</td></tr>
<tr><td>-6.53318</td><td>GO:1901992</td><td>positive regulation of mitotic cell cycle phase transition</td><td>19_GO:0048518 positive regulation of biological process</td><td>-6.53318</td><td>22.42931</td><td>11.117662</td><td>93</td><td>6</td><td>7.407407</td><td>2.909904</td><td>207|328|351|891|983|1956</td><td>AKT1|APEX1|APP|CCNB1|CDK1|EGFR</td><td>-4.70718</td><td>10</td><td>0</td><td>0</td><td>-13.3451</td><td>41.71852</td></tr>
<tr><td>-6.527</td><td>GO:0051900</td><td>regulation of mitochondrial</td><td>19_GO:0050789 regulation of</td><td>-6.527</td><td>69.53086</td><td>16.</td><td>20</td><td>4</td><td>4.938272</td><td>2.407398</td><td>142|239|3791|6714</td><td>PARP1|ALOX12|KDR|SRC</td><td>-4.7029</td><td>40</td><td>0</td><td>0</td><td>-7.1</td><td>115.8848</td></tr>
</table>

06		depolarization	biological process	06		46643							1				2867	
-6.5229	GO:0006692	prostanoid metabolic process	19_GO:0008152 metabolic process	-6.5229	35.47493	12.97256	49	5	6.17284	2.674018	1645|1646|5743|8644|9536	AKR1C1|AKR1C2|PTGS2|AKR1C3|PTGES	-4.70242	15	0	0	-12.2065	35.47493
-6.5229	GO:0006693	prostaglandin metabolic process	19_GO:0008152 metabolic process	-6.5229	35.47493	12.97256	49	5	6.17284	2.674018	1645|1646|5743|8644|9536	AKR1C1|AKR1C2|PTGS2|AKR1C3|PTGES	-4.70242	15	0	0	-12.2065	35.47493
-6.50552	GO:0051591	response to cAMP	19_GO:0050896 response to stimulus	-6.50552	22.1907	11.052981	94	6	7.407407	2.909904	196|328|351|1080|5294|7299	AHR|APEX1|APP|CFTR|PIK3CG|TYR	-4.68867	23	0	0	-9.78876	32.19021
-6.50552	GO:0010389	regulation of G2/M transition of mitotic cell cycle	19_GO:0050789 regulation of biological process	-6.50552	22.1907	11.052981	94	6	7.407407	2.909904	351|891|983|1017|5347|9212	APP|CCNB1|CDK1|CDK2|PLK1|AURKB	-4.68867	34	0	0	-7.56608	35.65685
-6.49006	GO:0045787	positive regulation of cell cycle	19_GO:0048518 positive regulation of biological process	-6.49006	9.901547	8.5468	316	9	11.11111	3.491885	207|328|351|891|983|1956|3643|6714|9212	AKT1|APEX1|APP|CCNB1|CDK1|EGFR|INSR|SRC|AURKB	-4.67502	10	0	0	-13.3451	41.71852
-6.47634	GO:0008285	negative regulation of cell population proliferation	19_GO:0048519 negative regulation of biological process	-6.47634	5.801677	7.29957	779	13	16.04938	4.078483	134|140|240|351|367|383|1021|1545|3558|5467|5743|7498|9536	ADORA1|ADORA3|ALOX5|APP|AR|ARG1|CDK6|CYP1B1|IL2|PPARD|PTGS2|XDH|PTGES	-4.6631	46	1	1	-6.47634	10.53498

-6.46709	GO:1904951	positive regulation of establishment of protein localization	19_GO:0048518 positive regulation of biological process	-6.46709	9.839273	8.514129	318	9	11.11111	3.491885	43|983|1080|1956|2932|5295|5467|5743|6714	ACHE|CDK1|CFTR|EGFR|GSK3B|PIK3R1|PPARD|PTGS2|SRC	-4.65563	16	0	0	-11.5558	31.96821
-6.4482	GO:1902532	negative regulation of intracellular signal transduction	19_GO:0048519 negative regulation of biological process	-6.4482	7.201879	7.7749046	531	11	13.58025	3.80643	207|2099|2932|3156|3480|4233|4318|5743|5770|6714|7498	AKT1|ESR1|GSK3B|HMGCR|IGF1R|MET|MMP9|PTGS2|PTPN1|SRC|XDH	-4.63853	17	0	0	-11.2655	11.58848
-6.43624	GO:0038083	peptidyl-tyrosine autophosphorylation	19_GO:0008152 metabolic process	-6.43624	66.21987	16.05815	21	4	4.938272	2.407398	1956|3480|3791|6714	EGFR|IGF1R|KDR|SRC	-4.63032	4	0	0	-17.1911	66.21987
-6.43624	GO:1902644	tertiary alcohol metabolic process	19_GO:0008152 metabolic process	-6.43624	66.21987	16.05815	21	4	4.938272	2.407398	1645|1646|8644|57016	AKR1C1|AKR1C2|AKR1C3|AKR1B10	-4.63032	12	0	0	-13.14	260.7407
-6.43428	GO:0006636	unsaturated fatty acid biosynthetic process	19_GO:0008152 metabolic process	-6.43428	34.08376	12.70099	51	5	6.17284	2.674018	239|246|5743|8644|9536	ALOX12|ALOX15|PTGS2|AKR1C3|PTGES	-4.63032	11	0	0	-13.1836	260.7407
-6.43293	GO:0016042	lipid catabolic process	19_GO:0008152 metabolic process	-6.43293	9.747317	8.465661	321	9	11.11111	3.491885	134|207|238|1545|1588|5294|5467|8644|57016	ADORA1|AKT1|ALK|CYP1B1|CYP19A1|PIK3CG|PPARD|AKR1C3|AKR1B10	-4.63032	47	1	1	-6.43293	55.62469

-6.42162	GO:0036293	response to decreased oxygen levels	19_GO:0050896 response to stimulus	-6.42162	9.717046	8.449647	322	9	11.11111	3.491885	134|207|768|891|4313|4843|5467|5743|6714	ADORA1|AKT1|CA9|CCNB1|MMP2|NOS2|PPARD|PTGS2|SRC	-4.6225	19	0	0	-10.8808	39.73192
-6.42162	GO:0009895	negative regulation of catabolic process	19_GO:0048519 negative regulation of biological process	-6.42162	9.717046	8.449647	322	9	11.11111	3.491885	134|207|238|1457|1956|3156|4233|4843|5294	ADORA1|AKT1|ALK|CSNK2A1|EGFR|HMGCR|MET|NOS2|PIK3CG	-4.6225	48	1	1	-6.42162	11.4927
-6.39796	GO:0034308	primary alcohol metabolic process	19_GO:0008152 metabolic process	-6.39796	21.28496	10.80409	98	6	7.407407	2.909904	1545|1645|1646|7276|8644|57016	CYP1B1|AKR1C1|AKR1C2|TTR|AKR1C3|AKR1B10	-4.60057	12	0	0	-13.14	260.7407
-6.38976	GO:0048608	reproductive structure development	19_GO:0022414 reproductive process	-6.38976	8.1609	7.998829	426	10	12.34568	3.65512	207|367|558|1588|2099|5467|5743|5747|6714|8644	AKT1|AR|AXL|CYP19A1|ESR1|PPARD|PTGS2|PTK2|SRC|AKR1C3	-4.5941	19	0	0	-10.8808	39.73192
-6.38659	GO:0090068	positive regulation of cell cycle process	19_GO:0048518 positive regulation of biological process	-6.38659	11.78489	8.936003	236	8	9.876543	3.314965	207|328|351|891|983|1956|3643|9212	AKT1|APEX1|APP|CCNB1|CDK1|EGFR|INSR|AURKB	-4.59265	10	0	0	-13.3451	41.71852
-6.38408	GO:0043065	positive regulation of apoptotic process	19_GO:0048518 positive regulation of biological process	-6.38408	7.094986	7.673751	539	11	13.58025	3.80643	207|239|1509|1545|1612|3156|4318|5743|6714|7153|8644	AKT1|ALOX12|CTSD|CYP1B1|DAPK1|HMGCR|MMP9|PTGS2|SRC|TOP2A|AKR1C3	-4.59185	8	0	0	-14.0025	18.03867

-6.36196	GO:0061458	reproductive system development	19_GO:0032502 developmental process	-6.36196	8.10383	7.963404	429	10	12.34568	3.65512	207|367|558|1588|2099|5467|5743|5747|6714|8644	AKT1|AR|AXL|CYP19A1|ESR1|PPARD|PTGS2|PTK2|SRC|AKR1C3	-4.57144	19	0	0	-10.8808	39.73192
-6.35004	GO:0016137	glycoside metabolic process	19_GO:0008152 metabolic process	-6.35004	63.20988	15.67777	22	4	4.938272	2.407398	1645|1646|8644|57016	AKR1C1|AKR1C2|AKR1C3|AKR1B10	-4.56122	12	0	0	-13.14	260.7407
-6.34133	GO:1990962	xenobiotic transport across blood-brain barrier	19_GO:0051179 localization	-6.34133	173.8272	22.73934	6	3	3.703704	2.098362	4363|5243|9429	ABCC1|ABCB1|ABCG2	-4.5542	49	1	1	-6.34133	173.8272
-6.3332	GO:0006633	fatty acid biosynthetic process	19_GO:0008152 metabolic process	-6.3332	14.92994	9.579605	163	7	8.641975	3.122032	239|240|246|5743|8644|9536|10062	ALOX12|ALOX5|ALOX15|PTGS2|AKR1C3|PTGES|NR1H3	-4.54776	11	0	0	-13.1836	260.7407
-6.31767	GO:0031334	positive regulation of protein-containing complex assembly	19_GO:0048518 positive regulation of biological process	-6.31767	11.54039	8.826239	241	8	9.876543	3.314965	246|2099|2932|4137|4233|4314|6714|6850	ALOX15|ESR1|GSK3B|MAPT|MET|MMP3|SRC|SYK	-4.53403	50	1	1	-6.31767	11.54039
-6.31611	GO:0042391	regulation of membrane potential	19_GO:0065007 biological regulation	-6.31611	8.010468	7.905122	434	10	12.34568	3.65512	134|142|207|239|351|1080|2932|3791|4137|6714	ADORA1|PARP1|AKT1|ALOX12|APP|CFTR|GSK3B|KDR|MAPT|SRC	-4.53403	51	1	1	-6.31611	22.67311

-6.30807	GO:0030520	intracellular estrogen receptor signaling pathway	19_GO:0023052 signaling	-6.30807	32.19021	12.32185	54	5	6.17284	2.674018	142|367|2099|2100|6714	PARP1|AR|ESR1|ESR2|SRC	-4.52931	2	0	0	-20.8734	36.9845
-6.30807	GO:0071320	cellular response to cAMP	19_GO:0050896 response to stimulus	-6.30807	32.19021	12.32185	54	5	6.17284	2.674018	196|328|351|1080|5294	AHR|APEX1|APP|CFTR|PIK3CG	-4.52931	23	0	0	-9.78876	32.19021
-6.29497	GO:1902749	regulation of cell cycle G2/M phase transition	19_GO:0050789 regulation of biological process	-6.29497	20.45025	10.56957	102	6	7.407407	2.909904	351|891|983|1017|5347|9212	APP|CCNB1|CDK1|CDK2|PLK1|AURKB	-4.51787	34	0	0	-7.56608	35.65685
-6.26802	GO:0051882	mitochondrial depolarization	19_GO:0065007 biological regulation	-6.26802	60.46162	15.32223	23	4	4.938272	2.407398	142|239|3791|6714	PARP1|ALOX12|KDR|SRC	-4.49256	40	0	0	-7.12867	115.8848
-6.26187	GO:0048732	gland development	19_GO:0032502 developmental process	-6.26187	7.901235	7.836404	440	10	12.34568	3.65512	207|367|383|1956|2099|4233|6714|7298|7299|7498	AKT1|AR|ARG1|EGFR|ESR1|MET|SRC|TYMS|TYR|XDH	-4.4897	29	0	0	-8.87872	35.96424
-6.26187	GO:0051090	regulation of DNA-binding transcription factor activity	19_GO:0050789 regulation of biological process	-6.26187	7.901235	7.836404	440	10	12.34568	3.65512	140|207|238|351|367|1545|2099|2100|5292|6850	ADORA3|AKT1|ALK|APP|AR|CYP1B1|ESR1|ESR2|PIM1|SYK	-4.4897	52	1	1	-6.26187	13.03704

-6.2565	GO:0070371	ERK1 and ERK2 cascade	19_GO:0023052 signaling	-6.2565	9.284537	8.217534	337	9	11.11111	3.491885	246|351|983|1956|3156|3791|5770|6714|6850	ALOX15|APP|CDK1|EGFR|HMGCR|KDR|PTPN1|SRC|SYK	-4.48595	4	0	0	-17.1911	66.21987
-6.25291	GO:0045936	negative regulation of phosphate metabolic process	19_GO:0048519 negative regulation of biological process	-6.25291	7.883318	7.825078	441	10	12.34568	3.65512	142|207|891|2932|3156|3558|4137|5347|5770|7498	PARP1|AKT1|CCNB1|GSK3B|HMGCR|IL2|MAPT|PLK1|PTPN1|XDH	-4.48399	17	0	0	-11.2655	11.58848
-6.24398	GO:0010563	negative regulation of phosphorus metabolic process	19_GO:0048519 negative regulation of biological process	-6.24398	7.865482	7.813787	442	10	12.34568	3.65512	142|207|891|2932|3156|3558|4137|5347|5770|7498	PARP1|AKT1|CCNB1|GSK3B|HMGCR|IL2|MAPT|PLK1|PTPN1|XDH	-4.47667	17	0	0	-11.2655	11.58848
-6.22802	GO:0046456	icosanoid biosynthetic process	19_GO:0008152 metabolic process	-6.22802	31.04056	12.08588	56	5	6.17284	2.674018	240|5743|6850|8644|9536	ALOX5|PTGS2|SYK|AKR1C3|PTGES	-4.46232	15	0	0	-12.2065	35.47493
-6.18978	GO:0032928	regulation of superoxide anion generation	19_GO:0050789 regulation of biological process	-6.18978	57.94239	14.98892	24	4	4.938272	2.407398	207|1956|4137|6850	AKT1|EGFR|MAPT|SYK	-4.42569	7	0	0	-14.1215	57.94239
-6.17138	GO:0046883	regulation of hormone secretion	19_GO:0051179 localization	-6.17138	11.03665	8.59577	252	8	9.876543	3.314965	134|240|1080|1588|1956|3156|4843|5467	ADORA1|ALOX5|CFTR|CYP19A1|EGFR|HMGCR|NOS2|PPARD	-4.40889	28	0	0	-8.92043	22.1907
-	GO:007	response to oxygen	19_GO:0050896	-	9.016	8.	34	9	11.1	3.491	134|207|768|891|	ADORA1|AKT1|CA9|CCNB1|	-	19	0	0	-	39.73

<table>
<tr><th></th><th></th><th></th><th></th><th></th><th></th><th></th><th></th><th></th><th></th><th></th><th></th><th></th><th></th><th></th><th></th><th></th><th></th><th></th></tr>
<tr><td>6.15088</td><td>0482</td><td>levels</td><td>response to stimulus</td><td>6.15088</td><td>971</td><td>070704</td><td>7</td><td></td><td>1111</td><td>885</td><td>4313|4843|5467|5743|6714</td><td>MMP2|NOS2|PPARD|PTGS2|SRC</td><td>4.38998</td><td></td><td></td><td></td><td>10.8808</td><td>192</td></tr>
<tr><td>-6.14202</td><td>GO:0048639</td><td>positive regulation of developmental growth</td><td>19_GO:0040007 growth</td><td>-6.14202</td><td>13.98609</td><td>9.228753</td><td>174</td><td>7</td><td>8.641975</td><td>3.122032</td><td>207|891|983|3643|4137|5292|5467</td><td>AKT1|CCNB1|CDK1|INSR|MAPT|PIM1|PPARD</td><td>-4.3843</td><td>35</td><td>0</td><td>0</td><td>-7.56218</td><td>38.62826</td></tr>
<tr><td>-6.14202</td><td>GO:0001659</td><td>temperature homeostasis</td><td>19_GO:0032501 multicellular organismal process</td><td>-6.14202</td><td>13.98609</td><td>9.228753</td><td>174</td><td>7</td><td>8.641975</td><td>3.122032</td><td>43|134|3480|5743|6850|9536|10062</td><td>ACHE|ADORA1|IGF1R|PTGS2|SYK|PTGES|NR1H3</td><td>-4.3843</td><td>53</td><td>1</td><td>1</td><td>-6.14202</td><td>13.98609</td></tr>
<tr><td>-6.1247</td><td>GO:0007173</td><td>epidermal growth factor receptor signaling pathway</td><td>19_GO:0023052 signaling</td><td>-6.1247</td><td>19.13694</td><td>10.189774</td><td>109</td><td>6</td><td>7.407407</td><td>2.909904</td><td>134|207|1956|4318|5747|6714</td><td>ADORA1|AKT1|EGFR|MMP9|PTK2|SRC</td><td>-4.36855</td><td>21</td><td>0</td><td>0</td><td>-9.98291</td><td>27.26701</td></tr>
<tr><td>-6.11501</td><td>GO:0050995</td><td>negative regulation of lipid catabolic process</td><td>19_GO:0048519 negative regulation of biological process</td><td>-6.11501</td><td>55.62469</td><td>14.67556</td><td>25</td><td>4</td><td>4.938272</td><td>2.407398</td><td>134|207|238|5294</td><td>ADORA1|AKT1|ALK|PIK3CG</td><td>-4.36045</td><td>47</td><td>0</td><td>0</td><td>-6.43293</td><td>55.62469</td></tr>
<tr><td>-6.11346</td><td>GO:0048010</td><td>vascular endothelial growth factor receptor signaling pathway</td><td>19_GO:0023052 signaling</td><td>-6.11346</td><td>29.46223</td><td>11.754223</td><td>59</td><td>5</td><td>6.17284</td><td>2.674018</td><td>558|3791|5747|5770|6714</td><td>AXL|KDR|PTK2|PTPN1|SRC</td><td>-4.36045</td><td>41</td><td>0</td><td>0</td><td>-7.04885</td><td>38.62826</td></tr>
<tr><td>-6.108</td><td>GO:0050728</td><td>negative regulation of inflammatory response</td><td>19_GO:0048519 negative</td><td>-6.108</td><td>13.82716</td><td>9.1</td><td>176</td><td>7</td><td>8.641975</td><td>3.122032</td><td>134|240|3558|5467|6714|6850|</td><td>ADORA1|ALOX5|IL2|PPARD|SRC|SYK|NR1H3</td><td>-4.3572</td><td>25</td><td>0</td><td>0</td><td>-9.4</td><td>22.67311</td></tr>
</table>

67			regulation of biological process	67		68373					10062		7				8567	
−6.10718	GO:1903522	regulation of blood circulation	19_GO:0032501 multicellular organismal process	−6.10718	10.82192	8.495677	257	8	9.876543	3.314965	134|140|207|1956|3156|3558|5294|5743	ADORA1|ADORA3|AKT1|EGFR|HMGCR|IL2|PIK3CG|PTGS2	−4.35727	19	0	0	−10.8808	39.73192
−6.09545	GO:0007346	regulation of mitotic cell cycle	19_GO:0050789 regulation of biological process	−6.09545	7.574168	7.627105	459	10	12.34568	3.65512	207|328|351|891|983|1017|1956|3643|5347|9212	AKT1|APEX1|APP|CCNB1|CDK1|CDK2|EGFR|INSR|PLK1|AURKB	−4.34709	10	0	0	−13.3451	41.71852
−6.07572	GO:0035265	organ growth	19_GO:0040007 growth	−6.07572	13.6718	9.108969	178	7	8.641975	3.122032	207|367|891|983|2099|4322|5292	AKT1|AR|CCNB1|CDK1|ESR1|MMP13|PIM1	−4.32891	35	0	0	−7.56218	38.62826
−6.06931	GO:0042593	glucose homeostasis	19_GO:0065007 biological regulation	−6.06931	10.69706	8.436937	260	8	9.876543	3.314965	207|240|1080|3156|3480|3643|5295|5467	AKT1|ALOX5|CFTR|HMGCR|IGF1R|INSR|PIK3R1|PPARD	−4.32504	54	1	1	−6.06931	37.58425
−6.06878	GO:0015711	organic anion transport	19_GO:0051179 localization	−6.06878	8.813772	7.957447	355	9	11.11111	3.491885	134|383|1080|1645|4363|4843|6850|9429|9536	ADORA1|ARG1|CFTR|AKR1C1|ABCC1|NOS2|SYK|ABCG2|PTGES	−4.32504	33	0	0	−8.21676	32.59259
−6.058	GO:2001233	regulation of apoptotic signaling pathway	19_GO:0023052 signaling	−6.058	8.789014	7.9	356	9	11.11111	3.491885	142|207|367|1457|2932|4318|5743|	PARP1|AKT1|AR|CSNK2A1|GSK3B|MMP9|PTGS2|PTPN1|	−4.3164	21	0	0	−9.9	27.26701

67				67		43541					5770|6714	SRC	5				8291	
-6.05679	GO:0033500	carbohydrate homeostasis	19_GO:0065007 biological regulation	-6.05679	10.65607	8.417569	261	8	9.876543	3.314965	207|240|1080|3156|3480|3643|5295|5467	AKT1|ALOX5|CFTR|HMGCR|IGF1R|INSR|PIK3R1|PPARD	-4.31609	54	0	0	-6.06931	37.58425
-6.05527	GO:0001676	long-chain fatty acid metabolic process	19_GO:0008152 metabolic process	-6.05527	18.62434	10.03763	112	6	7.407407	2.909904	239|240|246|1545|5743|8644	ALOX12|ALOX5|ALOX15|CYP1B1|PTGS2|AKR1C3	-4.31609	11	0	0	-13.1836	260.7407
-6.04048	GO:0071384	cellular response to corticosteroid stimulus	19_GO:0050896 response to stimulus	-6.04048	28.49626	11.54658	61	5	6.17284	2.674018	383|1956|2322|2908|8644	ARG1|EGFR|FLT3|NR3C1|AKR1C3	-4.30282	29	0	0	-8.87872	35.96424
-6.02768	GO:0001819	positive regulation of cytokine production	19_GO:0048518 positive regulation of biological process	-6.02768	7.444418	7.542537	467	10	12.34568	3.65512	351|1545|3558|4321|4843|5294|5295|5743|6714|6850	APP|CYP1B1|IL2|MMP12|NOS2|PIK3CG|PIK3R1|PTGS2|SRC|SYK	-4.29152	5	0	0	-15.0818	15.19312
-6.01955	GO:0050730	regulation of peptidyl-tyrosine phosphorylation	19_GO:0050789 regulation of biological process	-6.01955	10.53498	8.360092	264	8	9.876543	3.314965	134|351|1956|2322|3558|5770|6714|6850	ADORA1|APP|EGFR|FLT3|IL2|PTPN1|SRC|SYK	-4.28489	4	0	0	-17.1911	66.21987
-5.987	GO:1901989	positive regulation of cell cycle phase	19_GO:0048518 positive	-5.987	18.13849	9.8	115	6	7.407407	2.909904	207|328|351|891|983|1956	AKT1|APEX1|APP|CCNB1|CDK1|EGFR	-4.2546	10	0	0	-13.	41.71852

79		transition	regulation of biological process	79		91313							3				3451	
-5.98599	GO:0019221	cytokine-mediated signaling pathway	19_GO:0023052 signaling	-5.98599	7.365558	7.490692	472	10	12.34568	3.65512	207|383|558|2322|3577|4321|5770|6714|6850|10062	AKT1|ARG1|AXL|FLT3|CXCR1|MMP12|PTPN1|SRC|SYK|NR1H3	-4.25432	55	1	1	-5.98599	20.05698
-5.97885	GO:0051098	regulation of binding	19_GO:0065007 biological regulation	-5.97885	8.595849	7.834227	364	9	11.11111	3.491885	142|207|351|2932|4233|4318|5347|6714|9212	PARP1|AKT1|APP|GSK3B|MET|MMP9|PLK1|SRC|AURKB	-4.24866	21	0	0	-9.98291	27.26701
-5.97061	GO:0050708	regulation of protein secretion	19_GO:0051179 localization	-5.97061	10.37774	8.284878	268	8	9.876543	3.314965	43|240|1080|1956|3156|4843|5467|10062	ACHE|ALOX5|CFTR|EGFR|HMGCR|NOS2|PPARD|NR1H3	-4.2419	28	0	0	-8.92043	22.1907
-5.96572	GO:0030518	intracellular steroid hormone receptor signaling pathway	19_GO:0023052 signaling	-5.96572	17.98212	9.843765	116	6	7.407407	2.909904	142|367|2099|2100|2908|6714	PARP1|AR|ESR1|ESR2|NR3C1|SRC	-4.23995	2	0	0	-20.8734	36.9845
-5.96572	GO:1990748	cellular detoxification	19_GO:0098754 detoxification	-5.96572	17.98212	9.843765	116	6	7.407407	2.909904	4353|5292|5743|9429|9536|57016	MPO|PIM1|PTGS2|ABCG2|PTGES|AKR1B10	-4.23995	15	0	0	-12.2065	35.47493
-5.960	GO:1903047	mitotic cell cycle process	19_GO:0009987 cellular process	-5.960	5.746352	6.9	726	12	14.81481	3.947186	207|328|351|891|983|1017|1021|	AKT1|APEX1|APP|CCNB1|CDK1|CDK2|CDK6|EGFR|	-4.2362	10	0	0	-13.	41.71852

52				52		58955					1956|3643|5243|5347|9212	INSR|ABCB1|PLK1|AURKB	1				3451	
-5.94385	GO:0018107	peptidyl-threonine phosphorylation	19_GO:0008152 metabolic process	-5.94385	17.82843	9.796806	117	6	7.407407	2.909904	207|351|983|1457|2932|5347	AKT1|APP|CDK1|CSNK2A1|GSK3B|PLK1	-4.221	43	0	0	-6.76848	45.34622
-5.93223	GO:0071478	cellular response to radiation	19_GO:0050896 response to stimulus	-5.93223	13.0138	8.85303	187	7	8.641975	3.122032	142|4313|4314|4318|5295|5743|9212	PARP1|MMP2|MMP3|MMP9|PIK3R1|PTGS2|AURKB	-4.21084	6	0	0	-14.9128	124.1623
-5.90187	GO:0022617	extracellular matrix disassembly	19_GO:0009987 cellular process	-5.90187	26.74264	11.15978	65	5	6.17284	2.674018	4313|4314|4318|4321|4322	MMP2|MMP3|MMP9|MMP12|MMP13	-4.18193	6	0	0	-14.9128	124.1623
-5.89869	GO:0046942	carboxylic acid transport	19_GO:0051179 localization	-5.89869	10.15049	8.174983	274	8	9.876543	3.314965	134|383|1645|4363|4843|6850|9429|9536	ADORA1|ARG1|AKR1C1|ABCC1|NOS2|SYK|ABCG2|PTGES	-4.18019	33	0	0	-8.21676	32.59259
-5.89598	GO:0051712	positive regulation of killing of cells of other organism	19_GO:0044419 biological process involved in interspecies interaction between organisms	-5.89598	130.3704	19.65556	8	3	3.703704	2.098362	383|4843|6850	ARG1|NOS2|SYK	-4.17892	56	1	1	-5.89598	130.3704
-5.8799	GO:0016049	cell growth	19_GO:0040007 growth	-5.8799	7.16813	7.35	485	10	12.34568	3.65512	207|351|1457|1956|2100|2147|2932|3558|4137|	AKT1|APP|CSNK2A1|EGFR|ESR2|F2|GSK3B|IL2|MAPT|PPARD	-4.16428	24	0	0	-9.6549	14.76524

						9374					5467						5	
-5.85831	GO:0045931	positive regulation of mitotic cell cycle	19_GO:0048518 positive regulation of biological process	-5.85831	17.23906	9.614629	121	6	7.407407	2.909904	207|328|351|891|983|1956	AKT1|APEX1|APP|CCNB1|CDK1|EGFR	-4.14413	10	0	0	-13.3451	41.71852
-5.8374	GO:0038127	ERBB signaling pathway	19_GO:0023052 signaling	-5.8374	17.09775	9.570442	122	6	7.407407	2.909904	134|207|1956|4318|5747|6714	ADORA1|AKT1|EGFR|MMP9|PTK2|SRC	-4.12464	21	0	0	-9.98291	27.26701
-5.83592	GO:0032890	regulation of organic acid transport	19_GO:0051179 localization	-5.83592	25.94435	10.97992	67	5	6.17284	2.674018	134|207|383|6850|9536	ADORA1|AKT1|ARG1|SYK|PTGES	-4.12458	33	0	0	-8.21676	32.59259
-5.82569	GO:0009617	response to bacterium	19_GO:0044419 biological process involved in interspecies interaction between organisms	-5.82569	5.577342	6.814816	748	12	14.81481	3.947186	207|383|558|2147|4353|4843|5467|5743|6714|6850|9536|10062	AKT1|ARG1|AXL|F2|MPO|NOS2|PPARD|PTGS2|SRC|SYK|PTGES|NR1H3	-4.11576	19	0	0	-10.8808	39.73192
-5.81693	GO:0043542	endothelial cell migration	19_GO:0040011 locomotion	-5.81693	9.897632	8.050999	281	8	9.876543	3.314965	207|239|1545|3791|4233|5294|5743|5747	AKT1|ALOX12|CYP1B1|KDR|MET|PIK3CG|PTGS2|PTK2	-4.10842	20	0	0	-10.2592	15.71319
-5.79612	GO:0030168	platelet activation	19_GO:0032501 multicellular organismal process	-5.79612	16.82198	9.48336	124	6	7.407407	2.909904	239|558|2147|5294|6714|6850	ALOX12|AXL|F2|PIK3CG|SRC|SYK	-4.09043	9	0	0	-13.6942	26.07407

						18											
-5.79612	GO:0097237	cellular response to toxic substance	19_GO:0050896 response to stimulus	-5.79612	16.82198	9.483618	124	6	7.407407	2.909904	4353|5292|5743|9429|9536|57016	MPO|PIM1|PTGS2|ABCG2|PTGES|AKR1B10	-4.09043	15	0	0	-12.2065	35.47493
-5.78131	GO:0043393	regulation of protein binding	19_GO:0065007 biological regulation	-5.78131	12.3532	8.588549	197	7	8.641975	3.122032	207|351|2932|4318|5347|6714|9212	AKT1|APP|GSK3B|MMP9|PLK1|SRC|AURKB	-4.07701	21	0	0	-9.98291	27.26701
-5.77204	GO:0043550	regulation of lipid kinase activity	19_GO:0050789 regulation of biological process	-5.77204	25.19234	10.80635	69	5	6.17284	2.674018	2147|2322|5295|5747|6714	F2|FLT3|PIK3R1|PTK2|SRC	-4.06913	41	0	0	-7.04885	38.62826
-5.76668	GO:0031099	regeneration	19_GO:0032502 developmental process	-5.76668	12.29081	8.563156	198	7	8.641975	3.122032	558|891|983|1956|2322|5467|7298	AXL|CCNB1|CDK1|EGFR|FLT3|PPARD|TYMS	-4.06516	57	1	1	-5.76668	22.87199
-5.75555	GO:0018210	peptidyl-threonine modification	19_GO:0008152 metabolic process	-5.75555	16.55497	9.398795	126	6	7.407407	2.909904	207|351|983|1457|2932|5347	AKT1|APP|CDK1|CSNK2A1|GSK3B|PLK1	-4.05542	43	0	0	-6.76848	45.34622
-5.73765	GO:0010952	positive regulation of peptidase activity	19_GO:0048518 positive regulation of biological process	-5.73765	12.1679	8.5129	200	7	8.641975	3.122032	239|351|1509|1612|4137|6850|7498	ALOX12|APP|CTSD|DAPK1|MAPT|SYK|XDH	-4.04139	8	0	0	-14.0025	18.03867

-5.73765	GO:0002791	regulation of peptide secretion	19_GO:0051179 localization	-5.73765	12.1679	158.5129	200	7	8.641975	3.122032	134|240|1080|1956|3156|4843|5467	ADORA1|ALOX5|CFTR|EGFR|HMGCR|NOS2|PPARD	-4.04139	28	0	0	-8.92043	22.1907
-5.73739	GO:0097193	intrinsic apoptotic signaling pathway	19_GO:0023052 signaling	-5.73739	9.657064	157.93127	288	8	9.876543	3.314965	142|207|1545|4318|5295|5743|5770|6714	PARP1|AKT1|CYP1B1|MMP9|PIK3R1|PTGS2|PTPN1|SRC	-4.04139	21	0	0	-9.98291	27.26701
-5.73236	GO:1901987	regulation of cell cycle phase transition	19_GO:0050789 regulation of biological process	-5.73236	8.022792	77.500919	390	9	11.11111	3.491885	207|328|351|891|983|1017|1956|5347|9212	AKT1|APEX1|APP|CCNB1|CDK1|CDK2|EGFR|PLK1|AURKB	-4.03773	10	0	0	-13.3451	41.71852
-5.72498	GO:0070633	transepithelial transport	19_GO:0051179 localization	-5.72498	44.85862	13.12257	31	4	4.938272	2.407398	1080|4363|5243|9429	CFTR|ABCC1|ABCB1|ABCG2	-4.03171	49	0	0	-6.34133	173.8272
-5.72079	GO:0051121	hepoxilin metabolic process	19_GO:0008152 metabolic process	-5.72079	115.8848	18.51387	9	3	3.703704	2.098362	239|240|246	ALOX12|ALOX5|ALOX15	-4.03158	11	0	0	-13.1836	260.7407
-5.72079	GO:0051122	hepoxilin biosynthetic process	19_GO:0008152 metabolic process	-5.72079	115.8848	18.513	9	3	3.703704	2.098362	239|240|246	ALOX12|ALOX5|ALOX15	-4.03158	11	0	0	-13.1836	260.7407

						87											
-5.72079	GO:0051901	positive regulation of mitochondrial depolarization	19_GO:0048518 positive regulation of biological process	-5.72079	115.8848	18.51387	9	3	3.703704	2.098362	142|239|3791	PARP1|ALOX12|KDR	-4.03158	40	0	0	-7.12867	115.8848
-5.68962	GO:0001568	blood vessel development	19_GO:0032502 developmental process	-5.68962	5.410962	6.670106	771	12	14.81481	3.947186	196|207|240|1545|3791|4313|4638|5294|5743|5747|6850|7498	AHR|AKT1|ALOX5|CYP1B1|KDR|MMP2|MYLK|PIK3CG|PTGS2|PTK2|SYK|XDH	-4.00177	20	0	0	-10.2592	15.71319
-5.68365	GO:0006897	endocytosis	19_GO:0051179 localization	-5.68365	6.012889	6.867856	636	11	13.58025	3.80643	43|246|351|558|3577|3643|5294|5770|6714|6850|10062	ACHE|ALOX15|APP|AXL|CXCR1|INSR|PIK3CG|PTPN1|SRC|SYK|NR1H3	-3.99714	44	0	0	-6.70994	13.24397
-5.66793	GO:0045737	positive regulation of cyclin-dependent protein serine/threonine kinase activity	19_GO:0048518 positive regulation of biological process	-5.66793	43.45679	12.90663	32	4	4.938272	2.407398	207|891|1956|6714	AKT1|CCNB1|EGFR|SRC	-3.98276	58	1	1	-5.66793	43.45679
-5.63786	GO:0043280	positive regulation of cysteine-type endopeptidase activity involved in apoptotic process	19_GO:0048518 positive regulation of biological process	-5.63786	15.80247	9.15557	132	6	7.407407	2.909904	239|1509|1612|4137|6850|7498	ALOX12|CTSD|DAPK1|MAPT|SYK|XDH	-3.95401	8	0	0	-14.0025	18.03867
-5.62738	GO:0046879	hormone secretion	19_GO:0051179 localization	-5.62738	9.333002	7.767188	298	8	9.876543	3.314965	134|240|1080|1588|1956|3156|4843|5467	ADORA1|ALOX5|CFTR|CYP19A1|EGFR|HMGCR|NOS2|PPARD	-3.94487	28	0	0	-8.92043	22.1907

7

-5.62461	GO:0071222	cellular response to lipopolysaccharide	19_GO:0044419 biological process involved in interspecies interaction between organisms	-5.62461	11.69991	8.3188891	208	7	8.641975	3.122032	207|383|558|4843|5467|6714|10062	AKT1|ARG1|AXL|NOS2|PPARD|SRC|NR1H3	-3.94343	19	0	0	-10.8808	39.73192
-5.59989	GO:0010595	positive regulation of endothelial cell migration	19_GO:0040011 locomotion	-5.59989	15.56661	9.078801	134	6	7.407407	2.909904	207|239|3791|4233|5294|5743	AKT1|ALOX12|KDR|MET|PIK3CG|PTGS2	-3.92135	20	0	0	-10.2592	15.71319
-5.59989	GO:0061041	regulation of wound healing	19_GO:0050896 response to stimulus	-5.59989	15.56661	9.078801	134	6	7.407407	2.909904	239|240|2147|3156|4638|5747	ALOX12|ALOX5|F2|HMGCR|MYLK|PTK2	-3.92135	59	1	1	-5.59989	17.82843
-5.59482	GO:0044089	positive regulation of cellular component biogenesis	19_GO:0048518 positive regulation of biological process	-5.59482	6.660044	7.010711	522	10	12.34568	3.65512	246|2099|2932|3791|4137|4233|4314|5295|6714|6850	ALOX15|ESR1|GSK3B|KDR|MAPT|MET|MMP3|PIK3R1|SRC|SYK	-3.91758	50	0	0	-6.31767	11.54039
-5.58003	GO:0010564	regulation of cell cycle process	19_GO:0050789 regulation of biological process	-5.58003	5.865334	6.750986	652	11	13.58025	3.80643	207|328|351|891|983|1017|1457|1956|3643|5347|9212	AKT1|APEX1|APP|CCNB1|CDK1|CDK2|CSNK2A1|EGFR|INSR|PLK1|AURKB	-3.90411	10	0	0	-13.3451	41.71852
-5.563	GO:0006809	nitric oxide biosynthetic process	19_GO:0008152 metabolic process	-5.563	22.87199	10.25477	76	5	6.17284	2.674018	207|1545|3643|4843|5743	AKT1|CYP1B1|INSR|NOS2|PTGS2	-3.88968	19	0	0	-10.8808	39.73192
-	GO:003	animal organ	19_GO:0032502	-	22.87	1	76	5	6.17	2.674	558|983|1956|	AXL|CDK1|EGFR|FLT3|TYMS	-	57	0	0	-	22.87

5.563	1100	regeneration	developmental process	5.563	199	0.254777			284	018	2322|7298		3.88968				5.76668	199
-5.55459	GO:0002252	immune effector process	19_GO:0002376 immune system process	-5.55459	5.829569	6.722375	656	11	13.58025	3.80643	196|351|383|2147|3558|4353|4843|5294|5747|6714|6850	AHR|APP|ARG1|F2|IL2|MPO|NOS2|PIK3CG|PTK2|SRC|SYK	-3.88257	56	0	0	-5.89598	130.3704
-5.5429	GO:0072330	monocarboxylic acid biosynthetic process	19_GO:0008152 metabolic process	-5.5429	11.37187	8.18021	214	7	8.641975	3.122032	239|240|246|5743|8644|9536|10062	ALOX12|ALOX5|ALOX15|PTGS2|AKR1C3|PTGES|NR1H3	-3.87218	11	0	0	-13.1836	260.7407
-5.53621	GO:0060627	regulation of vesicle-mediated transport	19_GO:0051179 localization	-5.53621	6.559515	6.939765	530	10	12.34568	3.65512	246|351|558|1080|2932|3643|5770|6714|6850|10062	ALOX15|APP|AXL|CFTR|GSK3B|INSR|PTPN1|SRC|SYK|NR1H3	-3.86678	44	0	0	-6.70994	13.24397
-5.51621	GO:0009612	response to mechanical stimulus	19_GO:0050896 response to stimulus	-5.51621	11.26658	8.135205	216	7	8.641975	3.122032	207|891|1956|4353|5743|5747|6714	AKT1|CCNB1|EGFR|MPO|PTGS2|PTK2|SRC	-3.84807	58	0	0	-5.66793	43.45679
-5.511	GO:0006909	phagocytosis	19_GO:0051179 localization	-5.511	9.000759	7.595395	309	8	9.876543	3.314965	134|246|558|4233|5747|6714|6850|10062	ADORA1|ALOX15|AXL|MET|PTK2|SRC|SYK|NR1H3	-3.84525	9	0	0	-13.6942	26.07407
-5.507	GO:0006691	leukotriene metabolic process	19_GO:0008152 metabolic process	-5.507	39.73192	12.	35	4	4.938272	2.407398	239|240|4363|6850	ALOX12|ALOX5|ABCC1|SYK	-3.8452	11	0	0	-13.	260.7407

6				6		31451							5				1836	
-5.5076	GO:0045907	positive regulation of vasoconstriction	19_GO:0048518 positive regulation of biological process	-5.5076	39.73192	12.314511	35	4	4.938272	2.407398	207|1956|3156|5743	AKT1|EGFR|HMGCR|PTGS2	-3.84525	19	0	0	-10.8808	39.73192
-5.5076	GO:0034405	response to fluid shear stress	19_GO:0050896 response to stimulus	-5.5076	39.73192	12.314511	35	4	4.938272	2.407398	207|760|5743|6714	AKT1|CA2|PTGS2|SRC	-3.84525	36	0	0	-7.34456	54.89279
-5.50702	GO:0043154	negative regulation of cysteine-type endopeptidase activity involved in apoptotic process	19_GO:0048519 negative regulation of biological process	-5.50702	22.28553	10.110063	78	5	6.17284	2.674018	207|1457|4318|5743|6714	AKT1|CSNK2A1|MMP9|PTGS2|SRC	-3.84525	21	0	0	-9.98291	27.26701
-5.50064	GO:0051047	positive regulation of secretion	19_GO:0048518 positive regulation of biological process	-5.50064	8.971724	7.580203	310	8	9.876543	3.314965	43|134|1080|1588|1956|5467|6850|9536	ACHE|ADORA1|CFTR|CYP19A1|EGFR|PPARD|SYK|PTGES	-3.84014	28	0	0	-8.92043	22.1907
-5.49518	GO:0001558	regulation of cell growth	19_GO:0040007 growth	-5.49518	7.503331	7.186004	417	9	11.11111	3.491885	207|1457|1956|2100|2147|2932|3558|4137|5467	AKT1|CSNK2A1|EGFR|ESR2|F2|GSK3B|IL2|MAPT|PPARD	-3.83594	24	0	0	-9.65495	14.76524
-5.489	GO:0030879	mammary gland development	19_GO:0032502 developmental	-5.489	14.89947	8.8	140	6	7.407407	2.909904	207|367|383|2099|6714|7498	AKT1|AR|ARG1|ESR1|SRC|XDH	-3.8315	60	1	1	-5.4	45.34622

56			process	56		54999							7				8956	
-5.48005	GO:0019932	second-messenger-mediated signaling	19_GO:0023052 signaling	-5.48005	8.914213	7.5500024	312	8	9.876543	3.314965	196|1956|2932|3577|3791|4137|4843|6850	AHR|EGFR|GSK3B|CXCR1|KDR|MAPT|NOS2|SYK	-3.82457	7	0	0	-14.1215	57.94239
-5.48005	GO:0060326	cell chemotaxis	19_GO:0040011 locomotion	-5.48005	8.914213	7.5500024	312	8	9.876543	3.314965	240|3577|3791|4233|4363|5294|5747|6850	ALOX5|CXCR1|KDR|MET|ABCC1|PIK3CG|PTK2|SYK	-3.82457	42	0	0	-6.93111	31.60494
-5.47668	GO:0034764	positive regulation of transmembrane transport	19_GO:0048518 positive regulation of biological process	-5.47668	11.11224	8.068795	219	7	8.641975	3.122032	207|760|1080|2147|3643|5243|5295	AKT1|CA2|CFTR|F2|INSR|ABCB1|PIK3R1	-3.82245	27	0	0	-8.92369	24.25495
-5.47166	GO:0035296	regulation of tube diameter	19_GO:0065007 biological regulation	-5.47166	14.7938	8.819166	141	6	7.407407	2.909904	134|207|1956|3156|5467|5743	ADORA1|AKT1|EGFR|HMGCR|PPARD|PTGS2	-3.82114	19	0	0	-10.8808	39.73192
-5.47166	GO:0097746	blood vessel diameter maintenance	19_GO:0032501 multicellular organismal process	-5.47166	14.7938	8.819166	141	6	7.407407	2.909904	134|207|1956|3156|5467|5743	ADORA1|AKT1|EGFR|HMGCR|PPARD|PTGS2	-3.82114	19	0	0	-10.8808	39.73192
-5.471	GO:0032355	response to estradiol	19_GO:0050896 response to	-5.471	14.7938	8.8	141	6	7.407407	2.909904	1588|1956|2099|2100|2101|5743	CYP19A1|EGFR|ESR1|ESR2|ESRRA|PTGS2	-3.8211	61	1	1	-5.4	27.44639

66			stimulus	66		19166							4				7166	
-5.46364	GO:0071219	cellular response to molecule of bacterial origin	19_GO:0044419 biological process involved in interspecies interaction between organisms	-5.46364	11.06173	8.046945	220	7	8.641975	3.122032	207|383|558|4843|5467|6714|10062	AKT1|ARG1|AXL|NOS2|PPARD|SRC|NR1H3	-3.81435	19	0	0	-10.8808	39.73192
-5.4574	GO:0090322	regulation of superoxide metabolic process	19_GO:0050789 regulation of biological process	-5.4574	38.62826	12.133353	36	4	4.938272	2.407398	207|1956|4137|6850	AKT1|EGFR|MAPT|SYK	-3.81179	7	0	0	-14.1215	57.94239
-5.4574	GO:0008207	C21-steroid hormone metabolic process	19_GO:0008152 metabolic process	-5.4574	38.62826	12.133353	36	4	4.938272	2.407398	1586|1645|1646|8644	CYP17A1|AKR1C1|AKR1C2|AKR1C3	-3.81179	12	0	0	-13.14	260.7407
-5.4574	GO:1904031	positive regulation of cyclin-dependent protein kinase activity	19_GO:0048518 positive regulation of biological process	-5.4574	38.62826	12.133353	36	4	4.938272	2.407398	207|891|1956|6714	AKT1|CCNB1|EGFR|SRC	-3.81179	58	0	0	-5.66793	43.45679
-5.4539	GO:0050729	positive regulation of inflammatory response	19_GO:0048518 positive regulation of biological process	-5.4539	14.68962	8.783696	142	6	7.407407	2.909904	351|1956|3558|4363|5294|5743	APP|EGFR|IL2|ABCC1|PIK3CG|PTGS2	-3.81073	5	0	0	-15.0818	15.19312
-5.453	GO:0035150	regulation of tube size	19_GO:0065007 biological	-5.453	14.68962	8.7	142	6	7.407407	2.909904	134|207|1956|3156|5467|5743	ADORA1|AKT1|EGFR|HMGCR|PPARD|PTGS2	-3.8107	19	0	0	-10.	39.73192

9			regulation	9		83696							3				8808	
-5.44943	GO:0070372	regulation of ERK1 and ERK2 cascade	19_GO:0023052 signaling	-5.44943	8.829316	7.505261	315	8	9.876543	3.314965	246|351|1956|3156|3791|5770|6714|6850	ALOX15|APP|EGFR|HMGCR|KDR|PTPN1|SRC|SYK	-3.80867	4	0	0	-17.1911	66.21987
-5.44943	GO:0046394	carboxylic acid biosynthetic process	19_GO:0008152 metabolic process	-5.44943	8.829316	7.505261	315	8	9.876543	3.314965	239|240|246|5743|6850|8644|9536|10062	ALOX12|ALOX5|ALOX15|PTGS2|SYK|AKR1C3|PTGES|NR1H3	-3.80867	11	0	0	-13.1836	260.7407
-5.42939	GO:0071492	cellular response to UV-A	19_GO:0050896 response to stimulus	-5.42939	94.81481	16.71462	11	3	3.703704	2.098362	4313|4314|4318	MMP2|MMP3|MMP9	-3.79324	6	0	0	-14.9128	124.1623
-5.42939	GO:0019372	lipoxygenase pathway	19_GO:0008152 metabolic process	-5.42939	94.81481	16.71462	11	3	3.703704	2.098362	239|240|246	ALOX12|ALOX5|ALOX15	-3.79324	11	0	0	-13.1836	260.7407
-5.42939	GO:1904181	positive regulation of membrane depolarization	19_GO:0048518 positive regulation of biological process	-5.42939	94.81481	16.71462	11	3	3.703704	2.098362	142|239|3791	PARP1|ALOX12|KDR	-3.79324	40	0	0	-7.12867	115.8848
-5.429	GO:0016053	organic acid biosynthetic process	19_GO:0008152 metabolic process	-5.429	8.773611	7.4	317	8	9.876543	3.314965	239|240|246|5743|6850|8644|9536|	ALOX12|ALOX5|ALOX15|PTGS2|SYK|AKR1C3|PTGES|	-3.7932	11	0	0	-13.	260.7407

2				2		75749					10062	NR1H3	4				1836	
-5.42585	GO:0048708	astrocyte differentiation	19_GO:0032502 developmental process	-5.42585	21.46014	9.904229	81	5	6.17284	2.674018	351|1021|1956|2147|4137	APP|CDK6|EGFR|F2|MAPT	-3.79227	10	0	0	-13.3451	41.71852
-5.42585	GO:0046209	nitric oxide metabolic process	19_GO:0008152 metabolic process	-5.42585	21.46014	9.904229	81	5	6.17284	2.674018	207|1545|3643|4843|5743	AKT1|CYP1B1|INSR|NOS2|PTGS2	-3.79227	19	0	0	-10.8808	39.73192
-5.40866	GO:0090218	positive regulation of lipid kinase activity	19_GO:0048518 positive regulation of biological process	-5.40866	37.58425	11.95982	37	4	4.938272	2.407398	2147|2322|5747|6714	F2|FLT3|PTK2|SRC	-3.77863	41	0	0	-7.04885	38.62826
-5.40866	GO:0048009	insulin-like growth factor receptor signaling pathway	19_GO:0023052 signaling	-5.40866	37.58425	11.95982	37	4	4.938272	2.407398	207|367|3480|5295	AKT1|AR|IGF1R|PIK3R1	-3.77863	54	0	0	-6.06931	37.58425
-5.40866	GO:0060416	response to growth hormone	19_GO:0050896 response to stimulus	-5.40866	37.58425	11.95982	37	4	4.938272	2.407398	207|5295|5747|5770	AKT1|PIK3R1|PTK2|PTPN1	-3.77863	62	1	1	-5.40866	45.34622
-5.403	GO:0031331	positive regulation of cellular catabolic	19_GO:0048518 positive	-5.403	7.310488	7.0	428	9	11.11111	3.491885	207|351|1612|2932|3643|3791|	AKT1|APP|DAPK1|GSK3B|INSR|KDR|PLK1|PTK2|	-3.7746	43	0	0	-6.7	45.34622

48		process	regulation of biological process	48		65689					5347|5747|5770	PTPN1	2				6848	
-5.3995	GO:2001057	reactive nitrogen species metabolic process	19_GO:0008152 metabolic process	-5.3995	21.19843	9.8378887	82	5	6.17284	2.674018	207|1545|3643|4843|5743	AKT1|CYP1B1|INSR|NOS2|PTGS2	-3.77286	19	0	0	-10.8808	39.73192
-5.39937	GO:0010001	glial cell differentiation	19_GO:0032502 developmental process	-5.39937	10.81591	7.939769	225	7	8.641975	3.122032	207|351|983|1021|1956|2147|4137	AKT1|APP|CDK1|CDK6|EGFR|F2|MAPT	-3.77286	10	0	0	-13.3451	41.71852
-5.38747	GO:0045596	negative regulation of cell differentiation	19_GO:0048519 negative regulation of biological process	-5.38747	5.599118	6.535225	683	11	13.58025	3.80643	351|1021|1956|2147|2932|3558|4318|5295|5467|7498|10062	APP|CDK6|EGFR|F2|GSK3B|IL2|MMP9|PIK3R1|PPARD|XDH|NR1H3	-3.76212	24	0	0	-9.65495	14.76524
-5.37079	GO:0043254	regulation of protein-containing complex assembly	19_GO:0050789 regulation of biological process	-5.37079	7.242798	7.022993	432	9	11.11111	3.491885	142|246|2099|2932|4137|4233|4314|6714|6850	PARP1|ALOX15|ESR1|GSK3B|MAPT|MET|MMP3|SRC|SYK	-3.7466	50	0	0	-6.31767	11.54039
-5.3501	GO:0051384	response to glucocorticoid	19_GO:0050896 response to stimulus	-5.3501	14.09409	8.578168	148	6	7.407407	2.909904	383|1956|2322|2908|5743|7298	ARG1|EGFR|FLT3|NR3C1|PTGS2|TYMS	-3.72707	29	0	0	-8.87872	35.96424
-5.330	GO:0045785	positive regulation of cell adhesion	19_GO:0022610 biological	-5.330	7.159929	6.9	437	9	11.11111	3.491885	207|240|246|1021|2932|3558|3791|	AKT1|ALOX5|ALOX15|CDK6|GSK3B|IL2|KDR|SRC|SYK	-3.7085	25	0	0	-9.4	22.67311

41			adhesion	41		70382					6714|6850		3				8567	
-5.32753	GO:0048514	blood vessel morphogenesis	19_GO:0032502 developmental process	-5.32753	5.518323	6.468425	693	11	13.58025	3.80643	207|240|1545|3791|4313|4638|5294|5743|5747|6850|7498	AKT1|ALOX5|CYP1B1|KDR|MMP2|MYLK|PIK3CG|PTGS2|PTK2|SYK|XDH	-3.70681	20	0	0	-10.2592	15.71319
-5.3165	GO:0001959	regulation of cytokine-mediated signaling pathway	19_GO:0023052 signaling	-5.3165	13.90617	8.512299	150	6	7.407407	2.909904	383|558|4321|5770|6850|10062	ARG1|AXL|MMP12|PTPN1|SYK|NR1H3	-3.69693	55	0	0	-5.98599	20.05698
-5.31526	GO:0016572	histone phosphorylation	19_GO:0008152 metabolic process	-5.31526	35.65685	11.63232	39	4	4.938272	2.407398	891|983|1017|9212	CCNB1|CDK1|CDK2|AURKB	-3.69684	34	0	0	-7.56608	35.65685
-5.29988	GO:2001056	positive regulation of cysteine-type endopeptidase activity	19_GO:0048518 positive regulation of biological process	-5.29988	13.81408	8.479834	151	6	7.407407	2.909904	239|1509|1612|4137|6850|7498	ALOX12|CTSD|DAPK1|MAPT|SYK|XDH	-3.68359	8	0	0	-14.0025	18.03867
-5.29973	GO:0010594	regulation of endothelial cell migration	19_GO:0040011 locomotion	-5.29973	10.44455	7.775126	233	7	8.641975	3.122032	207|239|3791|4233|5294|5743|5747	AKT1|ALOX12|KDR|MET|PIK3CG|PTGS2|PTK2	-3.68359	20	0	0	-10.2592	15.71319
-5.297	GO:2000117	negative regulation of cysteine-type	19_GO:0048519 negative	-5.297	20.21246	9.5	86	5	6.17284	2.674018	207|1457|4318|5743|6714	AKT1|CSNK2A1|MMP9|PTGS2|SRC	-3.6824	21	0	0	-9.9	27.26701

42		endopeptidase activity	regulation of biological process	42		83858							3				8291	
-5.28754	GO:0007160	cell-matrix adhesion	19_GO:0022610 biological adhesion	-5.28754	10.39992	7.755107	234	7	8.641975	3.122032	1021|2932|3791|4321|5295|5747|6714	CDK6|GSK3B|KDR|MMP12|PIK3R1|PTK2|SRC	-3.67368	41	0	0	-7.04885	38.62826
-5.27269	GO:0034103	regulation of tissue remodeling	19_GO:0032501 multicellular organismal process	-5.27269	19.98013	9.523022	87	5	6.17284	2.674018	760|1956|3558|6714|6850	CA2|EGFR|IL2|SRC|SYK	-3.65996	36	0	0	-7.34456	54.89279
-5.27045	GO:0071276	cellular response to cadmium ion	19_GO:0050896 response to stimulus	-5.27045	34.76543	11.4777	40	4	4.938272	2.407398	207|1956|4318|8644	AKT1|EGFR|MMP9|AKR1C3	-3.65885	14	0	0	-12.724	34.76543
-5.26699	GO:0016125	sterol metabolic process	19_GO:0008152 metabolic process	-5.26699	13.6335	8.415821	153	6	7.407407	2.909904	351|1080|1545|1588|3156|5467	APP|CFTR|CYP1B1|CYP19A1|HMGCR|PPARD	-3.65652	28	0	0	-8.92043	22.1907
-5.2513	GO:1901617	organic hydroxy compound biosynthetic process	19_GO:0008152 metabolic process	-5.2513	10.26827	7.695772	237	7	8.641975	3.122032	239|246|1080|3156|3292|7299|8644	ALOX12|ALOX15|CFTR|HMGCR|HSD17B1|TYR|AKR1C3	-3.6425	18	0	0	-10.9633	18.38557
-5.25072	GO:0006606	protein import into nucleus	19_GO:0051179 localization	-5.25072	13.54497	8.38	154	6	7.407407	2.909904	207|983|4321|5295|5743|6850	AKT1|CDK1|MMP12|PIK3R1|PTGS2|SYK	-3.6425	16	0	0	-11.555	31.96821

						4263											8	
-5.24826	GO:1901657	glycosyl compound metabolic process	19_GO:0008152 metabolic process	-5.24826	19.75309	9.463193	88	5	6.17284	2.674018	1645|1646|7498|8644|57016	AKR1C1|AKR1C2|XDH|AKR1C3|AKR1B10	-3.64115	12	0	0	-13.14	260.7407
-5.22783	GO:0022407	regulation of cell-cell adhesion	19_GO:0022610 biological adhesion	-5.22783	6.953086	6.837392	450	9	11.11111	3.491885	207|239|240|246|383|3558|5747|6714|6850	AKT1|ALOX12|ALOX5|ALOX15|ARG1|IL2|PTK2|SRC|SYK	-3.62183	25	0	0	-9.48567	22.67311
-5.22413	GO:1900407	regulation of cellular response to oxidative stress	19_GO:0050896 response to stimulus	-5.22413	19.53114	9.4043445	89	5	6.17284	2.674018	142|207|240|4233|4314	PARP1|AKT1|ALOX5|MET|MMP3	-3.61924	63	1	1	-5.22413	23.17695
-5.19231	GO:0051709	regulation of killing of cells of other organism	19_GO:0044419 biological process involved in interspecies interaction between organisms	-5.19231	80.22792	15.34596	13	3	3.703704	2.098362	383|4843|6850	ARG1|NOS2|SYK	-3.58935	56	0	0	-5.89598	130.3704
-5.19201	GO:0002790	peptide secretion	19_GO:0051179 localization	-5.19201	10.05612	7.5992	242	7	8.641975	3.122032	134|240|1080|1956|3156|4843|5467	ADORA1|ALOX5|CFTR|EGFR|HMGCR|NOS2|PPARD	-3.58935	28	0	0	-8.92043	22.1907
-5.18677	GO:0051170	import into nucleus	19_GO:0051179 localization	-5.18677	13.20206	8.26609	158	6	7.407407	2.909904	207|983|4321|5295|5743|6850	AKT1|CDK1|MMP12|PIK3R1|PTGS2|SYK	-3.58521	16	0	0	-11.5558	31.96821

						04												
-5.17671	GO:0002065	columnar/cuboidal epithelial cell differentiation	19_GO:0032502 developmental process	-5.17671	19.10189	9.289479	91	5	6.17284	2.674018	207|1021|2932|6714|7298	AKT1|CDK6|GSK3B|SRC|TYMS	-3.57625	43	0	0	-6.76848	45.34622
-5.15342	GO:0097306	cellular response to alcohol	19_GO:0050896 response to stimulus	-5.15342	18.89426	9.233411	92	5	6.17284	2.674018	196|207|1080|1646|8644	AHR|AKT1|CFTR|AKR1C2|AKR1C3	-3.55433	12	0	0	-13.14	260.7407
-5.1526	GO:0010638	positive regulation of organelle organization	19_GO:0048518 positive regulation of biological process	-5.1526	5.932668	6.480986	586	10	12.34568	3.65512	246|891|2932|3643|3791|4137|4233|4318|6714|9212	ALOX15|CCNB1|GSK3B|INSR|KDR|MAPT|MET|MMP9|SRC|AURKB	-3.55433	43	0	0	-6.76848	45.34622
-5.14284	GO:0014002	astrocyte development	19_GO:0032502 developmental process	-5.14284	32.33994	11.04608	43	4	4.938272	2.407398	351|1021|1956|4137	APP|CDK6|EGFR|MAPT	-3.54567	10	0	0	-13.3451	41.71852
-5.12454	GO:0060759	regulation of response to cytokine stimulus	19_GO:0050896 response to stimulus	-5.12454	12.87609	8.141935	162	6	7.407407	2.909904	383|558|4321|5770|6850|10062	ARG1|AXL|MMP12|PTPN1|SYK|NR1H3	-3.52845	55	0	0	-5.98599	20.05698
-5.10553	GO:0045861	negative regulation of proteolysis	19_GO:0048519 negative regulation of biological process	-5.10553	7.923745	7.0107	351	8	9.876543	3.314965	207|351|1457|2147|4318|5743|6714|10062	AKT1|APP|CSNK2A1|F2|MMP9|PTGS2|SRC|NR1H3	-3.51053	21	0	0	-9.98291	27.26701

						14												
-5.1024	GO:0045124	regulation of bone resorption	19_GO:0032501 multicellular organismal process	-5.1024	31.60494	10.91193	44	4	4.938272	2.407398	760|1956|6714|6850	CA2|EGFR|SRC|SYK	-3.50957	36	0	0	-7.34456	54.89279
-5.1024	GO:0060443	mammary gland morphogenesis	19_GO:0032502 developmental process	-5.1024	31.60494	10.91193	44	4	4.938272	2.407398	367|383|2099|6714	AR|ARG1|ESR1|SRC	-3.50957	60	0	0	-5.48956	45.34622
-5.09404	GO:2001242	regulation of intrinsic apoptotic signaling pathway	19_GO:0023052 signaling	-5.09404	12.71906	8.084014	164	6	7.407407	2.909904	142|207|4318|5743|5770|6714	PARP1|AKT1|MMP9|PTGS2|PTPN1|SRC	-3.50229	21	0	0	-9.98291	27.26701
-5.06395	GO:0030307	positive regulation of cell growth	19_GO:0040007 growth	-5.06395	12.56582	8.027092	166	6	7.407407	2.909904	207|1457|1956|2147|3558|4137	AKT1|CSNK2A1|EGFR|F2|IL2|MAPT	-3.47647	24	0	0	-9.65495	14.76524
-5.06395	GO:2000058	regulation of ubiquitin-dependent protein catabolic process	19_GO:0050789 regulation of biological process	-5.06395	12.56582	8.027092	166	6	7.407407	2.909904	207|1017|1457|2932|5347|5747	AKT1|CDK2|CSNK2A1|GSK3B|PLK1|PTK2	-3.47647	43	0	0	-6.76848	45.34622
-5.06292	GO:0003254	regulation of membrane depolarization	19_GO:0050789 regulation of biological process	-5.06292	30.90261	10.782	45	4	4.938272	2.407398	142|239|3791|6714	PARP1|ALOX12|KDR|SRC	-3.47647	40	0	0	-7.12867	115.8848

						18										
-5.06287	GO:0051304	chromosome separation	19_GO:0009987 cellular process	-5.06287	18.107	9.017681	96	5	6.17284	2.674018	891|1457|5347|7153|9212	CCNB1|CSNK2A1|PLK1|TOP2A|AURKB	-3.47647	34	0	0	-7.56608	35.65685
-5.06287	GO:0036473	cell death in response to oxidative stress	19_GO:0050896 response to stimulus	-5.06287	18.107	9.017681	96	5	6.17284	2.674018	142|207|1545|4233|4314	PARP1|AKT1|CYP1B1|MET|MMP3	-3.47647	63	0	0	-5.22413	23.17695
-5.04905	GO:1903034	regulation of response to wounding	19_GO:0050896 response to stimulus	-5.04905	12.49057	7.998997	167	6	7.407407	2.909904	239|240|2147|3156|4638|5747	ALOX12|ALOX5|F2|HMGCR|MYLK|PTK2	-3.46371	59	0	0	-5.59989	17.82843
-5.04086	GO:0000079	regulation of cyclin-dependent protein serine/threonine kinase activity	19_GO:0050789 regulation of biological process	-5.04086	17.92033	8.965775	97	5	6.17284	2.674018	207|891|1956|5347|6714	AKT1|CCNB1|EGFR|PLK1|SRC	-3.45658	58	0	0	-5.66793	43.45679
-5.03446	GO:0009306	protein secretion	19_GO:0051179 localization	-5.03446	7.747171	6.910334	359	8	9.876543	3.314965	43|240|1080|1956|3156|4843|5467|10062	ACHE|ALOX5|CFTR|EGFR|HMGCR|NOS2|PPARD|NR1H3	-3.45125	28	0	0	-8.92043	22.1907
-5.03292	GO:0034976	response to endoplasmic reticulum stress	19_GO:0050896 response to stimulus	-5.03292	9.506173	7.3431	256	7	8.641975	3.122032	240|246|1080|2932|5295|5770|10062	ALOX5|ALOX15|CFTR|GSK3B|PIK3R1|PTPN1|NR1H3	-3.45076	62	0	0	-5.40866	45.34622

						31												
-5.02571	GO:0035592	establishment of protein localization to extracellular region	19_GO:0051179 localization	-5.02571	7.725652	6.898005	360	8	9.876543	3.314965	43|240|1080|1956|3156|4843|5467|10062	ACHE|ALOX5|CFTR|EGFR|HMGCR|NOS2|PPARD|NR1H3	-3.4446	28	0	0	-8.92043	22.1907
-5.01909	GO:2001243	negative regulation of intrinsic apoptotic signaling pathway	19_GO:0048519 negative regulation of biological process	-5.01909	17.73747	8.914638	98	5	6.17284	2.674018	207|4318|5743|5770|6714	AKT1|MMP9|PTGS2|PTPN1|SRC	-3.43903	21	0	0	-9.98291	27.26701
-4.99756	GO:1902882	regulation of response to oxidative stress	19_GO:0050896 response to stimulus	-4.99756	17.5583	8.864251	99	5	6.17284	2.674018	142|207|240|4233|4314	PARP1|AKT1|ALOX5|MET|MMP3	-3.41855	63	0	0	-5.22413	23.17695
-4.97626	GO:0045833	negative regulation of lipid metabolic process	19_GO:0048519 negative regulation of biological process	-4.97626	17.38272	8.814595	100	5	6.17284	2.674018	134|207|238|5294|8644	ADORA1|AKT1|ALK|PIK3CG|AKR1C3	-3.39829	47	0	0	-6.43293	55.62469
-4.96516	GO:0071692	protein localization to extracellular region	19_GO:0051179 localization	-4.96516	7.578296	6.81301	367	8	9.876543	3.314965	43|240|1080|1956|3156|4843|5467|10062	ACHE|ALOX5|CFTR|EGFR|HMGCR|NOS2|PPARD|NR1H3	-3.38824	28	0	0	-8.92043	22.1907
-4.95518	GO:1904029	regulation of cyclin-dependent protein kinase activity	19_GO:0050789 regulation of biological process	-4.95518	17.21061	8.765655	101	5	6.17284	2.674018	207|891|1956|5347|6714	AKT1|CCNB1|EGFR|PLK1|SRC	-3.3793	58	0	0	-5.66793	43.45679

						3												
-4.94984	GO:0030261	chromosome condensation	19_GO:0009987 cellular process	-4.94984	28.97119	10.417008	48	4	4.938272	2.407398	891|983|5347|7153	CCNB1|CDK1|PLK1|TOP2A	-3.37706	34	0	0	-7.56608	35.65685
-4.94984	GO:0033628	regulation of cell adhesion mediated by integrin	19_GO:0022610 biological adhesion	-4.94984	28.97119	10.417008	48	4	4.938272	2.407398	1545|5294|5747|6850	CYP1B1|PIK3CG|PTK2|SYK	-3.37706	42	0	0	-6.93111	31.60494
-4.94984	GO:0002673	regulation of acute inflammatory response	19_GO:0050896 response to stimulus	-4.94984	28.97119	10.417008	48	4	4.938272	2.407398	134|5294|5743|9536	ADORA1|PIK3CG|PTGS2|PTGES	-3.37706	64	1	1	-4.94984	45.34622
-4.94738	GO:0006694	steroid biosynthetic process	19_GO:0008152 metabolic process	-4.94738	11.98808	7.808836	174	6	7.407407	2.909904	1080|1586|1588|3156|3292|8644	CFTR|CYP17A1|CYP19A1|HMGCR|HSD17B1|AKR1C3	-3.37563	18	0	0	-10.9633	18.38557
-4.93226	GO:0006091	generation of precursor metabolites and energy	19_GO:0008152 metabolic process	-4.93226	6.385488	6.459201	490	9	11.11111	3.491885	207|351|891|983|1588|2932|3643|4843|5467	AKT1|APP|CCNB1|CDK1|CYP19A1|GSK3B|INSR|NOS2|PPARD	-3.36154	35	0	0	-7.56218	38.62826
-4.90401	GO:0051348	negative regulation of transferase activity	19_GO:0065007 biological regulation	-4.90401	9.080523	7.13883	268	7	8.641975	3.122032	207|2932|3156|4137|5347|5770|6714	AKT1|GSK3B|HMGCR|MAPT|PLK1|PTPN1|SRC	-3.33452	17	0	0	-11.2655	11.58848

						5												
-4.9032	GO:0042574	retinal metabolic process	19_GO:0008152 metabolic process	-4.9032	65.18519	13.79311	16	3	3.703704	2.098362	1545|1645|8644	CYP1B1|AKR1C1|AKR1C3	-3.33452	12	0	0	-13.14	260.7407
-4.87854	GO:0042572	retinol metabolic process	19_GO:0008152 metabolic process	-4.87854	27.81235	10.19177	50	4	4.938272	2.407398	1545|7276|8644|57016	CYP1B1|TTR|AKR1C3|AKR1B10	-3.31089	12	0	0	-13.14	260.7407
-4.85005	GO:1905952	regulation of lipid localization	19_GO:0051179 localization	-4.85005	11.52445	7.629271	181	6	7.407407	2.909904	207|1588|5467|6850|9536|10062	AKT1|CYP19A1|PPARD|SYK|PTGES|NR1H3	-3.28342	33	0	0	-8.21676	32.59259
-4.84402	GO:0048013	ephrin receptor signaling pathway	19_GO:0023052 signaling	-4.84402	27.26701	10.08401	51	4	4.938272	2.407398	4313|4318|5747|6714	MMP2|MMP9|PTK2|SRC	-3.27941	21	0	0	-9.98291	27.26701
-4.84402	GO:0046850	regulation of bone remodeling	19_GO:0032501 multicellular organismal process	-4.84402	27.26701	10.08401	51	4	4.938272	2.407398	760|1956|6714|6850	CA2|EGFR|SRC|SYK	-3.27941	36	0	0	-7.34456	54.89279
-4.83648	GO:0010950	positive regulation of endopeptidase activity	19_GO:0048518 positive regulation of biological process	-4.83648	11.46113	7.60442	182	6	7.407407	2.909904	239|1509|1612|4137|6850|7498	ALOX12|CTSD|DAPK1|MAPT|SYK|XDH	-3.27289	8	0	0	-14.0025	18.03867

-4.83173	GO:0043270	positive regulation of ion transport	19_GO:0048518 positive regulation of biological process	-4.83173	8.849383	47.025493	275	7	8.641975	3.122032	134|207|1080|2147|4638|5243|9536	ADORA1|AKT1|CFTR|F2|MYLK|ABCB1|PTGES	-3.26996	27	0	0	-8.92369	24.25495
-4.83153	GO:0030099	myeloid cell differentiation	19_GO:0002376 immune system process	-4.83153	7.261709	6.626892	383	8	9.876543	3.314965	142|351|760|1021|2101|4318|5295|6714	PARP1|APP|CA2|CDK6|ESRRA|MMP9|PIK3R1|SRC	-3.26996	37	0	0	-7.31428	16.2963
-4.81528	GO:0042326	negative regulation of phosphorylation	19_GO:0048519 negative regulation of biological process	-4.81528	7.223986	6.604383	385	8	9.876543	3.314965	207|891|3156|3558|4137|5347|5770|7498	AKT1|CCNB1|HMGCR|IL2|MAPT|PLK1|PTPN1|XDH	-3.25571	17	0	0	-11.2655	11.58848
-4.81528	GO:0045786	negative regulation of cell cycle	19_GO:0048519 negative regulation of biological process	-4.81528	7.223986	6.604383	385	8	9.876543	3.314965	891|983|1017|1021|1956|5347|5743|9212	CCNB1|CDK1|CDK2|CDK6|EGFR|PLK1|PTGS2|AURKB	-3.25571	34	0	0	-7.56608	35.65685
-4.81362	GO:0034440	lipid oxidation	19_GO:0008152 metabolic process	-4.81362	16.09511	8.441633	108	5	6.17284	2.674018	207|239|240|246|5467	AKT1|ALOX12|ALOX5|ALOX15|PPARD	-3.25505	11	0	0	-13.1836	260.7407
-4.81145	GO:0007548	sex differentiation	19_GO:0022414 reproductive process	-4.81145	8.785488	6.99385	277	7	8.641975	3.122032	367|558|1586|1588|2099|6714|8644	AR|AXL|CYP17A1|CYP19A1|ESR1|SRC|AKR1C3	-3.25388	60	0	0	-5.48956	45.34622

-4.79967	GO:0043086	negative regulation of catalytic activity	19_GO:0065007 biological regulation	-4.79967	4.846892	5.886585	789	11	13.58025	3.80643	207|351|1457|2932|3156|4137|4318|5347|5743|5770|6714	AKT1|APP|CSNK2A1|GSK3B|HMGCR|MAPT|MMP9|PLK1|PTGS2|PTPN1|SRC	-3.24309	17	0	0	-11.2655	11.58848
-4.78134	GO:0033044	regulation of chromosome organization	19_GO:0050789 regulation of biological process	-4.78134	8.691358	6.946979	280	7	8.641975	3.122032	142|891|4137|5347|6714|7153|9212	PARP1|CCNB1|MAPT|PLK1|SRC|TOP2A|AURKB	-3.22576	34	0	0	-7.56608	35.65685
-4.77707	GO:0007566	embryo implantation	19_GO:0051704 multi-organism process	-4.77707	26.23806	9.877499	53	4	4.938272	2.407398	4313|4318|5467|5743	MMP2|MMP9|PPARD|PTGS2	-3.22346	19	0	0	-10.8808	39.73192
-4.77707	GO:0071715	icosanoid transport	19_GO:0051179 localization	-4.77707	26.23806	9.877499	53	4	4.938272	2.407398	4363|4843|6850|9536	ABCC1|NOS2|SYK|PTGES	-3.22346	33	0	0	-8.21676	32.59259
-4.77138	GO:1902105	regulation of leukocyte differentiation	19_GO:0002376 immune system process	-4.77138	8.660428	6.931511	281	7	8.641975	3.122032	558|760|1021|2101|3558|5295|6850	AXL|CA2|CDK6|ESRRA|IL2|PIK3R1|SYK	-3.21876	25	0	0	-9.48567	22.67311
-4.76335	GO:0050865	regulation of cell activation	19_GO:0050789 regulation of biological process	-4.76335	5.348528	6.024402	650	10	12.34568	3.65512	196|207|239|383|558|2147|3558|6714|6850|10062	AHR|AKT1|ALOX12|ARG1|AXL|F2|IL2|SRC|SYK|NR1H3	-3.21171	25	0	0	-9.48567	22.67311

-4.76146	GO:1903532	positive regulation of secretion by cell	19_GO:0048518 positive regulation of biological process	-4.76146	8.629717	6.916122	282	7	8.641975	3.122032	43|1080|1588|1956|5467|6850|9536	ACHE|CFTR|CYP19A1|EGFR|PPARD|SYK|PTGES	-3.21081	28	0	0	-8.92043	22.1907
-4.75593	GO:0015718	monocarboxylic acid transport	19_GO:0051179 localization	-4.75593	15.6601	8.311897	111	5	6.17284	2.674018	1645|4843|6850|9429|9536	AKR1C1|NOS2|SYK|ABCG2|PTGES	-3.20625	33	0	0	-8.21676	32.59259
-4.7415	GO:0033189	response to vitamin A	19_GO:0050896 response to stimulus	-4.7415	57.94239	12.9794	18	3	3.703704	2.098362	383|5467|7298	ARG1|PPARD|TYMS	-3.19467	65	1	1	-4.7415	57.94239
-4.74142	GO:0031400	negative regulation of protein modification process	19_GO:0048519 negative regulation of biological process	-4.74142	6.040326	6.218693	518	9	11.11111	3.491885	207|891|2932|3156|3558|4137|5347|5770|7498	AKT1|CCNB1|GSK3B|HMGCR|IL2|MAPT|PLK1|PTPN1|XDH	-3.19467	17	0	0	-11.2655	11.58848
-4.74142	GO:0090066	regulation of anatomical structure size	19_GO:0065007 biological regulation	-4.74142	6.040326	6.218693	518	9	11.11111	3.491885	134|207|246|1956|2932|3156|4137|5467|5743	ADORA1|AKT1|ALOX15|EGFR|GSK3B|HMGCR|MAPT|PPARD|PTGS2	-3.19467	19	0	0	-10.8808	39.73192
-4.73707	GO:0002526	acute inflammatory response	19_GO:0050896 response to stimulus	-4.73707	15.52028	8.26977	112	5	6.17284	2.674018	134|2147|5294|5743|9536	ADORA1|F2|PIK3CG|PTGS2|PTGES	-3.19129	64	0	0	-4.94984	45.34622
-4.69237	GO:0050731	positive regulation of peptidyl-tyrosine phosphorylation	19_GO:0048518 positive regulation of	-4.69237	10.80791	7.34	193	6	7.407407	2.909904	134|2322|3558|5770|6714|6850	ADORA1|FLT3|IL2|PTPN1|SRC|SYK	-3.14755	4	0	0	-17.191	66.21987

			biological process			3318											1	
-4.68152	GO:0071385	cellular response to glucocorticoid stimulus	19_GO:0050896 response to stimulus	-4.68152	24.83245	9.588242	56	4	4.938272	2.407398	383|1956|2322|2908	ARG1|EGFR|FLT3|NR3C1	-3.13863	29	0	0	-8.87872	35.96424
-4.68152	GO:0046622	positive regulation of organ growth	19_GO:0040007 growth	-4.68152	24.83245	9.588242	56	4	4.938272	2.407398	207|891|983|5292	AKT1|CCNB1|CDK1|PIM1	-3.13863	35	0	0	-7.56218	38.62826
-4.67971	GO:0032872	regulation of stress-activated MAPK cascade	19_GO:0023052 signaling	-4.67971	10.7522	7.320629	194	6	7.407407	2.909904	207|351|1956|3156|3480|7498	AKT1|APP|EGFR|HMGCR|IGF1R|XDH	-3.13779	10	0	0	-13.3451	41.71852
-4.66777	GO:0045780	positive regulation of bone resorption	19_GO:0048518 positive regulation of biological process	-4.66777	54.89279	12.62112	19	3	3.703704	2.098362	760|1956|6850	CA2|EGFR|SYK	-3.1268	36	0	0	-7.34456	54.89279
-4.6634	GO:0021782	glial cell development	19_GO:0032502 developmental process	-4.6634	14.9851	8.106528	116	5	6.17284	2.674018	207|351|1021|1956|4137	AKT1|APP|CDK6|EGFR|MAPT	-3.12339	10	0	0	-13.3451	41.71852
-4.65461	GO:0090276	regulation of peptide hormone secretion	19_GO:0051179 localization	-4.65461	10.64248	7.27	196	6	7.407407	2.909904	240|1080|1956|3156|4843|5467	ALOX5|CFTR|EGFR|HMGCR|NOS2|PPARD	-3.11652	28	0	0	-8.9204	22.1907

						5744											3	
-4.65461	GO:0071674	mononuclear cell migration	19_GO:0040011 locomotion	-4.65461	10.64248	7.275744	196	6	7.407407	2.909904	207|240|351|3577|5294|5747	AKT1|ALOX5|APP|CXCR1|PIK3CG|PTK2	-3.11652	42	0	0	-6.93111	31.60494
-4.65086	GO:0043551	regulation of phosphatidylinositol 3-kinase activity	19_GO:0050789 regulation of biological process	-4.65086	24.39679	9.49681	57	4	4.938272	2.407398	2322|5295|5747|6714	FLT3|PIK3R1|PTK2|SRC	-3.11372	41	0	0	-7.04885	38.62826
-4.64541	GO:0044403	biological process involved in symbiotic interaction	19_GO:0044419 biological process involved in interspecies interaction between organisms	-4.64541	8.277484	6.737176	294	7	8.641975	3.122032	383|558|983|1956|2147|3643|6714	ARG1|AXL|CDK1|EGFR|F2|INSR|SRC	-3.10922	57	0	0	-5.76668	22.87199
-4.64217	GO:0070302	regulation of stress-activated protein kinase signaling cascade	19_GO:0023052 signaling	-4.64217	10.58846	7.253544	197	6	7.407407	2.909904	207|351|1956|3156|3480|7498	AKT1|APP|EGFR|HMGCR|IGF1R|XDH	-3.10692	10	0	0	-13.3451	41.71852
-4.62658	GO:0050890	cognition	19_GO:0032501 multicellular organismal process	-4.62658	8.221555	6.708341	296	7	8.641975	3.122032	134|351|1956|3156|3643|4137|5743	ADORA1|APP|EGFR|HMGCR|INSR|MAPT|PTGS2	-3.09228	45	0	0	-6.56867	52.14815
-4.59809	GO:0032930	positive regulation of superoxide anion generation	19_GO:0048518 positive regulation of biological process	-4.59809	52.14815	12.28	20	3	3.703704	2.098362	1956|4137|6850	EGFR|MAPT|SYK	-3.06754	7	0	0	-14.1215	57.94239

						975												
−4.59809	GO:0043651	linoleic acid metabolic process	19_GO:0008152 metabolic process	−4.59809	52.14815	12.28975	20	3	3.703704	2.098362	239|240|246	ALOX12|ALOX5|ALOX15	−3.06754	11	0	0	−13.1836	260.7407
−4.59809	GO:0071379	cellular response to prostaglandin stimulus	19_GO:0050896 response to stimulus	−4.59809	52.14815	12.28975	20	3	3.703704	2.098362	207|1646|8644	AKT1|AKR1C2|AKR1C3	−3.06754	12	0	0	−13.14	260.7407
−4.59809	GO:0010042	response to manganese ion	19_GO:0050896 response to stimulus	−4.59809	52.14815	12.28975	20	3	3.703704	2.098362	351|383|5743	APP|ARG1|PTGS2	−3.06754	45	0	0	−6.56867	52.14815
−4.59124	GO:0002820	negative regulation of adaptive immune response	19_GO:0002376 immune system process	−4.59124	23.56978	9.320792	59	4	4.938272	2.407398	196|246|383|3558	AHR|ALOX15|ARG1|IL2	−3.06163	56	0	0	−5.89598	130.3704
−4.58935	GO:0006913	nucleocytoplasmic transport	19_GO:0051179 localization	−4.58935	8.111934	6.651477	300	7	8.641975	3.122032	207|983|2932|4321|5295|5743|6850	AKT1|CDK1|GSK3B|MMP12|PIK3R1|PTGS2|SYK	−3.06161	16	0	0	−11.5558	31.96821
−4.58935	GO:0051169	nuclear transport	19_GO:0051179 localization	−4.58935	8.111934	6.651	300	7	8.641975	3.122032	207|983|2932|4321|5295|5743|6850	AKT1|CDK1|GSK3B|MMP12|PIK3R1|PTGS2|SYK	−3.06161	16	0	0	−11.5558	31.96821

						477												
-4.58097	GO:0019722	calcium-mediated signaling	19_GO:0023052 signaling	-4.58097	10.32637	7.144887	202	6	7.407407	2.909904	1956|2932|3577|3791|4137|6850	EGFR|GSK3B|CXCR1|KDR|MAPT|SYK	-3.05517	7	0	0	-14.1215	57.94239
-4.58097	GO:0051054	positive regulation of DNA metabolic process	19_GO:0048518 positive regulation of biological process	-4.58097	10.32637	7.144887	202	6	7.407407	2.909904	142|207|1956|3558|6714|9212	PARP1|AKT1|EGFR|IL2|SRC|AURKB	-3.05517	40	0	0	-7.12867	115.8848
-4.58013	GO:0042063	gliogenesis	19_GO:0032502 developmental process	-4.58013	8.084984	6.637426	301	7	8.641975	3.122032	207|351|983|1021|1956|2147|4137	AKT1|APP|CDK1|CDK6|EGFR|F2|MAPT	-3.05517	10	0	0	-13.3451	41.71852
-4.57504	GO:0042752	regulation of circadian rhythm	19_GO:0048511 rhythmic process	-4.57504	14.36588	7.913513	121	5	6.17284	2.674018	134|983|2932|7153|10062	ADORA1|CDK1|GSK3B|TOP2A|NR1H3	-3.051	13	0	0	-12.7441	16.39879
-4.57094	GO:0030198	extracellular matrix organization	19_GO:0009987 cellular process	-4.57094	8.058213	6.623439	302	7	8.641975	3.122032	351|1545|4313|4314|4318|4321|4322	APP|CYP1B1|MMP2|MMP3|MMP9|MMP12|MMP13	-3.04782	6	0	0	-14.9128	124.1623
-4.56179	GO:0043062	extracellular structure organization	19_GO:0009987 cellular process	-4.56179	8.031618	6.609	303	7	8.641975	3.122032	351|1545|4313|4314|4318|4321|4322	APP|CYP1B1|MMP2|MMP3|MMP9|MMP12|MMP13	-3.03959	6	0	0	-14.9128	124.1623

						517												
-4.54504	GO:0017038	protein import	19_GO:0051179 localization	-4.54504	10.17525	7.081501	205	6	7.407407	2.909904	207|983|4321|5295|5743|6850	AKT1|CDK1|MMP12|PIK3R1|PTGS2|SYK	-3.02376	16	0	0	-11.5558	31.96821
-4.54358	GO:0045229	external encapsulating structure organization	19_GO:0009987 cellular process	-4.54358	7.978952	6.581863	305	7	8.641975	3.122032	351|1545|4313|4314|4318|4321|4322	APP|CYP1B1|MMP2|MMP3|MMP9|MMP12|MMP13	-3.02322	6	0	0	-14.9128	124.1623
-4.53371	GO:0050994	regulation of lipid catabolic process	19_GO:0050789 regulation of biological process	-4.53371	22.797	9.153277	61	4	4.938272	2.407398	134|207|238|5294	ADORA1|AKT1|ALK|PIK3CG	-3.01426	47	0	0	-6.43293	55.62469
-4.53204	GO:0071498	cellular response to fluid shear stress	19_GO:0050896 response to stimulus	-4.53204	49.6649	11.98206	21	3	3.703704	2.098362	760|5743|6714	CA2|PTGS2|SRC	-3.0135	36	0	0	-7.34456	54.89279
-4.50714	GO:1904375	regulation of protein localization to cell periphery	19_GO:0051179 localization	-4.50714	13.90617	7.767165	125	5	6.17284	2.674018	207|367|1956|5295|5347	AKT1|AR|EGFR|PIK3R1|PLK1	-2.9895	54	0	0	-6.06931	37.58425
-4.5057	GO:0046824	positive regulation of nucleocytoplasmic transport	19_GO:0048518 positive regulation of biological process	-4.5057	22.42931	9.072	62	4	4.938272	2.407398	983|2932|5295|5743	CDK1|GSK3B|PIK3R1|PTGS2	-2.98897	16	0	0	-11.5558	31.96821

						493												
−4.49865	GO:0032409	regulation of transporter activity	19_GO:0065007 biological regulation	−4.49865	7.850259	6.513817	310	7	8.641975	3.122032	351|1080|1612|4318|5243|5292|10062	APP|CFTR|DAPK1|MMP9|ABCB1|PIM1|NR1H3	−2.98311	27	0	0	−8.92369	24.25495
−4.49802	GO:0050679	positive regulation of epithelial cell proliferation	19_GO:0048518 positive regulation of biological process	−4.49802	9.980507	6.998994	209	6	7.407407	2.909904	207|367|383|1956|3791|4321	AKT1|AR|ARG1|EGFR|KDR|MMP12	−2.98311	39	0	0	−7.16624	9.980507
−4.48642	GO:0050866	negative regulation of cell activation	19_GO:0048519 negative regulation of biological process	−4.48642	9.932981	6.978715	210	6	7.407407	2.909904	239|383|558|2147|3558|10062	ALOX12|ARG1|AXL|F2|IL2|NR1H3	−2.97241	55	0	0	−5.98599	20.05698
−4.44153	GO:0007093	mitotic cell cycle checkpoint signaling	19_GO:0048519 negative regulation of biological process	−4.44153	13.47497	7.627382	129	5	6.17284	2.674018	891|983|1017|5347|9212	CCNB1|CDK1|CDK2|PLK1|AURKB	−2.92842	34	0	0	−7.56608	35.65685
−4.42932	GO:0044242	cellular lipid catabolic process	19_GO:0008152 metabolic process	−4.42932	9.701981	6.879322	215	6	7.407407	2.909904	207|1545|5294|5467|8644|57016	AKT1|CYP1B1|PIK3CG|PPARD|AKR1C3|AKR1B10	−2.9171	47	0	0	−6.43293	55.62469
−4.42444	GO:0045453	bone resorption	19_GO:0032501 multicellular organismal process	−4.42444	21.39411	8.841	65	4	4.938272	2.407398	760|1956|6714|6850	CA2|EGFR|SRC|SYK	−2.91312	36	0	0	−7.34456	54.89279

-4.41117	GO:0070661	leukocyte proliferation	19_GO:0009987 cellular process	-4.41117	7.604938	126.382186	320	7	8.641975	3.122032	196|383|2322|3558|5294|5747|6850	AHR|ARG1|FLT3|IL2|PIK3CG|PTK2|SYK	-2.90345	56	0	0	-5.89598	130.3704
-4.40948	GO:0003323	type B pancreatic cell development	19_GO:0032502 developmental process	-4.40948	45.34622	11.42725	23	3	3.703704	2.098362	207|1021|2932	AKT1|CDK6|GSK3B	-2.90345	43	0	0	-6.76848	45.34622
-4.40948	GO:0060444	branching involved in mammary gland duct morphogenesis	19_GO:0032502 developmental process	-4.40948	45.34622	11.42725	23	3	3.703704	2.098362	367|2099|6714	AR|ESR1|SRC	-2.90345	60	0	0	-5.48956	45.34622
-4.40948	GO:0060396	growth hormone receptor signaling pathway	19_GO:0023052 signaling	-4.40948	45.34622	11.42725	23	3	3.703704	2.098362	5295|5747|5770	PIK3R1|PTK2|PTPN1	-2.90345	62	0	0	-5.40866	45.34622
-4.40948	GO:0045932	negative regulation of muscle contraction	19_GO:0048519 negative regulation of biological process	-4.40948	45.34622	11.42725	23	3	3.703704	2.098362	134|5294|5743	ADORA1|PIK3CG|PTGS2	-2.90345	64	0	0	-4.94984	45.34622
-4.40943	GO:0050804	modulation of chemical synaptic transmission	19_GO:0023052 signaling	-4.40943	6.335386	6.0513	439	8	9.876543	3.314965	43|134|351|1956|2932|4137|5743|6714	ACHE|ADORA1|APP|EGFR|GSK3B|MAPT|PTGS2|SRC	-2.90345	51	0	0	-6.31611	22.67311

						74												
-4.40247	GO:0099177	regulation of trans-synaptic signaling	19_GO:0023052 signaling	-4.40247	6.320988	6.042022	440	8	9.876543	3.314965	43|134|351|1956|2932|4137|5743|6714	ACHE|ADORA1|APP|EGFR|GSK3B|MAPT|PTGS2|SRC	-2.89737	51	0	0	-6.31611	22.67311
-4.39824	GO:0019229	regulation of vasoconstriction	19_GO:0032501 multicellular organismal process	-4.39824	21.06996	8.767423	66	4	4.938272	2.407398	207|1956|3156|5743	AKT1|EGFR|HMGCR|PTGS2	-2.8949	19	0	0	-10.8808	39.73192
-4.39824	GO:0045600	positive regulation of fat cell differentiation	19_GO:0048518 positive regulation of biological process	-4.39824	21.06996	8.767423	66	4	4.938272	2.407398	207|5292|5467|5743	AKT1|PIM1|PPARD|PTGS2	-2.8949	19	0	0	-10.8808	39.73192
-4.39575	GO:0007596	blood coagulation	19_GO:0032501 multicellular organismal process	-4.39575	9.568468	6.821234	218	6	7.407407	2.909904	239|558|2147|5294|6714|6850	ALOX12|AXL|F2|PIK3CG|SRC|SYK	-2.89418	9	0	0	-13.6942	26.07407
-4.39575	GO:0045088	regulation of innate immune response	19_GO:0044419 biological process involved in interspecies interaction between organisms	-4.39575	9.568468	6.821234	218	6	7.407407	2.909904	383|4321|5770|6714|6850|10062	ARG1|MMP12|PTPN1|SRC|SYK|NR1H3	-2.89418	55	0	0	-5.98599	20.05698
-4.38468	GO:0097191	extrinsic apoptotic signaling pathway	19_GO:0023052 signaling	-4.38468	9.524776	6.80211	219	6	7.407407	2.909904	207|367|2932|3558|5295|6714	AKT1|AR|GSK3B|IL2|PIK3R1|SRC	-2.88397	43	0	0	-6.76848	45.34622

						21											
-4.37808	GO:0010565	regulation of cellular ketone metabolic process	19_GO:0050789 regulation of biological process	-4.37808	13.06971	7.493672	133	5	6.17284	2.674018	207|5467|5743|8644|10062	AKT1|PPARD|PTGS2|AKR1C3|NR1H3	-2.87825	19	0	0	-10.8808	39.73192
-4.36269	GO:0070374	positive regulation of ERK1 and ERK2 cascade	19_GO:0048518 positive regulation of biological process	-4.36269	9.438579	6.764261	221	6	7.407407	2.909904	246|351|1956|3156|3791|6714	ALOX15|APP|EGFR|HMGCR|KDR|SRC	-2.86373	4	0	0	-17.1911	66.21987
-4.35239	GO:0010288	response to lead ion	19_GO:0050896 response to stimulus	-4.35239	43.45679	11.17588	24	3	3.703704	2.098362	351|4137|5743	APP|MAPT|PTGS2	-2.85626	45	0	0	-6.56867	52.14815
-4.35239	GO:0071378	cellular response to growth hormone stimulus	19_GO:0050896 response to stimulus	-4.35239	43.45679	11.17588	24	3	3.703704	2.098362	5295|5747|5770	PIK3R1|PTK2|PTPN1	-2.85626	62	0	0	-5.40866	45.34622
-4.35178	GO:1903050	regulation of proteolysis involved in cellular protein catabolic process	19_GO:0050789 regulation of biological process	-4.35178	9.396063	6.74551	222	6	7.407407	2.909904	207|1017|1457|2932|5347|5747	AKT1|CDK2|CSNK2A1|GSK3B|PLK1|PTK2	-2.85626	43	0	0	-6.76848	45.34622
-4.35175	GO:0002831	regulation of response to biotic stimulus	19_GO:0050896 response to stimulus	-4.35175	7.442141	6.29338	327	7	8.641975	3.122032	196|383|4321|5770|6714|6850|10062	AHR|ARG1|MMP12|PTPN1|SRC|SYK|NR1H3	-2.85626	55	0	0	-5.98599	20.05698

						7											
-4.34707	GO:0031640	killing of cells of other organism	19_GO:0044419 biological process involved in interspecies interaction between organisms	-4.34707	20.45025	8.624794	68	4	4.938272	2.407398	383|2147|4843|6850	ARG1|F2|NOS2|SYK	-2.85244	56	0	0	-5.89598	130.3704
-4.34092	GO:0007599	hemostasis	19_GO:0065007 biological regulation	-4.34092	9.353928	6.726878	223	6	7.407407	2.909904	239|558|2147|5294|6714|6850	ALOX12|AXL|F2|PIK3CG|SRC|SYK	-2.84802	9	0	0	-13.6942	26.07407
-4.34092	GO:0050817	coagulation	19_GO:0032501 multicellular organismal process	-4.34092	9.353928	6.726878	223	6	7.407407	2.909904	239|558|2147|5294|6714|6850	ALOX12|AXL|F2|PIK3CG|SRC|SYK	-2.84802	9	0	0	-13.6942	26.07407
-4.33127	GO:0055080	cation homeostasis	19_GO:0065007 biological regulation	-4.33127	4.755873	5.526179	731	10	12.34568	3.65512	134|351|760|1080|1956|2099|2147|3558|3577|5294	ADORA1|APP|CA2|CFTR|EGFR|ESR1|F2|IL2|CXCR1|PIK3CG	-2.83922	38	0	0	-7.2588	41.71852
-4.32208	GO:0050805	negative regulation of synaptic transmission	19_GO:0048519 negative regulation of biological process	-4.32208	20.15387	8.555746	69	4	4.938272	2.407398	43|134|4137|5743	ACHE|ADORA1|MAPT|PTGS2	-2.83174	51	0	0	-6.31611	22.67311
-4.32208	GO:0042698	ovulation cycle	19_GO:0048511 rhythmic process	-4.32208	20.15387	8.55574	69	4	4.938272	2.407398	558|1956|2099|6714	AXL|EGFR|ESR1|SRC	-2.83174	57	0	0	-5.76668	22.87199

						6											
-4.29777	GO:0048143	astrocyte activation	19_GO:0032502 developmental process	-4.29777	41.71852	10.93953	25	3	3.703704	2.098362	351|1956|4137	APP|EGFR|MAPT	-2.80968	10	0	0	-13.3451	41.71852
-4.29777	GO:0045822	negative regulation of heart contraction	19_GO:0048519 negative regulation of biological process	-4.29777	41.71852	10.93953	25	3	3.703704	2.098362	134|3558|5294	ADORA1|IL2|PIK3CG	-2.80968	38	0	0	-7.2588	41.71852
-4.29747	GO:0061180	mammary gland epithelium development	19_GO:0032502 developmental process	-4.29747	19.86596	8.488137	70	4	4.938272	2.407398	207|367|2099|6714	AKT1|AR|ESR1|SRC	-2.80968	60	0	0	-5.48956	45.34622
-4.28666	GO:0045598	regulation of fat cell differentiation	19_GO:0032502 developmental process	-4.28666	12.50555	7.303538	139	5	6.17284	2.674018	207|240|5292|5467|5743	AKT1|ALOX5|PIM1|PPARD|PTGS2	-2.79972	19	0	0	-10.8808	39.73192
-4.27689	GO:0071695	anatomical structure maturation	19_GO:0032502 developmental process	-4.27689	9.108847	6.617499	229	6	7.407407	2.909904	351|558|891|1080|4313|7298	APP|AXL|CCNB1|CFTR|MMP2|TYMS	-2.7908	23	0	0	-9.78876	32.19021
-4.27324	GO:0150116	regulation of cell-substrate junction organization	19_GO:0050789 regulation of biological process	-4.27324	19.58616	8.42191	71	4	4.938272	2.407398	3791|5295|5747|6714	KDR|PIK3R1|PTK2|SRC	-2.78798	41	0	0	-7.04885	38.62826

						8												
-4.27184	GO:0007292	female gamete generation	19_GO:0051704 multi-organism process	-4.27184	12.41623	7.272987	140	5	6.17284	2.674018	891|5347|5743|6714|7153	CCNB1|PLK1|PTGS2|SRC|TOP2A	-2.78743	58	0	0	-5.66793	43.45679
-4.26244	GO:0098771	inorganic ion homeostasis	19_GO:0065007 biological regulation	-4.26244	4.666501	5.447486	745	10	12.34568	3.65512	134|351|760|1080|1956|2099|2147|3558|3577|5294	ADORA1|APP|CA2|CFTR|EGFR|ESR1|F2|IL2|CXCR1|PIK3CG	-2.77887	38	0	0	-7.2588	41.71852
-4.24936	GO:1905818	regulation of chromosome separation	19_GO:0050789 regulation of biological process	-4.24936	19.31413	8.357039	72	4	4.938272	2.407398	891|1457|5347|9212	CCNB1|CSNK2A1|PLK1|AURKB	-2.76747	34	0	0	-7.56608	35.65685
-4.24936	GO:0033627	cell adhesion mediated by integrin	19_GO:0022610 biological adhesion	-4.24936	19.31413	8.357039	72	4	4.938272	2.407398	1545|5294|5747|6850	CYP1B1|PIK3CG|PTK2|SYK	-2.76747	42	0	0	-6.93111	31.60494
-4.24543	GO:1903523	negative regulation of blood circulation	19_GO:0048519 negative regulation of biological process	-4.24543	40.11396	10.71675	26	3	3.703704	2.098362	134|3558|5294	ADORA1|IL2|PIK3CG	-2.76497	38	0	0	-7.2588	41.71852
-4.24519	GO:0001525	angiogenesis	19_GO:0032502 developmental process	-4.24519	5.214815	5.60499	600	9	11.11111	3.491885	207|240|1545|3791|4313|5294|5743|5747|6850	AKT1|ALOX5|CYP1B1|KDR|MMP2|PIK3CG|PTGS2|PTK2|SYK	-2.76497	20	0	0	-10.2592	15.71319

-4.22583	GO:0035924	cellular response to vascular endothelial growth factor stimulus	19_GO:0050896 response to stimulus	-4.22583	19.04955	8.293455	73	4	4.938272	2.407398	207|2322|3791|7498	AKT1|FLT3|KDR|XDH	-2.74644	66	1	1	-4.22583	21.28496
-4.21469	GO:0045930	negative regulation of mitotic cell cycle	19_GO:0048519 negative regulation of biological process	-4.21469	8.876281	6.512066	235	6	7.407407	2.909904	891|983|1017|1956|5347|9212	CCNB1|CDK1|CDK2|EGFR|PLK1|AURKB	-2.73633	34	0	0	-7.56608	35.65685
-4.21368	GO:0010821	regulation of mitochondrion organization	19_GO:0050789 regulation of biological process	-4.21368	12.07133	7.153825	144	5	6.17284	2.674018	207|2932|3791|4137|4318	AKT1|GSK3B|KDR|MAPT|MMP9	-2.73633	43	0	0	-6.76848	45.34622
-4.21323	GO:0001933	negative regulation of protein phosphorylation	19_GO:0048519 negative regulation of biological process	-4.21323	7.074361	6.088255	344	7	8.641975	3.122032	207|891|3156|3558|5347|5770|7498	AKT1|CCNB1|HMGCR|IL2|PLK1|PTPN1|XDH	-2.73633	17	0	0	-11.2655	11.58848
-4.20658	GO:0006935	chemotaxis	19_GO:0040011 locomotion	-4.20658	5.154677	5.557858	607	9	11.11111	3.491885	240|351|3577|3791|4233|4363|5294|5747|6850	ALOX5|APP|CXCR1|KDR|MET|ABCC1|PIK3CG|PTK2|SYK	-2.73051	42	0	0	-6.93111	31.60494
-4.2045	GO:0030072	peptide hormone secretion	19_GO:0051179 localization	-4.2045	8.838669	6.494861	236	6	7.407407	2.909904	240|1080|1956|3156|4843|5467	ALOX5|CFTR|EGFR|HMGCR|NOS2|PPARD	-2.72925	28	0	0	-8.92043	22.1907

-4.20265	GO:1903201	regulation of oxidative stress-induced cell death	19_GO:0050896 response to stimulus	-4.20265	18.79213	8.231122	74	4	4.938272	2.407398	142|207|4233|4314	PARP1|AKT1|MET|MMP3	-2.72822	63	0	0	-5.22413	23.17695
-4.19517	GO:0010971	positive regulation of G2/M transition of mitotic cell cycle	19_GO:0048518 positive regulation of biological process	-4.19517	38.62826	10.50626	27	3	3.703704	2.098362	351|891|983	APP|CCNB1|CDK1	-2.72321	35	0	0	-7.56218	38.62826
-4.19517	GO:0002433	immune response-regulating cell surface receptor signaling pathway involved in phagocytosis	19_GO:0002376 immune system process	-4.19517	38.62826	10.50626	27	3	3.703704	2.098362	5747|6714|6850	PTK2|SRC|SYK	-2.72321	41	0	0	-7.04885	38.62826
-4.19517	GO:0038096	Fc-gamma receptor signaling pathway involved in phagocytosis	19_GO:0002376 immune system process	-4.19517	38.62826	10.50626	27	3	3.703704	2.098362	5747|6714|6850	PTK2|SRC|SYK	-2.72321	41	0	0	-7.04885	38.62826
-4.1902	GO:0042330	taxis	19_GO:0040011 locomotion	-4.1902	5.129326	5.537881	610	9	11.11111	3.491885	240|351|3577|3791|4233|4363|5294|5747|6850	ALOX5|APP|CXCR1|KDR|MET|ABCC1|PIK3CG|PTK2|SYK	-2.71926	42	0	0	-6.93111	31.60494
-4.1896	GO:0007059	chromosome segregation	19_GO:0009987 cellular process	-4.1896	7.0132	6.053503	347	7	8.641975	3.122032	891|1457|2908|5347|7150|7153|9212	CCNB1|CSNK2A1|NR3C1|PLK1|TOP1|TOP2A|AURKB	-2.71926	34	0	0	-7.56608	35.65685

-4.17979	GO:0045913	positive regulation of carbohydrate metabolic process	19_GO:0048518 positive regulation of biological process	-4.17979	18.54156	8.1699999	75	4	4.938272	2.407398	207|351|3643|6714	AKT1|APP|INSR|SRC	-2.71027	43	0	0	-6.76848	45.34622
-4.17391	GO:0051301	cell division	19_GO:0009987 cellular process	-4.17391	5.104223	5.5180334	613	9	11.11111	3.491885	891|983|1017|1021|2908|5347|7150|7153|9212	CCNB1|CDK1|CDK2|CDK6|NR3C1|PLK1|TOP1|TOP2A|AURKB	-2.7052	34	0	0	-7.56608	35.65685
-4.16419	GO:0051403	stress-activated MAPK cascade	19_GO:0023052 signaling	-4.16419	8.691358	6.4270444	240	6	7.407407	2.909904	207|351|1956|3156|3480|7498	AKT1|APP|EGFR|HMGCR|IGF1R|XDH	-2.69629	10	0	0	-13.3451	41.71852
-4.15726	GO:0014855	striated muscle cell proliferation	19_GO:0009987 cellular process	-4.15726	18.2976	8.1100047	76	4	4.938272	2.407398	891|983|5292|5467	CCNB1|CDK1|PIM1|PPARD	-2.69017	35	0	0	-7.56218	38.62826
-4.14697	GO:0048729	tissue morphogenesis	19_GO:0032502 developmental process	-4.14697	5.062927	5.4852442	618	9	11.11111	3.491885	239|367|768|2099|3791|4233|4321|4638|6714	ALOX12|AR|CA9|ESR1|KDR|MET|MMP12|MYLK|SRC	-2.68219	60	0	0	-5.48956	45.34622
-4.14686	GO:1990776	response to angiotensin	19_GO:0050896 response to stimulus	-4.14686	37.24868	10.306697	28	3	3.703704	2.098362	760|5743|6714	CA2|PTGS2|SRC	-2.68219	36	0	0	-7.34456	54.89279

-4.14686	GO:0038094	Fc-gamma receptor signaling pathway	19_GO:0002376 immune system process	-4.14686	37.24868	10.306697	28	3	3.703704	2.098362	5747|6714|6850	PTK2|SRC|SYK	-2.68219	41	0	0	-7.04885	38.62826
-4.14339	GO:0032368	regulation of lipid transport	19_GO:0051179 localization	-4.14339	11.66625	7.011339	149	5	6.17284	2.674018	207|1588|6850|9536|10062	AKT1|CYP19A1|SYK|PTGES|NR1H3	-2.67953	33	0	0	-8.21676	32.59259
-4.13868	GO:0046649	lymphocyte activation	19_GO:0002376 immune system process	-4.13868	4.509135	5.306394	771	10	12.34568	3.65512	196|207|383|558|2322|3558|5294|5295|6714|6850	AHR|AKT1|ARG1|AXL|FLT3|IL2|PIK3CG|PIK3R1|SRC|SYK	-2.67562	25	0	0	-9.48567	22.67311
-4.13503	GO:0043536	positive regulation of blood vessel endothelial cell migration	19_GO:0040011 locomotion	-4.13503	18.05996	8.051227	77	4	4.938272	2.407398	207|239|3791|5743	AKT1|ALOX12|KDR|PTGS2	-2.67357	19	0	0	-10.8808	39.73192
-4.13503	GO:0031016	pancreas development	19_GO:0032502 developmental process	-4.13503	18.05996	8.051227	77	4	4.938272	2.407398	207|1021|2932|4233	AKT1|CDK6|GSK3B|MET	-2.67357	43	0	0	-6.76848	45.34622
-4.11599	GO:0071456	cellular response to hypoxia	19_GO:0050896 response to stimulus	-4.11599	11.51173	6.956236	151	5	6.17284	2.674018	207|891|5467|5743|6714	AKT1|CCNB1|PPARD|PTGS2|SRC	-2.65533	19	0	0	-10.8808	39.73192

-4.11312	GO:0061045	negative regulation of wound healing	19_GO:0048519 negative regulation of biological process	-4.11312	17.82843	7.9935502	78	4	4.938272	2.407398	239|240|2147|3156	ALOX12|ALOX5|F2|HMGCR	-2.65325	59	0	0	-5.59989	17.82843
-4.10244	GO:0048754	branching morphogenesis of an epithelial tube	19_GO:0032502 developmental process	-4.10244	11.436	6.9290071	152	5	6.17284	2.674018	367|2099|3791|4233|6714	AR|ESR1|KDR|MET|SRC	-2.646	60	0	0	-5.48956	45.34622
-4.10033	GO:0042759	long-chain fatty acid biosynthetic process	19_GO:0008152 metabolic process	-4.10033	35.96424	10.117791	29	3	3.703704	2.098362	239|240|246	ALOX12|ALOX5|ALOX15	-2.646	11	0	0	-13.1836	260.7407
-4.10033	GO:0071549	cellular response to dexamethasone stimulus	19_GO:0050896 response to stimulus	-4.10033	35.96424	10.117791	29	3	3.703704	2.098362	383|1956|2908	ARG1|EGFR|NR3C1	-2.646	29	0	0	-8.87872	35.96424
-4.10033	GO:0002068	glandular epithelial cell development	19_GO:0032502 developmental process	-4.10033	35.96424	10.117791	29	3	3.703704	2.098362	207|1021|2932	AKT1|CDK6|GSK3B	-2.646	43	0	0	-6.76848	45.34622
-4.10033	GO:0003309	type B pancreatic cell differentiation	19_GO:0032502 developmental process	-4.10033	35.96424	10.117791	29	3	3.703704	2.098362	207|1021|2932	AKT1|CDK6|GSK3B	-2.646	43	0	0	-6.76848	45.34622

-4.10033	GO:0005979	regulation of glycogen biosynthetic process	19_GO:0050789 regulation of biological process	-4.10033	35.96424	10.117791	29	3	3.703704	2.098362	207|2932|3643	AKT1|GSK3B|INSR	-2.646	43	0	0	-6.76848	45.34622
-4.10033	GO:0010962	regulation of glucan biosynthetic process	19_GO:0050789 regulation of biological process	-4.10033	35.96424	10.117791	29	3	3.703704	2.098362	207|2932|3643	AKT1|GSK3B|INSR	-2.646	43	0	0	-6.76848	45.34622
-4.08576	GO:0031098	stress-activated protein kinase signaling cascade	19_GO:0023052 signaling	-4.08576	8.410992	6.296033	248	6	7.407407	2.909904	207|351|1956|3156|3480|7498	AKT1|APP|EGFR|HMGCR|IGF1R|XDH	-2.63221	10	0	0	-13.3451	41.71852
-4.08228	GO:0070997	neuron death	19_GO:0009987 cellular process	-4.08228	6.74122	5.896624	361	7	8.641975	3.122032	134|142|207|351|558|2932|4137	ADORA1|PARP1|AKT1|APP|AXL|GSK3B|MAPT	-2.62951	51	0	0	-6.31611	22.67311
-4.06964	GO:0034765	regulation of ion transmembrane transport	19_GO:0051179 localization	-4.06964	5.664429	5.600019	491	8	9.876543	3.314965	207|351|1080|1612|2147|4318|5243|5294	AKT1|APP|CFTR|DAPK1|F2|MMP9|ABCB1|PIK3CG	-2.61765	27	0	0	-8.92369	24.25495
-4.05548	GO:0034694	response to prostaglandin	19_GO:0050896 response to stimulus	-4.05548	34.76543	9.938215	30	3	3.703704	2.098362	207|1646|8644	AKT1|AKR1C2|AKR1C3	-2.60583	12	0	0	-13.14	260.7407
-	GO:190	positive regulation of	19_GO:0048518	-	34.76	9.	30	3	3.70	2.098	351|891|983	APP|CCNB1|CDK1	-	35	0	0	-	38.62

4.05548	2751	cell cycle G2/M phase transition	positive regulation of biological process	4.05548	543	938215			3704	362			2.60583				7.56218	826
-4.05548	GO:0008209	androgen metabolic process	19_GO:0008152 metabolic process	-4.05548	34.76543	9.938215	30	3	3.703704	2.098362	1586|1588|2099	CYP17A1|CYP19A1|ESR1	-2.60583	67	1	1	-4.05548	34.76543
-4.0476	GO:0010951	negative regulation of endopeptidase activity	19_GO:0048519 negative regulation of biological process	-4.0476	8.277484	6.232718	252	6	7.407407	2.909904	207|351|1457|4318|5743|6714	AKT1|APP|CSNK2A1|MMP9|PTGS2|SRC	-2.59872	21	0	0	-9.98291	27.26701
-4.02835	GO:1905897	regulation of response to endoplasmic reticulum stress	19_GO:0050896 response to stimulus	-4.02835	16.95875	7.772898	82	4	4.938272	2.407398	240|5295|5770|10062	ALOX5|PIK3R1|PTPN1|NR1H3	-2.58025	62	0	0	-5.40866	45.34622
-4.01941	GO:1903362	regulation of cellular protein catabolic process	19_GO:0050789 regulation of biological process	-4.01941	8.180102	6.186143	255	6	7.407407	2.909904	207|1017|1457|2932|5347|5747	AKT1|CDK2|CSNK2A1|GSK3B|PLK1|PTK2	-2.57286	43	0	0	-6.76848	45.34622
-4.01941	GO:0007611	learning or memory	19_GO:0007610 behavior	-4.01941	8.180102	6.186143	255	6	7.407407	2.909904	351|1956|3156|3643|4137|5743	APP|EGFR|HMGCR|INSR|MAPT|PTGS2	-2.57286	45	0	0	-6.56867	52.14815
-	GO:190	regulation of	19_GO:0002376	-	6.577	5.	37	7	8.64	3.122	558|760|1021|	AXL|CA2|CDK6|ESRRA|IL2|	-	25	0	0	-	22.67

4.01577	3706	hemopoiesis	immune system process	4.01577	244	800114	0		1975	032	2101|3558|5295|6850	PIK3R1|SYK	2.56999				9.48567	311
-4.01217	GO:0001516	prostaglandin biosynthetic process	19_GO:0008152 metabolic process	-4.01217	33.64397	9.767129	31	3	3.703704	2.098362	5743|8644|9536	PTGS2|AKR1C3|PTGES	-2.56946	15	0	0	-12.2065	35.47493
-4.01217	GO:0046457	prostanoid biosynthetic process	19_GO:0008152 metabolic process	-4.01217	33.64397	9.767129	31	3	3.703704	2.098362	5743|8644|9536	PTGS2|AKR1C3|PTGES	-2.56946	15	0	0	-12.2065	35.47493
-4.01217	GO:0060045	positive regulation of cardiac muscle cell proliferation	19_GO:0040007 growth	-4.01217	33.64397	9.767129	31	3	3.703704	2.098362	891|983|5292	CCNB1|CDK1|PIM1	-2.56946	35	0	0	-7.56218	38.62826
-4.01217	GO:0060603	mammary gland duct morphogenesis	19_GO:0032502 developmental process	-4.01217	33.64397	9.767129	31	3	3.703704	2.098362	367|2099|6714	AR|ESR1|SRC	-2.56946	60	0	0	-5.48956	45.34622
-4.00784	GO:0051899	membrane depolarization	19_GO:0065007 biological regulation	-4.00784	16.75443	7.720165	83	4	4.938272	2.407398	142|239|3791|6714	PARP1|ALOX12|KDR|SRC	-2.5659	40	0	0	-7.12867	115.8848
-	GO:004	vasoconstriction	19_GO:0032501	-	16.55	7.	84	4	4.93	2.407	207|1956|3156|	AKT1|EGFR|HMGCR|PTGS2	-	19	0	0	-	39.73

3.9876	2310		multicellular organismal process	3.9876	497	668341			8272	398	5743		2.54718				10.8808	192
-3.9876	GO:1900542	regulation of purine nucleotide metabolic process	19_GO:0050789 regulation of biological process	-3.9876	16.55497	7.668341	84	4	4.938272	2.407398	142|351|3643|4843	PARP1|APP|INSR|NOS2	-2.54718	45	0	0	-6.56867	52.14815
-3.98465	GO:0036294	cellular response to decreased oxygen levels	19_GO:0050896 response to stimulus	-3.98465	10.79672	6.695493	161	5	6.17284	2.674018	207|891|5467|5743|6714	AKT1|CCNB1|PPARD|PTGS2|SRC	-2.54499	19	0	0	-10.8808	39.73192
-3.98052	GO:0030003	cellular cation homeostasis	19_GO:0065007 biological regulation	-3.98052	4.813675	5.283423	650	9	11.11111	3.491885	134|351|760|1080|2099|2147|3558|3577|5294	ADORA1|APP|CA2|CFTR|ESR1|F2|IL2|CXCR1|PIK3CG	-2.54162	38	0	0	-7.2588	41.71852
-3.97325	GO:0051091	positive regulation of DNA-binding transcription factor activity	19_GO:0050789 regulation of biological process	-3.97325	8.022792	6.110019	260	6	7.407407	2.909904	207|238|351|367|2099|2100	AKT1|ALK|APP|AR|ESR1|ESR2	-2.53511	52	0	0	-6.26187	13.03704
-3.97032	GO:2000191	regulation of fatty acid transport	19_GO:0051179 localization	-3.97032	32.59259	9.603978	32	3	3.703704	2.098362	207|6850|9536	AKT1|SYK|PTGES	-2.53368	33	0	0	-8.21676	32.59259
-3.970	GO:0035883	enteroendocrine cell differentiation	19_GO:0032502 developmental	-3.970	32.59259	9.6	32	3	3.703704	2.098362	207|1021|2932	AKT1|CDK6|GSK3B	-2.5336	43	0	0	-6.7	45.34622

32			process	32		03978							8				6848	
-3.96761	GO:0006970	response to osmotic stress	19_GO:0050896 response to stimulus	-3.96761	16.3602	7.617402	85	4	4.938272	2.407398	1956|4638|5243|5743	EGFR|MYLK|ABCB1|PTGS2	-2.53244	61	0	0	-5.47166	27.44639
-3.96757	GO:0072507	divalent inorganic cation homeostasis	19_GO:0065007 biological regulation	-3.96757	5.474871	5.466501	508	8	9.876543	3.314965	134|351|1956|2099|2147|3558|3577|5294	ADORA1|APP|EGFR|ESR1|F2|IL2|CXCR1|PIK3CG	-2.53244	38	0	0	-7.2588	41.71852
-3.9617	GO:0002009	morphogenesis of an epithelium	19_GO:0032502 developmental process	-3.9617	5.464115	5.458825	509	8	9.876543	3.314965	239|367|768|2099|3791|4233|4321|6714	ALOX12|AR|CA9|ESR1|KDR|MET|MMP12|SRC	-2.52731	60	0	0	-5.48956	45.34622
-3.95818	GO:0044282	small molecule catabolic process	19_GO:0008152 metabolic process	-3.95818	6.438043	5.716985	378	7	8.641975	3.122032	207|383|4843|5467|7498|8644|57016	AKT1|ARG1|NOS2|PPARD|XDH|AKR1C3|AKR1B10	-2.52455	19	0	0	-10.8808	39.73192
-3.95507	GO:0010466	negative regulation of peptidase activity	19_GO:0048519 negative regulation of biological process	-3.95507	7.961549	6.080374	262	6	7.407407	2.909904	207|351|1457|4318|5743|6714	AKT1|APP|CSNK2A1|MMP9|PTGS2|SRC	-2.52218	21	0	0	-9.98291	27.26701
-3.951	GO:0051346	negative regulation of hydrolase activity	19_GO:0065007 biological	-3.951	6.421056	5.7	379	7	8.641975	3.122032	207|351|1457|2932|4318|5743|	AKT1|APP|CSNK2A1|GSK3B|MMP9|PTGS2|SRC	-2.5189	21	0	0	-9.9	27.26701

08			regulation	08		06763					6714		5				8291	
-3.94788	GO:0015908	fatty acid transport	19_GO:0051179 localization	-3.94788	16.16997	7.567319	86	4	4.938272	2.407398	207|5467|6850|9536	AKT1|PPARD|SYK|PTGES	-2.51723	33	0	0	-8.21676	32.59259
-3.94788	GO:0006140	regulation of nucleotide metabolic process	19_GO:0050789 regulation of biological process	-3.94788	16.16997	7.567319	86	4	4.938272	2.407398	142|351|3643|4843	PARP1|APP|INSR|NOS2	-2.51723	45	0	0	-6.56867	52.14815
-3.94697	GO:0050806	positive regulation of synaptic transmission	19_GO:0048518 positive regulation of biological process	-3.94697	10.59922	6.621708	164	5	6.17284	2.674018	134|351|1956|2932|5743	ADORA1|APP|EGFR|GSK3B|PTGS2	-2.51707	51	0	0	-6.31611	22.67311
-3.93458	GO:0050796	regulation of insulin secretion	19_GO:0051179 localization	-3.93458	10.53498	6.597536	165	5	6.17284	2.674018	240|1080|3156|4843|5467	ALOX5|CFTR|HMGCR|NOS2|PPARD	-2.50616	28	0	0	-8.92043	22.1907
-3.93458	GO:0050680	negative regulation of epithelial cell proliferation	19_GO:0048519 negative regulation of biological process	-3.93458	10.53498	6.597536	165	5	6.17284	2.674018	240|367|1021|5467|7498	ALOX5|AR|CDK6|PPARD|XDH	-2.50616	46	0	0	-6.47634	10.53498
-3.929	GO:0002431	Fc receptor mediated stimulatory signaling	19_GO:0002376 immune system	-3.929	31.60494	9.4	33	3	3.703704	2.098362	5747|6714|6850	PTK2|SRC|SYK	-2.5036	41	0	0	-7.0	38.62826

83		pathway	process	83		48156							4				4885	
-3.92983	GO:0043552	positive regulation of phosphatidylinositol 3-kinase activity	19_GO:0048518 positive regulation of biological process	-3.92983	31.60494	9.448156	33	3	3.703704	2.098362	2322|5747|6714	FLT3|PTK2|SRC	-2.50364	41	0	0	-7.04885	38.62826
-3.92983	GO:0036336	dendritic cell migration	19_GO:0040011 locomotion	-3.92983	31.60494	9.448156	33	3	3.703704	2.098362	240|3577|5294	ALOX5|CXCR1|PIK3CG	-2.50364	42	0	0	-6.93111	31.60494
-3.92838	GO:0001776	leukocyte homeostasis	19_GO:0002376 immune system process	-3.92838	15.98411	7.51807	87	4	4.938272	2.407398	207|558|2322|3558	AKT1|AXL|FLT3|IL2	-2.50364	25	0	0	-9.48567	22.67311
-3.92838	GO:0150104	transport across blood-brain barrier	19_GO:0051179 localization	-3.92838	15.98411	7.51807	87	4	4.938272	2.407398	3643|4363|5243|9429	INSR|ABCC1|ABCB1|ABCG2	-2.50364	49	0	0	-6.34133	173.8272
-3.91004	GO:0043112	receptor metabolic process	19_GO:0008152 metabolic process	-3.91004	10.40881	6.549809	167	5	6.17284	2.674018	43|3577|3643|5770|6850	ACHE|CXCR1|INSR|PTPN1|SYK	-2.48604	44	0	0	-6.70994	13.24397
-3.90912	GO:0010232	vascular transport	19_GO:0051179 localization	-3.90912	15.80247	7.469	88	4	4.938272	2.407398	3643|4363|5243|9429	INSR|ABCC1|ABCB1|ABCG2	-2.48585	49	0	0	-6.34133	173.8272

						631												
−3.89607	GO:0006873	cellular ion homeostasis	19_GO:0065007 biological regulation	−3.89607	4.690988	5.181491	667	9	11.11111	3.491885	134|351|760|1080|2099|2147|3558|3577|5294	ADORA1|APP|CA2|CFTR|ESR1|F2|IL2|CXCR1|PIK3CG	−2.47352	38	0	0	−7.2588	41.71852
−3.89061	GO:0043276	anoikis	19_GO:0009987 cellular process	−3.89061	30.67538	9.299125	34	3	3.703704	2.098362	207|5747|6714	AKT1|PTK2|SRC	−2.4688	41	0	0	−7.04885	38.62826
−3.88582	GO:0031214	biomineral tissue development	19_GO:0110148 biomineralization	−3.88582	10.28563	6.502882	169	5	6.17284	2.674018	240|246|1080|4322|5743	ALOX5|ALOX15|CFTR|MMP13|PTGS2	−2.46546	11	0	0	−13.1836	260.7407
−3.88582	GO:0000075	cell cycle checkpoint signaling	19_GO:0048519 negative regulation of biological process	−3.88582	10.28563	6.502882	169	5	6.17284	2.674018	891|983|1017|5347|9212	CCNB1|CDK1|CDK2|PLK1|AURKB	−2.46546	34	0	0	−7.56608	35.65685
−3.8713	GO:0061097	regulation of protein tyrosine kinase activity	19_GO:0050789 regulation of biological process	−3.8713	15.4513	7.375091	90	4	4.938272	2.407398	134|351|5770|6714	ADORA1|APP|PTPN1|SRC	−2.45167	68	1	1	−3.8713	18.96296
−3.86191	GO:0110148	biomineralization	19_GO:0110148 biomineralization	−3.86191	10.16533	6.456	171	5	6.17284	2.674018	240|246|1080|4322|5743	ALOX5|ALOX15|CFTR|MMP13|PTGS2	−2.44373	11	0	0	−13.1836	260.7407

						733												
-3.86191	GO:0048469	cell maturation	19_GO:0032502 developmental process	-3.86191	10.16533	6.456733	171	5	6.17284	2.674018	351|558|891|1080|7298	APP|AXL|CCNB1|CFTR|TYMS	-2.44373	23	0	0	-9.78876	32.19021
-3.85272	GO:0034502	protein localization to chromosome	19_GO:0051179 localization	-3.85272	15.28151	7.328948	91	4	4.938272	2.407398	983|2099|5347|9212	CDK1|ESR1|PLK1|AURKB	-2.43872	34	0	0	-7.56608	35.65685
-3.85272	GO:0051983	regulation of chromosome segregation	19_GO:0050789 regulation of biological process	-3.85272	15.28151	7.328948	91	4	4.938272	2.407398	891|1457|5347|9212	CCNB1|CSNK2A1|PLK1|AURKB	-2.43872	34	0	0	-7.56608	35.65685
-3.85272	GO:0046849	bone remodeling	19_GO:0032501 multicellular organismal process	-3.85272	15.28151	7.328948	91	4	4.938272	2.407398	760|1956|6714|6850	CA2|EGFR|SRC|SYK	-2.43872	36	0	0	-7.34456	54.89279
-3.85259	GO:0001893	maternal placenta development	19_GO:0051704 multi-organism process	-3.85259	29.79894	9.156397	35	3	3.703704	2.098362	207|5467|5743	AKT1|PPARD|PTGS2	-2.43872	19	0	0	-10.8808	39.73192
-3.85259	GO:0033280	response to vitamin D	19_GO:0050896 response to stimulus	-3.85259	29.79894	9.156	35	3	3.703704	2.098362	5292|5743|7299	PIM1|PTGS2|TYR	-2.43872	30	0	0	-8.76561	29.79894

<table>
<tr><th></th><th></th><th></th><th></th><th></th><th></th><th></th><th></th><th></th><th></th><th></th><th></th><th></th><th></th><th></th><th></th><th></th><th></th><th></th></tr>
<tr><td></td><td></td><td></td><td></td><td></td><td></td><td>397</td><td></td><td></td><td></td><td></td><td></td><td></td><td></td><td></td><td></td><td></td><td></td><td></td></tr>
<tr><td>–3.85259</td><td>GO:0033146</td><td>regulation of intracellular estrogen receptor signaling pathway</td><td>19_GO:0023052 signaling</td><td>–3.85259</td><td>29.79894</td><td>9.156397</td><td>35</td><td>3</td><td>3.703704</td><td>2.098362</td><td>142|367|6714</td><td>PARP1|AR|SRC</td><td>–2.43872</td><td>40</td><td>0</td><td>0</td><td>–7.12867</td><td>115.8848</td></tr>
<tr><td>–3.83831</td><td>GO:0051099</td><td>positive regulation of binding</td><td>19_GO:0065007 biological regulation</td><td>–3.83831</td><td>10.04781</td><td>6.41134</td><td>173</td><td>5</td><td>6.17284</td><td>2.674018</td><td>142|351|2932|4233|4318</td><td>PARP1|APP|GSK3B|MET|MMP9</td><td>–2.42658</td><td>21</td><td>0</td><td>0</td><td>–9.98291</td><td>27.26701</td></tr>
<tr><td>–3.83831</td><td>GO:0001678</td><td>cellular glucose homeostasis</td><td>19_GO:0065007 biological regulation</td><td>–3.83831</td><td>10.04781</td><td>6.41134</td><td>173</td><td>5</td><td>6.17284</td><td>2.674018</td><td>1080|3156|3480|5295|5467</td><td>CFTR|HMGCR|IGF1R|PIK3R1|PPARD</td><td>–2.42658</td><td>28</td><td>0</td><td>0</td><td>–8.92043</td><td>22.1907</td></tr>
<tr><td>–3.83831</td><td>GO:0010469</td><td>regulation of signaling receptor activity</td><td>19_GO:0023052 signaling</td><td>–3.83831</td><td>10.04781</td><td>6.41134</td><td>173</td><td>5</td><td>6.17284</td><td>2.674018</td><td>134|351|1612|2100|6714</td><td>ADORA1|APP|DAPK1|ESR2|SRC</td><td>–2.42658</td><td>68</td><td>0</td><td>0</td><td>–3.8713</td><td>18.96296</td></tr>
<tr><td>–3.81622</td><td>GO:0019217</td><td>regulation of fatty acid metabolic process</td><td>19_GO:0050789 regulation of biological process</td><td>–3.81622</td><td>14.95287</td><td>7.238815</td><td>93</td><td>4</td><td>4.938272</td><td>2.407398</td><td>207|5467|5743|10062</td><td>AKT1|PPARD|PTGS2|NR1H3</td><td>–2.40681</td><td>19</td><td>0</td><td>0</td><td>–10.8808</td><td>39.73192</td></tr>
<tr><td>–3.81622</td><td>GO:0106027</td><td>neuron projection organization</td><td>19_GO:0009987 cellular process</td><td>–3.81622</td><td>14.95287</td><td>7.238815</td><td>93</td><td>4</td><td>4.938272</td><td>2.407398</td><td>351|2932|3480|3643</td><td>APP|GSK3B|IGF1R|INSR</td><td>–2.40681</td><td>45</td><td>0</td><td>0</td><td>–6.56867</td><td>52.14815</td></tr>
</table>

-3.81571	GO:0070873	regulation of glycogen metabolic process	19_GO:0050789 regulation of biological process	-3.81571	28.97119	9.019532	36	3	3.703704	2.098362	207|2932|3643	AKT1|GSK3B|INSR	-2.40681	43	0	0	-6.76848	45.34622
-3.81571	GO:0002446	neutrophil mediated immunity	19_GO:0002376 immune system process	-3.81571	28.97119	9.019532	36	3	3.703704	2.098362	383|2147|6850	ARG1|F2|SYK	-2.40681	56	0	0	-5.89598	130.3704
-3.81496	GO:0022898	regulation of transmembrane transporter activity	19_GO:0051179 localization	-3.81496	7.503331	5.852704	278	6	7.407407	2.909904	351|1080|1612|4318|5243|5292	APP|CFTR|DAPK1|MMP9|ABCB1|PIM1	-2.40677	27	0	0	-8.92369	24.25495
-3.79828	GO:0030316	osteoclast differentiation	19_GO:0002376 immune system process	-3.79828	14.7938	7.194787	94	4	4.938272	2.407398	760|2101|5295|6714	CA2|ESRRA|PIK3R1|SRC	-2.39201	37	0	0	-7.31428	16.2963
-3.79828	GO:1903035	negative regulation of response to wounding	19_GO:0048519 negative regulation of biological process	-3.79828	14.7938	7.194787	94	4	4.938272	2.407398	239|240|2147|3156	ALOX12|ALOX5|F2|HMGCR	-2.39201	59	0	0	-5.59989	17.82843
-3.79808	GO:0021700	developmental maturation	19_GO:0032502 developmental process	-3.79808	7.449735	5.825522	280	6	7.407407	2.909904	351|558|891|1080|4313|7298	APP|AXL|CCNB1|CFTR|MMP2|TYMS	-2.39201	23	0	0	-9.78876	32.19021

-3.79198	GO:0071453	cellular response to oxygen levels	19_GO:0050896 response to stimulus	-3.79198	9.820744	6.322735	177	5	6.17284	2.674018	207|891|5467|5743|6714	AKT1|CCNB1|PPARD|PTGS2|SRC	-2.38661	19	0	0	-10.8808	39.73192
-3.78058	GO:0006109	regulation of carbohydrate metabolic process	19_GO:0050789 regulation of biological process	-3.78058	9.765571	6.301024	178	5	6.17284	2.674018	207|351|2932|3643|6714	AKT1|APP|GSK3B|INSR|SRC	-2.37801	43	0	0	-6.76848	45.34622
-3.78054	GO:0007589	body fluid secretion	19_GO:0051179 localization	-3.78054	14.63808	7.151427	95	4	4.938272	2.407398	134|1956|7498|10062	ADORA1|EGFR|XDH|NR1H3	-2.37801	61	0	0	-5.47166	27.44639
-3.77988	GO:0032885	regulation of polysaccharide biosynthetic process	19_GO:0050789 regulation of biological process	-3.77988	28.18819	8.888134	37	3	3.703704	2.098362	207|2932|3643	AKT1|GSK3B|INSR	-2.37801	43	0	0	-6.76848	45.34622
-3.77988	GO:0032570	response to progesterone	19_GO:0050896 response to stimulus	-3.77988	28.18819	8.888134	37	3	3.703704	2.098362	6714|7298|10062	SRC|TYMS|NR1H3	-2.37801	69	1	1	-3.77988	28.18819
-3.77988	GO:0045730	respiratory burst	19_GO:0008152 metabolic process	-3.77988	28.18819	8.888134	37	3	3.703704	2.098362	3643|4353|5294	INSR|MPO|PIK3CG	-2.37801	70	1	1	-3.77988	28.18819

-3.76925	GO:1901991	negative regulation of mitotic cell cycle phase transition	19_GO:0048519 negative regulation of biological process	-3.76925	9.711015	6.279484	179	5	6.17284	2.674018	891|983|1017|5347|9212	CCNB1|CDK1|CDK2|PLK1|AURKB	-2.36876	34	0	0	-7.56608	35.65685
-3.76925	GO:0009266	response to temperature stimulus	19_GO:0050896 response to stimulus	-3.76925	9.711015	6.279484	179	5	6.17284	2.674018	134|207|2932|4137|5743	ADORA1|AKT1|GSK3B|MAPT|PTGS2	-2.36876	51	0	0	-6.31611	22.67311
-3.76716	GO:0000904	cell morphogenesis involved in differentiation	19_GO:0032502 developmental process	-3.76716	4.508485	5.026457	694	9	11.11111	3.491885	351|367|558|2932|4137|4233|5295|5747|6714	APP|AR|AXL|GSK3B|MAPT|MET|PIK3R1|PTK2|SRC	-2.36737	51	0	0	-6.31611	22.67311
-3.76474	GO:0022409	positive regulation of cell-cell adhesion	19_GO:0022610 biological adhesion	-3.76474	7.34481	5.771954	284	6	7.407407	2.909904	207|240|246|3558|6714|6850	AKT1|ALOX5|ALOX15|IL2|SRC|SYK	-2.36564	25	0	0	-9.48567	22.67311
-3.7625	GO:0034330	cell junction organization	19_GO:0009987 cellular process	-3.7625	4.501998	5.020869	695	9	11.11111	3.491885	43|351|3480|3643|3791|4137|5295|5747|6714	ACHE|APP|IGF1R|INSR|KDR|MAPT|PIK3R1|PTK2|SRC	-2.36409	45	0	0	-6.56867	52.14815
-3.74566	GO:2001237	negative regulation of extrinsic apoptotic signaling pathway	19_GO:0048519 negative regulation of biological process	-3.74566	14.33626	7.066644	97	4	4.938272	2.407398	207|367|2932|6714	AKT1|AR|GSK3B|SRC	-2.35215	43	0	0	-6.76848	45.34622

-3.74566	GO:1901216	positive regulation of neuron death	19_GO:0048518 positive regulation of biological process	-3.74566	14.33626	7.0666644	97	4	4.938272	2.407398	134|142|2932|4137	ADORA1|PARP1|GSK3B|MAPT	-2.35215	51	0	0	-6.31611	22.67311
-3.74507	GO:0007094	mitotic spindle assembly checkpoint signaling	19_GO:0048519 negative regulation of biological process	-3.74507	27.44639	8.7618843	38	3	3.703704	2.098362	891|5347|9212	CCNB1|PLK1|AURKB	-2.35215	34	0	0	-7.56608	35.65685
-3.74507	GO:0071173	spindle assembly checkpoint signaling	19_GO:0048519 negative regulation of biological process	-3.74507	27.44639	8.7618843	38	3	3.703704	2.098362	891|5347|9212	CCNB1|PLK1|AURKB	-2.35215	34	0	0	-7.56608	35.65685
-3.74507	GO:0071174	mitotic spindle checkpoint signaling	19_GO:0048519 negative regulation of biological process	-3.74507	27.44639	8.7618843	38	3	3.703704	2.098362	891|5347|9212	CCNB1|PLK1|AURKB	-2.35215	34	0	0	-7.56608	35.65685
-3.74507	GO:0097242	amyloid-beta clearance	19_GO:0032501 multicellular organismal process	-3.74507	27.44639	8.7618843	38	3	3.703704	2.098362	3156|3480|3643	HMGCR|IGF1R|INSR	-2.35215	45	0	0	-6.56867	52.14815
-3.74507	GO:0071392	cellular response to estradiol stimulus	19_GO:0050896 response to stimulus	-3.74507	27.44639	8.7618843	38	3	3.703704	2.098362	1956|2099|2100	EGFR|ESR1|ESR2	-2.35215	61	0	0	-5.47166	27.44639

-3.74507	GO:0008210	estrogen metabolic process	19_GO:0008152 metabolic process	-3.74507	27.44639	8.761843	38	3	3.703704	2.098362	1545|1588|3292	CYP1B1|CYP19A1|HSD17B1	-2.35215	67	0	0	-4.05548	34.76543
-3.73717	GO:0090407	organophosphate biosynthetic process	19_GO:0008152 metabolic process	-3.73717	5.066001	5.167741	549	8	9.876543	3.314965	43|142|246|4843|5294|5295|5467|7298	ACHE|PARP1|ALOX15|NOS2|PIK3CG|PIK3R1|PPARD|TYMS	-2.34493	71	1	1	-3.73717	6.897903
-3.72851	GO:0002532	production of molecular mediator involved in inflammatory response	19_GO:0032501 multicellular organismal process	-3.72851	14.18997	7.025187	98	4	4.938272	2.407398	240|2147|4843|6850	ALOX5|F2|NOS2|SYK	-2.33695	59	0	0	-5.59989	17.82843
-3.72461	GO:0061138	morphogenesis of a branching epithelium	19_GO:0032502 developmental process	-3.72461	9.498752	6.194987	183	5	6.17284	2.674018	367|2099|3791|4233|6714	AR|ESR1|KDR|MET|SRC	-2.33373	60	0	0	-5.48956	45.34622
-3.7157	GO:0046651	lymphocyte proliferation	19_GO:0002376 immune system process	-3.7157	7.192848	5.693525	290	6	7.407407	2.909904	196|383|2322|3558|5294|6850	AHR|ARG1|FLT3|IL2|PIK3CG|SYK	-2.3255	56	0	0	-5.89598	130.3704
-3.7112	GO:0045923	positive regulation of fatty acid metabolic process	19_GO:0048518 positive regulation of biological process	-3.7112	26.74264	8.640333	39	3	3.703704	2.098362	5467|5743|10062	PPARD|PTGS2|NR1H3	-2.3237	19	0	0	-10.8808	39.73192
-3.711	GO:0071548	response to dexamethasone	19_GO:0050896 response to	-3.711	26.74264	8.6	39	3	3.703704	2.098362	383|1956|2908	ARG1|EGFR|NR3C1	-2.3237	29	0	0	-8.8	35.96424

2			stimulus	2		4033											7872	
-3.7112	GO:0031577	spindle checkpoint signaling	19_GO:0048519 negative regulation of biological process	-3.7112	26.74264	8.64033	39	3	3.703704	2.098362	891|5347|9212	CCNB1|PLK1|AURKB	-2.3237	34	0	0	-7.56608	35.65685
-3.7112	GO:0046326	positive regulation of glucose import	19_GO:0048518 positive regulation of biological process	-3.7112	26.74264	8.64033	39	3	3.703704	2.098362	207|3643|5295	AKT1|INSR|PIK3R1	-2.3237	54	0	0	-6.06931	37.58425
-3.70268	GO:0001936	regulation of endothelial cell proliferation	19_GO:0050789 regulation of biological process	-3.70268	9.396063	6.153707	185	5	6.17284	2.674018	207|240|383|3791|7498	AKT1|ALOX5|ARG1|KDR|XDH	-2.31646	39	0	0	-7.16624	9.980507
-3.70261	GO:0072521	purine-containing compound metabolic process	19_GO:0008152 metabolic process	-3.70261	5.849953	5.352544	416	7	8.641975	3.122032	142|351|3156|3643|4843|7276|7498	PARP1|APP|HMGCR|INSR|NOS2|TTR|XDH	-2.31646	45	0	0	-6.56867	52.14815
-3.69476	GO:0060079	excitatory postsynaptic potential	19_GO:0023052 signaling	-3.69476	13.90617	6.944068	100	4	4.938272	2.407398	134|207|351|2932	ADORA1|AKT1|APP|GSK3B	-2.30927	51	0	0	-6.31611	22.67311
-3.69161	GO:0032943	mononuclear cell proliferation	19_GO:0009987 cellular process	-3.69161	7.119201	5.655514	293	6	7.407407	2.909904	196|383|2322|3558|5294|6850	AHR|ARG1|FLT3|IL2|PIK3CG|SYK	-2.30679	56	0	0	-5.89598	130.3704

						5												
-3.67825	GO:0150077	regulation of neuroinflammatory response	19_GO:0050896 response to stimulus	-3.67825	26.07407	8.523296	40	3	3.703704	2.098362	4314|4318|5743	MMP3|MMP9|PTGS2	-2.29866	6	0	0	-14.9128	124.1623
-3.67825	GO:0010543	regulation of platelet activation	19_GO:0032501 multicellular organismal process	-3.67825	26.07407	8.523296	40	3	3.703704	2.098362	239|2147|6850	ALOX12|F2|SYK	-2.29866	9	0	0	-13.6942	26.07407
-3.67825	GO:0045429	positive regulation of nitric oxide biosynthetic process	19_GO:0048518 positive regulation of biological process	-3.67825	26.07407	8.523296	40	3	3.703704	2.098362	207|3643|5743	AKT1|INSR|PTGS2	-2.29866	19	0	0	-10.8808	39.73192
-3.67825	GO:0045841	negative regulation of mitotic metaphase/anaphase transition	19_GO:0048519 negative regulation of biological process	-3.67825	26.07407	8.523296	40	3	3.703704	2.098362	891|5347|9212	CCNB1|PLK1|AURKB	-2.29866	34	0	0	-7.56608	35.65685
-3.67825	GO:0032941	secretion by tissue	19_GO:0051179 localization	-3.67825	26.07407	8.523296	40	3	3.703704	2.098362	134|1956|10062	ADORA1|EGFR|NR1H3	-2.29866	61	0	0	-5.47166	27.44639
-3.67825	GO:0006730	one-carbon metabolic process	19_GO:0008152 metabolic process	-3.67825	26.07407	8.52329	40	3	3.703704	2.098362	760|768|7298	CA2|CA9|TYMS	-2.29866	72	1	1	-3.67825	26.07407

						6												
-3.67825	GO:0009112	nucleobase metabolic process	19_GO:0008152 metabolic process	-3.67825	26.07407	8.523296	40	3	3.703704	2.098362	7276|7298|7498	TTR|TYMS|XDH	-2.29866	73	1	1	-3.67825	26.07407
-3.67815	GO:0150115	cell-substrate junction organization	19_GO:0009987 cellular process	-3.67815	13.76849	6.904375	101	4	4.938272	2.407398	3791|5295|5747|6714	KDR|PIK3R1|PTK2|SRC	-2.29866	41	0	0	-7.04885	38.62826
-3.67101	GO:0072594	establishment of protein localization to organelle	19_GO:0051179 localization	-3.67101	5.780476	5.307953	421	7	8.641975	3.122032	207|983|4137|4321|5295|5743|6850	AKT1|CDK1|MAPT|MMP12|PIK3R1|PTGS2|SYK	-2.29217	16	0	0	-11.5558	31.96821
-3.66173	GO:0002444	myeloid leukocyte mediated immunity	19_GO:0002376 immune system process	-3.66173	13.6335	6.865241	102	4	4.938272	2.407398	383|2147|5294|6850	ARG1|F2|PIK3CG|SYK	-2.28355	56	0	0	-5.89598	130.3704
-3.64869	GO:0007268	chemical synaptic transmission	19_GO:0023052 signaling	-3.64869	4.345679	4.884485	720	9	11.11111	3.491885	43|134|207|351|1956|2932|4137|5743|6714	ACHE|ADORA1|AKT1|APP|EGFR|GSK3B|MAPT|PTGS2|SRC	-2.27256	51	0	0	-6.31611	22.67311
-3.64869	GO:0098916	anterograde trans-synaptic signaling	19_GO:0023052 signaling	-3.64869	4.345679	4.88448	720	9	11.11111	3.491885	43|134|207|351|1956|2932|4137|5743|6714	ACHE|ADORA1|AKT1|APP|EGFR|GSK3B|MAPT|PTGS2|SRC	-2.27256	51	0	0	-6.31611	22.67311

						5												
-3.64614	GO:0042307	positive regulation of protein import into nucleus	19_GO:0048518 positive regulation of biological process	-3.64614	25.43812	8.410467	41	3	3.703704	2.098362	983|5295|5743	CDK1|PIK3R1|PTGS2	-2.27256	16	0	0	-11.5558	31.96821
-3.64614	GO:1904407	positive regulation of nitric oxide metabolic process	19_GO:0048518 positive regulation of biological process	-3.64614	25.43812	8.410467	41	3	3.703704	2.098362	207|3643|5743	AKT1|INSR|PTGS2	-2.27256	19	0	0	-10.8808	39.73192
-3.64614	GO:0055023	positive regulation of cardiac muscle tissue growth	19_GO:0040007 growth	-3.64614	25.43812	8.410467	41	3	3.703704	2.098362	891|983|5292	CCNB1|CDK1|PIM1	-2.27256	35	0	0	-7.56218	38.62826
-3.64614	GO:0001953	negative regulation of cell-matrix adhesion	19_GO:0022610 biological adhesion	-3.64614	25.43812	8.410467	41	3	3.703704	2.098362	4321|5295|6714	MMP12|PIK3R1|SRC	-2.27256	41	0	0	-7.04885	38.62826
-3.64614	GO:0010907	positive regulation of glucose metabolic process	19_GO:0048518 positive regulation of biological process	-3.64614	25.43812	8.410467	41	3	3.703704	2.098362	207|3643|6714	AKT1|INSR|SRC	-2.27256	43	0	0	-6.76848	45.34622
-3.64547	GO:0019395	fatty acid oxidation	19_GO:0008152 metabolic process	-3.64547	13.50114	6.82665	103	4	4.938272	2.407398	207|239|246|5467	AKT1|ALOX12|ALOX15|PPARD	-2.27253	11	0	0	-13.1836	260.7407

						4												
-3.64424	GO:0007276	gamete generation	19_GO:0051704 multi-organism process	-3.64424	4.339652	4.879159	721	9	11.11111	3.491885	207|367|558|891|1080|5347|5743|6714|7153	AKT1|AR|AXL|CCNB1|CFTR|PLK1|PTGS2|SRC|TOP2A	-2.27196	58	0	0	-5.66793	43.45679
-3.62938	GO:1903076	regulation of protein localization to plasma membrane	19_GO:0051179 localization	-3.62938	13.37132	6.788599	104	4	4.938272	2.407398	207|367|1956|5295	AKT1|AR|EGFR|PIK3R1	-2.25775	54	0	0	-6.06931	37.58425
-3.62696	GO:0055086	nucleobase-containing small molecule metabolic process	19_GO:0008152 metabolic process	-3.62696	4.879359	5.02553	570	8	9.876543	3.314965	142|351|3156|3643|4843|7276|7298|7498	PARP1|APP|HMGCR|INSR|NOS2|TTR|TYMS|XDH	-2.25598	45	0	0	-6.56867	52.14815
-3.61486	GO:1902100	negative regulation of metaphase/anaphase transition of cell cycle	19_GO:0048519 negative regulation of biological process	-3.61486	24.83245	8.301593	42	3	3.703704	2.098362	891|5347|9212	CCNB1|PLK1|AURKB	-2.24618	34	0	0	-7.56608	35.65685
-3.61486	GO:0055090	acylglycerol homeostasis	19_GO:0065007 biological regulation	-3.61486	24.83245	8.301593	42	3	3.703704	2.098362	134|3156|10062	ADORA1|HMGCR|NR1H3	-2.24618	74	1	1	-3.61486	24.83245
-3.61486	GO:0070328	triglyceride homeostasis	19_GO:0065007 biological regulation	-3.61486	24.83245	8.301593	42	3	3.703704	2.098362	134|3156|10062	ADORA1|HMGCR|NR1H3	-2.24618	74	0	0	-3.61486	24.83245

-3.61346	GO:0071887	leukocyte apoptotic process	19_GO:0009987 cellular process	-3.61346	13.24397	6.751064	105	4	4.938272	2.407398	207|558|3558|9212	AKT1|AXL|IL2|AURKB	-2.24618	25	0	0	-9.48567	22.67311
-3.61346	GO:0042116	macrophage activation	19_GO:0002376 immune system process	-3.61346	13.24397	6.751064	105	4	4.938272	2.407398	351|4137|6850|10062	APP|MAPT|SYK|NR1H3	-2.24618	44	0	0	-6.70994	13.24397
-3.6133	GO:0099537	trans-synaptic signaling	19_GO:0023052 signaling	-3.6133	4.297924	4.842147	728	9	11.11111	3.491885	43|134|207|351|1956|2932|4137|5743|6714	ACHE|ADORA1|AKT1|APP|EGFR|GSK3B|MAPT|PTGS2|SRC	-2.24618	51	0	0	-6.31611	22.67311
-3.5977	GO:0046822	regulation of nucleocytoplasmic transport	19_GO:0051179 localization	-3.5977	13.11903	6.714037	106	4	4.938272	2.407398	983|2932|5295|5743	CDK1|GSK3B|PIK3R1|PTGS2	-2.23315	16	0	0	-11.5558	31.96821
-3.5977	GO:0002793	positive regulation of peptide secretion	19_GO:0048518 positive regulation of biological process	-3.5977	13.11903	6.714037	106	4	4.938272	2.407398	134|1080|1956|5467	ADORA1|CFTR|EGFR|PPARD	-2.23315	28	0	0	-8.92043	22.1907
-3.5977	GO:0046620	regulation of organ growth	19_GO:0040007 growth	-3.5977	13.11903	6.714037	106	4	4.938272	2.407398	207|891|983|5292	AKT1|CCNB1|CDK1|PIM1	-2.23315	35	0	0	-7.56218	38.62826

-3.5977	GO:0099565	chemical synaptic transmission, postsynaptic	19_GO:0023052 signaling	-3.5977	13.11903	6.7140337	106	4	4.938272	2.407398	134|207|351|2932	ADORA1|AKT1|APP|GSK3B	-2.23315	51	0	0	-6.31611	22.67311
-3.59686	GO:0030073	insulin secretion	19_GO:0051179 localization	-3.59686	8.914213	5.956319	195	5	6.17284	2.674018	240|1080|3156|4843|5467	ALOX5|CFTR|HMGCR|NOS2|PPARD	-2.23295	28	0	0	-8.92043	22.1907
-3.58661	GO:0022408	negative regulation of cell-cell adhesion	19_GO:0022610 biological adhesion	-3.58661	8.868733	5.937361	196	5	6.17284	2.674018	207|239|383|3558|5747	AKT1|ALOX12|ARG1|IL2|PTK2	-2.2274	25	0	0	-9.48567	22.67311
-3.58661	GO:0006006	glucose metabolic process	19_GO:0008152 metabolic process	-3.58661	8.868733	5.937361	196	5	6.17284	2.674018	207|2932|3643|5467|6714	AKT1|GSK3B|INSR|PPARD|SRC	-2.2274	43	0	0	-6.76848	45.34622
-3.58435	GO:1904591	positive regulation of protein import	19_GO:0048518 positive regulation of biological process	-3.58435	24.25495	8.196444	43	3	3.703704	2.098362	983|5295|5743	CDK1|PIK3R1|PTGS2	-2.2274	16	0	0	-11.5558	31.96821
-3.58435	GO:1903793	positive regulation of anion transport	19_GO:0048518 positive regulation of biological process	-3.58435	24.25495	8.196444	43	3	3.703704	2.098362	1080|5243|9536	CFTR|ABCB1|PTGES	-2.2274	27	0	0	-8.92369	24.25495

-3.58435	GO:0140353	lipid export from cell	19_GO:0051179 localization	-3.58435	24.25495	8.196444	43	3	3.703704	2.098362	1588|4843|9536	CYP19A1|NOS2|PTGES	-2.2274	33	0	0	-8.21676	32.59259
-3.58435	GO:0033046	negative regulation of sister chromatid segregation	19_GO:0048519 negative regulation of biological process	-3.58435	24.25495	8.196444	43	3	3.703704	2.098362	891|5347|9212	CCNB1|PLK1|AURKB	-2.2274	34	0	0	-7.56608	35.65685
-3.58435	GO:0033048	negative regulation of mitotic sister chromatid segregation	19_GO:0048519 negative regulation of biological process	-3.58435	24.25495	8.196444	43	3	3.703704	2.098362	891|5347|9212	CCNB1|PLK1|AURKB	-2.2274	34	0	0	-7.56608	35.65685
-3.58435	GO:2000816	negative regulation of mitotic sister chromatid separation	19_GO:0048519 negative regulation of biological process	-3.58435	24.25495	8.196444	43	3	3.703704	2.098362	891|5347|9212	CCNB1|PLK1|AURKB	-2.2274	34	0	0	-7.56608	35.65685
-3.58435	GO:0045981	positive regulation of nucleotide metabolic process	19_GO:0048518 positive regulation of biological process	-3.58435	24.25495	8.196444	43	3	3.703704	2.098362	351|3643|4843	APP|INSR|NOS2	-2.2274	45	0	0	-6.56867	52.14815
-3.58435	GO:1900544	positive regulation of purine nucleotide metabolic process	19_GO:0048518 positive regulation of biological process	-3.58435	24.25495	8.196444	43	3	3.703704	2.098362	351|3643|4843	APP|INSR|NOS2	-2.2274	45	0	0	-6.56867	52.14815

-3.58435	GO:0120178	steroid hormone biosynthetic process	19_GO:0008152 metabolic process	-3.58435	24.25495	8.1964444	43	3	3.703704	2.098362	1586|1588|3292	CYP17A1|CYP19A1|HSD17B1	-2.2274	67	0	0	-4.05548	34.76543
-3.57641	GO:0048167	regulation of synaptic plasticity	19_GO:0023052 signaling	-3.57641	8.823714	5.9185399	197	5	6.17284	2.674018	134|351|2932|4137|5743	ADORA1|APP|GSK3B|MAPT|PTGS2	-2.22071	51	0	0	-6.31611	22.67311
-3.57641	GO:0001763	morphogenesis of a branching structure	19_GO:0032502 developmental process	-3.57641	8.823714	5.9185399	197	5	6.17284	2.674018	367|2099|3791|4233|6714	AR|ESR1|KDR|MET|SRC	-2.22071	60	0	0	-5.48956	45.34622
-3.57116	GO:0007610	behavior	19_GO:0007610 behavior	-3.57116	4.786979	4.9539954	581	8	9.876543	3.314965	134|238|351|1956|3156|3643|4137|5743	ADORA1|ALK|APP|EGFR|HMGCR|INSR|MAPT|PTGS2	-2.21608	45	0	0	-6.56867	52.14815
-3.56665	GO:0033138	positive regulation of peptidyl-serine phosphorylation	19_GO:0048518 positive regulation of biological process	-3.56665	12.87609	6.6414461	108	4	4.938272	2.407398	207|351|1956|5743	AKT1|APP|EGFR|PTGS2	-2.21407	19	0	0	-10.8808	39.73192
-3.56665	GO:0034446	substrate adhesion-dependent cell spreading	19_GO:0022610 biological adhesion	-3.56665	12.87609	6.6414461	108	4	4.938272	2.407398	558|5295|5747|6714	AXL|PIK3R1|PTK2|SRC	-2.21407	41	0	0	-7.04885	38.62826

-3.56665	GO:0002832	negative regulation of response to biotic stimulus	19_GO:0048519 negative regulation of biological process	-3.56665	12.87609	6.6641461	108	4	4.938272	2.407398	196|383|4321|10062	AHR|ARG1|MMP12|NR1H3	-2.21407	55	0	0	-5.98599	20.05698
-3.56665	GO:0035821	modulation of process of other organism	19_GO:0044419 biological process involved in interspecies interaction between organisms	-3.56665	12.87609	6.6641461	108	4	4.938272	2.407398	383|3643|4843|6850	ARG1|INSR|NOS2|SYK	-2.21407	56	0	0	-5.89598	130.3704
-3.55458	GO:0150076	neuroinflammatory response	19_GO:0050896 response to stimulus	-3.55458	23.7037	8.0948009	44	3	3.703704	2.098362	4314|4318|5743	MMP3|MMP9|PTGS2	-2.20509	6	0	0	-14.9128	124.1623
-3.55458	GO:0005978	glycogen biosynthetic process	19_GO:0008152 metabolic process	-3.55458	23.7037	8.0948009	44	3	3.703704	2.098362	207|2932|3643	AKT1|GSK3B|INSR	-2.20509	43	0	0	-6.76848	45.34622
-3.55458	GO:0009250	glucan biosynthetic process	19_GO:0008152 metabolic process	-3.55458	23.7037	8.0948009	44	3	3.703704	2.098362	207|2932|3643	AKT1|GSK3B|INSR	-2.20509	43	0	0	-6.76848	45.34622
-3.55458	GO:0032881	regulation of polysaccharide metabolic process	19_GO:0050789 regulation of biological process	-3.55458	23.7037	8.0948009	44	3	3.703704	2.098362	207|2932|3643	AKT1|GSK3B|INSR	-2.20509	43	0	0	-6.76848	45.34622

-3.55458	GO:1903573	negative regulation of response to endoplasmic reticulum stress	19_GO:0048519 negative regulation of biological process	-3.55458	23.7037	8.0948809	44	3	3.703704	2.098362	240|5770|10062	ALOX5|PTPN1|NR1H3	-2.20509	62	0	0	-5.40866	45.34622
-3.55136	GO:2000060	positive regulation of ubiquitin-dependent protein catabolic process	19_GO:0048518 positive regulation of biological process	-3.55136	12.75796	6.605888	109	4	4.938272	2.407398	207|2932|5347|5747	AKT1|GSK3B|PLK1|PTK2	-2.20249	43	0	0	-6.76848	45.34622
-3.54617	GO:0001935	endothelial cell proliferation	19_GO:0009987 cellular process	-3.54617	8.691358	5.862863	200	5	6.17284	2.674018	207|240|383|3791|7498	AKT1|ALOX5|ARG1|KDR|XDH	-2.19792	39	0	0	-7.16624	9.980507
-3.5429	GO:0002443	leukocyte mediated immunity	19_GO:0002376 immune system process	-3.5429	5.505838	5.128213	442	7	8.641975	3.122032	196|383|2147|3558|4843|5294|6850	AHR|ARG1|F2|IL2|NOS2|PIK3CG|SYK	-2.19526	56	0	0	-5.89598	130.3704
-3.53621	GO:0007088	regulation of mitotic nuclear division	19_GO:0050789 regulation of biological process	-3.53621	12.64198	6.570779	110	4	4.938272	2.407398	891|3643|5347|9212	CCNB1|INSR|PLK1|AURKB	-2.18919	34	0	0	-7.56608	35.65685
-3.53045	GO:0060249	anatomical structure homeostasis	19_GO:0065007 biological regulation	-3.53045	6.643076	5.400819	314	6	7.407407	2.909904	134|760|1956|5743|6714|6850	ADORA1|CA2|EGFR|PTGS2|SRC|SYK	-2.18404	36	0	0	-7.34456	54.89279

-3.52552	GO:0032309	icosanoid secretion	19_GO:0051179 localization	-3.52552	23.17695	7.996491	45	3	3.703704	2.098362	4843|6850|9536	NOS2|SYK|PTGES	-2.18276	33	0	0	-8.21676	32.59259
-3.52552	GO:0051985	negative regulation of chromosome segregation	19_GO:0048519 negative regulation of biological process	-3.52552	23.17695	7.996491	45	3	3.703704	2.098362	891|5347|9212	CCNB1|PLK1|AURKB	-2.18276	34	0	0	-7.56608	35.65685
-3.52552	GO:1905819	negative regulation of chromosome separation	19_GO:0048519 negative regulation of biological process	-3.52552	23.17695	7.996491	45	3	3.703704	2.098362	891|5347|9212	CCNB1|PLK1|AURKB	-2.18276	34	0	0	-7.56608	35.65685
-3.52552	GO:0060421	positive regulation of heart growth	19_GO:0040007 growth	-3.52552	23.17695	7.996491	45	3	3.703704	2.098362	891|983|5292	CCNB1|CDK1|PIM1	-2.18276	35	0	0	-7.56218	38.62826
-3.52552	GO:0048806	genitalia development	19_GO:0022414 reproductive process	-3.52552	23.17695	7.996491	45	3	3.703704	2.098362	367|558|2099	AR|AXL|ESR1	-2.18276	60	0	0	-5.48956	45.34622
-3.52552	GO:0008631	intrinsic apoptotic signaling pathway in response to oxidative stress	19_GO:0023052 signaling	-3.52552	23.17695	7.996491	45	3	3.703704	2.098362	142|207|1545	PARP1|AKT1|CYP1B1	-2.18276	63	0	0	-5.22413	23.17695

-3.52122	GO:0009408	response to heat	19_GO:0050896 response to stimulus	-3.52122	12.52808	6.536123	111	4	4.938272	2.407398	207|2932|4137|5743	AKT1|GSK3B|MAPT|PTGS2	-2.17968	51	0	0	-6.31611	22.67311
-3.52122	GO:1904659	glucose transmembrane transport	19_GO:0051179 localization	-3.52122	12.52808	6.536123	111	4	4.938272	2.407398	207|3643|5295|5467	AKT1|INSR|PIK3R1|PPARD	-2.17968	54	0	0	-6.06931	37.58425
-3.50993	GO:0099536	synaptic signaling	19_GO:0023052 signaling	-3.50993	4.160757	4.718692	752	9	11.11111	3.491885	43|134|207|351|1956|2932|4137|5743|6714	ACHE|ADORA1|AKT1|APP|EGFR|GSK3B|MAPT|PTGS2|SRC	-2.169	51	0	0	-6.31611	22.67311
-3.50662	GO:0051701	biological process involved in interaction with host	19_GO:0044419 biological process involved in interspecies interaction between organisms	-3.50662	8.520939	5.790418	204	5	6.17284	2.674018	558|983|1956|3643|6714	AXL|CDK1|EGFR|INSR|SRC	-2.16629	57	0	0	-5.76668	22.87199
-3.50178	GO:0006874	cellular calcium ion homeostasis	19_GO:0065007 biological regulation	-3.50178	5.420001	5.070838	449	7	8.641975	3.122032	134|351|2099|2147|3558|3577|5294	ADORA1|APP|ESR1|F2|IL2|CXCR1|PIK3CG	-2.16263	38	0	0	-7.2588	41.71852
-3.50115	GO:0071496	cellular response to external stimulus	19_GO:0050896 response to stimulus	-3.50115	6.559515	5.355014	318	6	7.407407	2.909904	207|558|1956|5292|5743|8644	AKT1|AXL|EGFR|PIM1|PTGS2|AKR1C3	-2.16263	19	0	0	-10.8808	39.73192

-3.50115	GO:0015980	energy derivation by oxidation of organic compounds	19_GO:0008152 metabolic process	-3.50115	6.559515	5.3550014	318	6	7.407407	2.909904	207|891|983|2932|3643|4843	AKT1|CCNB1|CDK1|GSK3B|INSR|NOS2	-2.16263	35	0	0	-7.56218	38.62826
-3.49713	GO:1900087	positive regulation of G1/S transition of mitotic cell cycle	19_GO:0048518 positive regulation of biological process	-3.49713	22.67311	7.90131	46	3	3.703704	2.098362	207|328|1956	AKT1|APEX1|EGFR	-2.1622	10	0	0	-13.3451	41.71852
-3.49713	GO:2000107	negative regulation of leukocyte apoptotic process	19_GO:0048519 negative regulation of biological process	-3.49713	22.67311	7.90131	46	3	3.703704	2.098362	558|3558|9212	AXL|IL2|AURKB	-2.1622	25	0	0	-9.48567	22.67311
-3.49713	GO:0033047	regulation of mitotic sister chromatid segregation	19_GO:0050789 regulation of biological process	-3.49713	22.67311	7.90131	46	3	3.703704	2.098362	891|5347|9212	CCNB1|PLK1|AURKB	-2.1622	34	0	0	-7.56608	35.65685
-3.49713	GO:0031018	endocrine pancreas development	19_GO:0032502 developmental process	-3.49713	22.67311	7.90131	46	3	3.703704	2.098362	207|1021|2932	AKT1|CDK6|GSK3B	-2.1622	43	0	0	-6.76848	45.34622
-3.49713	GO:1900271	regulation of long-term synaptic potentiation	19_GO:0048518 positive regulation of biological process	-3.49713	22.67311	7.90131	46	3	3.703704	2.098362	134|351|2932	ADORA1|APP|GSK3B	-2.1622	51	0	0	-6.31611	22.67311
-3.49713	GO:0010828	positive regulation of glucose transmembrane transport	19_GO:0048518 positive regulation of biological process	-3.49713	22.67311	7.9013	46	3	3.703704	2.098362	207|3643|5295	AKT1|INSR|PIK3R1	-2.1622	54	0	0	-6.06931	37.58425

						1												
-3.49389	GO:1901214	regulation of neuron death	19_GO:0050789 regulation of biological process	-3.49389	6.538953	5.343686	319	6	7.407407	2.909904	134|142|207|558|2932|4137	ADORA1|PARP1|AKT1|AXL|GSK3B|MAPT	-2.15956	51	0	0	-6.31611	22.67311
-3.48666	GO:0010506	regulation of autophagy	19_GO:0050789 regulation of biological process	-3.48666	6.518519	5.332406	320	6	7.407407	2.909904	207|1612|2932|3791|4137|4233	AKT1|DAPK1|GSK3B|KDR|MAPT|MET	-2.15292	43	0	0	-6.76848	45.34622
-3.4771	GO:0031623	receptor internalization	19_GO:0051179 localization	-3.4771	12.1984	6.434772	114	4	4.938272	2.407398	43|3577|3643|6850	ACHE|CXCR1|INSR|SYK	-2.14514	44	0	0	-6.70994	13.24397
-3.4771	GO:0008645	hexose transmembrane transport	19_GO:0051179 localization	-3.4771	12.1984	6.434772	114	4	4.938272	2.407398	207|3643|5295|5467	AKT1|INSR|PIK3R1|PPARD	-2.14514	54	0	0	-6.06931	37.58425
-3.4771	GO:0046660	female sex differentiation	19_GO:0022414 reproductive process	-3.4771	12.1984	6.434772	114	4	4.938272	2.407398	558|1588|2099|6714	AXL|CYP19A1|ESR1|SRC	-2.14514	60	0	0	-5.48956	45.34622
-3.46938	GO:0042311	vasodilation	19_GO:0032501 multicellular organismal process	-3.46938	22.1907	7.8091	47	3	3.703704	2.098362	134|1956|5467	ADORA1|EGFR|PPARD	-2.13802	28	0	0	-8.92043	22.1907
-	GO:000	regulation of	19_GO:0002376	-	4.589	4.	60	8	9.87	3.314	196|207|383|558|	AHR|AKT1|ARG1|AXL|IL2|	-	25	0	0	-	22.67

3.44895	2694	leukocyte activation	immune system process	3.44895	496	7977752	6		6543	965	3558|6714|6850|10062	SRC|SYK|NR1H3	2.11819				9.48567	311
-3.44837	GO:0015749	monosaccharide transmembrane transport	19_GO:0051179 localization	-3.44837	11.98808	6.3692922	116	4	4.938272	2.407398	207|3643|5295|5467	AKT1|INSR|PIK3R1|PPARD	-2.11819	54	0	0	-6.06931	37.58425
-3.44225	GO:0045839	negative regulation of mitotic nuclear division	19_GO:0048519 negative regulation of biological process	-3.44225	21.7284	7.7197055	48	3	3.703704	2.098362	891|5347|9212	CCNB1|PLK1|AURKB	-2.11325	34	0	0	-7.56608	35.65685
-3.44225	GO:0048146	positive regulation of fibroblast proliferation	19_GO:0048518 positive regulation of biological process	-3.44225	21.7284	7.7197055	48	3	3.703704	2.098362	891|1021|2099	CCNB1|CDK6|ESR1	-2.11325	75	1	1	-3.44225	21.7284
-3.43301	GO:0055074	calcium ion homeostasis	19_GO:0065007 biological regulation	-3.43301	5.278916	4.9752222	461	7	8.641975	3.122032	134|351|2099|2147|3558|3577|5294	ADORA1|APP|ESR1|F2|IL2|CXCR1|PIK3CG	-2.1046	38	0	0	-7.2588	41.71852
-3.43004	GO:0030258	lipid modification	19_GO:0008152 metabolic process	-3.43004	8.199394	5.6513066	212	5	6.17284	2.674018	207|239|240|246|5467	AKT1|ALOX12|ALOX5|ALOX15|PPARD	-2.10221	11	0	0	-13.1836	260.7407
-	GO:003	carbohydrate	19_GO:0051179	-	11.78	6.	11	4	4.93	2.407	207|3643|5295|	AKT1|INSR|PIK3R1|PPARD	-	54	0	0	-	37.58

3.42017	4219	transmembrane transport	localization	3.42017	489	3054	8		8272	398	5467		2.09352				6.06931	425
-3.42017	GO:0071346	cellular response to interferon-gamma	19_GO:0044419 biological process involved in interspecies interaction between organisms	-3.42017	11.78489	6.3054	118	4	4.938272	2.407398	383|1612|4843|10062	ARG1|DAPK1|NOS2|NR1H3	-2.09352	56	0	0	-5.89598	130.3704
-3.41571	GO:0006953	acute-phase response	19_GO:0050896 response to stimulus	-3.41571	21.28496	7.632983	49	3	3.703704	2.098362	2147|5743|9536	F2|PTGS2|PTGES	-2.09139	15	0	0	-12.2065	35.47493
-3.41571	GO:0030225	macrophage differentiation	19_GO:0002376 immune system process	-3.41571	21.28496	7.632983	49	3	3.703704	2.098362	142|351|4318	PARP1|APP|MMP9	-2.09139	21	0	0	-9.98291	27.26701
-3.41571	GO:0038084	vascular endothelial growth factor signaling pathway	19_GO:0023052 signaling	-3.41571	21.28496	7.632983	49	3	3.703704	2.098362	2322|3791|7498	FLT3|KDR|XDH	-2.09139	66	0	0	-4.22583	21.28496
-3.41571	GO:0060562	epithelial tube morphogenesis	19_GO:0032502 developmental process	-3.41571	6.320988	5.222193	330	6	7.407407	2.909904	239|367|2099|3791|4233|6714	ALOX12|AR|ESR1|KDR|MET|SRC	-2.09139	60	0	0	-5.48956	45.34622
-3.4114	GO:0000082	G1/S transition of mitotic cell cycle	19_GO:0009987 cellular process	-3.4114	8.122765	5.61	214	5	6.17284	2.674018	207|328|1017|1021|1956	AKT1|APEX1|CDK2|CDK6|EGFR	-2.08766	10	0	0	-13.345	41.71852

						7666											1	
-3.40627	GO:0030282	bone mineralization	19_GO:0110148 biomineralization	-3.40627	11.68586	6.274029	119	4	4.938272	2.407398	240|246|4322|5743	ALOX5|ALOX15|MMP13|PTGS2	-2.08369	11	0	0	-13.1836	260.7407
-3.40627	GO:0010906	regulation of glucose metabolic process	19_GO:0050789 regulation of biological process	-3.40627	11.68586	6.274029	119	4	4.938272	2.407398	207|2932|3643|6714	AKT1|GSK3B|INSR|SRC	-2.08369	43	0	0	-6.76848	45.34622
-3.39249	GO:0071901	negative regulation of protein serine/threonine kinase activity	19_GO:0048519 negative regulation of biological process	-3.39249	11.58848	6.24303	120	4	4.938272	2.407398	207|3156|5347|5770	AKT1|HMGCR|PLK1|PTPN1	-2.07165	17	0	0	-11.2655	11.58848
-3.39249	GO:0002761	regulation of myeloid leukocyte differentiation	19_GO:0002376 immune system process	-3.39249	11.58848	6.24303	120	4	4.938272	2.407398	760|1021|2101|5295	CA2|CDK6|ESRRA|PIK3R1	-2.07165	37	0	0	-7.31428	16.2963
-3.39249	GO:0022612	gland morphogenesis	19_GO:0032502 developmental process	-3.39249	11.58848	6.24303	120	4	4.938272	2.407398	367|383|2099|6714	AR|ARG1|ESR1|SRC	-2.07165	60	0	0	-5.48956	45.34622
-3.38974	GO:1901570	fatty acid derivative biosynthetic process	19_GO:0008152 metabolic process	-3.38974	20.85926	7.54888	50	3	3.703704	2.098362	239|240|246	ALOX12|ALOX5|ALOX15	-2.07005	11	0	0	-13.1836	260.7407
-	GO:003	Fc receptor signaling	19_GO:0002376	-	20.85	7.	50	3	3.70	2.098	5747|6714|6850	PTK2|SRC|SYK	-	41	0	0	-	38.62

3.38974	8093	pathway	immune system process	3.38974	926	5488			3704	362			2.07005				7.04885	826
-3.37883	GO:0042177	negative regulation of protein catabolic process	19_GO:0048519 negative regulation of biological process	-3.37883	11.4927	6.212397	121	4	4.938272	2.407398	1457|1956|3156|4843	CSNK2A1|EGFR|HMGCR|NOS2	-2.05972	48	0	0	-6.42162	11.4927
-3.37432	GO:1903037	regulation of leukocyte cell-cell adhesion	19_GO:0022610 biological adhesion	-3.37432	6.208113	5.158228	336	6	7.407407	2.909904	207|240|383|3558|6714|6850	AKT1|ALOX5|ARG1|IL2|SRC|SYK	-2.05578	25	0	0	-9.48567	22.67311
-3.3675	GO:0032386	regulation of intracellular transport	19_GO:0051179 localization	-3.3675	6.189691	5.1477719	337	6	7.407407	2.909904	983|2932|5295|5743|5770|6714	CDK1|GSK3B|PIK3R1|PTGS2|PTPN1|SRC	-2.04954	16	0	0	-11.5558	31.96821
-3.36633	GO:0051051	negative regulation of transport	19_GO:0048519 negative regulation of biological process	-3.36633	5.14499	4.8828884	473	7	8.641975	3.122032	134|207|351|3156|4318|5743|10062	ADORA1|AKT1|APP|HMGCR|MMP9|PTGS2|NR1H3	-2.04894	21	0	0	-9.98291	27.26701
-3.36431	GO:0060043	regulation of cardiac muscle cell proliferation	19_GO:0040007 growth	-3.36431	20.45025	7.4670031	51	3	3.703704	2.098362	891|983|5292	CCNB1|CDK1|PIM1	-2.04806	35	0	0	-7.56218	38.62826
-3.36431	GO:0032964	collagen biosynthetic process	19_GO:0008152 metabolic process	-3.36431	20.45025	7.46	51	3	3.703704	2.098362	383|2147|5467	ARG1|F2|PPARD	-2.04806	65	0	0	-4.7415	57.94239

						7031										
-3.35661	GO:0009746	response to hexose	19_GO:0050896 response to stimulus	-3.35661	7.901235	5.519316	220	5	6.17284	2.674018	1080|3156|3480|5467|5743	CFTR|HMGCR|IGF1R|PPARD|PTGS2	-2.04094	28	0	0	-8.92043	22.1907
-3.35189	GO:0010811	positive regulation of cell-substrate adhesion	19_GO:0022610 biological adhesion	-3.35189	11.30583	6.152196	123	4	4.938272	2.407398	246|1021|2932|3791	ALOX15|CDK6|GSK3B|KDR	-2.03678	43	0	0	-6.76848	45.34622
-3.34765	GO:0008406	gonad development	19_GO:0022414 reproductive process	-3.34765	7.865482	5.503286	221	5	6.17284	2.674018	367|1588|2099|6714|8644	AR|CYP19A1|ESR1|SRC|AKR1C3	-2.03311	60	0	0	-5.48956	45.34622
-3.34637	GO:0000226	microtubule cytoskeleton organization	19_GO:0009987 cellular process	-3.34637	4.428717	4.667163	628	8	9.876543	3.314965	891|983|1017|2932|4137|4233|5347|9212	CCNB1|CDK1|CDK2|GSK3B|MAPT|MET|PLK1|AURKB	-2.03241	34	0	0	-7.56608	35.65685
-3.3394	GO:0042908	xenobiotic transport	19_GO:0051179 localization	-3.3394	20.05698	7.387561	52	3	3.703704	2.098362	4363|5243|9429	ABCC1|ABCB1|ABCG2	-2.02657	49	0	0	-6.34133	173.8272
-3.3394	GO:0043277	apoptotic cell clearance	19_GO:0051179 localization	-3.3394	20.05698	7.38	52	3	3.703704	2.098362	246|558|10062	ALOX15|AXL|NR1H3	-2.02657	55	0	0	-5.9859	20.05698

						7561											9	
-3.32709	GO:0030336	negative regulation of cell migration	19_GO:0040011 locomotion	-3.32709	6.081417	5.085539	343	6	7.407407	2.909904	134|140|207|328|1545|5467	ADORA1|ADORA3|AKT1|APEX1|CYP1B1|PPARD	-2.01482	76	1	1	-3.32709	11.71868
-3.32541	GO:0071621	granulocyte chemotaxis	19_GO:0040011 locomotion	-3.32541	11.12494	6.093368	125	4	4.938272	2.407398	3577|5294|5747|6850	CXCR1|PIK3CG|PTK2|SYK	-2.01371	42	0	0	-6.93111	31.60494
-3.31499	GO:1990090	cellular response to nerve growth factor stimulus	19_GO:0050896 response to stimulus	-3.31499	19.67855	7.310281	53	3	3.703704	2.098362	207|351|4137	AKT1|APP|MAPT	-2.00386	10	0	0	-13.3451	41.71852
-3.31222	GO:0002274	myeloid leukocyte activation	19_GO:0002376 immune system process	-3.31222	7.725652	5.440159	225	5	6.17284	2.674018	351|4137|5294|6850|10062	APP|MAPT|PIK3CG|SYK|NR1H3	-2.00165	44	0	0	-6.70994	13.24397
-3.30347	GO:0034284	response to monosaccharide	19_GO:0050896 response to stimulus	-3.30347	7.691467	5.42462	226	5	6.17284	2.674018	1080|3156|3480|5467|5743	CFTR|HMGCR|IGF1R|PPARD|PTGS2	-1.99347	28	0	0	-8.92043	22.1907
-3.29939	GO:0048565	digestive tract development	19_GO:0032502 developmental process	-3.29939	10.94974	6.035	127	4	4.938272	2.407398	891|1956|6714|7298	CCNB1|EGFR|SRC|TYMS	-1.98994	58	0	0	-5.66793	43.45679

<table>
<tr><td></td><td></td><td></td><td></td><td></td><td></td><td>861</td><td></td><td></td><td></td><td></td><td></td><td></td><td></td><td></td><td></td><td></td><td></td><td></td></tr>
<tr><td>-3.29477</td><td>GO:0002703</td><td>regulation of leukocyte mediated immunity</td><td>19_GO:0002376 immune system process</td><td>-3.29477</td><td>7.657584</td><td>5.409176</td><td>227</td><td>5</td><td>6.17284</td><td>2.674018</td><td>196|383|3558|4843|6850</td><td>AHR|ARG1|IL2|NOS2|SYK</td><td>-1.98645</td><td>56</td><td>0</td><td>0</td><td>-5.89598</td><td>130.3704</td></tr>
<tr><td>-3.29477</td><td>GO:0045137</td><td>development of primary sexual characteristics</td><td>19_GO:0022414 reproductive process</td><td>-3.29477</td><td>7.657584</td><td>5.409176</td><td>227</td><td>5</td><td>6.17284</td><td>2.674018</td><td>367|1588|2099|6714|8644</td><td>AR|CYP19A1|ESR1|SRC|AKR1C3</td><td>-1.98645</td><td>60</td><td>0</td><td>0</td><td>-5.48956</td><td>45.34622</td></tr>
<tr><td>-3.29106</td><td>GO:0002823</td><td>negative regulation of adaptive immune response based on somatic recombination of immune receptors built from immunoglobulin superfamily domains</td><td>19_GO:0002376 immune system process</td><td>-3.29106</td><td>19.31413</td><td>7.235509</td><td>54</td><td>3</td><td>3.703704</td><td>2.098362</td><td>196|383|3558</td><td>AHR|ARG1|IL2</td><td>-1.98384</td><td>56</td><td>0</td><td>0</td><td>-5.89598</td><td>130.3704</td></tr>
<tr><td>-3.29103</td><td>GO:0072503</td><td>cellular divalent inorganic cation homeostasis</td><td>19_GO:0065007 biological regulation</td><td>-3.29103</td><td>4.997085</td><td>4.779033</td><td>487</td><td>7</td><td>8.641975</td><td>3.122032</td><td>134|351|2099|2147|3558|3577|5294</td><td>ADORA1|APP|ESR1|F2|IL2|CXCR1|PIK3CG</td><td>-1.98384</td><td>38</td><td>0</td><td>0</td><td>-7.2588</td><td>41.71852</td></tr>
<tr><td>-3.27749</td><td>GO:0033157</td><td>regulation of intracellular protein transport</td><td>19_GO:0051179 localization</td><td>-3.27749</td><td>7.590706</td><td>5.378569</td><td>229</td><td>5</td><td>6.17284</td><td>2.674018</td><td>983|2932|5295|5743|5770</td><td>CDK1|GSK3B|PIK3R1|PTGS2|PTPN1</td><td>-1.97085</td><td>16</td><td>0</td><td>0</td><td>-11.5558</td><td>31.96821</td></tr>
<tr><td>-3.27523</td><td>GO:0009117</td><td>nucleotide metabolic process</td><td>19_GO:0008152 metabolic process</td><td>-3.27523</td><td>4.96649</td><td>4.75</td><td>490</td><td>7</td><td>8.641975</td><td>3.122032</td><td>142|351|3156|3643|4843|7298|7498</td><td>PARP1|APP|HMGCR|INSR|NOS2|TYMS|XDH</td><td>-1.96915</td><td>45</td><td>0</td><td>0</td><td>-6.5686</td><td>52.14815</td></tr>
</table>

						7296										7	
-3.26892	GO:0045444	fat cell differentiation	19_GO:0032502 developmental process	-3.26892	7.557703	5.363404	230	5	6.17284	2.674018	207|240|5292|5467|5743	AKT1|ALOX5|PIM1|PPARD|PTGS2	-1.96431	19	0	0	-10.8808	39.73192
-3.26892	GO:0001649	osteoblast differentiation	19_GO:0032502 developmental process	-3.26892	7.557703	5.363404	230	5	6.17284	2.674018	43|207|1021|2101|5747	ACHE|AKT1|CDK6|ESRRA|PTK2	-1.96431	31	0	0	-8.64333	10.15049
-3.2676	GO:0002067	glandular epithelial cell differentiation	19_GO:0032502 developmental process	-3.2676	18.96296	7.161893	55	3	3.703704	2.098362	207|1021|2932	AKT1|CDK6|GSK3B	-1.96431	43	0	0	-6.76848	45.34622
-3.2676	GO:0010676	positive regulation of cellular carbohydrate metabolic process	19_GO:0048518 positive regulation of biological process	-3.2676	18.96296	7.161893	55	3	3.703704	2.098362	207|3643|6714	AKT1|INSR|SRC	-1.96431	43	0	0	-6.76848	45.34622
-3.2676	GO:0061098	positive regulation of protein tyrosine kinase activity	19_GO:0048518 positive regulation of biological process	-3.2676	18.96296	7.161893	55	3	3.703704	2.098362	134|5770|6714	ADORA1|PTPN1|SRC	-1.96431	68	0	0	-3.8713	18.96296
-3.26476	GO:0048285	organelle fission	19_GO:0009987 cellular process	-3.26476	4.946301	4.74	492	7	8.641975	3.122032	891|3643|3791|4137|5347|7153|9212	CCNB1|INSR|KDR|MAPT|PLK1|TOP2A|AURKB	-1.96202	34	0	0	-7.5660	35.65685

						2903											8	
-3.25508	GO:0051480	regulation of cytosolic calcium ion concentration	19_GO:0065007 biological regulation	-3.25508	5.892446	4.975294	354	6	7.407407	2.909904	134|2099|2147|3558|3577|5294	ADORA1|ESR1|F2|IL2|CXCR1|PIK3CG	-1.95289	38	0	0	-7.2588	41.71852
-3.25189	GO:0030595	leukocyte chemotaxis	19_GO:0040011 locomotion	-3.25189	7.49255	5.333345	232	5	6.17284	2.674018	240|3577|5294|5747|6850	ALOX5|CXCR1|PIK3CG|PTK2|SYK	-1.95081	42	0	0	-6.93111	31.60494
-3.25189	GO:0045732	positive regulation of protein catabolic process	19_GO:0048518 positive regulation of biological process	-3.25189	7.49255	5.333345	232	5	6.17284	2.674018	207|1457|2932|5347|5747	AKT1|CSNK2A1|GSK3B|PLK1|PTK2	-1.95081	43	0	0	-6.76848	45.34622
-3.24865	GO:0000723	telomere maintenance	19_GO:0008152 metabolic process	-3.24865	10.6154	5.924606	131	4	4.938272	2.407398	142|328|6714|9212	PARP1|APEX1|SRC|AURKB	-1.94812	40	0	0	-7.12867	115.8848
-3.24459	GO:1990089	response to nerve growth factor	19_GO:0050896 response to stimulus	-3.24459	18.62434	7.090602	56	3	3.703704	2.098362	207|351|4137	AKT1|APP|MAPT	-1.94516	10	0	0	-13.3451	41.71852
-3.24459	GO:0051784	negative regulation of nuclear division	19_GO:0048519 negative regulation of	-3.24459	18.62434	7.09	56	3	3.703704	2.098362	891|5347|9212	CCNB1|PLK1|AURKB	-1.94516	34	0	0	-7.5660	35.65685

			biological process			0602											8	
-3.23589	GO:1904062	regulation of cation transmembrane transport	19_GO:0051179 localization	-3.23589	5.84293	4.946603	357	6	7.407407	2.909904	207|351|1612|2147|4318|5294	AKT1|APP|DAPK1|F2|MMP9|PIK3CG	-1.937	27	0	0	-8.92369	24.25495
-3.23366	GO:0006753	nucleoside phosphate metabolic process	19_GO:0008152 metabolic process	-3.23366	4.886707	4.7001866	498	7	8.641975	3.122032	142|351|3156|3643|4843|7298|7498	PARP1|APP|HMGCR|INSR|NOS2|TYMS|XDH	-1.93533	45	0	0	-6.56867	52.14815
-3.22953	GO:2000146	negative regulation of cell motility	19_GO:0040011 locomotion	-3.22953	5.826609	4.9363349	358	6	7.407407	2.909904	134|140|207|328|1545|5467	ADORA1|ADORA3|AKT1|APEX1|CYP1B1|PPARD	-1.93174	76	0	0	-3.32709	11.71868
-3.22391	GO:0046328	regulation of JNK cascade	19_GO:0023052 signaling	-3.22391	10.45577	5.8707765	133	4	4.938272	2.407398	207|351|1956|3480	AKT1|APP|EGFR|IGF1R	-1.92722	10	0	0	-13.3451	41.71852
-3.22391	GO:0046887	positive regulation of hormone secretion	19_GO:0048518 positive regulation of biological process	-3.22391	10.45577	5.8707765	133	4	4.938272	2.407398	1080|1588|1956|5467	CFTR|CYP19A1|EGFR|PPARD	-1.92722	28	0	0	-8.92043	22.1907
-3.22201	GO:0050891	multicellular organismal water homeostasis	19_GO:0032501 multicellular organismal process	-3.22201	18.2976	7.021	57	3	3.703704	2.098362	239|1080|4233	ALOX12|CFTR|MET	-1.92586	77	1	1	-3.22201	18.2976

						132												
-3.21833	GO:0033673	negative regulation of kinase activity	19_GO:0048519 negative regulation of biological process	-3.21833	7.365558	5.274289	236	5	6.17284	2.674018	207|3156|4137|5347|5770	AKT1|HMGCR|MAPT|PLK1|PTPN1	-1.92272	17	0	0	-11.2655	11.58848
-3.21169	GO:1903052	positive regulation of proteolysis involved in cellular protein catabolic process	19_GO:0048518 positive regulation of biological process	-3.21169	10.37774	5.844271	134	4	4.938272	2.407398	207|2932|5347|5747	AKT1|GSK3B|PLK1|PTK2	-1.91664	43	0	0	-6.76848	45.34622
-3.21059	GO:0051052	regulation of DNA metabolic process	19_GO:0050789 regulation of biological process	-3.21059	5.778188	4.907526	361	6	7.407407	2.909904	142|207|1956|3558|6714|9212	PARP1|AKT1|EGFR|IL2|SRC|AURKB	-1.91608	40	0	0	-7.12867	115.8848
-3.21004	GO:0019318	hexose metabolic process	19_GO:0008152 metabolic process	-3.21004	7.334479	5.259741	237	5	6.17284	2.674018	207|2932|3643|5467|6714	AKT1|GSK3B|INSR|PPARD|SRC	-1.91607	43	0	0	-6.76848	45.34622
-3.20908	GO:0055065	metal ion homeostasis	19_GO:0065007 biological regulation	-3.20908	4.220386	4.493003	659	8	9.876543	3.314965	134|351|1956|2099|2147|3558|3577|5294	ADORA1|APP|EGFR|ESR1|F2|IL2|CXCR1|PIK3CG	-1.91565	38	0	0	-7.2588	41.71852
-3.19984	GO:1902808	positive regulation of cell cycle G1/S phase transition	19_GO:0048518 positive regulation of biological process	-3.19984	17.98212	6.953	58	3	3.703704	2.098362	207|328|1956	AKT1|APEX1|EGFR	-1.90885	10	0	0	-13.3451	41.71852

						406												
-3.19984	GO:0001954	positive regulation of cell-matrix adhesion	19_GO:0022610 biological adhesion	-3.19984	17.98212	6.953406	58	3	3.703704	2.098362	1021|2932|3791	CDK6|GSK3B|KDR	-1.90885	43	0	0	-6.76848	45.34622
-3.19984	GO:0042743	hydrogen peroxide metabolic process	19_GO:0008152 metabolic process	-3.19984	17.98212	6.953406	58	3	3.703704	2.098362	1956|4314|4353	EGFR|MMP3|MPO	-1.90885	70	0	0	-3.77988	28.18819
-3.19984	GO:0010043	response to zinc ion	19_GO:0050896 response to stimulus	-3.19984	17.98212	6.953406	58	3	3.703704	2.098362	142|383|760	PARP1|ARG1|CA2	-1.90885	72	0	0	-3.67825	26.07407
-3.19957	GO:0032434	regulation of proteasomal ubiquitin-dependent protein catabolic process	19_GO:0050789 regulation of biological process	-3.19957	10.30087	5.818056	135	4	4.938272	2.407398	207|1017|2932|5347	AKT1|CDK2|GSK3B|PLK1	-1.90885	43	0	0	-6.76848	45.34622
-3.19357	GO:1903039	positive regulation of leukocyte cell-cell adhesion	19_GO:0022610 biological adhesion	-3.19357	7.273103	5.230897	239	5	6.17284	2.674018	207|240|3558|6714|6850	AKT1|ALOX5|IL2|SRC|SYK	-1.90338	25	0	0	-9.48567	22.67311
-3.18755	GO:0050714	positive regulation of protein secretion	19_GO:0048518 positive regulation of biological process	-3.18755	10.22513	5.792	136	4	4.938272	2.407398	43|1080|1956|5467	ACHE|CFTR|EGFR|PPARD	-1.8979	28	0	0	-8.92043	22.1907

						113												
-3.18281	GO:0001501	skeletal system development	19_GO:0032502 developmental process	-3.18281	4.790512	4.630488	508	7	8.641975	3.122032	2101|3292|4313|4318|4322|6714|7298	ESRRA|HSD17B1|MMP2|MMP9|MMP13|SRC|TYMS	-1.89369	69	0	0	-3.77988	28.18819
-3.17942	GO:0051271	negative regulation of cellular component movement	19_GO:0048519 negative regulation of biological process	-3.17942	5.699251	4.860198	366	6	7.407407	2.909904	134|140|207|328|1545|5467	ADORA1|ADORA3|AKT1|APEX1|CYP1B1|PPARD	-1.89085	76	0	0	-3.32709	11.71868
-3.17807	GO:0046324	regulation of glucose import	19_GO:0051179 localization	-3.17807	17.67734	6.88735	59	3	3.703704	2.098362	207|3643|5295	AKT1|INSR|PIK3R1	-1.89057	54	0	0	-6.06931	37.58425
-3.17807	GO:0090303	positive regulation of wound healing	19_GO:0048518 positive regulation of biological process	-3.17807	17.67734	6.88735	59	3	3.703704	2.098362	2147|4638|5747	F2|MYLK|PTK2	-1.89057	59	0	0	-5.59989	17.82843
-3.17726	GO:0044843	cell cycle G1/S phase transition	19_GO:0009987 cellular process	-3.17726	7.212745	5.202384	241	5	6.17284	2.674018	207|328|1017|1021|1956	AKT1|APEX1|CDK2|CDK6|EGFR	-1.89029	10	0	0	-13.3451	41.71852
-3.17563	GO:0060078	regulation of postsynaptic membrane potential	19_GO:0065007 biological regulation	-3.17563	10.15049	5.76643	137	4	4.938272	2.407398	134|207|351|2932	ADORA1|AKT1|APP|GSK3B	-1.88972	51	0	0	-6.31611	22.67311

						9												
-3.17563	GO:0055123	digestive system development	19_GO:0032502 developmental process	-3.17563	10.15049	5.766439	137	4	4.938272	2.407398	891|1956|6714|7298	CCNB1|EGFR|SRC|TYMS	-1.88972	58	0	0	-5.66793	43.45679
-3.1638	GO:0008203	cholesterol metabolic process	19_GO:0008152 metabolic process	-3.1638	10.07694	5.741027	138	4	4.938272	2.407398	351|1080|3156|5467	APP|CFTR|HMGCR|PPARD	-1.87896	28	0	0	-8.92043	22.1907
-3.1638	GO:0045471	response to ethanol	19_GO:0050896 response to stimulus	-3.1638	10.07694	5.741027	138	4	4.938272	2.407398	983|3156|3558|7298	CDK1|HMGCR|IL2|TYMS	-1.87896	32	0	0	-8.52802	13.79581
-3.15669	GO:0006081	cellular aldehyde metabolic process	19_GO:0008152 metabolic process	-3.15669	17.38272	6.822895	60	3	3.703704	2.098362	1545|1645|8644	CYP1B1|AKR1C1|AKR1C3	-1.87503	12	0	0	-13.14	260.7407
-3.15669	GO:0042306	regulation of protein import into nucleus	19_GO:0051179 localization	-3.15669	17.38272	6.822895	60	3	3.703704	2.098362	983|5295|5743	CDK1|PIK3R1|PTGS2	-1.87503	16	0	0	-11.5558	31.96821
-3.15669	GO:0010972	negative regulation of G2/M transition of mitotic cell cycle	19_GO:0048519 negative regulation of biological process	-3.15669	17.38272	6.82289	60	3	3.703704	2.098362	983|5347|9212	CDK1|PLK1|AURKB	-1.87503	34	0	0	-7.56608	35.65685

						5												
-3.15669	GO:0030071	regulation of mitotic metaphase/anaphase transition	19_GO:0050789 regulation of biological process	-3.15669	17.38272	6.822895	60	3	3.703704	2.098362	891|5347|9212	CCNB1|PLK1|AURKB	-1.87503	34	0	0	-7.56608	35.65685
-3.15669	GO:0060038	cardiac muscle cell proliferation	19_GO:0040007 growth	-3.15669	17.38272	6.822895	60	3	3.703704	2.098362	891|983|5292	CCNB1|CDK1|PIM1	-1.87503	35	0	0	-7.56218	38.62826
-3.15669	GO:0009620	response to fungus	19_GO:0044419 biological process involved in interspecies interaction between organisms	-3.15669	17.38272	6.822895	60	3	3.703704	2.098362	383|4353|6850	ARG1|MPO|SYK	-1.87503	56	0	0	-5.89598	130.3704
-3.15206	GO:0051783	regulation of nuclear division	19_GO:0050789 regulation of biological process	-3.15206	10.00444	5.715874	139	4	4.938272	2.407398	891|3643|5347|9212	CCNB1|INSR|PLK1|AURKB	-1.87092	34	0	0	-7.56608	35.65685
-3.14875	GO:0007159	leukocyte cell-cell adhesion	19_GO:0022610 biological adhesion	-3.14875	5.622442	4.813736	371	6	7.407407	2.909904	207|240|383|3558|6714|6850	AKT1|ALOX5|ARG1|IL2|SRC|SYK	-1.86814	25	0	0	-9.48567	22.67311
-3.14792	GO:0051249	regulation of lymphocyte activation	19_GO:0002376 immune system process	-3.14792	4.725399	4.58277	515	7	8.641975	3.122032	196|207|383|558|3558|6714|6850	AHR|AKT1|ARG1|AXL|IL2|SRC|SYK	-1.86784	25	0	0	-9.48567	22.67311

						3												
-3.13714	GO:0070663	regulation of leukocyte proliferation	19_GO:0050789 regulation of biological process	-3.13714	7.066145	5.132507	246	5	6.17284	2.674018	196|383|3558|5747|6850	AHR|ARG1|IL2|PTK2|SYK	-1.85759	56	0	0	-5.89598	130.3704
-3.13569	GO:1904356	regulation of telomere maintenance via telomere lengthening	19_GO:0050789 regulation of biological process	-3.13569	17.09775	6.759975	61	3	3.703704	2.098362	142|6714|9212	PARP1|SRC|AURKB	-1.85666	40	0	0	-7.12867	115.8848
-3.12885	GO:0030183	B cell differentiation	19_GO:0002376 immune system process	-3.12885	9.862534	5.666325	141	4	4.938272	2.407398	2322|3558|5295|6850	FLT3|IL2|PIK3R1|SYK	-1.8514	25	0	0	-9.48567	22.67311
-3.12885	GO:0034341	response to interferon-gamma	19_GO:0044419 biological process involved in interspecies interaction between organisms	-3.12885	9.862534	5.666325	141	4	4.938272	2.407398	383|1612|4843|10062	ARG1|DAPK1|NOS2|NR1H3	-1.8514	56	0	0	-5.89598	130.3704
-3.12885	GO:0003158	endothelium development	19_GO:0032502 developmental process	-3.12885	9.862534	5.666325	141	4	4.938272	2.407398	239|3791|4233|7498	ALOX12|KDR|MET|XDH	-1.8514	66	0	0	-4.22583	21.28496
-3.11739	GO:2000045	regulation of G1/S transition of mitotic cell cycle	19_GO:0050789 regulation of biological process	-3.11739	9.793079	5.64192	142	4	4.938272	2.407398	207|328|1017|1956	AKT1|APEX1|CDK2|EGFR	-1.84045	10	0	0	-13.3451	41.71852

-3.11505	GO:0045428	regulation of nitric oxide biosynthetic process	19_GO:0050789 regulation of biological process	-3.11505	16.82198	6.6985288	62	3	3.703704	2.098362	207|3643|5743	AKT1|INSR|PTGS2	-1.84019	19	0	0	-10.8808	39.73192
-3.11505	GO:0007091	metaphase/anaphase transition of mitotic cell cycle	19_GO:0050789 regulation of biological process	-3.11505	16.82198	6.6985288	62	3	3.703704	2.098362	891|5347|9212	CCNB1|PLK1|AURKB	-1.84019	34	0	0	-7.56608	35.65685
-3.11505	GO:1902750	negative regulation of cell cycle G2/M phase transition	19_GO:0048519 negative regulation of biological process	-3.11505	16.82198	6.6985288	62	3	3.703704	2.098362	983|5347|9212	CDK1|PLK1|AURKB	-1.84019	34	0	0	-7.56608	35.65685
-3.11505	GO:1903078	positive regulation of protein localization to plasma membrane	19_GO:0048518 positive regulation of biological process	-3.11505	16.82198	6.6985288	62	3	3.703704	2.098362	207|1956|5295	AKT1|EGFR|PIK3R1	-1.84019	54	0	0	-6.06931	37.58425
-3.11351	GO:1901988	negative regulation of cell cycle phase transition	19_GO:0048519 negative regulation of biological process	-3.11351	6.98101	5.091513	249	5	6.17284	2.674018	891|983|1017|5347|9212	CCNB1|CDK1|CDK2|PLK1|AURKB	-1.8397	34	0	0	-7.56608	35.65685
-3.11351	GO:0006898	receptor-mediated endocytosis	19_GO:0051179 localization	-3.11351	6.98101	5.091513	249	5	6.17284	2.674018	43|351|3577|3643|6850	ACHE|APP|CXCR1|INSR|SYK	-1.8397	44	0	0	-6.70994	13.24397

-3.09475	GO:1904589	regulation of protein import	19_GO:0051179 localization	-3.09475	16.55497	6.6384966	63	3	3.703704	2.098362	983|5295|5743	CDK1|PIK3R1|PTGS2	-1.82506	16	0	0	-11.5558	31.96821
-3.09475	GO:1902099	regulation of metaphase/anaphase transition of cell cycle	19_GO:0050789 regulation of biological process	-3.09475	16.55497	6.6384966	63	3	3.703704	2.098362	891|5347|9212	CCNB1|PLK1|AURKB	-1.82506	34	0	0	-7.56608	35.65685
-3.09475	GO:0031343	positive regulation of cell killing	19_GO:0048518 positive regulation of biological process	-3.09475	16.55497	6.6384966	63	3	3.703704	2.098362	383|4843|6850	ARG1|NOS2|SYK	-1.82506	56	0	0	-5.89598	130.3704
-3.09475	GO:0099601	regulation of neurotransmitter receptor activity	19_GO:0023052 signaling	-3.09475	16.55497	6.6384966	63	3	3.703704	2.098362	351|1612|6714	APP|DAPK1|SRC	-1.82506	68	0	0	-3.8713	18.96296
-3.09472	GO:0033135	regulation of peptidyl-serine phosphorylation	19_GO:0050789 regulation of biological process	-3.09472	9.657064	5.593828	144	4	4.938272	2.407398	207|351|1956|5743	AKT1|APP|EGFR|PTGS2	-1.82506	19	0	0	-10.8808	39.73192
-3.09472	GO:0007612	learning	19_GO:0007610 behavior	-3.09472	9.657064	5.593828	144	4	4.938272	2.407398	351|3156|3643|5743	APP|HMGCR|INSR|PTGS2	-1.82506	45	0	0	-6.56867	52.14815

-3.09472	GO:0106106	cold-induced thermogenesis	19_GO:0032501 multicellular organismal process	-3.09472	9.657064	5.593828	144	4	4.938272	2.407398	43|3480|6850|10062	ACHE|IGF1R|SYK|NR1H3	-1.82506	53	0	0	-6.14202	13.98609
-3.09472	GO:0120161	regulation of cold-induced thermogenesis	19_GO:0032501 multicellular organismal process	-3.09472	9.657064	5.593828	144	4	4.938272	2.407398	43|3480|6850|10062	ACHE|IGF1R|SYK|NR1H3	-1.82506	53	0	0	-6.14202	13.98609
-3.09424	GO:0051493	regulation of cytoskeleton organization	19_GO:0050789 regulation of biological process	-3.09424	4.626578	4.50995	526	7	8.641975	3.122032	246|2932|4137|4233|5295|5347|5747	ALOX15|GSK3B|MAPT|MET|PIK3R1|PLK1|PTK2	-1.82506	50	0	0	-6.31767	11.54039
-3.0902	GO:0003015	heart process	19_GO:0032501 multicellular organismal process	-3.0902	6.897903	5.051192	252	5	6.17284	2.674018	134|140|3558|5294|6714	ADORA1|ADORA3|IL2|PIK3CG|SRC	-1.82205	38	0	0	-7.2588	41.71852
-3.0902	GO:0045017	glycerolipid biosynthetic process	19_GO:0008152 metabolic process	-3.0902	6.897903	5.051192	252	5	6.17284	2.674018	43|246|5294|5295|10062	ACHE|ALOX15|PIK3CG|PIK3R1|NR1H3	-1.82205	71	0	0	-3.73717	6.897903
-3.08882	GO:0030900	forebrain development	19_GO:0032502 developmental process	-3.08882	5.474871	4.723295	381	6	7.407407	2.909904	238|351|558|1021|2932|6714	ALK|APP|AXL|CDK6|GSK3B|SRC	-1.82118	52	0	0	-6.26187	13.03704
-3.084	GO:0006914	autophagy	19_GO:0008152 metabolic process	-3.084	4.609053	4.4	528	7	8.641975	3.122032	207|1612|2932|3791|4137|4233|	AKT1|DAPK1|GSK3B|KDR|MAPT|MET|SRC	-1.818	43	0	0	-6.7	45.34622

62				62		96395					6714						6848	
-3.08462	GO:0061919	process utilizing autophagic mechanism	19_GO:0009987 cellular process	-3.08462	4.609053	4.496395	528	7	8.641975	3.122032	207|1612|2932|3791|4137|4233|6714	AKT1|DAPK1|GSK3B|KDR|MAPT|MET|SRC	-1.818	43	0	0	-6.76848	45.34622
-3.08351	GO:0001890	placenta development	19_GO:0022414 reproductive process	-3.08351	9.590464	5.5570134	145	4	4.938272	2.407398	207|5467|5743|5747	AKT1|PPARD|PTGS2|PTK2	-1.8174	19	0	0	-10.8808	39.73192
-3.0825	GO:0008654	phospholipid biosynthetic process	19_GO:0008152 metabolic process	-3.0825	6.870639	5.037898	253	5	6.17284	2.674018	43|246|5294|5295|5467	ACHE|ALOX15|PIK3CG|PIK3R1|PPARD	-1.8169	71	0	0	-3.73717	6.897903
-3.07983	GO:0051640	organelle localization	19_GO:0051179 localization	-3.07983	4.600341	4.489867	529	7	8.641975	3.122032	891|1080|4137|5294|5347|6850|9212	CCNB1|CFTR|MAPT|PIK3CG|PLK1|SYK|AURKB	-1.81474	34	0	0	-7.56608	35.65685
-3.07484	GO:0009743	response to carbohydrate	19_GO:0050896 response to stimulus	-3.07484	6.843589	5.024676	254	5	6.17284	2.674018	1080|3156|3480|5467|5743	CFTR|HMGCR|IGF1R|PPARD|PTGS2	-1.81274	28	0	0	-8.92043	22.1907
-3.074	GO:0071560	cellular response to transforming growth	19_GO:0050896 response to	-3.074	6.843589	5.0	254	5	6.17284	2.674018	142|383|2908|5747|6714	PARP1|ARG1|NR3C1|PTK2|SRC	-1.8127	29	0	0	-8.8	35.96424

84		factor beta stimulus	stimulus	84		24676							4				7872	
-3.0748	GO:0080164	regulation of nitric oxide metabolic process	19_GO:0050789 regulation of biological process	-3.0748	16.2963	6.579825	64	3	3.703704	2.098362	207|3643|5743	AKT1|INSR|PTGS2	-1.81274	19	0	0	-10.8808	39.73192
-3.0748	GO:0061178	regulation of insulin secretion involved in cellular response to glucose stimulus	19_GO:0051179 localization	-3.0748	16.2963	6.579825	64	3	3.703704	2.098362	1080|3156|5467	CFTR|HMGCR|PPARD	-1.81274	28	0	0	-8.92043	22.1907
-3.0748	GO:0045670	regulation of osteoclast differentiation	19_GO:0002376 immune system process	-3.0748	16.2963	6.579825	64	3	3.703704	2.098362	760|2101|5295	CA2|ESRRA|PIK3R1	-1.81274	37	0	0	-7.31428	16.2963
-3.0748	GO:0030104	water homeostasis	19_GO:0065007 biological regulation	-3.0748	16.2963	6.579825	64	3	3.703704	2.098362	239|1080|4233	ALOX12|CFTR|MET	-1.81274	77	0	0	-3.22201	18.2976
-3.07239	GO:0038061	NIK/NF-kappaB signaling	19_GO:0023052 signaling	-3.07239	9.524776	5.546668	146	4	4.938272	2.407398	207|238|351|1956	AKT1|ALK|APP|EGFR	-1.81134	10	0	0	-13.3451	41.71852
-3.072	GO:0010675	regulation of cellular carbohydrate	19_GO:0050789 regulation of	-3.072	9.524776	5.5	146	4	4.938272	2.407398	207|2932|3643|6714	AKT1|GSK3B|INSR|SRC	-1.8113	43	0	0	-6.7	45.34622

39		metabolic process	biological process	39		46668							4				6848	
-3.07121	GO:0022412	cellular process involved in reproduction in multicellular organism	19_GO:0022414 reproductive process	-3.07121	5.432099	4.696783	384	6	7.407407	2.909904	207|891|1080|5347|6714|7153	AKT1|CCNB1|CFTR|PLK1|SRC|TOP2A	-1.81116	58	0	0	-5.66793	43.45679
-3.07121	GO:0006644	phospholipid metabolic process	19_GO:0008152 metabolic process	-3.07121	5.432099	4.696783	384	6	7.407407	2.909904	43|246|5294|5295|5467|10062	ACHE|ALOX15|PIK3CG|PIK3R1|PPARD|NR1H3	-1.81116	71	0	0	-3.73717	6.897903
-3.06721	GO:0051924	regulation of calcium ion transport	19_GO:0051179 localization	-3.06721	6.816751	5.011525	255	5	6.17284	2.674018	207|2147|4638|5294|5743	AKT1|F2|MYLK|PIK3CG|PTGS2	-1.80767	64	0	0	-4.94984	45.34622
-3.06135	GO:0001889	liver development	19_GO:0032502 developmental process	-3.06135	9.459982	5.523426	147	4	4.938272	2.407398	383|1956|4233|7298	ARG1|EGFR|MET|TYMS	-1.80231	29	0	0	-8.87872	35.96424
-3.05518	GO:0010965	regulation of mitotic sister chromatid separation	19_GO:0050789 regulation of biological process	-3.05518	16.04558	6.522461	65	3	3.703704	2.098362	891|5347|9212	CCNB1|PLK1|AURKB	-1.79913	34	0	0	-7.56608	35.65685
-3.055	GO:0044784	metaphase/anaphase transition of cell cycle	19_GO:0050789 regulation of	-3.055	16.04558	6.5	65	3	3.703704	2.098362	891|5347|9212	CCNB1|PLK1|AURKB	-1.7991	34	0	0	-7.5	35.65685

18			biological process	18		22461							3				6608	
-3.05518	GO:0038034	signal transduction in absence of ligand	19_GO:0023052 signaling	-3.05518	16.04558	6.522461	65	3	3.703704	2.098362	207|2932|3558	AKT1|GSK3B|IL2	-1.79913	43	0	0	-6.76848	45.34622
-3.05518	GO:0097192	extrinsic apoptotic signaling pathway in absence of ligand	19_GO:0023052 signaling	-3.05518	16.04558	6.522461	65	3	3.703704	2.098362	207|2932|3558	AKT1|GSK3B|IL2	-1.79913	43	0	0	-6.76848	45.34622
-3.05518	GO:1905330	regulation of morphogenesis of an epithelium	19_GO:0032502 developmental process	-3.05518	16.04558	6.522461	65	3	3.703704	2.098362	239|367|2099	ALOX12|AR|ESR1	-1.79913	60	0	0	-5.48956	45.34622
-3.05518	GO:1900076	regulation of cellular response to insulin stimulus	19_GO:0050896 response to stimulus	-3.05518	16.04558	6.522461	65	3	3.703704	2.098362	2101|5770|6714	ESRRA|PTPN1|SRC	-1.79913	68	0	0	-3.8713	18.96296
-3.05205	GO:0005996	monosaccharide metabolic process	19_GO:0008152 metabolic process	-3.05205	6.763703	4.985434	257	5	6.17284	2.674018	207|2932|3643|5467|6714	AKT1|GSK3B|INSR|PPARD|SRC	-1.7965	43	0	0	-6.76848	45.34622
-3.050	GO:1902652	secondary alcohol metabolic process	19_GO:0008152 metabolic process	-3.050	9.396063	5.5	148	4	4.938272	2.407398	351|1080|3156|5467	APP|CFTR|HMGCR|PPARD	-1.7953	28	0	0	-8.9	22.1907

39				39		00406							4				2043	
-3.03952	GO:0097530	granulocyte migration	19_GO:0040011 locomotion	-3.03952	9.333002	5.477604	149	4	4.938272	2.407398	3577|5294|5747|6850	CXCR1|PIK3CG|PTK2|SYK	-1.78546	42	0	0	-6.93111	31.60494
-3.03952	GO:0070507	regulation of microtubule cytoskeleton organization	19_GO:0050789 regulation of biological process	-3.03952	9.333002	5.477604	149	4	4.938272	2.407398	2932|4137|4233|5347	GSK3B|MAPT|MET|PLK1	-1.78546	50	0	0	-6.31767	11.54039
-3.03645	GO:0040013	negative regulation of locomotion	19_GO:0040011 locomotion	-3.03645	5.348528	4.644585	390	6	7.407407	2.909904	134|140|207|328|1545|5467	ADORA1|ADORA3|AKT1|APEX1|CYP1B1|PPARD	-1.78477	76	0	0	-3.32709	11.71868
-3.03587	GO:1905953	negative regulation of lipid localization	19_GO:0048519 negative regulation of biological process	-3.03587	15.80247	6.466357	66	3	3.703704	2.098362	207|5467|10062	AKT1|PPARD|NR1H3	-1.78477	19	0	0	-10.8808	39.73192
-3.03587	GO:0051893	regulation of focal adhesion assembly	19_GO:0022610 biological adhesion	-3.03587	15.80247	6.466357	66	3	3.703704	2.098362	3791|5747|6714	KDR|PTK2|SRC	-1.78477	41	0	0	-7.04885	38.62826
-3.035	GO:0090109	regulation of cell-substrate junction	19_GO:0050789 regulation of	-3.035	15.80247	6.4	66	3	3.703704	2.098362	3791|5747|6714	KDR|PTK2|SRC	-1.7847	41	0	0	-7.0	38.62826

87		assembly	biological process	87		66357							7				4885	
-3.03587	GO:0033692	cellular polysaccharide biosynthetic process	19_GO:0008152 metabolic process	-3.03587	15.80247	6.466357	66	3	3.703704	2.098362	207|2932|3643	AKT1|GSK3B|INSR	-1.78477	43	0	0	-6.76848	45.34622
-3.03587	GO:0042446	hormone biosynthetic process	19_GO:0008152 metabolic process	-3.03587	15.80247	6.466357	66	3	3.703704	2.098362	1586|1588|3292	CYP17A1|CYP19A1|HSD17B1	-1.78477	67	0	0	-4.05548	34.76543
-3.02955	GO:0071559	response to transforming growth factor beta	19_GO:0050896 response to stimulus	-3.02955	6.68566	4.946816	260	5	6.17284	2.674018	142|383|2908|5747|6714	PARP1|ARG1|NR3C1|PTK2|SRC	-1.77909	29	0	0	-8.87872	35.96424
-3.02872	GO:0051592	response to calcium ion	19_GO:0050896 response to stimulus	-3.02872	9.270782	5.455015	150	4	4.938272	2.407398	246|1956|8644|9536	ALOX15|EGFR|AKR1C3|PTGES	-1.77909	15	0	0	-12.2065	35.47493
-3.02872	GO:0061008	hepaticobiliary system development	19_GO:0032502 developmental process	-3.02872	9.270782	5.455015	150	4	4.938272	2.407398	383|1956|4233|7298	ARG1|EGFR|MET|TYMS	-1.77909	29	0	0	-8.87872	35.96424
-3.018	GO:2001236	regulation of extrinsic apoptotic signaling	19_GO:0023052 signaling	-3.018	9.209386	5.4	151	4	4.938272	2.407398	207|367|2932|6714	AKT1|AR|GSK3B|SRC	-1.7693	43	0	0	-6.7	45.34622

		pathway				32637							6				6848	
-3.018	GO:0008643	carbohydrate transport	19_GO:0051179 localization	-3.018	9.209386	5.4326377	151	4	4.938272	2.407398	207|3643|5295|5467	AKT1|INSR|PIK3R1|PPARD	-1.76936	54	0	0	-6.06931	37.58425
-3.01687	GO:0051306	mitotic sister chromatid separation	19_GO:0009987 cellular process	-3.01687	15.56661	6.411464	67	3	3.703704	2.098362	891|5347|9212	CCNB1|PLK1|AURKB	-1.7692	34	0	0	-7.56608	35.65685
-3.01687	GO:0046626	regulation of insulin receptor signaling pathway	19_GO:0023052 signaling	-3.01687	15.56661	6.411464	67	3	3.703704	2.098362	5295|5770|6714	PIK3R1|PTPN1|SRC	-1.7692	41	0	0	-7.04885	38.62826
-3.00736	GO:0043535	regulation of blood vessel endothelial cell migration	19_GO:0040011 locomotion	-3.00736	9.148798	5.410465	152	4	4.938272	2.407398	207|239|3791|5743	AKT1|ALOX12|KDR|PTGS2	-1.76115	19	0	0	-10.8808	39.73192
-3.00736	GO:0071333	cellular response to glucose stimulus	19_GO:0050896 response to stimulus	-3.00736	9.148798	5.410465	152	4	4.938272	2.407398	1080|3156|3480|5467	CFTR|HMGCR|IGF1R|PPARD	-1.76115	28	0	0	-8.92043	22.1907
-3.007	GO:0044409	entry into host	19_GO:0044419 biological process	-3.007	9.148798	5.4	152	4	4.938272	2.407398	558|983|1956|6714	AXL|CDK1|EGFR|SRC	-1.7611	57	0	0	-5.7	22.87199

36			involved in interspecies interaction between organisms	36		10465							5				6668	
-3.00004	GO:1905114	cell surface receptor signaling pathway involved in cell-cell signaling	19_GO:0023052 signaling	-3.00004	4.457107	4.381318	546	7	8.641975	3.122032	134|207|351|1457|1956|2932|6714	ADORA1|AKT1|APP|CSNK2A1|EGFR|GSK3B|SRC	-1.75431	51	0	0	-6.31611	22.67311
-2.99817	GO:0070527	platelet aggregation	19_GO:0022610 biological adhesion	-2.99817	15.33769	6.357738	68	3	3.703704	2.098362	239|5294|6850	ALOX12|PIK3CG|SYK	-1.75342	9	0	0	-13.6942	26.07407
-2.99817	GO:0010812	negative regulation of cell-substrate adhesion	19_GO:0022610 biological adhesion	-2.99817	15.33769	6.357738	68	3	3.703704	2.098362	4321|5295|6714	MMP12|PIK3R1|SRC	-1.75342	41	0	0	-7.04885	38.62826
-2.99669	GO:0006163	purine nucleotide metabolic process	19_GO:0008152 metabolic process	-2.99669	5.254221	4.585033	397	6	7.407407	2.909904	142|351|3156|3643|4843|7498	PARP1|APP|HMGCR|INSR|NOS2|XDH	-1.75242	45	0	0	-6.56867	52.14815
-2.98631	GO:0071331	cellular response to hexose stimulus	19_GO:0050896 response to stimulus	-2.98631	9.029982	5.36673	154	4	4.938272	2.407398	1080|3156|3480|5467	CFTR|HMGCR|IGF1R|PPARD	-1.74253	28	0	0	-8.92043	22.1907
-2.98548	GO:0051656	establishment of organelle localization	19_GO:0051179 localization	-2.98548	5.227885	4.56	399	6	7.407407	2.909904	891|4137|5294|5347|6850|9212	CCNB1|MAPT|PIK3CG|PLK1|SYK|AURKB	-1.74218	34	0	0	-7.5660	35.65685

						8276											8	
-2.97976	GO:1904377	positive regulation of protein localization to cell periphery	19_GO:0048518 positive regulation of biological process	-2.97976	15.11541	6.305138	69	3	3.703704	2.098362	207|1956|5295	AKT1|EGFR|PIK3R1	-1.73791	54	0	0	-6.06931	37.58425
-2.97976	GO:0051966	regulation of synaptic transmission, glutamatergic	19_GO:0023052 signaling	-2.97976	15.11541	6.305138	69	3	3.703704	2.098362	134|1956|5743	ADORA1|EGFR|PTGS2	-1.73791	61	0	0	-5.47166	27.44639
-2.97976	GO:0060193	positive regulation of lipase activity	19_GO:0065007 biological regulation	-2.97976	15.11541	6.305138	69	3	3.703704	2.098362	1956|2099|10062	EGFR|ESR1|NR1H3	-1.73791	61	0	0	-5.47166	27.44639
-2.9782	GO:0032412	regulation of ion transmembrane transporter activity	19_GO:0051179 localization	-2.9782	6.510381	4.859034	267	5	6.17284	2.674018	351|1080|1612|4318|5243	APP|CFTR|DAPK1|MMP9|ABCB1	-1.73682	27	0	0	-8.92369	24.25495
-2.9759	GO:0071326	cellular response to monosaccharide stimulus	19_GO:0050896 response to stimulus	-2.9759	8.971724	5.345159	155	4	4.938272	2.407398	1080|3156|3480|5467	CFTR|HMGCR|IGF1R|PPARD	-1.73501	28	0	0	-8.92043	22.1907
-2.97099	GO:0001894	tissue homeostasis	19_GO:0032501 multicellular organismal	-2.97099	6.486088	4.844	268	5	6.17284	2.674018	760|1956|5743|6714|6850	CA2|EGFR|PTGS2|SRC|SYK	-1.73057	36	0	0	-7.3445	54.89279

			process			675											6	
−2.96556	GO:1903364	positive regulation of cellular protein catabolic process	19_GO:0048518 positive regulation of biological process	−2.96556	8.914213	5.3237783	156	4	4.938272	2.407398	207|2932|5347|5747	AKT1|GSK3B|PLK1|PTK2	−1.72563	43	0	0	−6.76848	45.34622
−2.96164	GO:0034605	cellular response to heat	19_GO:0050896 response to stimulus	−2.96164	14.89947	6.2536223	70	3	3.703704	2.098362	2932|4137|5743	GSK3B|MAPT|PTGS2	−1.72219	51	0	0	−6.31611	22.67311
−2.95529	GO:1990845	adaptive thermogenesis	19_GO:0008152 metabolic process	−2.95529	8.857435	5.3025597	157	4	4.938272	2.407398	43|3480|6850|10062	ACHE|IGF1R|SYK|NR1H3	−1.71632	53	0	0	−6.14202	13.98609
−2.94676	GO:0010959	regulation of metal ion transport	19_GO:0051179 localization	−2.94676	5.137749	4.5104999	406	6	7.407407	2.909904	134|207|2147|4638|5294|5743	ADORA1|AKT1|F2|MYLK|PIK3CG|PTGS2	−1.70827	64	0	0	−4.94984	45.34622
−2.94378	GO:0045824	negative regulation of innate immune response	19_GO:0044419 biological process involved in interspecies interaction between organisms	−2.94378	14.68962	6.2031556	71	3	3.703704	2.098362	383|4321|10062	ARG1|MMP12|NR1H3	−1.70577	55	0	0	−5.98599	20.05698
−2.93539	GO:0048872	homeostasis of number of cells	19_GO:0065007 biological regulation	−2.93539	6.367295	4.786	273	5	6.17284	2.674018	207|558|1021|2322|3558	AKT1|AXL|CDK6|FLT3|IL2	−1.69886	25	0	0	−9.48567	22.67311

						259											
-2.93497	GO:1902107	positive regulation of leukocyte differentiation	19_GO:0002376 immune system process	-2.93497	8.746021	5.260787	159	4	4.938272	2.407398	558|760|3558|6850	AXL|CA2|IL2|SYK	-1.69886	25	0	0	-9.48567	22.67311
-2.93497	GO:1903708	positive regulation of hemopoiesis	19_GO:0002376 immune system process	-2.93497	8.746021	5.260787	159	4	4.938272	2.407398	558|760|3558|6850	AXL|CA2|IL2|SYK	-1.69886	25	0	0	-9.48567	22.67311
-2.93497	GO:0032200	telomere organization	19_GO:0009987 cellular process	-2.93497	8.746021	5.260787	159	4	4.938272	2.407398	142|328|6714|9212	PARP1|APEX1|SRC|AURKB	-1.69886	40	0	0	-7.12867	115.8848
-2.9262	GO:0070227	lymphocyte apoptotic process	19_GO:0009987 cellular process	-2.9262	14.4856	6.1537	72	3	3.703704	2.098362	207|3558|9212	AKT1|IL2|AURKB	-1.6934	25	0	0	-9.48567	22.67311
-2.9262	GO:0033045	regulation of sister chromatid segregation	19_GO:0050789 regulation of biological process	-2.9262	14.4856	6.1537	72	3	3.703704	2.098362	891|5347|9212	CCNB1|PLK1|AURKB	-1.6934	34	0	0	-7.56608	35.65685
-2.9262	GO:0055021	regulation of cardiac muscle tissue growth	19_GO:0040007 growth	-2.9262	14.4856	6.1537	72	3	3.703704	2.098362	891|983|5292	CCNB1|CDK1|PIM1	-1.6934	35	0	0	-7.56218	38.62826
-2.9262	GO:0005977	glycogen metabolic process	19_GO:0008152 metabolic process	-2.9262	14.4856	6.15	72	3	3.703704	2.098362	207|2932|3643	AKT1|GSK3B|INSR	-1.6934	43	0	0	-6.7684	45.34622

						37											8	
-2.9262	GO:0002534	cytokine production involved in inflammatory response	19_GO:0032501 multicellular organismal process	-2.9262	14.4856	6.15377	72	3	3.703704	2.098362	240|2147|4843	ALOX5|F2|NOS2	-1.6934	59	0	0	-5.59989	17.82843
-2.9262	GO:1900015	regulation of cytokine production involved in inflammatory response	19_GO:0032501 multicellular organismal process	-2.9262	14.4856	6.15377	72	3	3.703704	2.098362	240|2147|4843	ALOX5|F2|NOS2	-1.6934	59	0	0	-5.59989	17.82843
-2.9262	GO:1903036	positive regulation of response to wounding	19_GO:0048518 positive regulation of biological process	-2.9262	14.4856	6.15377	72	3	3.703704	2.098362	2147|4638|5747	F2|MYLK|PTK2	-1.6934	59	0	0	-5.59989	17.82843
-2.92492	GO:0090316	positive regulation of intracellular protein transport	19_GO:0048518 positive regulation of biological process	-2.92492	8.691358	5.240157	160	4	4.938272	2.407398	983|2932|5295|5743	CDK1|GSK3B|PIK3R1|PTGS2	-1.69259	16	0	0	-11.5558	31.96821
-2.91494	GO:0043271	negative regulation of ion transport	19_GO:0048519 negative regulation of biological process	-2.91494	8.637374	5.219706	161	4	4.938272	2.407398	134|207|4318|5743	ADORA1|AKT1|MMP9|PTGS2	-1.68308	21	0	0	-9.98291	27.26701
-2.90887	GO:0050848	regulation of calcium-mediated signaling	19_GO:0023052 signaling	-2.90887	14.28716	6.10522	73	3	3.703704	2.098362	2932|4137|6850	GSK3B|MAPT|SYK	-1.67982	7	0	0	-14.1215	57.94239
-2.90887	GO:0035773	insulin secretion involved in cellular response to glucose stimulus	19_GO:0051179 localization	-2.90887	14.28716	6.1052	73	3	3.703704	2.098362	1080|3156|5467	CFTR|HMGCR|PPARD	-1.67982	28	0	0	-8.92043	22.1907

-2.90887	GO:0000271	polysaccharide biosynthetic process	19_GO:0008152 metabolic process	-2.90887	14.28716	26.1052	73	3	3.703704	2.098362	207|2932|3643	AKT1|GSK3B|INSR	-1.67982	43	0	0	-6.76848	45.34622
-2.90887	GO:0006073	cellular glucan metabolic process	19_GO:0008152 metabolic process	-2.90887	14.28716	26.1052	73	3	3.703704	2.098362	207|2932|3643	AKT1|GSK3B|INSR	-1.67982	43	0	0	-6.76848	45.34622
-2.90887	GO:0044042	glucan metabolic process	19_GO:0008152 metabolic process	-2.90887	14.28716	26.1052	73	3	3.703704	2.098362	207|2932|3643	AKT1|GSK3B|INSR	-1.67982	43	0	0	-6.76848	45.34622
-2.90887	GO:0042058	regulation of epidermal growth factor receptor signaling pathway	19_GO:0023052 signaling	-2.90887	14.28716	26.1052	73	3	3.703704	2.098362	134|1956|4318	ADORA1|EGFR|MMP9	-1.67982	61	0	0	-5.47166	27.44639
-2.90744	GO:0046034	ATP metabolic process	19_GO:0008152 metabolic process	-2.90744	6.275349	24.73894	277	5	6.17284	2.674018	142|351|891|983|3643	PARP1|APP|CCNB1|CDK1|INSR	-1.67886	35	0	0	-7.56218	38.62826
-2.89281	GO:0016032	viral process	19_GO:0016032 viral process	-2.89281	5.014245	44.4302	416	6	7.407407	2.909904	558|983|1956|2932|3643|7153	AXL|CDK1|EGFR|GSK3B|INSR|TOP2A	-1.66554	57	0	0	-5.76668	22.87199
-2.89179	GO:1901568	fatty acid derivative metabolic process	19_GO:0008152 metabolic process	-2.89179	14.09409	36.057	74	3	3.703704	2.098362	239|240|246	ALOX12|ALOX5|ALOX15	-1.66554	11	0	0	-13.1836	260.7407

						685												
-2.89179	GO:0033143	regulation of intracellular steroid hormone receptor signaling pathway	19_GO:0023052 signaling	-2.89179	14.09409	6.057685	74	3	3.703704	2.098362	142|367|6714	PARP1|AR|SRC	-1.66554	40	0	0	-7.12867	115.8848
-2.89179	GO:0010822	positive regulation of mitochondrion organization	19_GO:0048518 positive regulation of biological process	-2.89179	14.09409	6.057685	74	3	3.703704	2.098362	2932|3791|4318	GSK3B|KDR|MMP9	-1.66554	43	0	0	-6.76848	45.34622
-2.89179	GO:0046323	glucose import	19_GO:0051179 localization	-2.89179	14.09409	6.057685	74	3	3.703704	2.098362	207|3643|5295	AKT1|INSR|PIK3R1	-1.66554	54	0	0	-6.06931	37.58425
-2.88539	GO:0071322	cellular response to carbohydrate stimulus	19_GO:0050896 response to stimulus	-2.88539	8.479374	5.159403	164	4	4.938272	2.407398	1080|3156|3480|5467	CFTR|HMGCR|IGF1R|PPARD	-1.6596	28	0	0	-8.92043	22.1907
-2.87496	GO:0043627	response to estrogen	19_GO:0050896 response to stimulus	-2.87496	13.90617	6.011062	75	3	3.703704	2.098362	367|760|2099	AR|CA2|ESR1	-1.64964	60	0	0	-5.48956	45.34622
-2.86637	GO:0015850	organic hydroxy compound transport	19_GO:0051179 localization	-2.86637	6.142302	4.669	283	5	6.17284	2.674018	1080|1588|1645|6850|10062	CFTR|CYP19A1|AKR1C1|SYK|NR1H3	-1.64152	74	0	0	-3.61486	24.83245

						691												
-2.85836	GO:0140115	export across plasma membrane	19_GO:0051179 localization	-2.85836	13.7232	5.965322	76	3	3.703704	2.098362	4363|5243|9429	ABCC1|ABCB1|ABCG2	-1.63397	49	0	0	-6.34133	173.8272
-2.85643	GO:0007254	JNK cascade	19_GO:0023052 signaling	-2.85643	8.32705	5.10062	167	4	4.938272	2.407398	207|351|1956|3480	AKT1|APP|EGFR|IGF1R	-1.63342	10	0	0	-13.3451	41.71852
-2.85643	GO:0002833	positive regulation of response to biotic stimulus	19_GO:0048518 positive regulation of biological process	-2.85643	8.32705	5.10062	167	4	4.938272	2.407398	383|4321|6714|6850	ARG1|MMP12|SRC|SYK	-1.63342	41	0	0	-7.04885	38.62826
-2.85643	GO:0055088	lipid homeostasis	19_GO:0065007 biological regulation	-2.85643	8.32705	5.10062	167	4	4.938272	2.407398	134|1645|3156|10062	ADORA1|AKR1C1|HMGCR|NR1H3	-1.63342	74	0	0	-3.61486	24.83245
-2.84691	GO:1902806	regulation of cell cycle G1/S phase transition	19_GO:0050789 regulation of biological process	-2.84691	8.277484	5.081351	168	4	4.938272	2.407398	207|328|1017|1956	AKT1|APEX1|CDK2|EGFR	-1.62436	10	0	0	-13.3451	41.71852
-2.842	GO:0010827	regulation of glucose transmembrane transport	19_GO:0051179 localization	-2.842	13.54497	5.920436	77	3	3.703704	2.098362	207|3643|5295	AKT1|INSR|PIK3R1	-1.61991	54	0	0	-6.06931	37.58425

-2.84067	GO:0006875	cellular metal ion homeostasis	19_GO:0065007 biological regulation	-2.84067	4.181409	4.165431	582	7	8.641975	3.122032	134|351|2099|2147|3558|3577|5294	ADORA1|APP|ESR1|F2|IL2|CXCR1|PIK3CG	-1.61921	38	0	0	-7.2588	41.71852
-2.84037	GO:1903131	mononuclear cell differentiation	19_GO:0002376 immune system process	-2.84037	4.89654	4.352492	426	6	7.407407	2.909904	558|1021|2322|3558|5295|6850	AXL|CDK6|FLT3|IL2|PIK3R1|SYK	-1.61921	25	0	0	-9.48567	22.67311
-2.83744	GO:0007259	receptor signaling pathway via JAK-STAT	19_GO:0023052 signaling	-2.83744	8.228505	5.062242	169	4	4.938272	2.407398	1545|2147|2322|3558	CYP1B1|F2|FLT3|IL2	-1.61673	78	1	1	-2.83744	8.228505
-2.81871	GO:0046890	regulation of lipid biosynthetic process	19_GO:0050789 regulation of biological process	-2.81871	8.132265	5.024492	171	4	4.938272	2.407398	207|5743|8644|10062	AKT1|PTGS2|AKR1C3|NR1H3	-1.59845	19	0	0	-10.8808	39.73192
-2.80995	GO:0060420	regulation of heart growth	19_GO:0040007 growth	-2.80995	13.20206	5.833119	79	3	3.703704	2.098362	891|983|5292	CCNB1|CDK1|PIM1	-1.59107	35	0	0	-7.56218	38.62826
-2.80995	GO:0034637	cellular carbohydrate biosynthetic process	19_GO:0008152 metabolic process	-2.80995	13.20206	5.833119	79	3	3.703704	2.098362	207|2932|3643	AKT1|GSK3B|INSR	-1.59107	43	0	0	-6.76848	45.34622

-2.80995	GO:1901184	regulation of ERBB signaling pathway	19_GO:0023052 signaling	-2.80995	13.20206	5.833119	79	3	3.703704	2.098362	134|1956|4318	ADORA1|EGFR|MMP9	-1.59107	61	0	0	-5.47166	27.44639
-2.79424	GO:0032204	regulation of telomere maintenance	19_GO:0050789 regulation of biological process	-2.79424	13.03704	5.790637	80	3	3.703704	2.098362	142|6714|9212	PARP1|SRC|AURKB	-1.57673	40	0	0	-7.12867	115.8848
-2.79424	GO:0021766	hippocampus development	19_GO:0032502 developmental process	-2.79424	13.03704	5.790637	80	3	3.703704	2.098362	238|1021|2932	ALK|CDK6|GSK3B	-1.57673	52	0	0	-6.26187	13.03704
-2.79424	GO:0048145	regulation of fibroblast proliferation	19_GO:0050789 regulation of biological process	-2.79424	13.03704	5.790637	80	3	3.703704	2.098362	891|1021|2099	CCNB1|CDK6|ESR1	-1.57673	75	0	0	-3.44225	21.7284
-2.79363	GO:0010948	negative regulation of cell cycle process	19_GO:0048519 negative regulation of biological process	-2.79363	5.912488	4.547767	294	5	6.17284	2.674018	891|983|1017|5347|9212	CCNB1|CDK1|CDK2|PLK1|AURKB	-1.57657	34	0	0	-7.56608	35.65685
-2.78608	GO:0007507	heart development	19_GO:0032502 developmental process	-2.78608	4.090051	4.091715	595	7	8.641975	3.122032	891|983|1956|3643|5292|5467|5747	CCNB1|CDK1|EGFR|INSR|PIM1|PPARD|PTK2	-1.56948	35	0	0	-7.56218	38.62826

-2.78195	GO:0043409	negative regulation of MAPK cascade	19_GO:0048519 negative regulation of biological process	-2.78195	7.946384	4.950801	175	4	4.938272	2.407398	207|3156|3480|5770	AKT1|HMGCR|IGF1R|PTPN1	-1.56625	17	0	0	-11.2655	11.58848
-2.78195	GO:1905475	regulation of protein localization to membrane	19_GO:0051179 localization	-2.78195	7.946384	4.950801	175	4	4.938272	2.407398	207|367|1956|5295	AKT1|AR|EGFR|PIK3R1	-1.56625	54	0	0	-6.06931	37.58425
-2.77875	GO:0071260	cellular response to mechanical stimulus	19_GO:0050896 response to stimulus	-2.77875	12.87609	5.748908	81	3	3.703704	2.098362	207|1956|5743	AKT1|EGFR|PTGS2	-1.56576	19	0	0	-10.8808	39.73192
-2.77875	GO:2000106	regulation of leukocyte apoptotic process	19_GO:0050789 regulation of biological process	-2.77875	12.87609	5.748908	81	3	3.703704	2.098362	558|3558|9212	AXL|IL2|AURKB	-1.56576	25	0	0	-9.48567	22.67311
-2.77875	GO:0044773	mitotic DNA damage checkpoint signaling	19_GO:0048519 negative regulation of biological process	-2.77875	12.87609	5.748908	81	3	3.703704	2.098362	983|1017|5347	CDK1|CDK2|PLK1	-1.56576	34	0	0	-7.56608	35.65685
-2.77875	GO:0010833	telomere maintenance via telomere lengthening	19_GO:0008152 metabolic process	-2.77875	12.87609	5.748908	81	3	3.703704	2.098362	142|6714|9212	PARP1|SRC|AURKB	-1.56576	40	0	0	-7.12867	115.8848

-2.77875	GO:0001960	negative regulation of cytokine-mediated signaling pathway	19_GO:0048519 negative regulation of biological process	-2.77875	12.87609	5.748908	81	3	3.703704	2.098362	383|4321|10062	ARG1|MMP12|NR1H3	-1.56576	55	0	0	-5.98599	20.05698
-2.77875	GO:0048144	fibroblast proliferation	19_GO:0009987 cellular process	-2.77875	12.87609	5.748908	81	3	3.703704	2.098362	891|1021|2099	CCNB1|CDK6|ESR1	-1.56576	75	0	0	-3.44225	21.7284
-2.7729	GO:0052126	movement in host environment	19_GO:0044419 biological process involved in interspecies interaction between organisms	-2.7729	7.901235	4.932743	176	4	4.938272	2.407398	558|983|1956|6714	AXL|CDK1|EGFR|SRC	-1.56037	57	0	0	-5.76668	22.87199
-2.76392	GO:0043534	blood vessel endothelial cell migration	19_GO:0040011 locomotion	-2.76392	7.856595	4.914828	177	4	4.938272	2.407398	207|239|3791|5743	AKT1|ALOX12|KDR|PTGS2	-1.55227	19	0	0	-10.8808	39.73192
-2.76345	GO:0046889	positive regulation of lipid biosynthetic process	19_GO:0048518 positive regulation of biological process	-2.76345	12.71906	5.707908	82	3	3.703704	2.098362	207|5743|10062	AKT1|PTGS2|NR1H3	-1.55227	19	0	0	-10.8808	39.73192
-2.76345	GO:0060968	regulation of gene silencing	19_GO:0048519 negative regulation of biological process	-2.76345	12.71906	5.707908	82	3	3.703704	2.098362	1017|1956|2099	CDK2|EGFR|ESR1	-1.55227	61	0	0	-5.47166	27.44639

-2.73729	GO:0032640	tumor necrosis factor production	19_GO:0032501 multicellular organismal process	-2.73729	7.725652	4.861912	180	4	4.938272	2.407398	351|558|5295|6850	APP|AXL|PIK3R1|SYK	-1.52745	44	0	0	-6.70994	13.24397
-2.73729	GO:0032680	regulation of tumor necrosis factor production	19_GO:0032501 multicellular organismal process	-2.73729	7.725652	4.861912	180	4	4.938272	2.407398	351|558|5295|6850	APP|AXL|PIK3R1|SYK	-1.52745	44	0	0	-6.70994	13.24397
-2.73729	GO:0097696	receptor signaling pathway via STAT	19_GO:0023052 signaling	-2.73729	7.725652	4.861912	180	4	4.938272	2.407398	1545|2147|2322|3558	CYP1B1|F2|FLT3|IL2	-1.52745	78	0	0	-2.83744	8.228505
-2.73346	GO:0032370	positive regulation of lipid transport	19_GO:0048518 positive regulation of biological process	-2.73346	12.41623	5.628801	84	3	3.703704	2.098362	1588|9536|10062	CYP19A1|PTGES|NR1H3	-1.52452	33	0	0	-8.21676	32.59259
-2.73346	GO:0006112	energy reserve metabolic process	19_GO:0008152 metabolic process	-2.73346	12.41623	5.628801	84	3	3.703704	2.098362	207|2932|3643	AKT1|GSK3B|INSR	-1.52452	43	0	0	-6.76848	45.34622
-2.71875	GO:0032092	positive regulation of protein binding	19_GO:0065007 biological regulation	-2.71875	12.27015	5.589072	85	3	3.703704	2.098362	351|2932|4318	APP|GSK3B|MMP9	-1.5107	21	0	0	-9.98291	27.26701
-2.718	GO:0044774	mitotic DNA integrity checkpoint signaling	19_GO:0048519 negative	-2.718	12.27015	5.5	85	3	3.703704	2.098362	983|1017|5347	CDK1|CDK2|PLK1	-1.5107	34	0	0	-7.5	35.65685

<table>
<tr><td>75</td><td></td><td></td><td>regulation of biological process</td><td>75</td><td></td><td>89072</td><td></td><td></td><td></td><td></td><td></td><td></td><td></td><td></td><td></td><td></td><td>6608</td><td></td></tr>
<tr><td>-2.71777</td><td>GO:0006650</td><td>glycerophospholipid metabolic process</td><td>19_GO:0008152 metabolic process</td><td>-2.71777</td><td>5.680626</td><td>4.421618</td><td>306</td><td>5</td><td>6.17284</td><td>2.674018</td><td>43|246|5294|5295|10062</td><td>ACHE|ALOX15|PIK3CG|PIK3R1|NR1H3</td><td>-1.51016</td><td>71</td><td>0</td><td>0</td><td>-3.73717</td><td>6.897903</td></tr>
<tr><td>-2.71116</td><td>GO:0002819</td><td>regulation of adaptive immune response</td><td>19_GO:0002376 immune system process</td><td>-2.71116</td><td>7.599002</td><td>4.810204</td><td>183</td><td>4</td><td>4.938272</td><td>2.407398</td><td>196|246|383|3558</td><td>AHR|ALOX15|ARG1|IL2</td><td>-1.50399</td><td>56</td><td>0</td><td>0</td><td>-5.89598</td><td>130.3704</td></tr>
<tr><td>-2.70423</td><td>GO:0060761</td><td>negative regulation of response to cytokine stimulus</td><td>19_GO:0048519 negative regulation of biological process</td><td>-2.70423</td><td>12.12748</td><td>5.550781</td><td>86</td><td>3</td><td>3.703704</td><td>2.098362</td><td>383|4321|10062</td><td>ARG1|MMP12|NR1H3</td><td>-1.49751</td><td>55</td><td>0</td><td>0</td><td>-5.98599</td><td>20.05698</td></tr>
<tr><td>-2.694</td><td>GO:0043433</td><td>negative regulation of DNA-binding transcription factor activity</td><td>19_GO:0050789 regulation of biological process</td><td>-2.694</td><td>7.51685</td><td>4.77638</td><td>185</td><td>4</td><td>4.938272</td><td>2.407398</td><td>140|1545|2099|5292</td><td>ADORA3|CYP1B1|ESR1|PIM1</td><td>-1.48772</td><td>79</td><td>1</td><td>1</td><td>-2.694</td><td>7.51685</td></tr>
<tr><td>-2.68988</td><td>GO:0048041</td><td>focal adhesion assembly</td><td>19_GO:0022610 biological adhesion</td><td>-2.68988</td><td>11.98808</td><td>5.513119</td><td>87</td><td>3</td><td>3.703704</td><td>2.098362</td><td>3791|5747|6714</td><td>KDR|PTK2|SRC</td><td>-1.48449</td><td>41</td><td>0</td><td>0</td><td>-7.04885</td><td>38.62826</td></tr>
<tr><td>-2.68988</td><td>GO:1903578</td><td>regulation of ATP metabolic process</td><td>19_GO:0050789 regulation of biological process</td><td>-2.68988</td><td>11.98808</td><td>5.51</td><td>87</td><td>3</td><td>3.703704</td><td>2.098362</td><td>142|351|3643</td><td>PARP1|APP|INSR</td><td>-1.48449</td><td>45</td><td>0</td><td>0</td><td>-6.5686</td><td>52.14815</td></tr>
</table>

						3119											7	
-2.68549	GO:0071706	tumor necrosis factor superfamily cytokine production	19_GO:0032501 multicellular organismal process	-2.68549	7.476437	4.759657	186	4	4.938272	2.407398	351|558|5295|6850	APP|AXL|PIK3R1|SYK	-1.48143	44	0	0	-6.70994	13.24397
-2.68549	GO:1903555	regulation of tumor necrosis factor superfamily cytokine production	19_GO:0032501 multicellular organismal process	-2.68549	7.476437	4.759657	186	4	4.938272	2.407398	351|558|5295|6850	APP|AXL|PIK3R1|SYK	-1.48143	44	0	0	-6.70994	13.24397
-2.68549	GO:0008217	regulation of blood pressure	19_GO:0032501 multicellular organismal process	-2.68549	7.476437	4.759657	186	4	4.938272	2.407398	134|367|4843|5743	ADORA1|AR|NOS2|PTGS2	-1.48143	64	0	0	-4.94984	45.34622
-2.67704	GO:0061136	regulation of proteasomal protein catabolic process	19_GO:0050789 regulation of biological process	-2.67704	7.436456	4.743058	187	4	4.938272	2.407398	207|1017|2932|5347	AKT1|CDK2|GSK3B|PLK1	-1.47386	43	0	0	-6.76848	45.34622
-2.67704	GO:0002695	negative regulation of leukocyte activation	19_GO:0002376 immune system process	-2.67704	7.436456	4.743058	187	4	4.938272	2.407398	383|558|3558|10062	ARG1|AXL|IL2|NR1H3	-1.47386	55	0	0	-5.98599	20.05698
-2.66864	GO:0002040	sprouting angiogenesis	19_GO:0032502 developmental process	-2.66864	7.3969	4.72	188	4	4.938272	2.407398	207|240|3791|5743	AKT1|ALOX5|KDR|PTGS2	-1.4659	19	0	0	-10.880	39.73192

						6581											8	
-2.66172	GO:0014910	regulation of smooth muscle cell migration	19_GO:0040011 locomotion	-2.66172	11.71868	5.439611	89	3	3.703704	2.098362	328|5467|6714	APEX1|PPARD|SRC	-1.45941	76	0	0	-3.32709	11.71868
-2.6394	GO:0071103	DNA conformation change	19_GO:0009987 cellular process	-2.6394	5.449127	4.29228	319	5	6.17284	2.674018	891|983|5347|7150|7153	CCNB1|CDK1|PLK1|TOP1|TOP2A	-1.43797	34	0	0	-7.56608	35.65685
-2.6394	GO:0007204	positive regulation of cytosolic calcium ion concentration	19_GO:0065007 biological regulation	-2.6394	5.449127	4.29228	319	5	6.17284	2.674018	2099|2147|3558|3577|5294	ESR1|F2|IL2|CXCR1|PIK3CG	-1.43797	38	0	0	-7.2588	41.71852
-2.63553	GO:0001906	cell killing	19_GO:0009987 cellular process	-2.63553	7.242798	4.661863	192	4	4.938272	2.407398	383|2147|4843|6850	ARG1|F2|NOS2|SYK	-1.43455	56	0	0	-5.89598	130.3704
-2.63422	GO:0034109	homotypic cell-cell adhesion	19_GO:0022610 biological adhesion	-2.63422	11.46113	5.368416	91	3	3.703704	2.098362	239|5294|6850	ALOX12|PIK3CG|SYK	-1.43411	9	0	0	-13.6942	26.07407
-2.63422	GO:0032436	positive regulation of proteasomal ubiquitin-dependent protein catabolic process	19_GO:0048518 positive regulation of biological process	-2.63422	11.46113	5.3684	91	3	3.703704	2.098362	207|2932|5347	AKT1|GSK3B|PLK1	-1.43411	43	0	0	-6.76848	45.34622

						16												
-2.62072	GO:0035249	synaptic transmission, glutamatergic	19_GO:0023052 signaling	-2.62072	11.33655	5.333647	92	3	3.703704	2.098362	134|1956|5743	ADORA1|EGFR|PTGS2	-1.42148	61	0	0	-5.47166	27.44639
-2.62072	GO:0060191	regulation of lipase activity	19_GO:0065007 biological regulation	-2.62072	11.33655	5.333647	92	3	3.703704	2.098362	1956|2099|10062	EGFR|ESR1|NR1H3	-1.42148	61	0	0	-5.47166	27.44639
-2.60737	GO:0032755	positive regulation of interleukin-6 production	19_GO:0048518 positive regulation of biological process	-2.60737	11.21466	5.29941	93	3	3.703704	2.098362	351|4843|6850	APP|NOS2|SYK	-1.40857	44	0	0	-6.70994	13.24397
-2.60319	GO:1901605	alpha-amino acid metabolic process	19_GO:0008152 metabolic process	-2.60319	7.094986	4.598979	196	4	4.938272	2.407398	383|4843|4953|7299	ARG1|NOS2|ODC1|TYR	-1.40483	56	0	0	-5.89598	130.3704
-2.59873	GO:0030111	regulation of Wnt signaling pathway	19_GO:0023052 signaling	-2.59873	5.332121	4.225537	326	5	6.17284	2.674018	351|1457|1956|2932|6714	APP|CSNK2A1|EGFR|GSK3B|SRC	-1.4008	51	0	0	-6.31611	22.67311
-2.59418	GO:0002042	cell migration involved in sprouting angiogenesis	19_GO:0040011 locomotion	-2.59418	11.09535	5.26569	94	3	3.703704	2.098362	207|3791|5743	AKT1|KDR|PTGS2	-1.39669	19	0	0	-10.8808	39.73192

						1												
-2.58114	GO:0007044	cell-substrate junction assembly	19_GO:0009987 cellular process	-2.58114	10.97856	5.232477	95	3	3.703704	2.098362	3791|5747|6714	KDR|PTK2|SRC	-1.38495	41	0	0	-7.04885	38.62826
-2.58114	GO:0032091	negative regulation of protein binding	19_GO:0065007 biological regulation	-2.58114	10.97856	5.232477	95	3	3.703704	2.098362	207|2932|9212	AKT1|GSK3B|AURKB	-1.38495	43	0	0	-6.76848	45.34622
-2.58114	GO:0008585	female gonad development	19_GO:0022414 reproductive process	-2.58114	10.97856	5.232477	95	3	3.703704	2.098362	1588|2099|6714	CYP19A1|ESR1|SRC	-1.38495	60	0	0	-5.48956	45.34622
-2.57594	GO:0050863	regulation of T cell activation	19_GO:0002376 immune system process	-2.57594	5.26749	4.188255	330	5	6.17284	2.674018	207|383|3558|6714|6850	AKT1|ARG1|IL2|SRC|SYK	-1.38018	25	0	0	-9.48567	22.67311
-2.56824	GO:0055017	cardiac muscle tissue growth	19_GO:0040007 growth	-2.56824	10.8642	5.199755	96	3	3.703704	2.098362	891|983|5292	CCNB1|CDK1|PIM1	-1.37335	35	0	0	-7.56218	38.62826
-2.56824	GO:0042632	cholesterol homeostasis	19_GO:0065007 biological regulation	-2.56824	10.8642	5.19975	96	3	3.703704	2.098362	1645|3156|10062	AKR1C1|HMGCR|NR1H3	-1.37335	74	0	0	-3.61486	24.83245

						5												
-2.56379	GO:0042098	T cell proliferation	19_GO:0002376 immune system process	-2.56379	6.918494	4.522814	201	4	4.938272	2.407398	383|3558|5294|6850	ARG1|IL2|PIK3CG|SYK	-1.36933	56	0	0	-5.89598	130.3704
-2.5629	GO:0042110	T cell activation	19_GO:0002376 immune system process	-2.5629	4.309764	3.944831	484	6	7.407407	2.909904	207|383|3558|5294|6714|6850	AKT1|ARG1|IL2|PIK3CG|SRC|SYK	-1.36887	25	0	0	-9.48567	22.67311
-2.55604	GO:0032388	positive regulation of intracellular transport	19_GO:0048518 positive regulation of biological process	-2.55604	6.884244	4.507893	202	4	4.938272	2.407398	983|2932|5295|5743	CDK1|GSK3B|PIK3R1|PTGS2	-1.36404	16	0	0	-11.5558	31.96821
-2.55549	GO:0043255	regulation of carbohydrate biosynthetic process	19_GO:0050789 regulation of biological process	-2.55549	10.7522	5.167512	97	3	3.703704	2.098362	207|2932|3643	AKT1|GSK3B|INSR	-1.36404	43	0	0	-6.76848	45.34622
-2.55549	GO:0044264	cellular polysaccharide metabolic process	19_GO:0008152 metabolic process	-2.55549	10.7522	5.167512	97	3	3.703704	2.098362	207|2932|3643	AKT1|GSK3B|INSR	-1.36404	43	0	0	-6.76848	45.34622
-2.55549	GO:0120162	positive regulation of cold-induced thermogenesis	19_GO:0048518 positive regulation of biological process	-2.55549	10.7522	5.16751	97	3	3.703704	2.098362	43|3480|6850	ACHE|IGF1R|SYK	-1.36404	53	0	0	-6.14202	13.98609

						2												
-2.55549	GO:0055092	sterol homeostasis	19_GO:0065007 biological regulation	-2.55549	10.7522	5.167512	97	3	3.703704	2.098362	1645|3156|10062	AKR1C1|HMGCR|NR1H3	-1.36404	74	0	0	-3.61486	24.83245
-2.55549	GO:0014909	smooth muscle cell migration	19_GO:0040011 locomotion	-2.55549	10.7522	5.167512	97	3	3.703704	2.098362	328|5467|6714	APEX1|PPARD|SRC	-1.36404	76	0	0	-3.32709	11.71868
-2.55348	GO:0042113	B cell activation	19_GO:0002376 immune system process	-2.55348	5.204406	4.151574	334	5	6.17284	2.674018	196|2322|3558|5295|6850	AHR|FLT3|IL2|PIK3R1|SYK	-1.36246	25	0	0	-9.48567	22.67311
-2.54792	GO:1990778	protein localization to cell periphery	19_GO:0051179 localization	-2.54792	5.18887	4.142496	335	5	6.17284	2.674018	207|367|1956|5295|5347	AKT1|AR|EGFR|PIK3R1|PLK1	-1.35732	54	0	0	-6.06931	37.58425
-2.54288	GO:0006805	xenobiotic metabolic process	19_GO:0050896 response to stimulus	-2.54288	10.64248	5.135737	98	3	3.703704	2.098362	196|1545|1645	AHR|CYP1B1|AKR1C1	-1.35272	12	0	0	-13.14	260.7407
-2.54068	GO:1901888	regulation of cell junction assembly	19_GO:0050789 regulation of biological process	-2.54068	6.816751	4.47835	204	4	4.938272	2.407398	351|3791|5747|6714	APP|KDR|PTK2|SRC	-1.35094	41	0	0	-7.04885	38.62826

						4												
-2.53306	GO:0000819	sister chromatid segregation	19_GO:0009987 cellular process	-2.53306	6.783499	4.463732	205	4	4.938272	2.407398	891|5347|7153|9212	CCNB1|PLK1|TOP2A|AURKB	-1.34375	34	0	0	-7.56608	35.65685
-2.53041	GO:0060291	long-term synaptic potentiation	19_GO:0048518 positive regulation of biological process	-2.53041	10.53498	5.104416	99	3	3.703704	2.098362	134|351|2932	ADORA1|APP|GSK3B	-1.34153	51	0	0	-6.31611	22.67311
-2.51808	GO:0046545	development of primary female sexual characteristics	19_GO:0022414 reproductive process	-2.51808	10.42963	5.07354	100	3	3.703704	2.098362	1588|2099|6714	CYP19A1|ESR1|SRC	-1.33033	60	0	0	-5.48956	45.34622
-2.51794	GO:0008016	regulation of heart contraction	19_GO:0032501 multicellular organismal process	-2.51794	6.717958	4.434781	207	4	4.938272	2.407398	134|140|3558|5294	ADORA1|ADORA3|IL2|PIK3CG	-1.33033	38	0	0	-7.2588	41.71852
-2.51794	GO:1902905	positive regulation of supramolecular fiber organization	19_GO:0048518 positive regulation of biological process	-2.51794	6.717958	4.434781	207	4	4.938272	2.407398	246|351|4137|4233	ALOX15|APP|MAPT|MET	-1.33033	50	0	0	-6.31767	11.54039
-2.51493	GO:0002697	regulation of immune effector process	19_GO:0002376 immune system process	-2.51493	5.097571	4.088775	341	5	6.17284	2.674018	196|383|3558|4843|6850	AHR|ARG1|IL2|NOS2|SYK	-1.32774	56	0	0	-5.89598	130.3704

-2.50588	GO:0098869	cellular oxidant detoxification	19_GO:0098754 detoxification	-2.50588	10.32637	5.0430977	101	3	3.703704	2.098362	4353|5743|9536	MPO|PTGS2|PTGES	-1.31954	15	0	0	-12.2065	35.47493
-2.50588	GO:0045807	positive regulation of endocytosis	19_GO:0048518 positive regulation of biological process	-2.50588	10.32637	5.0430977	101	3	3.703704	2.098362	558|3643|6850	AXL|INSR|SYK	-1.31954	44	0	0	-6.70994	13.24397
-2.4938	GO:1902106	negative regulation of leukocyte differentiation	19_GO:0002376 immune system process	-2.4938	10.22513	5.0130777	102	3	3.703704	2.098362	1021|3558|5295	CDK6|IL2|PIK3R1	-1.30916	25	0	0	-9.48567	22.67311
-2.4938	GO:0032760	positive regulation of tumor necrosis factor production	19_GO:0048518 positive regulation of biological process	-2.4938	10.22513	5.0130777	102	3	3.703704	2.098362	351|5295|6850	APP|PIK3R1|SYK	-1.30916	44	0	0	-6.70994	13.24397
-2.4938	GO:0031341	regulation of cell killing	19_GO:0050789 regulation of biological process	-2.4938	10.22513	5.0130777	102	3	3.703704	2.098362	383|4843|6850	ARG1|NOS2|SYK	-1.30916	56	0	0	-5.89598	130.3704
-2.4938	GO:0062014	negative regulation of small molecule metabolic process	19_GO:0048519 negative regulation of biological process	-2.4938	10.22513	5.0130777	102	3	3.703704	2.098362	142|207|8644	PARP1|AKT1|AKR1C3	-1.30916	63	0	0	-5.22413	23.17695

-2.48821	GO:0045637	regulation of myeloid cell differentiation	19_GO:0002376 immune system process	-2.48821	6.590603	4.378013	211	4	4.938272	2.407398	760|1021|2101|5295	CA2|CDK6|ESRRA|PIK3R1	-1.30441	37	0	0	-7.31428	16.2963
-2.48821	GO:0046474	glycerophospholipid biosynthetic process	19_GO:0008152 metabolic process	-2.48821	6.590603	4.378013	211	4	4.938272	2.407398	43|246|5294|5295	ACHE|ALOX15|PIK3CG|PIK3R1	-1.30441	71	0	0	-3.73717	6.897903
-2.48186	GO:0071466	cellular response to xenobiotic stimulus	19_GO:0050896 response to stimulus	-2.48186	10.12585	4.98347	103	3	3.703704	2.098362	196|1545|1645	AHR|CYP1B1|AKR1C1	-1.29974	12	0	0	-13.14	260.7407
-2.48186	GO:0090277	positive regulation of peptide hormone secretion	19_GO:0048518 positive regulation of biological process	-2.48186	10.12585	4.98347	103	3	3.703704	2.098362	1080|1956|5467	CFTR|EGFR|PPARD	-1.29974	28	0	0	-8.92043	22.1907
-2.48186	GO:0030593	neutrophil chemotaxis	19_GO:0040011 locomotion	-2.48186	10.12585	4.98347	103	3	3.703704	2.098362	3577|5294|6850	CXCR1|PIK3CG|SYK	-1.29974	42	0	0	-6.93111	31.60494
-2.48186	GO:0019233	sensory perception of pain	19_GO:0032501 multicellular organismal process	-2.48186	10.12585	4.98347	103	3	3.703704	2.098362	134|5743|9536	ADORA1|PTGS2|PTGES	-1.29974	64	0	0	-4.94984	45.34622
-2.48087	GO:0002685	regulation of leukocyte migration	19_GO:0040011 locomotion	-2.48087	6.559515	4.364	212	4	4.938272	2.407398	134|207|351|5747	ADORA1|AKT1|APP|PTK2	-1.29917	10	0	0	-13.3451	41.71852

<table>
<tr><td></td><td></td><td></td><td></td><td></td><td></td><td>051</td><td></td><td></td><td></td><td></td><td></td><td></td><td></td><td></td><td></td><td></td><td></td><td></td></tr>
<tr><td>-2.47358</td><td>GO:0006469</td><td>negative regulation of protein kinase activity</td><td>19_GO:0048519 negative regulation of biological process</td><td>-2.47358</td><td>6.52872</td><td>4.350178</td><td>213</td><td>4</td><td>4.938272</td><td>2.407398</td><td>207|3156|5347|5770</td><td>AKT1|HMGCR|PLK1|PTPN1</td><td>-1.29271</td><td>17</td><td>0</td><td>0</td><td>-11.2655</td><td>11.58848</td></tr>
<tr><td>-2.47358</td><td>GO:0009749</td><td>response to glucose</td><td>19_GO:0050896 response to stimulus</td><td>-2.47358</td><td>6.52872</td><td>4.350178</td><td>213</td><td>4</td><td>4.938272</td><td>2.407398</td><td>1080|3156|3480|5467</td><td>CFTR|HMGCR|IGF1R|PPARD</td><td>-1.29271</td><td>28</td><td>0</td><td>0</td><td>-8.92043</td><td>22.1907</td></tr>
<tr><td>-2.47004</td><td>GO:0045621</td><td>positive regulation of lymphocyte differentiation</td><td>19_GO:0002376 immune system process</td><td>-2.47004</td><td>10.02849</td><td>4.954265</td><td>104</td><td>3</td><td>3.703704</td><td>2.098362</td><td>558|3558|6850</td><td>AXL|IL2|SYK</td><td>-1.29001</td><td>25</td><td>0</td><td>0</td><td>-9.48567</td><td>22.67311</td></tr>
<tr><td>-2.47004</td><td>GO:0060419</td><td>heart growth</td><td>19_GO:0040007 growth</td><td>-2.47004</td><td>10.02849</td><td>4.954265</td><td>104</td><td>3</td><td>3.703704</td><td>2.098362</td><td>891|983|5292</td><td>CCNB1|CDK1|PIM1</td><td>-1.29001</td><td>35</td><td>0</td><td>0</td><td>-7.56218</td><td>38.62826</td></tr>
<tr><td>-2.45553</td><td>GO:0007005</td><td>mitochondrion organization</td><td>19_GO:0009987 cellular process</td><td>-2.45553</td><td>4.098086</td><td>3.788413</td><td>509</td><td>6</td><td>7.407407</td><td>2.909904</td><td>142|207|2932|3791|4137|4318</td><td>PARP1|AKT1|GSK3B|KDR|MAPT|MMP9</td><td>-1.27592</td><td>43</td><td>0</td><td>0</td><td>-6.76848</td><td>45.34622</td></tr>
<tr><td>-2.45192</td><td>GO:0050870</td><td>positive regulation of T cell activation</td><td>19_GO:0022610 biological adhesion</td><td>-2.45192</td><td>6.438043</td><td>4.309</td><td>216</td><td>4</td><td>4.938272</td><td>2.407398</td><td>207|3558|6714|6850</td><td>AKT1|IL2|SRC|SYK</td><td>-1.27273</td><td>25</td><td>0</td><td>0</td><td>-9.48567</td><td>22.67311</td></tr>
</table>

						09												
-2.44677	GO:1903707	negative regulation of hemopoiesis	19_GO:0002376 immune system process	-2.44677	9.839273	4.897026	106	3	3.703704	2.098362	1021|3558|5295	CDK6|IL2|PIK3R1	-1.26924	25	0	0	-9.48567	22.67311
-2.44677	GO:1903557	positive regulation of tumor necrosis factor superfamily cytokine production	19_GO:0048518 positive regulation of biological process	-2.44677	9.839273	4.897026	106	3	3.703704	2.098362	351|5295|6850	APP|PIK3R1|SYK	-1.26924	44	0	0	-6.70994	13.24397
-2.44677	GO:1905477	positive regulation of protein localization to membrane	19_GO:0048518 positive regulation of biological process	-2.44677	9.839273	4.897026	106	3	3.703704	2.098362	207|1956|5295	AKT1|EGFR|PIK3R1	-1.26924	54	0	0	-6.06931	37.58425
-2.44677	GO:0006766	vitamin metabolic process	19_GO:0008152 metabolic process	-2.44677	9.839273	4.897026	106	3	3.703704	2.098362	4363|5467|8644	ABCC1|PPARD|AKR1C3	-1.26924	65	0	0	-4.7415	57.94239
-2.43531	GO:0008637	apoptotic mitochondrial changes	19_GO:0009987 cellular process	-2.43531	9.747317	4.868974	107	3	3.703704	2.098362	207|2932|4318	AKT1|GSK3B|MMP9	-1.25944	43	0	0	-6.76848	45.34622
-2.43531	GO:0021761	limbic system development	19_GO:0032502 developmental process	-2.43531	9.747317	4.8689	107	3	3.703704	2.098362	238|1021|2932	ALK|CDK6|GSK3B	-1.25944	52	0	0	-6.26187	13.03704

						74												
-2.43531	GO:0071868	cellular response to monoamine stimulus	19_GO:0050896 response to stimulus	-2.43531	9.747317	4.868974	107	3	3.703704	2.098362	238|351|2932	ALK|APP|GSK3B	-1.25944	52	0	0	-6.26187	13.03704
-2.43531	GO:0071870	cellular response to catecholamine stimulus	19_GO:0050896 response to stimulus	-2.43531	9.747317	4.868974	107	3	3.703704	2.098362	238|351|2932	ALK|APP|GSK3B	-1.25944	52	0	0	-6.26187	13.03704
-2.42397	GO:0030038	contractile actin filament bundle assembly	19_GO:0009987 cellular process	-2.42397	9.657064	4.841288	108	3	3.703704	2.098362	4233|5295|6714	MET|PIK3R1|SRC	-1.24934	41	0	0	-7.04885	38.62826
-2.42397	GO:0043149	stress fiber assembly	19_GO:0009987 cellular process	-2.42397	9.657064	4.841288	108	3	3.703704	2.098362	4233|5295|6714	MET|PIK3R1|SRC	-1.24934	41	0	0	-7.04885	38.62826
-2.42397	GO:0005976	polysaccharide metabolic process	19_GO:0008152 metabolic process	-2.42397	9.657064	4.841288	108	3	3.703704	2.098362	207|2932|3643	AKT1|GSK3B|INSR	-1.24934	43	0	0	-6.76848	45.34622
-2.41274	GO:0007229	integrin-mediated signaling pathway	19_GO:0023052 signaling	-2.41274	9.568468	4.8139	109	3	3.703704	2.098362	5747|6714|6850	PTK2|SRC|SYK	-1.23852	41	0	0	-7.04885	38.62826

61

-2.40466	GO:0051251	positive regulation of lymphocyte activation	19_GO:0002376 immune system process	-2.40466	4.801855	3.91024	362	5	6.17284	2.674018	207|558|3558|6714|6850	AKT1|AXL|IL2|SRC|SYK	-1.23085	25	0	0	-9.48567	22.67311
-2.40269	GO:0097529	myeloid leukocyte migration	19_GO:0040011 locomotion	-2.40269	6.235952	4.216174	223	4	4.938272	2.407398	3577|5294|5747|6850	CXCR1|PIK3CG|PTK2|SYK	-1.23028	42	0	0	-6.93111	31.60494
-2.40162	GO:0009410	response to xenobiotic stimulus	19_GO:0050896 response to stimulus	-2.40162	9.481481	4.786984	110	3	3.703704	2.098362	196|1545|1645	AHR|CYP1B1|AKR1C1	-1.23028	12	0	0	-13.14	260.7407
-2.40162	GO:1905954	positive regulation of lipid localization	19_GO:0048518 positive regulation of biological process	-2.40162	9.481481	4.786984	110	3	3.703704	2.098362	1588|9536|10062	CYP19A1|PTGES|NR1H3	-1.23028	33	0	0	-8.21676	32.59259
-2.40162	GO:0002698	negative regulation of immune effector process	19_GO:0002376 immune system process	-2.40162	9.481481	4.786984	110	3	3.703704	2.098362	196|383|3558	AHR|ARG1|IL2	-1.23028	56	0	0	-5.89598	130.3704
-2.40162	GO:0006939	smooth muscle contraction	19_GO:0032501 multicellular organismal process	-2.40162	9.481481	4.78698	110	3	3.703704	2.098362	134|4638|5743	ADORA1|MYLK|PTGS2	-1.23028	64	0	0	-4.94984	45.34622

						4												
-2.40162	GO:0014812	muscle cell migration	19_GO:0040011 locomotion	-2.40162	9.481481	4.786984	110	3	3.703704	2.098362	328|5467|6714	APEX1|PPARD|SRC	-1.23028	76	0	0	-3.32709	11.71868
-2.39062	GO:0001938	positive regulation of endothelial cell proliferation	19_GO:0048518 positive regulation of biological process	-2.39062	9.396063	4.760349	111	3	3.703704	2.098362	207|383|3791	AKT1|ARG1|KDR	-1.2205	39	0	0	-7.16624	9.980507
-2.39062	GO:0071867	response to monoamine	19_GO:0050896 response to stimulus	-2.39062	9.396063	4.760349	111	3	3.703704	2.098362	238|351|2932	ALK|APP|GSK3B	-1.2205	52	0	0	-6.26187	13.03704
-2.39062	GO:0071869	response to catecholamine	19_GO:0050896 response to stimulus	-2.39062	9.396063	4.760349	111	3	3.703704	2.098362	238|351|2932	ALK|APP|GSK3B	-1.2205	52	0	0	-6.26187	13.03704
-2.38213	GO:0050670	regulation of lymphocyte proliferation	19_GO:0002376 immune system process	-2.38213	6.153174	4.177561	226	4	4.938272	2.407398	196|383|3558|6850	AHR|ARG1|IL2|SYK	-1.21242	56	0	0	-5.89598	130.3704
-2.36958	GO:0009150	purine ribonucleotide metabolic process	19_GO:0008152 metabolic process	-2.36958	4.710763	3.85376	369	5	6.17284	2.674018	142|351|3156|3643|7498	PARP1|APP|HMGCR|INSR|XDH	-1.20028	45	0	0	-6.56867	52.14815

-2.3686	GO:0032944	regulation of mononuclear cell proliferation	19_GO:0050789 regulation of biological process	-2.3686	6.099199	4.1522204	228	4	4.938272	2.407398	196|383|3558|6850	AHR|ARG1|IL2|SYK	-1.1997	56	0	0	-5.89598	130.3704
-2.36188	GO:0006323	DNA packaging	19_GO:0009987 cellular process	-2.36188	6.072565	4.1396638	229	4	4.938272	2.407398	891|983|5347|7153	CCNB1|CDK1|PLK1|TOP2A	-1.1934	34	0	0	-7.56608	35.65685
-2.35823	GO:0071675	regulation of mononuclear cell migration	19_GO:0040011 locomotion	-2.35823	9.148798	4.682425	114	3	3.703704	2.098362	207|351|5747	AKT1|APP|PTK2	-1.19137	10	0	0	-13.3451	41.71852
-2.35823	GO:1901800	positive regulation of proteasomal protein catabolic process	19_GO:0048518 positive regulation of biological process	-2.35823	9.148798	4.682425	114	3	3.703704	2.098362	207|2932|5347	AKT1|GSK3B|PLK1	-1.19137	43	0	0	-6.76848	45.34622
-2.35823	GO:0048259	regulation of receptor-mediated endocytosis	19_GO:0051179 localization	-2.35823	9.148798	4.682425	114	3	3.703704	2.098362	351|3643|6850	APP|INSR|SYK	-1.19137	44	0	0	-6.70994	13.24397
-2.35823	GO:0002244	hematopoietic progenitor cell differentiation	19_GO:0002376 immune system process	-2.35823	9.148798	4.682425	114	3	3.703704	2.098362	2322|3791|7153	FLT3|KDR|TOP2A	-1.19137	66	0	0	-4.22583	21.28496

-2.3552	GO:0045333	cellular respiration	19_GO:0008152 metabolic process	-2.3552	6.046162	4.1271447	230	4	4.938272	2.407398	207|891|983|4843	AKT1|CCNB1|CDK1|NOS2	-1.18874	35	0	0	-7.56218	38.62826
-2.3499	GO:0030098	lymphocyte differentiation	19_GO:0002376 immune system process	-2.3499	4.660246	3.8221118	373	5	6.17284	2.674018	558|2322|3558|5295|6850	AXL|FLT3|IL2|PIK3R1|SYK	-1.18384	25	0	0	-9.48567	22.67311
-2.34765	GO:0000077	DNA damage checkpoint signaling	19_GO:0048519 negative regulation of biological process	-2.34765	9.069243	4.6570887	115	3	3.703704	2.098362	983|1017|5347	CDK1|CDK2|PLK1	-1.18199	34	0	0	-7.56608	35.65685
-2.32677	GO:0072676	lymphocyte migration	19_GO:0040011 locomotion	-2.32677	8.914213	4.6073244	117	3	3.703704	2.098362	207|351|5294	AKT1|APP|PIK3CG	-1.16192	10	0	0	-13.3451	41.71852
-2.32677	GO:0030301	cholesterol transport	19_GO:0051179 localization	-2.32677	8.914213	4.6073244	117	3	3.703704	2.098362	1080|1645|10062	CFTR|AKR1C1|NR1H3	-1.16192	74	0	0	-3.61486	24.83245
-2.32228	GO:0060560	developmental growth involved in morphogenesis	19_GO:0040007 growth	-2.32228	5.91752	4.0657779	235	4	4.938272	2.407398	351|2099|2932|4137	APP|ESR1|GSK3B|MAPT	-1.15784	51	0	0	-6.31611	22.67311

-2.31648	GO:0007613	memory	19_GO:0007610 behavior	-2.31648	8.838669	4.5828887	118	3	3.703704	2.098362	3643|4137|5743	INSR|MAPT|PTGS2	-1.15244	19	0	0	-10.8808	39.73192
-2.31605	GO:1902903	regulation of supramolecular fiber organization	19_GO:0050789 regulation of biological process	-2.31605	4.574399	3.767781	380	5	6.17284	2.674018	246|351|4137|4233|5295	ALOX15|APP|MAPT|MET|PIK3R1	-1.15241	50	0	0	-6.31767	11.54039
-2.30934	GO:0046395	carboxylic acid catabolic process	19_GO:0008152 metabolic process	-2.30934	5.867583	4.041726	237	4	4.938272	2.407398	207|383|4843|5467	AKT1|ARG1|NOS2|PPARD	-1.1465	19	0	0	-10.8808	39.73192
-2.30934	GO:0002699	positive regulation of immune effector process	19_GO:0002376 immune system process	-2.30934	5.867583	4.041726	237	4	4.938272	2.407398	383|3558|4843|6850	ARG1|IL2|NOS2|SYK	-1.1465	56	0	0	-5.89598	130.3704
-2.29652	GO:0032886	regulation of microtubule-based process	19_GO:0050789 regulation of biological process	-2.29652	5.818482	4.017946	239	4	4.938272	2.407398	2932|4137|4233|5347	GSK3B|MAPT|MET|PLK1	-1.13414	50	0	0	-6.31767	11.54039
-2.29617	GO:0032411	positive regulation of transporter activity	19_GO:0048518 positive regulation of biological process	-2.29617	8.691358	4.534871	120	3	3.703704	2.098362	1080|5243|10062	CFTR|ABCB1|NR1H3	-1.13414	27	0	0	-8.92369	24.25495
-	GO:000	ribonucleotide	19_GO:0008152	-	4.503	3.	38	5	6.17	2.674	142|351|3156|	PARP1|APP|HMGCR|INSR|	-1.126	45	0	0	-	52.14

2.28763	9259	metabolic process	metabolic process	2.28763	294	7223	6		284	018	3643|7498	XDH					6.56867	815
-2.28382	GO:0016054	organic acid catabolic process	19_GO:0008152 metabolic process	-2.28382	5.770196	3.994433	241	4	4.938272	2.407398	207|383|4843|5467	AKT1|ARG1|NOS2|PPARD	-1.12259	19	0	0	-10.8808	39.73192
-2.27752	GO:0060047	heart contraction	19_GO:0032501 multicellular organismal process	-2.27752	5.746352	3.982775	242	4	4.938272	2.407398	134|140|3558|5294	ADORA1|ADORA3|IL2|PIK3CG	-1.11669	38	0	0	-7.2588	41.71852
-2.26641	GO:0007052	mitotic spindle organization	19_GO:0009987 cellular process	-2.26641	8.479374	4.464906	123	3	3.703704	2.098362	891|5347|9212	CCNB1|PLK1|AURKB	-1.10717	34	0	0	-7.56608	35.65685
-2.26641	GO:0031570	DNA integrity checkpoint signaling	19_GO:0048519 negative regulation of biological process	-2.26641	8.479374	4.464906	123	3	3.703704	2.098362	983|1017|5347	CDK1|CDK2|PLK1	-1.10717	34	0	0	-7.56608	35.65685
-2.26641	GO:1990266	neutrophil migration	19_GO:0040011 locomotion	-2.26641	8.479374	4.464906	123	3	3.703704	2.098362	3577|5294|6850	CXCR1|PIK3CG|SYK	-1.10717	42	0	0	-6.93111	31.60494
-2.26641	GO:0045446	endothelial cell differentiation	19_GO:0032502 developmental process	-2.26641	8.479374	4.46	123	3	3.703704	2.098362	3791|4233|7498	KDR|MET|XDH	-1.10717	66	0	0	-4.2258	21.28496

						4906											3	
-2.265	GO:0016358	dendrite development	19_GO:0032502 developmental process	-2.265	5.699251	3.959654	244	4	4.938272	2.407398	238|351|2932|3558	ALK|APP|GSK3B|IL2	-1.10616	52	0	0	-6.26187	13.03704
-2.25973	GO:0046486	glycerolipid metabolic process	19_GO:0008152 metabolic process	-2.25973	4.434366	3.677711	392	5	6.17284	2.674018	43|246|5294|5295|10062	ACHE|ALOX15|PIK3CG|PIK3R1|NR1H3	-1.10129	71	0	0	-3.73717	6.897903
-2.25666	GO:0010508	positive regulation of autophagy	19_GO:0048518 positive regulation of biological process	-2.25666	8.410992	4.442111	124	3	3.703704	2.098362	1612|2932|3791	DAPK1|GSK3B|KDR	-1.09901	43	0	0	-6.76848	45.34622
-2.25666	GO:1904019	epithelial cell apoptotic process	19_GO:0009987 cellular process	-2.25666	8.410992	4.442111	124	3	3.703704	2.098362	3791|5294|8644	KDR|PIK3CG|AKR1C3	-1.09901	47	0	0	-6.43293	55.62469
-2.247	GO:0032874	positive regulation of stress-activated MAPK cascade	19_GO:0048518 positive regulation of biological process	-2.247	8.343704	4.419572	125	3	3.703704	2.098362	351|3156|7498	APP|HMGCR|XDH	-1.09015	45	0	0	-6.56867	52.14815
-2.247	GO:0001909	leukocyte mediated cytotoxicity	19_GO:0002376 immune system process	-2.247	8.343704	4.41	125	3	3.703704	2.098362	383|2147|4843	ARG1|F2|NOS2	-1.09015	56	0	0	-5.8959	130.3704

						9572											8	
-2.24519	GO:0097435	supramolecular fiber organization	19_GO:0009987 cellular process	-2.24519	3.262172	3.363327	746	7	8.641975	3.122032	246|351|1545|4137|4233|5295|6714	ALOX15|APP|CYP1B1|MAPT|MET|PIK3R1|SRC	-1.08873	50	0	0	-6.31767	11.54039
-2.23688	GO:0019693	ribose phosphate metabolic process	19_GO:0008152 metabolic process	-2.23688	4.378518	3.641234	397	5	6.17284	2.674018	142|351|3156|3643|7498	PARP1|APP|HMGCR|INSR|XDH	-1.08082	45	0	0	-6.56867	52.14815
-2.22793	GO:0035270	endocrine system development	19_GO:0032502 developmental process	-2.22793	8.212307	4.375237	127	3	3.703704	2.098362	207|1021|2932	AKT1|CDK6|GSK3B	-1.07266	43	0	0	-6.76848	45.34622
-2.22793	GO:0070304	positive regulation of stress-activated protein kinase signaling cascade	19_GO:0048518 positive regulation of biological process	-2.22793	8.212307	4.375237	127	3	3.703704	2.098362	351|3156|7498	APP|HMGCR|XDH	-1.07266	45	0	0	-6.56867	52.14815
-2.21852	GO:0035303	regulation of dephosphorylation	19_GO:0050789 regulation of biological process	-2.21852	8.148148	4.353432	128	3	3.703704	2.098362	134|2932|6714	ADORA1|GSK3B|SRC	-1.06365	68	0	0	-3.8713	18.96296
-2.2161	GO:0090257	regulation of muscle system process	19_GO:0032501 multicellular organismal	-2.2161	5.518323	3.86	252	4	4.938272	2.407398	134|142|5294|5743	ADORA1|PARP1|PIK3CG|PTGS2	-1.06161	64	0	0	-4.9498	45.34622

			process			9666											4	
-2.20992	GO:0060537	muscle tissue development	19_GO:0032502 developmental process	-2.20992	4.313329	3.598248	403	5	6.17284	2.674018	891|983|3156|4638|5292	CCNB1|CDK1|HMGCR|MYLK|PIM1	-1.05583	35	0	0	-7.56218	38.62826
-2.19995	GO:0045089	positive regulation of innate immune response	19_GO:0044419 biological process involved in interspecies interaction between organisms	-2.19995	8.022792	4.310526	130	3	3.703704	2.098362	4321|6714|6850	MMP12|SRC|SYK	-1.04664	41	0	0	-7.04885	38.62826
-2.19995	GO:0015918	sterol transport	19_GO:0051179 localization	-2.19995	8.022792	4.310526	130	3	3.703704	2.098362	1080|1645|10062	CFTR|AKR1C1|NR1H3	-1.04664	74	0	0	-3.61486	24.83245
-2.19822	GO:0061448	connective tissue development	19_GO:0032502 developmental process	-2.19822	5.453401	3.836906	255	4	4.938272	2.407398	2101|4322|5467|7298	ESRRA|MMP13|PPARD|TYMS	-1.04531	69	0	0	-3.77988	28.18819
-2.19745	GO:0050778	positive regulation of immune response	19_GO:0002376 immune system process	-2.19745	3.621399	3.414099	576	6	7.407407	2.909904	383|3558|4321|5747|6714|6850	ARG1|IL2|MMP12|PTK2|SRC|SYK	-1.04493	41	0	0	-7.04885	38.62826
-2.19077	GO:0007098	centrosome cycle	19_GO:0009987 cellular process	-2.19077	7.961549	4.28	131	3	3.703704	2.098362	983|1017|5347	CDK1|CDK2|PLK1	-1.03943	34	0	0	-7.5660	35.65685

						9415											8	
-2.19077	GO:0042476	odontogenesis	19_GO:0032502 developmental process	-2.19077	7.961549	4.289415	131	3	3.703704	2.098362	760|1080|6714	CA2|CFTR|SRC	-1.03943	36	0	0	-7.34456	54.89279
-2.19077	GO:1904950	negative regulation of establishment of protein localization	19_GO:0048519 negative regulation of biological process	-2.19077	7.961549	4.289415	131	3	3.703704	2.098362	3156|4137|10062	HMGCR|MAPT|NR1H3	-1.03943	74	0	0	-3.61486	24.83245
-2.18344	GO:0002696	positive regulation of leukocyte activation	19_GO:0002376 immune system process	-2.18344	4.250053	3.556093	409	5	6.17284	2.674018	207|558|3558|6714|6850	AKT1|AXL|IL2|SRC|SYK	-1.03248	25	0	0	-9.48567	22.67311
-2.18168	GO:0045667	regulation of osteoblast differentiation	19_GO:0032502 developmental process	-2.18168	7.901235	4.268527	132	3	3.703704	2.098362	1021|2101|5747	CDK6|ESRRA|PTK2	-1.03151	31	0	0	-8.64333	10.15049
-2.18168	GO:0007569	cell aging	19_GO:0032502 developmental process	-2.18168	7.901235	4.268527	132	3	3.703704	2.098362	983|1017|1021	CDK1|CDK2|CDK6	-1.03151	34	0	0	-7.56608	35.65685
-2.15744	GO:0006260	DNA replication	19_GO:0008152 metabolic process	-2.15744	5.3077	3.76	262	4	4.938272	2.407398	983|1017|1956|7150	CDK1|CDK2|EGFR|TOP1	-1.00766	34	0	0	-7.5660	35.65685

						2442											8	
-2.15485	GO:0002705	positive regulation of leukocyte mediated immunity	19_GO:0002376 immune system process	-2.15485	7.725652	4.207157	135	3	3.703704	2.098362	383|3558|4843	ARG1|IL2|NOS2	-1.00545	56	0	0	-5.89598	130.3704
-2.15172	GO:0043588	skin development	19_GO:0032502 developmental process	-2.15172	5.287518	3.752022	263	4	4.938272	2.407398	239|1956|4233|8644	ALOX12|EGFR|MET|AKR1C3	-1.00271	77	0	0	-3.22201	18.2976
-2.14603	GO:0031330	negative regulation of cellular catabolic process	19_GO:0048519 negative regulation of biological process	-2.14603	5.26749	3.741655	264	4	4.938272	2.407398	207|1457|4233|5294	AKT1|CSNK2A1|MET|PIK3CG	-0.99741	48	0	0	-6.42162	11.4927
-2.13796	GO:0005975	carbohydrate metabolic process	19_GO:0008152 metabolic process	-2.13796	3.517582	3.327947	593	6	7.407407	2.909904	207|351|2932|3643|5467|6714	AKT1|APP|GSK3B|INSR|PPARD|SRC	-0.98987	43	0	0	-6.76848	45.34622
-2.13732	GO:0032273	positive regulation of protein polymerization	19_GO:0048518 positive regulation of biological process	-2.13732	7.612868	4.167285	137	3	3.703704	2.098362	246|4137|4233	ALOX15|MAPT|MET	-0.98987	50	0	0	-6.31767	11.54039
-2.13732	GO:0007586	digestion	19_GO:0032501 multicellular organismal	-2.13732	7.612868	4.16	137	3	3.703704	2.098362	1645|1646|10062	AKR1C1|AKR1C2|NR1H3	-0.98987	74	0	0	-3.6148	24.83245

			process			7285											6	
-2.1361	GO:0050867	positive regulation of cell activation	19_GO:0048518 positive regulation of biological process	-2.1361	4.138742	3.480866	420	5	6.17284	2.674018	207|558|3558|6714|6850	AKT1|AXL|IL2|SRC|SYK	-0.98903	25	0	0	-9.48567	22.67311
-2.13187	GO:0034329	cell junction assembly	19_GO:0009987 cellular process	-2.13187	4.128911	3.474155	421	5	6.17284	2.674018	43|351|3791|5747|6714	ACHE|APP|KDR|PTK2|SRC	-0.98519	41	0	0	-7.04885	38.62826
-2.12765	GO:0006816	calcium ion transport	19_GO:0051179 localization	-2.12765	4.119127	3.467464	422	5	6.17284	2.674018	207|2147|4638|5294|5743	AKT1|F2|MYLK|PIK3CG|PTGS2	-0.98136	64	0	0	-4.94984	45.34622
-2.11156	GO:0030177	positive regulation of Wnt signaling pathway	19_GO:0048518 positive regulation of biological process	-2.11156	7.449735	4.108962	140	3	3.703704	2.098362	1457|1956|6714	CSNK2A1|EGFR|SRC	-0.96565	51	0	0	-6.31611	22.67311
-2.11091	GO:0050808	synapse organization	19_GO:0009987 cellular process	-2.11091	4.08045	3.440907	426	5	6.17284	2.674018	43|351|3480|3643|4137	ACHE|APP|IGF1R|INSR|MAPT	-0.96539	45	0	0	-6.56867	52.14815
-2.10311	GO:1903038	negative regulation of leukocyte cell-cell adhesion	19_GO:0022610 biological adhesion	-2.10311	7.3969	4.08	141	3	3.703704	2.098362	207|383|3558	AKT1|ARG1|IL2	-0.95874	25	0	0	-9.4856	22.67311

						9904										7	
-2.10311	GO:0008584	male gonad development	19_GO:0022414 reproductive process	-2.10311	7.3969	4.089904	141	3	3.703704	2.098362	367|2099|8644	AR|ESR1|AKR1C3	-0.95874	60	0	0	-5.48956	45.34622
-2.10311	GO:0050921	positive regulation of chemotaxis	19_GO:0040011 locomotion	-2.10311	7.3969	4.089904	141	3	3.703704	2.098362	3791|4233|5747	KDR|MET|PTK2	-0.95874	66	0	0	-4.22583	21.28496
-2.09668	GO:0060322	head development	19_GO:0032502 developmental process	-2.09668	3.057262	3.162398	796	7	8.641975	3.122032	238|351|558|1021|2932|4313|6714	ALK|APP|AXL|CDK6|GSK3B|MMP2|SRC	-0.9527	52	0	0	-6.26187	13.03704
-2.09473	GO:0046546	development of primary male sexual characteristics	19_GO:0022414 reproductive process	-2.09473	7.34481	4.071032	142	3	3.703704	2.098362	367|2099|8644	AR|ESR1|AKR1C3	-0.95113	60	0	0	-5.48956	45.34622
-2.07815	GO:0031023	microtubule organizing center organization	19_GO:0009987 cellular process	-2.07815	7.242798	4.033832	144	3	3.703704	2.098362	983|1017|5347	CDK1|CDK2|PLK1	-0.93532	34	0	0	-7.56608	35.65685
-2.07815	GO:0031644	regulation of nervous system process	19_GO:0032501 multicellular organismal	-2.07815	7.242798	4.03	144	3	3.703704	2.098362	134|351|6714	ADORA1|APP|SRC	-0.93532	68	0	0	-3.8713	18.96296

			process			3832												
-2.06996	GO:0030010	establishment of cell polarity	19_GO:0009987 cellular process	-2.06996	7.192848	4.015498	145	3	3.703704	2.098362	2932|5347|5747	GSK3B|PLK1|PTK2	-0.9279	43	0	0	-6.76848	45.34622
-2.06996	GO:0046718	viral entry into host cell	19_GO:0016032 viral process	-2.06996	7.192848	4.015498	145	3	3.703704	2.098362	558|983|1956	AXL|CDK1|EGFR	-0.9279	57	0	0	-5.76668	22.87199
-2.06883	GO:0007281	germ cell development	19_GO:0051704 multi-organism process	-2.06883	5.00222	3.601818	278	4	4.938272	2.407398	207|891|1080|6714	AKT1|CCNB1|CFTR|SRC	-0.92715	58	0	0	-5.66793	43.45679
-2.05818	GO:0002263	cell activation involved in immune response	19_GO:0002376 immune system process	-2.05818	4.96649	3.582608	280	4	4.938272	2.407398	351|3558|5294|6850	APP|IL2|PIK3CG|SYK	-0.91688	44	0	0	-6.70994	13.24397
-2.05377	GO:1903531	negative regulation of secretion by cell	19_GO:0048519 negative regulation of biological process	-2.05377	7.094986	3.979348	147	3	3.703704	2.098362	134|3156|10062	ADORA1|HMGCR|NR1H3	-0.91285	74	0	0	-3.61486	24.83245
-2.04584	GO:0016055	Wnt signaling pathway	19_GO:0023052 signaling	-2.04584	3.932741	3.33	442	5	6.17284	2.674018	351|1457|1956|2932|6714	APP|CSNK2A1|EGFR|GSK3B|SRC	-0.9053	51	0	0	-6.3161	22.67311

						783											1	
-2.04236	GO:0098813	nuclear chromosome segregation	19_GO:0009987 cellular process	-2.04236	4.913842	3.554134	283	4	4.938272	2.407398	891|5347|7153|9212	CCNB1|PLK1|TOP2A|AURKB	-0.90259	34	0	0	-7.56608	35.65685
-2.04236	GO:0006520	cellular amino acid metabolic process	19_GO:0008152 metabolic process	-2.04236	4.913842	3.554134	283	4	4.938272	2.407398	383|4843|4953|7299	ARG1|NOS2|ODC1|TYR	-0.90259	56	0	0	-5.89598	130.3704
-2.04187	GO:0000280	nuclear division	19_GO:0009987 cellular process	-2.04187	3.923864	3.331549	443	5	6.17284	2.674018	891|3643|5347|7153|9212	CCNB1|INSR|PLK1|TOP2A|AURKB	-0.90248	34	0	0	-7.56608	35.65685
-2.03791	GO:0198738	cell-cell signaling by wnt	19_GO:0023052 signaling	-2.03791	3.915026	3.325286	444	5	6.17284	2.674018	351|1457|1956|2932|6714	APP|CSNK2A1|EGFR|GSK3B|SRC	-0.89918	51	0	0	-6.31611	22.67311
-2.03781	GO:1905039	carboxylic acid transmembrane transport	19_GO:0051179 localization	-2.03781	6.999751	3.943868	149	3	3.703704	2.098362	207|4363|5243	AKT1|ABCC1|ABCB1	-0.89918	49	0	0	-6.34133	173.8272
-2.03713	GO:0044262	cellular carbohydrate metabolic process	19_GO:0008152 metabolic process	-2.03713	4.89654	3.544	284	4	4.938272	2.407398	207|2932|3643|6714	AKT1|GSK3B|INSR|SRC	-0.89888	43	0	0	-6.76848	45.34622

						732										
−2.02993	GO:1902850	microtubule cytoskeleton organization involved in mitosis	19_GO:0009987 cellular process	−2.02993	6.953086	3.926373	150	3	3.703704	2.098362	891|5347|9212	CCNB1|PLK1|AURKB	−0.89281	34	0	0	−7.56608	35.65685
−2.02993	GO:0070665	positive regulation of leukocyte proliferation	19_GO:0048518 positive regulation of biological process	−2.02993	6.953086	3.926373	150	3	3.703704	2.098362	3558|5747|6850	IL2|PTK2|SYK	−0.89281	41	0	0	−7.04885	38.62826
−2.02993	GO:1903825	organic acid transmembrane transport	19_GO:0051179 localization	−2.02993	6.953086	3.926373	150	3	3.703704	2.098362	207|4363|5243	AKT1|ABCC1|ABCB1	−0.89281	49	0	0	−6.34133	173.8272
−2.02674	GO:0072659	protein localization to plasma membrane	19_GO:0051179 localization	−2.02674	4.862298	3.526058	286	4	4.938272	2.407398	207|367|1956|5295	AKT1|AR|EGFR|PIK3R1	−0.89	54	0	0	−6.06931	37.58425
−2.0221	GO:0055067	monovalent inorganic cation homeostasis	19_GO:0065007 biological regulation	−2.0221	6.907039	3.909038	151	3	3.703704	2.098362	134|760|1080	ADORA1|CA2|CFTR	−0.88574	36	0	0	−7.34456	54.89279
−2.01894	GO:0120039	plasma membrane bounded cell projection morphogenesis	19_GO:0032502 developmental process	−2.01894	3.316257	3.155	629	6	7.407407	2.909904	351|1956|2932|4137|5747|6714	APP|EGFR|GSK3B|MAPT|PTK2|SRC	−0.88296	51	0	0	−6.31611	22.67311

						451												
-2.00664	GO:0003012	muscle system process	19_GO:0032501 multicellular organismal process	-2.00664	3.845734	3.275829	452	5	6.17284	2.674018	134|142|4638|5294|5743	ADORA1|PARP1|MYLK|PIK3CG|PTGS2	-0.87141	64	0	0	-4.94984	45.34622
-2.00662	GO:0051092	positive regulation of NF-kappaB transcription factor activity	19_GO:0050789 regulation of biological process	-2.00662	6.816751	3.874835	153	3	3.703704	2.098362	238|351|367	ALK|APP|AR	-0.87141	52	0	0	-6.26187	13.03704
-2.00627	GO:0048858	cell projection morphogenesis	19_GO:0032502 developmental process	-2.00627	3.295302	3.137058	633	6	7.407407	2.909904	351|1956|2932|4137|5747|6714	APP|EGFR|GSK3B|MAPT|PTK2|SRC	-0.87141	51	0	0	-6.31611	22.67311
-2.00114	GO:0140014	mitotic nuclear division	19_GO:0009987 cellular process	-2.00114	4.778754	3.480124	291	4	4.938272	2.407398	891|3643|5347|9212	CCNB1|INSR|PLK1|AURKB	-0.86666	34	0	0	-7.56608	35.65685

GO-CC

_LogP_MyList	GO	Description	LogP	Enrichment	Z-score	#GeneInGO	#GeneInGOAndHitList	%InGO	STDV %InGO	GeneID	Hits	Log(q-value)	GROUP_ID	FirstInGroupByEnrichment	FirstInGroupByLogP	BestLogPInGroup	BestEnrichmentInGroup
-7.42921	GO:0043235	receptor complex	-7.42921	7.886298	8.587677	529	12	14.81481	3.947186	196|238|351|558|1956|2322|3480|3643|3791|4233|6850|10062	AHR|ALK|APP|AXL|EGFR|FLT3|IGF1R|INSR|KDR|MET|SYK|NR1H3	-4.14955	1	1	1	-7.42921	7.886298
-	GO:1	protein kinase complex	-	21.9	10.	95	6	7.4	2.90	891|983|1017|1021|	CCNB1|CDK1|CDK2|	-	2	1	1	-	33.91749

6.47818	902911		6.47818	5712	98933			07407	9904	3480|3643	CDK6|IGF1R|INSR	3.6758				6.47818	
-6.35883	GO:0061695	transferase complex, transferring phosphorus-containing groups	-6.35883	11.68586	8.891704	238	8	9.876543	3.314965	891|983|1017|1021|3480|3643|5294|5295	CCNB1|CDK1|CDK2|CDK6|IGF1R|INSR|PIK3CG|PIK3R1	-3.6758	2	0	0	-6.47818	33.91749
-6.2565	GO:0045121	membrane raft	-6.2565	9.284537	8.217534	337	9	11.11111	3.491885	351|1509|1956|3643|3791|4137|5743|6714|9429	APP|CTSD|EGFR|INSR|KDR|MAPT|PTGS2|SRC|ABCG2	-3.6758	3	1	1	-6.2565	16.82198
-6.2565	GO:0098857	membrane microdomain	-6.2565	9.284537	8.217534	337	9	11.11111	3.491885	351|1509|1956|3643|3791|4137|5743|6714|9429	APP|CTSD|EGFR|INSR|KDR|MAPT|PTGS2|SRC|ABCG2	-3.6758	3	0	0	-6.2565	16.82198
-5.7233	GO:0098552	side of membrane	-5.7233	6.070155	6.912716	630	11	13.58025	3.80643	207|246|2147|3577|3643|5243|5747|5770|6714|6850|9429	AKT1|ALOX15|F2|CXCR1|INSR|ABCB1|PTK2|PTPN1|SRC|SYK|ABCG2	-3.22178	4	1	1	-5.7233	6.070155
-5.56679	GO:0005641	nuclear envelope lumen	-5.56679	104.2963	17.54712	10	3	3.703704	2.098362	240|351|9536	ALOX5|APP|PTGES	-3.13222	5	1	1	-5.56679	104.2963
-5.44483	GO:0031252	cell leading edge	-5.44483	7.3969	7.119842	423	9	11.11111	3.491885	134|207|351|1021|1956|3643|4137|4638|6714	ADORA1|AKT1|APP|CDK6|EGFR|INSR|MAPT|MYLK|SRC	-3.06825	3	0	0	-6.2565	16.82198
-5.22682	GO:0000307	cyclin-dependent protein kinase holoenzyme complex	-5.22682	33.91749	11.32867	41	4	4.938272	2.407398	891|983|1017|1021	CCNB1|CDK1|CDK2|CDK6	-2.90139	2	0	0	-6.47818	33.91749
-5.06991	GO:0048471	perinuclear region of cytoplasm	-5.06991	5.181839	6.1831	738	11	13.58025	3.80643	43|240|328|351|1956|4843|4953|5295|6714|7299|9536	ACHE|ALOX5|APEX1|APP|EGFR|NOS2|ODC1|PIK3R1|SRC|TYR|PTGES	-2.79024	5	0	0	-5.56679	104.2963
-4.95518	GO:0031234	extrinsic component of cytoplasmic side of plasma membrane	-4.95518	17.21061	8.765653	101	5	6.17284	2.674018	207|246|5747|6714|6850	AKT1|ALOX15|PTK2|SRC|SYK	-2.71691	6	1	1	-4.95518	17.21061
-4.66713	GO:0098562	cytoplasmic side of membrane	-4.66713	10.69706	7.298105	195	6	7.407407	2.909904	207|246|5747|5770|6714|6850	AKT1|ALOX15|PTK2|PTPN1|SRC|SYK	-2.46664	6	0	0	-4.95518	17.21061
-4.525	GO:00198	extrinsic component of membrane	-4.5	7.92697	6.5544	307	7	8.6419	3.122032	207|246|5294|5295|5747|6714|6850	AKT1|ALOX15|PIK3CG|PIK3R1|PTK2|SRC|SYK	-2.35	6	0	0	-4.9551	17.21061

51	98		2551	1	6			75				979				8	
-4.35175	GO:0031983	vesicle lumen	-4.35175	7.442141	6.293387	327	7	8.641975	3.122032	240|351|383|1509|1956|4353|7276	ALOX5|APP|ARG1|CTSD|EGFR|MPO|TTR	-2.21821	7	1	1	-4.35175	11.46113
-4.1896	GO:0031253	cell projection membrane	-4.1896	7.0132	6.053503	347	7	8.641975	3.122032	134|768|1956|3643|4137|6714|9429	ADORA1|CA9|EGFR|INSR|MAPT|SRC|ABCG2	-2.10622	3	0	0	-6.2565	16.82198
-4.18177	GO:0098687	chromosomal region	-4.18177	6.993047	6.042011	348	7	8.641975	3.122032	142|328|891|983|5347|7153|9212	PARP1|APEX1|CCNB1|CDK1|PLK1|TOP2A|AURKB	-2.10622	8	1	1	-4.18177	28.97119
-4.07017	GO:1902554	serine/threonine protein kinase complex	-4.07017	17.38272	7.881205	80	4	4.938272	2.407398	891|983|1017|1021	CCNB1|CDK1|CDK2|CDK6	-2.02095	2	0	0	-6.47818	33.91749
-4.0101	GO:0009925	basal plasma membrane	-4.0101	8.148148	6.170788	256	6	7.407407	2.909904	134|760|768|1956|4233|4363	ADORA1|CA2|CA9|EGFR|MET|ABCC1	-1.98571	9	1	1	-4.0101	8.148148
-3.87383	GO:0009898	cytoplasmic side of plasma membrane	-3.87383	10.22513	6.479712	170	5	6.17284	2.674018	207|246|5747|6714|6850	AKT1|ALOX15|PTK2|SRC|SYK	-1.90328	6	0	0	-4.95518	17.21061
-3.86753	GO:0005819	spindle	-3.86753	6.22399	5.5869	391	7	8.641975	3.122032	207|351|891|983|2908|5347|9212	AKT1|APP|CCNB1|CDK1|NR3C1|PLK1|AURKB	-1.90328	8	0	0	-4.18177	28.97119
-3.85007	GO:0019897	extrinsic component of plasma membrane	-3.85007	10.10623	6.433943	172	5	6.17284	2.674018	207|246|5747|6714|6850	AKT1|ALOX15|PTK2|SRC|SYK	-1.90328	6	0	0	-4.95518	17.21061
-3.84053	GO:0045178	basal part of cell	-3.84053	7.585185	5.893989	275	6	7.407407	2.909904	134|760|768|1956|4233|4363	ADORA1|CA2|CA9|EGFR|MET|ABCC1	-1.90328	9	0	0	-4.0101	8.148148
-3.81571	GO:0051233	spindle midzone	-3.81571	28.97119	9.019532	36	3	3.703704	2.098362	351|5347|9212	APP|PLK1|AURKB	-1.89777	8	0	0	-4.18177	28.97119
-3.746	GO:00312	leading edge membrane	-3.7	9.60371	6.2369	181	5	6.1728	2.674018	134|1956|3643|4137|6714	ADORA1|EGFR|INSR|MAPT|SRC	-1.84	3	0	0	-6.2565	16.82198

79	56		4679	1	07			4				734					
-3.62696	GO:0031012	extracellular matrix	-3.62696	4.879359	5.02553	570	8	9.876543	3.314965	43|1509|2147|4313|4314|4318|4321|4322	ACHE|CTSD|F2|MMP2|MMP3|MMP9|MMP12|MMP13	-1.7764	10	1	1	-3.62696	8.410992
-3.62183	GO:0030312	external encapsulating structure	-3.62183	4.870814	5.018947	571	8	9.876543	3.314965	43|1509|2147|4313|4314|4318|4321|4322	ACHE|CTSD|F2|MMP2|MMP3|MMP9|MMP12|MMP13	-1.7764	10	0	0	-3.62696	8.410992
-3.60891	GO:0000323	lytic vacuole	-3.60891	4.292029	4.836897	729	9	11.11111	3.491885	383|1080|1509|3643|4353|6714|7276|7299|57016	ARG1|CFTR|CTSD|INSR|MPO|SRC|TTR|TYR|AKR1B10	-1.7764	7	0	0	-4.35175	11.46113
-3.60891	GO:0005764	lysosome	-3.60891	4.292029	4.836897	729	9	11.11111	3.491885	383|1080|1509|3643|4353|6714|7276|7299|57016	ARG1|CFTR|CTSD|INSR|MPO|SRC|TTR|TYR|AKR1B10	-1.7764	7	0	0	-4.35175	11.46113
-3.57275	GO:0045177	apical part of cell	-3.57275	5.568834	5.169949	437	7	8.641975	3.122032	351|760|1080|1956|4363|5243|9429	APP|CA2|CFTR|EGFR|ABCC1|ABCB1|ABCG2	-1.75548	9	0	0	-4.0101	8.148148
-3.47226	GO:0034774	secretory granule lumen	-3.47226	6.478031	5.30999	322	6	7.407407	2.909904	240|351|383|1509|4353|7276	ALOX5|APP|ARG1|CTSD|MPO|TTR	-1.66972	7	0	0	-4.35175	11.46113
-3.45087	GO:0060205	cytoplasmic vesicle lumen	-3.45087	6.418234	5.276721	325	6	7.407407	2.909904	240|351|383|1509|4353|7276	ALOX5|APP|ARG1|CTSD|MPO|TTR	-1.66256	7	0	0	-4.35175	11.46113
-3.40645	GO:0005813	centrosome	-3.40645	4.522333	4.743587	615	8	9.876543	3.314965	328|891|983|1017|1021|2932|5347|9212	APEX1|CCNB1|CDK1|CDK2|CDK6|GSK3B|PLK1|AURKB	-1.63272	2	0	0	-6.47818	33.91749
-3.39387	GO:0005635	nuclear envelope	-3.39387	5.199958	4.920973	468	7	8.641975	3.122032	142|240|351|1956|3643|5743|9536	PARP1|ALOX5|APP|EGFR|INSR|PTGS2|PTGES	-1.63272	5	0	0	-5.56679	104.2963
-3.26892	GO:0016323	basolateral plasma membrane	-3.26892	7.557703	5.363404	230	5	6.17284	2.674018	134|760|768|1956|4363	ADORA1|CA2|CA9|EGFR|ABCC1	-1.52073	9	0	0	-4.0101	8.148148

-3.24374	GO:0030424	axon	-3.24374	4.27225	4.536907	651	8	9.876543	3.314965	134|351|760|1586|2932|3480|3643|4137	ADORA1|APP|CA2|CYP17A1|GSK3B|IGF1R|INSR|MAPT	-1.50814	11	1	1	-3.24374	15.33769
-3.18784	GO:0043025	neuronal cell body	-3.18784	4.799961	4.637376	507	7	8.641975	3.122032	134|351|383|1586|3643|4137|7150	ADORA1|APP|ARG1|CYP17A1|INSR|MAPT|TOP1	-1.46447	11	0	0	-3.24374	15.33769
-3.11505	GO:0032589	neuron projection membrane	-3.11505	16.82198	6.698528	62	3	3.703704	2.098362	134|3643|4137	ADORA1|INSR|MAPT	-1.40358	3	0	0	-6.2565	16.82198
-2.99817	GO:0044304	main axon	-2.99817	15.33769	6.357738	68	3	3.703704	2.098362	134|351|4137	ADORA1|APP|MAPT	-1.29829	11	0	0	-3.24374	15.33769
-2.9262	GO:0005876	spindle microtubule	-2.9262	14.4856	6.1537	72	3	3.703704	2.098362	983|5347|9212	CDK1|PLK1|AURKB	-1.2376	8	0	0	-4.18177	28.97119
-2.85777	GO:0044297	cell body	-2.85777	4.210346	4.188546	578	7	8.641975	3.122032	134|351|383|1586|3643|4137|7150	ADORA1|APP|ARG1|CYP17A1|INSR|MAPT|TOP1	-1.18016	11	0	0	-3.24374	15.33769
-2.82665	GO:1990234	transferase complex	-2.82665	3.678882	4.010216	756	8	9.876543	3.314965	891|983|1017|1021|3480|3643|5294|5295	CCNB1|CDK1|CDK2|CDK6|IGF1R|INSR|PIK3CG|PIK3R1	-1.15977	2	0	0	-6.47818	33.91749
-2.79105	GO:0005775	vacuolar lumen	-2.79105	7.992053	4.969003	174	4	4.938272	2.407398	383|1509|4353|7276	ARG1|CTSD|MPO|TTR	-1.13463	7	0	0	-4.35175	11.46113
-2.71875	GO:0005901	caveola	-2.71875	12.27015	5.589072	85	3	3.703704	2.098362	3643|5743|6714	INSR|PTGS2|SRC	-1.07255	3	0	0	-6.2565	16.82198
-2.70255	GO:0043197	dendritic spine	-2.70255	7.557703	4.793228	184	4	4.938272	2.407398	134|351|4137|5747	ADORA1|APP|MAPT|PTK2	-1.06754	11	0	0	-3.24374	15.33769
-2.694	GO:0044309	neuron spine	-2.694	7.51685	4.77638	185	4	4.938272	2.407398	134|351|4137|5747	ADORA1|APP|MAPT|PTK2	-1.06754	11	0	0	-3.24374	15.33769

-2.63422	GO:0035578	azurophil granule lumen	-2.63422	11.46113	5.368416	91	3	3.703704	2.098362	383|4353|7276	ARG1|MPO|TTR	-1.02328	7	0	0	-4.35175	11.46113
-2.63085	GO:0098794	postsynapse	-2.63085	3.838455	3.882535	634	7	8.641975	3.122032	134|207|351|2932|4137|5747|6714	ADORA1|AKT1|APP|GSK3B|MAPT|PTK2|SRC	-1.02328	11	0	0	-3.24374	15.33769
-2.5873	GO:0000775	chromosome, centromeric region	-2.5873	7.02332	4.568196	198	4	4.938272	2.407398	891|5347|7153|9212	CCNB1|PLK1|TOP2A|AURKB	-0.98887	8	0	0	-4.18177	28.97119
-2.56824	GO:0031970	organelle envelope lumen	-2.56824	10.8642	5.199755	96	3	3.703704	2.098362	240|351|9536	ALOX5|APP|PTGES	-0.98755	5	0	0	-5.56679	104.2963
-2.56824	GO:0005798	Golgi-associated vesicle	-2.56824	10.8642	5.199755	96	3	3.703704	2.098362	351|1080|7299	APP|CFTR|TYR	-0.98755	12	1	1	-2.56824	10.8642
-2.37535	GO:0000228	nuclear chromosome	-2.37535	6.126067	4.164844	227	4	4.938272	2.407398	1457|5347|7150|7153	CSNK2A1|PLK1|TOP1|TOP2A	-0.80592	13	1	1	-2.37535	6.126067
-2.36958	GO:0016324	apical plasma membrane	-2.36958	4.710763	3.85376	369	5	6.17284	2.674018	1080|1956|4363|5243|9429	CFTR|EGFR|ABCC1|ABCB1|ABCG2	-0.80592	9	0	0	-4.0101	8.148148
-2.32677	GO:0044853	plasma membrane raft	-2.32677	8.914213	4.607324	117	3	3.703704	2.098362	3643|5743|6714	INSR|PTGS2|SRC	-0.77138	3	0	0	-6.2565	16.82198
-2.265	GO:0000793	condensed chromosome	-2.265	5.699251	3.959654	244	4	4.938272	2.407398	891|5347|7153|9212	CCNB1|PLK1|TOP2A|AURKB	-0.71773	8	0	0	-4.18177	28.97119
-2.25666	GO:1904813	ficolin-1-rich granule lumen	-2.25666	8.410992	4.442111	124	3	3.703704	2.098362	240|1509|4318	ALOX5|CTSD|MMP9	-0.71736	10	0	0	-3.62696	8.410992
-2.21885	GO:0005769	early endosome	-2.21885	4.334842	3.612483	401	5	6.17284	2.674018	351|1080|1956|3791|5770	APP|CFTR|EGFR|KDR|PTPN1	-0.68737	12	0	0	-2.56824	10.8642

-2.16604	GO:0005667	transcription regulator complex	-2.16604	4.20889	3.528435	413	5	6.17284	2.674018	142|196|328|1457|10062	PARP1|AHR|APEX1|CSNK2A1|NR1H3	-0.64861	14	1	1	-2.16604	4.20889
-2.16372	GO:0000776	kinetochore	-2.16372	7.783306	4.227402	134	3	3.703704	2.098362	891|5347|9212	CCNB1|PLK1|AURKB	-0.64861	8	0	0	-4.18177	28.97119
-2.15742	GO:0009897	external side of plasma membrane	-2.15742	4.188606	3.514737	415	5	6.17284	2.674018	2147|3577|3643|5243|9429	F2|CXCR1|INSR|ABCB1|ABCG2	-0.64861	4	0	0	-5.7233	6.070155
-2.11091	GO:0062023	collagen-containing extracellular matrix	-2.11091	4.08045	3.440907	426	5	6.17284	2.674018	43|1509|2147|4313|4318	ACHE|CTSD|F2|MMP2|MMP9	-0.6094	10	0	0	-3.62696	8.410992
-2.07815	GO:0000779	condensed chromosome, centromeric region	-2.07815	7.242798	4.033832	144	3	3.703704	2.098362	891|5347|9212	CCNB1|PLK1|AURKB	-0.58963	8	0	0	-4.18177	28.97119
-2.06996	GO:0005777	peroxisome	-2.06996	7.192848	4.015498	145	3	3.703704	2.098362	3156|4843|7498	HMGCR|NOS2|XDH	-0.58963	15	1	1	-2.06996	7.192848
-2.06996	GO:0042579	microbody	-2.06996	7.192848	4.015498	145	3	3.703704	2.098362	3156|4843|7498	HMGCR|NOS2|XDH	-0.58963	15	0	0	-2.06996	7.192848

GO-MF

_LogP_MyList	GO	Description	LogP	Enrichment	Z-score	#GeneInGO	#GeneInGOAndHitList	%InGO	STDV%InGO	GeneID	Hits	Log(q-value)	GROUP_ID	FirstInGroupByEnrichment	FirstInGroupByLogP	BestLogPInGroup	BestEnrichmentInGroup
-20.8206	GO:0004672	protein kinase activity	-20.8220	14.43547	17.52292	578	24	29.6296	5.073592	207|238|558|983|1017|1021|1457|1612|1956|2322|2932|3480|3643|3791|4233|4638|5292|5294|5347|5747|6714|6850|7150|9212	AKT1|ALK|AXL|CDK1|CDK2|CDK6|CSNK2A1|DAPK1|EGFR|FLT3|GSK3B|IGF1R|INSR|KDR|MET|MYLK|PIM1|PIK3CG|PLK1|PTK2|SRC|SYK|TOP1|AURKB	-17.1357	1	1	1	-20.8206	14.43547

<table>
<tr><td></td><td></td><td></td><td>6</td><td></td><td>9</td><td></td><td></td><td>3</td><td></td><td></td><td></td><td></td><td></td><td></td><td></td><td></td></tr>
<tr><td>–19.0946</td><td>GO:0016773</td><td>phosphotransferase activity, alcohol group as acceptor</td><td>–19.0946</td><td>12.16283</td><td>15.89776</td><td>686</td><td>24</td><td>29.62963</td><td>5.073592</td><td>207|238|558|983|1017|1021|1457|1612|1956|2322|2932|3480|3643|3791|4233|4638|5292|5294|5347|5747|6714|6850|7150|9212</td><td>AKT1|ALK|AXL|CDK1|CDK2|CDK6|CSNK2A1|DAPK1|EGFR|FLT3|GSK3B|IGF1R|INSR|KDR|MET|MYLK|PIM1|PIK3CG|PLK1|PTK2|SRC|SYK|TOP1|AURKB</td><td>–15.7108</td><td>1</td><td>0</td><td>0</td><td>–20.8206</td><td>14.43547</td></tr>
<tr><td>–18.2581</td><td>GO:0016301</td><td>kinase activity</td><td>–18.2581</td><td>11.18459</td><td>15.14213</td><td>746</td><td>24</td><td>29.62963</td><td>5.073592</td><td>207|238|558|983|1017|1021|1457|1612|1956|2322|2932|3480|3643|3791|4233|4638|5292|5294|5347|5747|6714|6850|7150|9212</td><td>AKT1|ALK|AXL|CDK1|CDK2|CDK6|CSNK2A1|DAPK1|EGFR|FLT3|GSK3B|IGF1R|INSR|KDR|MET|MYLK|PIM1|PIK3CG|PLK1|PTK2|SRC|SYK|TOP1|AURKB</td><td>–15.0504</td><td>1</td><td>0</td><td>0</td><td>–20.8206</td><td>14.43547</td></tr>
<tr><td>–13.0282</td><td>GO:0004714</td><td>transmembrane receptor protein tyrosine kinase activity</td><td>–13.0282</td><td>30.11179</td><td>17.66023</td><td>127</td><td>11</td><td>13.58025</td><td>3.806643</td><td>238|558|1956|2322|3480|3643|3791|4233|5747|6714|6850</td><td>ALK|AXL|EGFR|FLT3|IGF1R|INSR|KDR|MET|PTK2|SRC|SYK</td><td>–9.9454</td><td>2</td><td>1</td><td>1</td><td>–13.0282</td><td>30.11179</td></tr>
<tr><td>–12.5675</td><td>GO:0016491</td><td>oxidoreductase activity</td><td>–12.5675</td><td>8.878269</td><td>11.69703</td><td>744</td><td>19</td><td>23.45679</td><td>4.708094</td><td>239|240|246|328|1545|1586|1588|1645|1646|3156|3292|4353|4843|5743|7299|7498|8644|9536|57016</td><td>ALOX12|ALOX5|ALOX15|APEX1|CYP1B1|CYP17A1|CYP19A1|AKR1C1|AKR1C2|HMGCR|HSD17B1|MPO|NOS2|PTGS2|TYR|XDH|AKR1C3|PTGES|AKR1B10</td><td>–9.61611</td><td>3</td><td>1</td><td>1</td><td>–12.5675</td><td>27.44639</td></tr>
<tr><td>–12.5228</td><td>GO:0004713</td><td>protein tyrosine kinase activity</td><td>–12.5228</td><td>27.12197</td><td>16.70127</td><td>141</td><td>11</td><td>13.58025</td><td>3.806643</td><td>238|558|1956|2322|3480|3643|3791|4233|5747|6714|6850</td><td>ALK|AXL|EGFR|FLT3|IGF1R|INSR|KDR|MET|PTK2|SRC|SYK</td><td>–9.61611</td><td>2</td><td>0</td><td>0</td><td>–13.0282</td><td>30.11179</td></tr>
<tr><td>–12.3552</td><td>GO:0019199</td><td>transmembrane receptor protein kinase activity</td><td>–12.3555</td><td>26.19313</td><td>16.39119</td><td>146</td><td>11</td><td>13.58025</td><td>3.806643</td><td>238|558|1956|2322|3480|3643|3791|4233|5747|6714|6850</td><td>ALK|AXL|EGFR|FLT3|IGF1R|INSR|KDR|MET|PTK2|SRC|SYK</td><td>–9.51545</td><td>2</td><td>0</td><td>0</td><td>–13.0282</td><td>30.11179</td></tr>
</table>

			2		8			5									
-11.7785	GO:0004674	protein serine/threonine kinase activity	-11.7785	12.01557	12.42149	434	15	18.51852	4.316086	207|983|1017|1021|1457|1612|1956|2932|4638|5292|5294|5347|6850|7150|9212	AKT1|CDK1|CDK2|CDK6|CSNK2A1|DAPK1|EGFR|GSK3B|MYLK|PIM1|PIK3CG|PLK1|SYK|TOP1|AURKB	-8.99674	1	0	0	-20.8206	14.43547
-11.6513	GO:0004879	nuclear receptor activity	-11.6513	53.48528	20.34626	52	8	9.876543	3.314965	196|367|2099|2100|2101|2908|5467|10062	AHR|AR|ESR1|ESR2|ESRRA|NR3C1|PPARD|NR1H3	-8.96646	4	1	1	-11.6513	115.8848
-11.6513	GO:0098531	ligand-activated transcription factor activity	-11.6513	53.48528	20.34626	52	8	9.876543	3.314965	196|367|2099|2100|2101|2908|5467|10062	AHR|AR|ESR1|ESR2|ESRRA|NR3C1|PPARD|NR1H3	-8.96646	4	0	0	-11.6513	115.8848
-9.83714	GO:0042562	hormone binding	-9.83714	32.33994	15.63347	86	8	9.876543	3.314965	367|1956|2908|3480|3643|5295|6462|7276	AR|EGFR|NR3C1|IGF1R|INSR|PIK3R1|SHBG|TTR	-7.19369	5	1	1	-9.83714	208.5926
-9.36016	GO:0019900	kinase binding	-9.36016	7.328681	9.493169	759	16	19.75309	4.423735	142|207|891|1956|2099|2908|2932|4137|5295|5347|5747|5770|6714|6850|7153|9212	PARP1|AKT1|CCNB1|EGFR|ESR1|NR3C1|GSK3B|MAPT|PIK3R1|PLK1|PTK2|PTPN1|SRC|SYK|TOP2A|AURKB	-6.7545	6	1	1	-9.36016	43.45679
-9.30623	GO:0005496	steroid binding	-9.3062	27.81235	14.4261	100	8	9.87654	3.314965	367|2099|2100|2101|2908|3292|6462|10062	AR|ESR1|ESR2|ESRRA|NR3C1|HSD17B1|SHBG|NR1H3	-6.73533	4	0	0	-11.6513	115.8848

			3		8			3									
-9.20281	GO:0004497	monooxygenase activity	-9.20281	27.00228	14.19939	103	8	9.876543	3.314965	1545|1586|1588|1645|1646|4843|7299|8644	CYP1B1|CYP17A1|CYP19A1|AKR1C1|AKR1C2|NOS2|TYR|AKR1C3	-6.66409	3	0	0	-12.5675	27.44639
-9.07017	GO:0019901	protein kinase binding	-9.07017	7.691467	9.472669	678	15	18.51852	4.316086	142|207|891|1956|2099|2908|2932|4137|5295|5347|5747|5770|6714|6850|7153	PARP1|AKT1|CCNB1|EGFR|ESR1|NR3C1|GSK3B|MAPT|PIK3R1|PLK1|PTK2|PTPN1|SRC|SYK|TOP2A	-6.56142	6	0	0	-9.36016	43.45679
-8.57061	GO:0016705	oxidoreductase activity, acting on paired donors, with incorporation or reduction of molecular oxygen	-8.57061	17.28668	11.80636	181	9	11.11111	3.491885	1545|1586|1588|1645|1646|4843|5743|7299|8644	CYP1B1|CYP17A1|CYP19A1|AKR1C1|AKR1C2|NOS2|PTGS2|TYR|AKR1C3	-6.08988	3	0	0	-12.5675	27.44639
-8.26763	GO:0019902	phosphatase binding	-8.26763	15.96372	11.29089	196	9	11.11111	3.491885	207|1956|3156|4137|4233|5295|5747|5770|6850	AKT1|EGFR|HMGCR|MAPT|MET|PIK3R1|PTK2|PTPN1|SYK	-5.81323	6	0	0	-9.36016	43.45679
-8.16849	GO:0106310	protein serine kinase activity	-8.16849	10.65236	9.885778	359	11	13.58025	3.80643	207|983|1017|1021|1457|1612|2932|5292|5294|5347|9212	AKT1|CDK1|CDK2|CDK6|CSNK2A1|DAPK1|GSK3B|PIM1|PIK3CG|PLK1|AURKB	-5.76655	1	0	0	-20.8206	14.43547
-8.16849	GO:0106311	protein threonine kinase activity	-8.1684	10.65236	9.88577	359	11	13.5802	3.80643	207|983|1017|1021|1457|1612|2932|5292|5294|5347|9212	AKT1|CDK1|CDK2|CDK6|CSNK2A1|DAPK1|GSK3B|PIM1|PIK3CG|PLK1|AURKB	-5.76655	1	0	0	-20.8206	14.43547

			9		8			5									
-8.15037	GO:0003707	steroid hormone receptor activity	-8.15037	72.42798	18.80196	24	5	6.17284	2.674018	2099|2100|2101|2908|5467	ESR1|ESR2|ESRRA|NR3C1|PPARD	-5.76655	4	0	0	-11.6513	115.8848
-7.90531	GO:0019903	protein phosphatase binding	-7.90531	18.54156	11.569558	150	8	9.876543	3.314965	207|1956|3156|4137|4233|5295|5747|5770	AKT1|EGFR|HMGCR|MAPT|MET|PIK3R1|PTK2|PTPN1	-5.54269	6	0	0	-9.36016	43.45679
-7.63965	GO:0018636	phenanthrene 9,10-monooxygenase activity	-7.63965	347.6543	32.2496	3	3	3.703704	2.098362	1645|1646|8644	AKR1C1|AKR1C2|AKR1C3	-5.31653	7	1	1	-7.63965	347.6543
-7.63965	GO:0047718	indanol dehydrogenase activity	-7.63965	347.6543	32.2496	3	3	3.703704	2.098362	1645|1646|57016	AKR1C1|AKR1C2|AKR1B10	-5.31653	7	0	0	-7.63965	347.6543
-7.51014	GO:0004032	alditol:NADP+ 1-oxidoreductase activity	-7.51014	115.8848	21.37911	12	4	4.938272	2.407398	1645|1646|8644|57016	AKR1C1|AKR1C2|AKR1C3|AKR1B10	-5.20551	7	0	0	-7.63965	347.6543
-7.35139	GO:0072582	17-beta-hydroxysteroid dehydrogenase (NADP+) activity	-7.3513	106.9706	20.5258	13	4	4.93827	2.407398	1645|1646|3292|8644	AKR1C1|AKR1C2|HSD17B1|AKR1C3	-5.06449	7	0	0	-7.63965	347.6543

			9		5			2									
-7.206 22	GO:0044594	17-beta-hydroxysteroid dehydrogenase (NAD+) activity	-7.206222	99.32981	19.76522	14	4	4.938272	2.407398	1645|1646|3292|8644	AKR1C1|AKR1C2|HSD17B1|AKR1C3	-4.93634	7	0	0	-7.63965	347.6543
-7.038 49	GO:0035410	dihydrotestosterone 17-beta-dehydrogenase activity	-7.03849	260.7407	27.90261	4	3	3.703704	2.098362	1645|3292|8644	AKR1C1|HSD17B1|AKR1C3	-4.80081	7	0	0	-7.63965	347.6543
-7.038 49	GO:0004052	arachidonate 12(S)-lipoxygenase activity	-7.03849	260.7407	27.90261	4	3	3.703704	2.098362	239|240|246	ALOX12|ALOX5|ALOX15	-4.80081	8	1	1	-7.03849	260.7407
-6.803 38	GO:0020037	heme binding	-6.80338	17.50777	10.47887	139	7	8.641975	3.122032	1545|1586|1588|4353|4843|5743|6714	CYP1B1|CYP17A1|CYP19A1|MPO|NOS2|PTGS2|SRC	-4.58093	3	0	0	-12.5675	27.44639
-6.762 51	GO:0051879	Hsp90 protein binding	-6.76251	39.50617	13.72904	44	5	6.17284	2.674018	196|1457|2908|3791|4137	AHR|CSNK2A1|NR3C1|KDR|MAPT	-4.55478	9	1	1	-6.76251	39.50617
-6.724 73	GO:0004303	estradiol 17-beta-dehydrogenase activity	-6.72447	77.25652	17.3818	18	4	4.93827	2.407398	1645|1646|3292|8644	AKR1C1|AKR1C2|HSD17B1|AKR1C3	-4.54504	7	0	0	-7.63965	347.6543

			3		5			2									
-6.72473	GO:0047834	D-threo-aldose 1-dehydrogenase activity	-6.724733	77.25652	17.381885	18	4	4.938272	2.407398	1645|1646|8644|57016	AKR1C1|AKR1C2|AKR1C3|AKR1B10	-4.54504	7	0	0	-7.63965	347.6543
-6.64146	GO:0043559	insulin binding	-6.641446	208.5926	24.93328	5	3	3.703704	2.098362	3480|3643|5295	IGF1R|INSR|PIK3R1	-4.48809	5	0	0	-9.83714	208.5926
-6.64146	GO:0047086	ketosteroid monooxygenase activity	-6.641446	208.5926	24.93328	5	3	3.703704	2.098362	1645|1646|8644	AKR1C1|AKR1C2|AKR1C3	-4.48809	7	0	0	-7.63965	347.6543
-6.59763	GO:0046906	tetrapyrrole binding	-6.597763	16.33275	10.07881	149	7	8.641975	3.122032	1545|1586|1588|4353|4843|5743|6714	CYP1B1|CYP17A1|CYP19A1|MPO|NOS2|PTGS2|SRC	-4.45685	3	0	0	-12.5675	27.44639
-6.55826	GO:0005506	iron ion binding	-6.558826	16.11643	10.00343	151	7	8.641975	3.122032	239|240|246|1545|1586|1588|7498	ALOX12|ALOX5|ALOX15|CYP1B1|CYP17A1|CYP19A1|XDH	-4.42971	8	0	0	-7.03849	260.7407
-6.35004	GO:0008106	alcohol dehydrogenase (NADP+) activity	-6.350000	63.20988	15.67777	22	4	4.938272	2.407398	1645|1646|8644|57016	AKR1C1|AKR1C2|AKR1C3|AKR1B10	-4.2334	7	0	0	-7.63965	347.6543

-6.26802	GO:0016702	oxidoreductase activity, acting on single donors with incorporation of molecular oxygen, incorporation of two atoms of oxygen	-6.26802	60.46162	15.32223	23	4	4.938272	2.407398	239|240|246|5743	ALOX12|ALOX5|ALOX15|PTGS2	-4.17575	8	0	0	-7.03849	260.7407
-6.26802	GO:0005158	insulin receptor binding	-6.26802	60.46162	15.32223	23	4	4.938272	2.407398	3480|5295|5770|6714	IGF1R|PIK3R1|PTPN1|SRC	-4.17575	10	1	1	-6.26802	60.46162
-6.25853	GO:0042803	protein homodimerization activity	-6.25853	6.135076	7.280367	680	12	14.81481	3.947186	43|196|207|3292|4843|4953|5743|7153|7298|7299|7498|9429	ACHE|AHR|AKT1|HSD17B1|NOS2|ODC1|PTGS2|TOP2A|TYMS|TYR|XDH|ABCG2	-4.17575	11	1	1	-6.25853	6.135076
-6.18978	GO:0016701	oxidoreductase activity, acting on single donors with incorporation of molecular oxygen	-6.18978	57.94239	14.98892	24	4	4.938272	2.407398	239|240|246|5743	ALOX12|ALOX5|ALOX15|PTGS2	-4.11772	8	0	0	-7.03849	260.7407
-6.04978	GO:0008134	transcription factor binding	-6.04978	6.559515	7.285477	583	11	13.58025	3.80643	142|196|328|367|2099|2322|2932|5292|5295|5467|6714	PARP1|AHR|APEX1|AR|ESR1|FLT3|GSK3B|PIM1|PIK3R1|PPARD|SRC	-3.98819	12	1	1	-6.04978	25.43812
-5.90873	GO:0004033	aldo-keto reductase (NADP) activity	-5.9087	49.6649	13.8374	28	4	4.93827	2.407398	1645|1646|8644|57016	AKR1C1|AKR1C2|AKR1C3|AKR1B10	-3.86434	7	0	0	-7.63965	347.6543

			3		1			2									
-5.89598	GO:0047023	androsterone dehydrogenase activity	-5.89598	130.3704	19.65556	8	3	3.703704	2.098362	1645|1646|8644	AKR1C1|AKR1C2|AKR1C3	-3.86434	7	0	0	-7.63965	347.6543
-5.89598	GO:0047045	testosterone 17-beta-dehydrogenase (NADP+) activity	-5.89598	130.3704	19.65556	8	3	3.703704	2.098362	1645|3292|8644	AKR1C1|HSD17B1|AKR1C3	-3.86434	7	0	0	-7.63965	347.6543
-5.78402	GO:0033764	steroid dehydrogenase activity, acting on the CH-OH group of donors, NAD or NADP as acceptor	-5.78402	46.35391	13.34906	30	4	4.938272	2.407398	1645|1646|3292|8644	AKR1C1|AKR1C2|HSD17B1|AKR1C3	-3.76194	7	0	0	-7.63965	347.6543
-5.75555	GO:0016616	oxidoreductase activity, acting on the CH-OH group of donors, NAD or NADP as acceptor	-5.755555	16.55497	9.398795	126	6	7.407407	2.909904	1645|1646|3156|3292|8644|57016	AKR1C1|AKR1C2|HMGCR|HSD17B1|AKR1C3|AKR1B10	-3.7428	7	0	0	-7.63965	347.6543
-5.72079	GO:0030235	nitric-oxide synthase regulator activity	-5.72079	115.8848	18.51387	9	3	3.703704	2.098362	207|1956|2099	AKT1|EGFR|ESR1	-3.72614	4	0	0	-11.6513	115.8848
-5.72079	GO:0047044	androstan-3-alpha,17-beta-diol dehydrogenase activity	-5.7207	115.8848	18.5138	9	3	3.70370	2.098362	1645|1646|8644	AKR1C1|AKR1C2|AKR1C3	-3.72614	7	0	0	-7.63965	347.6543

			9		7			4									
-5.695 97	GO:0031072	heat shock protein binding	-5.695975 97	16.169697	9.2751414 1	129	6	7.4074074 07	2.909904	134|196|1457|2908|3791|4137	ADORA1|AHR|CSNK2A1|NR3C1|KDR|MAPT	-3.71548	9	0	0	-6.76251	39.50617
-5.68053	GO:0017171	serine hydrolase activity	-5.680533	11.92931	8.4145481	204	7	8.6419750	3.122032	43|2147|4313|4314|4318|4321|4322	ACHE|F2|MMP2|MMP3|MMP9|MMP12|MMP13	-3.71548	13	1	1	-5.68053	20.15387
-5.66793	GO:0001223	transcription coactivator binding	-5.667933	43.45679	12.906663	32	4	4.9382 72	2.407398	196|367|2099|5467	AHR|AR|ESR1|PPARD	-3.71548	4	0	0	-11.6513	115.8848
-5.66793	GO:0043548	phosphatidylinositol 3-kinase binding	-5.667933	43.45679	12.906663	32	4	4.938272	2.407398	558|3480|3643|5295	AXL|IGF1R|INSR|PIK3R1	-3.71548	5	0	0	-9.83714	208.5926
-5.66793	GO:0051721	protein phosphatase 2A binding	-5.667933	43.45679	12.906663	32	4	4.938272	2.407398	207|3156|4137|5770	AKT1|HMGCR|MAPT|PTPN1	-3.71548	6	0	0	-9.36016	43.45679
-5.61661	GO:0061629	RNA polymerase II-specific DNA-binding transcription factor binding	-5.61666	9.301788	7.75120	299	8	9.87654	3.314965	142|328|367|2099|2322|2932|5467|6714	PARP1|APEX1|AR|ESR1|FLT3|GSK3B|PPARD|SRC	-3.67213	12	0	0	-6.04978	25.43812

-5.52576	GO:0016614	oxidoreductase activity, acting on CH-OH group of donors	1 -5.52576	15.11541	48.927783	138	6	37.407407	2.909904	1645|1646|3156|3292|8644|57016	AKR1C1|AKR1C2|HMGCR|HSD17B1|AKR1C3|AKR1B10	-3.5891	7	0	0	-7.63965	347.6543
-5.4574	GO:0016229	steroid dehydrogenase activity	-5.4574	38.62826	12.13353	36	4	4.938272	2.407398	1645|1646|3292|8644	AKR1C1|AKR1C2|HSD17B1|AKR1C3	-3.52843	7	0	0	-7.63965	347.6543
-5.42939	GO:0043560	insulin receptor substrate binding	-5.42939	94.81481	16.71462	11	3	3.703704	2.098362	3480|3643|5295	IGF1R|INSR|PIK3R1	-3.52269	5	0	0	-9.83714	208.5926
-5.42939	GO:0030283	testosterone dehydrogenase [NAD(P)] activity	-5.42939	94.81481	16.71462	11	3	3.703704	2.098362	1645|3292|8644	AKR1C1|HSD17B1|AKR1C3	-3.52269	7	0	0	-7.63965	347.6543
-5.42939	GO:0032052	bile acid binding	-5.42939	94.81481	16.71462	11	3	3.703704	2.098362	1645|1646|8644	AKR1C1|AKR1C2|AKR1C3	-3.52269	7	0	0	-7.63965	347.6543
-5.2686	GO:0019904	protein domain specific binding	-5.2686	5.439826	6.40290	703	11	13.5802	3.80643	351|367|1017|1080|2101|3643|4137|5747|6714|6850|7150	APP|AR|CDK2|CFTR|ESRRA|INSR|MAPT|PTK2|SRC|SYK|TOP1	-3.36909	14	1	1	-5.2686	5.439826

-5.14284	GO:0016709	oxidoreductase activity, acting on paired donors, with incorporation or reduction of molecular oxygen, NAD(P)H as one donor, and incorporation of one atom of oxygen	-5.14284	32.33994	911.04608	43	4	54.938272	2.407398	1645|1646|4843|8644	AKR1C1|AKR1C2|NOS2|AKR1C3	-3.205039	7	0	0	-7.63965	347.6543
-5.08848	GO:0015562	efflux transmembrane transporter activity	-5.08848	74.49735	14.77365	14	3	3.703704	2.098362	4363|5243|9429	ABCC1|ABCB1|ABCG2	-3.20297	15	1	1	-5.08848	74.49735
-4.99247	GO:0008559	ABC-type xenobiotic transporter activity	-4.99247	69.53086	14.25908	15	3	3.703704	2.098362	4363|5243|9429	ABCC1|ABCB1|ABCG2	-3.11381	15	0	0	-5.08848	74.49735
-4.9032	GO:0035173	histone kinase activity	-4.9032	65.18519	13.79311	16	3	3.703704	2.098362	983|1017|9212	CDK1|CDK2|AURKB	-3.03126	16	1	1	-4.9032	65.18519
-4.82299	GO:0004252	serine-type endopeptidase activity	-4.82299	11.3985	7.579771	183	6	7.407407	2.909904	2147|4313|4314|4318|4321|4322	F2|MMP2|MMP3|MMP9|MMP12|MMP13	-2.95769	13	0	0	-5.68053	20.15387
-4.8102	GO:0140359	ABC-type transporter activity	-4.8102	26.74264	9.9793	52	4	4.93827	2.407398	1080|4363|5243|9429	CFTR|ABCC1|ABCB1|ABCG2	-2.95143	15	0	0	-5.08848	74.49735

-4.783	GO:0031406	carboxylic acid binding	-4.783	11.21466	7.506945	186	6	27.407407	2.909904	1645|1646|4843|5467|7298|8644	AKR1C1|AKR1C2|NOS2|PPARD|TYMS|AKR1C3	-2.93067	17	1	1	-4.783	11.21466
-4.74328	GO:0140297	DNA-binding transcription factor binding	-4.74328	7.058971	6.50505	394	8	9.876543	3.314965	142|328|367|2099|2322|2932|5467|6714	PARP1|APEX1|AR|ESR1|FLT3|GSK3B|PPARD|SRC	-2.89729	12	0	0	-6.04978	25.43812
-4.69989	GO:0004222	metalloendopeptidase activity	-4.69989	15.248	8.187118	114	5	6.17284	2.674018	4313|4314|4318|4321|4322	MMP2|MMP3|MMP9|MMP12|MMP13	-2.86014	13	0	0	-5.68053	20.15387
-4.60525	GO:0008236	serine-type peptidase activity	-4.60525	10.42963	7.1878891	200	6	7.407407	2.909904	2147|4313|4314|4318|4321|4322	F2|MMP2|MMP3|MMP9|MMP12|MMP13	-2.77166	13	0	0	-5.68053	20.15387
-4.59809	GO:0001091	RNA polymerase II general transcription initiation factor binding	-4.59809	52.14815	2.289975	20	3	3.703704	2.098362	196|367|2099	AHR|AR|ESR1	-2.77057	4	0	0	-11.6513	115.8848
-4.32208	GO:0005518	collagen binding	-4.3220	20.15387	8.555574	69	4	4.93827	2.407398	43|4318|4321|4322	ACHE|MMP9|MMP12|MMP13	-2.50056	13	0	0	-5.68053	20.15387

			8		6			2									
-4.24254	GO:0019838	growth factor binding	-4.24254	12.24135	7.212808	142	5	6.17284	2.674018	1956|2322|3480|3643|3791	EGFR|FLT3|IGF1R|INSR|KDR	-2.42692	2	0	0	-13.0282	30.11179
-4.21368	GO:0005178	integrin binding	-4.21368	12.07133	7.153825	144	5	6.17284	2.674018	1956|3791|5747|6714|6850	EGFR|KDR|PTK2|SRC|SYK	-2.40389	2	0	0	-13.0282	30.11179
-4.14686	GO:0046875	ephrin receptor binding	-4.14686	37.24868	10.30697	28	3	3.703704	2.098362	5294|5770|6714	PIK3CG|PTPN1|SRC	-2.34282	10	0	0	-6.26802	60.46162
-4.11312	GO:0001221	transcription coregulator binding	-4.11312	17.82843	7.993502	78	4	4.938272	2.407398	196|367|2099|5467	AHR|AR|ESR1|PPARD	-2.32397	4	0	0	-11.6513	115.8848
-4.10033	GO:0016628	oxidoreductase activity, acting on the CH-CH group of donors, NAD or NADP as acceptor	-4.10033	35.96424	10.11791	29	3	3.703704	2.098362	1645|1646|8644	AKR1C1|AKR1C2|AKR1C3	-2.32397	7	0	0	-7.63965	347.6543
-4.10033	GO:0004693	cyclin-dependent protein serine/threonine kinase activity	-4.1003	35.96424	10.1179	29	3	3.70370	2.098362	983|1017|1021	CDK1|CDK2|CDK6	-2.32397	16	0	0	-4.9032	65.18519

			3		1			4									
-4.10033	GO:0097472	cyclin-dependent protein kinase activity	-4.10033	35.96424	10.11791	29	3	3.703704	2.098362	983|1017|1021	CDK1|CDK2|CDK6	-2.32397	16	0	0	-4.9032	65.18519
-4.10033	GO:0051059	NF-kappaB binding	-4.10033	35.96424	10.11791	29	3	3.703704	2.098362	328|2932|5467	APEX1|GSK3B|PPARD	-2.32397	18	1	1	-4.10033	35.96424
-4.04912	GO:0033293	monocarboxylic acid binding	-4.04912	17.16811	7.826568	81	4	4.938272	2.407398	1645|1646|5467|8644	AKR1C1|AKR1C2|PPARD|AKR1C3	-2.27809	7	0	0	-7.63965	347.6543
-4.01217	GO:0042910	xenobiotic transmembrane transporter activity	-4.01217	33.64397	9.767129	31	3	3.703704	2.098362	4363|5243|9429	ABCC1|ABCB1|ABCG2	-2.24641	15	0	0	-5.08848	74.49735
-3.97032	GO:0030332	cyclin binding	-3.97032	32.59259	9.603978	32	3	3.703704	2.098362	983|1017|1021	CDK1|CDK2|CDK6	-2.20976	16	0	0	-4.9032	65.18519
-3.89789	GO:0016853	isomerase activity	-3.8978	10.34685	6.52624	168	5	6.17284	2.674018	246|1080|7150|7153|9536	ALOX15|CFTR|TOP1|TOP2A|PTGES	-2.14247	19	1	1	-3.89789	10.34685

			9		7												
-3.8901	GO:0051117	ATPase binding	-3.8901	15.62491	7.421978	89	4	4.938272	2.407398	367|1956|2099|6714	AR|EGFR|ESR1|SRC	-2.13975	4	0	0	-11.6513	115.848
-3.74566	GO:0051213	dioxygenase activity	-3.74566	14.33626	7.066644	97	4	4.938272	2.407398	239|240|246|5743	ALOX12|ALOX5|ALOX15|PTGS2	-2.0047	8	0	0	-7.03849	260.7407
-3.74507	GO:0008395	steroid hydroxylase activity	-3.74507	27.44639	8.761843	38	3	3.703704	2.098362	1545|1586|1588	CYP1B1|CYP17A1|CYP19A1	-2.0047	3	0	0	-12.5675	27.44639
-3.67825	GO:0042169	SH2 domain binding	-3.67825	26.07407	8.523296	40	3	3.703704	2.098362	5747|6714|6850	PTK2|SRC|SYK	-1.94279	2	0	0	-13.0282	30.11179
-3.64614	GO:0030331	estrogen receptor binding	-3.64614	25.43812	8.410467	41	3	3.703704	2.098362	142|2099|6714	PARP1|ESR1|SRC	-1.91554	12	0	0	-6.04978	25.43812
-3.57641	GO:0008237	metallopeptidase activity	-3.5764	8.823714	5.91853	197	5	6.17284	2.674018	4313|4314|4318|4321|4322	MMP2|MMP3|MMP9|MMP12|MMP13	-1.85061	13	0	0	-5.68053	20.15387

-3.55136	GO:0042626	ATPase-coupled transmembrane transporter activity	1-3.55136	12.75796	96.605888	109	4	4.938272	2.407398	1080|4363|5243|9429	CFTR|ABCC1|ABCB1|ABCG2	-1.8303	15	0	0	-5.08848	74.49735
-3.54617	GO:0016829	lyase activity	-3.54617	8.691358	5.862863	200	5	6.17284	2.674018	760|768|1545|1586|4953	CA2|CA9|CYP1B1|CYP17A1|ODC1	-1.82981	20	1	1	-3.54617	16.2963
-3.53698	GO:0004175	endopeptidase activity	-3.53698	5.493409	5.119942	443	7	8.641975	3.122032	1509|2147|4313|4314|4318|4321|4322	CTSD|F2|MMP2|MMP3|MMP9|MMP12|MMP13	-1.82908	13	0	0	-5.68053	20.15387
-3.5362	GO:0005516	calmodulin binding	-3.5362	8.648117	5.844563	201	5	6.17284	2.674018	207|1612|1956|4638|4843	AKT1|DAPK1|EGFR|MYLK|NOS2	-1.82908	21	1	1	-3.5362	8.648117
-3.49713	GO:0004715	non-membrane spanning protein tyrosine kinase activity	-3.49713	22.67311	7.90131	46	3	3.703704	2.098362	5747|6714|6850	PTK2|SRC|SYK	-1.79455	2	0	0	-13.0282	30.11179
-3.44225	GO:0140296	general transcription initiation factor binding	-3.44222	21.7284	7.71970	48	3	3.70370	2.098362	196|367|2099	AHR|AR|ESR1	-1.74418	4	0	0	-11.6513	115.8848

			5		5			4									
−3.364 31	GO:0004601	peroxidase activity	−3.36431	20.45025	7.467031	51	3	3.703704	2.098362	4353|5743|9536	MPO|PTGS2|PTGES	−1.67069	22	1	1	−3.36431	20.45025
−3.3394	GO:0017046	peptide hormone binding	−3.33394	20.05698	7.387561	52	3	3.703704	2.098362	3480|3643|5295	IGF1R|INSR|PIK3R1	−1.65019	5	0	0	−9.83714	208.5926
−3.31499	GO:0050661	NADP binding	−3.31499	19.67855	7.310281	53	3	3.703704	2.098362	3156|3292|4843	HMGCR|HSD17B1|NOS2	−1.63014	23	1	1	−3.31499	19.67855
−3.2676	GO:0016684	oxidoreductase activity, acting on peroxide as acceptor	−3.2676	18.96296	7.161893	55	3	3.703704	2.098362	4353|5743|9536	MPO|PTGS2|PTGES	−1.59136	22	0	0	−3.36431	20.45025
−3.2676	GO:0005080	protein kinase C binding	−3.2676	18.96296	7.161893	55	3	3.703704	2.098362	207|6714|7153	AKT1|SRC|TOP2A	−1.59136	24	1	1	−3.2676	18.96296
−3.22201	GO:0016655	oxidoreductase activity, acting on NAD(P)H, quinone or similar compound as acceptor	−3.2220	18.2976	7.02113	57	3	3.70370	2.098362	1645|1646|8644	AKR1C1|AKR1C2|AKR1C3	−1.55	7	0	0	−7.63965	347.6543

			1		2			4									
-3.15669	GO:0016627	oxidoreductase activity, acting on the CH-CH group of donors	-3.156669	17.38272	6.822895	60	3	3.703704	2.098362	1645|1646|8644	AKR1C1|AKR1C2|AKR1C3	-1.48888	7	0	0	-7.63965	347.6543
-3.09472	GO:0016922	nuclear receptor binding	-3.09472	9.657064	5.593828	144	4	4.938272	2.407398	142|2099|2322|6714	PARP1|ESR1|FLT3|SRC	-1.43106	12	0	0	-6.04978	25.43812
-3.0748	GO:0016836	hydro-lyase activity	-3.0748	16.2963	6.579825	64	3	3.703704	2.098362	760|768|1545	CA2|CA9|CYP1B1	-1.41526	20	0	0	-3.54617	16.2963
-2.96164	GO:0001098	basal transcription machinery binding	-2.96164	14.89947	6.253623	70	3	3.703704	2.098362	196|367|2099	AHR|AR|ESR1	-1.31022	4	0	0	-11.6513	115.8848
-2.96164	GO:0001099	basal RNA polymerase II transcription machinery binding	-2.96164	14.89947	6.253623	70	3	3.703704	2.098362	196|367|2099	AHR|AR|ESR1	-1.31022	4	0	0	-11.6513	115.8848
-2.9142	GO:0033218	amide binding	-2.9142	5.062927	4.462202	412	6	7.407407	2.909904	43|3480|3643|5295|7298|9536	ACHE|IGF1R|INSR|PIK3R1|TYMS|PTGES	-1.26678	5	0	0	-9.83714	208.5926

-2.88369	GO:0003682	chromatin binding	-2.88369	4.254511	64.223611	572	7	78.641975	3.122032	328|367|1956|2099|4353|7150|7153	APEX1|AR|EGFR|ESR1|MPO|TOP1|TOP2A	-1.24024	4	0	0	-11.6513	115.8848
-2.82586	GO:0001618	virus receptor activity	-2.82586	13.37132	5.876377	78	3	3.703704	2.098362	558|983|1956	AXL|CDK1|EGFR	-1.18634	16	0	0	-4.9032	65.18519
-2.80995	GO:0140272	exogenous protein binding	-2.80995	13.20206	5.833119	79	3	3.703704	2.098362	558|983|1956	AXL|CDK1|EGFR	-1.17432	16	0	0	-4.9032	65.18519
-2.79424	GO:0016835	carbon-oxygen lyase activity	-2.79424	13.03704	5.790637	80	3	3.703704	2.098362	760|768|1545	CA2|CA9|CYP1B1	-1.16247	20	0	0	-3.54617	16.2963
-2.72853	GO:0015399	primary active transmembrane transporter activity	-2.72853	7.682968	4.844544	181	4	4.938272	2.407398	1080|4363|5243|9429	CFTR|ABCC1|ABCB1|ABCG2	-1.10058	15	0	0	-5.08848	74.49735
-2.70423	GO:0008013	beta-catenin binding	-2.7042	12.12748	5.55078	86	3	3.70370	2.098362	367|2099|2932	AR|ESR1|GSK3B	-1.08008	4	0	0	-11.6513	115.8848

			3		1			4								
-2.68988	GO:0016651	oxidoreductase activity, acting on NAD(P)H	-2.68988	11.98808	5.513119	87	3	3.703704	2.098362	1645|1646|8644	AKR1C1|AKR1C2|AKR1C3	-1.07322	7	0	0	-7.63965	347.6543
-2.68988	GO:0016209	antioxidant activity	-2.68988	11.98808	5.513119	87	3	3.703704	2.098362	4353|5743|9536	MPO|PTGS2|PTGES	-1.07322	22	0	0	-3.36431	20.45025
-2.67571	GO:0051219	phosphoprotein binding	-2.67571	11.85185	5.476068	88	3	3.703704	2.098362	5295|6714|6850	PIK3R1|SRC|SYK	-1.06275	2	0	0	-13.0282	30.11179
-2.65715	GO:0044389	ubiquitin-like protein ligase binding	-2.65715	5.50086	4.32149	316	5	6.17284	2.674018	891|1956|2932|5243|6714	CCNB1|EGFR|GSK3B|ABCB1|SRC	-1.04785	25	1	1	-2.65715	5.50086
-2.61941	GO:0008233	peptidase activity	-2.61941	3.820377	3.867135	637	7	8.641975	3.122032	1509|2147|4313|4314|4318|4321|4322	CTSD|F2|MMP2|MMP3|MMP9|MMP12|MMP13	-1.01375	13	0	0	-5.68053	20.15387
-2.58114	GO:0072341	modified amino acid binding	-2.58111	10.97856	5.23247	95	3	3.70370	2.098362	558|7298|9536	AXL|TYMS|PTGES	-0.97908	26	1	1	-2.58114	10.97856

			4		7				4								
-2.5703	GO:0042277	peptide binding	-2.57033	5.251576	4.179029	331	5	6.172844	2.674018	43|3480|3643|5295|9536	ACHE|IGF1R|INSR|PIK3R1|PTGES	-0.97181	5	0	0	-9.83714	208.5926
-2.31573	GO:0050839	cell adhesion molecule binding	-2.31573	3.834423	3.585453	544	6	7.407407	2.909904	1956|3791|5747|5770|6714|6850	EGFR|KDR|PTK2|PTPN1|SRC|SYK	-0.72079	2	0	0	-13.0282	30.11179
-2.12909	GO:1901681	sulfur compound binding	-2.12909	5.208304	3.710868	267	4	4.938272	2.407398	351|2147|4353|9536	APP|F2|MPO|PTGES	-0.53766	22	0	0	-3.36431	20.45025
-2.10311	GO:0070851	growth factor receptor binding	-2.10311	7.3969	4.089904	141	3	3.703704	2.098362	351|3558|6714	APP|IL2|SRC	-0.51518	24	0	0	-3.2676	18.96296
-2.07815	GO:0043177	organic acid binding	-2.07815	7.242798	4.033832	144	3	3.703704	2.098362	4843|5467|7298	NOS2|PPARD|TYMS	-0.49368	17	0	0	-4.783	11.21466

KEGG-pathway

_LogP_MyList	GO	Description	LogP	Enrichment	Z-score	#GeneInGO	#GeneInGOAndHitList	%InGO	STDV %InGO	GeneID	Hits	Log(q-value)	GROUP_ID	FirstInGroupByEnrichment	FirstInGroupByLogP	BestLogPInGroup	BestEnrichmentInGroup

–14.8972	hsa05200	Pathways in cancer	–14.8972	14.96234	15.00957	395	17	20.98765	4.52467	207|367|1017|1021|1612|1956|2322|2932|3480|4233|4313|4318|4843|5295|5467|5743|5747	AKT1|AR|CDK2|CDK6|DAPK1|EGFR|FLT3|GSK3B|IGF1R|MET|MMP2|MMP9|NOS2|PIK3R1|PPARD|PTGS2|PTK2	–11.9979	1	1	1	–14.8972	39.60618847
–13.7121	ko04913	Ovarian steroidogenesis	–13.7121	62.57778	23.40663	50	9	11.11111	3.491885	240|1545|1586|1588|3292|3480|3643|5743|8644	ALOX5|CYP1B1|CYP17A1|CYP19A1|HSD17B1|IGF1R|INSR|PTGS2|AKR1C3	–11.1138	2	1	1	–13.7121	62.57777778
–13.3126	hsa04913	Ovarian steroidogenesis	–13.3126	56.88889	22.28309	55	9	11.11111	3.491885	240|1545|1586|1588|3292|3480|3643|5743|8644	ALOX5|CYP1B1|CYP17A1|CYP19A1|HSD17B1|IGF1R|INSR|PTGS2|AKR1C3	–10.8905	2	0	0	–13.7121	62.57777778
–12.6956	hsa01522	Endocrine resistance	–12.6956	36.21399	18.5625	96	10	12.34568	3.65512	207|1956|2099|2100|3480|4313|4318|5295|5747|6714	AKT1|EGFR|ESR1|ESR2|IGF1R|MMP2|MMP9|PIK3R1|PTK2|SRC	–10.4953	1	0	0	–14.8972	39.60618847
–12.6956	ko01522	Endocrine resistance	–12.6956	36.21399	18.5625	96	10	12.34568	3.65512	207|1956|2099|2100|3480|4313|4318|5295|5747|6714	AKT1|EGFR|ESR1|ESR2|IGF1R|MMP2|MMP9|PIK3R1|PTK2|SRC	–10.4953	1	0	0	–14.8972	39.60618847
–12.6073	hsa04151	PI3K-Akt signaling pathway	–12.6073	13.7232	13.41165	380	15	18.51852	4.316086	207|1017|1021|1956|2322|2932|3480|3558|3643|3791|4233|5294|5295|5747|6850	AKT1|CDK2|CDK6|EGFR|FLT3|GSK3B|IGF1R|IL2|INSR|KDR|MET|PIK3CG|PIK3R1|PTK2|SYK	–10.4862	1	0	0	–14.8972	39.60618847
–11.9914	ko04151	PI3K-Akt signaling pathway	–11.9914	14.23146	13.22264	342	14	17.28395	4.201205	207|1017|1021|1956|2932|3480|3558|3643|3791|4233|5294|5295|5747|6850	AKT1|CDK2|CDK6|EGFR|GSK3B|IGF1R|IL2|INSR|KDR|MET|PIK3CG|PIK3R1|PTK2|SYK	–9.9372	1	0	0	–14.8972	39.60618847
–11.8265	hsa01521	EGFR tyrosine kinase inhibitor resistance	–11.8265	39.60619	18.45544	79	9	11.11111	3.491885	207|558|1956|2932|3480|3791|4233|5295|6714	AKT1|AXL|EGFR|GSK3B|IGF1R|KDR|MET|PIK3R1|SRC	–9.8815	1	0	0	–14.8972	39.60618847
–11.8265	ko01521	EGFR tyrosine kinase inhibitor resistance	–11.8265	39.60619	18.45544	79	9	11.11111	3.491885	207|558|1956|2932|3480|3791|4233|5295|6714	AKT1|AXL|EGFR|GSK3B|IGF1R|KDR|MET|PIK3R1|SRC	–9.8815	1	0	0	–14.8972	39.60618847
–10.7893	ko05205	Proteoglycans in cancer	–10.7893	18.83841	13.69996	203	11	13.58025	3.80643	207|1956|2099|3480|3791|4233|4313|4318|5295|5747|6714	AKT1|EGFR|ESR1|IGF1R|KDR|MET|MMP2|MMP9|PIK3R1|PTK2|SRC	–8.89007	1	0	0	–14.8972	39.60618847
–10.4	hsa05	Proteoglycans in cancer	–10.	17.542	13.16	218	11	13.58	3.80643	207|1956|2099|3480|3791|4233|4313|4318|5295|5747|	AKT1|EGFR|ESR1|IGF1R|KDR|MET|MMP2|MMP9|PIK3R1|PTK2|SRC	–8.59	1	0	0	–14.89	39.60618847

553	205		4553	19	9			025		6714		744				72	
-9.52178	hsa04510	Focal adhesion	-9.52178	17.47007	12.52293	199	10	12.34568	3.65512	207|1956|2932|3480|3791|4233|4638|5295|5747|6714	AKT1|EGFR|GSK3B|IGF1R|KDR|MET|MYLK|PIK3R1|PTK2|SRC	-7.75316	1	0	0	-14.8972	39.60618847
-9.52178	ko04510	Focal adhesion	-9.52178	17.47007	12.52293	199	10	12.34568	3.65512	207|1956|2932|3480|3791|4233|4638|5295|5747|6714	AKT1|EGFR|GSK3B|IGF1R|KDR|MET|MYLK|PIK3R1|PTK2|SRC	-7.75316	1	0	0	-14.8972	39.60618847
-9.47634	hsa00140	Steroid hormone biosynthesis	-9.47634	41.95828	16.77055	58	7	8.641975	3.122032	1545|1586|1588|1645|1646|3292|8644	CYP1B1|CYP17A1|CYP19A1|AKR1C1|AKR1C2|HSD17B1|AKR1C3	-7.75316	2	0	0	-13.7121	62.57777778
-9.47634	ko00140	Steroid hormone biosynthesis	-9.47634	41.95828	16.77055	58	7	8.641975	3.122032	1545|1586|1588|1645|1646|3292|8644	CYP1B1|CYP17A1|CYP19A1|AKR1C1|AKR1C2|HSD17B1|AKR1C3	-7.75316	2	0	0	-13.7121	62.57777778
-9.42356	hsa04915	Estrogen signaling pathway	-9.42356	21.57854	13.34331	145	9	11.11111	3.491885	207|1509|1956|2099|2100|4313|4318|5295|6714	AKT1|CTSD|EGFR|ESR1|ESR2|MMP2|MMP9|PIK3R1|SRC	-7.72841	1	0	0	-14.8972	39.60618847
-9.37703	ko04915	Estrogen signaling pathway	-9.37703	28.37994	14.58299	98	8	9.876543	3.314965	207|1956|2099|2100|4313|4318|5295|6714	AKT1|EGFR|ESR1|ESR2|MMP2|MMP9|PIK3R1|SRC	-7.70821	1	0	0	-14.8972	39.60618847
-8.88769	ko04917	Prolactin signaling pathway	-8.88769	34.76543	15.19168	70	7	8.641975	3.122032	207|1586|2099|2100|2932|5295|6714	AKT1|CYP17A1|ESR1|ESR2|GSK3B|PIK3R1|SRC	-7.24369	1	0	0	-14.8972	39.60618847
-8.8002	hsa04520	Adherens junction	-8.8002	33.79973	14.96706	72	7	8.641975	3.122032	1457|1956|3480|3643|4233|5770|6714	CSNK2A1|EGFR|IGF1R|INSR|MET|PTPN1|SRC	-7.20195	3	1	1	-8.8002	33.79972565
-8.8002	ko04520	Adherens junction	-8.8002	33.79973	14.96706	72	7	8.641975	3.122032	1457|1956|3480|3643|4233|5770|6714	CSNK2A1|EGFR|IGF1R|INSR|MET|PTPN1|SRC	-7.20195	3	0	0	-8.8002	33.79972565
-8.63274	hsa04917	Prolactin signaling pathway	-8.63274	32.02079	14.54422	76	7	8.641975	3.122032	207|1586|2099|2100|2932|5295|6714	AKT1|CYP17A1|ESR1|ESR2|GSK3B|PIK3R1|SRC	-7.05569	1	0	0	-14.8972	39.60618847

-8.32439	hsa05222	Small cell lung cancer	-8.324339	28.97119	13.78934	84	7	8.641975	3.122032	207|1017|1021|4843|5295|5743|5747	AKT1|CDK2|CDK6|NOS2|PIK3R1|PTGS2|PTK2	-6.78684	1	0	0	-14.8972	39.60618847
-8.32439	ko05222	Small cell lung cancer	-8.324339	28.97119	13.78934	84	7	8.641975	3.122032	207|1017|1021|4843|5295|5743|5747	AKT1|CDK2|CDK6|NOS2|PIK3R1|PTGS2|PTK2	-6.78684	1	0	0	-14.8972	39.60618847
-8.21676	hsa05215	Prostate cancer	-8.21676	27.97219	13.53293	87	7	8.641975	3.122032	207|367|1017|1956|2932|3480|5295	AKT1|AR|CDK2|EGFR|GSK3B|IGF1R|PIK3R1	-6.73092	1	0	0	-14.8972	39.60618847
-8.21676	ko05215	Prostate cancer	-8.21676	27.97219	13.53293	87	7	8.641975	3.122032	207|367|1017|1956|2932|3480|5295	AKT1|AR|CDK2|EGFR|GSK3B|IGF1R|PIK3R1	-6.73092	1	0	0	-14.8972	39.60618847
-8.21522	hsa04068	foxo signaling pathway	-8.21522	20.30098	12.16306	137	8	9.876543	3.314965	207|891|1017|1956|3480|3643|5295|5347	AKT1|CCNB1|CDK2|EGFR|IGF1R|INSR|PIK3R1|PLK1	-6.73092	4	1	1	-8.21522	25.34979424
-8.04463	ko05224	Breast cancer	-8.04463	19.31413	11.83381	144	8	9.876543	3.314965	207|1021|1956|2099|2100|2932|3480|5295	AKT1|CDK6|EGFR|ESR1|ESR2|GSK3B|IGF1R|PIK3R1	-6.57672	1	0	0	-14.8972	39.60618847
-7.91617	hsa04914	Progesterone-mediated oocyte maturation	-7.91617	25.34979	12.83561	96	7	8.641975	3.122032	207|891|983|1017|3480|5295|5347	AKT1|CCNB1|CDK1|CDK2|IGF1R|PIK3R1|PLK1	-6.4793	4	0	0	-8.21522	25.34979424
-7.91617	ko04914	Progesterone-mediated oocyte maturation	-7.91617	25.34979	12.83561	96	7	8.641975	3.122032	207|891|983|1017|3480|5295|5347	AKT1|CCNB1|CDK1|CDK2|IGF1R|PIK3R1|PLK1	-6.4793	4	0	0	-8.21522	25.34979424
-7.72779	hsa04370	VEGF signaling pathway	-7.72779	35.35468	14.18768	59	6	7.407407	2.909904	207|3791|5295|5743|5747|6714	AKT1|KDR|PIK3R1|PTGS2|PTK2|SRC	-6.31988	1	0	0	-14.8972	39.60618847
-7.72779	ko04370	VEGF signaling pathway	-7.72779	35.35468	14.18768	59	6	7.407407	2.909904	207|3791|5295|5743|5747|6714	AKT1|KDR|PIK3R1|PTGS2|PTK2|SRC	-6.31988	1	0	0	-14.8972	39.60618847
-7.64	hsa05	Breast cancer	-7.6	17.168	11.08	162	8	9.876	3.31496	207|1021|1956|2099|2100|2932|3480|5295	AKT1|CDK6|EGFR|ESR1|ESR2|GSK3B|IGF1R|PIK3R1	-6.24	1	0	0	-14.89	39.60618847

375	224		4375	11	444			543	5			963				72	
–7.60218	hsa04072	Phospholipase D signaling pathway	–7.602018	16.95875	11.00862	164	8	9.876543	3.314965	207|1956|2147|3577|3643|5294|5295|6850	AKT1|EGFR|F2|CXCR1|INSR|PIK3CG|PIK3R1|SYK	–6.24063	1	0	0	–14.8972	39.60618847
–7.59584	hsa00590	Arachidonic acid metabolism	–7.59584	33.64397	13.82042	62	6	7.407407	2.909904	239|240|246|5743|8644|9536	ALOX12|ALOX5|ALOX15|PTGS2|AKR1C3|PTGES	–6.24063	5	1	1	–7.59584	33.64396655
–7.59584	ko00590	Arachidonic acid metabolism	–7.59584	33.64397	13.82042	62	6	7.407407	2.909904	239|240|246|5743|8644|9536	ALOX12|ALOX5|ALOX15|PTGS2|AKR1C3|PTGES	–6.24063	5	0	0	–7.59584	33.64396655
–7.31274	hsa05218	Melanoma	–7.31274	30.23081	13.05696	69	6	7.407407	2.909904	207|1021|1956|3480|4233|5295	AKT1|CDK6|EGFR|IGF1R|MET|PIK3R1	–5.98167	1	0	0	–14.8972	39.60618847
–7.31274	ko05218	Melanoma	–7.31274	30.23081	13.05696	69	6	7.407407	2.909904	207|1021|1956|3480|4233|5295	AKT1|CDK6|EGFR|IGF1R|MET|PIK3R1	–5.98167	1	0	0	–14.8972	39.60618847
–7.00258	hsa04611	Platelet activation	–7.00258	18.71985	10.87628	130	7	8.641975	3.122032	207|2147|4638|5294|5295|6714|6850	AKT1|F2|MYLK|PIK3CG|PIK3R1|SRC|SYK	–5.68309	6	1	1	–7.00258	42.39686841
–6.9207	hsa05219	Bladder cancer	–6.9207	42.39687	14.24688	41	5	6.17284	2.674018	1612|1956|4313|4318|6714	DAPK1|EGFR|MMP2|MMP9|SRC	–5.62579	6	0	0	–7.00258	42.39686841
–6.9207	ko05219	Bladder cancer	–6.9207	42.39687	14.24688	41	5	6.17284	2.674018	1612|1956|4313|4318|6714	DAPK1|EGFR|MMP2|MMP9|SRC	–5.62579	6	0	0	–7.00258	42.39686841
–6.91228	ko05162	Measles	–6.91228	18.16105	10.69488	134	7	8.641975	3.122032	207|1017|1021|1457|2932|3558|5295	AKT1|CDK2|CDK6|CSNK2A1|GSK3B|IL2|PIK3R1	–5.62579	1	0	0	–14.8972	39.60618847
–6.77259	ko04015	Rap1 signaling pathway	–6.77259	13.24397	9.565363	210	8	9.876543	3.314965	207|1956|3480|3643|3791|4233|5295|6714	AKT1|EGFR|IGF1R|INSR|KDR|MET|PIK3R1|SRC	–5.49657	1	0	0	–14.8972	39.60618847

-6.74003	ko05418	Fluid shear stress and atherosclerosis	-6.74003	17.13789	10.35458	142	7	8.641975	3.120322	207|3791|4313|4318|5295|5747|6714	AKT1|KDR|MMP2|MMP9|PIK3R1|PTK2|SRC	-5.49003	1	0	0	-14.8972	39.60618847
-6.73609	hsa04012	ErbB signaling pathway	-6.73609	24.25495	11.6004	86	6	7.407407	2.909904	207|1956|2932|5295|5747|6714	AKT1|EGFR|GSK3B|PIK3R1|PTK2|SRC	-5.49003	1	0	0	-14.8972	39.60618847
-6.73609	ko04012	ErbB signaling pathway	-6.73609	24.25495	11.6004	86	6	7.407407	2.909904	207|1956|2932|5295|5747|6714	AKT1|EGFR|GSK3B|PIK3R1|PTK2|SRC	-5.49003	1	0	0	-14.8972	39.60618847
-6.69202	ko05206	MicroRNAs in cancer	-6.69202	10.46451	8.836815	299	9	11.11111	3.491885	1021|1545|1956|4233|4318|4363|5243|5292|5743	CDK6|CYP1B1|EGFR|MET|MMP9|ABCC1|ABCB1|PIM1|PTGS2	-5.4555	7	1	1	-6.69202	10.46451133
-6.65776	ko04072	Phospholipase D signaling pathway	-6.65776	16.66836	10.19466	146	7	8.641975	3.120322	207|1956|3577|3643|5294|5295|6850	AKT1|EGFR|CXCR1|INSR|PIK3CG|PIK3R1|SYK	-5.43059	1	0	0	-14.8972	39.60618847
-6.61753	hsa05418	Fluid shear stress and atherosclerosis	-6.61753	16.44311	10.11705	148	7	8.641975	3.120322	207|3791|4313|4318|5295|5747|6714	AKT1|KDR|MMP2|MMP9|PIK3R1|PTK2|SRC	-5.3995	1	0	0	-14.8972	39.60618847
-6.59545	hsa05206	MicroRNAs in cancer	-6.59545	10.19182	8.697512	307	9	11.11111	3.491885	1021|1545|1956|4233|4318|4363|5243|5292|5743	CDK6|CYP1B1|EGFR|MET|MMP9|ABCC1|ABCB1|PIM1|PTGS2	-5.38637	7	0	0	-6.69202	10.46451133
-6.50024	hsa04015	Rap1 signaling pathway	-6.50024	12.1984	9.118693	228	8	9.876543	3.314965	207|1956|3480|3643|3791|4233|5295|6714	AKT1|EGFR|IGF1R|INSR|KDR|MET|PIK3R1|SRC	-5.29993	1	0	0	-14.8972	39.60618847
-6.42474	hsa05162	Measles	-6.42474	15.40241	9.750556	158	7	8.641975	3.120322	207|1017|1021|1457|2932|3558|5295	AKT1|CDK2|CDK6|CSNK2A1|GSK3B|IL2|PIK3R1	-5.23303	1	0	0	-14.8972	39.60618847
-6.26766	hsa05221	Acute myeloid leukemia	-6.26766	31.60494	12.20229	55	5	6.17284	2.674018	207|2322|5292|5295|5467	AKT1|FLT3|PIM1|PIK3R1|PPARD	-5.09266	1	0	0	-14.8972	39.60618847
-6.26	ko05	Acute myeloid leukemia	-6.2	31.604	12.20	55	5	6.172	2.67401	207|2322|5292|5295|5467	AKT1|FLT3|PIM1|PIK3R1|PPARD	-5.09	1	0	0	-14.89	39.60618847

766	221		6766	94	229			84	8			266				72	
-6.22726	hsa05160	Hepatitis C	-6.22726	14.39988	9.384161	169	7	8.641975	3.122032	207|1017|1021|1956|2932|5295|10062	AKT1|CDK2|CDK6|EGFR|GSK3B|PIK3R1|NR1H3	-5.06038	1	0	0	-14.8972	39.60618847
-6.15094	hsa04923	Regulation of lipolysis in adipocytes	-6.15094	29.9702	11.861198	58	5	6.17284	2.674018	134|207|3643|5295|5743	ADORA1|AKT1|INSR|PIK3R1|PTGS2	-5.00755	1	0	0	-14.8972	39.60618847
-6.15094	hsa05223	Non-small cell lung cancer	-6.15094	29.9702	11.861198	58	5	6.17284	2.674018	207|238|1021|1956|5295	AKT1|ALK|CDK6|EGFR|PIK3R1	-5.00755	1	0	0	-14.8972	39.60618847
-6.15094	ko05223	Non-small cell lung cancer	-6.15094	29.9702	11.861198	58	5	6.17284	2.674018	207|238|1021|1956|5295	AKT1|ALK|CDK6|EGFR|PIK3R1	-5.00755	1	0	0	-14.8972	39.60618847
-6.08188	hsa04014	Ras signaling pathway	-6.08188	10.73836	8.45641	259	8	9.876543	3.314965	207|1956|2322|3480|3643|3791|4233|5295	AKT1|EGFR|FLT3|IGF1R|INSR|KDR|MET|PIK3R1	-4.94603	1	0	0	-14.8972	39.60618847
-6.04316	ko05202	Transcriptional misregulation in cancer	-6.04316	13.51989	9.050514	180	7	8.641975	3.122032	2322|3480|4233|4314|4318|4353|5747	FLT3|IGF1R|MET|MMP3|MMP9|MPO|PTK2	-4.91474	8	1	1	-6.04316	13.51989026
-6.03256	hsa04931	insulin resistance	-6.03256	18.45952	9.988233	113	6	7.407407	2.909904	207|2932|3643|5295|5770|10062	AKT1|GSK3B|INSR|PIK3R1|PTPN1|NR1H3	-4.91144	1	0	0	-14.8972	39.60618847
-5.93565	hsa05214	Glioma	-5.93565	27.16049	11.25315	64	5	6.17284	2.674018	207|1021|1956|3480|5295	AKT1|CDK6|EGFR|IGF1R|PIK3R1	-4.82877	1	0	0	-14.8972	39.60618847
-5.93565	ko05214	Glioma	-5.93565	27.16049	11.25315	64	5	6.17284	2.674018	207|1021|1956|3480|5295	AKT1|CDK6|EGFR|IGF1R|PIK3R1	-4.82877	1	0	0	-14.8972	39.60618847
-5.92217	ko04071	Sphingolipid signaling pathway	-5.92217	17.67734	9.750425	118	6	7.407407	2.909904	134|140|207|1509|4363|5295	ADORA1|ADORA3|AKT1|CTSD|ABCC1|PIK3R1	-4.82224	1	0	0	-14.8972	39.60618847

-5.90187	ko05230	Central carbon metabolism in cancer	-5.90187	26.74264	11.159778	65	5	6.17284	2.674018	207|1956|2322|4233|5295	AKT1|EGFR|FLT3|MET|PIK3R1	-4.81433	1	0	0	-14.8972	39.60618847
-5.90069	hsa04066	HIF-1 signaling pathway	-5.90069	17.52879	9.70461	119	6	7.40407	2.909904	207|1956|3480|3643|4843|5295	AKT1|EGFR|IGF1R|INSR|NOS2|PIK3R1	-4.81433	1	0	0	-14.8972	39.60618847
-5.87941	ko04152	AMPK signaling pathway	-5.87941	17.38272	9.659348	120	6	7.40407	2.909904	207|1080|3156|3480|3643|5295	AKT1|CFTR|HMGCR|IGF1R|INSR|PIK3R1	-4.79968	1	0	0	-14.8972	39.60618847
-5.79612	hsa04110	Cell cycle	-5.79612	16.82198	9.483618	124	6	7.40407	2.909904	891|983|1017|1021|2932|5347	CCNB1|CDK1|CDK2|CDK6|GSK3B|PLK1	-4.74195	4	0	0	-8.21522	25.34979424
-5.79612	hsa04114	Oocyte meiosis	-5.79612	16.82198	9.483618	124	6	7.40407	2.909904	367|891|983|1017|3480|5347	AR|CCNB1|CDK1|CDK2|IGF1R|PLK1	-4.74195	4	0	0	-8.21522	25.34979424
-5.79612	ko04110	Cell cycle	-5.79612	16.82198	9.483618	124	6	7.40407	2.909904	891|983|1017|1021|2932|5347	CCNB1|CDK1|CDK2|CDK6|GSK3B|PLK1	-4.74195	4	0	0	-8.21522	25.34979424
-5.79612	ko04114	Oocyte meiosis	-5.79612	16.82198	9.483618	124	6	7.40407	2.909904	367|891|983|1017|3480|5347	AR|CCNB1|CDK1|CDK2|IGF1R|PLK1	-4.74195	4	0	0	-8.21522	25.34979424
-5.72498	hsa01523	Antifolate resistance	-5.72498	44.85862	13.122257	31	4	4.938272	2.407398	239|4363|7298|9429	ALOX12|ABCC1|TYMS|ABCG2	-4.6873	9	1	1	-5.72498	44.85862206
-5.72498	ko01523	Antifolate resistance	-5.72498	44.85862	13.122257	31	4	4.938272	2.407398	239|4363|7298|9429	ALOX12|ABCC1|TYMS|ABCG2	-4.6873	9	0	0	-5.72498	44.85862206
-5.72325	ko05169	Epstein-Barr virus infection	-5.72325	12.10736	8.488062	201	7	8.641975	3.122032	207|983|1017|1457|2932|5295|6850	AKT1|CDK1|CDK2|CSNK2A1|GSK3B|PIK3R1|SYK	-4.6873	1	0	0	-14.8972	39.60618847
-5.71	hsa04	Sphingolipid signaling pathway	-5.7	16.296	9.315	128	6	7.407	2.90990	134|140|207|1509|4363|5295	ADORA1|ADORA3|AKT1|CTSD|ABCC1|PIK3R1	-4.68	1	0	0	-14.89	39.60618847

567	071		1567	3	894			407	4			588				72	
-5.71009	ko04976	Bile secretion	-5.71009	24.4827	10.64068	71	5	6.17284	2.674018	760|1080|3156|5243|9429	CA2|CFTR|HMGCR|ABCB1|ABCG2	-4.68588	10	1	1	-5.71009	31.60493827
-5.69597	hsa04152	AMPK signaling pathway	-5.69597	16.16997	9.275141	129	6	7.407407	2.909904	207|1080|3156|3480|3643|5295	AKT1|CFTR|HMGCR|IGF1R|INSR|PIK3R1	-4.67751	1	0	0	-14.8972	39.60618847
-5.68053	hsa05202	Transcriptional misregulation in cancer	-5.68053	11.92931	8.414548	204	7	8.641975	3.122032	2322|3480|4233|4314|4318|4353|5747	FLT3|IGF1R|MET|MMP3|MMP9|MPO|PTK2	-4.66775	8	0	0	-6.04316	13.51989026
-5.61661	hsa05169	Epstein-Barr virus infection	-5.61661	9.301788	7.751204	299	8	9.876543	3.314965	207|983|1017|1021|1457|2932|5295|6850	AKT1|CDK1|CDK2|CDK6|CSNK2A1|GSK3B|PIK3R1|SYK	-4.60943	1	0	0	-14.8972	39.60618847
-5.59158	hsa04976	Bile secretion	-5.59158	23.17695	10.32894	75	5	6.17284	2.674018	760|1080|3156|5243|9429	CA2|CFTR|HMGCR|ABCB1|ABCG2	-4.58993	10	0	0	-5.71009	31.60493827
-5.50702	hsa05230	Central carbon metabolism in cancer	-5.50702	22.28553	10.11063	78	5	6.17284	2.674018	207|1956|2322|4233|5295	AKT1|EGFR|FLT3|MET|PIK3R1	-4.51083	1	0	0	-14.8972	39.60618847
-5.13039	ko04657	IL-17 signaling pathway	-5.13039	18.69109	9.178219	93	5	6.17284	2.674018	2932|4314|4318|4322|5743	GSK3B|MMP3|MMP9|MMP13|PTGS2	-4.1396	11	1	1	-5.13039	18.69109253
-5.10924	ko04022	cGMP-PKG signaling pathway	-5.10924	12.79709	8.112847	163	6	7.407407	2.909904	134|140|207|3643|4638|5294	ADORA1|ADORA3|AKT1|INSR|MYLK|PIK3CG	-4.12741	12	1	1	-5.10924	12.79709157
-5.1024	hsa02010	ABC transporters	-5.1024	31.60494	10.91193	44	4	4.938272	2.407398	1080|4363|5243|9429	CFTR|ABCC1|ABCB1|ABCG2	-4.12741	10	0	0	-5.71009	31.60493827
-5.1024	ko02010	ABC transporters	-5.1024	31.60494	10.91193	44	4	4.938272	2.407398	1080|4363|5243|9429	CFTR|ABCC1|ABCB1|ABCG2	-4.12741	10	0	0	-5.71009	31.60493827

-5.08512	hsa04657	IL-17 signaling pathway	-5.08512	18.2976	9.070377	95	5	6.17284	2.674018	2932|4314|4318|4322|5743	GSK3B|MMP3|MMP9|MMP13|PTGS2	-4.11527	11	0	0	-5.13039	18.69109253
-5.01953	hsa04932	Non-alcoholic fatty liver disease (NAFLD)	-5.01953	12.34276	7.943522	169	6	7.407407	2.909904	207|2932|3643|5295|5743|10062	AKT1|GSK3B|INSR|PIK3R1|PTGS2|NR1H3	-4.05476	1	0	0	-14.8972	39.60618847
-4.96163	hsa04022	cGMP-PKG signaling pathway	-4.96163	12.05738	7.835329	173	6	7.407407	2.909904	134|140|207|3643|4638|5294	ADORA1|ADORA3|AKT1|INSR|MYLK|PIK3CG	-4.00187	12	0	0	-5.10924	12.79709157
-4.90515	hsa05161	Hepatitis B	-4.90515	11.78489	7.730643	177	6	7.407407	2.909904	207|1017|1021|4318|5295|6714	AKT1|CDK2|CDK6|MMP9|PIK3R1|SRC	-3.95036	1	0	0	-14.8972	39.60618847
-4.87854	hsa05213	Endometrial cancer	-4.87854	27.81235	10.19177	50	4	4.938272	2.407398	207|1956|2932|5295	AKT1|EGFR|GSK3B|PIK3R1	-3.93351	1	0	0	-14.8972	39.60618847
-4.87854	ko05213	Endometrial cancer	-4.87854	27.81235	10.19177	50	4	4.938272	2.407398	207|1956|2932|5295	AKT1|EGFR|GSK3B|PIK3R1	-3.93351	1	0	0	-14.8972	39.60618847
-4.83648	hsa04062	Chemokine signaling pathway	-4.83648	11.46113	7.604424	182	6	7.407407	2.909904	207|2932|3577|5295|5747|6714	AKT1|GSK3B|CXCR1|PIK3R1|PTK2|SRC	-3.90244	1	0	0	-14.8972	39.60618847
-4.83648	ko04062	Chemokine signaling pathway	-4.83648	11.46113	7.604424	182	6	7.407407	2.909904	207|2932|3577|5295|5747|6714	AKT1|GSK3B|CXCR1|PIK3R1|PTK2|SRC	-3.90244	1	0	0	-14.8972	39.60618847
-4.83323	hsa05146	Amoebiasis	-4.83323	16.24553	8.486039	107	5	6.17284	2.674018	383|1645|4843|5295|5747	ARG1|AKR1C1|NOS2|PIK3R1|PTK2	-3.90244	13	1	1	-4.83323	20.85925926
-4.81362	ko04668	TNF signaling pathway	-4.81362	16.09511	8.441633	108	5	6.17284	2.674018	207|4314|4318|5295|5743	AKT1|MMP3|MMP9|PIK3R1|PTGS2	-3.88748	11	0	0	-5.13039	18.69109253
-4.68	hsa04	TNF signaling pathway	-4.6	15.115	8.146	115	5	6.172	2.67401	207|4314|4318|5295|5743	AKT1|MMP3|MMP9|PIK3R1|PTGS2	-3.76	11	0	0	-5.130	18.69109253

<table>
<tr><td>156</td><td>668</td><td></td><td>8156</td><td>41</td><td>571</td><td></td><td></td><td>84</td><td>8</td><td></td><td></td><td>001</td><td></td><td></td><td></td><td>39</td><td></td></tr>
<tr><td>-4.6099</td><td>hsa04726</td><td>Serotonergic synapse</td><td>-4.609099</td><td>14.60732</td><td>7.989319</td><td>119</td><td>5</td><td>6.17284</td><td>2.674018</td><td>239|240|246|351|5743</td><td>ALOX12|ALOX5|ALOX15|APP|PTGS2</td><td>-3.6929</td><td>5</td><td>0</td><td>0</td><td>-7.59584</td><td>33.64396655</td></tr>
<tr><td>-4.59308</td><td>ko05203</td><td>Viral carcinogenesis</td><td>-4.59308</td><td>10.37774</td><td>7.166313</td><td>201</td><td>6</td><td>7.407407</td><td>2.909904</td><td>983|1017|1021|5295|6714|6850</td><td>CDK1|CDK2|CDK6|PIK3R1|SRC|SYK</td><td>-3.68058</td><td>1</td><td>0</td><td>0</td><td>-14.8972</td><td>39.60618847</td></tr>
<tr><td>-4.54079</td><td>hsa04919</td><td>thyroid hormone signaling pathway</td><td>-4.54079</td><td>14.13229</td><td>7.839485</td><td>123</td><td>5</td><td>6.17284</td><td>2.674018</td><td>207|2099|2932|5295|6714</td><td>AKT1|ESR1|GSK3B|PIK3R1|SRC</td><td>-3.63274</td><td>1</td><td>0</td><td>0</td><td>-14.8972</td><td>39.60618847</td></tr>
<tr><td>-4.5057</td><td>hsa04213</td><td>Longevity regulating pathway - multiple species</td><td>-4.50577</td><td>22.42931</td><td>9.072493</td><td>62</td><td>4</td><td>4.938272</td><td>2.407398</td><td>207|3480|3643|5295</td><td>AKT1|IGF1R|INSR|PIK3R1</td><td>-3.60643</td><td>1</td><td>0</td><td>0</td><td>-14.8972</td><td>39.60618847</td></tr>
<tr><td>-4.5057</td><td>ko04213</td><td>Longevity regulating pathway - multiple species</td><td>-4.50577</td><td>22.42931</td><td>9.072493</td><td>62</td><td>4</td><td>4.938272</td><td>2.407398</td><td>207|3480|3643|5295</td><td>AKT1|IGF1R|INSR|PIK3R1</td><td>-3.60643</td><td>1</td><td>0</td><td>0</td><td>-14.8972</td><td>39.60618847</td></tr>
<tr><td>-4.47488</td><td>hsa05203</td><td>Viral carcinogenesis</td><td>-4.47488</td><td>9.885905</td><td>6.958571</td><td>211</td><td>6</td><td>7.407407</td><td>2.909904</td><td>983|1017|1021|5295|6714|6850</td><td>CDK1|CDK2|CDK6|PIK3R1|SRC|SYK</td><td>-3.57993</td><td>1</td><td>0</td><td>0</td><td>-14.8972</td><td>39.60618847</td></tr>
<tr><td>-4.46341</td><td>hsa04810</td><td>Regulation of actin cytoskeleton</td><td>-4.46341</td><td>9.839273</td><td>6.938561</td><td>212</td><td>6</td><td>7.407407</td><td>2.909904</td><td>1956|2147|4638|5295|5747|6714</td><td>EGFR|F2|MYLK|PIK3R1|PTK2|SRC</td><td>-3.57711</td><td>6</td><td>0</td><td>0</td><td>-7.00258</td><td>42.39686841</td></tr>
<tr><td>-4.46341</td><td>ko04810</td><td>Regulation of actin cytoskeleton</td><td>-4.46341</td><td>9.839273</td><td>6.938561</td><td>212</td><td>6</td><td>7.407407</td><td>2.909904</td><td>1956|2147|4638|5295|5747|6714</td><td>EGFR|F2|MYLK|PIK3R1|PTK2|SRC</td><td>-3.57711</td><td>6</td><td>0</td><td>0</td><td>-7.00258</td><td>42.39686841</td></tr>
<tr><td>-4.45772</td><td>ko04140</td><td>Autophagy - animal</td><td>-4.45772</td><td>13.58025</td><td>7.66174</td><td>128</td><td>5</td><td>6.17284</td><td>2.674018</td><td>207|1509|1612|3480|5295</td><td>AKT1|CTSD|DAPK1|IGF1R|PIK3R1</td><td>-3.57711</td><td>1</td><td>0</td><td>0</td><td>-14.8972</td><td>39.60618847</td></tr>
<tr><td>-4.45108</td><td>hsa05212</td><td>Pancreatic cancer</td><td>-4.45108</td><td>21.7284</td><td>8.916486</td><td>64</td><td>4</td><td>4.938272</td><td>2.407398</td><td>207|1021|1956|5295</td><td>AKT1|CDK6|EGFR|PIK3R1</td><td>-3.57711</td><td>1</td><td>0</td><td>0</td><td>-14.8972</td><td>39.60618847</td></tr>
</table>

<table>
<tr><td>–4.45108</td><td>ko05212</td><td>Pancreatic cancer</td><td>–4.45108</td><td>21.7284</td><td>8.916486</td><td>64</td><td>4</td><td>4.938272</td><td>2.407398</td><td>207|1021|1956|5295</td><td>AKT1|CDK6|EGFR|PIK3R1</td><td>–3.57711</td><td>1</td><td>0</td><td>0</td><td>–14.8972</td><td>39.60618847</td></tr>
<tr><td>–4.40954</td><td>ko05160</td><td>Hepatitis C</td><td>–4.40954</td><td>13.26925</td><td>7.559797</td><td>131</td><td>5</td><td>6.17284</td><td>2.674018</td><td>207|1956|2932|5295|10062</td><td>AKT1|EGFR|GSK3B|PIK3R1|NR1H3</td><td>–3.53965</td><td>1</td><td>0</td><td>0</td><td>–14.8972</td><td>39.60618847</td></tr>
<tr><td>–4.39375</td><td>hsa04140</td><td>Regulation of autophagy</td><td>–4.39375</td><td>13.16872</td><td>7.526555</td><td>132</td><td>5</td><td>6.17284</td><td>2.674018</td><td>207|1509|1612|3480|5295</td><td>AKT1|CTSD|DAPK1|IGF1R|PIK3R1</td><td>–3.5279</td><td>1</td><td>0</td><td>0</td><td>–14.8972</td><td>39.60618847</td></tr>
<tr><td>–4.34707</td><td>hsa04664</td><td>Fc epsilon RI signaling pathway</td><td>–4.34707</td><td>20.45025</td><td>8.624794</td><td>68</td><td>4</td><td>4.938272</td><td>2.407398</td><td>207|240|5295|6850</td><td>AKT1|ALOX5|PIK3R1|SYK</td><td>–3.49701</td><td>1</td><td>0</td><td>0</td><td>–14.8972</td><td>39.60618847</td></tr>
<tr><td>–4.34707</td><td>ko04664</td><td>Fc epsilon RI signaling pathway</td><td>–4.34707</td><td>20.45025</td><td>8.624794</td><td>68</td><td>4</td><td>4.938272</td><td>2.407398</td><td>207|240|5295|6850</td><td>AKT1|ALOX5|PIK3R1|SYK</td><td>–3.49701</td><td>1</td><td>0</td><td>0</td><td>–14.8972</td><td>39.60618847</td></tr>
<tr><td>–4.34707</td><td>hsa05120</td><td>Epithelial cell signaling in Helicobacter pylori infection</td><td>–4.34707</td><td>20.45025</td><td>8.624794</td><td>68</td><td>4</td><td>4.938272</td><td>2.407398</td><td>1956|3577|4233|6714</td><td>EGFR|CXCR1|MET|SRC</td><td>–3.49701</td><td>3</td><td>0</td><td>0</td><td>–8.8002</td><td>33.79972565</td></tr>
<tr><td>–4.34707</td><td>ko05120</td><td>Epithelial cell signaling in Helicobacter pylori infection</td><td>–4.34707</td><td>20.45025</td><td>8.624794</td><td>68</td><td>4</td><td>4.938272</td><td>2.407398</td><td>1956|3577|4233|6714</td><td>EGFR|CXCR1|MET|SRC</td><td>–3.49701</td><td>3</td><td>0</td><td>0</td><td>–8.8002</td><td>33.79972565</td></tr>
<tr><td>–4.32208</td><td>hsa04115</td><td>p53 signaling pathway</td><td>–4.32208</td><td>20.15387</td><td>8.555746</td><td>69</td><td>4</td><td>4.938272</td><td>2.407398</td><td>891|983|1017|1021</td><td>CCNB1|CDK1|CDK2|CDK6</td><td>–3.47971</td><td>4</td><td>0</td><td>0</td><td>–8.21522</td><td>25.34979424</td></tr>
<tr><td>–4.32208</td><td>ko04115</td><td>p53 signaling pathway</td><td>–4.32208</td><td>20.15387</td><td>8.555746</td><td>69</td><td>4</td><td>4.938272</td><td>2.407398</td><td>891|983|1017|1021</td><td>CCNB1|CDK1|CDK2|CDK6</td><td>–3.47971</td><td>4</td><td>0</td><td>0</td><td>–8.21522</td><td>25.34979424</td></tr>
<tr><td>–4.3016</td><td>hsa04910</td><td>Insulin signaling pathway</td><td>–4.3016</td><td>12.59617</td><td>7.334405</td><td>138</td><td>5</td><td>6.17284</td><td>2.674018</td><td>207|2932|3643|5295|5770</td><td>AKT1|GSK3B|INSR|PIK3R1|PTPN1</td><td>–3.46678</td><td>1</td><td>0</td><td>0</td><td>–14.8972</td><td>39.60618847</td></tr>
<tr><td>–4.30</td><td>ko04</td><td>Insulin signaling pathway</td><td>–4.3</td><td>12.596</td><td>7.334</td><td>138</td><td>5</td><td>6.172</td><td>2.67401</td><td>207|2932|3643|5295|5770</td><td>AKT1|GSK3B|INSR|PIK3R1|PTPN1</td><td>–3.46</td><td>1</td><td>0</td><td>0</td><td>–14.89</td><td>39.60618847</td></tr>
</table>

16	910		016	17	405			84	8			678				72	
-4.27324	hsa04662	B cell receptor signaling pathway	-4.27324	19.58616	8.421918	71	4	4.938272	2.407398	207|2932|5295|6850	AKT1|GSK3B|PIK3R1|SYK	-3.44584	1	0	0	-14.8972	39.60618847
-4.27324	ko04662	B cell receptor signaling pathway	-4.27324	19.58616	8.421918	71	4	4.938272	2.407398	207|2932|5295|6850	AKT1|GSK3B|PIK3R1|SYK	-3.44584	1	0	0	-14.8972	39.60618847
-4.15726	ko05100	Bacterial invasion of epithelial cells	-4.15726	18.2976	8.110047	76	4	4.938272	2.407398	4233|5295|5747|6714	MET|PIK3R1|PTK2|SRC	-3.33353	1	0	0	-14.8972	39.60618847
-4.14339	ko04932	Non-alcoholic fatty liver disease (NAFLD)	-4.14339	11.66625	7.011339	149	5	6.17284	2.674018	207|2932|3643|5295|10062	AKT1|GSK3B|INSR|PIK3R1|NR1H3	-3.3233	1	0	0	-14.8972	39.60618847
-4.11599	hsa04150	mTOR signaling pathway	-4.11599	11.51173	6.956236	151	5	6.17284	2.674018	207|2932|3480|3643|5295	AKT1|GSK3B|IGF1R|INSR|PIK3R1	-3.30308	1	0	0	-14.8972	39.60618847
-4.11599	ko04150	mTOR signaling pathway	-4.11599	11.51173	6.956236	151	5	6.17284	2.674018	207|2932|3480|3643|5295	AKT1|GSK3B|IGF1R|INSR|PIK3R1	-3.30308	1	0	0	-14.8972	39.60618847
-4.10244	ko04921	Oxytocin signaling pathway	-4.10244	11.436	6.929071	152	5	6.17284	2.674018	1956|4638|5294|5743|6714	EGFR|MYLK|PIK3CG|PTGS2|SRC	-3.29307	6	0	0	-7.00258	42.39686841
-3.98465	hsa04921	Oxytocin signaling pathway	-3.98465	10.79672	6.695493	161	5	6.17284	2.674018	1956|4638|5294|5743|6714	EGFR|MYLK|PIK3CG|PTGS2|SRC	-3.1788	6	0	0	-7.00258	42.39686841
-3.97325	hsa04144	Endocytosis	-3.97325	8.022792	6.11019	260	6	7.407407	2.909904	1956|3480|3577|3791|4233|6714	EGFR|IGF1R|CXCR1|KDR|MET|SRC	-3.17435	3	0	0	-8.8002	33.79972565
-3.97325	ko04144	Endocytosis	-3.97325	8.022792	6.11019	260	6	7.407407	2.909904	1956|3480|3577|3791|4233|6714	EGFR|IGF1R|CXCR1|KDR|MET|SRC	-3.17435	3	0	0	-8.8002	33.79972565

-3.96761	hsa05100	Bacterial invasion of epithelial cells	-3.96761	16.3602	7.617402	85	4	4.938272	2.407398	4233|5295|5747|6714	MET|PIK3R1|PTK2|SRC	-3.17214	1	0	0	-14.8972	39.60618847
-3.88385	hsa04060	Cytokine-cytokine receptor interaction	-3.88385	7.725652	5.964201	270	6	7.407407	2.909904	1956|2322|3558|3577|3791|4233	EGFR|FLT3|IL2|CXCR1|KDR|MET	-3.09516	3	0	0	-8.8002	33.79972565
-3.88385	ko04060	Cytokine-cytokine receptor interaction	-3.88385	7.725652	5.964201	270	6	7.407407	2.909904	1956|2322|3558|3577|3791|4233	EGFR|FLT3|IL2|CXCR1|KDR|MET	-3.09516	3	0	0	-8.8002	33.79972565
-3.815	hsa04360	Axon guidance	-3.815	9.932981	6.366681	175	5	6.17284	2.674018	2932|4233|5295|5747|6714	GSK3B|MET|PIK3R1|PTK2|SRC	-3.033	1	0	0	-14.8972	39.60618847
-3.815	ko04360	Axon guidance	-3.815	9.932981	6.366681	175	5	6.17284	2.674018	2932|4233|5295|5747|6714	GSK3B|MET|PIK3R1|PTK2|SRC	-3.033	1	0	0	-14.8972	39.60618847
-3.78054	ko04064	NF-kappa B signaling pathway	-3.78054	14.63808	7.151427	95	4	4.938272	2.407398	142|1457|5743|6850	PARP1|CSNK2A1|PTGS2|SYK	-3.00184	14	1	1	-3.78054	14.63807667
-3.76925	ko05152	Tuberculosis	-3.76925	9.711015	6.279484	179	5	6.17284	2.674018	207|1509|4843|6714|6850	AKT1|CTSD|NOS2|SRC|SYK	-2.99383	15	1	1	-3.76925	9.711014553
-3.763	ko05146	Amoebiasis	-3.763	14.4856	7.108718	96	4	4.938272	2.407398	383|4843|5295|5747	ARG1|NOS2|PIK3R1|PTK2	-2.99084	13	0	0	-4.83323	20.85925926
-3.71154	ko04933	AGE-RAGE signaling pathway in diabetic complications	-3.71154	14.04664	6.984334	99	4	4.938272	2.407398	207|4313|5292|5295	AKT1|MMP2|PIM1|PIK3R1	-2.9426	1	0	0	-14.8972	39.60618847
-3.66173	ko05142	Chagas disease (American trypanosomiasis)	-3.66173	13.6335	6.865241	102	4	4.938272	2.407398	207|3558|4843|5295	AKT1|IL2|NOS2|PIK3R1	-2.89599	1	0	0	-14.8972	39.60618847
-3.64	hsa04	T cell receptor signaling pathway	-3.6	13.501	6.826	103	4	4.938	2.40739	207|2932|3558|5295	AKT1|GSK3B|IL2|PIK3R1	-2.88	1	0	0	-14.89	39.60618847

547	660		4547	14	654			272	8			607				72	
−3.64547	ko04660	T cell receptor signaling pathway	−3.64547	13.50114	6.826654	103	4	4.938272	2.407398	207|2932|3558|5295	AKT1|GSK3B|IL2|PIK3R1	−2.88607	1	0	0	−14.8972	39.60618847
−3.62797	hsa05152	Tuberculosis	−3.62797	9.053498	6.014019	192	5	6.17284	2.674018	207|1509|4843|6714|6850	AKT1|CTSD|NOS2|SRC|SYK	−2.87171	15	0	0	−3.76925	9.711014553
−3.5821	hsa04933	AGE-RAGE signaling pathway in diabetic complications	−3.5821	12.99642	6.677506	107	4	4.938272	2.407398	207|4313|5292|5295	AKT1|MMP2|PIM1|PIK3R1	−2.83511	1	0	0	−14.8972	39.60618847
−3.5821	hsa05142	Chagas disease (American trypanosomiasis)	−3.5821	12.99642	6.677506	107	4	4.938272	2.407398	207|3558|4843|5295	AKT1|IL2|NOS2|PIK3R1	−2.83511	1	0	0	−14.8972	39.60618847
−3.5821	hsa04064	NF-kappa B signaling pathway	−3.5821	12.99642	6.677506	107	4	4.938272	2.407398	142|1457|5743|6850	PARP1|CSNK2A1|PTGS2|SYK	−2.83511	14	0	0	−3.78054	14.63807667
−3.55136	hsa04211	Longevity regulating pathway	−3.55136	12.75796	6.605888	109	4	4.938272	2.407398	207|3480|3643|5295	AKT1|IGF1R|INSR|PIK3R1	−2.80742	1	0	0	−14.8972	39.60618847
−3.49166	ko05145	Toxoplasmosis	−3.49166	12.30635	6.468129	113	4	4.938272	2.407398	207|240|4843|5294	AKT1|ALOX5|NOS2|PIK3CG	−2.75075	12	0	0	−5.10924	12.79709157
−3.4771	hsa04670	Leukocyte transendothelial migration	−3.4771	12.1984	6.434772	114	4	4.938272	2.407398	4313|4318|5295|5747	MMP2|MMP9|PIK3R1|PTK2	−2.74218	8	0	0	−6.04316	13.51989026
−3.4771	ko04670	Leukocyte transendothelial migration	−3.4771	12.1984	6.434772	114	4	4.938272	2.407398	4313|4318|5295|5747	MMP2|MMP9|PIK3R1|PTK2	−2.74218	8	0	0	−6.04316	13.51989026
−3.46267	hsa04725	Cholinergic synapse	−3.46267	12.09232	6.401829	115	4	4.938272	2.407398	43|207|5294|5295	ACHE|AKT1|PIK3CG|PIK3R1	−2.73071	1	0	0	−14.8972	39.60618847

−3.44837	hsa05145	Toxoplasmosis	−3.44837	11.98808	6.369292	116	4	4.938272	2.407398	207|240|4843|5294	AKT1|ALOX5|NOS2|PIK3CG	−2.71936	12	0	0	−5.10924	12.79709157
−3.38974	hsa00330	Arginine and proline metabolism	−3.38974	20.85926	7.5488	50	3	3.703704	2.098362	383|4843|4953	ARG1|NOS2|ODC1	−2.66655	13	0	0	−4.83323	20.85925926
−3.38974	ko00330	Arginine and proline metabolism	−3.38974	20.85926	7.5488	50	3	3.703704	2.098362	383|4843|4953	ARG1|NOS2|ODC1	−2.66655	13	0	0	−4.83323	20.85925926
−3.1638	hsa04210	Apoptosis	−3.1638	10.07694	5.741027	138	4	4.938272	2.407398	142|207|1509|5295	PARP1|AKT1|CTSD|PIK3R1	−2.44637	1	0	0	−14.8972	39.60618847
−3.1638	ko04210	Apoptosis	−3.1638	10.07694	5.741027	138	4	4.938272	2.407398	142|207|1509|5295	PARP1|AKT1|CTSD|PIK3R1	−2.44637	1	0	0	−14.8972	39.60618847
−3.15669	hsa05210	Colorectal cancer	−3.15669	17.38272	6.822895	60	3	3.703704	2.098362	207|2932|5295	AKT1|GSK3B|PIK3R1	−2.44494	1	0	0	−14.8972	39.60618847
−3.15669	ko05210	Colorectal cancer	−3.15669	17.38272	6.822895	60	3	3.703704	2.098362	207|2932|5295	AKT1|GSK3B|PIK3R1	−2.44494	1	0	0	−14.8972	39.60618847
−3.15206	ko04550	Signaling pathways regulating pluripotency of stem cells	−3.15206	10.00444	5.715874	139	4	4.938272	2.407398	207|2932|3480|5295	AKT1|GSK3B|IGF1R|PIK3R1	−2.44312	1	0	0	−14.8972	39.60618847
−3.07239	hsa04371	Apelin signaling pathway	−3.07239	9.524776	5.546668	146	4	4.938272	2.407398	207|4638|4843|5294	AKT1|MYLK|NOS2|PIK3CG	−2.36624	12	0	0	−5.10924	12.79709157
−3.05518	hsa05211	Renal cell carcinoma	−3.05518	16.04558	6.522461	65	3	3.703704	2.098362	207|4233|5295	AKT1|MET|PIK3R1	−2.35456	1	0	0	−14.8972	39.60618847
−3.05	ko05	Renal cell carcinoma	−3.0	16.045	6.522	65	3	3.703	2.09836	207|4233|5295	AKT1|MET|PIK3R1	−2.35	1	0	0	−14.89	39.60618847

518	211		5518	58	461			704	2			456				72	
-2.9968	hsa04550	Signaling pathways regulating pluripotency of stem cells	-2.9968	9.089002	5.388497	153	4	4.938272	2.407398	207|2932|3480|5295	AKT1|GSK3B|IGF1R|PIK3R1	-2.29892	1	0	0	-14.8972	39.60618847
-2.96556	hsa04630	Jak-STAT signaling pathway	-2.96556	8.914213	5.323783	156	4	4.938272	2.407398	207|3558|5292|5295	AKT1|IL2|PIM1|PIK3R1	-2.27311	1	0	0	-14.8972	39.60618847
-2.96556	ko04630	Jak-STAT signaling pathway	-2.96556	8.914213	5.323783	156	4	4.938272	2.407398	207|3558|5292|5295	AKT1|IL2|PIM1|PIK3R1	-2.27311	1	0	0	-14.8972	39.60618847
-2.94378	hsa05220	Chronic myeloid leukemia	-2.94378	14.68962	6.203156	71	3	3.703704	2.098362	207|1021|5295	AKT1|CDK6|PIK3R1	-2.2567	1	0	0	-14.8972	39.60618847
-2.94378	ko05220	Chronic myeloid leukemia	-2.94378	14.68962	6.203156	71	3	3.703704	2.098362	207|1021|5295	AKT1|CDK6|PIK3R1	-2.2567	1	0	0	-14.8972	39.60618847
-2.90887	hsa01524	Platinum drug resistance	-2.90887	14.28716	6.10522	73	3	3.703704	2.098362	207|5295|7153	AKT1|PIK3R1|TOP2A	-2.22708	1	0	0	-14.8972	39.60618847
-2.90887	ko01524	Platinum drug resistance	-2.90887	14.28716	6.10522	73	3	3.703704	2.098362	207|5295|7153	AKT1|PIK3R1|TOP2A	-2.22708	1	0	0	-14.8972	39.60618847
-2.87496	ko04971	Gastric acid secretion	-2.87496	13.90617	6.011062	75	3	3.703704	2.098362	760|1080|4638	CA2|CFTR|MYLK	-2.19579	10	0	0	-5.71009	31.60493827
-2.85836	hsa04971	Gastric acid secretion	-2.85836	13.7232	5.965322	76	3	3.703704	2.098362	760|1080|4638	CA2|CFTR|MYLK	-2.18181	10	0	0	-5.71009	31.60493827
-2.76345	ko05204	Chemical carcinogenesis	-2.76345	12.71906	5.707908	82	3	3.703704	2.098362	1545|1646|5743	CYP1B1|AKR1C2|PTGS2	-2.08949	2	0	0	-13.7121	62.57777778

-2.67571	hsa04540	Gap junction	-2.67571	11.85185	5.476068	88	3	3.703704	2.098362	983|1956|6714	CDK1|EGFR|SRC	-2.00689	6	0	0	-7.00258	42.39686841
-2.67571	ko04540	Gap junction	-2.67571	11.85185	5.476068	88	3	3.703704	2.098362	983|1956|6714	CDK1|EGFR|SRC	-2.00689	6	0	0	-7.00258	42.39686841
-2.66172	hsa05204	Chemical carcinogenesis	-2.66172	11.71868	5.439611	89	3	3.703704	2.098362	1545|1646|5743	CYP1B1|AKR1C2|PTGS2	-1.99544	2	0	0	-13.7121	62.57777778
-2.63422	hsa04666	Fc gamma R-mediated phagocytosis	-2.63422	11.46113	5.368416	91	3	3.703704	2.098362	207|5295|6850	AKT1|PIK3R1|SYK	-1.973	1	0	0	-14.8972	39.60618847
-2.63422	ko04666	Fc gamma R-mediated phagocytosis	-2.63422	11.46113	5.368416	91	3	3.703704	2.098362	207|5295|6850	AKT1|PIK3R1|SYK	-1.973	1	0	0	-14.8972	39.60618847
-2.62072	hsa04912	GnRH signaling pathway	-2.62072	11.33655	5.333647	92	3	3.703704	2.098362	1956|4313|6714	EGFR|MMP2|SRC	-1.96448	6	0	0	-7.00258	42.39686841
-2.62072	ko04912	GnRH signaling pathway	-2.62072	11.33655	5.333647	92	3	3.703704	2.098362	1956|4313|6714	EGFR|MMP2|SRC	-1.96448	6	0	0	-7.00258	42.39686841
-2.5873	ko04024	cAMP signaling pathway	-2.5873	7.02332	4.568196	198	4	4.938272	2.407398	134|207|1080|5295	ADORA1|AKT1|CFTR|PIK3R1	-1.93354	1	0	0	-14.8972	39.60618847
-2.53041	ko05231	Choline metabolism in cancer	-2.53041	10.53498	5.104416	99	3	3.703704	2.098362	207|1956|5295	AKT1|EGFR|PIK3R1	-1.87911	1	0	0	-14.8972	39.60618847
-2.47004	hsa04750	inflammatory mediator regulation of trp channels	-2.47004	10.02849	4.954265	104	3	3.703704	2.098362	239|5295|6714	ALOX12|PIK3R1|SRC	-1.82119	16	1	1	-2.47004	10.02849003
-2.43	hsa05	Choline metabolism in cancer	-2.4	9.7473	4.868	107	3	3.703	2.09836	207|1956|5295	AKT1|EGFR|PIK3R1	-1.78	1	0	0	-14.89	39.60618847

531	231		3531	17	974			704	2			889				72	
-2.38213	hsa04024	cAMP signaling pathway	-2.382813	6.153174	4.177561	226	4	4.938272	2.407398	134|207|1080|5295	ADORA1|AKT1|CFTR|PIK3R1	-1.73813	1	0	0	-14.8972	39.60618847
-2.30628	hsa04722	Neurotrophin signaling pathway	-2.330628	8.764395	4.558738	119	3	3.703704	2.098362	207|2932|5295	AKT1|GSK3B|PIK3R1	-1.66708	1	0	0	-14.8972	39.60618847
-2.30628	ko04722	Neurotrophin signaling pathway	-2.330628	8.764395	4.558738	119	3	3.703704	2.098362	207|2932|5295	AKT1|GSK3B|PIK3R1	-1.66708	1	0	0	-14.8972	39.60618847
-2.19995	ko04380	Osteoclast differentiation	-2.199995	8.022792	4.310526	130	3	3.703704	2.098362	207|5295|6850	AKT1|PIK3R1|SYK	-1.56312	1	0	0	-14.8972	39.60618847
-2.19232	hsa05166	HTLV-I infection	-2.192232	5.432099	3.826101	256	4	4.938272	2.407398	207|2932|3558|5295	AKT1|GSK3B|IL2|PIK3R1	-1.56022	1	0	0	-14.8972	39.60618847
-2.19232	ko05166	HTLV-I infection	-2.192232	5.432099	3.826101	256	4	4.938272	2.407398	207|2932|3558|5295	AKT1|GSK3B|IL2|PIK3R1	-1.56022	1	0	0	-14.8972	39.60618847
-2.12867	hsa04380	Osteoclast differentiation	-2.128667	7.557703	4.147649	138	3	3.703704	2.098362	207|5295|6850	AKT1|PIK3R1|SYK	-1.49891	1	0	0	-14.8972	39.60618847
-2.08641	hsa04310	Wnt signaling pathway	-2.086641	7.293447	4.052342	143	3	3.703704	2.098362	1457|2932|5467	CSNK2A1|GSK3B|PPARD	-1.46129	17	1	1	-2.08641	7.293447293
-2.08641	ko04310	Wnt signaling pathway	-2.086641	7.293447	4.052342	143	3	3.703704	2.098362	1457|2932|5467	CSNK2A1|GSK3B|PPARD	-1.46129	17	0	0	-2.08641	7.293447293
-2.07419	hsa04080	Neuroactive ligand-receptor interaction	-2.074419	5.020279	3.611492	277	4	4.938272	2.407398	134|140|2147|2908	ADORA1|ADORA3|F2|NR3C1	-1.45367	18	1	1	-2.07419	5.020279003

-2.07419	ko04080	Neuroactive ligand-receptor interaction	-2.07419	5.020279	3.611492	277	4	4.938272	2.407398	134|140|2147|2908	ADORA1|ADORA3|F2|NR3C1	-1.45367	18	0	0	-2.07419	5.020279003
